# Sulforaphane suppresses metastasis of triple-negative breast cancer cells by targeting the RAF/MEK/ERK pathway

**DOI:** 10.1038/s41523-022-00402-4

**Published:** 2022-03-24

**Authors:** Ying Zhang, Qian Lu, Nan Li, Ming Xu, Tatsuo Miyamoto, Jing Liu

**Affiliations:** 1 Department of Molecular and Cellular Physiology, Yamaguchi University, Graduate School of Medicine, 1-1-1 Minami-Kogushi, Ube, Yamaguchi, 755-8505 Japan; 2 Department of Gastroenterological, Breast and Endocrine Surgery, Yamaguchi University, Graduate School of Medicine, 1-1-1 Minami-Kogushi, Ube, Yamaguchi, 755-8505 Japan; 3 College of Pharmacy, Dalian Medical University, No.9 West Section Lvshun South Road, Dalian, 116044 China

**Keywords:** Focal adhesion, Breast cancer

## Abstract

Breast cancer metastasis is the main cause of cancer death in women, so far, no effective treatment has inhibited breast cancer metastasis. Sulforaphane (SFN), a natural compound derived from broccoli, has shown potential health benefits in many cancers. However, research on breast cancer metastasis is still insufficient. Here, we showed that SFN, including its two isomers of R-SFN and S-SFN, significantly inhibited TGF-β1-induced migration and invasion in breast cancer cells. Proteomic and phosphoproteomic analysis showed that SFN affected the formation of the cytoskeleton. Subsequent experiments confirmed that SFN significantly inhibited TGF-β1-induced actin stress fiber formation and the expression of actin stress fiber formation-associated proteins, including paxillin, IQGAP1, FAK, PAK2, and ROCK. Additionally, SFN is directly bound to RAF family proteins (including ARAF, BRAF, and CRAF) and inhibited MEK and ERK phosphorylation. These in vitro results indicate that SFN targets the RAF/MEK/ERK signaling pathway to inhibit the formation of actin stress fibers, thereby inhibiting breast cancer cell metastasis.

## Introduction

Breast cancer metastasis is the main cause of cancer death in women, especially triple-negative breast cancer (TNBC) is more aggressive and has a poorer prognosis than other types of breast cancer. So far, no effective treatment has inhibited breast cancer metastasis. Due to the high rate of metastasis, the mortality of young women (<45 years) with breast cancer, especially with TNBC type, has increased significantly recently^1–4^. The metastatic potential of cancer cells is coordinated through cell-cell adhesion, cell-matrix adhesion, protrusion, and contraction, all of which require proper cytoskeleton regulation and its dynamics^5–8^. Therefore, inhibiting the formation of the cytoskeleton is a promising strategy for the treatment of cancer metastasis.

Actin stress fibers are a higher-order cytoskeletal structure composed of cross-linked actin filament bundles, which play a vital role in cell migration and invasion. The actin stress fiber organization promotes cell stiffening and proliferation of preinvasive breast cancer cells^9^. Numerous studies have demonstrated that RhoA and Rho-kinase (ROCK) signaling pathway is involved in actin stress fiber formation^10–14^. Actin stress fibers connect to focal adhesions and focal adhesion-associated proteins, such as paxillin^15,16^, focal adhesion kinase (FAK)^17,18^, and Ras GTPase-activating-like protein (IQGAP1)^19,20^, which are key regulators of actin cytoskeleton dynamics.

RAF is a serine/threonine-protein kinase that can directly phosphorylate or promote protein phosphorylation by activating the downstream mitogen-activated protein kinase (MEK)/extracellular signal-regulated kinase (ERK) signaling pathway^21,22^. The RAF/MEK/ERK cascade is involved in regulating the development and metastasis of multiple types of cancer, including lung cancer, liver cancer, and breast cancer^23–26^. RAF family members include ARAF, BRAF, and CRAF. Recent studies revealed that a dimerization-dependent mechanism drives ARAF catalytic activation and dimerization of ARAF promotes MAPK pathway activation and cell migration^27^.

Sulforaphane (SFN), a natural compound derived from broccoli/broccoli sprouts, has been proved to exhibit chemoprotective effects for various cancers, including prostate cancer, lung cancer, and colon cancer^28–30^. Multiple action mechanisms involved in the anticancer properties of SFN have been reported, including induction of apoptosis^31,32^, cell cycle arrest^33,34^, and inhibition of angiogenesis and metastasis^35,36^. Although SFN has shown potential health benefits in many cancers, there are few studies on the involvement of SFN in breast cancer metastasis. Whether SFN can inhibit breast cancer metastasis and its mechanism are still unclear.

In the present study, we aimed to investigate whether SFN has an inhibitory effect on migration and invasion of breast cancer cells. We show that SFN and its two isomers (R-SFN and S-SFN) inhibited transforming growth factor-β1 (TGF-β1)-induced migration and invasion in human TNBC cells. Further, the mechanism by which SFN suppresses migration and invasion was also investigated.

## Results

### Effect of SFN on the viability of MDA-MB-231 and MDA-MB-157 cells

**Fig. 1:**
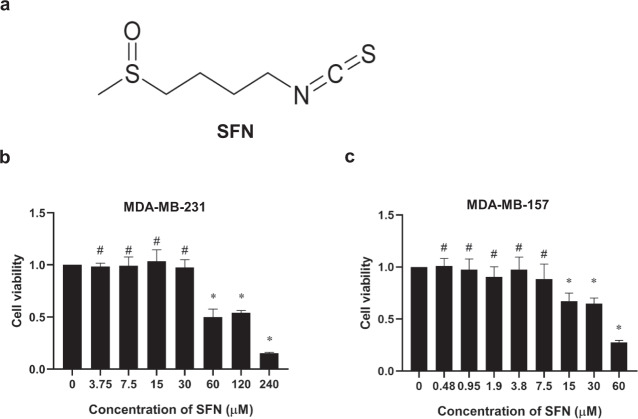
The chemical structure of SFN and its effects on the viability of MDA-MB-231 and MDA-MB-157 cells. **a** The chemical structure of SFN. **b** Effects of SFN at different concentrations on the viability of MDA-MB-231 cells. Data shown are mean ± SEM. *n* = 5, ^*^P < 0.05; ^#^*P* > 0.05. **c** Effects of SFN at different concentrations on the viability of MDA-MB-157 cells. Data shown are mean ± SEM. *n* = 5, ^*^P < 0.05; ^#^*P* > 0.05.

Many studies have demonstrated that TGF-β1 induced the migration and invasion of breast cancer cells^37–40^. However, the precise mechanism underlying TGF-β1-induced migration and invasion in breast cancer has not been fully elucidated. To determine the effect of SFN on TGF-β1-induced migration and invasion in breast cancer cells, we first measured the viability of cells after treatment with SFN (the chemical structure of SFN is shown in Fig. 1a) by using the CCK-8 assay. The previous report showed that the IC_50_ value of SFN varies greatly in the breast cancer cell lines^41^. Therefore, we treated the cells with various concentrations of SFN from 0.5 to 240 μM for 24 h and then cell viability was measured. As shown in Fig. 1b, in MDA-MB-231 cells, SFN has no significant effect on cell viability when the concentration is below 30 µM. However, at concentrations of 60, 120, and 240 µM SFN, significant toxicity of SFN was observed. In MDA-MB-157 cells, SFN showed no significant effect on cell viability when the concentration was lower than 7.5 µM (Fig. 1c). It is well known that when the compound produces a toxic response to cells, meaning that the compound can activate many intracellular signaling pathways. Avoiding toxicity to cells is crucial to selectively examine anti-migratory effects and mechanism(s) of SFN. Therefore, we used the concentration of 7.5, 15, and 30 µM for MDA-MB-231 cells and 1.9, 3.8, and 7.5 µM for MDA-MB-157 cells to study the effect of SFN on the migration and invasion in two human TNBC cell lines of MDA-MB-231 and MDA-MB-157.

### SFN inhibits migration and invasion of MDA-MB-231 and MDA-MB-157 cells

**Fig. 2:**
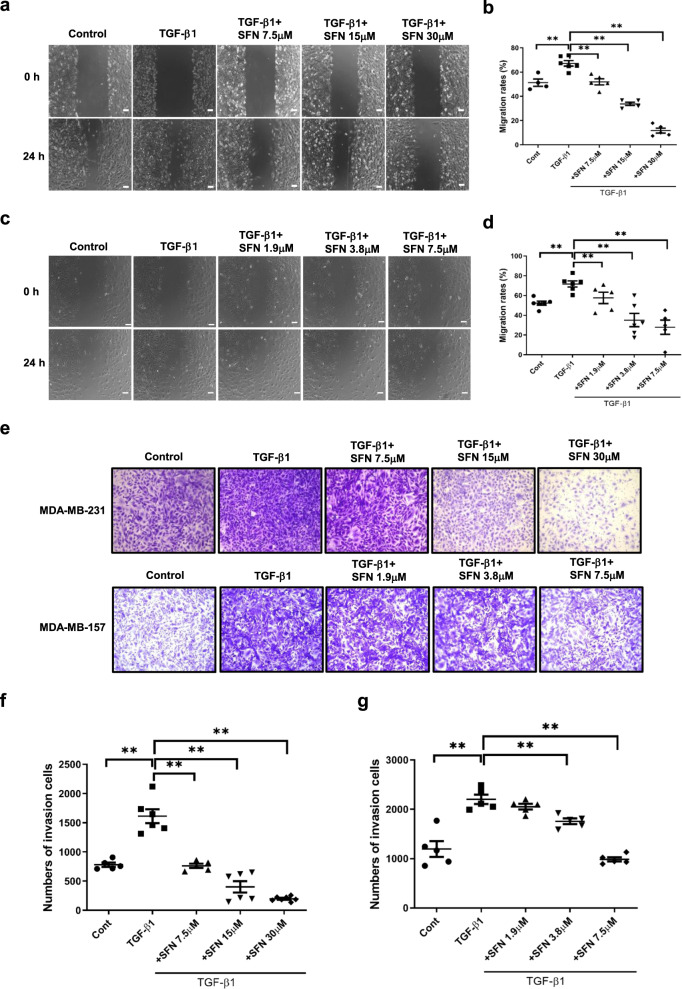
SFN inhibits TGF-β1-induced migration and invasion of MDA-MB-231 and MDA-MB-157 cells. **a**, **c** Representative images showing that wound width of MDA-MB-231 (**a**) and MDA-MB-157 (**c**) cells in the presence of SFN at different concentrations (7.5, 15, and 30 μM for MDA-MB-231 cells and 1.9, 3.8, and 7.5 μM for MDA-MB-157 cells) at 24 h by wound healing assay. Scale bar = 100 μm. **b**, **d** Statistical assay showing the effects of SFN on TGF-β1-induced migration of MDA-MB-231 (**b**) and MDA-MB-157 (**d**) cells at different concentrations at 24 h. Data shown are mean ± SEM. *n* = 5, ^**^P < 0.05. **e** Representative images showing that invasion of MDA-MB-231 and MDA-MB-157 cells in the presence of SFN at different concentrations (7.5, 15, and 30 μM for MDA-MB-231 cells and 1.9, 3.8, and 7.5 μM for MDA-MB-157 cells) at 24 h by transwell assay. **f**, **g** Statistical assay showing the effects of SFN on TGF-β1-induced invasion of MDA-MB-231 (**f**) and MDA-MB-157 (**g**) cells at different concentrations at 24 h. Data shown are mean ± SEM. *n* = 5 or 6, ^**^P < 0.05.

To investigate whether SFN inhibits breast cancer metastasis, we first examined the effect of SFN on the migration of MDA-MB-231 cells using a wound healing assay and recorded the real-time migration. As shown in Fig. 2a, b, TGF-β1 induced remarkable migration. However, after being treated for 24 h with SFN at the concentration of 7.5, 15, and 30 μM, the number of MDA-MB-231 cells migrated to the wound was markedly reduced (Fig. 2a). Quantitative analysis showed that SFN significantly inhibited cell migration, and as the concentration increased, the inhibitory effect became stronger (Fig. 2b). Similarly, SFN also showed a significant inhibitory effect on TGF-β1-induced invasion of MDA-MB-231 cells (Fig. 2e, f). In addition, SFN also significantly inhibited TGF-β1-induced migration and invasion of MDA-MB-157 cells (Fig. 2c, d, e, g).

**Fig. 3:**
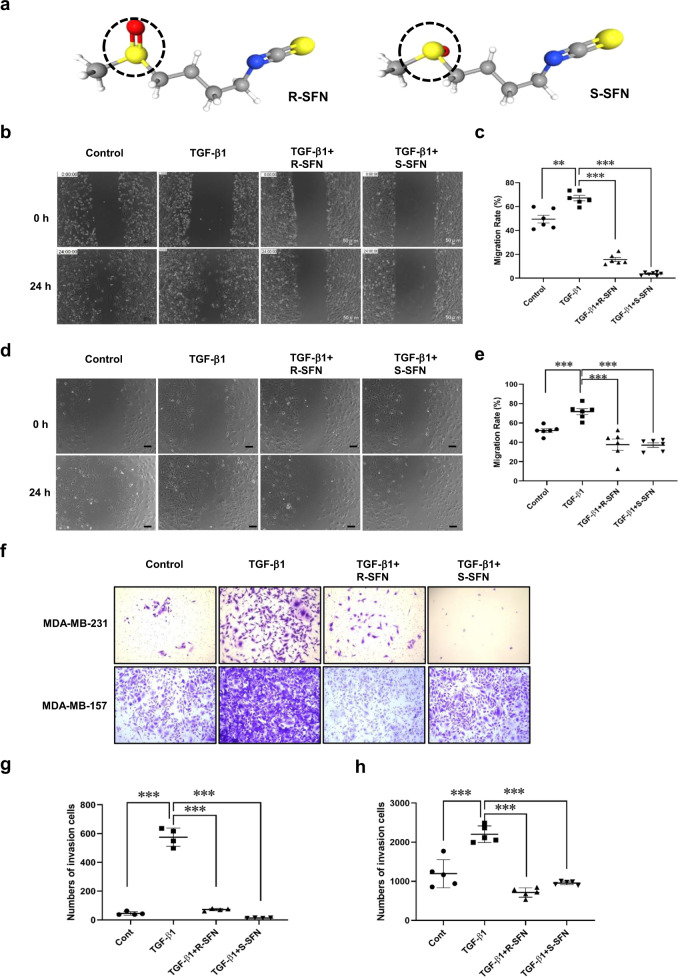
SFN isomers, R-SFN, and S-SFN, equally inhibit TGF-β1-induced migration and invasion of MDA-MB-231 and MDA-MB-157 cells. **a** The 3D-conformer of R-SFN and S-SFN. The dotted circle shows the difference in structure between R-SFN (PubChem Identifier: CID 9577379. URL: https://pubchem.ncbi.nlm.nih.gov/compound/9577379#section=3D-Conformer) and S-SFN (PubChem Identifier: CID 10479733. URL: https://pubchem.ncbi.nlm.nih.gov/compound/S_-sulforaphane#section=3D-Conformer). **b**, **d** Representative images showing the wound width of MDA-MB-231 (**b**) and MDA-MB-157 (**d**) cells in the presence of R-SFN and S-SFN at 24 h by wound healing assay. Scale bar = 100 μm. **c**, **e** Statistical assay showing that the effects of R-SFN and S-SFN on TGF-β1-induced migration of MDA-MB-231 (**c**) and MDA-MB-157 (**e**) cells at 24 h. Data shown are mean ± SEM. *n* = 5 or 6, ^**^P < 0.05; ^***^P < 0.01. **f** Representative images showing that invasion of MDA-MB-231 and MDA-MB-157 cells in the presence of R-SFN and S-SFN at 24 h by transwell assay. **g**, **h** Statistical assay showing that the effects of R-SFN and S-SFN on TGF-β1-induced invasion of MDA-MB-231 (**g**) and MDA-MB-157 (**h**) cells at 24 h. Data shown are mean ± SEM. *n* = 4 or 5, ^**^P < 0.05; ^***^P < 0.001.

R-sulforaphane (R-SFN) and S-sulforaphane (S-SFN) are two isomers of SFN. Their chemical structures are shown in Fig. 3a. R-SFN is the naturally-occurring isomer, found in broccoli and other vegetables. S-SFN is a synthesized isomer. To investigate whether the conformational structure affects its function, we then compared the effects of two isomers of SFN, R-SFN, and S-SFN, on cell migration and invasion. As shown in Fig. 3b, c, R-SFN and S-SFN significantly inhibited TGF-β1-induced migration of MDA-MB-231 cells. In the transwell invasion assay, R-SFN and S-SFN also significantly inhibited TGF-β1-induced invasion of MDA-MB-231 cells (Fig. 3f, g). Similarly, R-SFN and S-SFN also inhibited TGF-β1-induced migration and invasion of MDA-MB-157 cells (Fig. 3d, e, f, h). These results showed that R-SFN and S-SFN have equally inhibitory effects on breast cancer cell migration and invasion.

### The inhibition of SFN on migration and invasion is highly correlated with the regulation of actin cytoskeleton

**Fig. 4:**
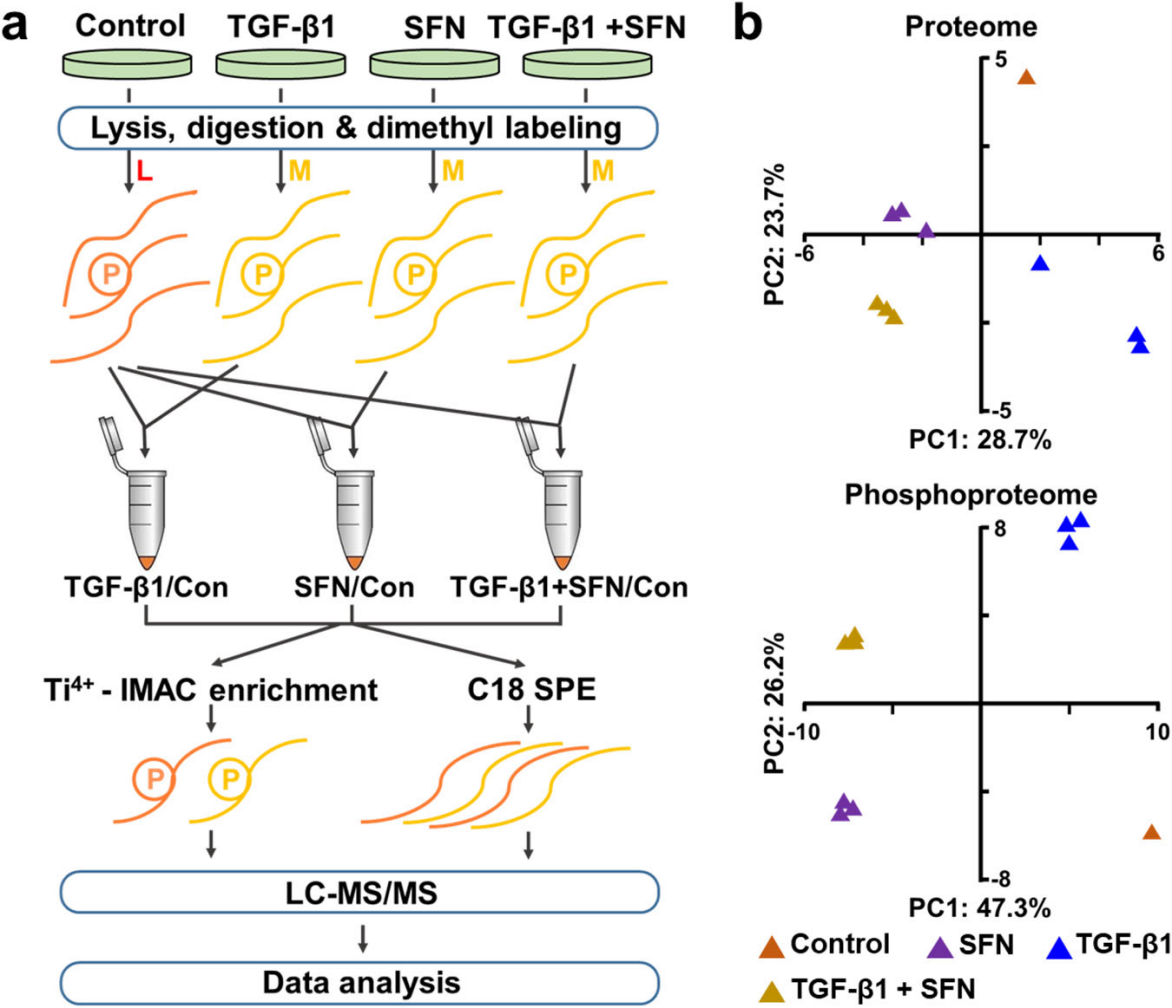
Mass spectrometry-based quantitative proteomics and phosphoproteomic profiling in MDA-MB-231 cells treated with TGF-β1 in the absence or presence of SFN. **a** The workflow of the mass spectrometry-based phosphoproteomic analysis. **b** Principal-component analysis of all identified normal proteins and phosphorylation sites shows that samples cluster tightly according to biology. The log2 ratios of normal proteins and phosphorylation sites in the control group was defaulted to 0.

To investigate the potential mechanism by which SFN inhibited TGF-β1-induced migration and invasion of MDA-MB-231 cells, we performed quantitative proteomic and phosphoproteomic analysis. In total, 1669 normal proteins (Supplementary Table S1, <1% false discovery rate [FDR]) and 3198 phosphorylation sites in 1321 phosphoproteins were quantified (Supplementary Table S2, <1% FDR). The principal-component analysis (PCA) of normal proteins and phosphorylation sites verified the reproducibility of these results (Fig. 4b).

**Fig. 5:**
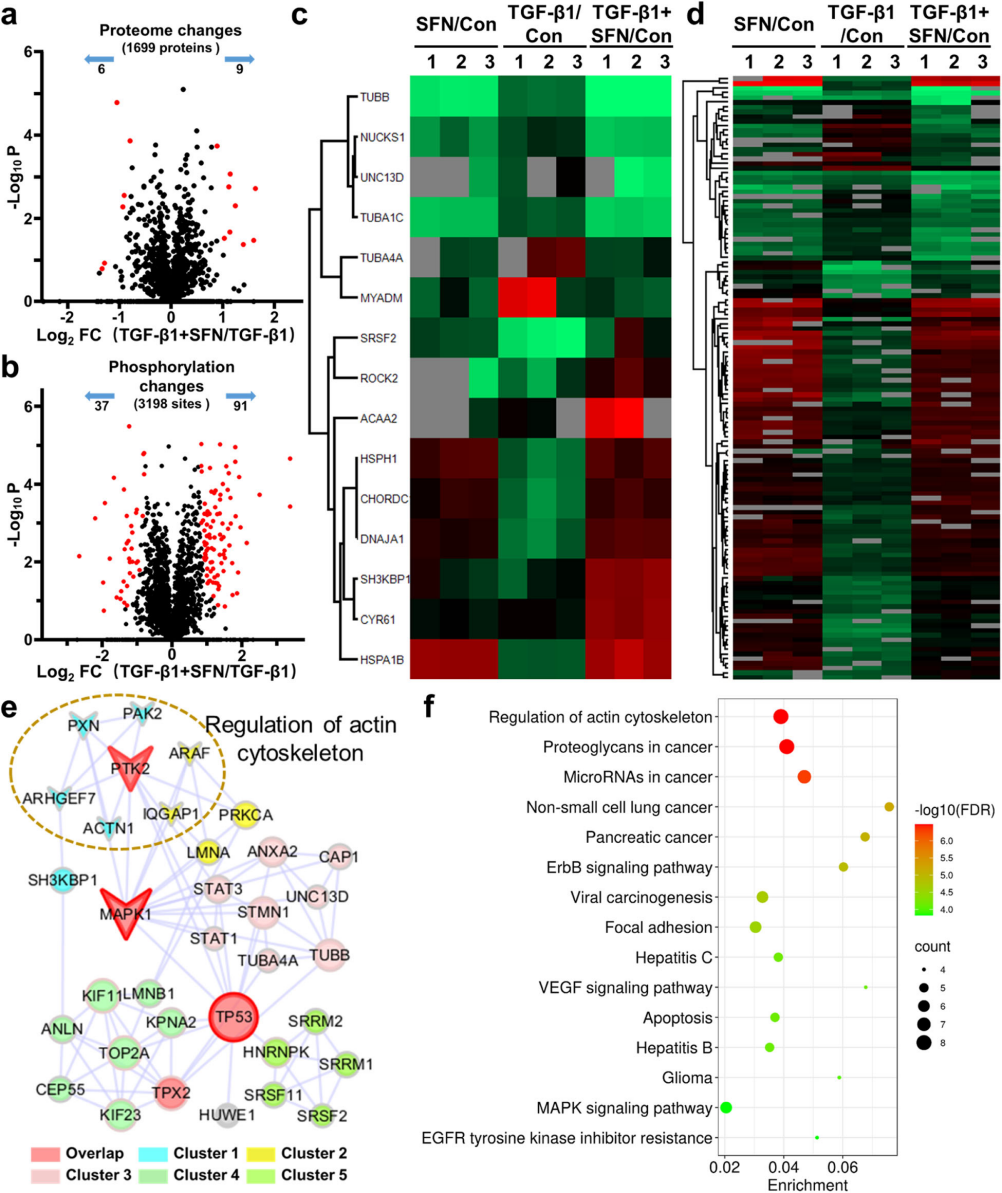
Proteosomic and phosphoproteomic analysis in MDA-MB-231 cells treated with TGF-β1 in the absence or presence of SFN. **a** A volcano plot shows that 15 proteins (red dots) changed significantly (FDR <0.05, S0 of 1) upon SFN treatment. **b** A volcano plot shows that 128 localized phosphorylation sites (red dots) changed significantly (FDR <0.05, S0 of 1) upon SFN treatment. **c** Cluster analysis of differentially expressed proteins. **d** Cluster analysis of **d**ifferentially expressed phosphorylation sites. **e** The core network of protein–protein interaction map of the altered proteins and phosphoproteins that are illustrated with color by clusters and size by degree. MAPK1, TP53, and PTK2 are in the top three degrees and served with a red border. The nodes involved in the regulation of the actin cytoskeleton are displayed by arrows. **f** KEGG pathways enrichment of core altered proteins and phosphoproteins.

Compared with the TGF-β1 treatment group, SFN treatment significantly changed the expression of 15 proteins (six downregulated and nine upregulated; Fig. 5a, c) and the phosphorylation level of 128 phosphorylation sites (37 downregulated and 91 upregulated; Fig. 5b, d) in 100 proteins. Next, to explore the possible cellular functions and signal pathways activated after SFN treatment, we used the String database (https://string-db.org/) to establish protein–protein interactions (PPI) of differentially expressed proteins and phosphoproteins and extracted the core network by combining the interaction score >0.7 and degree >5 (Fig. 5e). PPI analysis and the KEGG pathway enrichment of the extracted core network showed that the inhibitory effect of SFN on migration and invasion was highly correlated with the regulation of actin cytoskeleton (Fig. 5f).

### SFN inhibits actin stress fiber formation and the expression of actin stress fiber formation-associated proteins

**Fig. 6:**
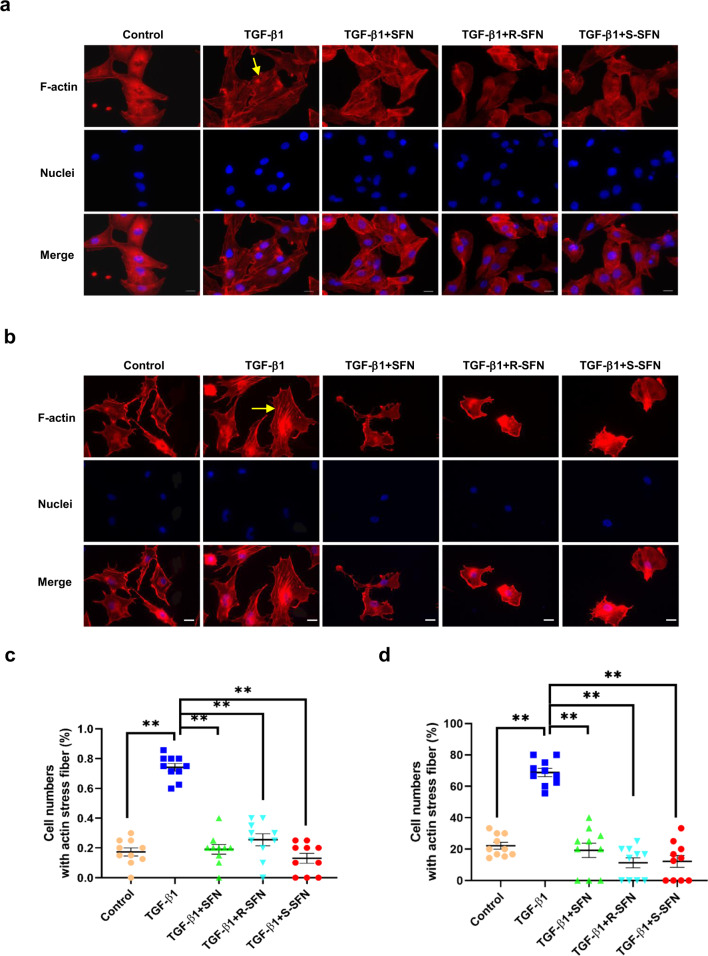
SFN inhibits TGF-β1-induced actin stress fiber formation. **a**, **b** Representative images showing the effect of SFN, R-SFN, and S-SFN on TGF-β1-induced actin stress fiber formation in MDA-MB-231 (**a**) and MDA-MB-157 (**b**) cells. Yellow arrows label the cells with remarkable actin stress fiber formation. Scale bar = 20 μm. **c**, **d** Statistical assay showing the effect of SFN on TGF-β1-induced actin stress fiber formation in MDA-MB-231 (**c**) and MDA-MB-157 (**d**) cells. Ten fields of view were randomly selected and then the ratio of the cells with actin stress fiber formation in each group was calculated. At least 60 cells in three independent experiments were counted. Data shown are mean ± SEM.^**^P < 0.05.

Proteomics analysis results indicated that the actin cytoskeleton played an important role in SFN inhibiting cell migration and invasion. The driving force for cell migration and invasion is provided by actin cytoskeleton polymerization, which induces membrane protrusion and pseudopod formation^42,43^. Therefore, we next evaluated the effect of SFN on actin stress fiber formation in breast cancer cells. The results showed that TGF-β1 induced actin stress fiber formation in MDA-MB-231 cells. However, when the cells were treated with SFN, TGF-β1-induced actin stress fiber formation was almost completely abolished (Fig. 6a, c). Similar phenomena were also observed in MDA-MB-157 cells (Fig. 6b, d).

**Fig. 7:**
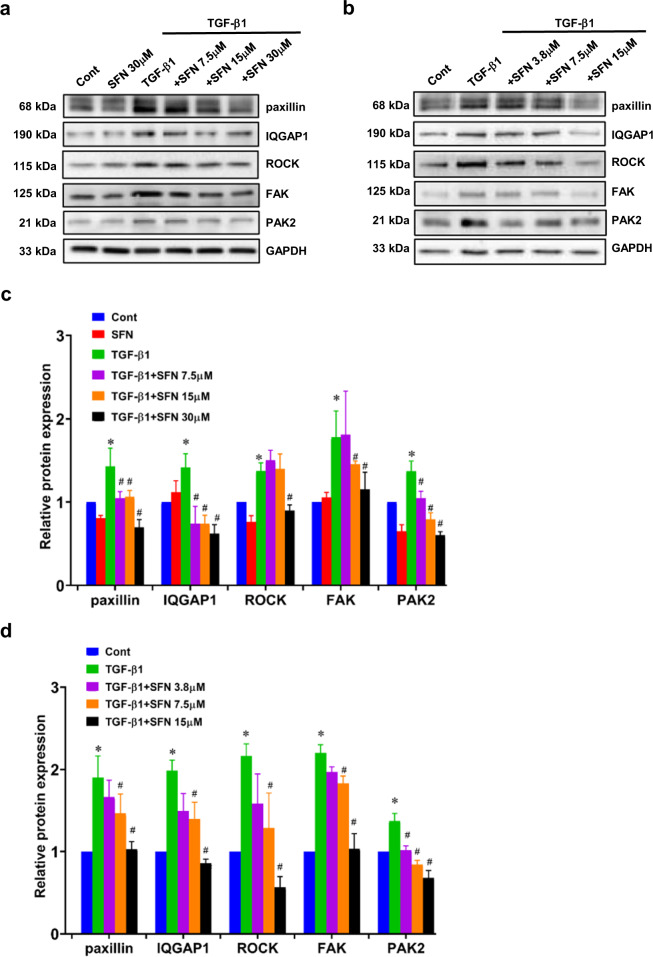
SFN inhibits TGF-β1-induced actin stress fiber formation-associated protein expression. **a**, **b** Representative western blot results showing that the effects of SFN at different concentrations (7.5, 15, and 30 μM for MDA-MB-231 cells and 3.8, 7.5, and 15 μM for MDA-MB-157 cells) on TGF-β1-induced actin stress fiber formation-associated protein expression, including paxillin, focal adhesion kinase (FAK), Ras GTPase-activating-like protein (IQGAP1), Rho-kinase (ROCK), and p21-activated kinase 2 (PAK2) in MDA-MB-231 (**a**) and MDA-MB-157 (**b**) cells. **c**, **d** Statistical assay showing that the effects of SFN at different concentrations on TGF-β1-induced actin stress fiber formation-associated proteins expression in MDA-MB-231 (**c**) and MDA-MB-157 (**d**) cells. Data shown are mean ± SEM. *n* = 3, ^∗^*P* < 0.05 vs Cont; ^#^*P* < 0.01 vs TGF-β1.

Then the expression of actin stress fiber formation-associated proteins was examined. TGF-β1 treatment increased the expression of actin stress fiber formation-associated proteins, including paxillin, IQGAP1, FAK, PAK2, and ROCK. However, SFN treatment decreased the expression of those proteins, indicating that SFN inhibited TGF-β1-induced expression of paxillin, IQGAP1, FAK, PAK2, and ROCK. With the increase of SFN concentration, a more obvious inhibitory effect was observed, suggesting that SFN concentration-dependently inhibits actin stress fiber formation-associated proteins expression (Fig. 7a–d).

### SFN inhibits the RAF/MEK/ERK signaling pathway by direct binding to RAF kinases

**Fig. 8:**
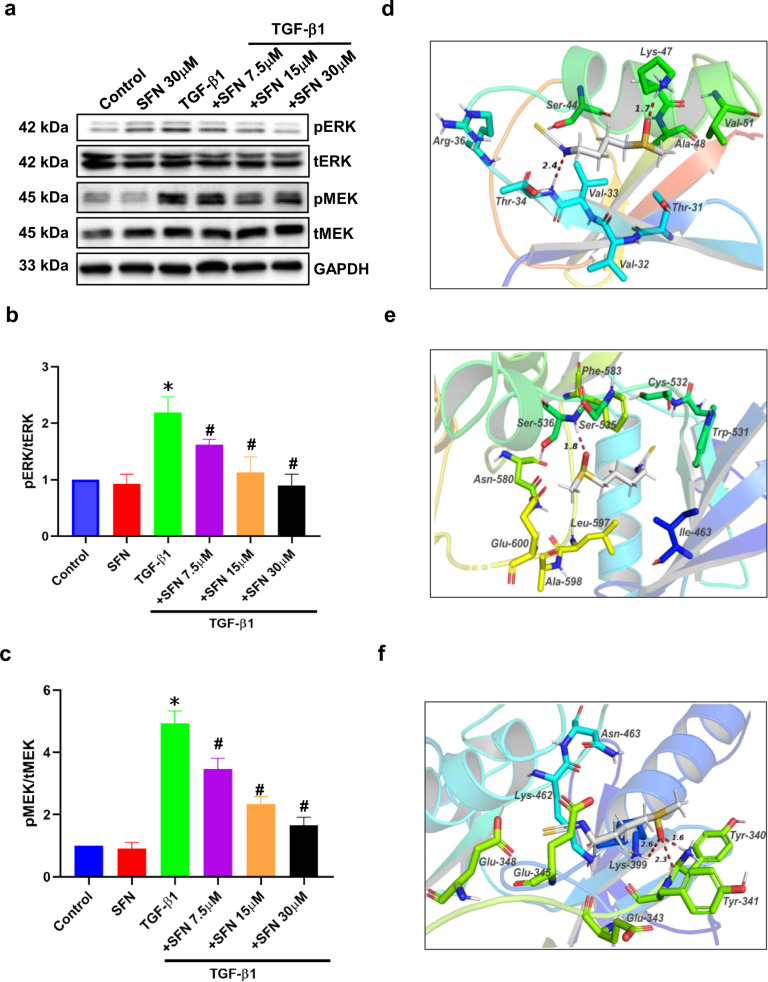
SFN inhibits TGF-β1-induced RAF/MEK/ERK pathway by direct binding to RAF family kinases. **a** Representative western blot results showing the effects of SFN at different concentrations (7.5, 15, and 30 μM) on TGF-β1-induced MEK and ERK phosphorylation. **b**, **c** Statistical assay showing the effects of SFN at different concentrations (7.5, 15, and 30 μM) on MEK and ERK phosphorylation, respectively. Data shown are mean ± SEM. *n* = 3, ^∗^*P* < 0.05 vs Control; ^#^*P* < 0.05 vs TGF-β1. **d**–**f** Molecular docking showing that SFN directly binds to RAF family kinases, including ARAF, BRAF, and CRAF through H-bond. The dotted lines and the corresponding values present the H-bonds and their distances. pERK phosphorylated ERK, tERK total ERK, pMEK phosphorylated MEK, tMEK total MEK.

The core network of the PPI map of the altered proteins and phosphoproteins (Fig. 5e) showed ARAF may also be involved in actin cytoskeleton regulation. ARAF is one of the three members of the RAF serine/threonine kinase family, and the other two are BRAF and CRAF. RAF kinases activation can cause the phosphorylation of many proteins, including MEK and ERK phosphorylation. Previous reports demonstrated the RAF/MEK/ERK signaling pathway plays a pivotal role in cancer metastasis^44,45^. Therefore, we then examined whether SFN could regulate the MEK and ERK phosphorylation. As shown in Fig. 8a–c, SFN concentration-dependently inhibited TGF-β1-induced MEK and ERK phosphorylation, suggesting that SFN inhibited the function of RAF.

SFN is a small-molecule compound and is reported to be an inhibitor of histone deacetylase (HDAC)^46,47^. Recent studies have shown that several small-molecule HDAC inhibitors are also RAF kinase inhibitors^48,49^. Additionally, inhibition of HDAC6 activity, as well as the EGFR-RAS-RAF-MEK-ERK signaling pathway, may cooperatively reduce cell migration^50^. Therefore, we hypothesized that SFN may directly act on RAF family proteins to regulate the RAF-MEK-ERK signaling pathway. Then, we examined the molecular docking of SFN and RAF family proteins, including ARAF, BRAF, and CRAF. Our results showed that SFN could interact with ARAF, BRAF, and CRAF through hydrogen bonds. As shown in Fig. 8d, SFN can form two hydrogen bonds with the Thr34 and Lys36 of ARAF. SFN forms a hydrogen bond with BRAF at the position of Ser536 (Fig. 8e). SFN can form three hydrogen bonds with CRAF at the position of Lys399, Tyr340, and Tyr341 (Fig. 8f). These results suggest that SFN targets RAF serine/threonine kinases to inhibit the RAF/MEK/ERK signaling pathway, thereby inhibiting the migration and invasion of breast cancer cells.

## Discussion

In this study, we demonstrated that SFN effectively suppressed the metastasis of TNBC cells, including cell migration and invasion. The results of mass spectrometry-based quantitative phosphoproteomic profiling, immunofluorescence, and western blot showed that the inhibitory effect of SFN on breast cancer cell migration and invasion is attributed to the reduction of actin stress fiber formation and its associated protein expression, including paxillin, FAK, IQGAP1, ROCK, and PAK2. Further, we demonstrated that SFN bound to the RAF family proteins, including ARAF, BRAF, and CRAF, subsequently inhibited phosphorylation of MEK and ERK. These in vitro results indicate that SFN suppresses metastasis of breast cancer cells by targeting the RAF/MEK/ERK pathway to inhibit actin stress fiber formation. Our results provide a novel insight into the role of SFN in breast cancer metastasis through the RAF/MEK/ERK signaling pathway.

Many studies have shown that SFN has anticancer properties by affecting different biological functions of tumor cells. SFN acts as an HDAC inhibitor in breast cancer cell lines and decreases the protein expression of ER, EGFR, and HER-2^51^. SFN also induces cell cycle arrest and apoptosis in HT29 human colon cancer cells^52^. In addition, SFX-01, a stabilized formulation of SFN, has been reported to inhibit endocrine-resistant stem-like cells in ER-positive breast cancer^53^. A recent clinical study showed that RAF, MEK, pMEK, ERK, and pERK mRNA and protein expressions are higher in the axillary lymph node metastasis (ALNM) group compared to the non-ALNM group and normal group, suggesting that the RAF/MEK/ERK signaling pathway may be a higher correlation with breast cancer metastasis^44^. Here, we demonstrated that SFN inhibits the metastasis of TNBC cells through targeting the RAF/MEK/ERK signaling pathway to inhibit the formation of actin stress fiber.

The RAF/MEK/ERK signaling pathway plays a crucial role in diverse cellular processes, including cell proliferation, migration, and survival. RAS-RAF interaction is the first step during the process of RAF activation^54^. The activated RAF phosphorylates and activates MEK kinases, which in turn phosphorylate and activate ERK. Although the activation process of RAF is not completely understood, the interaction between RAS and RAF and RAF phosphorylation are essential for the activation of RAF. The structure of RAF kinases is divided into two functional regions as the regulatory domain and the kinase domain^55^. Although all RAF kinases have a high degree of sequence similarity, they are under different regulations and have their own functions. This may be the reason why our molecular docking results indicate that SFN binds to all molecules but the binding sites are different. Thr34 of the ARAF kinase is in a RAS binding domain^56,57^. Molecular docking results showed that the N atom of SFN can form a hydrogen bond with Thr34 of the ARAF kinase, suggesting that SFN may inhibit the activation of ARAF by blocking the binding of RAS and ARAF, thereby inhibiting the RAF/MEK/ERK signaling pathway. For BRAF, as shown in Fig. 8e, SFN forms a hydrogen bond with Ser536 of the BRAF kinase. Ser536 of BRAF is in the kinase domain, which is the MEK binding site^56,58^. The combination of SFN and BRAF may inhibit RAF to phosphorylate MEK. As shown in Fig. 8a, c, SFN treatment inhibited MEK phosphorylation. However, unlike ARAF and BRAF, CRAF provides three sites of Lys399, Tyr340, and Tyr341 in the kinase domain^58^ interacting with SFN through three hydrogen bonds. The phosphorylation of RAF plays an important role in the kinase activation and provides a conformation to interact with regulatory molecules such as MEK and 14-3-3 protein^58^. The phosphorylation of Ser338, Tyr340, and Tyr341 positively regulates the kinase activity of CRAF^59^. Our recent data showed that SFN inhibits TGF-β1-induced phosphorylation of Ser338 in CRAF (Unpublished data), indicating that SFN can affect the activity of CRAF kinase. In summary, SFN interacts with RAF kinases to inhibit RAF/MEK/ERK signaling pathway. The detailed function of the interaction between SFN and RAF kinases needs further study in the future.

In addition, we also examined the effects of the two isomers of SFN, R-SFN, and S-SFN, on breast cancer metastasis. Research suggests that the R-isomer may be more bioactive than the S-isomer. However, in our current study, R-SFN and S-SFN exhibited equivalent anti-metastasis activity. A principal mechanism of the chemopreventive activity of SFN is modulation of the carcinogen-metabolizing enzyme systems. R-SFN and S-SFN have different effects on the elevation of hepatic glutathione *S*-transferase and quinone reductase in different tissues. A study by AFA Razis et al. showed that R-SFN is a more effective inducer of the carcinogen-detoxification enzyme system than S-SFN in rat liver and lung^60^. So far, few studies have compared the effects of R-SFN and S-SFN, and the equivalent inhibitory mechanism of R-SFN and S-SFN on breast cancer metastasis is unclear. Based on previous reports, we speculate that R-SFN and S-SFN may have the same effect on the carcinogen-metabolizing enzyme system in breast cancer cells. As a result, they inhibited the migration and invasion of breast cancer cells to the same extent. P Kiełbasiński et al. synthesized some new enantiomeric fluorine-containing derivatives of SFN and examined the relationship between absolute configuration and biological activity, suggesting that absolute configuration of the fluorine-containing derivatives of SFN is not a key factor determining their activity^61^. In addition, both isomers are ligands of RAF family kinases and regulate migration and invasion of breast cancer cells through RAF/MEK/ERK signaling pathway. R-SFN and S-SFN showed similar binding affinity (Supplementary Table S3), suggesting that both isomers may exhibit equivalent anti-metastasis activity.

SFN, a natural compound derived from cruciferous vegetables, has attracted more and more attention in the adjuvant treatment and chemoprevention of breast cancer due to its lower toxicity and fewer side effects. In MDA-MB-231 cell lines, the combined treatment of SFN and Withaferin A is more effective than either compound alone^62^. The combination of SFN and Gemcitabine, or 5-fluorouracil, or doxorubicin showed a synergistic inhibitory effect in breast cancer cell growth^63–65^. These studies indicate that SFN is a promising anticancer regent in adjuvant therapy in breast cancer patients. Although not many clinical studies have investigated the effect of SFN on breast cancer, in vitro and the in vivo studies have been conducted to show that SFN has a potential clinical scenario as a promising antineoplastic and chemopreventive compound. Atwell *et al*. provided evidence for chemopreventive activity of SFN in human breast tissue from a clinical trial in 54 women. SFN decreased HDAC activity and Ki-67 level, which are malignancy and proliferation markers^66^. Another study on the preclinical and clinical evaluation of SFN for breast chemoprevention showed that SFN is distributed to breast epithelial cells in vivo and exerts pharmacological effects in these target cells, further supporting the role of SFN for breast chemoprevention^67^. In the future, clinical trials are needed to clarify the efficacy of SFN and its mechanism in the adjuvant therapy of breast cancer.

**Fig. 9:**
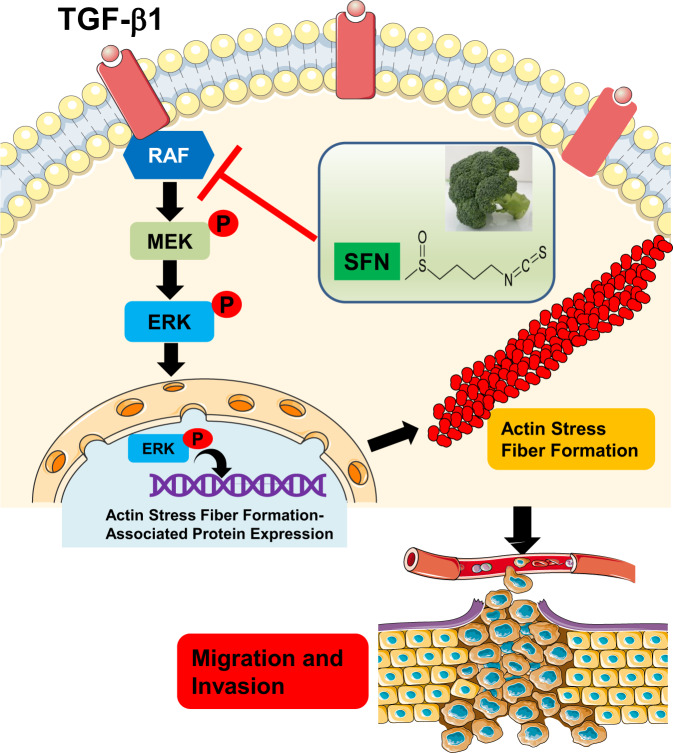
A schematic diagram showing SFN suppresses migration and invasion of breast cancer by targeting the TGF-β1-induced RAF/MEK/ERK pathway to reduce actin stress fiber formation. The illustrations such as receptors, plasma membrane, and cell migration and invasion were obtained from Smart Servier Medical Art (https://smart.servier.com/).

In this study, we provided in vitro evidence that SFN inhibits breast cancer cell metastasis by targeting the RAF/MEK/ERK pathway to attenuate actin stress fiber formation (Fig. 9). These results provide some new ideas and insights for exploring more effective treatment strategies for breast cancer metastasis, especially TNBC breast cancer. In addition, our data indicate that R-SFN and S-SFN have equal effects on migration and invasion of breast cancer cells. The current findings raise the possibility that the chemopreventive potential of the S-enantiomer may be underestimated. However, our study is limited to the results of in vitro studies, and the results of in vivo studies are needed to carefully evaluate whether SFN influences the metastasis of breast cancer patients.

## Methods

### Chemicals and reagents

SFN (a synthetic compound containing R- and S- isomers), R-SFN, and S-SFN were purchased from Abcam Laboratories (St. Paul, MN, USA) and resuspended in dimethyl sulfoxide (EMD Millipore, Darmstadt, Germany) to make a 100 mM stock stored at −20 °C. TGF-β1 was purchased from Fujifilm Wako Pure Chemical Corporation (Osaka, Japan) and resuspended in phosphate-buffered saline (PBS) containing 2 mg/mL bovine serum albumin to make a 40 μg/mL stock stored at ­20 °C.

The following antibodies were used: anti-paxillin antibody (BD Biosciences, 711725, CA, USA), anti-GAPDH monoclonal antibody (Fujifilm, 014–25524, Osaka, Japan), anti-Rho-kinase II antibody (BD Biosciences, 610624, CA, USA), anti-IQGAP1 antibody (H-109, Santa Cruz, sc-10792, Dallas, USA), anti-FAK antibody (Sigma-Aldrich, 06–543, St. Louis, MO), anti-PAK2 antibody (Cosmo Bio Co., LTD, CSB-PA010537, Tokyo, Japan), anti-phospho**-**MEK1/2 (Ser217/221) (41G9) rabbit antibody (Cell Signaling, 9154 S, MA, USA), anti-MEK antibody (Cell Signaling, 9122 S, MA, USA), anti-phospho-p44/42 Erk1/2 (Thr202/Tyr204) antibody (Cell Signaling, 9101 S, MA, USA), anti-ERK antibody (Sigma-Aldrich, 06–182, St. Louis, MO). Secondary HRP-labeled antibodies (anti-mouse, W4021 and anti-rabbit, W4011) were purchased from Promega. Alexa Fluor-conjugated secondary antibodies for immunofluorescence imaging (Molecular Probes, Eugene, OR) were used in accordance with the manufacturer’s protocol.

### Cell culture

Two human TNBC cell lines were used in this study. MDA-MB-231 (Ds Pharma Biomedical, Osaka, Japan) was cultured in L-15 medium containing glutamine supplemented with 15% FBS and 0.5% NaHCO_3_ in the presence of 50 U/ml penicillin and 50 mg/ml streptomycin at 37 °C with 95% air and 5% CO_2_. MDA-MB-157 (ATCC, Rockville, MD) was cultured in L-15 medium containing glutamine supplemented with 10% FBS in the presence of 50 U/ml penicillin and 50 mg/ml streptomycin at 37 °C without CO_2_. The cells were digested using trypsin/EDTA and planted into the new flask at a ratio between 1:3 and 1:6 to passage. We stocked the cells using Cellbanker 1 (Kurabo, Osaka, Japan) at −80 °C or liquid nitrogen. All cells were used in experiments during the linear phase of growth.

### Cell viability assay

A cell counting kit-8 (CCK-8) (Dojindo Laboratories, Kumamoto, Japan) was used to measure the cytotoxicity of SFN on MDA-MB-231 cells and MDA-MB-157 cells. Cells were seeded at a density of 1 × 10^4^ cells per well in 100 μL of L-15 medium into a 96-well plate, and cultured overnight. Cells were treated with different concentrations of SFN (0–240 μM) and incubated for 24 h in five parallel wells. At the high concentration of 240 μM, we treated cells with an equal amount of DMSO as control. Next, added 10 µL of CCK-8 solution to each well and incubated for another 2 h. The absorbance was measured at a wavelength of 450 nm using a microplate reader (Bio-Rad Laboratories, Richmond, CA, USA). Cell viability was expressed as a percentage of that of the control (untreated) cells.

### Wound healing assay

The cells were grown in 35-mm glass-based dishes (Iwaki, Japan). When cell confluence reached 90–100%, FBS-free L-15 medium (GE Healthcare Bioscience, Piscataway, NJ) supplemented with 0.5% NaHCO_3_ for MDA-MB-231 cells or without 0.5% NaHCO_3_ for MDA-MB-157 cells in the presence of 50 U/ml penicillin and 50 mg/ml streptomycin was changed. After starving treatment for 24 h, cells were wounded using 200 μL micropipette tips and then TGF-β1 (20 ng/mL) and SFN were added. Time-lapse recording of cell migration was performed under a fluorescence microscope with a phase-contrast model (KEYENCE, Osaka, Japan) with a chamber at 37 °C and 5% CO_2_. Time-lapse images were captured at 30 min intervals for 24 h under phase contrast with 10× magnification of microscope objective. The data were used for quantitative analyses of cell migration using NIH Image J software. We calculated the migration rate as follows: The migration rate (%) = (scratch width at 0 h − scratch width at 24 h)/scratch width at 0 h× 100%.

### Transwell invasion assay

The invasion of MDA-MB-231 cells or MDA-MB-157 cells was determined using a corning invasion chamber 24-well plate (354480, Corning BioCoat, USA). The membrane was coated with extracellular matrigel matrix (BD Biosciences, CA, USA) at 37 °C for 30 min. Then serum-free cell suspension (1 × 10^6^/well) was placed into the upper chamber. The lower compartment was filled with L-15 medium adding with TGF-β1 (20 ng/mL) in the absence or presence of SFN, R-SFN, and S-SFN. After being cultured for 24 h, uninvaded cells were removed. The invading cells on the underside of the membrane were fixed with formaldehyde (4%) and stained with crystal violet (0.1%). Cells were visualized under a bright-field microscope (KEYENCE, Osaka, Japan).

### Digestion, stable isotope dimethyl labeling, and phosphopeptide enrichment

The protein extracts were precipitated with five volumes of ice-cold acetone/ethanol/acetic acid (v/v/v = 50/50/0.1) at −20 °C overnight. The precipitated protein sample was centrifuged at 15,000 g for 30 min and washed with acetone and 75% ethanol. The protein sample was re-dissolved into 8 M urea/50 mM HEPES (pH 8.0) solution and the protein concentration was determined by BCA assay. Then, the protein was reduced by 10 mM dithiothreitol (DTT) at 60 °C for 1 h and alkylated by 20 mM Iodoacetamide (IAA) in the darkness at room temperature for 30 min. Then the protein concentration was diluted to 1 mg/mL with 100 mM HEPES buffer, and digested by trypsin (enzyme/substrate, 1/25 (w/w)) overnight at 37 °C. Finally, the tryptic digests were isotopically labeled with dual mass-differentiate dimethyl groups as shown in Fig. 4a. The phosphopeptides were enriched by Ti^4+^-IMAC microspheres.

### LC-MS/MS analysis and database searching

All samples were analyzed by Orbitrap Fusion™ Tribrid™ mass spectrometer coupled with an UltiMateTM 3000 RSLCnano System (Thermo Fisher, San Jose, CA). The samples were loaded on a C18 capillary trap column (200 μm i.d. × 4 cm) packed with C18 AQ beads (5 μm, 120 Å). 15 cm × 75 µm i.d. analytical column packed with C18 AQ beads (3 μm, 120 Å) was used for LC separation. The buffers used for online separation were 0.1% (v/v) formic acid in water and 0.1% (v/v) formic acid in acetonitrile (ACN), the flow rate was 200 nL/min for the nanoflow LC-MS/MS analysis. The gradient from 5 to 35% (v/v) ACN was performed in 90 min. The MS and MS/MS spectra were collected by high collision-induced dissociation (HCD) at 35% energy in a data-dependent mode that 1 MS scan from 400 to 2000 m/z with the mass resolution of 60,000 at m/z 400 in the Orbitrap analyzer followed by 40 MS/MS scans in the ion trap, respectively. The dynamic exclusion repeat count was 1 with a duration time of 30 s, and the charge state rejection function was enabled to reject the ions with single and “unassigned” charge states.

The obtained raw files were searched using MaxQuant (Version 1.5.5.1) with a Uniprot protein FASTA database of humans. Trypsin was selected as the specific enzyme with up to 2 miss cleavages. The oxidation of methionine (M) was set as variable modifications. Meanwhile, the carbamidomethylation of cysteine (C) was set as fixed modification. The false discovery rate (FDR) for both peptide and protein identification were set below 1%. The results were then imported into the MaxQuant associated software suite Perseus for further analysis.

### Network analysis and KEGG enrichment of differentially expressed proteins

Digestion, stable isotope dimethyl labeling, phosphopeptide enrichment, and LC-MS/MS analysis are shown in the supplementary materials. Protein–protein interactions (PPI) of the differentially expressed proteins and phosphoproteins were predicted by STRING (https://string-db.org/) database with a filter of combined score >0.7 which was considered as high confidence^68,69^, and then visualized using Cytoscape v3.8.2. Topology parameters of the PPI network were calculated by NetworkAnalyzer. The core network was extracted according to the degree value (greater than the median degree value for all nodes in the network)^70^, followed by the cluster analysis with ClusterONE and KEGG pathways enrichment with String database^69^.

### Actin stress fiber staining

Actin stress fiber staining were performed as described previously^71^. Briefly, breast cancer cells were cultured on 0.3% gelatin-coated glass coverslips. After treatment with TGF-β1 (20 ng/mL) for 24 h with or without SFN, cells were fixed in 2% paraformaldehyde in PBS for 10 min, permeabilized with 0.1% Triton X-100 in PBS for 2 min, and blocked in NanoBio blocker solution (Nano Bio-Tech Co., Ltd) diluted in PBS for 60 min at 25 °C. Cells were then incubated with rhodamine-phalloidin (1:100; Molecular Probes, Eugene, OR) and DAPI (Molecular Probes, Eugene, OR) for 60 min at 25 °C. Coverslips were washed with PBS, rinsed with deionized water, and mounted with PermaFluor aqueous mounting medium (Thermo Fisher Scientific, Waltham, MA). Specimens were observed using fluorescence microscopy (KEYENCE, Osaka, Japan) at 60× magnification of microscope objective. F-actin with a thick linear structure was observed in the whole cells, which means the actin stress fiber formation. Actin stress fiber formation was quantified by counting the number of the cells that contain a thick linear structure of actin stress fibers in the cell cortex or not within cells in at least ten random independent fields (n > 60) under a fluorescence microscope^12,72^.

### Western blotting analysis

MDA-MB-231 or MDA-MB-157 cells were serum-starved for 24 h, then stimulated with TGF-β1 (20 ng/mL) and in the presence of SFN or vehicle control for 24 h. Cell lysates were prepared in RIPA cell lysis buffer (Wako, Osaka, Japan) supplemented with protease inhibitor cocktail (Sigma-Aldrich, St. Louis, MO), 0.2 mM Na_3_VO_4_, 10 mM NaF, 10 μM leupeptin, and 1 mg/ml aprotinin. The lysates were centrifuged, and the supernatants were boiled in sodium dodecyl sulfate (SDS) loading buffer. Proteins in the total cell lysate (10 μg) were separated using 10% SDS-polyacrylamide gel electrophoresis, transferred to Amersham Hybond-P PVDF membrane (GE Healthcare Life Sciences), and blocked in 5% nonfat milk in 0.05% Tween-20 phosphate-buffered saline (TBS-T) for 60 min at room temperature. The membranes were incubated with primary antibodies overnight at 4 °C, followed by incubation with HRP-conjugated secondary antibodies (anti-mouse or anti-rabbit antibodies, 1:5,000) for 1 h at room temperature. Immunoreactive bands were detected using a Supersignal West Pico Chemiluminescent Substrate kit (Thermo Fisher Scientific, Waltham, MA). GAPDH was used as a loading control. All blots were processed in parallel and derived from the same experiment (Uncropped blots are shown in Supplementary Fig. S1).

The following concentrations of primary antibodies were used: anti-paxillin antibody, 1:5,000; anti-IQGAP1 antibody, 1:1,000; anti-ROCK antibody, 1:500; anti-FAK antibody, 1:1,000; anti-PAK2 antibody, 1:1,000; anti-GAPDH antibody, 1:10,000; anti-phospho-ERK, 1:1,000; anti-ERK antibody, 1:1,000; anti-phospho-MEK antibody, 1:1,000; anti-MEK antibody, 1:1,000.

### Molecular docking

The binding ability, sites, and interactions between SFN and RAF serine/threonine kinases were achieved and analyzed by classical molecular dynamics using DockThor database^73^ and visualized using Pymol 2.4. The 3D chemical structural formula of SFN was obtained from PubChem (http://pubchem.ncbi.nlm.nih.gov) and the crystal structures of putative RAF serine/threonine kinases were obtained from the Protein Data Bank (http: //www.rcsb.org). PDB code of ARAF: 1wxm; PDB code of BRAF: 6p3d; PDB code of CRAF: 3omv.

### ProteomeXchange accession

The mass spectrometry proteomics data have been deposited to the ProteomeXchange Consortium via the PRIDE^74^ partner repository with the dataset identifier PXD025639.

### Statistical analysis

Statistical analysis was performed using GraphPad Prism 8.0.2. All experiments were performed on at least three independent occasions. Data shown represent mean ± SEM. The student’s *t*-test was used to evaluate the differences between the control group and the experimental group, and a one-way analysis of variance followed by Tukey’s test was used to evaluate the differences among multiple groups. *P* < 0.05 was considered statistically significant.

## Supplementary information

Supplementary material

Related Manuscript File

## Data Availability

The datasets generated during and/or analysed during the current study are available from the corresponding author on reasonable request.

## Code availability

Detailed data analysis can be found in the methods section. All relevant data are deposited into a public repository and accession codes are provided.
