# Supplementary material for: Sulforaphane suppresses metastasis of triple-negative breast cancer cells by targeting the RAF/MEK/ERK pathway
Source: NPJ Breast Cancer. 2022 Mar 24;8:40. doi: 10.1038/s41523-022-00402-4 (PMC8948359; doi:10.1038/s41523-022-00402-4)

Supplementary Table S1 The results of proteomic analysis

| Protein ID | Protein names | Gene names | Unique peptides | Sequence coverage [%] | Q-value | Score | Ratio of TGF-β 1+SFN | Ratio of TGF-β 1+SFN | Ratio of TGF-β 1+SFN | Ratio of SFN / Con_1 | Ratio of SFN / Con_2 | Ratio of SFN / Con_3 | Ratio of TGF-β1/ Con_1 | Ratio of TGF-β1/ Con_2 | Ratio of TGF-β1/ Con_3 |
|---|---|---|---|---|---|---|---|---|---|---|---|---|---|---|---|
| A0A2R8YD12 | Serpin B6 | SERPINB6 | 9 | 38.6 | 0 | 77.31 | 1.23 | 1.36 | 1.18 | 1.15 | 0.92 | 1.19 | 1.11 | 1.24 | 1.13 |
| A0A024R4E5 | Vigilin | HDLBP | 15 | 16.7 | 0 | 33.19 | 1.00 | 1.25 | NaN | NaN | 0.85 | 0.93 | NaN | 0.97 | 0.79 |
| A0A024R4M0 | 40S ribosomal protein S9 | RPS9 | 15 | 57.7 | 0 | 32.79 | 0.74 | 0.75 | 0.73 | 0.77 | 0.95 | 0.80 | 0.85 | 0.68 | 0.75 |
| A0A024RA52 | Proteasome subunit alpha type;Proteasome subunit alpha type-2 | PSMA2 | 10 | 53.4 | 0 | 39.83 | 0.95 | 1.12 | 1.03 | 1.06 | 0.88 | 1.04 | 1.05 | 0.93 | 0.89 |
| E9PKG6 | Nucleobindin-2;Nesfatin-1 | NUCB2 | 4 | 15.1 | 0 | 3.55 | 0.92 | 0.85 | 0.93 | 0.77 | 0.77 | 0.91 | 0.79 | 0.89 | NaN |
| A0A087WSW 9 | Thioredoxin reductase 1, cytoplasmic | TXNRD1 | 15 | 51.1 | 0 | 322.7 | 1.42 | 1.38 | 1.38 | 1.24 | 1.65 | 1.45 | 1.02 | 1.01 | 0.88 |
| A0A087WT44 | Heme oxygenase 2 | HMOX2 | 3 | 11.6 | 0 | 6.981 | NaN | NaN | NaN | 0.80 | NaN | 0.61 | NaN | 0.51 | 0.63 |
| A0A087WTP3 | Far upstream element-binding protein 2 | KHSRP | 22 | 40.2 | 0 | 323.3 | 1.16 | 1.19 | 1.11 | 1.00 | 1.17 | 1.27 | 1.14 | 0.84 | 1.07 |
| A0A087WU07 | MICOS complex subunit MIC10 | MINOS1 | 2 | 24.2 | 0.009 | 1.412 | 1.33 | 1.33 | 1.09 | NaN | NaN | 1.12 | NaN | NaN | 0.91 |
| A0A087WUB9 | Beta-catenin-like protein 1 | CTNNBL1 | 4 | 6.5 | 0 | 3.143 | 0.91 | 1.10 | 1.04 | NaN | 1.13 | 0.96 | 1.14 | 1.25 | 0.54 |
| E9PL01 | Signal peptidase complex subunit 2 | SPCS2 | 3 | 26.1 | 0 | 112.1 | 1.13 | 1.27 | 1.23 | 1.14 | 1.12 | NaN | NaN | 1.35 | 1.22 |
| A0A087WUD3 | Oligosaccharyltransferase complex subunit OSTC | OSTC | 1 | 14.5 | 0.002 | 1.956 | 1.05 | 1.06 | 0.94 | 0.91 | NaN | 0.93 | 0.98 | NaN | 0.99 |
| A0A087WUK2 | Heterogeneous nuclear ribonucleoprotein D-like | HNRNPDL | 7 | 23.7 | 0 | 185.9 | 0.79 | 0.83 | 0.82 | NaN | 0.77 | 0.81 | 0.94 | 0.95 | 0.78 |
| A0A087WZG9 | Retrotransposon-derived protein PEG10 | PEG10 | 2 | 6.7 | 1E-03 | 2.249 | 1.68 | 1.72 | 1.69 | 1.46 | 1.53 | 2.05 | NaN | 1.48 | 1.38 |
| A0A087WUN7 | SRA stem-loop-interacting RNA-binding protein, mitochondrial | SLIRP | 4 | 55.4 | 0 | 24.8 | 0.90 | 1.49 | 0.79 | 0.64 | 0.77 | 0.80 | 0.69 | 0.68 | NaN |
| A0A087WUQ 6 | Glutathione peroxidase;Glutathione peroxidase 1 | GPX1 | 3 | 22.8 | 0 | 5.291 | 1.17 | NaN | 0.96 | NaN | NaN | NaN | NaN | NaN | NaN |
| E7ETK0 | 40S ribosomal protein S24 | RPS24 | 4 | 26.7 | 0 | 49.47 | 0.74 | 0.78 | 0.90 | 0.94 | 0.82 | 0.74 | 0.74 | 0.74 | 0.67 |
| A0A087WUT6 | Eukaryotic translation initiation factor 5B | EIF5B | 18 | 21.7 | 0 | 38.88 | 1.31 | 0.93 | 1.03 | 1.16 | 0.77 | 0.91 | NaN | 0.98 | 0.99 |
| A0A087WV05 | C-Myc-binding protein | MYCBP | 2 | 28.2 | 0 | 7.662 | NaN | NaN | 0.75 | NaN | 1.17 | NaN | NaN | 0.99 | 1.18 |
| A0A087WVQ6 | Clathrin heavy chain;Clathrin heavy chain 1 | CLTC | 58 | 51.7 | 0 | 323.3 | 1.05 | 1.01 | 1.06 | 1.01 | 1.01 | 1.05 | 0.97 | 0.99 | 1.00 |
| A0A087WW4 | Endophilin-B1 | SH3GLB1 | 7 | 28.4 | 0 | 28.76 | 1.16 | NaN | 1.80 | 2.06 | 0.71 | 0.77 | 0.91 | 2.76 | 0.98 |
| A0A087WWJ1 | DNA mismatch repair protein | MSH6 | 4 | 4.6 | 0 | 3.205 | NaN | NaN | NaN | 0.73 | 0.67 | 0.59 | 0.76 | 0.77 | NaN |
| A0A087WWU | | TPM3 | 0 | 81.1 | 0 | 323.3 | 1.03 | 1.00 | 1.02 | 0.99 | 1.08 | 0.97 | 0.99 | 1.07 | 0.98 |
| Q8IXW7 | Fragile X mental retardation protein 1 | FMR1 | 1 | 7.4 | 1E-03 | 2.234 | 1.13 | 1.76 | 1.32 | NaN | NaN | NaN | NaN | NaN | NaN |
| A0A087WXM | 60S ribosomal protein L17 | RPL17 | 7 | 37.9 | 0 | 97.17 | 0.81 | 0.68 | 0.88 | 0.89 | 0.83 | 0.83 | 0.79 | 0.86 | 1.12 |

| | | | | | | | | | | | | | | | | |
|---|---|---|---|---|---|---|---|---|---|---|---|---|---|---|---|---|
| A0A087WXR5 | NADH dehydrogenase [ubiquinone] 1 alpha subcomplex subunit 5 | NDUFA5 | 1 | 33.8 | 0 | 54.98 | 0.97 | 1.01 | 1.05 | NaN | NaN | 0.86 | NaN | NaN | 1.18 |
| A0A087WY55 | Vacuolar protein sorting-associated protein VTA1 | VTA1 | 5 | 27.9 | 0 | 10.58 | NaN | 0.94 | 0.70 | 1.11 | NaN | 0.93 | 1.09 | NaN | 0.90 |
| A0A087WY71 | AP-2 complex subunit mu | AP2M1 | 6 | 15.2 | 0 | 27.73 | 0.76 | 0.86 | 0.83 | 0.96 | NaN | 0.91 | 1.23 | 1.12 | 1.16 |
| A0A087WYN9 | ATP-dependent RNA helicase DHX29 | DHX29 | 1 | 1.1 | 5E-04 | 2.859 | 0.63 | 0.58 | NaN | NaN | NaN | NaN | NaN | NaN | NaN |
| A0A087WYS1 | UTP--glucose-1-phosphate uridylyltransferase | UGP2 | 9 | 38 | 0 | 145 | 0.90 | 0.96 | 0.85 | 0.97 | 0.94 | 0.95 | 0.92 | 1.34 | 1.05 |
| A0A087WZF1 | Lipoma-preferred partner | LPP | 6 | 19.8 | 0 | 92.35 | 1.11 | NaN | 1.35 | 1.12 | 1.32 | 1.00 | 0.99 | 0.97 | 1.62 |
| A0A087X020 | Ribosome maturation protein SBDS | SBDS | 11 | 39.6 | 0 | 30.45 | 1.00 | 0.99 | 1.09 | 1.49 | 1.08 | 1.15 | 1.59 | 1.12 | 0.90 |
| J3KN36 | Nodal modulator 1;Nodal modulator 3;Nodal modulator 2 | NOMO3 | 5 | 5.6 | 0 | 4.633 | NaN | NaN | 0.54 | 0.50 | 0.36 | 0.83 | 0.71 | 0.79 | 0.55 |
| J3QT73 | COP9 signalosome complex subunit 7b | COPS7B | 1 | 5.2 | 0.006 | 1.706 | 1.00 | 1.43 | 0.60 | 1.43 | NaN | NaN | NaN | 1.14 | 0.13 |
| A0A087X1Z3 | Proteasome activator complex subunit 2 | PSME2 | 13 | 61 | 0 | 142 | 1.11 | 1.15 | 1.25 | 1.12 | 1.03 | 1.24 | 1.08 | 1.17 | 1.11 |
| A0A087X2D8 | C-Jun-amino-terminal kinase-interacting protein 4 | SPAG9 | 7 | 8.1 | 0 | 6.925 | NaN | 0.77 | 0.74 | NaN | NaN | NaN | NaN | NaN | NaN |
| F1T0B3 | ATP-dependent RNA helicase DDX1 | DDX1 | 11 | 23.1 | 0 | 42.57 | 0.89 | 0.72 | 0.89 | 0.87 | 0.52 | 0.63 | 1.22 | 0.82 | 0.75 |
| A0A087X2I1 | 26S protease regulatory subunit 10B | PSMC6 | 15 | 48.6 | 0 | 79.13 | 1.16 | 1.10 | 1.15 | 1.05 | 1.22 | 1.03 | 1.07 | 1.09 | 1.03 |
| A0A0A0MR02 | Voltage-dependent anion-selective channel protein 2 | VDAC2 | 12 | 51.1 | 0 | 323.3 | 1.08 | 1.22 | 1.02 | 1.00 | 1.06 | 1.03 | 1.05 | 1.03 | 1.24 |
| B9ZVX7 | Glutathione S-transferase Mu 4;Glutathione S-transferase Mu 1;Glutathione S-transferase Mu 5 | GSTM1 | 2 | 28.2 | 0 | 8.262 | 0.49 | 0.60 | 0.43 | NaN | 0.62 | 0.77 | NaN | 1.25 | 0.77 |
| A0A0A0MRA5 | Heterogeneous nuclear ribonucleoprotein U-like protein 1 | HNRNPUL | 14 | 22.3 | 0 | 33.05 | 0.82 | 0.94 | 0.96 | 1.04 | 0.91 | 0.79 | 0.98 | 1.08 | 0.96 |
| A0A0A0MRF9 | Phosphoinositide phospholipase C;1-phosphatidylinositol 4,5-bisphosphate phosphodiesterase gamma-2 | PLCG2 | 1 | 1 | 0.008 | 1.522 | 1.38 | 1.32 | 1.26 | NaN | 1.26 | 1.20 | NaN | 1.88 | 1.54 |
| A0A0A0MRI2 | Sorting nexin-6;Sorting nexin-6, N-terminally processed | SNX6 | 5 | 11.7 | 0 | 5.436 | NaN | 0.90 | 0.91 | 0.70 | 1.33 | NaN | NaN | 0.92 | 1.03 |
| F6S8N6 | Protein-L-isoaspartate O-methyltransferase;Protein-L-isoaspartate(D-aspartate) O-methyltransferase | PCMT1 | 8 | 54 | 0 | 98.42 | 1.02 | 1.09 | 1.13 | 1.22 | 1.13 | 0.99 | 1.61 | 1.78 | 1.02 |
| A0A0A0MRM | Unconventional myosin-VI | MYO6 | 15 | 18.8 | 0 | 43.09 | 2.03 | 0.97 | 1.89 | 1.42 | 1.43 | 1.03 | 0.63 | 0.95 | 0.76 |
| A0A0A0MRR7 | U1 small nuclear ribonucleoprotein C | SNRPC | 3 | 21.7 | 0 | 34.23 | 1.08 | 0.96 | 1.16 | 0.96 | 0.96 | 0.90 | 1.08 | 1.09 | 0.99 |
| J3KNW4 | Four and a half LIM domains protein 2 | FHL2 | 7 | 21.3 | 0 | 32.29 | 1.06 | 1.10 | 0.96 | 1.24 | 0.89 | 1.49 | 1.13 | 0.87 | NaN |

| | | | | | | | | | | | | | | | |
|---|---|---|---|---|---|---|---|---|---|---|---|---|---|---|---|
| A0A0G2JKZ1 | Tapasin | TAPBP | 5 | 16.7 | 0 | 7.431 | 0.74 | 0.72 | 0.80 | 0.98 | 0.99 | 1.19 | 0.90 | NaN | 0.89 |
| B3KYB6 | Phosphatidylinositol transfer protein beta isoform | PITPNB | 4 | 38.3 | 0 | 5.232 | 1.02 | 0.94 | NaN | NaN | NaN | 1.44 | NaN | NaN | 1.28 |
| A0A0A0MTH3 | Integrin-linked protein kinase | ILK | 9 | 23.6 | 0 | 15.01 | NaN | NaN | 1.94 | NaN | 0.83 | 0.80 | NaN | NaN | 1.21 |
| A0A0R4J2G3 | Neutral cholesterol ester hydrolase 1 | NCEH1 | 6 | 21.4 | 0 | 69.66 | 1.04 | 0.75 | 0.95 | 1.07 | 1.10 | 0.78 | 0.75 | 0.88 | 0.84 |
| Q5T2B5 | Cullin-2 | CUL2 | 3 | 5.7 | 0.008 | 1.484 | NaN | 0.68 | NaN | 0.69 | 1.06 | NaN | NaN | NaN | NaN |
| F6UJY9 | Hydroxypyruvate isomerase;Putative hydroxypyruvate isomerase | HYI | 4 | 38.5 | 0 | 23.56 | 1.27 | NaN | NaN | 0.64 | 0.39 | NaN | 0.95 | 1.22 | 0.81 |
| A0A0A0MTS2 | Glucose-6-phosphate isomerase | GPI | 22 | 44.9 | 0 | 323.3 | 0.94 | 0.98 | 0.98 | 1.04 | 1.01 | 1.08 | 0.95 | 1.01 | 1.06 |
| A0A0A0MTS7 | Titin | TTN | 7 | 0.3 | 5E-04 | 2.855 | 2.48 | 1.85 | 1.48 | NaN | 1.37 | NaN | NaN | 1.45 | 1.08 |
| A0A0A6YYL4 | Coronin;Coronin-7 | CORO7-P/ | 4 | 7.8 | 0 | 4.627 | 0.85 | NaN | NaN | 0.49 | 0.88 | 0.95 | NaN | NaN | NaN |
| A0A0B4J1Z1 | Serine/arginine-rich splicing factor 7 | SRSF7 | 7 | 54.7 | 0 | 12.37 | 1.11 | 1.03 | 1.02 | 0.74 | 0.77 | 1.16 | NaN | 0.79 | 1.04 |
| A0A0C4DFT3 | Disks large homolog 1 | DLG1 | 6 | 10.1 | 0 | 16.77 | 1.73 | 0.77 | 1.43 | 0.88 | NaN | 1.01 | NaN | NaN | 1.06 |
| A0A0C4DFV9 | Protein SET;Protein SETSIP | SET | 6 | 27.8 | 0 | 323.3 | 1.11 | 1.08 | 1.04 | 1.14 | 1.07 | 1.10 | 1.05 | 1.09 | 1.07 |
| C9J9K3 | 40S ribosomal protein SA | RPSA | 6 | 65.8 | 0 | 323.3 | 1.05 | 1.11 | 1.23 | 0.95 | 1.03 | 1.02 | 1.14 | 0.96 | 1.05 |
| E7ENN3 | Nesprin-1 | SYNE1 | 3 | 0.6 | 0.008 | 1.526 | 0.88 | 1.10 | 1.77 | NaN | NaN | 1.03 | NaN | NaN | NaN |
| A0A0C4DG44 | Serine protease HTRA2, mitochondrial | HTRA2 | 1 | 5.7 | 0 | 5.089 | 1.65 | 1.58 | 1.34 | NaN | 1.10 | NaN | NaN | NaN | 1.21 |
| A0A0C4DG49 | Poliovirus receptor | PVR | 1 | 3.4 | 0 | 64.55 | 1.47 | 1.59 | 1.38 | 0.99 | 1.13 | NaN | NaN | 1.06 | 1.06 |
| A0A0C4DG89 | Probable ATP-dependent RNA helicase DDX46 | DDX46 | 8 | 10.1 | 0 | 10.61 | 1.38 | 0.80 | 1.33 | 0.56 | 0.72 | 1.94 | NaN | NaN | 0.83 |
| A0A0C4DGA2 | Enoyl-CoA delta isomerase 2, mitochondrial | ECI2 | 8 | 30.2 | 0 | 14.07 | 1.03 | 0.51 | NaN | 0.60 | 0.72 | 1.12 | NaN | NaN | NaN |
| A0A0C4DGB0 | Short coiled-coil protein | SCOC | 1 | 22 | 0 | 22.86 | 0.97 | 1.31 | 1.09 | NaN | NaN | 1.13 | NaN | NaN | 1.10 |
| A0A0C4DGB5 | Calpastatin | CAST | 16 | 54 | 0 | 323.3 | 0.88 | 0.90 | 0.88 | 1.11 | 1.12 | 1.07 | 1.01 | 1.00 | 0.91 |
| A0A2R8Y8B3 | Chromodomain-helicase-DNA-binding protein 4 | CHD4 | 3 | 4.9 | 0 | 7.371 | 0.67 | 0.93 | 0.87 | NaN | NaN | NaN | 1.55 | NaN | 1.28 |
| A0A0C4DGQ | Calpain small subunit 1 | CAPNS1 | 8 | 51.9 | 0 | 323.3 | 1.00 | 0.93 | 0.98 | 1.02 | 1.10 | 1.07 | 1.16 | 1.36 | 1.09 |
| A0A0C4DGS1 | Dolichyl-diphosphooligosaccharide--protein glycosyltransferase 48 kDa subunit | DDOST | 7 | 24.4 | 0 | 36.77 | 1.08 | 0.92 | 1.13 | 1.04 | 0.90 | 1.15 | 0.99 | 0.99 | 0.99 |
| F6S8Q4 | ATP-dependent RNA helicase DDX3X;ATP-dependent RNA helicase DDX3Y | DDX3X | 19 | 40.1 | 0 | 70.33 | 1.00 | 1.09 | 1.08 | 0.78 | 1.08 | 0.97 | 1.02 | 0.92 | 0.96 |
| A0A0D9SFK2 | Unconventional myosin-XVIIIa | MYO18A | 5 | 3 | 0 | 17.1 | 0.93 | 1.06 | 0.95 | 1.07 | 0.78 | 0.92 | NaN | 1.02 | 0.98 |
| A0A0D9SFM0 | E3 ubiquitin-protein ligase | SHPRH | 3 | 2.3 | 0.008 | 1.521 | 1.21 | NaN | 1.22 | 1.54 | NaN | 1.07 | NaN | NaN | 1.11 |
| A0A1W2PNM 1 | Hydroxyacyl-coenzyme A dehydrogenase, mitochondrial | HADH | 5 | 26.2 | 0 | 43.04 | 0.81 | 0.91 | NaN | 1.14 | 1.18 | 0.96 | 1.14 | 0.85 | NaN |
| A0A1B0GWF | Syntaxin-binding protein 1 | STXBP1 | 3 | 12.2 | 0 | 4.767 | NaN | 1.68 | 1.11 | NaN | NaN | 0.95 | NaN | NaN | NaN |
| A0A140T8Z0 | Protein diaphanous homolog 1 | DIAPH1 | 16 | 16.6 | 0 | 18.03 | 0.91 | 0.75 | 0.96 | 1.00 | NaN | 0.77 | 0.99 | 0.75 | 1.03 |
| A0A0G2JHC2 | Phostensin | PPP1R18 | 2 | 4.2 | 0 | 14.06 | 0.78 | NaN | 1.21 | NaN | NaN | NaN | NaN | NaN | NaN |
| A0A0G2JIW1 | Heat shock 70 kDa protein 1B;Heat shock 70 kDa protein 1A | HSPA1B | 19 | 68.7 | 0 | 323.3 | 2.70 | 3.13 | 2.66 | 2.64 | 2.57 | 2.59 | 0.91 | 0.92 | 0.91 |

| | | | | | | | | | | | | | | | | |
|---|---|---|---|---|---|---|---|---|---|---|---|---|---|---|---|---|
| A0A0J9YYL3 | Poly(U)-binding-splicing factor PUF60 | PUF60 | 10 | 30.9 | 0 | 18.7 | 1.11 | 1.24 | NaN | 0.82 | NaN | 1.17 | NaN | 2.57 | NaN | |
| C9JB42 | DnaJ homolog subfamily B member 3;DnaJ homolog subfamily B member 6 | DNAJB6 | 1 | 19.7 | 0 | 28.37 | 2.28 | NaN | 2.53 | NaN | NaN | NaN | NaN | NaN | NaN | |
| A0A0R4J2E8 | Matrin-3 | MATR3 | 26 | 37.8 | 0 | 207.3 | 1.08 | 0.86 | 1.00 | 1.06 | 1.01 | 1.04 | 0.94 | 0.77 | 0.69 | |
| A0A0R4J2F3 | | | 1 | 18.1 | 5E-04 | 2.832 | 0.90 | NaN | 0.61 | NaN | NaN | NaN | NaN | NaN | NaN | |
| A0A0U1RQC9 | Cellular tumor antigen p53 | TP53 | 6 | 19.5 | 0 | 25.15 | 0.34 | NaN | 0.30 | NaN | NaN | NaN | NaN | NaN | 1.40 | |
| A0A0U1RR32 | Histone H2A type 1-J;Histone H2A type 1-H;Histone H2A.J;Histone H2A type 2-C;Histone H2A type 2-A;Histone H2A type 1-D;Histone H2A type 1;Histone H2A;Histone H2A type 1-C;Histone H2A type 3;Histone | HIST1H2A | 2 | 27.2 | 0 | 246.9 | 0.63 | 0.61 | 0.68 | 1.14 | 0.94 | 0.74 | 0.43 | NaN | NaN | |
| A0A0U1RRL7 | | | 3 | 87.3 | 0 | 40.97 | 1.32 | 1.16 | 1.13 | 1.18 | 0.91 | 1.30 | 1.43 | 1.32 | 1.38 | |
| A0A0U1RRM4 | Polypyrimidine tract-binding protein 1 | PTBP1 | 2 | 52.2 | 0 | 323.3 | 0.99 | 1.06 | 1.08 | 0.94 | 1.00 | 0.94 | 0.77 | 0.91 | 0.95 | |
| A0A0U1RRM | Fermitin family homolog 2 | FERMT2 | 3 | 8.8 | 0 | 61 | NaN | NaN | 1.44 | NaN | 1.41 | 1.33 | NaN | NaN | 1.06 | |
| A0A140T9I0 | HLA class I histocompatibility antigen, A-2 alpha chain;HLA class I histocompatibility antigen, A-74 alpha chain | HLA-A | 0 | 54 | 0 | 323.3 | 0.97 | 0.97 | 1.04 | 1.06 | 1.08 | 1.15 | 1.00 | 0.95 | 0.98 | |
| Q5JNW7 | Proteasome subunit beta type;Proteasome subunit beta type-8 | PSMB8 | 2 | 10.3 | 0 | 4.054 | 1.08 | NaN | 1.03 | 1.03 | NaN | 0.75 | 1.07 | NaN | 0.77 | |
| A0A140T9R1 | Flotillin-1 | FLOT1 | 6 | 21.6 | 0 | 61.35 | 0.91 | 0.75 | 1.39 | 0.82 | 0.75 | NaN | 0.91 | 1.01 | NaN | |
| A0A1B0GTG2 | Alpha-aminoadipic semialdehyde dehydrogenase | ALDH7A1 | 16 | 43.3 | 0 | 69.66 | 0.94 | 0.83 | 1.04 | 0.64 | 0.97 | 0.95 | 0.81 | 0.76 | 0.95 | |
| A0A1B0GWJ0 | Adenylosuccinate lyase | ADSL | 10 | 34.6 | 0 | 57.49 | 1.31 | 0.90 | 0.91 | 0.82 | 1.00 | 1.25 | 0.84 | 1.03 | 1.13 | |
| F5GZ78 | Paxillin | PXN | 9 | 21.2 | 0 | 159.8 | 1.29 | 1.76 | 1.72 | 1.32 | 1.33 | 1.36 | 1.30 | 0.99 | 1.73 | |
| A0A1B0GUA3 | KIF1-binding protein | KIAA1279 | 1 | 2.9 | 0 | 8.056 | 1.75 | 1.54 | NaN | NaN | NaN | 1.47 | NaN | NaN | 1.71 | |
| A0A1B0GVD5 | Cathepsin D;Cathepsin D light chain;Cathepsin D heavy chain | CTSD | 18 | 50.4 | 0 | 236.5 | 0.92 | 0.94 | 0.87 | 1.11 | 1.06 | 1.15 | 0.93 | 0.87 | 0.96 | |
| A0A1C7CYX9 | Dihydropyrimidinase-related protein 2 | DPYSL2 | 24 | 52.4 | 0 | 323.3 | 0.88 | 0.68 | 0.74 | 0.94 | 0.96 | 0.76 | 0.61 | 0.75 | 0.74 | |
| C9J4M6 | DNA-directed RNA polymerase;DNA-directed RNA polymerase II subunit RPB2 | POLR2B | 4 | 5.5 | 0 | 5.379 | NaN | NaN | NaN | NaN | NaN | NaN | 0.19 | 0.18 | NaN | |
| A0A1W2PPZ5 | Transcription elongation factor A protein 1 | TCEA1 | 3 | 12 | 0 | 4.836 | 0.91 | 0.82 | 1.25 | NaN | NaN | 1.25 | 1.08 | 1.24 | 1.04 | |
| E9PJA7 | Bcl-2-associated transcription factor 1 | BCLAF1 | 4 | 12.8 | 0 | 48.17 | 1.10 | 0.99 | 1.22 | 0.71 | 1.02 | 1.11 | 1.07 | 0.81 | 1.03 | |
| A0A1X7SBZ2 | Probable ATP-dependent RNA helicase DDX17 | DDX17 | 15 | 36.6 | 0 | 134.6 | 0.85 | 0.91 | 0.83 | 0.68 | 0.79 | 0.90 | 0.83 | 0.79 | 1.19 | |
| C9JP51 | Protein FAM131A | FAM131A | 1 | 6.1 | 0 | 2.987 | 1.44 | 1.98 | 1.63 | NaN | 2.02 | 1.61 | NaN | NaN | NaN | |

| | | | | | | | | | | | | | | |
|---|---|---|---|---|---|---|---|---|---|---|---|---|---|---|
| A0A1W2PQV2 | Glycine cleavage system H protein, mitochondrial | GCSH | 2 | 30 | 0 | 3.652 | NaN | NaN | NaN | 1.25 | 1.29 | NaN | 1.55 | 0.86 | NaN |
| A8MYT4 | Phosphatidylinositol 3-kinase;Phosphatidylinositol 3-kinase catalytic subunit type 3 | PIK3C3 | 1 | 1 | 0.009 | 1.455 | 1.04 | 1.14 | 1.10 | 1.07 | 0.98 | 1.13 | 1.11 | 1.12 | 1.37 |
| A0A2R8Y3X5 | Dynamin-like 120 kDa protein, mitochondrial;Dynamin-like 120 kDa protein, form S1 | OPA1 | 8 | 13.7 | 0 | 19.31 | 0.30 | 0.95 | NaN | NaN | NaN | 1.46 | NaN | NaN | NaN |
| A0A2R8Y430 | Glutathione synthetase | GSS | 15 | 45.1 | 0 | 80.66 | 1.15 | 0.94 | 0.97 | 1.01 | 1.10 | 0.96 | 1.07 | 1.08 | 0.99 |
| A6NNI4 | Tetraspanin;CD9 antigen | CD9 | 5 | 34 | 0 | 11.58 | 1.09 | 1.04 | 1.13 | 1.00 | 1.07 | 0.79 | 0.66 | 0.88 | 1.47 |
| A0A2R8Y484 | Leukocyte surface antigen CD47 | CD47 | 3 | 15.3 | 0 | 3.662 | 0.85 | 1.55 | 1.73 | 1.06 | 1.61 | 0.85 | 0.90 | 1.39 | 0.94 |
| H7C048 | SWI/SNF-related matrix-associated actin-dependent regulator of chromatin subfamily E member 1 | SMARCE1 | 1 | 8.3 | 0.009 | 1.431 | 0.96 | NaN | 1.14 | NaN | NaN | NaN | NaN | NaN | 0.77 |
| A0A2R8YEZ4 | DNA-directed RNA polymerases I and III subunit RPAC1 | POLR1C | 2 | 10.1 | 0 | 15.31 | 0.98 | 1.29 | NaN | NaN | 1.02 | NaN | 0.95 | 1.13 | NaN |
| A0A2R8Y7E7 | Tubulin-specific chaperone E | TBCE | 5 | 11.1 | 0 | 4.228 | 0.74 | 0.86 | 1.43 | NaN | 1.03 | NaN | NaN | NaN | 0.77 |
| A0A2R8Y5S7 | Radixin | RDX | 13 | 45.9 | 0 | 67.06 | NaN | 1.25 | 1.39 | 1.20 | 1.89 | NaN | 0.97 | 0.75 | 0.91 |
| A0A2R8Y793 | | | 1 | 90 | 0.003 | 1.862 | NaN | 0.85 | NaN | NaN | NaN | 0.91 | 0.70 | 0.92 | 0.91 |
| B1ALA9 | Ribose-phosphate pyrophosphokinase 1 | PRPS1 | 2 | 35.4 | 0 | 15.4 | 0.71 | 0.84 | 1.00 | 0.53 | 0.98 | 1.05 | 0.86 | 0.75 | 0.90 |
| A0A2R8Y811 | 40S ribosomal protein S14 | RPS14 | 8 | 41.3 | 0 | 55.93 | 0.95 | 0.91 | 0.98 | 0.86 | 0.97 | 0.82 | 0.95 | 1.02 | 0.90 |
| A0A2R8YGD1 | Tripeptidyl-peptidase 1 | TPP1 | 3 | 9.8 | 0 | 5.059 | 0.81 | NaN | NaN | NaN | 0.84 | 1.10 | NaN | NaN | 0.73 |
| A0A2R8YD50 | Peroxisomal multifunctional enzyme type 2;(3R)-hydroxyacyl-CoA dehydrogenase;Enoyl-CoA hydratase 2 | HSD17B4 | 7 | 14.8 | 0 | 12.18 | 1.06 | 1.01 | 1.49 | NaN | NaN | NaN | NaN | NaN | NaN |
| A0A2R8YF43 | Casein kinase II subunit alpha 3;Casein kinase II subunit alpha | CSNK2A1 | 5 | 23.1 | 0 | 23.85 | 1.27 | 1.04 | 1.04 | 1.16 | 1.22 | 1.40 | 0.84 | 0.80 | 1.38 |
| A0A2R8YEA7 | | | 2 | 76.4 | 0 | 92.95 | 1.76 | 1.63 | 1.69 | 1.66 | 1.73 | 1.88 | 1.90 | 1.89 | 1.37 |
| A0A2R8YEP4 | Flavin reductase (NADPH) | BLVRB | 5 | 31.4 | 0 | 54.92 | 1.25 | 1.38 | 0.94 | 1.03 | 1.24 | 1.17 | 1.20 | 1.20 | 0.83 |
| A0A2R8YFH5 | Protein transport protein Sec23B | SEC23B | 4 | 12.1 | 0 | 6.65 | NaN | 0.91 | 1.07 | 0.90 | NaN | NaN | NaN | 1.27 | 1.15 |
| A0A2U3TZH3 | Elongation factor 1-alpha 2 | EEF1A2 | 3 | 44 | 0 | 11 | 0.28 | 0.29 | NaN | 0.94 | NaN | 0.44 | NaN | NaN | 0.20 |
| E9PNW4 | CD59 glycoprotein | CD59 | 4 | 29.6 | 0 | 55.51 | 1.06 | 1.04 | 1.09 | 1.07 | 0.96 | 1.03 | 1.16 | 1.19 | 1.05 |
| V9GYY3 | C-1-tetrahydrofolate synthase, cytoplasmic;Methylenetetrahydrofolate dehydrogenase;Methenyltetrahydrofolate cyclohydrolase;Formyltetrahydrofolate synthetase;C-1-tetrahydrofolate synthase, cytoplasmic, N-terminally | MTHFD1 | 35 | 46.4 | 0 | 140.7 | 0.67 | 0.72 | 0.76 | 0.73 | 0.87 | 0.79 | 0.79 | 0.78 | 0.67 |
| A0A3B3IRI2 | CTP synthase 1;CTP synthase | CTPS1 | 11 | 25.2 | 0 | 31.69 | 1.03 | 1.16 | 1.09 | 1.03 | 0.84 | 0.90 | 1.21 | 1.16 | 1.10 |

| | | | | | | | | | | | | | | |
|---|---|---|---|---|---|---|---|---|---|---|---|---|---|---|
| A0A3B3IRK6 | Mannosyl-oligosaccharide glucosidase | MOGS | 11 | 20.6 | 0 | 27.05 | 1.20 | 1.06 | 1.11 | 1.14 | 0.82 | 1.07 | 0.91 | 1.02 | 0.97 |
| A0A3B3IRT8 | Translocon-associated protein subunit alpha | SSR1 | 4 | 20.8 | 0 | 46.35 | 0.79 | 0.88 | 0.91 | 0.92 | 0.90 | 0.86 | 1.02 | 1.03 | 1.11 |
| A0A3B3IS06 | Treacle protein | TCOF1 | 5 | 4.1 | 0 | 6.04 | 1.18 | 1.08 | 0.92 | 0.81 | NaN | 1.58 | 0.94 | 0.82 | 1.08 |
| A0A3B3IS84 | Coatomer subunit alpha;Xenin;Proxenin | COPA | 32 | 35.9 | 0 | 323.3 | 0.82 | 0.75 | 0.81 | 0.73 | 0.77 | 0.78 | 0.97 | 0.84 | 0.90 |
| B4DKB2 | Endothelin-converting enzyme 1 | ECE1 | 3 | 7.3 | 0 | 40.4 | 0.80 | NaN | 0.68 | NaN | NaN | NaN | NaN | NaN | 0.92 |
| A0A3B3ISG5 | Insulin-degrading enzyme | IDE | 3 | 5.7 | 0 | 5.832 | 0.48 | NaN | NaN | NaN | NaN | 0.77 | NaN | 0.75 | 0.89 |
| A0A3B3ISV4 | Vitamin K epoxide reductase complex subunit 1-like protein 1 | VKORC1L1 | 1 | 4.9 | 0.001 | 2.745 | 1.05 | 1.02 | 0.99 | 1.23 | 0.98 | 2.02 | 0.78 | 1.44 | 1.08 |
| A0A3B3IUC9 | Acylglycerol kinase, | AGK | 2 | 27.6 | 0.007 | 1.613 | 0.89 | NaN | 1.15 | 0.73 | NaN | 0.99 | NaN | NaN | 0.62 |
| A0A3B3IT92 | DNA replication licensing factor MCM4 | MCM4 | 10 | 17.7 | 0 | 20.16 | NaN | 0.63 | NaN | NaN | 1.03 | 0.72 | NaN | NaN | 0.67 |
| A0A3B3ITF0 | m7GpppX diphosphatase | DCPS | 3 | 17.2 | 0 | 3.726 | 0.91 | 0.92 | 1.02 | 0.57 | 0.71 | NaN | 1.00 | 0.60 | 3.48 |
| A0A3B3ITN8 | Retinoic acid-induced protein 3 | GPRC5A | 4 | 12.4 | 0 | 7.827 | NaN | NaN | NaN | 0.80 | 0.82 | 0.66 | NaN | NaN | 0.81 |
| A0A3B3ITT5 | 60S ribosomal protein L29 | RPL29 | 2 | 13.8 | 0 | 36.32 | 1.24 | 1.59 | 1.18 | 1.14 | 1.32 | 1.28 | 1.18 | 1.11 | 1.20 |
| E9PN17 | ATP synthase subunit g, mitochondrial | ATP5L | 2 | 31.6 | 0 | 4.062 | 1.54 | 1.19 | 1.16 | 0.94 | 1.54 | 1.69 | 1.10 | 0.76 | 1.94 |
| A0A3B3ITZ9 | Thyroid hormone receptor-associated protein 3 | THRAP3 | 9 | 13.8 | 0 | 17.51 | 1.25 | NaN | 1.11 | 1.27 | 0.98 | 0.90 | 1.31 | 1.33 | 1.33 |
| A0A3B3IUA2 | | NHP2L1 | 2 | 16.9 | 0 | 77.82 | 0.94 | 1.06 | 0.97 | 1.30 | 1.15 | 1.24 | 0.89 | 1.04 | 1.09 |
| A0A3B3IUB1 | Translation initiation factor eIF-2B subunit epsilon | EIF2B5 | 2 | 3 | 0 | 3.485 | 1.13 | 1.00 | 0.85 | 1.32 | 1.13 | 0.89 | 1.09 | NaN | 0.96 |
| A0A3B3IUB5 | Minor histocompatibility antigen H13 | HM13 | 7 | 20.7 | 0 | 111.4 | 1.02 | 0.91 | 0.91 | 1.36 | 1.15 | 1.04 | 1.28 | NaN | 1.66 |
| A0A3F2YNY6 | Pre-mRNA-processing factor 40 homolog A | PRPF40A | 6 | 8.4 | 0 | 14.71 | 0.51 | 0.80 | 0.49 | 0.91 | 2.07 | NaN | NaN | 0.83 | 0.71 |
| A0A494C017 | Myeloid-associated differentiation marker | MYADM | 2 | 27 | 0 | 323.3 | 1.12 | 0.94 | 0.89 | 0.87 | 1.27 | 0.87 | 3.59 | 3.89 | 1.04 |
| H0Y993 | Protein DEK | DEK | 9 | 24.2 | 0 | 26.45 | 0.66 | 0.79 | 1.01 | 0.75 | 0.76 | 1.08 | 0.91 | 0.82 | 0.65 |
| A0A494C039 | Hypoxia up-regulated protein 1 | HYOU1 | 28 | 36.9 | 0 | 209.8 | 0.84 | 0.88 | 0.83 | 0.95 | 0.90 | 0.98 | 0.97 | 0.91 | 0.85 |
| A0A494C0B4 | UV excision repair protein RAD23 homolog A | RAD23A | 4 | 20.9 | 0 | 3.135 | 0.90 | 1.02 | NaN | 1.15 | 0.84 | 0.97 | 1.17 | NaN | NaN |
| A0A494C128 | Nucleolar protein 56 | NOP56 | 3 | 8.6 | 0 | 9.37 | NaN | NaN | 0.82 | 1.33 | 1.12 | NaN | 1.18 | 1.22 | 1.06 |
| A0A494C1F2 | Nuclear pore complex protein Nup214 | NUP214 | 4 | 2.3 | 0 | 3.742 | 1.60 | 1.64 | 1.92 | 1.46 | NaN | NaN | NaN | NaN | 1.47 |
| A0A494C1K3 | General transcription factor II-I | GTF2I | 8 | 9.5 | 0 | 9.474 | NaN | NaN | NaN | NaN | 1.00 | 1.01 | NaN | NaN | NaN |
| A0A494C1L1 | Lamin-B receptor | LBR | 5 | 14.4 | 0 | 41.92 | 0.99 | 0.84 | NaN | NaN | 0.94 | 0.87 | 0.84 | NaN | NaN |
| A0A494C1N0 | Peptidyl-prolyl cis-trans isomerase FKBP2 | FKBP2 | 2 | 15.8 | 0 | 3.199 | NaN | NaN | NaN | 1.79 | 0.98 | NaN | NaN | NaN | NaN |
| A0A499FIX8 | Extended synaptotagmin-2 | ESYT2 | 15 | 25.6 | 0 | 111.8 | 0.93 | 0.87 | 1.01 | 0.67 | 0.86 | 0.99 | 0.91 | 0.78 | 1.02 |
| H3BUX2 | Cytochrome b5 type B | CYB5B | 3 | 22.9 | 0 | 9.274 | 0.71 | 1.07 | 0.89 | NaN | NaN | 0.97 | NaN | NaN | NaN |
| A0AVT1 | Ubiquitin-like modifier-activating enzyme 6 | UBA6 | 15 | 18.4 | 0 | 19.74 | NaN | NaN | 0.69 | NaN | 0.66 | 0.56 | NaN | 1.84 | NaN |
| M0R026 | Acetolactate synthase-like | ILVBL | 4 | 14.5 | 0 | 28.02 | NaN | 1.60 | 1.37 | NaN | 0.69 | NaN | NaN | NaN | NaN |
| A2A274 | Aconitate hydratase, | ACO2 | 12 | 24.2 | 0 | 44.06 | 0.74 | 0.52 | 0.84 | NaN | 0.81 | 0.82 | NaN | NaN | NaN |

| | | | | | | | | | | | | | | | |
|---|---|---|---|---|---|---|---|---|---|---|---|---|---|---|---|
| A2A2V1 | Major prion protein | PRNP | 1 | 4.8 | 0 | 6.304 | 1.48 | 1.17 | 1.02 | NaN | NaN | 1.02 | NaN | NaN | 0.95 |
| H3BTL1 | Microtubule-associated proteins 1A/1B light chain 3B;Microtubule-associated proteins 1A/1B light chain 3 beta 2 | MAP1LC3E | 1 | 18.7 | 0 | 7.386 | 1.00 | 1.54 | NaN | NaN | NaN | NaN | NaN | NaN | 0.56 |
| A6NEM2 | Host cell factor 1;HCF N-terminal chain 1;HCF N-terminal chain 2;HCF N-terminal chain 3;HCF N-terminal chain 4;HCF N-terminal chain 5;HCF N-terminal chain 6;HCF C-terminal chain 1;HCF C-terminal chain 2;HCF C-terminal chain 3;HCF C-terminal chain 4;HCF C-terminal | HCFC1 | 2 | 1.6 | 0 | 3.005 | NaN | NaN | NaN | NaN | 1.54 | NaN | 0.78 | 0.88 | NaN |
| A6NFX8 | ADP-sugar pyrophosphatase | NUDT5 | 8 | 33.2 | 0 | 59.09 | 0.91 | 0.92 | 0.72 | 1.50 | 1.29 | 1.20 | NaN | NaN | NaN |
| A6NGP5 | Hematological and neurological expressed 1-like protein | HN1L | 7 | 61.2 | 0 | 77.31 | 1.17 | 1.46 | 1.42 | 0.95 | 1.03 | 1.09 | 1.06 | 1.81 | 1.38 |
| A6NHQ2 | rRNA/tRNA 2-O-methyltransferase fibrillarin-like | FBLL1 | 1 | 6.6 | 0.001 | 2.363 | NaN | 0.98 | NaN | 0.79 | 1.04 | NaN | 0.75 | 0.97 | NaN |
| J3KTL8 | Structural maintenance of chromosomes flexible hinge domain-containing protein 1 | SMCHD1 | 1 | 0.7 | 0.002 | 1.953 | 0.78 | 0.85 | 0.75 | NaN | NaN | 0.77 | NaN | NaN | 0.97 |
| A6NLH6 | Protein cornichon homolog 4 | CNIH4 | 1 | 14.6 | 0 | 15.56 | NaN | 0.68 | NaN | 1.24 | 1.09 | 1.17 | NaN | NaN | NaN |
| E9PJK1 | Tetraspanin;CD81 antigen | CD81 | 3 | 35.8 | 0 | 40.33 | 1.09 | 1.02 | 1.07 | NaN | NaN | 1.26 | 0.85 | 0.74 | 1.19 |
| A6NMQ3 | Alpha-endosulfine | ENSA | 3 | 32.9 | 0 | 180 | NaN | NaN | 1.27 | NaN | NaN | 1.54 | NaN | 1.62 | 4.03 |
| Q5T949 | Serine/threonine-protein phosphatase 2A activator | PPP2R4 | 3 | 17.6 | 0.001 | 2.754 | 1.18 | NaN | NaN | NaN | 1.59 | 1.13 | 1.04 | 1.41 | 0.94 |
| A8K878 | Mesencephalic astrocyte-derived neurotrophic factor | MANF | 7 | 41.1 | 0 | 11.72 | 1.71 | 1.19 | 2.74 | 1.15 | 1.02 | 1.31 | 0.88 | NaN | 1.59 |
| A8MUA9 | Small ubiquitin-related modifier 3 | SUMO3 | 1 | 15.6 | 1E-03 | 2.3 | NaN | 0.91 | 1.12 | 0.85 | 1.06 | 0.74 | 0.89 | 0.96 | 1.13 |
| P62308 | Small nuclear ribonucleoprotein G;Putative small nuclear ribonucleoprotein G-like protein | SNRPG | 3 | 35.5 | 0 | 24.09 | 0.97 | 1.08 | 0.98 | 1.17 | 1.08 | 1.27 | 1.20 | 0.85 | 1.06 |
| J3QRI9 | Developmentally-regulated GTP-binding protein 2 | DRG2 | 3 | 25.6 | 0 | 7.428 | 0.91 | 1.00 | 1.08 | NaN | 1.12 | 0.82 | NaN | NaN | 1.00 |
| A8MZH8 | Pituitary tumor-transforming gene 1 protein-interacting protein | PTTG1IP | 2 | 23.4 | 0 | 3.994 | 1.14 | 1.53 | 1.30 | 1.10 | 1.53 | 1.35 | NaN | 1.00 | 1.38 |
| B0QY89 | Eukaryotic translation initiation factor 3 subunit L | EIF3L | 15 | 31.3 | 0 | 184.3 | 1.09 | 1.13 | 0.98 | 1.06 | 1.03 | 0.96 | NaN | 0.78 | 0.96 |
| B0QYK0 | RNA-binding protein EWS | EWSR1 | 6 | 14.6 | 0 | 44.06 | 0.94 | 0.98 | 1.01 | 1.07 | 1.03 | 1.00 | 0.73 | 0.99 | 0.93 |
| H3BQQ9 | SUMO-conjugating enzyme | UBE2I | 5 | 51.1 | 0 | 11.52 | 0.73 | 0.74 | NaN | NaN | 0.85 | 0.71 | 0.79 | NaN | 0.50 |
| B0QZ36 | mRNA export factor | RAE1 | 3 | 38.7 | 0 | 7.664 | 1.18 | 1.20 | NaN | NaN | NaN | 1.41 | NaN | NaN | 1.49 |
| B0YIW6 | Coatomer subunit delta | ARCN1 | 21 | 43.7 | 0 | 114.8 | 0.89 | 0.93 | 1.00 | 0.96 | 1.02 | 0.90 | 1.04 | 1.03 | 0.86 |
| B1AHB1 | DNA helicase;DNA replication licensing factor MCM5 | MCM5 | 9 | 16.6 | 0 | 6.905 | 0.62 | 0.72 | 0.67 | 1.01 | 0.85 | 0.86 | NaN | 0.83 | 0.77 |

| | | | | | | | | | | | | | | |
|---|---|---|---|---|---|---|---|---|---|---|---|---|---|---|
| B1AJY7 | 26S proteasome non-ATPase regulatory subunit 10 | PSMD10 | 4 | 32.6 | 0 | 170.7 | NaN | NaN | NaN | NaN | 1.09 | NaN | NaN | 1.15 | 3.07 |
| B1AK87 | | CAPZB | 3 | 56.5 | 0 | 158.2 | 1.07 | 1.09 | 1.03 | 0.99 | 0.92 | 1.04 | 1.10 | 0.85 | 1.00 |
| B1AKD8 | Rootletin | CROCC | 1 | 0.6 | 0.009 | 1.407 | 1.03 | 0.97 | 1.02 | 0.99 | 0.96 | 1.02 | 0.93 | 0.06 | 0.95 |
| B1AKJ5 | Nardilysin | NRD1 | 3 | 3.3 | 0 | 3.554 | NaN | 0.54 | 0.47 | NaN | 0.55 | NaN | NaN | NaN | NaN |
| B3KT28 | FAS-associated factor 1 | FAF1 | 1 | 1.9 | 0.004 | 1.774 | 0.63 | 1.30 | NaN | NaN | NaN | NaN | NaN | NaN | NaN |
| B1ANR0 | Polyadenylate-binding protein;Polyadenylate-binding protein 4 | PABPC4 | 6 | 28.9 | 0 | 8.143 | NaN | 1.08 | 0.56 | 0.86 | NaN | 1.95 | NaN | 0.81 | 2.04 |
| H3BLV0 | Complement decay-accelerating factor | CD55 | 9 | 31.9 | 0 | 30.26 | NaN | 1.21 | 1.10 | 0.99 | 1.11 | 1.24 | 0.90 | 0.98 | 1.00 |
| B4DG22 | Ribosomal protein S6 kinase alpha-3;Ribosomal protein S6 kinase;Ribosomal protein S6 kinase alpha-2;Ribosomal protein S6 kinase alpha-1 | RPS6KA3 | 3 | 5.5 | 0.001 | 2.448 | 1.62 | 1.03 | 0.83 | NaN | NaN | 0.92 | NaN | NaN | 1.54 |
| G3V4W0 | Heterogeneous nuclear ribonucleoproteins C1/C2;Heterogeneous nuclear ribonucleoprotein C-like 1;Heterogeneous nuclear ribonucleoprotein C-like 2 | HNRNPC | 22 | 55 | 0 | 323.3 | 0.80 | 0.82 | 0.79 | 0.75 | 0.83 | 0.82 | 0.89 | 0.94 | 0.89 |
| B3KS98 | Eukaryotic translation initiation factor 3 subunit H | EIF3H | 6 | 34.4 | 0 | 323.3 | 1.20 | 1.10 | 1.09 | 1.12 | 1.19 | 0.92 | 0.91 | 1.14 | 0.66 |
| M0QZP4 | Branched-chain-amino-acid aminotransferase;Branched-chain-amino-acid aminotransferase, mitochondrial | BCAT2 | 4 | 17.9 | 0 | 17.89 | 1.28 | 0.86 | 1.12 | 0.90 | 1.00 | 0.89 | 1.19 | 1.02 | 1.27 |
| B4DDD8 | Histidine--tRNA ligase, | HARS | 12 | 33.8 | 0 | 30.35 | 0.79 | 0.68 | 0.63 | NaN | 0.74 | 0.68 | NaN | NaN | 0.76 |
| B4DDF4 | Calponin;Calponin-2 | CNN2 | 12 | 55.4 | 0 | 323.3 | 1.16 | 1.03 | 1.16 | 1.07 | 1.14 | 1.08 | 1.14 | 1.01 | 1.12 |
| U3KQP3 | NADH dehydrogenase [ubiquinone] 1 alpha subcomplex subunit 13 | NDUFA13 | 1 | 18.7 | 0.008 | 1.546 | 0.56 | 0.56 | 0.66 | NaN | 0.57 | 0.56 | 0.82 | 1.06 | 0.64 |
| H0YKU5 | COP9 signalosome complex subunit 2 | COPS2 | 3 | 12.5 | 0 | 3.914 | NaN | NaN | NaN | NaN | 1.78 | 0.68 | NaN | 1.25 | 0.88 |
| B4DJ81 | NADH-ubiquinone oxidoreductase 75 kDa subunit, | NDUFS1 | 9 | 24.5 | 0 | 13.34 | 1.12 | 0.83 | 0.72 | NaN | 0.63 | NaN | NaN | NaN | NaN |
| B4DJV2 | Citrate synthase;Citrate synthase, mitochondrial | CS | 11 | 41.1 | 0 | 306.5 | 1.14 | 1.06 | 1.14 | 0.97 | 1.10 | 1.05 | 0.95 | 0.95 | 0.99 |
| B4DKY1 | Cysteine--tRNA ligase, cytoplasmic | CARS | 6 | 10.1 | 0 | 6.645 | 0.69 | 0.69 | 1.00 | NaN | NaN | NaN | NaN | NaN | NaN |
| B4DLN1 | 39S ribosomal protein L12, mitochondrial | MRPL12 | 7 | 22.2 | 0 | 56.66 | 0.80 | 0.90 | 0.84 | 1.08 | 1.16 | 0.84 | 1.04 | 0.95 | 0.97 |
| B4DR61 | Protein transport protein Sec61 subunit alpha isoform 1 | SEC61A1 | 5 | 10.4 | 0 | 7.812 | NaN | 0.67 | NaN | 1.20 | 0.85 | 1.21 | 0.89 | 0.58 | NaN |

| | | | | | | | | | | | | | | |
|---|---|---|---|---|---|---|---|---|---|---|---|---|---|---|
| H0Y630 | Serine/threonine-protein kinase 24;Serine/threonine-protein kinase 24 36 kDa subunit;Serine/threonine-protein kinase 24 12 kDa subunit | STK24 | 5 | 22 | 0 | 7.651 | 1.00 | 1.06 | 0.87 | 0.91 | 0.82 | 0.79 | 0.76 | 0.68 | 1.13 |
| B4DY09 | Interleukin enhancer-binding factor 2 | ILF2 | 17 | 66.8 | 0 | 121.4 | 0.95 | 0.90 | 0.96 | 0.90 | 0.89 | 0.96 | 1.00 | 1.00 | 0.94 |
| B4E0K5 | Mitogen-activated protein kinase 14 | MAPK14 | 4 | 15.5 | 0 | 5.133 | 0.94 | NaN | 0.73 | NaN | NaN | 0.99 | 0.69 | NaN | 1.14 |
| B5MBZ0 | Echinoderm microtubule-associated protein-like 4 | EML4 | 2 | 2.8 | 1E-03 | 2.285 | NaN | NaN | NaN | 0.60 | NaN | NaN | 0.97 | 0.60 | NaN |
| B5MBZ8 | Protein phosphatase 1 regulatory subunit 7 | PPP1R7 | 3 | 17.9 | 0 | 19.14 | NaN | NaN | NaN | NaN | NaN | NaN | 1.37 | NaN | 0.78 |
| B5MC59 | Replication protein A 14 kDa subunit | RPA3 | 3 | 52.4 | 0 | 5.935 | 1.17 | 1.26 | 1.30 | 1.65 | NaN | 1.59 | NaN | 0.51 | 1.07 |
| B5MDF5 | GTP-binding nuclear protein Ran | RAN | 10 | 41.2 | 0 | 151.3 | 1.03 | 0.93 | 0.94 | 0.93 | 0.94 | 0.92 | 0.92 | 0.97 | 0.90 |
| E7EW69 | Septin-10 | SEPT10 | 5 | 24.4 | 0 | 70.93 | NaN | 1.29 | 0.93 | 1.10 | 0.94 | 1.21 | 0.80 | 0.77 | 1.49 |
| B7Z4M1 | Reticulon | RTN3 | 1 | 12.2 | 0 | 67.28 | 0.98 | 1.29 | 1.02 | NaN | 1.26 | 1.15 | NaN | NaN | 1.36 |
| B7Z7F3 | Ran-binding protein 3 | RANBP3 | 3 | 9.9 | 0 | 2.945 | 1.15 | 1.26 | NaN | NaN | NaN | 0.88 | NaN | NaN | NaN |
| B7Z7P8 | Eukaryotic peptide chain release factor subunit 1 | ETF1 | 9 | 25.8 | 0 | 16.54 | 1.26 | 0.83 | 1.11 | 0.92 | 1.18 | 0.94 | 0.99 | 0.99 | 0.92 |
| B7ZC38 | Endophilin-B2 | SH3GLB2 | 6 | 22.8 | 0 | 20.72 | 1.01 | NaN | 0.99 | 0.97 | 1.09 | 1.08 | 1.14 | 1.01 | NaN |
| B8ZZN6 | Small ubiquitin-related modifier 1 | SUMO1 | 3 | 25.3 | 0 | 7.793 | 0.60 | NaN | 0.67 | 0.60 | NaN | 0.65 | 0.74 | 0.88 | 0.81 |
| B8ZZQ6 | Prothymosin alpha;Prothymosin alpha, N-terminally processed;Thymosin alpha-1 | PTMA | 7 | 37.4 | 0 | 323.3 | 0.97 | 1.06 | 1.01 | 0.97 | 0.99 | 0.91 | 1.00 | 1.06 | 0.98 |
| B8ZZU8 | Transcription elongation factor B polypeptide 2 | TCEB2 | 5 | 79.6 | 0 | 51.56 | 1.08 | 0.83 | 1.12 | 1.01 | 1.13 | 1.06 | 0.86 | 0.97 | 1.01 |
| C9J0K6 | Sorcin | SRI | 7 | 55.5 | 0 | 43.71 | 1.01 | 1.15 | 1.03 | 1.17 | 1.24 | 1.40 | NaN | 1.08 | 1.10 |
| D6RBD7 | Eukaryotic translation elongation factor 1 epsilon-1 | EEF1E1 | 3 | 30.7 | 0 | 41.23 | NaN | 1.08 | 0.93 | NaN | NaN | 0.88 | NaN | 0.54 | 0.84 |
| C9J1Z8 | ADP-ribosylation factor 5 | ARF5 | 3 | 67.3 | 0 | 4.913 | 0.81 | 0.89 | 0.87 | 0.62 | 0.79 | 0.80 | 0.93 | 0.99 | 1.17 |
| C9J4Z3 | 60S ribosomal protein L37a | RPL37A | 4 | 69.1 | 0 | 18.85 | 0.82 | 0.73 | 0.73 | 0.80 | 0.92 | 0.78 | 0.91 | 0.63 | 0.83 |
| C9JA28 | Translocon-associated protein subunit gamma | SSR3 | 1 | 8 | 0 | 29.45 | NaN | NaN | 1.16 | 0.69 | NaN | 0.98 | NaN | 0.76 | 1.19 |
| C9JAF7 | Synaptosomal-associated protein;Synaptosomal-associated protein 29 | SNAP29 | 2 | 15 | 0 | 2.948 | NaN | NaN | NaN | 0.88 | 0.70 | NaN | NaN | NaN | NaN |
| C9JBI3 | Phosphoserine phosphatase | PSPH | 2 | 12.3 | 1E-03 | 2.167 | 1.32 | 1.31 | 1.33 | 1.65 | 1.52 | 1.46 | 0.78 | 0.96 | 0.91 |
| E9PNC7 | Dr1-associated corepressor | DRAP1 | 2 | 14.8 | 0.001 | 2.632 | 0.97 | 0.98 | 1.12 | NaN | 1.13 | 0.89 | NaN | NaN | 1.00 |
| C9JCD9 | Protein BUD31 homolog | BUD31 | 2 | 29.1 | 0.008 | 1.526 | 1.01 | 1.30 | 0.89 | 1.15 | 1.61 | 1.05 | 0.92 | 0.98 | 1.21 |
| C9JFE4 | COP9 signalosome complex subunit 1 | GPS1 | 6 | 18 | 0 | 25.62 | 0.73 | 1.11 | 0.89 | 0.90 | 0.70 | 0.75 | 1.12 | NaN | 0.95 |
| C9JFM5 | Syntaxin-4 | STX4 | 2 | 17.1 | 0 | 14.69 | 1.21 | 1.17 | NaN | NaN | 1.22 | 1.64 | NaN | NaN | 0.99 |
| C9JFR7 | Cytochrome c | CYCS | 6 | 57.4 | 0 | 39.05 | 1.01 | 1.05 | 0.92 | 0.95 | 1.21 | 1.11 | 0.81 | 0.59 | 0.69 |
| C9JG08 | | | 2 | 0.6 | 0.008 | 1.485 | 1.29 | 1.02 | 1.14 | NaN | 1.31 | 1.21 | NaN | NaN | NaN |

| | | | | | | | | | | | | | | | |
|---|---|---|---|---|---|---|---|---|---|---|---|---|---|---|---|
| C9JJT5 | ATP synthase subunit f, mitochondrial | ATP5J2-PT | 2 | 44.4 | 0 | 4.72 | 0.98 | NaN | 1.08 | 1.07 | 1.01 | 1.04 | NaN | 0.89 | 0.93 |
| C9JXB8 | 60S ribosomal protein L24 | RPL24 | 6 | 44.6 | 0 | 35.58 | 0.83 | 0.91 | 0.85 | 1.06 | 0.96 | 0.99 | 0.86 | 1.04 | 0.78 |
| C9JRR5 | Protein PTHB1 | BBS9 | 1 | 15.8 | 1E-03 | 2.311 | 0.76 | 0.76 | 0.77 | 0.95 | 0.95 | 0.93 | 0.66 | 0.63 | 0.63 |
| C9JRZ6 | MICOS complex subunit MIC19 | CHCHD3 | 9 | 28.9 | 0 | 34.38 | 1.01 | 1.08 | 1.13 | 1.03 | 1.11 | 0.98 | 0.94 | 0.91 | 0.89 |
| C9JSF2 | Heat shock factor 2-binding protein | HSF2BP | 1 | 4.4 | 0.002 | 1.993 | 1.29 | 1.29 | 1.15 | 1.20 | 1.10 | 1.17 | 1.45 | 1.26 | 1.35 |
| C9K055 | Prenylcysteine oxidase 1 | PCYOX1 | 3 | 15.3 | 0 | 3.014 | 0.79 | 0.97 | 0.95 | 0.74 | 0.86 | 1.18 | 0.64 | 0.55 | 0.77 |
| C9K0J5 | Ras-associated and pleckstrin homology domains-containing protein 1 | RAPH1 | 4 | 4.8 | 0 | 6.37 | NaN | 1.72 | 1.46 | 0.70 | 0.61 | NaN | 0.98 | NaN | 0.87 |
| F6U1T9 | Calcineurin subunit B type 1 | PPP3R1 | 5 | 55.6 | 0 | 42.48 | 0.89 | 0.96 | 1.20 | 0.96 | 0.73 | 0.97 | 0.97 | NaN | 0.77 |
| D3YTB1 | 60S ribosomal protein L32 | RPL32 | 7 | 45.1 | 0 | 55.43 | 0.75 | 0.77 | 0.89 | 1.00 | 0.90 | 0.85 | 1.08 | 1.04 | 0.91 |
| D6R967 | Inorganic pyrophosphatase 2, mitochondrial | PPA2 | 4 | 28.3 | 0 | 10.14 | 0.76 | 0.59 | NaN | 1.32 | 1.18 | 1.02 | NaN | NaN | 1.15 |
| D6R9P3 | Heterogeneous nuclear ribonucleoprotein A/B | HNRNPAB | 8 | 31.8 | 0 | 236.2 | 0.89 | 0.89 | 0.84 | 1.28 | 0.94 | 1.01 | 1.32 | 0.88 | 0.84 |
| D6RBQ9 | | HNRNPD | 1 | 40 | 0 | 35.16 | 0.96 | 0.81 | 0.79 | 0.97 | 0.91 | 1.16 | 0.93 | 0.84 | 0.94 |
| D6RBT3 | NADH dehydrogenase [ubiquinone] iron-sulfur protein 6, mitochondrial | NDUFS6 | 1 | 8.7 | 0.004 | 1.782 | 1.32 | NaN | 0.91 | NaN | NaN | 1.84 | NaN | NaN | 1.35 |
| D6RBV2 | Vesicular integral-membrane protein VIP36 | LMAN2 | 5 | 26.2 | 0 | 5.502 | NaN | 1.08 | 0.96 | NaN | 1.12 | 1.04 | 1.00 | 1.24 | 1.14 |
| D6REM1 | Golgi phosphoprotein 3 | GOLPH3 | 1 | 17.2 | 0 | 203.9 | 0.87 | 1.05 | 0.74 | 0.91 | NaN | NaN | NaN | 0.89 | 1.02 |
| D6RGI3 | Septin-11 | SEPT11 | 10 | 35.5 | 0 | 139.3 | 1.03 | 1.32 | 1.06 | 1.05 | 1.14 | 0.94 | 1.24 | 1.01 | 1.35 |
| D6REX3 | Protein transport protein Sec31A | SEC31A | 8 | 8 | 0 | 15.66 | 0.80 | 0.79 | NaN | NaN | 0.56 | 0.48 | 0.57 | 0.94 | 0.91 |
| D6RFI1 | Drebrin | DBN1 | 3 | 33.1 | 0 | 3.437 | 1.03 | 1.13 | 0.99 | 0.72 | NaN | NaN | NaN | 0.81 | NaN |
| D6RFM5 | Succinate dehydrogenase [ubiquinone] flavoprotein subunit, mitochondrial | SDHA | 10 | 27.6 | 0 | 122.6 | 0.79 | 1.00 | 0.89 | 0.68 | 0.84 | 0.84 | 0.83 | 0.76 | 1.05 |
| D6RG15 | Twinfilin-2 | TWF2 | 3 | 24 | 0 | 4.087 | NaN | NaN | 0.91 | NaN | NaN | NaN | 1.12 | 0.86 | 1.67 |
| E5RFP0 | NudC domain-containing protein | NUDCD2 | 1 | 8.3 | 5E-04 | 2.834 | NaN | 1.32 | NaN | 1.23 | 1.36 | 1.17 | NaN | NaN | NaN |
| E5RGN3 | Copper transport protein ATOX1 | ATOX1 | 4 | 45.8 | 0 | 33.52 | 1.14 | 0.77 | 1.18 | NaN | 0.92 | 0.88 | 1.70 | NaN | 0.93 |
| E5RGS4 | Prefoldin subunit 1 | PFDN1 | 3 | 23.9 | 0 | 3.332 | NaN | 0.29 | NaN | NaN | 0.93 | 0.84 | 1.49 | 1.63 | NaN |
| E5RGX5 | Stathmin;Stathmin-2 | STMN2 | 1 | 16.7 | 0 | 70.07 | 0.83 | 0.87 | 0.85 | 0.86 | 0.91 | 0.86 | 0.94 | 0.90 | 0.97 |
| E5RHG8 | Transcription elongation factor B polypeptide 1 | TCEB1 | 4 | 62.9 | 0 | 126.9 | 0.98 | 1.08 | 1.15 | 0.95 | 1.04 | 0.95 | 0.97 | NaN | 0.91 |
| E5RHT6 | ADP-ribosylation factor GTPase-activating protein 1 | ARFGAP1 | 3 | 19.7 | 0 | 4.239 | 1.07 | 0.85 | NaN | NaN | NaN | NaN | NaN | NaN | NaN |
| E5RIW3 | Tubulin-specific chaperone A | TBCA | 10 | 76.2 | 0 | 88.15 | 1.03 | 1.11 | 1.23 | 0.91 | 0.98 | 1.01 | 0.73 | 0.61 | 0.94 |
| E5RJR5 | S-phase kinase-associated protein 1 | SKP1 | 5 | 38.7 | 0 | 73.37 | 1.20 | 1.37 | 1.47 | 0.96 | 1.12 | 1.08 | 0.99 | 0.77 | 2.10 |
| E7EM64 | COP9 signalosome complex subunit 6 | COPS6 | 3 | 13.8 | 0 | 3.158 | NaN | NaN | NaN | NaN | 1.00 | 1.84 | NaN | NaN | 0.90 |
| J3QLD9 | Flotillin-2 | FLOT2 | 10 | 29.7 | 0 | 27.28 | 0.66 | 0.70 | 0.71 | 0.73 | 0.77 | 0.98 | 0.81 | 0.73 | 0.73 |
| J3KMY5 | Epididymal secretory protein E1 | NPC2 | 5 | 43.9 | 0 | 80.67 | 1.36 | 1.37 | 1.38 | 1.06 | 1.05 | 1.06 | 1.38 | 1.40 | 1.51 |
| K7EJL1 | AP-1 complex subunit mu-1 | AP1M1 | 5 | 19.1 | 0 | 3.592 | 0.83 | 0.69 | 0.76 | NaN | NaN | 0.77 | NaN | NaN | 0.57 |

| | | | | | | | | | | | | | | |
|---|---|---|---|---|---|---|---|---|---|---|---|---|---|---|
| I3L4G9 | Phosphoribosyl pyrophosphate synthase-associated protein 2 | PRPSAP2 | 1 | 21.7 | 0.008 | 1.493 | 0.83 | 0.83 | NaN | NaN | NaN | NaN | NaN | NaN | 0.68 |
| E7ES33 | Septin-7 | SEPT7 | 15 | 43.6 | 0 | 168.6 | 1.07 | 1.12 | 1.08 | 1.00 | 0.97 | 0.95 | 1.06 | 0.95 | 0.97 |
| E7EPN9 | Protein PRRC2C | PRRC2C | 5 | 3.2 | 0 | 16.21 | 1.23 | NaN | 1.35 | 1.00 | 1.10 | 1.00 | NaN | 0.99 | 1.30 |
| E7EPT4 | NADH dehydrogenase [ubiquinone] flavoprotein 2, mitochondrial | NDUFV2 | 2 | 9.1 | 1E-03 | 2.259 | NaN | 1.25 | 1.09 | NaN | NaN | 0.98 | NaN | NaN | NaN |
| E7EQ69 | N-alpha-acetyltransferase 50 | NAA50 | 3 | 20.2 | 0 | 3.046 | 1.14 | 1.29 | 1.11 | 0.81 | NaN | NaN | 0.77 | 1.16 | 1.04 |
| E7EQR4 | Ezrin | EZR | 31 | 64 | 0 | 323.3 | 0.90 | 0.88 | 0.87 | 0.92 | 0.97 | 0.93 | 0.81 | 0.96 | 0.83 |
| S4R3H4 | Apoptotic chromatin condensation inducer in the | ACIN1 | 9 | 10.1 | 0 | 28.77 | 1.20 | 0.96 | 1.02 | 1.12 | 0.65 | 0.70 | 1.78 | 1.01 | 1.33 |
| E7EQY1 | Protein FAM136A | FAM136A | 4 | 19.6 | 0 | 132.3 | NaN | 0.64 | NaN | NaN | 1.31 | 1.16 | NaN | NaN | NaN |
| E7ESI2 | Cyclin-dependent kinase 2;Cyclin-dependent kinase 3 | CDK2 | 2 | 20.6 | 0 | 4.727 | NaN | NaN | NaN | 0.91 | 0.69 | NaN | NaN | NaN | NaN |
| E7ETU9 | Procollagen-lysine,2-oxoglutarate 5-dioxygenase 2 | PLOD2 | 16 | 30.3 | 0 | 82.36 | 1.63 | 1.50 | 1.37 | 1.19 | 1.60 | 1.32 | 2.03 | 1.54 | 1.02 |
| E7ETZ4 | Basic leucine zipper and W2 domain-containing protein 2 | BZW2 | 2 | 10 | 0 | 63.02 | NaN | 0.97 | 1.20 | 0.92 | 0.97 | 0.85 | 0.93 | 0.90 | 0.87 |
| E7EUU4 | Eukaryotic translation initiation factor 4 gamma 1 | EIF4G1 | 30 | 29.4 | 0 | 323.3 | 1.04 | 1.04 | 0.99 | 1.04 | 0.87 | 1.03 | 0.89 | 0.97 | 0.90 |
| G5E9S8 | Kinesin light chain 1 | KLC1 | 6 | 17.1 | 0 | 6.55 | NaN | 0.57 | 0.94 | NaN | NaN | 0.70 | 0.75 | 0.72 | NaN |
| E7EWB4 | Dihydropyrimidinase-related protein 5 | DPYSL5 | 1 | 3.7 | 0.007 | 1.655 | NaN | 0.97 | 0.96 | 0.63 | NaN | 0.71 | 0.91 | 0.87 | 0.94 |
| E7EWR4 | Cleavage stimulation factor subunit 2;Cleavage stimulation factor subunit 2 tau variant | CSTF2 | 6 | 14.4 | 0 | 5.016 | 0.67 | 1.24 | 1.02 | NaN | NaN | 1.21 | NaN | NaN | 0.64 |
| E7EX17 | Eukaryotic translation initiation factor 4B | EIF4B | 16 | 31.5 | 0 | 323.3 | 1.24 | 1.22 | 1.24 | 0.98 | 1.08 | 1.18 | 1.04 | 0.98 | 1.06 |
| E7EX90 | Dynactin subunit 1 | DCTN1 | 18 | 19.8 | 0 | 76.36 | 0.83 | 0.73 | 1.17 | 0.98 | 0.71 | 0.87 | 0.94 | 0.98 | 0.77 |
| E7EXA3 | NIF3-like protein 1 | NIF3L1 | 1 | 5.3 | 1E-03 | 2.115 | NaN | NaN | NaN | 1.17 | NaN | 0.76 | NaN | NaN | 0.60 |
| F8VZJ2 | Nascent polypeptide-associated complex subunit alpha;Nascent polypeptide-associated complex subunit alpha, muscle-specific form | NACA | 5 | 45.6 | 0 | 122.3 | 1.22 | 1.16 | 1.21 | 1.05 | 0.96 | 1.07 | 1.04 | 1.11 | 1.12 |
| E9PBT8 | Sulfatase-modifying factor 2 | SUMF2 | 3 | 17.2 | 0 | 16.87 | 0.94 | 1.05 | 0.97 | NaN | 1.73 | NaN | 1.15 | NaN | 0.60 |
| E9PC52 | Histone-binding protein RBBP7 | RBBP7 | 6 | 35.8 | 0 | 54.82 | 0.67 | 0.64 | 0.90 | 0.77 | 0.80 | 0.86 | 0.91 | 0.87 | 0.99 |
| E9PCR7 | 2-oxoglutarate dehydrogenase, mitochondrial | OGDH | 8 | 17.3 | 0 | 21.72 | NaN | 1.00 | 0.81 | 1.01 | 0.57 | 0.92 | 0.65 | 0.83 | 1.15 |
| E9PCY7 | Heterogeneous nuclear ribonucleoprotein H;Heterogeneous nuclear ribonucleoprotein H, N-terminally processed | HNRNPH1 | 8 | 47.8 | 0 | 323.3 | 0.88 | 0.88 | 0.91 | 0.85 | 0.85 | 0.87 | 0.97 | 0.99 | 0.96 |

| | | | | | | | | | | | | | | | |
|---|---|---|---|---|---|---|---|---|---|---|---|---|---|---|---|
| E9PD53 | Structural maintenance of chromosomes protein;Structural maintenance of chromosomes protein 4 | SMC4 | 8 | 7.4 | 0 | 14.87 | NaN | 0.83 | 0.76 | 0.73 | NaN | 0.80 | NaN | 0.89 | NaN |
| E9PDF6 | Unconventional myosin-Ib | MYO1B | 5 | 7.1 | 0 | 6.527 | NaN | 0.71 | 0.94 | NaN | 1.06 | 0.83 | 0.96 | NaN | 0.88 |
| E9PEB5 | Far upstream element-binding protein 1 | FUBP1 | 11 | 22.7 | 0 | 50.74 | 1.01 | 0.88 | 0.95 | 0.98 | 1.04 | 0.93 | 0.88 | 0.84 | 1.16 |
| E9PF10 | Nuclear pore complex protein Nup155 | NUP155 | 4 | 3.8 | 0 | 5.073 | 0.59 | NaN | 0.65 | 0.84 | NaN | 1.12 | 1.05 | 1.13 | 0.81 |
| E9PF63 | Rho-associated protein kinase 2 | ROCK2 | 4 | 5.5 | 0 | 13.93 | 1.55 | 2.01 | 1.55 | NaN | NaN | 0.50 | 0.85 | 0.62 | 1.09 |
| E9PFH4 | Transportin-3 | TNPO3 | 3 | 5.3 | 0 | 13.85 | 1.22 | 1.34 | NaN | NaN | 0.96 | NaN | NaN | NaN | NaN |
| E9PFP8 | Poly(rC)-binding protein 3 | PCBP3 | 0 | 20.2 | 0 | 20.36 | NaN | 1.40 | NaN | 1.18 | 1.13 | NaN | NaN | NaN | 0.88 |
| E9PFR3 | Serine/threonine-protein phosphatase 2A 56 kDa regulatory subunit delta isoform | PPP2R5D | 10 | 22.7 | 0 | 53.85 | 0.94 | 0.69 | 1.03 | 1.00 | 1.12 | 0.99 | 1.02 | 1.05 | 0.86 |
| E9PGC0 | Ras GTPase-activating protein 1 | RASA1 | 4 | 8.2 | 0 | 8.536 | 0.51 | NaN | NaN | 0.74 | 0.73 | NaN | 0.93 | 1.04 | NaN |
| E9PGT1 | Translin | TSN | 6 | 42.6 | 0 | 19.55 | 0.99 | 0.88 | 0.97 | 0.83 | 0.90 | 0.86 | 0.69 | 0.79 | 0.72 |
| E9PGZ1 | Caldesmon | CALD1 | 19 | 38.1 | 0 | 174.1 | 1.08 | 0.90 | 1.20 | 0.79 | 0.96 | 0.62 | 1.12 | 1.11 | 1.31 |
| E9PHI4 | SUN domain-containing protein 1 | SUN1 | 3 | 5.7 | 5E-04 | 2.766 | 1.09 | 0.89 | 1.22 | NaN | NaN | NaN | NaN | NaN | 0.83 |
| E9PHY5 | Band 4.1-like protein 2 | EPB41L2 | 13 | 20.2 | 0 | 14.33 | 0.86 | 0.99 | 0.89 | 1.15 | 0.99 | 0.66 | 3.08 | 0.83 | 0.83 |
| E9PIQ8 | Stromal interaction molecule 1 | STIM1 | 2 | 16.5 | 0.001 | 2.726 | NaN | NaN | NaN | 0.93 | 0.98 | NaN | NaN | NaN | NaN |
| E9PK01 | Elongation factor 1-delta | EEF1D | 4 | 57.9 | 0 | 323.3 | 1.19 | 1.16 | 1.09 | 1.18 | 1.12 | 1.10 | 0.83 | 0.93 | 1.03 |
| E9PK54 | | HSPA8 | 1 | 88 | 0 | 6.235 | 1.62 | 1.61 | 1.56 | 1.53 | 1.48 | 1.42 | 1.11 | 1.03 | 1.06 |
| E9PKG1 | Protein arginine N-methyltransferase 1 | PRMT1 | 13 | 45.8 | 0 | 109.6 | 1.04 | 1.09 | 0.92 | 1.14 | 1.07 | 1.29 | 1.03 | 1.01 | 0.96 |
| E9PN51 | NADH dehydrogenase [ubiquinone] iron-sulfur protein 8, mitochondrial | NDUFS8 | 1 | 8.2 | 0.001 | 2.11 | 1.09 | 1.13 | 1.00 | 1.12 | NaN | 1.04 | NaN | 1.03 | 1.13 |
| E9PKZ0 | 60S ribosomal protein L8 | RPL8 | 6 | 32.7 | 0 | 176.2 | 0.98 | 0.97 | 0.93 | 0.86 | 0.94 | 0.81 | 1.03 | NaN | 0.73 |
| F8VSA6 | NEDD8 | NEDD8 | 2 | 46 | 0 | 18.88 | 0.79 | 0.91 | 0.90 | NaN | 0.83 | 0.92 | NaN | NaN | 0.75 |
| E9PL71 | | EEF1D | 1 | 54 | 0 | 136.5 | NaN | NaN | 1.49 | 1.28 | 1.21 | NaN | NaN | 1.31 | 1.40 |
| E9PLK3 | Puromycin-sensitive aminopeptidase | NPEPPS | 27 | 36.7 | 0 | 168.3 | 1.08 | 1.09 | 1.12 | 1.11 | 1.08 | 1.14 | 1.03 | 1.00 | 1.04 |
| E9PLL6 | 60S ribosomal protein L27a | RPL27A | 4 | 42.6 | 0 | 18.76 | 0.75 | 1.10 | 0.72 | 0.76 | 1.12 | 0.84 | 1.02 | 1.02 | 0.75 |
| I3L3B4 | Vitamin K epoxide reductase complex subunit 1 | VKORC1 | 1 | 12.5 | 0 | 5.325 | NaN | NaN | NaN | 1.50 | NaN | NaN | 2.30 | 1.48 | NaN |
| K4DIA7 | Tetraspanin;CD151 antigen | CD151 | 4 | 15.7 | 0 | 9.393 | 0.94 | 1.17 | 1.04 | 1.32 | 1.20 | 1.00 | 1.21 | 1.20 | 1.11 |
| E9PR30 | 40S ribosomal protein S30 | FAU | 2 | 11.2 | 0 | 3.659 | 1.11 | 1.20 | 1.20 | 1.39 | 1.23 | 1.39 | 1.40 | 1.42 | 1.79 |
| E9PS97 | Alpha-parvin | PARVA | 4 | 27.8 | 0 | 17.42 | 1.08 | 1.39 | 1.15 | NaN | NaN | 1.07 | 0.64 | NaN | 1.30 |
| F2Z2E2 | Ras GTPase-activating-like protein IQGAP3 | IQGAP3 | 5 | 6.5 | 1E-03 | 2.284 | NaN | 1.10 | 0.58 | NaN | NaN | NaN | NaN | NaN | NaN |
| F2Z2Y8 | Toll-interacting protein | TOLLIP | 2 | 12.7 | 0 | 5.787 | 0.62 | 0.71 | NaN | NaN | NaN | 0.98 | NaN | NaN | NaN |
| F5GWX2 | Heme-binding protein 1 | HEBP1 | 5 | 62.4 | 0 | 78.33 | 1.17 | 0.93 | NaN | 1.19 | 1.06 | 1.18 | 0.85 | 0.67 | 1.01 |
| F5H5V4 | 26S proteasome non-ATPase regulatory subunit 9 | PSMD9 | 5 | 39.9 | 0 | 17.75 | NaN | 0.84 | NaN | NaN | 0.84 | NaN | 0.92 | 0.80 | NaN |
| H0YLY7 | Calcineurin B homologous protein 1 | CHP1 | 1 | 17.6 | 0.002 | 1.971 | NaN | NaN | 1.27 | 1.63 | NaN | NaN | 1.06 | 1.36 | NaN |

| | | | | | | | | | | | | | | |
|---|---|---|---|---|---|---|---|---|---|---|---|---|---|---|
| F5GX77 | Multifunctional methyltransferase subunit TRM112-like protein | TRMT112 | 3 | 38.7 | 0 | 3.864 | 0.97 | 1.49 | NaN | 0.97 | NaN | 1.48 | NaN | NaN | 1.61 |
| F5GXJ9 | CD166 antigen | ALCAM | 15 | 45.3 | 0 | 283.8 | 1.06 | 1.01 | 1.00 | 1.33 | 1.19 | 1.15 | 0.90 | 1.02 | 1.23 |
| F5GXX5 | Dolichyl-diphosphooligosaccharide--protein glycosyltransferase | DAD1 | 3 | 37.6 | 0 | 13.26 | NaN | 1.29 | NaN | 0.79 | 1.06 | 0.96 | 0.90 | 0.97 | NaN |
| H0YG54 | Oligoribonuclease, mitochondrial | REXO2 | 8 | 45.7 | 0 | 10.66 | 1.09 | 1.19 | 1.05 | 0.95 | 0.98 | 0.87 | 1.17 | 1.28 | 1.11 |
| F5GZS6 | 4F2 cell-surface antigen heavy chain | SLC3A2 | 17 | 34.2 | 0 | 323.3 | 1.12 | 1.07 | 1.10 | 0.94 | 1.06 | 0.99 | 1.13 | 1.10 | 1.12 |
| F5H157 | Ras-related protein Rab-35 | RAB35 | 1 | 5.9 | 0 | 208.8 | 1.40 | 1.22 | 1.01 | 0.98 | 1.39 | 1.33 | NaN | 1.01 | 1.07 |
| F5H2U8 | High mobility group protein HMGI-C | HMGA2 | 2 | 36.4 | 0 | 18.51 | NaN | NaN | 0.72 | 1.22 | NaN | NaN | 0.71 | 1.02 | NaN |
| F5H365 | Protein transport protein Sec23A | SEC23A | 7 | 18.9 | 0 | 26.35 | 0.74 | 0.67 | 0.83 | 0.93 | 2.07 | 0.85 | 0.90 | 0.93 | 0.97 |
| F5H442 | Tumor susceptibility gene 101 protein | TSG101 | 4 | 15.6 | 0 | 6.658 | NaN | NaN | 1.00 | 0.86 | NaN | 1.07 | NaN | NaN | NaN |
| F8VY35 | Nucleosome assembly protein 1-like 1 | NAP1L1 | 3 | 75.4 | 0 | 323.3 | 1.09 | 1.05 | 1.03 | 1.08 | 1.04 | 1.02 | 1.13 | 1.09 | 1.07 |
| F5H5D3 | Tubulin alpha-1C chain | TUBA1C | 4 | 69.7 | 0 | 323.3 | 0.53 | 0.56 | 0.53 | 0.56 | 0.58 | 0.59 | 0.96 | 0.89 | 0.94 |
| F5H6E2 | Unconventional myosin-Ic | MYO1C | 20 | 25.1 | 0 | 85.7 | 0.84 | 0.79 | 0.97 | 0.92 | 1.18 | 1.00 | 1.06 | 0.92 | 1.02 |
| F5H7F6 | Microsomal glutathione S-transferase 1 | MGST1 | 2 | 18.2 | 0 | 11.02 | NaN | NaN | NaN | NaN | 0.94 | 0.64 | NaN | NaN | NaN |
| H0Y3C5 | Non-specific protein-tyrosine kinase;Tyrosine-protein kinase Lyn;Tyrosine-protein kinase HCK;Proto-oncogene tyrosine-protein kinase Src;Tyrosine-protein kinase Fyn;Tyrosine-protein kinase Yes | HCK | 1 | 1.5 | 0 | 5.689 | 0.89 | 0.78 | NaN | 0.84 | NaN | 0.66 | NaN | 0.76 | 0.89 |
| F6TLX2 | Glyoxalase domain-containing protein 4 | GLOD4 | 11 | 26.5 | 0 | 25.02 | 1.02 | 1.05 | 1.00 | 0.93 | 1.14 | 1.08 | 0.90 | 0.92 | 0.91 |
| F6TR53 | HCLS1-binding protein 3 | HS1BP3 | 2 | 11.7 | 0.008 | 1.561 | 1.09 | NaN | 1.07 | NaN | NaN | 0.92 | NaN | NaN | NaN |
| F8VNT9 | Tetraspanin;CD63 antigen | CD63 | 1 | 6 | 0 | 4.749 | 0.83 | 0.96 | 0.93 | 2.00 | 1.62 | 1.18 | 0.98 | 1.19 | 0.95 |
| F8VPD4 | CAD protein;Glutamine-dependent carbamoyl-phosphate synthase;Aspartate carbamoyltransferase;Dihydroorotase | CAD | 32 | 20.8 | 0 | 242.8 | 0.79 | 0.76 | 0.76 | 0.74 | 0.74 | 0.77 | 1.02 | 1.02 | 0.96 |
| F8VQE1 | LIM domain and actin-binding protein 1 | LIMA1 | 14 | 32.1 | 0 | 82.95 | 0.97 | 0.84 | 0.75 | 0.74 | 0.90 | 1.09 | 0.69 | 0.64 | 0.88 |
| F8VVA7 | Coatomer subunit zeta-1 | COPZ1 | 6 | 31.3 | 0 | 26.73 | 0.99 | NaN | 1.08 | 1.08 | 1.32 | 1.11 | 0.86 | NaN | 1.00 |
| F8VVL1 | Density-regulated protein | DENR | 5 | 36.2 | 0 | 156.7 | NaN | NaN | 0.84 | 1.48 | NaN | NaN | 1.33 | 1.25 | 0.89 |
| F8VVM2 | | SLC25A3 | 1 | 38.9 | 0 | 231.3 | 0.86 | 0.91 | 1.06 | 1.07 | 1.06 | 1.05 | 0.77 | 1.04 | 0.83 |
| F8VXC8 | SWI/SNF complex subunit SMARCC2;SWI/SNF complex subunit SMARCC1 | SMARCC2 | 4 | 4.6 | 0 | 12.4 | 1.06 | 1.52 | 0.99 | 1.30 | 1.00 | NaN | NaN | 0.61 | 0.66 |
| F8VXU5 | Vacuolar protein sorting-associated protein 29 | VPS29 | 3 | 15.9 | 0 | 149 | 1.08 | 1.19 | 0.93 | 1.18 | 0.85 | 1.30 | 1.15 | NaN | 1.16 |

| | | | | | | | | | | | | | | | |
|---|---|---|---|---|---|---|---|---|---|---|---|---|---|---|---|
| F8VYE8 | Serine/threonine-protein phosphatase;Serine/threonine-protein phosphatase PP1-gamma catalytic subunit | PPP1CC | 3 | 70.4 | 0 | 45.99 | 1.20 | 1.14 | 1.14 | NaN | 1.62 | 1.38 | NaN | NaN | NaN |
| F8VZQ9 | SAP domain-containing ribonucleoprotein | SARNP | 4 | 17.4 | 0 | 3.912 | 0.92 | 0.91 | 0.91 | NaN | NaN | NaN | NaN | NaN | 0.86 |
| F8W031 | Protein canopy homolog 2 | CNPY2 | 3 | 19 | 0 | 10.07 | 1.26 | 1.21 | 1.22 | 1.13 | NaN | 0.99 | NaN | 1.03 | 0.84 |
| F8W1A4 | Adenylate kinase 2, mitochondrial;Adenylate kinase 2, mitochondrial;Adenylate kinase 2, mitochondrial, N-terminally processed | AK2 | 9 | 42.2 | 0 | 12.19 | 0.89 | 1.07 | 1.56 | NaN | 1.72 | 1.52 | NaN | NaN | 0.77 |
| F8W6G1 | Nuclear receptor-binding protein | NRBP1 | 5 | 12.3 | 0 | 18.78 | 0.71 | 0.94 | 0.79 | 1.08 | 0.74 | 1.12 | NaN | NaN | NaN |
| F8W6I7 | Heterogeneous nuclear ribonucleoprotein A1;Heterogeneous nuclear ribonucleoprotein A1, N-terminally processed;Heterogeneous | HNRNPA1 | 9 | 58.6 | 0 | 323.3 | 0.88 | 0.85 | 0.92 | 0.88 | 0.90 | 0.92 | 0.95 | 0.91 | 0.94 |
| F8W726 | Ubiquitin-associated protein 2- | UBAP2L | 12 | 17.4 | 0 | 230.3 | 1.26 | 1.07 | 1.27 | 0.85 | 1.00 | 1.27 | 1.11 | 0.94 | 1.02 |
| F8W7U8 | Double-strand break repair protein MRE11A | MRE11A | 4 | 8.1 | 0 | 5.058 | 0.82 | 1.35 | NaN | 0.98 | NaN | 0.88 | NaN | 0.89 | NaN |
| F8W930 | Insulin-like growth factor 2 mRNA-binding protein 2 | IGF2BP2 | 9 | 22 | 0 | 63.12 | 1.45 | 1.16 | 1.26 | 0.54 | 0.78 | 0.63 | 0.85 | NaN | 1.69 |
| F8WCF6 | Actin-related protein 2/3 complex subunit 4 | ARPC4-TT | 8 | 48.6 | 0 | 13.86 | 0.95 | 0.88 | 1.20 | 0.92 | 1.04 | 0.92 | 1.01 | 0.86 | 1.07 |
| G3V0I5 | NADH dehydrogenase [ubiquinone] flavoprotein 1, mitochondrial | NDUFV1 | 2 | 8.1 | 0 | 4.215 | 1.04 | NaN | 0.82 | NaN | NaN | NaN | NaN | NaN | 1.81 |
| G3V1D3 | Dipeptidyl peptidase 3 | DPP3 | 13 | 27.6 | 0 | 147.3 | 0.72 | 0.88 | 0.86 | NaN | 0.76 | 0.89 | NaN | 1.08 | 0.91 |
| G3V1C3 | Apoptosis inhibitor 5 | API5 | 9 | 24.7 | 0 | 231.8 | 0.93 | 0.93 | 0.99 | 1.26 | 1.28 | 0.80 | 1.33 | 1.46 | 0.91 |
| G3V1D4 | Protein lin-7 homolog C | LIN7C | 5 | 24.9 | 0 | 3.92 | NaN | 0.80 | 0.59 | NaN | NaN | NaN | NaN | NaN | NaN |
| G3V1U3 | Methionine aminopeptidase 2 | METAP2 | 7 | 32.9 | 0 | 140.6 | 0.89 | 0.62 | NaN | NaN | NaN | 0.79 | 1.20 | 1.12 | 0.85 |
| G3V2U7 | Acylphosphatase;Acylphosphatase-1 | ACYP1 | 2 | 17.8 | 0 | 14.98 | NaN | 1.33 | NaN | 1.08 | 1.13 | 1.34 | 1.08 | 0.95 | NaN |
| G3V4P8 | Glia maturation factor beta | GMFB | 5 | 46 | 0 | 168.3 | 1.09 | 1.12 | 1.11 | 0.93 | 0.98 | 1.02 | 1.02 | 0.83 | 1.13 |
| G3V5Z7 | Proteasome subunit alpha type;Proteasome subunit alpha type-6 | PSMA6 | 12 | 49.6 | 0 | 122.2 | 1.00 | 0.82 | 0.99 | 0.98 | 1.02 | 0.97 | 0.99 | 0.94 | 0.81 |
| G5EA06 | 28S ribosomal protein S27, mitochondrial | MRPS27 | 2 | 7.3 | 0 | 2.895 | 0.89 | NaN | 1.15 | NaN | NaN | NaN | NaN | NaN | 0.66 |
| G5EA30 | CUGBP Elav-like family member | CELF1 | 4 | 10.3 | 0.001 | 2.655 | 0.87 | 0.57 | NaN | NaN | NaN | 0.80 | NaN | NaN | NaN |
| G8JLA2 | | MYL6 | 1 | 69.7 | 0 | 323.3 | 0.98 | 0.97 | 0.96 | 0.95 | 1.01 | 0.99 | 1.09 | 1.01 | 1.04 |
| G8JLD5 | Dynamin-1-like protein | DNM1L | 14 | 28.1 | 0 | 88.27 | 1.19 | 0.98 | 1.21 | 0.99 | 1.22 | 1.11 | 0.80 | 0.82 | 1.02 |
| H0Y2V6 | Centrosomal protein of 170 kDa | CEP170 | 5 | 4.5 | 0 | 10.16 | NaN | NaN | NaN | NaN | NaN | 0.86 | 0.95 | 1.48 | NaN |
| H0Y2Y8 | Zyxin | ZYX | 11 | 30.6 | 0 | 152.9 | 1.72 | 1.56 | 1.45 | 1.37 | 1.37 | 1.38 | 1.32 | 1.37 | 1.46 |

| | | | | | | | | | | | | | | | |
|---|---|---|---|---|---|---|---|---|---|---|---|---|---|---|---|
| Q5QPK2 | Dolichol-phosphate mannosyltransferase subunit 1 | DPM1 | 6 | 23.7 | 0 | 10.71 | 1.19 | 0.94 | 1.30 | 1.34 | 0.95 | 0.99 | NaN | 1.68 | 1.29 |
| H0Y4E0 | Ubiquitin carboxyl-terminal hydrolase;Ubiquitin carboxyl-terminal hydrolase isozyme L5 | UCHL5 | 2 | 20.8 | 0 | 8.369 | 0.93 | 0.87 | 1.11 | 0.76 | 0.85 | 1.13 | NaN | 0.99 | NaN |
| H0Y5B4 | 60S ribosomal protein L36a;60S ribosomal protein L36a-like | RPL36A | 5 | 35.7 | 0 | 17.39 | 0.99 | 1.68 | 1.20 | 1.00 | NaN | 1.28 | 1.17 | 1.11 | 1.14 |
| P0DP25 | | CALM2 | 11 | 60.4 | 0 | 323.3 | 1.01 | 0.97 | 0.98 | 0.99 | 0.96 | 0.94 | 1.01 | 1.02 | 1.01 |
| H0Y8E6 | DNA replication licensing factor MCM2 | MCM2 | 11 | 18.5 | 0 | 29 | 0.72 | 0.76 | NaN | 0.98 | 0.91 | 1.00 | 0.66 | 0.89 | 0.61 |
| H0Y8Z9 | Acyl-CoA dehydrogenase family member 9, mitochondrial | ACAD9 | 2 | 7 | 0.001 | 2.755 | 0.84 | 0.72 | 0.98 | NaN | NaN | 0.79 | NaN | NaN | 0.90 |
| H0Y987 | Phosphoacetylglucosamine mutase | PGM3 | 15 | 33.6 | 0 | 121 | 0.99 | 0.93 | 0.97 | 1.01 | 1.01 | 0.89 | 1.06 | 1.15 | 1.06 |
| H0YBL1 | Inositol monophosphatase 1 | IMPA1 | 4 | 20.8 | 0 | 6.095 | 0.89 | 0.69 | 1.31 | 1.02 | 0.80 | NaN | NaN | 1.12 | 0.81 |
| H0YC42 | Tumor protein D52 | TPD52 | 6 | 34.9 | 0 | 36.81 | 1.28 | 1.30 | 1.21 | 1.11 | 1.13 | NaN | 1.18 | 1.24 | NaN |
| H0YD13 | CD44 antigen | CD44 | 8 | 47.6 | 0 | 322.3 | 1.22 | 1.24 | 1.18 | 1.06 | 1.04 | 1.08 | 1.21 | 0.95 | 0.97 |
| H0YDU8 | Serine/threonine-protein phosphatase;Serine/threonine-protein phosphatase 5 | PPP5C | 6 | 14.4 | 0 | 10.67 | 1.26 | NaN | 2.19 | 0.71 | 0.97 | 0.77 | 0.96 | 0.70 | 1.09 |
| H0YFD6 | Trifunctional enzyme subunit alpha, mitochondrial;Long-chain enoyl-CoA hydratase;Long chain 3-hydroxyacyl-CoA dehydrogenase | HADHA | 27 | 50.5 | 0 | 323.3 | 1.04 | 0.76 | 1.04 | 1.07 | 0.95 | 0.85 | 0.87 | 0.87 | 0.91 |
| H0YHA7 | 60S ribosomal protein L18 | RPL18 | 7 | 46.7 | 0 | 161.9 | 0.98 | 0.99 | 1.05 | 0.84 | 0.92 | 1.02 | 0.91 | 0.96 | 1.05 |
| H0YMV8 | 40S ribosomal protein S27;40S ribosomal protein S27-like | RPS27L | 1 | 32 | 1E-03 | 2.141 | NaN | NaN | NaN | 0.67 | NaN | NaN | 1.16 | 1.03 | NaN |
| H0YNW5 | Deoxyuridine 5-triphosphate nucleotidohydrolase, | DUT | 5 | 37.1 | 0 | 26.77 | 1.09 | 1.25 | 1.20 | 0.88 | 0.87 | 1.06 | 0.71 | NaN | 0.87 |
| H3BLV9 | SRSF protein kinase 1 | SRPK1 | 5 | 12.1 | 0 | 26.41 | 0.88 | 0.94 | 1.10 | 0.66 | 0.79 | 0.94 | NaN | 0.79 | 0.66 |
| H3BM14 | NEDD8 ultimate buster 1 | NUB1 | 2 | 3.7 | 0 | 3.501 | 0.77 | 0.86 | 0.89 | NaN | 0.47 | NaN | NaN | NaN | 1.67 |
| H3BN50 | Na(+)/H(+) exchange regulatory cofactor NHE-RF2 | SLC9A3R2 | 4 | 23.3 | 0 | 2.892 | 1.08 | 0.77 | 1.20 | NaN | 0.66 | NaN | NaN | NaN | 0.75 |
| H3BN98 | | | 2 | 23.6 | 0.002 | 1.984 | 0.89 | 1.35 | 1.04 | 1.17 | 1.07 | NaN | 0.94 | 0.74 | 1.04 |
| H3BNC9 | 40S ribosomal protein S17 | RPS17 | 6 | 14 | 0 | 323.3 | 0.77 | 0.84 | 0.76 | 0.82 | 0.89 | 0.81 | 0.73 | 0.83 | 0.77 |
| H3BPE1 | Microtubule-actin cross-linking factor 1, isoforms 1/2/3/5 | MACF1 | 11 | 2.6 | 0 | 9.739 | 0.77 | NaN | 0.70 | 0.83 | 1.15 | 0.86 | 1.22 | 1.11 | 1.31 |
| H3BPK3 | Hydroxyacylglutathione hydrolase, mitochondrial | HAGH | 3 | 14.2 | 0.001 | 2.585 | NaN | 1.23 | 1.06 | NaN | 1.06 | 0.86 | NaN | NaN | 0.93 |
| H3BUU8 | | VAC14 | 1 | 14.4 | 0 | 5.888 | 0.66 | 0.66 | 0.67 | NaN | NaN | 0.72 | NaN | NaN | 1.50 |
| H7BYN3 | Transcription factor A, mitochondrial | TFAM | 2 | 10 | 0.009 | 1.449 | NaN | NaN | 1.04 | 0.87 | 0.94 | NaN | NaN | NaN | NaN |
| H7BYY1 | | TPM1 | 7 | 65.3 | 0 | 86.32 | 1.23 | 1.17 | 1.37 | 0.98 | 1.15 | 0.97 | 1.88 | 1.92 | 1.93 |
| H7BZI1 | Nucleobindin-1 | NUCB1 | 4 | 17.3 | 0 | 7.564 | 1.41 | 0.99 | 1.37 | NaN | NaN | 0.94 | NaN | 1.24 | NaN |
| H7BZJ3 | | PDIA3 | 1 | 53.7 | 0 | 127.7 | 1.32 | 1.28 | 1.72 | 1.18 | 1.16 | 1.24 | 1.20 | 1.25 | 1.34 |
| H7C0E5 | Zinc finger protein ZPR1 | ZPR1 | 4 | 12.2 | 0 | 6.483 | 1.26 | 0.96 | 1.17 | 1.12 | 1.38 | 0.98 | 1.00 | NaN | 0.99 |

| | | | | | | | | | | | | | | | |
|---|---|---|---|---|---|---|---|---|---|---|---|---|---|---|---|
| H7C2Q8 | Probable rRNA-processing protein EBP2 | EBNA1BP2 | 4 | 15 | 0 | 3.79 | 1.91 | NaN | NaN | 1.43 | 0.83 | 1.05 | NaN | NaN | 1.43 |
| H7C4H2 | Signal recognition particle receptor subunit beta | SRPRB | 1 | 8.1 | 0 | 4.804 | 1.24 | 0.87 | 0.93 | NaN | 1.01 | 1.03 | NaN | NaN | 1.17 |
| H7C4T5 | Muscleblind-like protein 1;Muscleblind-like protein 2 | MBNL1 | 5 | 20.7 | 0 | 3.455 | NaN | 0.73 | NaN | NaN | 1.24 | 1.62 | NaN | NaN | 0.59 |
| H7C5G1 | Isoamyl acetate-hydrolyzing esterase 1 homolog | IAH1 | 2 | 17.1 | 0 | 48.11 | 2.42 | 2.32 | NaN | 1.80 | NaN | NaN | NaN | 1.40 | 2.20 |
| I1E4Y6 | PERQ amino acid-rich with GYF domain-containing protein 2 | GIGYF2 | 3 | 3.6 | 0 | 2.904 | NaN | NaN | NaN | 1.38 | 1.41 | NaN | NaN | NaN | NaN |
| I3L0H8 | ATP-dependent RNA helicase DDX19A;ATP-dependent RNA helicase DDX19B | DDX19A | 2 | 5.8 | 0 | 7.61 | NaN | NaN | NaN | 0.80 | NaN | NaN | 1.50 | 0.85 | NaN |
| I3L0N3 | Vesicle-fusing ATPase | NSF | 6 | 8.5 | 0 | 5.778 | 0.60 | 0.72 | 0.90 | 0.90 | NaN | NaN | NaN | NaN | NaN |
| I3L1P8 | Mitochondrial 2-oxoglutarate/malate carrier | SLC25A11 | 3 | 11.8 | 0 | 153.1 | 0.65 | 1.09 | NaN | NaN | 1.55 | NaN | NaN | NaN | 0.81 |
| I3L294 | Monoacylglycerol lipase ABHD12 | ABHD12 | 2 | 19.5 | 0.001 | 2.084 | 0.68 | NaN | 0.95 | 1.01 | NaN | 1.16 | NaN | 1.07 | 1.01 |
| I3L397 | Eukaryotic translation initiation factor 5A;Eukaryotic translation initiation factor 5A-1;Eukaryotic translation initiation factor 5A-1-like;Eukaryotic translation initiation factor 5A-2 | EIF5A | 14 | 76.7 | 0 | 296.5 | 0.98 | 1.01 | 0.92 | 1.06 | 0.98 | 0.97 | 0.86 | 0.95 | 0.94 |
| J3KMZ9 | Low-density lipoprotein receptor | LDLR | 13 | 20.1 | 0 | 108.5 | 1.29 | 1.03 | 0.94 | 1.53 | 1.76 | 1.73 | 0.95 | 1.32 | 0.94 |
| J3KN16 | Proteasome-associated protein ECM29 homolog | KIAA0368 | 6 | 4.3 | 0 | 5.975 | NaN | NaN | NaN | 0.93 | NaN | 1.19 | 1.04 | 1.20 | 0.72 |
| J3KN66 | | TOR1AIP1 | 1 | 15.9 | 0 | 30.88 | 1.35 | 1.30 | 1.07 | 0.98 | 0.81 | 1.43 | NaN | 1.35 | 1.36 |
| J3KN67 | | TPM3 | 0 | 62.8 | 0 | 15.42 | 1.03 | 0.95 | 1.00 | NaN | 1.65 | 0.76 | NaN | NaN | 1.13 |
| J3QL05 | Serine/arginine-rich splicing factor 2 | SRSF2 | 5 | 45.4 | 0 | 93.49 | 0.85 | 1.80 | 1.23 | 1.02 | 0.96 | 0.95 | 0.51 | 0.46 | 0.44 |
| J3KPX7 | Prohibitin-2 | PHB2 | 16 | 58.1 | 0 | 105.3 | 1.00 | 0.91 | 0.94 | 1.03 | 0.95 | 0.95 | 0.78 | 1.21 | 0.77 |
| J3KQ18 | D-dopachrome decarboxylase;D-dopachrome decarboxylase-like protein | DDT | 5 | 40.9 | 0 | 22.72 | 1.10 | 1.10 | 0.99 | 0.90 | 1.14 | 1.04 | NaN | 0.91 | 0.97 |
| J3KQ32 | Obg-like ATPase 1 | OLA1 | 16 | 55.8 | 0 | 102.5 | 0.83 | 0.91 | 0.89 | 1.00 | 1.10 | 0.89 | 0.81 | 1.06 | 0.90 |
| J3KQ48 | Peptidyl-tRNA hydrolase 2, mitochondrial | PTRH2 | 3 | 26.7 | 0 | 8.885 | 1.46 | 1.60 | 0.80 | 0.86 | 0.93 | 1.44 | NaN | NaN | 0.86 |
| J3KR44 | Ubiquitin thioesterase OTUB1 | OTUB1 | 11 | 55.1 | 0 | 119.5 | 0.84 | 0.97 | 0.80 | 1.20 | 0.81 | 0.74 | 1.24 | 1.05 | 0.80 |
| J3KR97 | Tubulin-specific chaperone D | TBCD | 3 | 4.2 | 0 | 9.811 | NaN | NaN | 0.40 | 0.89 | 0.71 | 0.70 | NaN | NaN | 0.77 |
| J3QR09 | Ribosomal protein L19;60S ribosomal protein L19 | RPL19 | 8 | 31.6 | 0 | 44.42 | 1.05 | 1.05 | 0.99 | 1.07 | 1.10 | 1.09 | 1.16 | 1.02 | 1.10 |
| J3KTI2 | Migration and invasion enhancer | MIEN1 | 2 | 29.3 | 0 | 5.034 | 0.84 | 0.78 | 0.91 | NaN | NaN | 1.18 | NaN | NaN | NaN |
| J3KTL2 | Serine/arginine-rich splicing factor 1 | SRSF1 | 13 | 46.2 | 0 | 107.5 | 1.04 | 0.95 | 1.11 | 0.97 | 0.95 | 0.92 | 1.04 | 1.01 | 1.01 |

| | | | | | | | | | | | | | | | |
|---|---|---|---|---|---|---|---|---|---|---|---|---|---|---|---|
| J3QLE5 | Small nuclear ribonucleoprotein-associated protein N;Small nuclear ribonucleoprotein-associated proteins B and B | SNRPN | 7 | 38.5 | 0 | 15.84 | 0.84 | 0.85 | 0.86 | 0.86 | 0.89 | 0.85 | 1.02 | 0.99 | 0.96 |
| J3QRS3 | Myosin regulatory light chain 12A;Myosin regulatory light chain 12B | MYL12A | 4 | 64.4 | 0 | 323.3 | 0.90 | 0.96 | 0.86 | 0.99 | 1.05 | 0.98 | 0.95 | 0.94 | 0.79 |
| K4DI93 | Cullin-4B | CUL4B | 6 | 13.7 | 0 | 61.68 | 0.96 | 1.07 | 1.09 | 1.04 | 1.16 | 1.10 | 0.73 | 0.75 | 0.92 |
| K7EM02 | Katanin p60 ATPase-containing subunit A-like 2 | KATNAL2 | 1 | 9.4 | 0 | 4.338 | 0.94 | NaN | 0.90 | 1.01 | 1.01 | NaN | 0.90 | 0.84 | NaN |
| K7EJE8 | Lon protease homolog, mitochondrial | LONP1 | 11 | 21.4 | 0 | 170.2 | 0.90 | 0.86 | 1.00 | NaN | 0.83 | 0.97 | 0.63 | NaN | 1.05 |
| K7EK07 | Histone H3;Histone H3.3 | H3F3B | 1 | 40.9 | 0.001 | 2.751 | 0.33 | 0.52 | NaN | NaN | NaN | NaN | NaN | NaN | NaN |
| K7EQ02 | DAZ-associated protein 1 | DAZAP1 | 5 | 22.6 | 0 | 25.6 | 1.34 | 1.14 | 1.34 | 1.93 | 1.15 | 1.11 | NaN | 1.37 | 1.05 |
| K7EKL3 | Granulins;Acrogranin;Paragranulin;Granulin-1;Granulin-2;Granulin-3;Granulin-4;Granulin-5;Granulin-6;Granulin-7 | GRN | 3 | 10.8 | 0 | 69.04 | NaN | NaN | NaN | NaN | 0.57 | 0.66 | NaN | NaN | 2.13 |
| K7EL20 | Eukaryotic translation initiation factor 3 subunit G | EIF3G | 1 | 43.5 | 0 | 182.9 | 1.07 | 1.10 | 1.07 | 0.94 | 1.16 | 0.86 | 1.01 | 1.02 | 0.88 |
| K7ELD9 | Synaptogyrin-2 | SYNGR2 | 2 | 30.8 | 0.001 | 2.671 | NaN | NaN | NaN | 1.03 | 0.84 | NaN | NaN | NaN | NaN |
| K7ELL7 | Glucosidase 2 subunit beta | PRKCSH | 23 | 48 | 0 | 323.3 | 1.13 | 1.03 | 1.02 | 1.11 | 1.12 | 1.15 | 0.99 | 0.97 | 0.96 |
| K7ELP0 | | TPM4 | 1 | 15.9 | 0 | 24.89 | 0.88 | 0.97 | 0.74 | NaN | NaN | 0.82 | 1.60 | 1.49 | 1.33 |
| K7ELS0 | Uncharacterized protein | C19orf43 | 1 | 10.6 | 0 | 3.023 | 0.98 | 1.26 | NaN | 0.98 | 1.85 | 2.93 | NaN | 0.91 | 1.14 |
| K7EM18 | Eukaryotic translation initiation factor 1;Eukaryotic translation initiation factor 1b | EIF1 | 6 | 62 | 0 | 49.15 | 0.86 | 1.03 | 1.09 | 1.72 | 1.01 | 1.07 | 1.01 | NaN | NaN |
| K7ENT6 | | TPM4 | 2 | 63.6 | 0 | 40.36 | 1.27 | 1.27 | 1.18 | 1.21 | 0.94 | 1.18 | 1.44 | 1.63 | 1.49 |
| Q08E86 | Protein KIAA0100 | KIAA0100 | 1 | 0.4 | 0.009 | 1.414 | NaN | NaN | NaN | NaN | 1.03 | 0.68 | NaN | NaN | 1.33 |
| K7EQX8 | | MXRA7 | 1 | 73.3 | 0 | 24.03 | 1.16 | 1.84 | NaN | 2.12 | NaN | NaN | NaN | NaN | 1.50 |
| K7ER90 | | EIF3G | 1 | 48.9 | 0 | 33.35 | 1.18 | 1.27 | 1.25 | 1.13 | 1.06 | 1.07 | 0.98 | 0.98 | 0.99 |
| K7ER96 | Thioredoxin-like protein 1 | TXNL1 | 4 | 19.2 | 0 | 9.479 | 0.75 | 0.65 | 0.86 | 0.87 | 1.52 | 0.89 | 1.06 | 0.84 | 1.11 |
| K7ERF1 | Eukaryotic translation initiation factor 3 subunit K | EIF3K | 7 | 42.2 | 0 | 24.88 | 1.18 | 0.96 | 1.27 | 1.13 | 1.13 | 0.97 | NaN | 0.59 | NaN |
| K7ERV3 | Thymidine kinase;Thymidine kinase, cytosolic | TK1 | 2 | 11.2 | 0.007 | 1.635 | NaN | NaN | NaN | 0.86 | 1.20 | NaN | NaN | NaN | NaN |
| M0QXB4 | Coatomer subunit epsilon | COPE | 8 | 32.9 | 0 | 21.94 | 0.72 | 0.73 | 0.98 | 0.90 | 1.10 | 0.92 | 0.87 | 0.75 | 0.91 |
| M0QXN5 | Nuclear pore glycoprotein p62 | NUP62 | 4 | 17.9 | 0 | 27.4 | NaN | NaN | 0.87 | 0.90 | 0.98 | NaN | 0.87 | NaN | NaN |
| M0QYT0 | | | 0 | 42.7 | 0 | 5.035 | 1.21 | 1.10 | 1.20 | 0.96 | 1.53 | 1.18 | 0.77 | 0.81 | 1.01 |
| M0R0N4 | AP-2 complex subunit sigma | AP2S1 | 2 | 11.1 | 0.003 | 1.883 | 0.75 | 0.85 | NaN | 0.73 | 1.00 | 0.95 | NaN | 0.77 | 1.04 |
| M0R0F0 | 40S ribosomal protein S5;40S ribosomal protein S5, N-terminally processed | RPS5 | 12 | 50 | 0 | 248.9 | 0.88 | 0.89 | 0.78 | 0.78 | 0.75 | 1.13 | 0.94 | 0.90 | 0.93 |
| M0R117 | 60S ribosomal protein L18a | RPL18A | 9 | 53.9 | 0 | 84.35 | 0.82 | 0.81 | 0.79 | 0.83 | 0.73 | 0.80 | 0.72 | 0.70 | 1.01 |

| | | | | | | | | | | | | | | | |
|---|---|---|---|---|---|---|---|---|---|---|---|---|---|---|---|
| M0R208 | ATP-dependent Clp protease proteolytic subunit;ATP-dependent Clp protease proteolytic subunit, mitochondrial | CLPP | 2 | 12.1 | 0.003 | 1.862 | 1.46 | 1.50 | NaN | NaN | 1.71 | NaN | NaN | NaN | NaN |
| O00116 | Alkyldihydroxyacetonephosphate synthase, peroxisomal | AGPS | 7 | 16.9 | 0 | 30.57 | 0.73 | 0.62 | 0.58 | NaN | NaN | 0.78 | 1.16 | 2.46 | 0.82 |
| O00148 | ATP-dependent RNA helicase DDX39A | DDX39A | 3 | 48.7 | 0 | 61.82 | 0.74 | 0.80 | 0.69 | NaN | NaN | 0.99 | 0.71 | NaN | 0.83 |
| O00151 | PDZ and LIM domain protein 1 | PDLIM1 | 18 | 82.7 | 0 | 323.3 | 0.87 | 0.91 | 0.91 | 0.93 | 0.99 | 0.96 | 0.86 | 0.97 | 1.06 |
| O00154 | Cytosolic acyl coenzyme A thioester hydrolase | ACOT7 | 5 | 15.5 | 0 | 23.3 | 1.28 | NaN | 1.11 | NaN | NaN | 1.18 | NaN | NaN | 1.29 |
| O00170 | AH receptor-interacting protein | AIP | 11 | 41.8 | 0 | 14.15 | 0.96 | 1.29 | 1.09 | 0.83 | 1.03 | 0.97 | 0.93 | 1.00 | 1.26 |
| O00203 | AP-3 complex subunit beta-1 | AP3B1 | 5 | 8 | 0 | 6.681 | 2.06 | NaN | NaN | 1.18 | NaN | NaN | 1.04 | 1.05 | NaN |
| O00231 | 26S proteasome non-ATPase regulatory subunit 11 | PSMD11 | 17 | 48.6 | 0 | 61.35 | 1.05 | 1.11 | 1.03 | 1.04 | 0.94 | 1.13 | 1.03 | 1.00 | 0.99 |
| O00232 | 26S proteasome non-ATPase regulatory subunit 12 | PSMD12 | 10 | 27.6 | 0 | 17.33 | 1.34 | 1.07 | 1.07 | 0.91 | 0.95 | 0.91 | NaN | 1.46 | 1.00 |
| O00273 | DNA fragmentation factor subunit alpha | DFFA | 1 | 5.1 | 0.002 | 2.021 | 1.17 | 1.19 | NaN | NaN | NaN | NaN | NaN | NaN | 0.95 |
| O00299 | Chloride intracellular channel protein 1 | CLIC1 | 17 | 87.1 | 0 | 323.3 | 1.00 | 0.93 | 1.01 | 1.02 | 1.00 | 0.99 | 0.96 | 0.93 | 0.97 |
| O00303 | Eukaryotic translation initiation factor 3 subunit F | EIF3F | 11 | 38.9 | 0 | 73.51 | 1.14 | 1.22 | 1.23 | 1.08 | 1.20 | 0.99 | 1.18 | 0.73 | 1.05 |
| O00410 | Importin-5 | IPO5 | 38 | 47.7 | 0 | 323.3 | 1.11 | 1.07 | 0.99 | 1.01 | 1.04 | 0.98 | 0.73 | 0.79 | 1.01 |
| O00422 | Histone deacetylase complex subunit SAP18 | SAP18 | 5 | 40.5 | 0 | 3.092 | 0.60 | NaN | 1.44 | NaN | 1.15 | 1.17 | 1.17 | NaN | 1.37 |
| O00483 | Cytochrome c oxidase subunit NDUFA4 | NDUFA4 | 3 | 37 | 0 | 6.352 | 1.13 | 0.92 | 0.98 | 1.08 | 1.24 | 1.14 | 1.15 | 1.17 | NaN |
| O00487 | 26S proteasome non-ATPase regulatory subunit 14 | PSMD14 | 6 | 34.8 | 0 | 89.9 | 0.86 | 0.88 | 1.11 | 0.92 | 0.98 | 1.09 | 0.89 | 0.79 | 1.20 |
| O00499 | Myc box-dependent-interacting protein 1 | BIN1 | 6 | 18.9 | 0 | 24.63 | 1.42 | 0.96 | 0.90 | NaN | 3.51 | 0.73 | NaN | 0.95 | 0.93 |
| O00505 | Importin subunit alpha-4 | KPNA3 | 6 | 23.6 | 0 | 62.26 | 0.82 | 0.79 | 0.48 | 1.01 | 0.91 | 0.86 | 1.26 | 1.06 | 0.89 |
| O00560 | Syntenin-1 | SDCBP | 2 | 13.8 | 0 | 18.49 | 0.82 | 0.87 | 0.78 | NaN | NaN | 0.85 | NaN | NaN | 0.81 |
| O00592 | Podocalyxin | PODXL | 7 | 17.4 | 0 | 99.48 | 2.17 | 2.51 | 2.14 | 1.15 | 1.18 | 1.06 | 1.95 | 1.98 | 2.85 |
| O00622 | Protein CYR61 | CYR61 | 21 | 68.2 | 0 | 234.2 | 2.49 | 2.33 | 2.61 | 1.27 | 1.34 | 1.20 | 1.39 | 1.38 | 1.25 |
| O00629 | Importin subunit alpha-3 | KPNA4 | 7 | 29.2 | 0 | 54.42 | 1.37 | 0.91 | 0.93 | 0.95 | 1.43 | 0.63 | NaN | 0.97 | 0.77 |
| O00743 | Serine/threonine-protein phosphatase 6 catalytic subunit;Serine/threonine-protein phosphatase 6 catalytic subunit, N-terminally processed | PPP6C | 3 | 11.1 | 0.008 | 1.48 | NaN | 0.48 | NaN | 0.69 | 0.95 | 0.45 | NaN | NaN | NaN |
| O00764 | Pyridoxal kinase | PDXK | 8 | 41.7 | 0 | 264.8 | 1.18 | 1.31 | 1.10 | 0.78 | 0.93 | 1.17 | 1.12 | NaN | 1.20 |
| O14493 | Claudin-4;Claudin-9;Claudin-6;Claudin-3 | CLDN4 | 2 | 12.9 | 0 | 15.07 | 1.52 | 0.79 | NaN | NaN | NaN | 0.98 | 1.39 | 1.45 | NaN |
| O14561 | Acyl carrier protein, mitochondrial;Acyl carrier protein | NDUFAB1 | 3 | 21.2 | 0 | 9.133 | 0.98 | 1.09 | 1.01 | 1.19 | 1.07 | 1.09 | 0.93 | 0.93 | 1.04 |

| | | | | | | | | | | | | | | |
|---|---|---|---|---|---|---|---|---|---|---|---|---|---|---|
| O14617 | AP-3 complex subunit delta-1 | AP3D1 | 6 | 9 | 0 | 21.65 | 0.79 | 0.66 | NaN | NaN | 0.52 | 1.00 | NaN | NaN | 0.58 |
| O14737 | Programmed cell death protein 5 | PDCD5 | 7 | 56 | 0 | 218.2 | 1.20 | 1.11 | 1.31 | 1.21 | 1.13 | 1.06 | 1.51 | 1.19 | 1.15 |
| O14744 | Protein arginine N-methyltransferase 5;Protein arginine N-methyltransferase 5, N-terminally processed | PRMT5 | 6 | 11.9 | 0 | 35.91 | 1.07 | 0.94 | 1.27 | NaN | 0.80 | 0.95 | NaN | 1.40 | 0.88 |
| O14745 | Na(+)/H(+) exchange regulatory cofactor NHE-RF1 | SLC9A3R1 | 7 | 28.5 | 0 | 35.57 | NaN | 0.79 | 1.08 | 1.13 | 0.67 | NaN | NaN | 0.48 | 0.78 |
| O14763 | Tumor necrosis factor receptor superfamily member 10B | TNFRSF10 | 1 | 3.9 | 0.009 | 1.457 | NaN | 1.85 | 1.21 | NaN | NaN | NaN | NaN | NaN | NaN |
| O14818 | Proteasome subunit alpha type-7 | PSMA7 | 14 | 66.5 | 0 | 323.3 | 0.98 | 1.10 | 0.97 | 0.94 | 1.00 | 0.99 | 0.92 | 0.92 | 0.97 |
| O14828 | Secretory carrier-associated membrane protein 3 | SCAMP3 | 3 | 12.4 | 0 | 6.226 | 0.89 | 1.43 | NaN | 2.16 | NaN | 1.41 | NaN | 1.43 | NaN |
| O14907 | Tax1-binding protein 3 | TAX1BP3 | 3 | 39.5 | 0 | 14.17 | 1.18 | 1.45 | NaN | NaN | NaN | NaN | NaN | NaN | NaN |
| O14929 | Histone acetyltransferase type B catalytic subunit | HAT1 | 3 | 12.9 | 0.001 | 2.618 | NaN | 0.64 | 0.84 | NaN | 0.94 | NaN | 1.35 | NaN | NaN |
| O14964 | Hepatocyte growth factor-regulated tyrosine kinase substrate | HGS | 7 | 10.7 | 0 | 6.788 | 0.96 | 1.11 | 0.86 | 0.98 | 0.86 | 0.95 | NaN | 1.14 | 1.27 |
| O14974 | Protein phosphatase 1 regulatory subunit 12A | PPP1R12A | 11 | 12.9 | 0 | 91.9 | NaN | 1.32 | NaN | NaN | 2.78 | 0.61 | NaN | NaN | 1.86 |
| O14980 | Exportin-1 | XPO1 | 26 | 31.6 | 0 | 173.3 | 0.95 | 0.96 | 0.95 | 0.93 | 1.04 | 0.93 | 0.88 | 1.00 | 0.94 |
| O15067 | Phosphoribosylformylglycinamidine synthase | PFAS | 6 | 7.2 | 0 | 9.764 | NaN | 0.89 | NaN | 0.72 | 0.85 | NaN | NaN | 0.84 | 1.14 |
| O15131 | Importin subunit alpha-6;Importin subunit alpha-5;Importin subunit alpha-5, N-terminally processed;Importin subunit alpha-7 | KPNA5 | 3 | 6 | 0 | 8.008 | NaN | 0.88 | 0.93 | 1.10 | 0.99 | 0.77 | 0.95 | 0.98 | 0.97 |
| O15143 | Actin-related protein 2/3 complex subunit 1B | ARPC1B | 4 | 55.1 | 0 | 319.7 | 0.87 | 1.08 | 0.91 | 0.95 | 0.91 | 0.96 | 0.84 | 0.98 | 0.90 |
| O15144 | Actin-related protein 2/3 complex subunit 2 | ARPC2 | 13 | 40.3 | 0 | 66.77 | 0.82 | 0.95 | 0.87 | 1.16 | 1.02 | 0.90 | 1.08 | 1.06 | 0.69 |
| O15145 | Actin-related protein 2/3 complex subunit 3 | ARPC3 | 8 | 42.1 | 0 | 33.33 | 0.97 | 0.95 | 0.99 | 1.13 | 1.00 | 0.97 | 0.96 | 1.12 | 1.06 |
| O15173 | Membrane-associated progesterone receptor component 2 | PGRMC2 | 5 | 29.6 | 0 | 35.45 | NaN | NaN | NaN | 0.86 | 0.91 | 0.84 | 1.02 | 2.17 | 1.26 |
| O15212 | Prefoldin subunit 6 | PFDN6 | 5 | 32.6 | 0 | 27.76 | 0.87 | NaN | 0.89 | 1.19 | NaN | NaN | 0.97 | 1.17 | NaN |
| O15231 | Zinc finger protein 185 | ZNF185 | 12 | 27.4 | 0 | 90.95 | 1.03 | 0.96 | NaN | 1.19 | 1.00 | 1.04 | 1.22 | 1.85 | 1.78 |
| Q5T8U5 | Surfeit locus protein 4 | SURF4 | 4 | 26.9 | 0 | 29.83 | 1.04 | 0.95 | 0.84 | 1.05 | 1.16 | 1.02 | 0.78 | 0.65 | 0.78 |
| O15355 | Protein phosphatase 1G | PPM1G | 13 | 33.3 | 0 | 234.5 | 1.08 | 1.11 | 1.28 | 0.98 | 1.14 | 1.00 | 0.95 | 1.03 | 0.99 |
| O15371 | Eukaryotic translation initiation factor 3 subunit D | EIF3D | 15 | 43.1 | 0 | 88.33 | 1.09 | 0.96 | 1.08 | 1.36 | 0.92 | 1.01 | 0.89 | 0.91 | 0.93 |
| O15400 | Syntaxin-7 | STX7 | 5 | 25.7 | 0 | 17.37 | 1.41 | NaN | NaN | NaN | 0.88 | 1.00 | 0.67 | 0.87 | NaN |
| O15427 | Monocarboxylate transporter 4 | SLC16A3 | 6 | 15.3 | 0 | 38.68 | 1.11 | 1.02 | 0.99 | 0.93 | 0.97 | 1.21 | 1.17 | 1.20 | 1.32 |

| | | | | | | | | | | | | | | | |
|---|---|---|---|---|---|---|---|---|---|---|---|---|---|---|---|
| O15460 | Prolyl 4-hydroxylase subunit alpha-2 | P4HA2 | 5 | 15.3 | 0 | 13.19 | NaN | 1.28 | NaN | NaN | NaN | NaN | 1.23 | NaN | 1.38 |
| O15498 | Synaptobrevin homolog YKT6 | YKT6 | 2 | 11.1 | 0.001 | 2.54 | 0.80 | NaN | 0.82 | NaN | 0.71 | 1.22 | NaN | NaN | NaN |
| O15511 | Actin-related protein 2/3 complex subunit 5 | ARPC5 | 3 | 40.4 | 0 | 60.31 | 1.17 | 1.05 | 1.09 | 0.90 | 0.96 | 1.01 | NaN | 0.96 | 1.14 |
| O43143 | Pre-mRNA-splicing factor ATP-dependent RNA helicase DHX15 | DHX15 | 14 | 20.4 | 0 | 33.13 | 0.99 | 0.70 | 0.64 | 0.97 | 1.26 | 0.95 | 0.78 | 0.61 | 0.94 |
| O43172 | U4/U6 small nuclear ribonucleoprotein Prp4 | PRPF4 | 3 | 7.1 | 0 | 7.048 | 1.12 | NaN | 1.21 | NaN | NaN | NaN | NaN | NaN | 1.18 |
| O43237 | Cytoplasmic dynein 1 light intermediate chain 2 | DYNC1LI2 | 6 | 30.1 | 0 | 24.86 | 1.50 | 1.15 | 0.67 | 0.73 | NaN | NaN | 0.71 | 0.44 | 0.80 |
| O43242 | 26S proteasome non-ATPase regulatory subunit 3 | PSMD3 | 13 | 28.1 | 0 | 259.4 | 1.16 | 1.27 | 1.14 | 1.00 | 1.03 | 1.07 | 0.95 | 1.01 | 0.95 |
| O43264 | Centromere/kinetochore protein zw10 homolog | ZW10 | 3 | 6.2 | 0 | 24.3 | 0.83 | 0.84 | 0.80 | NaN | 0.77 | NaN | NaN | NaN | 0.89 |
| O43291 | Kunitz-type protease inhibitor 2 | SPINT2 | 2 | 9.5 | 0.001 | 2.599 | 1.08 | 1.31 | 1.54 | NaN | NaN | NaN | NaN | NaN | 1.08 |
| O43294 | Transforming growth factor beta-1-induced transcript 1 protein | TGFB1I1 | 2 | 5.4 | 0 | 10.06 | NaN | 1.32 | 0.88 | NaN | NaN | 1.00 | NaN | NaN | 0.97 |
| O43390 | Heterogeneous nuclear ribonucleoprotein R | HNRNPR | 17 | 43.4 | 0 | 58.51 | 0.79 | 0.82 | 0.92 | 0.76 | 0.84 | 0.91 | 1.05 | 1.10 | 0.87 |
| O43399 | Tumor protein D54 | TPD52L2 | 12 | 68.9 | 0 | 323.3 | 1.18 | 1.06 | 1.11 | 1.09 | 1.37 | 1.02 | 0.98 | 1.04 | 0.97 |
| O43447 | Peptidyl-prolyl cis-trans isomerase H;Peptidyl-prolyl cis-trans isomerase | PPIH | 4 | 38.4 | 0 | 5.263 | 0.80 | NaN | NaN | NaN | 0.80 | 0.90 | NaN | NaN | NaN |
| O43493 | Trans-Golgi network integral membrane protein 2 | TGOLN2 | 4 | 14 | 0 | 119.3 | 1.53 | 1.85 | 1.31 | NaN | 1.50 | 1.30 | 0.63 | NaN | 1.21 |
| O43592 | Exportin-T | XPOT | 5 | 7.8 | 0 | 9.218 | 0.89 | 0.78 | 0.88 | NaN | 1.10 | 1.08 | NaN | NaN | NaN |
| O43615 | Mitochondrial import inner membrane translocase subunit TIM44 | TIMM44 | 6 | 15.3 | 0 | 15.14 | 0.95 | 1.09 | 1.29 | 0.96 | NaN | 1.07 | 0.90 | 1.13 | 1.04 |
| O43684 | Mitotic checkpoint protein BUB3 | BUB3 | 8 | 35.4 | 0 | 27.91 | 1.05 | 0.95 | 1.03 | 0.73 | 1.04 | 0.96 | 0.81 | 1.04 | 1.04 |
| O43707 | Alpha-actinin-4 | ACTN4 | 45 | 74.2 | 0 | 323.3 | 1.05 | 1.04 | 1.02 | 1.05 | 0.99 | 1.00 | 1.01 | 1.02 | 1.03 |
| O43719 | HIV Tat-specific factor 1 | HTATSF1 | 2 | 3 | 0.003 | 1.822 | 1.07 | 1.61 | NaN | NaN | NaN | 1.12 | NaN | NaN | NaN |
| O43765 | Small glutamine-rich tetratricopeptide repeat-containing protein alpha | SGTA | 8 | 29.4 | 0 | 77.26 | 1.19 | 1.22 | 1.32 | 0.98 | 1.23 | 1.13 | 1.18 | 0.80 | 1.23 |
| O43776 | Asparagine--tRNA ligase, cytoplasmic | NARS | 8 | 19.5 | 0 | 13.35 | 0.72 | 0.75 | 0.72 | 1.79 | 0.71 | 1.04 | NaN | NaN | 1.40 |
| O43852 | Calumenin | CALU | 20 | 72.4 | 0 | 323.3 | 1.16 | 1.09 | 1.19 | 1.17 | 1.09 | 1.11 | 1.17 | 1.17 | 0.97 |
| O60361 | Putative nucleoside diphosphate kinase | NME2P1 | 1 | 66.4 | 0.001 | 2.415 | 1.32 | 1.36 | 1.41 | 1.40 | 1.22 | 1.26 | 1.61 | 1.49 | 1.57 |
| O60488 | Long-chain-fatty-acid--CoA ligase 4 | ACSL4 | 11 | 29.3 | 0 | 55.16 | 0.85 | 0.89 | 0.61 | 1.24 | 0.75 | 0.63 | 1.24 | 1.14 | 1.08 |
| O60493 | Sorting nexin-3 | SNX3 | 9 | 67.3 | 0 | 31.17 | 1.56 | 1.38 | 1.52 | 1.27 | 1.57 | 1.36 | 1.13 | 1.13 | 1.25 |
| O60502 | Protein O-GlcNAcase | MGEA5 | 2 | 3.8 | 0 | 3.574 | NaN | NaN | 0.77 | NaN | 0.64 | 0.58 | NaN | NaN | NaN |
| O60506 | Heterogeneous nuclear ribonucleoprotein Q | SYNCRIP | 5 | 47.8 | 0 | 323.3 | 0.86 | 0.82 | 0.84 | 0.86 | 0.91 | 0.95 | 0.75 | 0.81 | 0.90 |

| | | | | | | | | | | | | | | |
|---|---|---|---|---|---|---|---|---|---|---|---|---|---|---|
| O60568 | Procollagen-lysine,2-oxoglutarate 5-dioxygenase 3 | PLOD3 | 10 | 18.8 | 0 | 29.07 | 1.19 | 1.44 | 1.19 | NaN | 0.97 | 1.07 | NaN | 1.06 | 1.26 |
| O60664 | Perilipin-3 | PLIN3 | 21 | 66.4 | 0 | 323.3 | 0.96 | 0.89 | 0.94 | 1.00 | 0.98 | 0.91 | 1.05 | 1.10 | 1.04 |
| O60701 | UDP-glucose 6-dehydrogenase | UGDH | 7 | 19.8 | 0 | 11.05 | 1.22 | 1.21 | 1.12 | 1.56 | 1.33 | 1.42 | NaN | 1.00 | NaN |
| O60749 | Sorting nexin-2 | SNX2 | 4 | 13.9 | 0 | 6.876 | NaN | 0.96 | NaN | NaN | NaN | 0.71 | 0.87 | 1.06 | NaN |
| O60763 | General vesicular transport factor p115 | USO1 | 14 | 21.3 | 0 | 323.3 | 1.07 | 1.02 | 1.18 | 0.98 | 0.93 | 0.88 | NaN | 1.30 | 1.11 |
| U3KQK0 | Histone H2B;Histone H2B type 1-L;Histone H2B type 1-M;Histone H2B type 1-N;Histone H2B type 1-H;Histone H2B type 2-F;Histone H2B type 1-C/E/F/G/I;Histone H2B type 1-D;Histone H2B type 1-K;Histone H2B type F-S;Histone H2B type | HIST1H2BI | 2 | 42.8 | 0 | 11.52 | 0.47 | 0.43 | 0.44 | 0.46 | 0.46 | 0.45 | 0.49 | 0.33 | 0.33 |
| O60869 | Endothelial differentiation-related factor 1 | EDF1 | 6 | 43.2 | 0 | 37.4 | 0.99 | 1.04 | 0.92 | 0.97 | 0.90 | 1.02 | 0.96 | 0.98 | 0.81 |
| O60884 | DnaJ homolog subfamily A member 2 | DNAJA2 | 6 | 24.5 | 0 | 28.64 | 1.31 | 1.06 | 1.40 | NaN | 0.91 | 1.10 | NaN | NaN | 0.83 |
| O60888 | Protein CutA | CUTA | 4 | 40.8 | 0 | 92.14 | 0.90 | 1.03 | 1.07 | 0.89 | 0.86 | 0.94 | 0.91 | 1.00 | 0.86 |
| O75083 | WD repeat-containing protein 1 | WDR1 | 33 | 67 | 0 | 323.3 | 0.85 | 0.82 | 0.80 | 0.86 | 0.86 | 0.82 | 1.08 | 0.94 | 0.99 |
| O75131 | Copine-3 | CPNE3 | 11 | 26.1 | 0 | 79.9 | 0.84 | 0.82 | 0.83 | 0.73 | 0.86 | 0.92 | 0.73 | 1.22 | 1.10 |
| O75223 | Gamma-glutamylcyclotransferase | GGCT | 4 | 31.9 | 0 | 86.19 | 0.69 | 0.89 | 1.37 | 0.60 | NaN | 0.77 | 1.35 | 0.98 | 0.83 |
| O75340 | Programmed cell death protein 6 | PDCD6 | 5 | 39.3 | 0 | 61.98 | 0.71 | 0.78 | 0.85 | 0.77 | 0.83 | 0.90 | NaN | NaN | 0.98 |
| O75348 | V-type proton ATPase subunit G | ATP6V1G1 | 2 | 21.2 | 0 | 4.913 | 1.71 | 1.52 | NaN | NaN | 1.22 | NaN | NaN | NaN | 1.74 |
| O75351 | Vacuolar protein sorting-associated protein 4B | VPS4B | 2 | 9.9 | 5E-04 | 2.856 | 1.16 | 1.07 | 1.05 | NaN | NaN | 1.00 | NaN | NaN | 0.93 |
| O75367 | Core histone macro-H2A.1;Histone H2A | H2AFY | 11 | 41.9 | 0 | 195.8 | 1.21 | 1.11 | 1.11 | 1.03 | 1.03 | 1.21 | 1.11 | 1.17 | 1.26 |
| O75368 | SH3 domain-binding glutamic acid-rich-like protein | SH3BGRL | 4 | 51.8 | 0 | 12.22 | 1.04 | 1.06 | 1.12 | 1.06 | NaN | 1.06 | 1.12 | 1.09 | 1.25 |
| O75369 | Filamin-B | FLNB | 129 | 73.6 | 0 | 323.3 | 1.12 | 1.09 | 1.10 | 1.03 | 1.01 | 1.01 | 1.11 | 1.08 | 1.09 |
| O75396 | Vesicle-trafficking protein | SEC22B | 8 | 42.3 | 0 | 89.19 | 0.99 | 1.05 | 1.06 | 1.04 | 1.03 | 1.07 | 0.97 | 1.01 | 0.95 |
| O75436 | Vacuolar protein sorting-associated protein 26A | VPS26A | 10 | 38.5 | 0 | 64.05 | 0.92 | 1.19 | 1.27 | 0.93 | 0.77 | 1.15 | NaN | NaN | 0.69 |
| O75475 | PC4 and SFRS1-interacting protein | PSIP1 | 3 | 10.8 | 0 | 6.28 | 0.60 | 0.47 | NaN | NaN | NaN | NaN | NaN | NaN | 0.75 |
| O75477 | Erlin-1 | ERLIN1 | 3 | 14.7 | 0 | 75.84 | 0.94 | 1.02 | 1.05 | 0.98 | 1.05 | 1.03 | 0.74 | 0.80 | 0.78 |
| Q5JRI1 | Serine/arginine-rich splicing factor 10 | SRSF10 | 3 | 22.7 | 0 | 15.21 | NaN | NaN | 0.71 | NaN | 0.67 | 0.84 | NaN | NaN | NaN |
| O75531 | Barrier-to-autointegration factor;Barrier-to-autointegration factor, N-terminally processed | BANF1 | 8 | 67.4 | 0 | 128 | 1.21 | 1.26 | 1.23 | 1.11 | 1.15 | 1.18 | 1.26 | 1.26 | 1.31 |
| O75533 | Splicing factor 3B subunit 1 | SF3B1 | 27 | 30.9 | 0 | 249.8 | 0.95 | 0.94 | 0.91 | 0.98 | 0.97 | 0.97 | 0.93 | 0.98 | 0.91 |
| O75534 | Cold shock domain-containing protein E1 | CSDE1 | 9 | 14 | 0 | 8.572 | 1.50 | 1.32 | 1.17 | 0.59 | 1.05 | 1.33 | 0.86 | 0.93 | 1.06 |
| O75607 | Nucleoplasmin-3 | NPM3 | 2 | 20.8 | 0 | 31.32 | 0.90 | NaN | 1.20 | NaN | NaN | NaN | NaN | NaN | NaN |

| | | | | | | | | | | | | | | |
|---|---|---|---|---|---|---|---|---|---|---|---|---|---|---|
| O75643 | U5 small nuclear ribonucleoprotein 200 kDa | SNRNP20( | 18 | 10.9 | 0 | 38.16 | 0.75 | 0.97 | 1.00 | NaN | 0.94 | 1.11 | 1.29 | 1.18 | 1.10 |
| O75663 | TIP41-like protein | TIPRL | 5 | 28.3 | 0 | 6.936 | 1.36 | 1.42 | 1.26 | NaN | NaN | 1.02 | 1.27 | NaN | 1.66 |
| O75695 | Protein XRP2 | RP2 | 3 | 8.3 | 0.008 | 1.547 | 1.28 | 0.90 | 0.82 | 0.98 | 0.65 | NaN | NaN | 0.96 | 1.00 |
| O75822 | Eukaryotic translation initiation factor 3 subunit J | EIF3J | 5 | 20.5 | 0 | 5.276 | 1.51 | NaN | NaN | 1.06 | 0.76 | NaN | 1.01 | 0.88 | NaN |
| O75844 | CAAX prenyl protease 1 homolog | ZMPSTE24 | 5 | 13.9 | 0 | 18.89 | 1.10 | 1.62 | 1.90 | 1.05 | 1.01 | NaN | 0.84 | 1.03 | 1.06 |
| O75874 | Isocitrate dehydrogenase [NADP] cytoplasmic | IDH1 | 14 | 35 | 0 | 27.31 | 0.67 | 0.65 | 0.80 | 0.66 | 0.74 | 0.69 | 0.71 | 0.69 | 0.74 |
| O75915 | PRA1 family protein 3 | ARL6IP5 | 3 | 20.7 | 0 | 26.66 | 0.72 | 0.96 | 0.93 | 0.87 | 0.78 | 1.02 | 0.83 | 0.66 | 0.81 |
| O75934 | Pre-mRNA-splicing factor SPF27 | BCAS2 | 3 | 29.8 | 0 | 9.632 | 1.00 | 0.76 | NaN | NaN | NaN | NaN | NaN | NaN | NaN |
| O75937 | DnaJ homolog subfamily C member 8 | DNAJC8 | 5 | 32 | 0 | 32.58 | 0.91 | 0.85 | 1.23 | 0.76 | NaN | 0.81 | NaN | NaN | 1.03 |
| O75947 | ATP synthase subunit d, mitochondrial | ATP5H | 6 | 51.6 | 0 | 23.52 | 0.97 | 1.00 | 1.03 | 1.09 | 1.05 | 1.17 | 1.17 | 1.25 | 0.96 |
| O75976 | Carboxypeptidase D | CPD | 12 | 11.4 | 0 | 131.6 | 1.21 | 1.26 | 0.98 | 1.00 | 1.35 | 1.18 | 0.95 | 1.29 | 0.67 |
| O76003 | Glutaredoxin-3 | GLRX3 | 10 | 41.5 | 0 | 65.54 | 1.02 | 1.03 | 1.07 | 0.96 | 0.79 | 0.86 | 0.92 | 1.15 | 0.85 |
| O76021 | Ribosomal L1 domain-containing protein 1 | RSL1D1 | 7 | 16.1 | 0 | 14.99 | NaN | 0.75 | 1.18 | NaN | 1.01 | 1.41 | NaN | NaN | NaN |
| O76094 | Signal recognition particle subunit SRP72 | SRP72 | 9 | 24.1 | 0 | 19.14 | 0.83 | 1.05 | NaN | 0.84 | 1.20 | NaN | NaN | NaN | 0.58 |
| O94760 | N(G),N(G)-dimethylarginine dimethylaminohydrolase 1 | DDAH1 | 11 | 50.9 | 0 | 44.43 | 1.17 | 0.96 | 1.12 | 1.23 | 0.98 | 1.07 | 1.28 | 1.30 | 1.27 |
| O94804 | Serine/threonine-protein kinase | STK10 | 5 | 6.3 | 0 | 14.27 | NaN | NaN | NaN | 1.09 | 1.06 | NaN | 0.97 | NaN | NaN |
| O94826 | Mitochondrial import receptor subunit TOM70 | TOMM70A | 12 | 29.1 | 0 | 113.1 | 1.08 | 1.16 | 1.05 | 1.27 | 1.18 | 1.26 | 1.13 | 1.00 | 0.80 |
| O94919 | Endonuclease domain-containing 1 protein | ENDOD1 | 2 | 6.8 | 0 | 4.182 | 0.97 | 1.22 | NaN | NaN | NaN | NaN | 1.60 | NaN | NaN |
| O94925 | Glutaminase kidney isoform, mitochondrial | GLS | 11 | 24.7 | 0 | 82.57 | 1.19 | 1.94 | 1.49 | 1.33 | 1.09 | 1.20 | 1.26 | 1.32 | 1.22 |
| O94973 | AP-2 complex subunit alpha-2 | AP2A2 | 6 | 19.4 | 0 | 14.13 | 0.80 | 1.05 | 0.80 | NaN | 0.86 | 0.76 | NaN | NaN | 0.87 |
| O95197 | Reticulon-3 | RTN3 | 1 | 1.1 | 0 | 6.785 | NaN | 1.13 | 0.95 | 0.79 | 0.81 | 1.00 | 0.91 | 0.83 | NaN |
| O95202 | LETM1 and EF-hand domain-containing protein 1, | LETM1 | 7 | 15.2 | 0 | 27.07 | 0.82 | 0.86 | 0.91 | NaN | 0.80 | NaN | NaN | NaN | 0.98 |
| O95292 | Vesicle-associated membrane protein-associated protein B/C | VAPB | 7 | 38.3 | 0 | 12.01 | 1.49 | 0.87 | 1.57 | NaN | 1.38 | NaN | NaN | 0.71 | 1.83 |
| O95336 | 6-phosphogluconolactonase | PGLS | 12 | 67.4 | 0 | 42.15 | 1.06 | 1.04 | 1.19 | 1.10 | 1.30 | 1.00 | 1.04 | 1.06 | NaN |
| O95340 | Bifunctional 3-phosphoadenosine 5-phosphosulfate synthase 2;Sulfate adenylyltransferase;Adenylyl-sulfate kinase | PAPSS2 | 5 | 13.4 | 0 | 13.66 | 0.81 | 0.91 | 0.82 | NaN | 1.04 | 1.13 | 1.37 | 0.58 | 0.67 |
| O95347 | Structural maintenance of chromosomes protein 2 | SMC2 | 14 | 17.5 | 0 | 22.98 | 0.73 | NaN | 0.73 | 0.83 | 0.97 | 0.94 | 0.81 | NaN | 0.81 |
| O95372 | Acyl-protein thioesterase 2 | LYPLA2 | 3 | 29.9 | 0 | 42.73 | 0.78 | 0.91 | NaN | NaN | NaN | 1.29 | NaN | NaN | 0.71 |
| O95373 | Importin-7 | IPO7 | 23 | 28.4 | 0 | 323.3 | 1.13 | 1.16 | 1.11 | 0.97 | 0.98 | 1.16 | 0.86 | 0.89 | 1.14 |
| Q5H8X8 | Urotensin-2 | UTS2 | 1 | 5.8 | 0 | 8.813 | 0.98 | 1.03 | 1.04 | 0.98 | 0.90 | 0.89 | 1.08 | 1.11 | 1.02 |

| | | | | | | | | | | | | | | |
|---|---|---|---|---|---|---|---|---|---|---|---|---|---|---|
| O95433 | Activator of 90 kDa heat shock protein ATPase homolog 1 | AHSA1 | 9 | 37.6 | 0 | 52.89 | 1.19 | 1.39 | 1.02 | NaN | 1.13 | 1.09 | NaN | 1.09 | 0.91 |
| O95456 | Proteasome assembly chaperone 1 | PSMG1 | 5 | 22.6 | 0 | 10.29 | 0.71 | 1.07 | 0.77 | 1.22 | 0.70 | 0.91 | 0.98 | 1.36 | 0.87 |
| O95573 | Long-chain-fatty-acid--CoA ligase 3 | ACSL3 | 8 | 18.2 | 0 | 69.32 | 0.84 | NaN | 0.90 | NaN | NaN | 1.15 | 1.08 | 1.69 | 1.47 |
| O95630 | STAM-binding protein | STAMBP | 3 | 8.5 | 0.007 | 1.652 | 0.91 | NaN | 1.05 | NaN | NaN | 1.03 | NaN | NaN | 0.76 |
| O95747 | Serine/threonine-protein kinase OSR1 | OXSR1 | 10 | 23.3 | 0 | 117.9 | 1.16 | 1.06 | 1.14 | 1.05 | 1.08 | 1.12 | 1.41 | 1.03 | 1.12 |
| O95777 | U6 snRNA-associated Sm-like protein LSm8 | LSM8 | 5 | 71.9 | 0 | 18.16 | 1.10 | 1.04 | 1.09 | 0.92 | 1.13 | 1.00 | 1.02 | NaN | 1.15 |
| O95782 | AP-2 complex subunit alpha-1 | AP2A1 | 19 | 37.6 | 0 | 124.9 | 0.89 | 0.99 | 0.92 | 0.97 | 0.92 | 0.96 | 0.85 | 0.88 | 1.13 |
| O95793 | Double-stranded RNA-binding protein Staufen homolog 1 | STAU1 | 7 | 16.6 | 0 | 33.45 | 1.20 | 1.43 | 1.15 | 1.10 | 0.85 | 1.21 | 1.14 | NaN | 0.96 |
| O95810 | Serum deprivation-response protein | SDPR | 6 | 18.6 | 0 | 9.281 | 0.76 | 0.87 | 0.70 | 0.89 | 0.85 | 0.44 | 0.80 | 0.60 | 1.14 |
| O95816 | BAG family molecular chaperone regulator 2 | BAG2 | 10 | 50.2 | 0 | 53.65 | 0.88 | 0.93 | 0.88 | 0.88 | 0.81 | 1.02 | 0.99 | NaN | 0.85 |
| O95817 | BAG family molecular chaperone regulator 3 | BAG3 | 13 | 26.3 | 0 | 46.83 | 1.30 | 1.70 | 1.59 | 1.51 | 1.55 | 1.53 | NaN | 1.26 | NaN |
| O95833 | Chloride intracellular channel protein 3 | CLIC3 | 3 | 18.2 | 0 | 4.488 | 0.90 | 0.84 | 0.72 | NaN | NaN | 0.46 | 1.44 | 1.47 | 1.50 |
| O95864 | Fatty acid desaturase 2 | FADS2 | 3 | 7.4 | 0 | 3.182 | 1.04 | NaN | 1.27 | NaN | NaN | NaN | NaN | NaN | NaN |
| O95865 | N(G),N(G)-dimethylarginine dimethylaminohydrolase 2 | DDAH2 | 3 | 17.5 | 0 | 14.37 | NaN | 1.02 | 1.32 | 0.90 | NaN | NaN | 1.20 | 0.97 | 1.15 |
| O95881 | Thioredoxin domain-containing protein 12 | TXNDC12 | 3 | 22.7 | 0 | 3.612 | 1.02 | 1.02 | 1.01 | NaN | NaN | 1.05 | NaN | NaN | 1.04 |
| O96008 | Mitochondrial import receptor subunit TOM40 homolog | TOMM40 | 6 | 26 | 0 | 69.93 | 1.00 | 0.75 | 0.79 | 0.76 | 0.94 | 0.74 | NaN | 0.98 | 0.78 |
| O96019 | Actin-like protein 6A | ACTL6A | 2 | 6.8 | 0 | 3.043 | NaN | NaN | 0.82 | NaN | 0.69 | 0.52 | NaN | NaN | 0.86 |
| P00338 | L-lactate dehydrogenase A chain | LDHA | 30 | 81.6 | 0 | 323.3 | 0.94 | 0.96 | 0.92 | 1.03 | 1.03 | 0.97 | 1.11 | 1.07 | 1.06 |
| P00367 | Glutamate dehydrogenase 1, mitochondrial;Glutamate dehydrogenase 2, mitochondrial | GLUD1 | 20 | 43.4 | 0 | 244.1 | 0.97 | 0.93 | 1.03 | 0.86 | 1.02 | 0.93 | 0.84 | 0.86 | 1.00 |
| P00387 | NADH-cytochrome b5 reductase 3;NADH-cytochrome b5 reductase 3 membrane-bound form;NADH-cytochrome b5 reductase 3 soluble form | CYB5R3 | 10 | 52.2 | 0 | 189.4 | 1.04 | 1.09 | 0.97 | 0.91 | 0.99 | 1.04 | 0.85 | 0.98 | 1.07 |
| P00390 | Glutathione reductase, mitochondrial | GSR | 2 | 4.8 | 0 | 3.402 | 0.93 | 0.81 | 0.92 | 1.18 | 1.10 | 1.05 | 0.89 | 0.78 | 0.82 |
| P00403 | Cytochrome c oxidase subunit 2 | MT-CO2 | 4 | 24.7 | 0 | 58.59 | 1.07 | 1.02 | 0.95 | 1.18 | 1.08 | 1.08 | 0.96 | 0.93 | 0.98 |
| P00441 | Superoxide dismutase [Cu-Zn] | SOD1 | 8 | 77.9 | 0 | 323.3 | 1.32 | 1.26 | 1.27 | 1.14 | 1.12 | 1.14 | 1.07 | 1.03 | 1.07 |
| P00491 | Purine nucleoside phosphorylase | PNP | 6 | 31.5 | 0 | 10.55 | 0.82 | 0.86 | 0.91 | 1.07 | 0.54 | 0.70 | NaN | NaN | 0.72 |
| P00492 | Hypoxanthine-guanine phosphoribosyltransferase | HPRT1 | 8 | 45 | 0 | 137.5 | 1.01 | 1.29 | 1.04 | 1.08 | 1.09 | 1.11 | 1.09 | 1.14 | 1.00 |

| | | | | | | | | | | | | | | |
|---|---|---|---|---|---|---|---|---|---|---|---|---|---|---|
| P00505 | Aspartate aminotransferase, mitochondrial | GOT2 | 16 | 39.5 | 0 | 139 | 1.16 | 1.14 | 1.12 | 1.08 | 1.07 | 1.14 | 1.08 | 0.90 | 1.07 |
| P00533 | Epidermal growth factor receptor;Receptor protein-tyrosine kinase | EGFR | 14 | 17.9 | 0 | 41.73 | 1.24 | 0.95 | 1.13 | 1.13 | NaN | 1.15 | 1.17 | 1.28 | 1.11 |
| P00558 | Phosphoglycerate kinase 1 | PGK1 | 35 | 82.5 | 0 | 323.3 | 1.18 | 1.20 | 1.18 | 1.21 | 1.21 | 1.21 | 1.12 | 1.17 | 1.20 |
| Q5T9B7 | Adenylate kinase isoenzyme 1 | AK1 | 5 | 31.9 | 0 | 10.95 | 0.86 | 1.07 | 0.67 | 0.90 | NaN | 0.66 | 1.00 | 0.74 | 0.74 |
| P00846 | ATP synthase subunit a | MT-ATP6 | 1 | 4.4 | 0.008 | 1.537 | 0.98 | 0.93 | NaN | 1.18 | NaN | NaN | NaN | 1.46 | NaN |
| P01111 | GTPase NRas;GTPase KRas;GTPase KRas, N-terminally processed;GTPase HRas;GTPase HRas, N- | NRAS | 4 | 33.3 | 0.001 | 2.441 | 0.69 | 0.45 | 0.62 | 0.72 | NaN | 0.81 | 0.30 | 0.50 | 0.41 |
| P02545 | Prelamin-A/C;Lamin-A/C | LMNA | 3 | 69.4 | 0 | 323.3 | 0.94 | 0.94 | 0.92 | 1.03 | 1.03 | 0.99 | 0.99 | 1.04 | 0.98 |
| P02786 | Transferrin receptor protein 1;Transferrin receptor protein 1, serum form | TFRC | 30 | 44.2 | 0 | 323.3 | 1.27 | 1.30 | 1.31 | 1.30 | 1.40 | 1.50 | 1.12 | 1.08 | 1.25 |
| P02795 | Metallothionein-2;Metallothionein- | MT2A | 2 | 67.2 | 0 | 151.4 | 1.91 | 1.94 | 1.80 | 1.58 | 1.58 | 1.52 | 1.08 | 1.22 | 1.30 |
| P04040 | Catalase | CAT | 16 | 40.8 | 0 | 60.08 | 1.10 | 1.08 | 1.02 | 0.96 | 0.99 | 1.24 | 1.03 | 1.02 | 0.96 |
| P04075 | Fructose-bisphosphate aldolase A;Fructose-bisphosphate | ALDOA | 27 | 84.1 | 0 | 323.3 | 1.03 | 1.06 | 1.05 | 1.07 | 1.10 | 1.05 | 1.11 | 1.09 | 1.09 |
| P04080 | Cystatin-B | CSTB | 7 | 88.8 | 0 | 323.3 | 1.16 | 1.35 | 1.09 | 1.01 | 1.19 | 1.20 | 0.67 | 0.58 | 1.11 |
| P04083 | Annexin A1 | ANXA1 | 25 | 71.1 | 0 | 323.3 | 0.94 | 0.95 | 0.92 | 0.99 | 0.99 | 0.99 | 0.86 | 0.86 | 0.86 |
| P04179 | Superoxide dismutase [Mn], mitochondrial | SOD2 | 6 | 30.6 | 0 | 31.37 | 0.99 | 0.75 | 0.90 | 0.82 | 1.08 | 1.13 | 0.58 | 1.00 | 0.66 |
| P04181 | Ornithine aminotransferase, mitochondrial;Ornithine aminotransferase, hepatic form;Ornithine aminotransferase, renal form | OAT | 15 | 48.7 | 0 | 210.1 | 0.71 | 0.59 | 0.65 | 0.74 | 0.78 | 0.77 | 0.92 | 1.00 | 1.11 |
| P04406 | Glyceraldehyde-3-phosphate dehydrogenase | GAPDH | 27 | 80.9 | 0 | 323.3 | 0.93 | 0.90 | 0.92 | 0.98 | 0.99 | 0.96 | 0.95 | 0.95 | 0.93 |
| P04792 | Heat shock protein beta-1 | HSPB1 | 11 | 78 | 0 | 323.3 | 0.86 | 0.82 | 0.86 | 0.82 | 0.82 | 0.84 | 1.00 | 0.90 | 0.89 |
| P04843 | Dolichyl-diphosphooligosaccharide--protein glycosyltransferase | RPN1 | 23 | 44.8 | 0 | 263.3 | 1.10 | 0.97 | 1.06 | 0.97 | 0.98 | 1.08 | 1.05 | 1.00 | 0.98 |
| P04844 | Dolichyl-diphosphooligosaccharide--protein glycosyltransferase | RPN2 | 13 | 31.2 | 0 | 204.6 | 0.98 | 0.96 | 0.97 | 1.16 | 1.11 | 1.06 | 1.20 | 1.17 | 0.99 |
| P04899 | Guanine nucleotide-binding protein G(i) subunit alpha-2;Guanine nucleotide-binding protein G(i) subunit alpha-1 | GNAI2 | 5 | 34.9 | 0 | 43.46 | 0.72 | 0.76 | 0.82 | 0.77 | 0.89 | 0.96 | 0.87 | 0.77 | 0.96 |
| P05023 | Sodium/potassium-transporting ATPase subunit alpha-1 | ATP1A1 | 32 | 36 | 0 | 323.3 | 0.94 | 0.99 | 1.01 | 1.26 | 1.31 | 1.30 | 0.77 | 0.75 | 0.86 |
| P05026 | Sodium/potassium-transporting ATPase subunit beta-1 | ATP1B1 | 10 | 36.3 | 0 | 26.75 | 1.04 | 0.78 | 0.83 | 1.16 | 1.14 | 1.63 | 1.41 | 0.85 | NaN |

| | | | | | | | | | | | | | | | |
|---|---|---|---|---|---|---|---|---|---|---|---|---|---|---|---|
| P05091 | Aldehyde dehydrogenase, mitochondrial | ALDH2 | 8 | 23 | 0 | 33.59 | 1.21 | NaN | NaN | NaN | 0.55 | 0.95 | NaN | NaN | 0.72 |
| P05114 | Non-histone chromosomal protein HMG-14 | HMGN1 | 7 | 50 | 0 | 195.2 | 0.69 | 0.76 | 0.73 | 0.52 | 0.80 | 0.71 | 0.77 | 0.60 | 0.86 |
| P05121 | Plasminogen activator inhibitor 1 | SERPINE1 | 12 | 40 | 0 | 241.6 | 13.08 | 8.99 | 10.74 | NaN | NaN | 1.76 | NaN | 16.26 | 13.89 |
| P05141 | ADP/ATP translocase 2;ADP/ATP translocase 2, N-terminally processed | SLC25A5 | 5 | 44.6 | 0 | 39.26 | 1.05 | 1.12 | 1.11 | 0.96 | 1.11 | 1.07 | 0.98 | 0.99 | 1.05 |
| P05198 | Eukaryotic translation initiation factor 2 subunit 1 | EIF2S1 | 16 | 54.6 | 0 | 323.3 | 0.98 | 0.82 | 0.94 | 1.07 | 0.92 | 1.02 | 0.80 | 0.90 | 0.75 |
| P05204 | Non-histone chromosomal protein HMG-17 | HMGN2 | 4 | 45.6 | 0 | 215.3 | 0.76 | 0.76 | NaN | 0.95 | 0.61 | 0.49 | NaN | 0.66 | 0.68 |
| P05386 | 60S acidic ribosomal protein P1 | RPLP1 | 2 | 51.8 | 0 | 234.9 | 0.94 | 1.01 | 0.98 | 0.88 | 1.03 | 0.89 | 0.99 | 0.94 | 1.01 |
| P05387 | 60S acidic ribosomal protein P2 | RPLP2 | 8 | 78.3 | 0 | 323.3 | 0.98 | 1.11 | 1.02 | 0.94 | 0.98 | 0.91 | 0.99 | 0.96 | 0.98 |
| P05388 | 60S acidic ribosomal protein P0;60S acidic ribosomal protein P0-like | RPLP0 | 18 | 65.6 | 0 | 323.3 | 0.86 | 0.85 | 0.87 | 0.85 | 0.81 | 0.85 | 0.88 | 0.82 | 0.79 |
| P05455 | Lupus La protein | SSB | 26 | 57.4 | 0 | 154.1 | 0.82 | 0.87 | 0.89 | 0.86 | 0.94 | 0.86 | 0.92 | 0.93 | 0.82 |
| P05556 | Integrin beta-1 | ITGB1 | 25 | 36.8 | 0 | 323.3 | 1.37 | 1.33 | 1.31 | 1.16 | 1.17 | 1.21 | 1.28 | 1.24 | 1.39 |
| P05783 | Keratin, type I cytoskeletal 18 | KRT18 | 33 | 76.5 | 0 | 323.3 | 1.21 | 1.21 | 1.02 | 0.97 | 0.93 | 0.96 | 1.48 | 1.26 | 1.16 |
| P05976 | Myosin light chain 1/3, skeletal muscle isoform;Myosin light chain 3 | MYL1 | 2 | 8.2 | 0 | 190.4 | 0.93 | 0.81 | 0.82 | 1.05 | 1.04 | 0.99 | 0.67 | 0.63 | 0.76 |
| P06493 | Cyclin-dependent kinase 1 | CDK1 | 14 | 62 | 0 | 45.21 | 0.95 | 1.04 | 0.85 | 1.05 | 1.12 | 0.98 | 1.03 | 0.95 | 0.84 |
| P06576 | ATP synthase subunit beta, mitochondrial;ATP synthase subunit beta | ATP5B | 21 | 57.5 | 0 | 323.3 | 0.98 | 1.02 | 1.02 | 1.07 | 1.06 | 1.03 | 0.84 | 0.78 | 0.87 |
| R4GN98 | Protein S100;Protein S100-A6 | S100A6 | 7 | 60 | 0 | 67.15 | 0.98 | 0.96 | 0.87 | 0.98 | 0.94 | 0.98 | 0.89 | 0.84 | 0.74 |
| P06733 | Alpha-enolase | ENO1 | 33 | 79.5 | 0 | 323.3 | 0.96 | 0.95 | 0.97 | 1.03 | 0.99 | 1.03 | 0.97 | 0.91 | 0.95 |
| P06737 | Glycogen phosphorylase, liver form;Alpha-1,4 glucan phosphorylase | PYGL | 25 | 44.2 | 0 | 253.5 | 0.72 | 0.89 | 0.93 | 0.94 | 0.68 | 0.99 | 0.73 | 1.01 | 1.04 |
| P06748 | Nucleophosmin | NPM1 | 18 | 56.1 | 0 | 323.3 | 0.94 | 0.96 | 0.92 | 0.96 | 1.01 | 0.98 | 0.91 | 0.91 | 0.85 |
| Q16778 | Histone H2B type 2-E;Histone H2B type 1-B;Histone H2B type 1-O;Histone H2B type 1-J | HIST2H2BI | 0 | 56.3 | 0 | 323.3 | 0.79 | 0.78 | 0.77 | 0.79 | 0.77 | 0.80 | 0.66 | 0.74 | 0.67 |
| P07108 | Acyl-CoA-binding protein | DBI | 7 | 73.6 | 0 | 323.3 | 0.83 | 0.76 | 0.85 | 0.96 | 1.07 | 0.88 | 0.87 | 0.78 | 0.90 |
| P07195 | L-lactate dehydrogenase B chain;L-lactate dehydrogenase | LDHB | 16 | 52.4 | 0 | 323.3 | 0.96 | 0.95 | 0.94 | 0.92 | 0.96 | 0.98 | 0.94 | 0.88 | 0.92 |
| P07237 | Protein disulfide-isomerase | P4HB | 39 | 78 | 0 | 323.3 | 1.07 | 1.08 | 1.09 | 1.06 | 1.08 | 1.11 | 1.04 | 1.03 | 1.05 |
| P07355 | Annexin A2;Annexin;Putative annexin A2-like protein | ANXA2 | 16 | 79.9 | 0 | 323.3 | 0.82 | 0.82 | 0.81 | 0.86 | 0.83 | 0.84 | 0.98 | 0.94 | 0.91 |
| P07384 | Calpain-1 catalytic subunit | CAPN1 | 12 | 20.7 | 0 | 37.55 | 0.72 | 0.74 | 0.81 | 0.85 | 0.88 | 0.73 | 1.02 | 1.25 | 0.81 |
| Q5JP53 | Tubulin beta chain | TUBB | 5 | 66 | 0 | 323.3 | 0.41 | 0.40 | 0.41 | 0.49 | 0.47 | 0.47 | 0.85 | 0.81 | 0.83 |
| P07602 | Prosaposin;Saposin-A;Saposin-B-Val;Saposin-B;Saposin-C;Saposin-D | PSAP | 13 | 26.1 | 0 | 56.57 | 1.55 | 1.47 | 1.52 | 1.55 | 1.26 | 1.46 | 1.36 | 1.63 | 1.37 |

| | | | | | | | | | | | | | | | |
|---|---|---|---|---|---|---|---|---|---|---|---|---|---|---|---|
| Q5URX0 | Beta-hexosaminidase subunit beta;Beta-hexosaminidase subunit beta chain B;Beta-hexosaminidase subunit beta | HEXB | 4 | 15.7 | 0 | 6.247 | 1.01 | NaN | NaN | 1.20 | 1.18 | 1.27 | NaN | NaN | NaN |
| P07737 | Profilin-1 | PFN1 | 12 | 89.3 | 0 | 323.3 | 1.10 | 1.15 | 1.15 | 1.16 | 1.11 | 1.08 | 1.10 | 1.01 | 0.97 |
| P07741 | Adenine phosphoribosyltransferase | APRT | 9 | 58.9 | 0 | 43.54 | 1.49 | 1.30 | 2.18 | NaN | 1.60 | 0.96 | 1.25 | 1.10 | 1.21 |
| P07814 | Bifunctional glutamate/proline--tRNA ligase;Glutamate--tRNA ligase;Proline--tRNA ligase | EPRS | 34 | 32.8 | 0 | 211.2 | 0.96 | 0.78 | 0.97 | 0.89 | 0.84 | 0.89 | 0.74 | 0.80 | 0.92 |
| P07858 | Cathepsin B;Cathepsin B light chain;Cathepsin B heavy chain | CTSB | 6 | 24.2 | 0 | 36.75 | 1.44 | 0.98 | 1.33 | 1.32 | 1.23 | 1.60 | 1.36 | 1.24 | 1.31 |
| P07900 | Heat shock protein HSP 90-alpha | HSP90AA1 | 33 | 61.3 | 0 | 323.3 | 1.14 | 1.17 | 1.15 | 1.17 | 1.22 | 1.20 | 0.88 | 0.83 | 0.84 |
| P07919 | Cytochrome b-c1 complex subunit 6, mitochondrial;Cytochrome b-c1 | UQCRH | 2 | 28.6 | 0 | 7.431 | 1.78 | 1.69 | 1.10 | NaN | 1.11 | 1.20 | 1.73 | NaN | NaN |
| P07954 | Fumarate hydratase, | FH | 15 | 48.4 | 0 | 59.62 | 0.98 | 1.06 | NaN | NaN | 0.90 | 1.23 | 1.36 | 0.95 | 0.60 |
| P08133 | Annexin A6;Annexin | ANXA6 | 15 | 63 | 0 | 323.3 | 0.88 | 0.84 | 0.91 | 0.89 | 0.87 | 0.92 | 1.12 | 1.01 | 0.94 |
| Q5JR08 | Rho-related GTP-binding protein RhoC | RHOC | 2 | 45.2 | 0 | 3.774 | 1.13 | 1.05 | 1.09 | NaN | 1.06 | 1.09 | NaN | NaN | 1.09 |
| P08238 | Heat shock protein HSP 90-beta | HSP90AB1 | 28 | 64.4 | 0 | 323.3 | 1.16 | 1.15 | 1.16 | 1.08 | 1.08 | 1.11 | 0.92 | 0.89 | 0.94 |
| P08240 | Signal recognition particle receptor subunit alpha | SRPR | 5 | 12.1 | 0 | 18.94 | 0.68 | 0.77 | 0.68 | NaN | NaN | 0.69 | NaN | NaN | NaN |
| P08243 | Asparagine synthetase [glutamine-hydrolyzing] | ASNS | 5 | 14.1 | 0 | 5.348 | NaN | 0.81 | NaN | 0.49 | 0.69 | 0.85 | NaN | 0.76 | 0.82 |
| P08559 | Pyruvate dehydrogenase E1 component subunit alpha, somatic form, mitochondrial | PDHA1 | 11 | 29 | 0 | 38.17 | 0.67 | 0.72 | 0.81 | 1.03 | 0.82 | 0.87 | 0.98 | 0.96 | 1.16 |
| P08574 | Cytochrome c1, heme protein, mitochondrial | CYC1 | 2 | 8.6 | 0 | 3.586 | 1.05 | 0.94 | 1.10 | 1.06 | 1.12 | 1.18 | NaN | 1.16 | 1.21 |
| P08579 | U2 small nuclear ribonucleoprotein B | SNRPB2 | 1 | 11.6 | 0 | 70.84 | 1.00 | 1.02 | 1.33 | NaN | 1.43 | 1.16 | NaN | NaN | 1.15 |
| P08582 | Melanotransferrin | MFI2 | 8 | 15.3 | 0 | 29.24 | 0.91 | 1.07 | 0.90 | NaN | NaN | 1.07 | NaN | 1.03 | 1.19 |
| P08621 | U1 small nuclear ribonucleoprotein 70 kDa | SNRNP70 | 12 | 30.7 | 0 | 233.4 | 0.90 | 1.03 | 0.89 | 0.91 | 0.85 | 1.06 | 0.76 | 0.64 | 0.81 |
| P08670 | Vimentin | VIM | 47 | 88.8 | 0 | 323.3 | 1.02 | 1.00 | 1.00 | 0.94 | 0.96 | 0.95 | 1.14 | 1.10 | 1.10 |
| P08754 | Guanine nucleotide-binding protein G(k) subunit alpha | GNAI3 | 2 | 25.7 | 0 | 31.42 | 0.51 | 0.67 | 0.56 | 0.58 | NaN | 0.73 | 0.81 | NaN | 0.78 |
| P08758 | Annexin A5;Annexin | ANXA5 | 27 | 80.6 | 0 | 323.3 | 1.07 | 1.08 | 1.13 | 1.00 | 1.06 | 1.02 | 0.96 | 0.97 | 0.95 |
| P09012 | U1 small nuclear ribonucleoprotein A | SNRPA | 5 | 21.3 | 0 | 9.592 | 0.74 | 0.75 | 0.81 | 0.77 | 0.74 | 0.73 | 0.69 | 0.81 | 0.73 |
| P09104 | Gamma-enolase;Enolase | ENO2 | 13 | 63.1 | 0 | 323.3 | 1.11 | 1.09 | 1.20 | 1.15 | 1.27 | 1.15 | 1.11 | 1.20 | 1.17 |
| P09211 | Glutathione S-transferase P | GSTP1 | 12 | 69.5 | 0 | 323.3 | 0.99 | 0.90 | 1.01 | 1.00 | 1.14 | 0.96 | 1.05 | 1.01 | 1.04 |
| P09382 | Galectin-1 | LGALS1 | 9 | 77.8 | 0 | 323.3 | 1.10 | 1.03 | 1.07 | 1.06 | 1.09 | 1.07 | 1.18 | 1.14 | 1.12 |
| Q5T7C4 | High mobility group protein B1;Putative high mobility group protein B1-like 1 | HMGB1 | 9 | 48.7 | 0 | 120.7 | 0.88 | 0.86 | 0.88 | 0.95 | 0.94 | 0.98 | 0.92 | 0.84 | 0.81 |

| | | | | | | | | | | | | | | | |
|---|---|---|---|---|---|---|---|---|---|---|---|---|---|---|---|
| P09496 | Clathrin light chain A | CLTA | 7 | 19.4 | 0 | 9.796 | 1.27 | 1.01 | 1.12 | 0.93 | 1.08 | 0.95 | 1.02 | 1.05 | 0.98 |
| P09497 | Clathrin light chain B | CLTB | 8 | 24.9 | 0 | 8.794 | 0.88 | 1.31 | 0.85 | 1.10 | 1.04 | 1.68 | 0.82 | 0.92 | 1.46 |
| P09525 | Annexin A4;Annexin | ANXA4 | 12 | 40.1 | 0 | 323.3 | 0.83 | 0.90 | 0.99 | 1.56 | 1.09 | 1.07 | 0.90 | 0.98 | 0.94 |
| P09543 | 2,3-cyclic-nucleotide 3-phosphodiesterase | CNP | 5 | 20 | 0 | 152.2 | 0.83 | 0.98 | 1.33 | 1.18 | 0.96 | 1.04 | 1.16 | NaN | 0.64 |
| P09622 | Dihydrolipoyl dehydrogenase, mitochondrial;Dihydrolipoyl dehydrogenase | DLD | 14 | 35.8 | 0 | 238.7 | 1.02 | 1.01 | 1.05 | 0.97 | 0.93 | 0.97 | 1.01 | 1.07 | 1.02 |
| P09874 | Poly [ADP-ribose] polymerase 1 | PARP1 | 26 | 32.7 | 0 | 86.42 | 0.78 | 0.75 | 0.77 | 0.81 | 0.97 | 0.82 | 0.90 | 0.84 | 1.05 |
| P09960 | Leukotriene A-4 hydrolase | LTA4H | 16 | 34.7 | 0 | 52.5 | 0.65 | 0.74 | 0.77 | 1.05 | 0.89 | 1.04 | 1.49 | 0.93 | 0.71 |
| P09972 | Fructose-bisphosphate aldolase C;Fructose-bisphosphate | ALDOC | 4 | 28.3 | 0 | 237.3 | 1.23 | 1.14 | 1.06 | 1.25 | 1.14 | 1.20 | 1.17 | 1.23 | 1.15 |
| Q71UI9 | Histone H2A.V;Histone H2A.Z;Histone H2A | H2AFV | 2 | 31.2 | 0 | 13.49 | NaN | 0.83 | NaN | 0.64 | NaN | NaN | 0.54 | NaN | 1.10 |
| P0DN76 | Splicing factor U2AF 35 kDa subunit | U2AF1 | 7 | 32.9 | 0 | 42.96 | 1.46 | 1.23 | 1.02 | 1.04 | NaN | 0.79 | 1.01 | 1.03 | 0.88 |
| P10155 | 60 kDa SS-A/Ro | TROVE2 | 2 | 4.1 | 0 | 4.793 | 0.69 | 0.86 | 0.86 | NaN | NaN | 0.83 | NaN | NaN | 0.72 |
| P10301 | Ras-related protein R-Ras | RRAS | 1 | 19.7 | 0.008 | 1.476 | 1.06 | 0.98 | 1.08 | 0.86 | NaN | 0.53 | NaN | 0.73 | 1.12 |
| P10515 | Dihydrolipoyllysine-residue acetyltransferase component of pyruvate dehydrogenase complex, mitochondrial;Acetyltransferase component of pyruvate | DLAT | 12 | 25.7 | 0 | 54.78 | 1.00 | 0.89 | 0.88 | 0.97 | 0.91 | 0.93 | 0.88 | 0.73 | 0.89 |
| P10599 | Thioredoxin | TXN | 9 | 88.6 | 0 | 135.8 | 1.22 | 1.18 | 1.16 | 1.17 | 1.17 | 1.16 | 1.11 | 0.95 | 0.97 |
| P10606 | Cytochrome c oxidase subunit 5B, mitochondrial | COX5B | 6 | 31 | 0 | 7.049 | 1.06 | 0.90 | 1.14 | 0.93 | 1.19 | 1.15 | 1.26 | 1.14 | 1.17 |
| X6R5C5 | Carboxypeptidase;Lysosomal protective protein;Lysosomal protective protein 32 kDa chain;Lysosomal protective protein 20 kDa chain | CTSA | 6 | 16.8 | 0 | 25.66 | 1.44 | 1.34 | 1.44 | 1.17 | 1.30 | 1.21 | 2.04 | 1.51 | 1.54 |
| P10644 | cAMP-dependent protein kinase type I-alpha regulatory subunit;cAMP-dependent protein kinase type I-alpha regulatory subunit, N-terminally processed | PRKAR1A | 6 | 19.2 | 0 | 41.88 | 0.86 | 1.14 | 1.30 | 0.79 | 1.45 | 0.92 | NaN | 0.92 | 1.27 |
| P10768 | S-formylglutathione hydrolase | ESD | 8 | 52.5 | 0 | 97.23 | NaN | 0.95 | 1.00 | 0.89 | 0.86 | 0.85 | 0.78 | 0.76 | 0.67 |
| P10809 | 60 kDa heat shock protein, mitochondrial | HSPD1 | 38 | 72.3 | 0 | 323.3 | 1.19 | 1.19 | 1.18 | 1.19 | 1.21 | 1.19 | 1.00 | 1.00 | 1.01 |
| P11021 | 78 kDa glucose-regulated protein | HSPA5 | 39 | 57.3 | 0 | 323.3 | 0.93 | 1.00 | 1.04 | 0.91 | 0.94 | 0.98 | 0.98 | 1.00 | 1.01 |
| P11047 | Laminin subunit gamma-1 | LAMC1 | 1 | 1.4 | 0 | 3.372 | 8.86 | 1.98 | 1.49 | NaN | NaN | NaN | NaN | NaN | 1.39 |
| P11142 | Heat shock cognate 71 kDa protein | HSPA8 | 27 | 72.3 | 0 | 323.3 | 1.30 | 1.31 | 1.31 | 1.27 | 1.26 | 1.25 | 0.93 | 0.91 | 0.93 |
| P11166 | Solute carrier family 2, facilitated glucose transporter member 1 | SLC2A1 | 8 | 15.2 | 0 | 299.8 | 1.15 | 1.22 | 1.30 | 1.00 | 1.06 | 1.01 | 1.21 | 0.92 | 1.26 |

| | | | | | | | | | | | | | | | |
|---|---|---|---|---|---|---|---|---|---|---|---|---|---|---|---|
| P11177 | Pyruvate dehydrogenase E1 component subunit beta, mitochondrial | PDHB | 11 | 42.9 | 0 | 109.2 | 1.08 | 0.52 | 0.95 | 1.00 | 1.13 | 1.06 | 1.05 | 1.14 | 1.27 |
| P11216 | Glycogen phosphorylase, brain form | PYGB | 27 | 48.2 | 0 | 166.4 | 0.89 | 0.79 | 0.79 | 0.87 | 0.77 | 0.89 | 0.95 | 0.85 | 0.94 |
| P11233 | Ras-related protein Ral-A | RALA | 2 | 21.8 | 0 | 43.17 | 0.89 | 1.08 | 0.83 | 1.19 | 1.05 | 0.93 | 1.24 | 1.17 | 0.72 |
| P11279 | Lysosome-associated membrane glycoprotein 1 | LAMP1 | 3 | 8.6 | 0 | 98.51 | 1.07 | 1.10 | 1.05 | 1.11 | 0.91 | 1.32 | 0.81 | 0.98 | 0.96 |
| P11388 | DNA topoisomerase 2-alpha | TOP2A | 3 | 5.3 | 0 | 6.997 | NaN | NaN | NaN | NaN | 1.87 | 1.80 | NaN | NaN | NaN |
| P11413 | Glucose-6-phosphate 1-dehydrogenase | G6PD | 28 | 64.7 | 0 | 323.3 | 0.94 | 0.89 | 1.01 | 1.08 | 1.07 | 1.10 | 0.83 | 0.92 | 0.96 |
| P11498 | Pyruvate carboxylase, mitochondrial | PC | 22 | 26.1 | 0 | 72.31 | 0.90 | 0.85 | 0.81 | 0.88 | 1.00 | 0.90 | 0.84 | 0.99 | 0.94 |
| P11717 | Cation-independent mannose-6-phosphate receptor | IGF2R | 7 | 3.2 | 0 | 5.257 | 1.39 | 0.60 | 0.89 | 0.96 | NaN | NaN | NaN | NaN | NaN |
| P11766 | Alcohol dehydrogenase class-3 | ADH5 | 10 | 40.1 | 0 | 57.47 | 0.85 | NaN | 0.73 | 0.86 | 0.47 | 0.84 | 0.85 | 1.05 | 0.97 |
| P11908 | Ribose-phosphate pyrophosphokinase 2 | PRPS2 | 1 | 27.7 | 0 | 5.664 | 0.74 | 0.76 | 0.50 | NaN | NaN | NaN | NaN | NaN | 0.76 |
| P11940 | Polyadenylate-binding protein 1;Polyadenylate-binding protein | PABPC1 | 20 | 53.6 | 0 | 323.3 | 0.91 | 1.00 | 0.86 | 1.09 | 1.05 | 1.07 | 0.98 | 0.87 | 0.73 |
| P12004 | Proliferating cell nuclear antigen | PCNA | 12 | 56.3 | 0 | 323.3 | 1.00 | 0.99 | 0.98 | 1.06 | 0.97 | 1.03 | 0.97 | 0.89 | 0.92 |
| P12235 | ADP/ATP translocase 1 | SLC25A4 | 2 | 47.7 | 0.004 | 1.802 | 0.97 | NaN | 0.74 | NaN | NaN | 0.66 | NaN | NaN | NaN |
| P12236 | ADP/ATP translocase 3;ADP/ATP translocase 3, N-terminally processed | SLC25A6 | 6 | 66.8 | 0 | 265.3 | 1.04 | 1.02 | 1.03 | 1.06 | 1.00 | 1.08 | 0.95 | 0.93 | 0.95 |
| P12268 | Inosine-5-monophosphate dehydrogenase 2 | IMPDH2 | 24 | 51.6 | 0 | 229.5 | 0.92 | 0.99 | 0.95 | 0.98 | 0.99 | 0.93 | 0.88 | 0.84 | 0.94 |
| P12270 | Nucleoprotein TPR | TPR | 20 | 11.2 | 0 | 53.14 | 0.94 | 1.24 | 0.99 | 0.74 | 0.88 | 1.69 | NaN | 1.35 | 0.89 |
| P12277 | Creatine kinase B-type | CKB | 4 | 12.3 | 0 | 26.41 | 2.08 | 2.01 | 0.94 | NaN | 1.00 | NaN | NaN | NaN | NaN |
| P12429 | Annexin A3;Annexin | ANXA3 | 18 | 59.1 | 0 | 309.1 | 0.99 | 0.86 | 0.87 | 0.99 | 0.93 | 0.99 | 1.02 | 1.01 | 0.94 |
| P12814 | Alpha-actinin-1 | ACTN1 | 41 | 73.2 | 0 | 323.3 | 1.19 | 1.16 | 1.17 | 1.10 | 1.04 | 1.08 | 1.12 | 1.22 | 1.18 |
| P12955 | Xaa-Pro dipeptidase | PEPD | 9 | 21.7 | 0 | 39.74 | NaN | 1.10 | NaN | NaN | NaN | 1.16 | 0.86 | 1.24 | NaN |
| P12956 | X-ray repair cross-complementing protein 6 | XRCC6 | 27 | 47.3 | 0 | 311.8 | 0.89 | 0.88 | 0.90 | 0.83 | 0.85 | 0.98 | 0.83 | 0.89 | 0.86 |
| P13010 | X-ray repair cross-complementing protein 5 | XRCC5 | 28 | 45.1 | 0 | 323.3 | 0.98 | 0.92 | 0.92 | 0.90 | 0.97 | 0.99 | 0.97 | 0.89 | 0.89 |
| P13073 | Cytochrome c oxidase subunit 4 isoform 1, mitochondrial | COX4I1 | 6 | 31.4 | 0 | 10.17 | 1.01 | 0.97 | 0.93 | 1.19 | NaN | 0.98 | 0.98 | 0.75 | 0.80 |
| P13473 | Lysosome-associated membrane glycoprotein 2 | LAMP2 | 3 | 7.3 | 0 | 34.14 | 1.00 | 0.98 | 1.22 | NaN | 0.74 | 1.11 | NaN | NaN | 1.00 |
| P13489 | Ribonuclease inhibitor | RNH1 | 22 | 64.9 | 0 | 323.3 | 0.91 | 0.86 | 0.92 | 0.92 | 0.91 | 0.93 | 1.14 | 1.14 | 1.14 |
| P13639 | Elongation factor 2 | EEF2 | 50 | 69.9 | 0 | 323.3 | 0.79 | 0.78 | 0.82 | 0.88 | 0.84 | 0.85 | 0.84 | 0.89 | 0.90 |
| P13667 | Protein disulfide-isomerase A4 | PDIA4 | 37 | 53.2 | 0 | 323.3 | 0.84 | 0.88 | 0.83 | 0.96 | 0.95 | 0.84 | 0.85 | 0.96 | 0.91 |
| P13674 | Prolyl 4-hydroxylase subunit alpha-1 | P4HA1 | 13 | 34.1 | 0 | 122.7 | 1.67 | 1.21 | 1.56 | 1.47 | 1.12 | 1.51 | 0.93 | 1.60 | 1.50 |
| P13693 | Translationally-controlled tumor protein | TPT1 | 9 | 39 | 0 | 114.2 | 1.13 | 1.34 | 1.17 | 1.32 | 1.22 | 1.30 | 0.94 | 0.95 | 1.01 |

| | | | | | | | | | | | | | | | |
|---|---|---|---|---|---|---|---|---|---|---|---|---|---|---|---|
| P13726 | Tissue factor | F3 | 9 | 37.3 | 0 | 323.3 | 1.14 | 1.18 | 0.97 | 1.18 | 1.19 | 1.20 | 1.08 | 1.04 | 0.99 |
| P13797 | Plastin-3 | PLS3 | 32 | 59.5 | 0 | 231.7 | 1.00 | 0.99 | 1.03 | 0.98 | 0.92 | 0.90 | 0.98 | 1.00 | 0.97 |
| P13804 | Electron transfer flavoprotein subunit alpha, mitochondrial | ETFA | 10 | 43.2 | 0 | 24.17 | 1.07 | NaN | 1.28 | 0.82 | 1.11 | 1.31 | 1.13 | NaN | 2.25 |
| P13807 | Glycogen [starch] synthase, muscle | GYS1 | 1 | 3.3 | 0 | 6.875 | 0.89 | 0.86 | NaN | NaN | NaN | 1.14 | NaN | NaN | 1.28 |
| P13861 | cAMP-dependent protein kinase type II-alpha regulatory subunit | PRKAR2A | 8 | 24.5 | 0 | 41.73 | 0.90 | 1.27 | 0.93 | 1.20 | 0.90 | 0.84 | 1.53 | NaN | 1.40 |
| P14174 | Macrophage migration inhibitory factor | MIF | 2 | 26.1 | 0 | 46.08 | 1.34 | 1.27 | 1.29 | 1.19 | 1.11 | 1.34 | 1.34 | 1.16 | 1.18 |
| P14324 | Farnesyl pyrophosphate | FDPS | 6 | 17.4 | 0 | 37.34 | 0.70 | 0.72 | 0.76 | 0.90 | 0.79 | 0.83 | 1.22 | 0.86 | 0.86 |
| V9GYG2 | Alcohol dehydrogenase | AKR1A1 | 2 | 18.5 | 0 | 4.092 | 0.64 | 0.66 | NaN | 0.83 | 0.78 | 0.87 | NaN | NaN | NaN |
| P14618 | Pyruvate kinase PKM;Pyruvate kinase | PKM | 5 | 83.8 | 0 | 323.3 | 0.90 | 0.92 | 0.89 | 0.99 | 0.98 | 1.00 | 0.99 | 0.94 | 0.89 |
| P14625 | Endoplasmin | HSP90B1 | 53 | 58.8 | 0 | 323.3 | 1.05 | 1.03 | 1.04 | 1.08 | 1.10 | 1.10 | 0.86 | 1.00 | 0.99 |
| P14854 | Cytochrome c oxidase subunit | COX6B1 | 4 | 58.1 | 0 | 65.81 | 0.97 | 1.01 | 1.08 | 1.14 | 1.12 | 1.02 | 1.57 | 1.70 | 1.08 |
| P14866 | Heterogeneous nuclear ribonucleoprotein L | HNRNPL | 8 | 49.9 | 0 | 323.3 | 0.94 | 0.91 | 1.03 | 1.06 | 0.97 | 0.99 | 0.82 | 1.00 | 0.95 |
| P14868 | Aspartate--tRNA ligase, cytoplasmic | DARS | 27 | 57.5 | 0 | 311 | 1.03 | 1.09 | 0.99 | 1.14 | 0.99 | 1.00 | 1.05 | 0.98 | 0.99 |
| P15121 | Aldose reductase | AKR1B1 | 15 | 59.5 | 0 | 123.5 | 0.86 | 0.98 | 0.94 | 0.92 | 0.90 | 1.03 | 0.93 | 0.88 | 0.77 |
| P15170 | Eukaryotic peptide chain release factor GTP-binding subunit ERF3A;Eukaryotic peptide chain release factor GTP-binding subunit ERF3B | GSPT1 | 11 | 27.5 | 0 | 22.46 | 1.07 | 0.99 | 0.95 | 0.90 | 1.24 | 1.36 | NaN | 0.87 | 1.16 |
| P15374 | Ubiquitin carboxyl-terminal hydrolase isozyme L3;Ubiquitin carboxyl-terminal hydrolase | UCHL3 | 8 | 60.4 | 0 | 99.72 | 0.91 | 1.08 | 0.95 | 0.98 | 0.99 | 0.91 | 0.58 | 1.17 | 0.88 |
| P15531 | Nucleoside diphosphate kinase A | NME1 | 1 | 70.4 | 0 | 13.27 | 1.11 | 1.00 | 0.96 | 0.99 | 0.94 | 0.95 | 1.05 | 1.01 | 1.07 |
| P15586 | N-acetylglucosamine-6-sulfatase | GNS | 6 | 13.4 | 0 | 5.204 | 0.84 | 0.69 | NaN | 0.66 | NaN | 0.77 | NaN | NaN | 0.69 |
| P15880 | 40S ribosomal protein S2 | RPS2 | 16 | 54.3 | 0 | 130.7 | 0.85 | 0.81 | 0.76 | 0.83 | 0.79 | 0.82 | 0.87 | 0.84 | 0.84 |
| P15927 | Replication protein A 32 kDa subunit | RPA2 | 5 | 39.6 | 0 | 65.78 | 0.78 | 0.74 | 0.77 | NaN | 1.01 | 0.83 | 1.10 | NaN | 0.49 |
| P16104 | Histone H2AX;Histone H2A type 1-A | H2AFX | 2 | 38.5 | 0 | 243 | 0.88 | 0.83 | 0.95 | 0.83 | 0.82 | 0.88 | 0.90 | 0.86 | 0.94 |
| P16144 | Integrin beta-4 | ITGB4 | 12 | 8.8 | 0 | 30.47 | NaN | 0.56 | 0.58 | NaN | 0.94 | 0.92 | NaN | 0.91 | 0.83 |
| P16152 | Carbonyl reductase [NADPH] 1 | CBR1 | 11 | 59.2 | 0 | 290.4 | 0.93 | 0.92 | 0.94 | 0.95 | 0.89 | 0.98 | 1.91 | 1.13 | 0.96 |
| P16403 | Histone H1.2 | HIST1H1C | 1 | 26.8 | 0 | 14.56 | 1.69 | 1.19 | 1.28 | 1.38 | 1.50 | 1.44 | 1.28 | 1.27 | 1.39 |
| P16615 | Sarcoplasmic/endoplasmic reticulum calcium ATPase 2 | ATP2A2 | 27 | 32 | 0 | 177.8 | 0.86 | 0.83 | 0.91 | 1.10 | 0.95 | 0.90 | 1.07 | 1.07 | 0.92 |
| P16930 | Fumarylacetoacetase | FAH | 4 | 14.1 | 0 | 8.331 | NaN | 0.82 | 0.50 | NaN | 0.70 | 1.01 | 0.97 | NaN | NaN |
| P16949 | Stathmin | STMN1 | 11 | 63.1 | 0 | 155.5 | 0.81 | 0.78 | 0.78 | 0.83 | 0.84 | 0.83 | 0.91 | 0.90 | 0.91 |
| P17066 | Heat shock 70 kDa protein 6;Putative heat shock 70 kDa protein 7 | HSPA6 | 1 | 17.4 | 0 | 58.27 | 1.48 | 1.42 | 1.40 | 1.35 | 1.30 | 1.33 | 1.04 | 1.01 | 0.98 |

| | | | | | | | | | | | | | | | |
|---|---|---|---|---|---|---|---|---|---|---|---|---|---|---|---|
| P17096 | High mobility group protein HMG-I/HMG-Y | HMGA1 | 5 | 40.2 | 0 | 119.9 | 1.03 | 1.07 | 1.12 | 0.90 | 0.96 | 0.84 | 0.96 | 0.98 | 1.10 |
| P17174 | Aspartate aminotransferase, cytoplasmic | GOT1 | 13 | 42.9 | 0 | 181.3 | 0.82 | 0.88 | 1.04 | 0.90 | 1.07 | 0.84 | 0.95 | 0.97 | 0.95 |
| P17252 | Protein kinase C alpha type | PRKCA | 8 | 16.8 | 0 | 25.31 | NaN | 0.40 | 0.36 | 0.89 | 1.73 | 0.71 | 0.67 | 0.80 | 0.41 |
| P17301 | Integrin alpha-2 | ITGA2 | 9 | 9.9 | 0 | 27.14 | 1.32 | 1.15 | 1.65 | NaN | 0.69 | 0.91 | NaN | 1.52 | 0.96 |
| P17612 | cAMP-dependent protein kinase catalytic subunit alpha;cAMP-dependent protein kinase catalytic subunit beta | PRKACA | 2 | 14 | 0 | 3.949 | 0.90 | 0.88 | 0.97 | NaN | NaN | NaN | NaN | 0.84 | 1.33 |
| P17655 | Calpain-2 catalytic subunit | CAPN2 | 29 | 53.3 | 0 | 323.3 | 0.98 | 0.95 | 0.92 | 0.99 | 1.08 | 0.98 | 0.91 | 1.10 | 1.02 |
| P17844 | Probable ATP-dependent RNA helicase DDX5 | DDX5 | 16 | 45.6 | 0 | 168.7 | 0.79 | 0.85 | 0.88 | 0.88 | 0.87 | 0.89 | 0.79 | 0.93 | 0.78 |
| P17858 | ATP-dependent 6-phosphofructokinase, liver type | PFKL | 16 | 31.9 | 0 | 182 | 0.90 | 0.86 | 0.97 | 1.01 | 0.88 | 1.02 | 0.88 | 1.01 | 1.10 |
| P17931 | Galectin-3;Galectin | LGALS3 | 8 | 34 | 0 | 29.68 | 0.73 | 0.84 | 0.87 | 0.94 | 0.87 | 0.92 | 1.17 | 0.88 | 0.98 |
| P17987 | T-complex protein 1 subunit | TCP1 | 27 | 53.8 | 0 | 323.3 | 1.18 | 1.07 | 1.08 | 1.05 | 1.05 | 1.04 | 0.82 | 0.90 | 0.97 |
| P18031 | Tyrosine-protein phosphatase non-receptor type 1;Tyrosine-protein phosphatase non-receptor type | PTPN1 | 18 | 55.9 | 0 | 98.77 | 0.73 | 0.93 | 1.61 | 1.18 | 0.96 | 1.04 | 0.83 | 1.77 | 1.00 |
| P18077 | 60S ribosomal protein L35a | RPL35A | 6 | 39.1 | 0 | 31.03 | 0.80 | 0.81 | 0.81 | 0.87 | 0.80 | 0.90 | 0.87 | 0.95 | 0.89 |
| P18085 | ADP-ribosylation factor 4 | ARF4 | 6 | 64.4 | 0 | 250.5 | 0.92 | 1.05 | 0.95 | 0.90 | 0.96 | 1.03 | 0.77 | 0.77 | 0.83 |
| P18124 | 60S ribosomal protein L7 | RPL7 | 16 | 46.4 | 0 | 323.3 | 0.77 | 0.80 | 0.76 | 0.73 | 0.78 | 0.74 | 0.83 | 0.84 | 0.82 |
| P18206 | Vinculin | VCL | 59 | 63.5 | 0 | 323.3 | 1.04 | 1.04 | 1.07 | 1.05 | 1.05 | 1.01 | 1.08 | 1.00 | 1.10 |
| P18669 | Phosphoglycerate mutase 1 | PGAM1 | 17 | 70.9 | 0 | 323.3 | 1.08 | 0.98 | 1.06 | 1.08 | 1.09 | 1.06 | 1.03 | 0.95 | 1.04 |
| P18754 | Regulator of chromosome condensation | RCC1 | 6 | 21.9 | 0 | 20.91 | 0.99 | 0.80 | 0.73 | 0.80 | 0.52 | 0.79 | NaN | 0.68 | 0.81 |
| P18827 | Syndecan-1 | SDC1 | 2 | 10.3 | 0 | 3.499 | 0.81 | NaN | 0.64 | NaN | 0.53 | NaN | NaN | 1.33 | 1.21 |
| P18859 | ATP synthase-coupling factor 6, mitochondrial | ATP5J | 3 | 44.4 | 0 | 168.7 | 1.35 | 1.00 | 1.23 | 1.11 | 1.26 | 1.12 | 1.35 | 1.25 | NaN |
| P19338 | Nucleolin | NCL | 33 | 40 | 0 | 323.3 | 0.96 | 0.96 | 0.94 | 1.00 | 0.96 | 0.97 | 0.87 | 0.82 | 0.84 |
| P19367 | Hexokinase-1 | HK1 | 24 | 34.8 | 0 | 92.56 | 1.08 | NaN | 1.21 | 0.98 | 1.23 | 0.99 | 1.03 | 1.01 | 0.97 |
| P19525 | Interferon-induced, double-stranded RNA-activated protein kinase | EIF2AK2 | 5 | 10 | 0 | 4.583 | NaN | 1.43 | NaN | NaN | 1.11 | 0.99 | NaN | NaN | 0.86 |
| P19623 | Spermidine synthase | SRM | 9 | 50.3 | 0 | 59.04 | 0.76 | 0.96 | 0.95 | 0.90 | 0.74 | 0.77 | 0.81 | 0.86 | 0.98 |
| P20042 | Eukaryotic translation initiation factor 2 subunit 2 | EIF2S2 | 16 | 44.4 | 0 | 166.9 | 1.13 | 1.09 | 0.97 | 0.96 | 1.06 | 1.00 | 1.10 | 0.84 | 0.91 |
| P20073 | Annexin A7 | ANXA7 | 12 | 26.6 | 0 | 20.76 | 0.95 | 1.05 | 0.96 | 1.09 | 1.01 | 0.82 | 1.35 | 1.39 | 1.00 |
| P20290 | Transcription factor BTF3 | BTF3 | 9 | 60.2 | 0 | 200.3 | 0.89 | 0.73 | 0.88 | 0.97 | 1.17 | 0.87 | 1.21 | 1.01 | 0.83 |
| P20339 | Ras-related protein Rab-5A | RAB5A | 4 | 39.5 | 0 | 26.2 | 1.03 | 1.13 | 1.23 | 1.24 | 1.35 | 1.13 | 0.70 | 1.54 | 0.94 |
| P20340 | Ras-related protein Rab-6A | RAB6A | 7 | 37 | 0 | 36.49 | 0.69 | 0.79 | 0.87 | 1.10 | 1.03 | 0.85 | 0.96 | 0.76 | 0.99 |
| P20618 | Proteasome subunit beta type-1 | PSMB1 | 9 | 45.6 | 0 | 178.3 | 0.87 | 0.80 | 0.94 | 0.79 | 0.97 | 1.01 | 1.38 | 0.70 | 0.95 |
| P20700 | Lamin-B1 | LMNB1 | 24 | 52.7 | 0 | 323.3 | 0.98 | 0.86 | 1.12 | 0.90 | 0.99 | 0.90 | 0.97 | 0.89 | 1.04 |
| P20962 | Parathymosin | PTMS | 2 | 11.8 | 0 | 88.24 | 1.07 | 1.18 | 1.12 | 1.04 | 1.23 | 1.01 | NaN | NaN | 1.08 |
| P21266 | Glutathione S-transferase Mu 3 | GSTM3 | 14 | 65.3 | 0 | 56.38 | 1.09 | 1.02 | 0.96 | 0.96 | 0.94 | 0.90 | 1.02 | 1.18 | 0.95 |

| | | | | | | | | | | | | | | | |
|---|---|---|---|---|---|---|---|---|---|---|---|---|---|---|---|
| P21281 | V-type proton ATPase subunit B, brain isoform | ATP6V1B2 | 6 | 17.6 | 0 | 11.61 | NaN | NaN | NaN | NaN | 1.39 | 0.74 | NaN | NaN | NaN |
| P21283 | V-type proton ATPase subunit C | ATP6V1C1 | 3 | 9.2 | 0 | 4.809 | 0.84 | 1.90 | 1.18 | NaN | NaN | 1.34 | 1.10 | NaN | 1.11 |
| P21291 | Cysteine and glycine-rich protein | CSRP1 | 7 | 59.6 | 0 | 294.3 | 0.95 | 0.85 | 0.90 | 1.00 | 0.99 | 1.06 | 0.83 | 1.18 | 0.89 |
| P21333 | Filamin-A | FLNA | 112 | 62.6 | 0 | 323.3 | 1.06 | 1.02 | 1.06 | 0.99 | 0.99 | 0.97 | 1.04 | 1.00 | 1.05 |
| P21399 | Cytoplasmic aconitate hydratase | ACO1 | 16 | 27.1 | 0 | 76.11 | 0.83 | 0.70 | 0.79 | 0.77 | 0.61 | 0.79 | 0.76 | 0.99 | 1.00 |
| P21589 | 5-nucleotidase | NT5E | 21 | 40.4 | 0 | 323.3 | 1.14 | 1.18 | 1.16 | 1.18 | 1.04 | 1.25 | 1.13 | 1.00 | 0.95 |
| P21796 | Voltage-dependent anion-selective channel protein 1 | VDAC1 | 18 | 83.4 | 0 | 323.3 | 1.03 | 1.01 | 1.02 | 1.03 | 0.99 | 1.08 | 1.06 | 0.95 | 1.00 |
| P21964 | Catechol O-methyltransferase | COMT | 9 | 49.1 | 0 | 23.85 | 1.01 | 0.73 | 0.95 | NaN | NaN | 1.22 | NaN | 1.11 | 1.08 |
| P21980 | Protein-glutamine gamma-glutamyltransferase 2 | TGM2 | 32 | 53.6 | 0 | 323.3 | 1.14 | 1.21 | 1.19 | 1.16 | 1.13 | 1.15 | 1.30 | 1.29 | 1.31 |
| P22087 | rRNA 2-O-methyltransferase fibrillarin | FBL | 4 | 44.9 | 0 | 14.99 | 0.57 | 0.99 | 0.90 | 0.78 | 0.85 | 0.93 | 0.65 | 0.75 | 0.61 |
| P22102 | Trifunctional purine biosynthetic protein adenosine-3;Phosphoribosylamine--glycine ligase;Phosphoribosylformylglycinamidine cyclo-ligase;Phosphoribosylglycinamide formyltransferase | GART | 22 | 40.1 | 0 | 303.3 | 1.01 | 0.91 | 0.89 | 0.82 | 0.90 | 0.86 | 0.82 | 0.78 | 0.97 |
| P22234 | Multifunctional protein ADE2;Phosphoribosylaminoimidazole-succinocarboxamide synthase;Phosphoribosylaminoimidazole carboxylase | PAICS | 19 | 51.5 | 0 | 177.3 | 0.83 | 0.89 | 0.81 | 0.94 | 0.95 | 0.98 | 0.90 | 1.04 | 0.94 |
| P22307 | Non-specific lipid-transfer protein | SCP2 | 15 | 24.9 | 0 | 39.27 | 0.76 | 0.70 | 0.78 | 0.94 | 0.90 | 0.94 | 0.71 | 0.70 | 0.75 |
| P22314 | Ubiquitin-like modifier-activating enzyme 1 | UBA1 | 43 | 54.2 | 0 | 323.3 | 1.02 | 1.02 | 1.05 | 0.94 | 0.97 | 0.96 | 1.13 | 1.07 | 1.15 |
| P22413 | Ectonucleotide pyrophosphatase/phosphodiesterase family member 1;Alkaline phosphodiesterase I;Nucleotide pyrophosphatase | ENPP1 | 4 | 6.7 | 0 | 4.262 | NaN | NaN | 1.18 | NaN | 1.10 | 0.73 | NaN | NaN | NaN |
| P22626 | Heterogeneous nuclear ribonucleoproteins A2/B1 | HNRNPA2I | 23 | 69.1 | 0 | 323.3 | 0.92 | 0.91 | 0.91 | 0.91 | 0.92 | 0.94 | 0.79 | 0.92 | 0.94 |
| P22695 | Cytochrome b-c1 complex subunit 2, mitochondrial | UQCRC2 | 8 | 32.9 | 0 | 79.28 | 1.30 | 1.13 | 1.22 | 0.99 | 1.02 | 0.95 | 0.99 | 0.81 | 0.97 |
| P23229 | Integrin alpha-6;Integrin alpha-6 heavy chain;Integrin alpha-6 light chain;Processed integrin alpha-6 | ITGA6 | 10 | 12.4 | 0 | 162.4 | 0.73 | 0.83 | 0.93 | 0.73 | NaN | 0.83 | 0.56 | NaN | 1.54 |
| P23246 | Splicing factor, proline- and glutamine-rich | SFPQ | 23 | 38 | 0 | 206.4 | 1.15 | 1.19 | 1.08 | 1.13 | 1.15 | 1.09 | 1.17 | 1.12 | 0.88 |
| P23284 | Peptidyl-prolyl cis-trans isomerase B | PPIB | 14 | 61.1 | 0 | 232.1 | 1.13 | 1.11 | 1.04 | 0.99 | 1.01 | 1.06 | 0.93 | 1.01 | 0.99 |
| P23381 | Tryptophan--tRNA ligase, cytoplasmic;T1-TrpRS;T2-TrpRS | WARS | 9 | 27.2 | 0 | 86.23 | 0.96 | 0.80 | 0.97 | 0.69 | 0.73 | 0.94 | 0.97 | 0.94 | 0.95 |

| | | | | | | | | | | | | | | | |
|---|---|---|---|---|---|---|---|---|---|---|---|---|---|---|---|
| P23396 | 40S ribosomal protein S3 | RPS3 | 18 | 79.8 | 0 | 146.8 | 0.85 | 0.86 | 0.86 | 0.87 | 0.86 | 0.91 | 0.88 | 0.94 | 0.90 |
| P23526 | Adenosylhomocysteinase | AHCY | 20 | 46.3 | 0 | 323.3 | 0.87 | 0.85 | 0.87 | 0.86 | 0.90 | 0.80 | 0.91 | 0.82 | 0.76 |
| P23528 | Cofilin-1 | CFL1 | 16 | 89.8 | 0 | 323.3 | 0.93 | 0.91 | 0.88 | 0.93 | 0.95 | 1.02 | 1.00 | 0.97 | 0.90 |
| P23634 | Plasma membrane calcium-transporting ATPase 4 | ATP2B4 | 2 | 7.4 | 0 | 15.08 | NaN | 0.67 | 1.06 | 0.99 | 0.52 | 0.71 | 0.88 | NaN | NaN |
| P23919 | Thymidylate kinase | DTYMK | 9 | 38.2 | 0 | 9.826 | 0.97 | 1.05 | 1.25 | 0.91 | 1.39 | 0.96 | 1.23 | 1.26 | 1.11 |
| P24534 | Elongation factor 1-beta | EEF1B2 | 8 | 47.6 | 0 | 323.3 | 1.01 | 1.01 | 0.91 | 1.03 | 1.15 | 1.05 | 0.92 | 0.95 | 1.03 |
| Q5QNZ2 | ATP synthase F(0) complex subunit B1, mitochondrial | ATP5F1 | 6 | 27.7 | 0 | 127.3 | 1.18 | 1.06 | 1.02 | 0.99 | 1.19 | 1.46 | 1.59 | 0.74 | 0.99 |
| P24666 | Low molecular weight phosphotyrosine protein phosphatase | ACP1 | 4 | 41.1 | 0 | 6.582 | 0.78 | NaN | 0.60 | NaN | NaN | NaN | NaN | NaN | NaN |
| P24752 | Acetyl-CoA acetyltransferase, mitochondrial | ACAT1 | 18 | 55.3 | 0 | 264.1 | 0.83 | 0.80 | 0.94 | 0.87 | 0.94 | 1.10 | 0.80 | 0.75 | 0.93 |
| P24844 | Myosin regulatory light polypeptide 9 | MYL9 | 3 | 66.3 | 0 | 19.32 | 1.28 | 1.06 | 1.14 | 1.05 | 0.90 | 1.08 | 1.13 | 1.29 | 1.16 |
| P25205 | DNA replication licensing factor MCM3 | MCM3 | 11 | 15 | 0 | 17.6 | 0.89 | 0.78 | 0.54 | 0.82 | 0.68 | 0.85 | 0.72 | 0.73 | 0.73 |
| P25398 | 40S ribosomal protein S12 | RPS12 | 8 | 71.2 | 0 | 262.6 | 0.95 | 1.01 | 0.99 | 0.89 | 0.97 | 1.01 | 1.01 | 1.06 | 1.00 |
| P25685 | DnaJ homolog subfamily B member 1 | DNAJB1 | 11 | 36.5 | 0 | 106.8 | 1.71 | 1.55 | 1.84 | 1.50 | 1.82 | 1.43 | 1.45 | 1.03 | 1.02 |
| P25705 | ATP synthase subunit alpha, mitochondrial | ATP5A1 | 29 | 61.7 | 0 | 323.3 | 1.06 | 1.06 | 1.09 | 0.97 | 0.93 | 0.97 | 0.95 | 0.93 | 0.93 |
| P25786 | Proteasome subunit alpha type-1 | PSMA1 | 17 | 75.3 | 0 | 153.5 | 1.03 | 0.98 | 1.01 | 0.96 | 1.04 | 0.99 | 1.11 | 1.09 | 1.03 |
| P25788 | Proteasome subunit alpha type-3 | PSMA3 | 11 | 42.7 | 0 | 63.51 | 1.13 | 1.11 | 1.08 | 0.91 | 1.06 | 1.00 | 0.93 | 0.77 | 1.06 |
| P25789 | Proteasome subunit alpha type-4;Proteasome subunit alpha type;Proteasome subunit beta | PSMA4 | 13 | 58.2 | 0 | 306.2 | 1.08 | 1.02 | 1.12 | 1.05 | 0.97 | 1.02 | 0.91 | 0.95 | 0.95 |
| P26006 | Integrin alpha-3;Integrin alpha-3 heavy chain;Integrin alpha-3 light chain | ITGA3 | 18 | 19 | 0 | 174.2 | 0.85 | 1.00 | 0.95 | 0.99 | 0.95 | 0.95 | 0.89 | 0.97 | 0.93 |
| P26038 | Moesin | MSN | 44 | 76.8 | 0 | 323.3 | 0.95 | 0.95 | 0.97 | 0.99 | 1.01 | 1.01 | 0.94 | 0.92 | 0.90 |
| P26196 | Probable ATP-dependent RNA helicase DDX6 | DDX6 | 7 | 25.5 | 0 | 100.3 | 1.05 | 0.99 | 0.99 | 1.20 | 1.06 | 0.89 | 0.72 | 0.79 | 0.83 |
| P26368 | Splicing factor U2AF 65 kDa subunit | U2AF2 | 12 | 42.5 | 0 | 79.94 | 0.93 | 0.93 | 0.80 | 0.96 | 1.09 | 0.89 | 1.10 | 0.98 | 0.92 |
| P26373 | 60S ribosomal protein L13 | RPL13 | 9 | 35.5 | 0 | 150.9 | 0.98 | 0.94 | 0.95 | 0.96 | 0.96 | 0.98 | 1.04 | 0.97 | 1.00 |
| P26447 | Protein S100-A4 | S100A4 | 5 | 36.6 | 0 | 96.57 | 0.76 | 0.93 | 0.86 | 0.83 | 0.84 | 0.87 | 0.80 | 0.86 | 0.81 |
| P26583 | High mobility group protein B2 | HMGB2 | 9 | 28.7 | 0 | 50.5 | 0.93 | 1.09 | 0.82 | 0.81 | 1.09 | 0.74 | 0.72 | 0.76 | 0.67 |
| P26639 | Threonine--tRNA ligase, cytoplasmic | TARS | 32 | 49.8 | 0 | 323.3 | 1.05 | 1.02 | 1.08 | 0.99 | 1.11 | 0.97 | 1.03 | 0.94 | 0.89 |
| P26640 | Valine--tRNA ligase | VARS | 20 | 21.8 | 0 | 86.93 | 0.88 | 0.89 | 0.96 | 0.92 | 0.84 | 0.83 | 1.01 | 0.94 | 0.89 |
| P26641 | Elongation factor 1-gamma | EEF1G | 27 | 61.1 | 0 | 323.3 | 1.03 | 1.07 | 1.05 | 1.01 | 1.00 | 1.03 | 0.96 | 0.94 | 0.93 |
| P27144 | Adenylate kinase 4, | AK4 | 5 | 25.1 | 0 | 13.66 | 1.30 | 1.10 | 1.32 | 1.38 | 1.41 | 1.60 | 1.29 | 1.31 | 1.11 |
| P27348 | 14-3-3 protein theta | YWHAQ | 14 | 59.2 | 0 | 323.3 | 1.02 | 1.02 | 1.00 | 0.96 | 1.02 | 1.00 | 0.91 | 0.97 | 1.00 |
| X1WI28 | 60S ribosomal protein L10 | RPL10 | 3 | 49.5 | 0 | 233.6 | 0.67 | 0.80 | 0.70 | 0.79 | 0.78 | 0.82 | 0.73 | 0.69 | 0.65 |

| | | | | | | | | | | | | | | |
|---|---|---|---|---|---|---|---|---|---|---|---|---|---|---|
| P27658 | Collagen alpha-1(VIII) chain;Vastatin | COL8A1 | 1 | 1.3 | 0.001 | 2.085 | 0.95 | 1.10 | 1.13 | 1.83 | 1.44 | 1.39 | NaN | NaN | NaN |
| P27694 | Replication protein A 70 kDa DNA-binding subunit;Replication protein A 70 kDa DNA-binding subunit, N-terminally processed | RPA1 | 9 | 21.8 | 0 | 15.03 | 0.88 | 0.59 | 0.66 | 1.02 | 0.88 | 0.75 | 0.81 | 1.01 | NaN |
| P27695 | DNA-(apurinic or apyrimidinic site) lyase;DNA-(apurinic or apyrimidinic site) lyase, mitochondrial | APEX1 | 11 | 44.7 | 0 | 87.77 | 1.01 | 0.88 | 0.89 | 0.93 | 0.99 | 0.99 | 0.89 | 0.92 | 0.97 |
| P27701 | CD82 antigen;Tetraspanin | CD82 | 2 | 9.7 | 0 | 3.613 | 0.79 | NaN | 1.32 | NaN | NaN | NaN | 1.80 | 1.08 | NaN |
| P27797 | Calreticulin | CALR | 28 | 71.5 | 0 | 323.3 | 1.07 | 1.03 | 1.07 | 1.04 | 1.09 | 1.06 | 1.09 | 1.14 | 1.09 |
| P27816 | Microtubule-associated protein 4;Microtubule-associated protein | MAP4 | 39 | 49 | 0 | 323.3 | 0.82 | 0.85 | 0.90 | 0.89 | 0.95 | 0.97 | 0.91 | 0.90 | 1.06 |
| P27824 | Calnexin | CANX | 27 | 45.6 | 0 | 323.3 | 1.04 | 1.05 | 1.04 | 1.02 | 1.04 | 1.06 | 1.04 | 0.93 | 1.10 |
| P28066 | Proteasome subunit alpha type-5 | PSMA5 | 12 | 57.3 | 0 | 130.1 | 0.93 | 0.81 | 0.88 | 0.99 | 0.81 | 1.01 | 1.13 | 0.86 | 0.76 |
| P28070 | Proteasome subunit beta type-4 | PSMB4 | 8 | 48.5 | 0 | 132.8 | 0.97 | 1.08 | 1.12 | 1.02 | 0.96 | 1.11 | 0.90 | 1.03 | 1.11 |
| P28072 | Proteasome subunit beta type-6 | PSMB6 | 4 | 16.7 | 0 | 15.63 | 1.18 | 1.22 | 1.20 | 1.22 | 1.10 | 1.01 | 1.14 | 1.28 | 1.05 |
| P28074 | Proteasome subunit beta type-5 | PSMB5 | 7 | 36.5 | 0 | 25.96 | 0.79 | 0.88 | 1.04 | 0.79 | 0.97 | 0.96 | 0.62 | 0.80 | 1.09 |
| P28288 | ATP-binding cassette sub-family D member 3 | ABCD3 | 5 | 10.3 | 0 | 8.774 | 1.26 | 1.15 | 1.19 | 0.84 | 1.01 | 1.17 | 1.03 | 0.84 | 1.23 |
| P28482 | Mitogen-activated protein kinase | MAPK1 | 8 | 38.9 | 0 | 80.56 | 0.99 | 0.84 | 0.82 | 1.11 | 1.05 | 0.89 | 1.20 | 1.13 | 1.09 |
| P28838 | Cytosol aminopeptidase | LAP3 | 16 | 45.1 | 0 | 62.52 | 0.96 | 0.84 | 0.87 | NaN | 0.71 | 1.09 | 1.22 | 0.63 | 0.98 |
| P29317 | Ephrin type-A receptor 2 | EPHA2 | 8 | 11.3 | 0 | 23.66 | 1.35 | 1.37 | 1.47 | 1.29 | 1.04 | 1.20 | NaN | 1.10 | 1.10 |
| P29353 | SHC-transforming protein 1 | SHC1 | 2 | 4.6 | 0 | 3.337 | 0.95 | 1.01 | NaN | NaN | NaN | 1.35 | NaN | NaN | NaN |
| P29401 | Transketolase | TKT | 35 | 68.2 | 0 | 323.3 | 1.01 | 1.01 | 1.02 | 1.03 | 1.09 | 1.02 | 0.89 | 0.89 | 0.92 |
| P29966 | Myristoylated alanine-rich C-kinase substrate | MARCKS | 10 | 50.3 | 0 | 323.3 | 0.77 | 0.69 | 0.68 | 0.71 | 0.68 | 0.79 | 1.20 | 1.23 | 1.19 |
| P29992 | Guanine nucleotide-binding protein subunit alpha-11;Guanine nucleotide-binding protein subunit alpha-14;Guanine nucleotide-binding protein G(q) | GNA11 | 2 | 7.2 | 0 | 5.236 | 0.94 | 0.58 | 0.84 | NaN | 0.91 | 1.16 | NaN | NaN | 0.93 |
| P30040 | Endoplasmic reticulum resident protein 29 | ERP29 | 5 | 21.8 | 0 | 34.05 | 0.96 | 1.04 | 1.02 | 0.91 | 1.04 | 1.05 | 1.08 | 1.00 | 0.97 |
| P30041 | Peroxiredoxin-6 | PRDX6 | 18 | 66.1 | 0 | 323.3 | 1.01 | 0.95 | 1.08 | 1.08 | 1.01 | 1.02 | 1.07 | 1.04 | 0.91 |
| P30044 | Peroxiredoxin-5, mitochondrial | PRDX5 | 9 | 55.1 | 0 | 50.49 | 0.92 | 1.01 | 1.02 | 0.89 | 1.10 | 1.07 | 1.33 | 1.49 | 1.41 |
| P30048 | Thioredoxin-dependent peroxide reductase, mitochondrial | PRDX3 | 9 | 43.4 | 0 | 181.4 | 0.91 | 0.86 | 0.92 | 1.05 | 1.09 | 1.04 | 1.09 | 0.90 | 0.90 |
| P30049 | ATP synthase subunit delta, mitochondrial | ATP5D | 2 | 13.7 | 0 | 6.676 | NaN | NaN | NaN | 0.99 | NaN | NaN | 0.93 | 0.76 | NaN |
| P30050 | 60S ribosomal protein L12 | RPL12 | 8 | 63 | 0 | 299 | 0.77 | 0.76 | 0.77 | 0.73 | 0.75 | 0.82 | 0.88 | 0.88 | 0.86 |
| P30084 | Enoyl-CoA hydratase, mitochondrial | ECHS1 | 8 | 36.6 | 0 | 104.3 | 1.00 | 0.87 | 1.17 | 1.09 | 1.03 | 1.10 | 1.13 | 0.99 | 0.99 |
| P30085 | UMP-CMP kinase | CMPK1 | 7 | 47.4 | 0 | 36.19 | 0.95 | 1.05 | 1.07 | 0.95 | 0.88 | 1.04 | 1.12 | 1.04 | 1.15 |

| | | | | | | | | | | | | | | | |
|---|---|---|---|---|---|---|---|---|---|---|---|---|---|---|---|
| P30086 | Phosphatidylethanolamine-binding protein 1;Hippocampal cholinergic neurostimulating | PEBP1 | 11 | 79.7 | 0 | 323.3 | 0.98 | 1.01 | 0.96 | 0.98 | 1.02 | 1.10 | 0.98 | 1.04 | 0.98 |
| P30101 | Protein disulfide-isomerase A3 | PDIA3 | 29 | 68.3 | 0 | 323.3 | 1.02 | 0.99 | 1.00 | 0.99 | 1.05 | 1.06 | 0.93 | 1.07 | 0.96 |
| P30153 | Serine/threonine-protein phosphatase 2A 65 kDa regulatory subunit A alpha | PPP2R1A | 25 | 54.5 | 0 | 323.3 | 1.14 | 1.16 | 1.20 | 1.29 | 1.23 | 1.23 | 0.96 | 1.06 | 0.89 |
| P30405 | Peptidyl-prolyl cis-trans isomerase F, mitochondrial;Peptidyl-prolyl cis- | PPIF | 5 | 49.8 | 0 | 45.81 | 0.78 | 0.72 | NaN | 1.41 | NaN | 1.38 | NaN | NaN | NaN |
| P30419 | Glycylpeptide N-tetradecanoyltransferase 1 | NMT1 | 9 | 20.8 | 0 | 284.4 | 0.88 | 0.81 | 0.86 | 0.87 | 1.02 | 0.88 | 0.63 | 0.60 | 0.82 |
| P30479 | HLA class I histocompatibility antigen, B-41 alpha chain;HLA class I histocompatibility antigen, B-42 alpha chain;HLA class I histocompatibility antigen, B-8 alpha chain;HLA class I histocompatibility antigen, B-18 alpha chain | HLA-B | 1 | 37.3 | 0 | 246 | 1.07 | 1.02 | 1.03 | 1.14 | 1.15 | 1.13 | 1.02 | 1.10 | 1.04 |
| P30520 | Adenylosuccinate synthetase isozyme 2 | ADSS | 12 | 37.3 | 0 | 60.86 | 0.81 | 0.84 | 0.87 | 0.99 | 0.94 | 1.04 | 1.09 | 0.87 | 0.91 |
| P30740 | Leukocyte elastase inhibitor | SERPINB1 | 15 | 51.2 | 0 | 70.14 | 0.81 | 0.85 | 0.86 | 1.01 | 0.88 | 0.92 | 0.82 | 0.94 | 0.83 |
| P30825 | High affinity cationic amino acid transporter 1 | SLC7A1 | 1 | 3 | 0 | 4.961 | 1.07 | 0.93 | 1.22 | NaN | NaN | 0.88 | NaN | NaN | 0.89 |
| P30837 | Aldehyde dehydrogenase X, mitochondrial | ALDH1B1 | 8 | 23.4 | 0 | 16.85 | 0.86 | 0.92 | 0.73 | 0.81 | 0.81 | 0.88 | 0.71 | 0.86 | 0.95 |
| P31150 | Rab GDP dissociation inhibitor alpha | GDI1 | 12 | 60.4 | 0 | 190.1 | 0.86 | 0.76 | 0.70 | 1.40 | 1.01 | 1.07 | 0.80 | 1.65 | 0.94 |
| P31153 | S-adenosylmethionine synthase isoform type-2 | MAT2A | 8 | 22.8 | 0 | 27.98 | 1.23 | 0.88 | 1.05 | 1.20 | 0.93 | 0.97 | 0.91 | 1.20 | 0.94 |
| P31689 | DnaJ homolog subfamily A member 1 | DNAJA1 | 9 | 39.8 | 0 | 43.53 | 1.87 | 1.98 | 1.94 | 1.59 | 1.50 | 1.56 | 0.81 | 0.72 | 0.93 |
| P31930 | Cytochrome b-c1 complex subunit 1, mitochondrial | UQCRC1 | 10 | 34.8 | 0 | 29.99 | 1.21 | 1.06 | 1.22 | 1.14 | 0.94 | 1.20 | NaN | 1.41 | 0.99 |
| P31937 | 3-hydroxyisobutyrate dehydrogenase, mitochondrial | HIBADH | 8 | 40.5 | 0 | 48.33 | 0.95 | 0.70 | 0.82 | NaN | 0.96 | 1.09 | 1.11 | NaN | 0.57 |
| P31939 | Bifunctional purine biosynthesis protein PURH;Phosphoribosylaminoimidazolecarboxamide formyltransferase;IMP cyclohydrolase | ATIC | 21 | 50.3 | 0 | 210 | 0.88 | 0.80 | 0.85 | 0.81 | 0.95 | 0.81 | 0.76 | 0.76 | 0.86 |
| P31942 | Heterogeneous nuclear ribonucleoprotein H3 | HNRNPH3 | 7 | 34.7 | 0 | 233.9 | 0.85 | 0.77 | 0.87 | 0.85 | 0.88 | 0.91 | 0.95 | 0.97 | 1.07 |

| | | | | | | | | | | | | | | |
|---|---|---|---|---|---|---|---|---|---|---|---|---|---|---|
| P31946 | 14-3-3 protein beta/alpha;14-3-3 protein beta/alpha, N-terminally processed | YWHAB | 9 | 52.8 | 0 | 323.3 | 0.97 | 0.95 | 0.97 | 1.00 | 0.91 | 0.97 | 1.01 | 0.99 | 1.00 |
| P31947 | 14-3-3 protein sigma | SFN | 11 | 61.3 | 0 | 323.3 | 0.91 | 0.88 | 0.90 | 0.92 | 0.96 | 0.98 | 0.95 | 0.98 | 1.11 |
| P31948 | Stress-induced-phosphoprotein 1 | STIP1 | 44 | 65 | 0 | 323.3 | 1.08 | 1.12 | 1.18 | 1.29 | 1.32 | 1.16 | 0.86 | 0.92 | 0.90 |
| P31949 | Protein S100-A11;Protein S100-A11, N-terminally processed | S100A11 | 6 | 52.4 | 0 | 283.2 | 1.02 | 0.95 | 1.01 | 0.92 | 1.01 | 0.91 | 0.92 | 0.96 | 1.06 |
| P32119 | Peroxiredoxin-2 | PRDX2 | 12 | 60.6 | 0 | 182.6 | 0.97 | 1.00 | 1.01 | 0.94 | 1.02 | 0.93 | 1.01 | 0.96 | 1.07 |
| P32320 | Cytidine deaminase | CDA | 4 | 45.2 | 0 | 123 | 0.97 | 0.85 | 0.67 | 1.06 | 1.13 | 1.10 | 1.77 | 1.60 | 1.14 |
| P32321 | Deoxycytidylate deaminase | DCTD | 2 | 18.5 | 0 | 4.252 | NaN | NaN | 1.30 | NaN | 1.33 | 0.71 | NaN | NaN | 0.77 |
| P32969 | 60S ribosomal protein L9 | RPL9 | 8 | 65.1 | 0 | 323.3 | 0.79 | 0.71 | 0.79 | 0.69 | 0.73 | 0.68 | 0.80 | 0.73 | 0.87 |
| P33176 | Kinesin-1 heavy chain | KIF5B | 38 | 49.1 | 0 | 323.3 | 0.89 | 0.94 | 0.72 | 0.95 | 0.87 | 0.85 | 0.92 | 0.85 | 0.81 |
| P33993 | DNA replication licensing factor MCM7 | MCM7 | 15 | 28.1 | 0 | 47.29 | 0.90 | 0.54 | 0.69 | 0.96 | 0.96 | 0.74 | NaN | NaN | 0.94 |
| P34897 | Serine hydroxymethyltransferase, mitochondrial;Serine hydroxymethyltransferase | SHMT2 | 15 | 42.5 | 0 | 152.3 | 0.80 | 0.66 | 0.74 | 0.87 | 0.85 | 0.88 | NaN | 0.87 | 1.00 |
| P34932 | Heat shock 70 kDa protein 4 | HSPA4 | 39 | 56.7 | 0 | 323.3 | 1.10 | 1.17 | 1.11 | 1.20 | 1.15 | 1.07 | 1.02 | 0.94 | 0.96 |
| P35221 | Catenin alpha-1 | CTNNA1 | 13 | 23.5 | 0 | 23.62 | 1.46 | 1.53 | 1.25 | 0.64 | 0.83 | 0.75 | NaN | NaN | NaN |
| P35232 | Prohibitin | PHB | 16 | 72.8 | 0 | 189.3 | 1.12 | 0.97 | 1.09 | 1.04 | 1.02 | 1.09 | 1.11 | 0.96 | 1.12 |
| P35268 | 60S ribosomal protein L22 | RPL22 | 4 | 39.8 | 0 | 231.1 | 0.93 | 0.78 | 0.81 | 0.74 | 0.66 | 0.98 | 0.98 | 0.70 | 0.65 |
| P35269 | General transcription factor IIF subunit 1 | GTF2F1 | 5 | 13.5 | 0 | 22.42 | 0.76 | 1.18 | 0.95 | 0.71 | 1.01 | 0.97 | NaN | 1.21 | 0.98 |
| P35270 | Sepiapterin reductase | SPR | 10 | 51 | 0 | 88.06 | 0.84 | 0.96 | 0.97 | 1.10 | 1.09 | 0.94 | 0.92 | 0.88 | 1.06 |
| P35579 | Myosin-9 | MYH9 | 147 | 69.3 | 0 | 323.3 | 0.90 | 0.94 | 0.91 | 0.94 | 0.97 | 0.96 | 0.95 | 0.95 | 0.99 |
| P35580 | Myosin-10 | MYH10 | 15 | 21.9 | 0 | 62.77 | 0.92 | 0.58 | 0.58 | 0.89 | 1.10 | 0.52 | NaN | 0.73 | NaN |
| P35606 | Coatomer subunit beta | COPB2 | 23 | 35 | 0 | 136 | 0.92 | 0.87 | 0.90 | 1.15 | 0.90 | 0.92 | 1.01 | 0.78 | 1.22 |
| P35610 | Sterol O-acyltransferase 1 | SOAT1 | 5 | 11.3 | 0 | 11.41 | 1.17 | 1.14 | 1.14 | 0.94 | 1.38 | 1.17 | NaN | NaN | 1.12 |
| P35613 | Basigin | BSG | 9 | 25.7 | 0 | 148.2 | 0.95 | 1.07 | 1.20 | 0.97 | 1.01 | 0.98 | 0.62 | 0.64 | 1.06 |
| P35637 | RNA-binding protein FUS | FUS | 7 | 20 | 0 | 141.5 | 0.82 | 0.70 | 0.85 | 0.77 | 0.83 | 0.77 | 0.60 | 0.72 | 0.69 |
| P35998 | 26S protease regulatory subunit | PSMC2 | 22 | 61.7 | 0 | 323.3 | 1.10 | 1.08 | 1.05 | 1.09 | 1.01 | 1.02 | 1.02 | 1.07 | 1.04 |
| P36405 | ADP-ribosylation factor-like protein 3 | ARL3 | 7 | 47.8 | 0 | 25.45 | 0.89 | 1.13 | 0.85 | 1.02 | 1.07 | 0.92 | 0.99 | 0.99 | 1.10 |
| P36406 | E3 ubiquitin-protein ligase | TRIM23 | 1 | 1.7 | 0 | 3.773 | 0.62 | 0.61 | 0.71 | NaN | 0.92 | 0.73 | NaN | NaN | 0.50 |
| P36507 | Dual specificity mitogen-activated protein kinase kinase 2 | MAP2K2 | 3 | 19.2 | 0 | 36.46 | 0.93 | 1.17 | 0.94 | 0.90 | 0.89 | 0.94 | 1.16 | 1.03 | 1.03 |
| P36542 | ATP synthase subunit gamma, mitochondrial | ATP5C1 | 6 | 21.8 | 0 | 5.852 | 0.93 | 0.84 | 0.85 | NaN | 1.29 | 1.11 | 0.85 | 1.35 | 0.68 |
| P36543 | V-type proton ATPase subunit E 1;V-type proton ATPase subunit E 2 | ATP6V1E1 | 2 | 10.2 | 0 | 6.45 | NaN | NaN | 1.50 | NaN | 0.63 | 0.86 | NaN | NaN | 1.14 |
| P36551 | Oxygen-dependent coproporphyrinogen-III oxidase, mitochondrial | CPOX | 13 | 36.3 | 0 | 141.9 | 1.13 | 1.16 | 1.03 | 1.04 | 1.01 | 1.06 | 0.97 | 1.08 | 1.18 |
| P36578 | 60S ribosomal protein L4 | RPL4 | 18 | 48.5 | 0 | 182.8 | 0.82 | 0.86 | 0.78 | 0.92 | 0.83 | 0.90 | 0.77 | 0.76 | 0.82 |
| P36871 | Phosphoglucomutase-1 | PGM1 | 22 | 47.7 | 0 | 305.2 | 1.12 | 0.96 | 1.10 | 1.04 | 1.09 | 1.11 | 1.03 | 1.16 | 1.16 |

| | | | | | | | | | | | | | | | |
|---|---|---|---|---|---|---|---|---|---|---|---|---|---|---|---|
| P36957 | Dihydrolipoyllysine-residue succinyltransferase component of 2-oxoglutarate dehydrogenase complex, mitochondrial | DLST | 8 | 18.8 | 0 | 16.23 | 0.94 | 0.99 | 0.94 | 1.10 | 0.96 | 1.03 | 1.07 | 0.86 | 0.83 |
| P37108 | Signal recognition particle 14 kDa protein | SRP14 | 4 | 47.1 | 0 | 31.51 | 0.92 | 1.00 | 0.86 | 0.63 | 0.74 | 0.85 | 0.93 | 0.92 | 1.01 |
| P37235 | Hippocalcin-like protein 1;Neuron-specific calcium-binding protein hippocalcin | HPCAL1 | 12 | 59.6 | 0 | 193.9 | 1.23 | 1.20 | 1.22 | 1.32 | 1.41 | 1.42 | 0.99 | 1.04 | 1.24 |
| X6RJP6 | Transgelin-2 | TAGLN2 | 16 | 77.5 | 0 | 323.3 | 1.02 | 1.06 | 1.02 | 0.96 | 0.97 | 0.98 | 1.30 | 1.13 | 1.39 |
| P37837 | Transaldolase | TALDO1 | 22 | 52.8 | 0 | 183 | 1.15 | 1.12 | 1.13 | 1.18 | 1.22 | 1.23 | 0.95 | 1.01 | 1.01 |
| P38117 | Electron transfer flavoprotein subunit beta | ETFB | 7 | 32.9 | 0 | 6.24 | 1.08 | 0.96 | 0.93 | 1.48 | 1.25 | 1.02 | NaN | 0.86 | NaN |
| P38159 | RNA-binding motif protein, X chromosome;RNA-binding motif protein, X chromosome, N-terminally processed;RNA binding motif protein, X-linked- | RBMX | 17 | 40.4 | 0 | 98.15 | 0.72 | 0.75 | 0.82 | 0.68 | 0.72 | 0.85 | 0.65 | 0.70 | 0.74 |
| P38606 | V-type proton ATPase catalytic subunit A | ATP6V1A | 10 | 24.6 | 0 | 204.4 | 0.88 | 0.94 | 0.96 | 1.20 | 0.88 | 0.97 | 1.10 | 1.29 | 1.15 |
| P38646 | Stress-70 protein, mitochondrial | HSPA9 | 41 | 58.9 | 0 | 323.3 | 0.92 | 1.03 | 0.97 | 1.07 | 1.13 | 1.09 | 0.93 | 0.87 | 0.89 |
| P38919 | Eukaryotic initiation factor 4A-III;Eukaryotic initiation factor 4A-III, N-terminally processed | EIF4A3 | 12 | 44 | 0 | 66.13 | 0.80 | 0.92 | 0.80 | 0.98 | 0.82 | 0.95 | 0.85 | 1.00 | 0.93 |
| P39019 | 40S ribosomal protein S19 | RPS19 | 12 | 67.6 | 0 | 53.31 | 0.86 | 0.79 | 0.78 | 0.79 | 0.81 | 0.80 | 0.77 | 0.75 | 0.80 |
| P39023 | 60S ribosomal protein L3 | RPL3 | 19 | 47.6 | 0 | 180.1 | 0.79 | 0.77 | 0.80 | 0.88 | 0.83 | 0.78 | 0.79 | 0.84 | 0.77 |
| P39687 | Acidic leucine-rich nuclear phosphoprotein 32 family member A | ANP32A | 6 | 34.1 | 0 | 216 | 1.16 | 1.08 | 1.02 | 0.87 | 0.89 | 1.11 | 1.21 | 1.17 | 0.92 |
| P39748 | Flap endonuclease 1 | FEN1 | 9 | 36.8 | 0 | 323.3 | 0.74 | 0.74 | 0.81 | 0.93 | 0.81 | 0.77 | 0.85 | 0.85 | 0.91 |
| P40121 | Macrophage-capping protein | CAPG | 16 | 56.6 | 0 | 323.3 | 0.93 | 0.97 | 1.01 | 0.90 | 0.94 | 1.04 | 1.07 | 0.80 | 0.98 |
| P40227 | T-complex protein 1 subunit zeta | CCT6A | 22 | 56.9 | 0 | 274 | 0.97 | 0.93 | 1.03 | 1.02 | 0.95 | 1.00 | 0.95 | 0.87 | 0.82 |
| P40306 | Proteasome subunit beta type-10 | PSMB10 | 2 | 11 | 0 | 34.47 | 0.70 | 0.61 | 0.81 | NaN | NaN | 1.07 | NaN | NaN | 0.88 |
| P40429 | 60S ribosomal protein L13a | RPL13A | 11 | 44.8 | 0 | 28.09 | 0.83 | 0.82 | 0.79 | 0.79 | 0.80 | 0.88 | 0.88 | 0.77 | 0.82 |
| P40616 | ADP-ribosylation factor-like protein 1 | ARL1 | 4 | 35.4 | 0 | 4.354 | 0.71 | 0.93 | 1.06 | 0.89 | 0.94 | 1.07 | 0.87 | 0.74 | 0.92 |
| P40763 | Signal transducer and activator of transcription 3;Signal transducer and activator of transcription | STAT3 | 12 | 21.4 | 0 | 192.8 | NaN | 0.78 | 0.74 | 0.95 | 0.92 | 1.15 | NaN | NaN | 0.74 |
| P40925 | Malate dehydrogenase, cytoplasmic;Malate dehydrogenase | MDH1 | 15 | 46.4 | 0 | 323.3 | 0.88 | 0.94 | 0.86 | 0.92 | 1.01 | 0.99 | 0.94 | 0.96 | 0.97 |
| P40926 | Malate dehydrogenase, mitochondrial;Malate dehydrogenase | MDH2 | 18 | 64.8 | 0 | 323.3 | 1.00 | 1.00 | 0.95 | 1.03 | 1.04 | 1.07 | 0.85 | 0.88 | 0.89 |

| | | | | | | | | | | | | | | | | |
|---|---|---|---|---|---|---|---|---|---|---|---|---|---|---|---|---|
| P41091 | Eukaryotic translation initiation factor 2 subunit 3;Putative eukaryotic translation initiation factor 2 subunit 3-like protein | EIF2S3 | 21 | 52.3 | 0 | 323.3 | 0.88 | 0.96 | 0.94 | 0.96 | 0.93 | 0.87 | 0.88 | 0.99 | 0.75 |
| P41219 | Peripherin | PRPH | 1 | 9.1 | 0 | 17.17 | 1.19 | 1.23 | 1.10 | NaN | NaN | 1.00 | NaN | NaN | 1.15 |
| P41227 | N-alpha-acetyltransferase 10 | NAA10 | 6 | 32.3 | 0 | 47.69 | 1.06 | 1.15 | 1.09 | 1.22 | 1.16 | 0.97 | 0.96 | 1.14 | 1.26 |
| P41250 | Glycine--tRNA ligase | GARS | 18 | 34.9 | 0 | 176.7 | 1.07 | 0.92 | 1.05 | 1.02 | 0.83 | 0.95 | 0.82 | 1.21 | 0.86 |
| P41252 | Isoleucine--tRNA ligase, cytoplasmic | IARS | 27 | 29 | 0 | 87.13 | 0.98 | 1.00 | 0.94 | 0.80 | 0.97 | 0.97 | 1.09 | 0.94 | 0.89 |
| P42126 | Enoyl-CoA delta isomerase 1, mitochondrial | ECI1 | 4 | 20.5 | 0 | 11.94 | 0.82 | 0.87 | NaN | NaN | NaN | 1.14 | NaN | NaN | NaN |
| P42166 | Lamina-associated polypeptide 2, isoform alpha;Thymopoietin;Thymopentin | TMPO | 7 | 28.8 | 0 | 21.56 | NaN | NaN | NaN | NaN | 0.88 | 0.61 | 0.82 | 1.52 | NaN |
| P42167 | Lamina-associated polypeptide 2, isoforms beta/gamma;Thymopoietin;Thymopentin | TMPO | 7 | 39.6 | 0 | 287.4 | 1.03 | 1.06 | 1.12 | 1.01 | 1.00 | 1.03 | 1.01 | 1.03 | 0.98 |
| P42224 | Signal transducer and activator of transcription 1-alpha/beta;Signal transducer and activator of transcription | STAT1 | 11 | 18.3 | 0 | 26.33 | NaN | 0.68 | NaN | 0.89 | 0.40 | 0.24 | 1.68 | NaN | 0.48 |
| P42677 | 40S ribosomal protein S27 | RPS27 | 2 | 39.3 | 0 | 23.07 | 0.90 | 0.97 | 0.85 | 0.99 | 0.77 | 0.85 | 1.14 | 1.04 | 0.93 |
| P42704 | Leucine-rich PPR motif-containing protein, mitochondrial | LRPPRC | 51 | 49.1 | 0 | 323.3 | 0.85 | 0.86 | 0.75 | 0.83 | 0.92 | 0.90 | 1.11 | 0.85 | 0.86 |
| P42765 | 3-ketoacyl-CoA thiolase, mitochondrial | ACAA2 | 6 | 23.4 | 0 | 47.21 | 3.66 | 4.52 | NaN | NaN | NaN | 1.07 | 1.41 | 1.29 | NaN |
| P42766 | 60S ribosomal protein L35 | RPL35 | 3 | 26 | 0 | 8.24 | 0.99 | 1.00 | 0.96 | 1.02 | 1.09 | 0.97 | 1.02 | 0.89 | 0.90 |
| P43034 | Platelet-activating factor acetylhydrolase IB subunit alpha | PAFAH1B1 | 9 | 27.6 | 0 | 35.14 | 0.99 | 1.02 | 0.72 | NaN | 1.56 | 0.81 | NaN | NaN | 0.85 |
| P43304 | Glycerol-3-phosphate dehydrogenase, mitochondrial;Glycerol-3-phosphate dehydrogenase | GPD2 | 4 | 8 | 0 | 14.55 | NaN | NaN | 0.84 | 0.70 | 0.92 | 1.14 | 0.63 | 0.63 | 0.72 |
| P43487 | Ran-specific GTPase-activating protein | RANBP1 | 7 | 54.7 | 0 | 177.2 | 0.96 | 1.12 | 1.09 | 1.06 | 1.02 | 0.91 | 1.21 | 1.12 | 1.03 |
| P43490 | Nicotinamide phosphoribosyltransferase | NAMPT | 27 | 71.5 | 0 | 323.3 | 1.14 | 1.26 | 1.12 | 1.23 | 1.20 | 1.27 | 0.96 | 0.93 | 0.97 |
| P43686 | 26S protease regulatory subunit 6B | PSMC4 | 14 | 45.7 | 0 | 137.4 | 1.01 | 1.06 | 0.91 | 1.00 | 1.25 | 0.99 | 1.34 | 1.00 | 0.91 |
| P45974 | Ubiquitin carboxyl-terminal hydrolase 5 | USP5 | 15 | 30.9 | 0 | 134 | 0.91 | 1.09 | 1.13 | 1.14 | 0.86 | 1.05 | 0.75 | 0.53 | 1.06 |
| P46013 | Antigen KI-67 | MKI67 | 4 | 1.7 | 0.001 | 2.654 | NaN | NaN | NaN | 1.99 | NaN | 0.90 | NaN | 0.80 | NaN |
| P46060 | Ran GTPase-activating protein 1 | RANGAP1 | 15 | 32.5 | 0 | 105.5 | 0.78 | 1.06 | 1.33 | 0.82 | 0.85 | 0.93 | 0.93 | 0.74 | 0.74 |
| P46108 | Adapter molecule crk | CRK | 6 | 26 | 0 | 22.05 | 1.76 | 1.91 | NaN | NaN | 1.18 | 1.33 | NaN | NaN | 1.06 |
| P46777 | 60S ribosomal protein L5 | RPL5 | 12 | 42.4 | 0 | 61.84 | 0.93 | 0.89 | 0.87 | 0.96 | 0.90 | 0.99 | 0.94 | 0.91 | 0.92 |
| P46778 | 60S ribosomal protein L21 | RPL21 | 5 | 31.2 | 0 | 73.61 | 0.79 | 0.75 | 0.73 | 0.82 | 0.73 | 0.89 | 0.72 | 0.81 | 0.86 |

| | | | | | | | | | | | | | | | |
|---|---|---|---|---|---|---|---|---|---|---|---|---|---|---|---|
| P46779 | 60S ribosomal protein L28 | RPL28 | 6 | 32.8 | 0 | 73.49 | 0.68 | 0.76 | 0.66 | 0.85 | 0.78 | 0.92 | 0.92 | 0.98 | 0.69 |
| P46783 | 40S ribosomal protein S10;Putative 40S ribosomal protein S10-like | RPS10 | 12 | 58.8 | 0 | 24.65 | 1.02 | 0.96 | 0.96 | 0.94 | 0.92 | 0.95 | 1.05 | 1.10 | 0.98 |
| P46821 | Microtubule-associated protein 1B;MAP1B heavy chain;MAP1 light chain LC1 | MAP1B | 24 | 16.1 | 0 | 221.9 | 0.72 | 0.79 | 0.88 | 0.60 | 0.80 | 0.78 | 0.85 | 0.83 | 0.92 |
| P46939 | Utrophin | UTRN | 7 | 3.5 | 0 | 11.54 | NaN | NaN | NaN | NaN | 0.59 | 2.09 | NaN | NaN | NaN |
| P46940 | Ras GTPase-activating-like protein IQGAP1 | IQGAP1 | 67 | 56 | 0 | 323.3 | 0.84 | 0.78 | 0.83 | 0.88 | 0.92 | 0.92 | 0.94 | 0.95 | 0.92 |
| P46977 | Dolichyl-diphosphooligosaccharide--protein glycosyltransferase subunit STT3A | STT3A | 4 | 7.7 | 0 | 6.068 | NaN | 1.18 | NaN | 0.75 | 1.15 | 1.05 | NaN | 0.82 | NaN |
| P47755 | F-actin-capping protein subunit alpha-2 | CAPZA2 | 5 | 43.4 | 0 | 55.95 | 1.10 | 1.03 | 1.37 | 1.42 | 1.08 | 1.08 | 1.17 | 1.11 | 1.45 |
| P47813 | Eukaryotic translation initiation factor 1A, X-chromosomal;Eukaryotic translation initiation factor 1A, Y-chromosomal | EIF1AX | 5 | 35.4 | 0 | 5.237 | 0.75 | NaN | 1.36 | 1.20 | NaN | 0.87 | NaN | NaN | NaN |
| P47897 | Glutamine--tRNA ligase | QARS | 5 | 36.1 | 0 | 34.15 | 0.72 | 0.81 | 0.73 | 1.17 | 0.95 | 0.91 | 0.83 | NaN | 0.92 |
| P47985 | Cytochrome b-c1 complex subunit Rieske, mitochondrial;Cytochrome b-c1 complex subunit 11;Putative cytochrome b-c1 complex subunit | UQCRFS1 | 2 | 8 | 0 | 3.381 | 0.77 | 1.31 | NaN | 1.26 | NaN | 0.77 | NaN | NaN | NaN |
| P48047 | ATP synthase subunit O, mitochondrial | ATP5O | 8 | 48.4 | 0 | 106.6 | 1.12 | 1.08 | 1.34 | 1.03 | 1.04 | 1.02 | 0.77 | 0.84 | 0.88 |
| P48059 | LIM and senescent cell antigen-like-containing domain protein 1 | LIMS1 | 2 | 13.8 | 0 | 11.98 | 1.04 | 1.55 | 1.08 | 1.38 | NaN | 0.96 | 0.93 | NaN | 1.04 |
| P48147 | Prolyl endopeptidase | PREP | 7 | 12.8 | 0 | 15.33 | 1.52 | 1.01 | 1.29 | 1.17 | 0.79 | 1.23 | 0.78 | 1.32 | 1.39 |
| P48449 | Lanosterol synthase | LSS | 4 | 6.3 | 0 | 2.993 | NaN | 1.13 | 1.11 | NaN | 2.28 | NaN | NaN | NaN | NaN |
| P48507 | Glutamate--cysteine ligase regulatory subunit | GCLM | 4 | 20.8 | 0 | 52.89 | 1.81 | 1.82 | 1.75 | 1.34 | 2.10 | 1.18 | NaN | NaN | 1.15 |
| R4GMR5 | 26S proteasome non-ATPase regulatory subunit 8 | PSMD8 | 8 | 35.9 | 0 | 8.603 | 1.04 | 0.91 | 1.28 | 1.35 | 0.92 | 1.14 | 1.07 | 0.92 | 1.15 |
| P48643 | T-complex protein 1 subunit epsilon | CCT5 | 30 | 60.3 | 0 | 323.3 | 1.00 | 1.07 | 1.07 | 0.88 | 1.09 | 1.03 | 0.59 | 0.64 | 0.95 |
| P48735 | Isocitrate dehydrogenase [NADP], mitochondrial | IDH2 | 6 | 15.5 | 0 | 9.779 | 0.73 | 0.85 | 0.93 | 1.23 | 1.14 | 0.99 | NaN | NaN | 1.09 |
| P48960 | CD97 antigen;CD97 antigen subunit alpha;CD97 antigen subunit beta | CD97 | 10 | 19.5 | 0 | 138 | 1.11 | 1.14 | 1.04 | 0.90 | 0.97 | 1.08 | 0.72 | 0.74 | 0.93 |
| P49189 | 4-trimethylaminobutyraldehyde dehydrogenase | ALDH9A1 | 10 | 23.1 | 0 | 49.82 | 0.65 | 0.64 | 0.90 | NaN | 0.87 | 0.73 | 0.87 | 0.90 | 0.99 |
| P49207 | 60S ribosomal protein L34 | RPL34 | 4 | 29.9 | 0 | 7.227 | 1.12 | 1.10 | 0.98 | 0.83 | 0.99 | 1.01 | 0.73 | 0.85 | 0.91 |

| | | | | | | | | | | | | | | | |
|---|---|---|---|---|---|---|---|---|---|---|---|---|---|---|---|
| P49257 | Protein ERGIC-53 | LMAN1 | 10 | 31.8 | 0 | 52.31 | 1.21 | 1.11 | 1.13 | 0.87 | 1.24 | 1.21 | 0.71 | 1.20 | 0.94 |
| P49321 | Nuclear autoantigenic sperm protein | NASP | 13 | 21.1 | 0 | 53.19 | 1.19 | 0.95 | 1.10 | 0.91 | 1.05 | 1.12 | 0.92 | 1.16 | 0.74 |
| P49327 | Fatty acid synthase;[Acyl-carrier-protein] S-acetyltransferase;[Acyl-carrier-protein] S-malonyltransferase;3-oxoacyl-[acyl-carrier-protein] synthase;3-oxoacyl-[acyl-carrier-protein] reductase;3-hydroxyacyl-[acyl-carrier-protein] dehydratase;Enoyl-[acyl-carrier-protein] reductase;Oleoyl-[acyl- | FASN | 88 | 48.1 | 0 | 323.3 | 0.62 | 0.58 | 0.52 | 0.67 | 0.67 | 0.67 | 0.81 | 0.78 | 0.84 |
| Q5J8M5 | Deoxyhypusine synthase | DHPS | 2 | 9 | 0.009 | 1.409 | NaN | 0.75 | NaN | NaN | 1.00 | 0.61 | 0.79 | 0.83 | NaN |
| P49368 | T-complex protein 1 subunit gamma | CCT3 | 33 | 66.8 | 0 | 290.2 | 1.01 | 0.99 | 1.05 | 0.92 | 1.06 | 0.94 | 0.88 | 0.89 | 0.88 |
| P49411 | Elongation factor Tu, | TUFM | 21 | 54.6 | 0 | 323.3 | 1.17 | 1.08 | 1.08 | 1.08 | 1.02 | 1.07 | 0.99 | 0.99 | 1.05 |
| P49458 | Signal recognition particle 9 kDa protein | SRP9 | 3 | 32.6 | 0 | 6.256 | 0.81 | 0.91 | 0.81 | 1.00 | 1.32 | 0.91 | 1.00 | 1.07 | 0.91 |
| P49588 | Alanine--tRNA ligase, | AARS | 18 | 25.9 | 0 | 81.5 | 0.68 | 0.59 | 0.94 | 0.83 | 1.06 | 0.86 | 1.02 | 1.14 | 0.90 |
| Q5T5C7 | Serine--tRNA ligase, cytoplasmic | SARS | 12 | 30.6 | 0 | 42.16 | 0.71 | 0.82 | 0.52 | 0.63 | 0.79 | 0.79 | 0.92 | 0.95 | 0.96 |
| P49593 | Protein phosphatase 1F | PPM1F | 5 | 20.7 | 0 | 27.08 | NaN | 1.10 | 1.28 | NaN | NaN | 0.73 | NaN | NaN | 1.50 |
| P49720 | Proteasome subunit beta type-3 | PSMB3 | 8 | 46.8 | 0 | 143.2 | 0.88 | 0.92 | 0.95 | 1.10 | 1.13 | 0.92 | 0.98 | 1.07 | 0.59 |
| P49721 | Proteasome subunit beta type-2 | PSMB2 | 10 | 50.2 | 0 | 28.24 | 0.91 | 0.97 | 1.01 | 0.90 | 0.98 | 1.09 | 0.85 | 0.70 | 0.87 |
| P49748 | Very long-chain specific acyl-CoA dehydrogenase, mitochondrial | ACADVL | 23 | 47.3 | 0 | 323.3 | 1.12 | 1.07 | 1.18 | 1.15 | 1.18 | 1.15 | 1.32 | 1.24 | 1.20 |
| P49755 | Transmembrane emp24 domain-containing protein 10 | TMED10 | 4 | 19.6 | 0 | 16.78 | 0.87 | 0.73 | 0.74 | 1.19 | 1.07 | 0.99 | 0.97 | 0.95 | 0.51 |
| P49756 | RNA-binding protein 25 | RBM25 | 3 | 5.6 | 0 | 10.3 | 1.05 | 1.31 | 0.81 | 1.23 | NaN | 1.40 | NaN | 1.00 | 1.24 |
| P49773 | Histidine triad nucleotide-binding protein 1 | HINT1 | 6 | 78.6 | 0 | 187.4 | 1.33 | 1.30 | 1.31 | 1.09 | 1.28 | 1.26 | 1.12 | 1.16 | 1.08 |
| P49792 | E3 SUMO-protein ligase RanBP2 | RANBP2 | 4 | 2.1 | 0 | 4.926 | 1.06 | NaN | NaN | NaN | NaN | NaN | 1.11 | NaN | 0.75 |
| P49915 | GMP synthase [glutamine-hydrolyzing] | GMPS | 13 | 30 | 0 | 33.75 | 0.80 | 0.94 | 0.79 | NaN | 0.74 | 1.01 | NaN | 0.91 | 0.96 |
| P50213 | Isocitrate dehydrogenase [NAD] subunit alpha, mitochondrial | IDH3A | 4 | 16.1 | 0 | 5.695 | NaN | 1.34 | 1.17 | 1.20 | 1.10 | 1.36 | NaN | NaN | 1.05 |
| P50395 | Rab GDP dissociation inhibitor beta | GDI2 | 21 | 72.4 | 0 | 323.3 | 0.96 | 0.96 | 0.98 | 0.98 | 1.00 | 1.01 | 0.95 | 1.02 | 1.00 |
| P50402 | Emerin | EMD | 6 | 31.1 | 0 | 66.91 | NaN | 1.31 | 1.25 | NaN | 1.12 | 0.93 | NaN | NaN | 1.74 |
| P50416 | Carnitine O-palmitoyltransferase 1, liver isoform | CPT1A | 8 | 13.2 | 0 | 17.54 | 0.98 | 0.95 | 0.91 | 0.83 | 1.26 | 0.96 | 1.38 | NaN | 0.99 |
| P50453 | Serpin B9 | SERPINB9 | 10 | 37.2 | 0 | 29.11 | 1.25 | NaN | 1.43 | 0.66 | 1.03 | NaN | NaN | NaN | 1.43 |
| P50454 | Serpin H1 | SERPINH1 | 17 | 47.6 | 0 | 307.8 | 1.06 | 1.01 | 1.04 | 0.98 | 1.07 | 0.95 | 0.96 | 1.01 | 1.03 |
| P50502 | Hsc70-interacting protein;Putative protein FAM10A5;Putative protein | ST13 | 9 | 29 | 0 | 121.4 | 1.05 | 1.04 | 1.11 | 1.04 | 1.00 | 1.00 | 0.87 | 0.84 | 0.99 |

| | | | | | | | | | | | | | | |
|---|---|---|---|---|---|---|---|---|---|---|---|---|---|---|
| P50552 | Vasodilator-stimulated phosphoprotein | VASP | 11 | 36.6 | 0 | 73.08 | 1.09 | 1.21 | 1.21 | 1.61 | 1.08 | 0.69 | 2.01 | 0.93 | 1.06 |
| P50570 | Dynamin-2 | DNM2 | 13 | 30.8 | 0 | 97.16 | 1.25 | 1.01 | 0.99 | 0.66 | 0.98 | 1.13 | 1.29 | 1.16 | 0.86 |
| P50583 | Bis(5-nucleosyl)-tetraphosphatase [asymmetrical] | NUDT2 | 1 | 16.3 | 0 | 10.78 | 0.77 | 0.80 | 0.74 | NaN | NaN | 0.82 | NaN | NaN | NaN |
| P50914 | 60S ribosomal protein L14 | RPL14 | 5 | 26 | 0 | 21.07 | 0.80 | 0.84 | 0.88 | 0.88 | 0.61 | 0.82 | 0.74 | NaN | 0.72 |
| P50990 | T-complex protein 1 subunit theta | CCT8 | 39 | 73.9 | 0 | 323.3 | 1.06 | 1.02 | 1.07 | 1.10 | 0.99 | 1.10 | 0.96 | 0.87 | 0.92 |
| P50991 | T-complex protein 1 subunit delta | CCT4 | 27 | 61.2 | 0 | 323.3 | 1.05 | 0.98 | 1.09 | 0.99 | 1.03 | 1.11 | 0.85 | 0.85 | 0.91 |
| P50995 | Annexin A11 | ANXA11 | 15 | 31.5 | 0 | 110.2 | 0.72 | 0.67 | 0.88 | 0.79 | 0.90 | 1.01 | 0.91 | 1.10 | 0.88 |
| P51114 | Fragile X mental retardation syndrome-related protein 1 | FXR1 | 7 | 21.7 | 0 | 67.08 | 1.08 | 1.00 | 1.27 | 0.82 | 1.33 | 1.28 | 2.11 | 0.89 | 1.16 |
| P51116 | Fragile X mental retardation syndrome-related protein 2 | FXR2 | 3 | 8.8 | 0 | 5.968 | 0.99 | 0.95 | NaN | NaN | NaN | NaN | 0.73 | NaN | NaN |
| P51148 | Ras-related protein Rab-5C | RAB5C | 7 | 65.3 | 0 | 271.2 | 0.96 | 1.00 | 0.97 | 0.95 | 1.01 | 1.03 | 1.02 | 1.08 | 0.90 |
| P51149 | Ras-related protein Rab-7a | RAB7A | 11 | 69.6 | 0 | 71.23 | 0.93 | 0.90 | 0.84 | 0.96 | 0.87 | 0.91 | 1.17 | 1.52 | 1.04 |
| P51397 | Death-associated protein 1 | DAP | 2 | 19.6 | 5E-04 | 2.767 | NaN | NaN | NaN | 0.97 | NaN | 1.13 | NaN | 0.58 | NaN |
| P51571 | Translocon-associated protein subunit delta | SSR4 | 3 | 24.3 | 0 | 25.4 | 0.99 | 1.13 | NaN | NaN | 0.93 | NaN | NaN | NaN | 1.06 |
| P51572 | B-cell receptor-associated protein 31 | BCAP31 | 14 | 48 | 0 | 35.45 | 0.75 | 0.72 | 0.66 | 1.16 | 0.96 | 1.10 | 1.11 | 1.16 | 0.69 |
| P51665 | 26S proteasome non-ATPase regulatory subunit 7 | PSMD7 | 8 | 36.4 | 0 | 150 | 1.11 | 1.03 | 1.13 | 1.34 | 1.02 | 0.98 | 0.56 | 1.13 | 0.89 |
| P51858 | Hepatoma-derived growth factor | HDGF | 13 | 65 | 0 | 323.3 | 0.89 | 0.72 | 0.79 | 1.04 | 1.08 | 0.83 | 0.96 | 1.33 | 0.79 |
| P51970 | NADH dehydrogenase [ubiquinone] 1 alpha subcomplex subunit 8 | NDUFA8 | 3 | 20.3 | 0 | 5.947 | 1.02 | 1.17 | 1.11 | 1.57 | 1.31 | 1.27 | NaN | 1.14 | 1.20 |
| P51991 | Heterogeneous nuclear ribonucleoprotein A3 | HNRNPA3 | 19 | 49.7 | 0 | 323.3 | 0.84 | 0.83 | 0.81 | 0.82 | 0.93 | 0.84 | 0.99 | 0.94 | 0.92 |
| P52209 | 6-phosphogluconate dehydrogenase, decarboxylating | PGD | 19 | 44.9 | 0 | 184.5 | 0.86 | 0.93 | 0.89 | 0.94 | 0.94 | 1.01 | 0.95 | 0.82 | 1.00 |
| P52272 | Heterogeneous nuclear ribonucleoprotein M | HNRNPM | 38 | 61.2 | 0 | 323.3 | 0.88 | 0.88 | 0.83 | 1.04 | 0.99 | 0.96 | 0.80 | 1.10 | 0.78 |
| P52292 | Importin subunit alpha-1 | KPNA2 | 9 | 21.7 | 0 | 265.6 | 1.33 | 1.11 | 1.19 | 1.37 | 1.45 | 1.39 | 0.76 | 0.64 | 1.22 |
| P52306 | Rap1 GTPase-GDP dissociation stimulator 1 | RAP1GDS | 7 | 16.5 | 0 | 9.593 | NaN | 0.66 | 0.69 | 1.09 | NaN | NaN | 1.13 | 1.01 | 1.39 |
| P52565 | Rho GDP-dissociation inhibitor 1 | ARHGDIA | 11 | 60.3 | 0 | 323.3 | 0.93 | 0.97 | 1.05 | 0.95 | 1.02 | 0.94 | 0.91 | 1.02 | 0.94 |
| P52566 | Rho GDP-dissociation inhibitor 2 | ARHGDIB | 6 | 50.7 | 0 | 49.38 | 0.88 | 1.14 | 0.92 | NaN | NaN | 0.93 | NaN | 1.38 | 0.91 |
| P52597 | Heterogeneous nuclear ribonucleoprotein F;Heterogeneous nuclear ribonucleoprotein F, N-terminally processed | HNRNPF | 13 | 54.7 | 0 | 112.2 | 0.99 | 1.16 | 1.00 | 1.09 | 0.98 | 1.02 | 0.94 | 0.97 | 0.90 |
| P52788 | Spermine synthase | SMS | 21 | 73 | 0 | 323.3 | 0.92 | 1.04 | 0.98 | 1.02 | 1.00 | 1.04 | 1.05 | 0.98 | 1.01 |
| P52888 | Thimet oligopeptidase | THOP1 | 4 | 8.9 | 0 | 16.69 | 0.90 | 0.82 | 1.09 | NaN | 0.97 | NaN | 2.18 | 1.23 | 1.17 |
| P52907 | F-actin-capping protein subunit alpha-1 | CAPZA1 | 11 | 64.7 | 0 | 323.3 | 0.98 | 1.16 | 1.17 | 0.99 | 1.04 | 1.05 | 1.03 | 0.92 | 1.08 |
| P53004 | Biliverdin reductase A | BLVRA | 8 | 32.4 | 0 | 153.2 | 0.85 | 0.71 | 0.72 | 0.96 | 0.92 | 0.77 | 0.69 | 0.72 | 0.76 |

| | | | | | | | | | | | | | | | |
|---|---|---|---|---|---|---|---|---|---|---|---|---|---|---|---|
| P53007 | Tricarboxylate transport protein, mitochondrial | SLC25A1 | 6 | 26.4 | 0 | 31.7 | 0.82 | 0.80 | 0.95 | 0.77 | 1.15 | 0.84 | 0.95 | 0.67 | 0.85 |
| P53396 | ATP-citrate synthase | ACLY | 36 | 38.1 | 0 | 308.1 | 0.95 | 0.91 | 0.92 | 0.94 | 0.91 | 0.82 | 1.04 | 1.04 | 1.02 |
| P53582 | Methionine aminopeptidase 1 | METAP1 | 2 | 8.5 | 0 | 2.872 | 1.09 | 0.93 | 1.24 | NaN | NaN | NaN | NaN | NaN | 1.44 |
| P53597 | Succinyl-CoA ligase [ADP/GDP-forming] subunit alpha, mitochondrial | SUCLG1 | 5 | 22.8 | 0 | 11.53 | NaN | 0.75 | 1.14 | NaN | 0.47 | 0.46 | 0.63 | 1.91 | 0.81 |
| P53618 | Coatomer subunit beta | COPB1 | 37 | 49.3 | 0 | 281.2 | 0.95 | 0.87 | 0.98 | 0.95 | 0.99 | 0.91 | 1.02 | 1.03 | 0.90 |
| P53992 | Protein transport protein Sec24C | SEC24C | 8 | 9.7 | 0 | 26.82 | 0.84 | 0.66 | 0.82 | 0.78 | 1.01 | 0.81 | 0.71 | NaN | 0.80 |
| P53999 | Activated RNA polymerase II transcriptional coactivator p15 | SUB1 | 8 | 46.5 | 0 | 60.41 | 1.01 | 1.02 | 1.00 | 1.14 | NaN | 1.31 | NaN | 1.81 | 1.22 |
| P54136 | Arginine--tRNA ligase, | RARS | 24 | 43.5 | 0 | 102.6 | 0.99 | 0.94 | 0.94 | 0.96 | 0.98 | 0.95 | 0.86 | 0.77 | 0.89 |
| P54577 | Tyrosine--tRNA ligase, cytoplasmic;Tyrosine--tRNA ligase, cytoplasmic, N-terminally processed;Tyrosine--tRNA ligase | YARS | 16 | 41.1 | 0 | 63.11 | 1.01 | 1.06 | 1.00 | 0.95 | 0.79 | 0.92 | 0.86 | 1.00 | 1.31 |
| P54578 | Ubiquitin carboxyl-terminal hydrolase 14;Ubiquitin carboxyl-terminal hydrolase | USP14 | 18 | 38.7 | 0 | 302.2 | 1.00 | 0.79 | 0.79 | 1.04 | 0.95 | 1.20 | 0.62 | 1.10 | 1.06 |
| P54709 | Sodium/potassium-transporting ATPase subunit beta-3 | ATP1B3 | 7 | 29.4 | 0 | 11.58 | 1.13 | 1.33 | 1.24 | 1.17 | 1.24 | 0.95 | 1.03 | 1.15 | 0.86 |
| P54727 | UV excision repair protein RAD23 homolog B | RAD23B | 9 | 32 | 0 | 323.3 | 0.96 | 0.96 | 0.61 | 1.07 | 0.94 | 0.82 | 1.07 | 1.13 | 1.00 |
| P54886 | Delta-1-pyrroline-5-carboxylate synthase;Glutamate 5-kinase;Gamma-glutamyl phosphate reductase | ALDH18A1 | 14 | 26.8 | 0 | 26.55 | 0.73 | 0.76 | 0.75 | 0.91 | NaN | 0.73 | 0.78 | 0.73 | 0.83 |
| P54920 | Alpha-soluble NSF attachment protein | NAPA | 6 | 25.8 | 0 | 9.499 | NaN | NaN | 1.06 | 0.63 | 0.71 | 0.93 | NaN | NaN | 0.60 |
| P55010 | Eukaryotic translation initiation factor 5 | EIF5 | 9 | 26.5 | 0 | 37.49 | 1.20 | 1.23 | 1.34 | 1.07 | 0.93 | 0.91 | 0.99 | 0.93 | 0.92 |
| P55036 | 26S proteasome non-ATPase regulatory subunit 4 | PSMD4 | 6 | 36.1 | 0 | 56.78 | 0.86 | 0.90 | 1.43 | 1.30 | 1.05 | 0.91 | 0.86 | 0.79 | NaN |
| P55060 | Exportin-2 | CSE1L | 34 | 37.5 | 0 | 323.3 | 0.92 | 0.91 | 0.94 | 0.92 | 0.93 | 0.90 | 0.84 | 0.83 | 0.92 |
| P55072 | Transitional endoplasmic reticulum ATPase | VCP | 40 | 58.6 | 0 | 323.3 | 0.96 | 1.02 | 1.01 | 1.04 | 1.02 | 1.07 | 0.99 | 0.96 | 0.93 |
| P55084 | Trifunctional enzyme subunit beta, mitochondrial;3-ketoacyl-CoA thiolase | HADHB | 11 | 31 | 0 | 30.96 | 1.09 | 0.95 | 1.10 | 1.23 | 0.68 | 1.28 | 0.64 | 1.07 | 0.65 |
| P55263 | Adenosine kinase | ADK | 11 | 35.9 | 0 | 49.97 | 0.98 | 0.97 | 0.84 | 0.62 | 0.80 | 0.63 | 0.95 | 1.40 | 1.17 |
| P55735 | Protein SEC13 homolog | SEC13 | 6 | 31.4 | 0 | 167.9 | NaN | NaN | 1.68 | 1.35 | 1.00 | 1.30 | 1.42 | NaN | NaN |
| P55795 | Heterogeneous nuclear ribonucleoprotein H2 | HNRNPH2 | 4 | 31.6 | 0 | 11.62 | 1.08 | 0.67 | 1.01 | 1.34 | 1.27 | 0.85 | NaN | 0.88 | 0.75 |
| P55884 | Eukaryotic translation initiation factor 3 subunit B | EIF3B | 25 | 39.4 | 0 | 323.3 | 1.18 | 1.10 | 1.19 | 0.91 | 1.23 | 1.02 | 0.97 | 1.18 | 0.94 |

| | | | | | | | | | | | | | | | | |
|---|---|---|---|---|---|---|---|---|---|---|---|---|---|---|---|---|
| P55957 | BH3-interacting domain death agonist;BH3-interacting domain death agonist p15;BH3-interacting domain death agonist p13;BH3-interacting domain death agonist p11 | BID | 4 | 36.4 | 0 | 19.15 | 0.90 | NaN | 1.38 | NaN | NaN | 1.23 | NaN | NaN | 0.89 |
| P56182 | Ribosomal RNA processing protein 1 homolog A | RRP1 | 1 | 2.6 | 0.001 | 2.732 | 1.07 | NaN | 1.21 | NaN | NaN | NaN | NaN | NaN | NaN |
| P56192 | Methionine--tRNA ligase, cytoplasmic | MARS | 12 | 20.4 | 0 | 46.39 | 0.89 | 1.01 | 1.02 | 0.77 | 0.95 | 0.87 | 0.93 | 0.81 | 0.93 |
| P56385 | ATP synthase subunit e, mitochondrial | ATP5I | 2 | 31.9 | 5E-04 | 2.788 | 1.05 | 1.16 | 1.17 | 1.14 | 1.09 | 1.94 | NaN | NaN | 1.09 |
| P56537 | Eukaryotic translation initiation factor 6 | EIF6 | 7 | 40.8 | 0 | 269.5 | 0.78 | 0.80 | 0.59 | 0.85 | 0.82 | 0.96 | 0.90 | 1.08 | 0.69 |
| P58546 | Myotrophin | MTPN | 4 | 57.6 | 0 | 195.7 | 1.14 | 1.15 | 0.92 | 1.26 | 0.85 | 1.10 | 0.96 | 1.04 | 0.97 |
| P60174 | Triosephosphate isomerase | TPI1 | 18 | 74.1 | 0 | 323.3 | 1.18 | 1.19 | 1.16 | 1.23 | 1.15 | 1.17 | 1.14 | 1.11 | 1.15 |
| P60228 | Eukaryotic translation initiation factor 3 subunit E | EIF3E | 17 | 45.4 | 0 | 109.6 | 1.03 | 1.10 | 1.09 | 0.94 | 1.23 | 1.17 | 0.83 | 0.79 | 0.93 |
| P60842 | Eukaryotic initiation factor 4A-I | EIF4A1 | 13 | 59.9 | 0 | 323.3 | 1.02 | 1.05 | 1.01 | 1.17 | 1.11 | 1.08 | 0.83 | 0.89 | 0.80 |
| P60866 | 40S ribosomal protein S20 | RPS20 | 4 | 28.6 | 0 | 18.36 | 0.86 | 0.88 | 0.89 | 0.83 | 0.95 | 0.94 | 0.80 | 0.87 | 0.84 |
| P60903 | Protein S100-A10 | S100A10 | 5 | 37.1 | 0 | 95.7 | 0.87 | 0.82 | 0.80 | 0.93 | 0.93 | 0.91 | 0.87 | 0.90 | 0.94 |
| P60953 | Cell division control protein 42 homolog | CDC42 | 6 | 42.4 | 0 | 56.09 | 1.12 | 1.04 | 1.01 | 0.99 | 0.95 | 0.99 | 0.87 | 0.91 | 0.89 |
| P60981 | Destrin | DSTN | 14 | 66.1 | 0 | 226.9 | 0.82 | 0.89 | 0.94 | 1.03 | 1.04 | 1.02 | 1.04 | 1.04 | 0.96 |
| P61006 | Ras-related protein Rab-8A | RAB8A | 5 | 35.7 | 0 | 36.55 | 1.06 | 1.05 | 1.07 | 1.15 | 0.95 | 0.85 | 1.12 | 0.86 | 0.82 |
| P61009 | Signal peptidase complex subunit 3 | SPCS3 | 2 | 11.1 | 0.005 | 1.709 | 1.42 | NaN | 1.28 | 0.75 | NaN | 1.03 | NaN | 2.03 | 1.53 |
| P61011 | Signal recognition particle 54 kDa protein | SRP54 | 8 | 21.8 | 0 | 39.59 | 0.84 | 0.60 | 0.88 | NaN | 0.72 | 0.75 | NaN | NaN | 0.84 |
| P61019 | Ras-related protein Rab-2A;Ras-related protein Rab-2B | RAB2A | 10 | 60.4 | 0 | 65.63 | 1.11 | 0.95 | 0.67 | NaN | 0.91 | 0.81 | NaN | 1.14 | 0.67 |
| P61020 | Ras-related protein Rab-5B | RAB5B | 5 | 45.1 | 0 | 30.39 | NaN | 0.91 | 0.44 | 0.86 | NaN | 1.30 | NaN | 1.11 | 1.36 |
| Q5T179 | Cyclin-dependent kinases regulatory subunit;Cyclin-dependent kinases regulatory subunit 1 | CKS1B | 2 | 38.8 | 0 | 10.59 | 1.21 | 1.20 | 1.19 | NaN | 0.98 | 1.70 | NaN | NaN | NaN |
| P61026 | Ras-related protein Rab-10 | RAB10 | 6 | 43 | 0 | 46.05 | 0.99 | 0.95 | 1.04 | 1.18 | 1.07 | 1.02 | 1.24 | 0.97 | 0.96 |
| P61081 | NEDD8-conjugating enzyme Ubc12 | UBE2M | 8 | 39.3 | 0 | 100.6 | 0.69 | 0.96 | 1.50 | 0.81 | 0.84 | 1.10 | 0.91 | 0.88 | 0.68 |
| P61086 | Ubiquitin-conjugating enzyme E2 | UBE2K | 7 | 44 | 0 | 17.9 | 1.14 | 0.92 | 1.19 | NaN | 1.04 | 1.13 | NaN | NaN | 1.03 |
| P61088 | Ubiquitin-conjugating enzyme E2 N;Putative ubiquitin-conjugating enzyme E2 N-like | UBE2N | 8 | 59.2 | 0 | 70.22 | 1.03 | 0.93 | 1.01 | 1.09 | 0.98 | 1.01 | 1.08 | 1.10 | 0.91 |
| P61106 | Ras-related protein Rab-14 | RAB14 | 13 | 70.7 | 0 | 95.77 | 1.00 | 1.02 | 1.09 | 1.02 | 0.96 | 1.05 | 0.88 | 0.93 | 0.90 |
| P61158 | Actin-related protein 3 | ACTR3 | 18 | 57.2 | 0 | 267.7 | 1.01 | 1.09 | 0.90 | 1.02 | 1.02 | 1.04 | 1.06 | 1.12 | 1.16 |
| P61160 | Actin-related protein 2 | ACTR2 | 13 | 36.3 | 0 | 92.17 | 1.18 | 1.00 | 1.12 | 1.03 | 1.20 | 0.97 | NaN | NaN | 1.06 |
| P61163 | Alpha-centractin | ACTR1A | 5 | 32.2 | 0 | 75.76 | 0.94 | 0.85 | 0.85 | 1.24 | 0.98 | 0.99 | 1.73 | 1.33 | 1.18 |

| | | | | | | | | | | | | | | | | |
|---|---|---|---|---|---|---|---|---|---|---|---|---|---|---|---|---|
| P61221 | ATP-binding cassette sub-family E member 1 | ABCE1 | 15 | 30.7 | 0 | 43.66 | 0.72 | 0.62 | 0.84 | 0.80 | 0.86 | 0.69 | 0.94 | 0.76 | 0.86 |
| P61224 | Ras-related protein Rap-1b;Ras-related protein Rap-1b-like | RAP1B | 3 | 65.2 | 0 | 90.52 | 1.14 | 0.99 | 1.06 | 0.97 | 0.90 | 0.97 | 1.12 | 1.05 | 0.80 |
| P61247 | 40S ribosomal protein S3a | RPS3A | 15 | 51.9 | 0 | 212.1 | 0.84 | 0.81 | 0.87 | 0.81 | 0.82 | 0.81 | 0.78 | 0.82 | 0.82 |
| P61254 | 60S ribosomal protein L26;60S ribosomal protein L26-like 1 | RPL26 | 6 | 36.6 | 0 | 5.751 | 0.83 | 0.87 | 0.88 | 1.53 | 0.84 | 0.79 | 0.95 | 0.66 | 0.79 |
| P61289 | Proteasome activator complex subunit 3 | PSME3 | 6 | 28.7 | 0 | 26.4 | 1.04 | 1.13 | 1.18 | 1.31 | 1.18 | 1.05 | 1.17 | 1.00 | 1.13 |
| P61313 | 60S ribosomal protein L15;Ribosomal protein L15 | RPL15 | 11 | 51 | 0 | 166.2 | 0.67 | 0.70 | 0.67 | 0.75 | 0.66 | 0.81 | 0.81 | 0.78 | 0.78 |
| P61326 | Protein mago nashi homolog | MAGOH | 1 | 42.5 | 0 | 11.13 | 0.94 | 0.91 | 1.09 | 1.21 | 1.07 | 1.05 | 0.83 | 0.97 | 1.07 |
| P61353 | 60S ribosomal protein L27 | RPL27 | 6 | 50.7 | 0 | 7.782 | 0.80 | 0.71 | 0.78 | 0.88 | 0.81 | 0.79 | 0.93 | 0.93 | 0.86 |
| P61586 | Transforming protein RhoA | RHOA | 4 | 56.5 | 0 | 285.2 | 0.97 | 0.92 | 0.93 | 1.00 | 1.01 | 1.03 | 0.88 | 0.85 | 0.89 |
| P61604 | 10 kDa heat shock protein, mitochondrial | HSPE1 | 11 | 83.3 | 0 | 120.3 | 1.22 | 1.24 | 1.32 | 1.23 | 1.21 | 1.21 | 0.99 | 0.98 | 1.00 |
| P61758 | Prefoldin subunit 3 | VBP1 | 8 | 43.7 | 0 | 5.424 | 0.77 | 0.91 | NaN | 1.09 | NaN | NaN | 0.93 | 1.01 | 1.23 |
| P61769 | Beta-2-microglobulin;Beta-2-microglobulin form pI 5.3 | B2M | 4 | 37.8 | 0 | 80.95 | 0.97 | 1.02 | 0.99 | 1.09 | 1.18 | 1.08 | 0.93 | 1.01 | 0.98 |
| P61956 | Small ubiquitin-related modifier 2;Small ubiquitin-related modifier | SUMO2 | 1 | 23.2 | 0 | 108.4 | 1.00 | 1.00 | 0.99 | 0.87 | 1.10 | 1.14 | 0.91 | 0.95 | 1.02 |
| P61970 | Nuclear transport factor 2 | NUTF2 | 5 | 62.2 | 0 | 32.49 | 1.43 | 1.18 | 1.48 | 1.54 | 1.89 | 1.45 | 1.22 | 1.29 | 1.06 |
| P61978 | Heterogeneous nuclear ribonucleoprotein K | HNRNPK | 4 | 61.6 | 0 | 323.3 | 0.89 | 0.90 | 0.92 | 0.92 | 0.95 | 0.95 | 0.88 | 0.91 | 0.99 |
| P61981 | 14-3-3 protein gamma;14-3-3 protein gamma, N-terminally processed | YWHAG | 13 | 72.5 | 0 | 323.3 | 1.14 | 1.20 | 1.13 | 1.09 | 1.15 | 1.09 | 1.08 | 1.01 | 1.05 |
| P62070 | Ras-related protein R-Ras2 | RRAS2 | 2 | 25 | 0 | 10.48 | 1.08 | 1.01 | 0.93 | 1.17 | 1.06 | 1.00 | 0.72 | 1.00 | 0.84 |
| P62081 | 40S ribosomal protein S7 | RPS7 | 3 | 60.3 | 0 | 323.3 | 0.99 | 0.93 | 0.94 | 0.83 | 0.96 | 0.89 | 0.96 | 0.93 | 1.06 |
| P62136 | Serine/threonine-protein phosphatase PP1-alpha catalytic subunit;Serine/threonine-protein phosphatase | PPP1CA | 6 | 73.6 | 0 | 319.7 | 1.17 | 1.03 | 1.09 | 1.20 | 1.16 | 1.02 | 1.12 | 1.26 | 1.17 |
| P62140 | Serine/threonine-protein phosphatase PP1-beta catalytic subunit;Serine/threonine-protein phosphatase | PPP1CB | 5 | 63.3 | 0 | 59.66 | 0.93 | 1.09 | 1.14 | 1.07 | NaN | 0.80 | NaN | 1.12 | 1.12 |
| P62191 | 26S protease regulatory subunit | PSMC1 | 15 | 45 | 0 | 133.6 | 1.04 | 1.04 | 1.03 | 0.99 | 1.12 | 1.13 | 1.09 | 0.95 | 0.86 |
| P62195 | 26S protease regulatory subunit | PSMC5 | 14 | 47 | 0 | 182.3 | 1.06 | 0.95 | 1.08 | 0.85 | 0.96 | 1.11 | 0.93 | 1.10 | 0.86 |
| P62241 | 40S ribosomal protein S8 | RPS8 | 8 | 45.7 | 0 | 143.2 | 0.91 | 0.95 | 1.03 | 0.78 | 0.91 | 0.91 | 0.85 | 0.74 | 0.73 |
| P62244 | 40S ribosomal protein S15a | RPS15A | 3 | 54.6 | 0 | 54.12 | 0.88 | 0.89 | 0.80 | 0.85 | 0.82 | 0.90 | 0.66 | 0.64 | 0.96 |
| P62249 | 40S ribosomal protein S16 | RPS16 | 8 | 50 | 0 | 23.09 | 0.51 | 0.61 | 0.74 | 0.87 | 0.73 | 0.72 | 0.48 | 0.31 | 0.65 |
| P62258 | 14-3-3 protein epsilon | YWHAE | 24 | 78.4 | 0 | 323.3 | 0.91 | 0.94 | 0.96 | 0.94 | 0.90 | 0.97 | 0.85 | 0.92 | 0.93 |
| P62266 | 40S ribosomal protein S23 | RPS23 | 6 | 43.4 | 0 | 24.33 | 0.64 | 0.64 | 0.66 | 0.62 | 0.56 | 0.67 | 0.57 | 0.79 | 0.73 |
| P62269 | 40S ribosomal protein S18 | RPS18 | 10 | 48.7 | 0 | 41.95 | 0.77 | 0.81 | 0.78 | 0.75 | 0.74 | 0.86 | 0.89 | 0.91 | 0.82 |
| P62277 | 40S ribosomal protein S13 | RPS13 | 10 | 57 | 0 | 58.17 | 0.80 | 0.75 | 0.72 | 0.62 | 0.72 | 0.83 | 0.57 | 0.82 | 0.75 |
| P62280 | 40S ribosomal protein S11 | RPS11 | 9 | 51.9 | 0 | 192.8 | 0.90 | 0.88 | 0.92 | 0.78 | 0.79 | 0.86 | 0.84 | 0.84 | 0.87 |

| | | | | | | | | | | | | | | |
|---|---|---|---|---|---|---|---|---|---|---|---|---|---|---|
| P62304 | Small nuclear ribonucleoprotein | SNRPE | 2 | 25 | 0 | 14.2 | 0.80 | 0.79 | 0.88 | 1.06 | 0.92 | 0.95 | 0.67 | 0.69 | 0.70 |
| P62306 | Small nuclear ribonucleoprotein F | SNRPF | 2 | 24.4 | 0 | 77.87 | 1.25 | 1.18 | 0.95 | 1.06 | 1.01 | 1.09 | 0.72 | 0.71 | 1.05 |
| P62310 | U6 snRNA-associated Sm-like protein LSm3 | LSM3 | 2 | 32.4 | 0 | 135.4 | 1.24 | 1.06 | 1.37 | 1.07 | NaN | 1.13 | NaN | NaN | 1.05 |
| P62314 | Small nuclear ribonucleoprotein Sm D1 | SNRPD1 | 3 | 37 | 0 | 8.623 | 0.82 | 1.01 | 0.75 | 0.76 | 4.06 | 1.26 | 0.67 | 0.90 | 1.27 |
| P62316 | Small nuclear ribonucleoprotein Sm D2 | SNRPD2 | 8 | 61.9 | 0 | 97.68 | 0.75 | 0.91 | 0.93 | 0.84 | 0.92 | 0.99 | 0.71 | 0.89 | 0.85 |
| P62318 | Small nuclear ribonucleoprotein Sm D3 | SNRPD3 | 5 | 53.2 | 0 | 23.21 | 0.75 | 0.78 | 0.90 | 1.09 | 0.92 | 0.92 | 0.81 | 0.74 | 0.76 |
| P62328 | Thymosin beta-4;Hematopoietic system regulatory peptide | TMSB4X | 11 | 88.6 | 0 | 323.3 | 0.97 | 0.96 | 0.95 | 1.03 | 1.00 | 1.01 | 0.91 | 0.86 | 0.90 |
| P62330 | ADP-ribosylation factor 6 | ARF6 | 5 | 48.6 | 0 | 60.36 | 1.01 | 0.73 | 0.88 | 0.71 | 1.09 | 0.84 | 0.57 | 0.87 | 0.72 |
| P62424 | 60S ribosomal protein L7a | RPL7A | 12 | 37.6 | 0 | 195.1 | 0.77 | 0.77 | 0.73 | 0.78 | 0.83 | 0.81 | 0.74 | 0.71 | 0.69 |
| P62633 | Cellular nucleic acid-binding protein | CNBP | 6 | 40.1 | 0 | 82.73 | 0.95 | 0.78 | 0.89 | 1.03 | 0.87 | 0.88 | 1.00 | 0.98 | 1.06 |
| P62701 | 40S ribosomal protein S4, X isoform | RPS4X | 18 | 63.5 | 0 | 238.5 | 0.81 | 0.68 | 0.83 | 0.69 | 0.73 | 0.71 | 0.69 | 0.65 | 0.75 |
| P62750 | 60S ribosomal protein L23a | RPL23A | 9 | 42.3 | 0 | 8.203 | 0.85 | 0.87 | 0.95 | 0.80 | 0.88 | 0.83 | 0.87 | 0.87 | 0.95 |
| P62753 | 40S ribosomal protein S6 | RPS6 | 6 | 27.3 | 0 | 110 | 1.02 | 1.16 | 1.09 | 0.97 | 1.02 | 0.91 | 0.80 | 0.91 | 1.16 |
| P62805 | Histone H4 | HIST1H4A | 9 | 59.2 | 0 | 135.4 | 0.84 | 0.84 | 0.83 | 0.65 | 0.66 | 0.66 | 0.90 | 0.86 | 0.91 |
| P62820 | Ras-related protein Rab-1A | RAB1A | 7 | 78.5 | 0 | 194.3 | 1.04 | 0.94 | 0.97 | 0.93 | 0.97 | 1.02 | 0.92 | 0.89 | 0.96 |
| P62829 | 60S ribosomal protein L23 | RPL23 | 7 | 52.1 | 0 | 101 | 0.72 | 0.73 | 0.66 | 0.72 | 0.83 | 0.77 | 0.82 | 0.91 | 0.90 |
| P62841 | 40S ribosomal protein S15 | RPS15 | 6 | 54.5 | 0 | 124.1 | 0.76 | 0.88 | 0.78 | 0.82 | 0.80 | 0.96 | 0.66 | 0.50 | 0.71 |
| P62851 | 40S ribosomal protein S25 | RPS25 | 6 | 37.6 | 0 | 12.05 | 0.87 | 0.85 | 0.86 | 0.84 | 0.77 | 0.86 | 0.82 | 0.85 | 0.91 |
| P62854 | 40S ribosomal protein S26;Putative 40S ribosomal protein S26-like 1 | RPS26 | 4 | 44.3 | 0 | 17.45 | 0.50 | 0.74 | 0.86 | 0.78 | 0.88 | 0.76 | 0.58 | 0.54 | NaN |
| P62857 | 40S ribosomal protein S28 | RPS28 | 3 | 46.4 | 0 | 323.3 | 1.13 | 1.20 | 1.03 | 1.12 | 1.11 | 1.21 | 1.09 | 1.05 | 0.88 |
| P62873 | Guanine nucleotide-binding protein G(I)/G(S)/G(T) subunit beta-1 | GNB1 | 9 | 50 | 0 | 240 | 0.88 | 0.73 | 0.83 | 0.72 | 0.88 | 0.96 | 0.81 | 0.88 | 0.72 |
| P62877 | E3 ubiquitin-protein ligase RBX1;E3 ubiquitin-protein ligase RBX1, N-terminally processed | RBX1 | 3 | 29.6 | 0 | 40.71 | NaN | 1.12 | 0.93 | NaN | NaN | 1.03 | NaN | NaN | 0.84 |
| P62879 | Guanine nucleotide-binding protein G(I)/G(S)/G(T) subunit beta-2 | GNB2 | 5 | 50 | 0 | 151.4 | 0.86 | 0.85 | 1.02 | NaN | 0.96 | 0.96 | 0.44 | 1.39 | 0.79 |
| P62888 | 60S ribosomal protein L30 | RPL30 | 6 | 66.1 | 0 | 109.7 | 1.04 | 0.89 | 0.93 | 1.03 | 1.08 | 0.90 | 1.03 | 1.08 | 0.99 |
| Q59GN2 | Putative 60S ribosomal protein L39-like 5;60S ribosomal protein L39 | RPL39P5 | 1 | 19.6 | 0 | 3.923 | 1.33 | 1.33 | 1.36 | NaN | 0.98 | 1.15 | NaN | NaN | 1.48 |
| P62899 | 60S ribosomal protein L31 | RPL31 | 5 | 40 | 0 | 91.88 | 0.78 | 0.91 | NaN | 0.78 | 0.69 | 0.76 | 0.87 | 0.91 | 0.75 |
| P62906 | 60S ribosomal protein L10a | RPL10A | 12 | 48.4 | 0 | 53.56 | 0.87 | 0.82 | 0.90 | 0.88 | 0.74 | 0.80 | 0.72 | 0.90 | 1.01 |
| Q5VVC8 | 60S ribosomal protein L11 | RPL11 | 4 | 22.2 | 0 | 80.59 | 0.78 | 0.75 | 0.76 | 0.81 | 0.82 | NaN | 0.92 | 0.88 | 0.86 |

| | | | | | | | | | | | | | | | |
|---|---|---|---|---|---|---|---|---|---|---|---|---|---|---|---|
| P62937 | Peptidyl-prolyl cis-trans isomerase A;Peptidyl-prolyl cis-trans isomerase A, N-terminally processed;Peptidyl-prolyl cis-trans isomerase | PPIA | 11 | 78.8 | 0 | 323.3 | 1.08 | 1.14 | 1.10 | 1.20 | 1.14 | 1.14 | 1.07 | 1.08 | 1.13 |
| P62942 | Peptidyl-prolyl cis-trans isomerase FKBP1A;Peptidyl-prolyl cis-trans isomerase | FKBP1A | 7 | 73.1 | 0 | 184.9 | 0.99 | 1.10 | 1.18 | 1.69 | 1.14 | 1.17 | 1.39 | 1.42 | 0.93 |
| P62979 | Ubiquitin-40S ribosomal protein S27a;Ubiquitin;40S ribosomal protein S27a;Polyubiquitin-B;Ubiquitin;Polyubiquitin-C;Ubiquitin | RPS27A | 3 | 57.1 | 0 | 323.3 | 0.93 | 1.06 | 1.25 | 0.81 | 0.81 | 0.90 | 0.98 | 0.90 | 1.14 |
| P62993 | Growth factor receptor-bound protein 2 | GRB2 | 7 | 41.5 | 0 | 16.31 | NaN | 1.44 | 1.72 | 1.16 | NaN | 1.16 | 1.02 | NaN | 0.98 |
| P62995 | Transformer-2 protein homolog beta | TRA2B | 7 | 28.1 | 0 | 18.21 | 0.97 | 0.97 | 0.96 | NaN | 1.24 | 1.14 | NaN | NaN | 0.90 |
| P63000 | Ras-related C3 botulinum toxin substrate 1 | RAC1 | 4 | 41.1 | 0 | 52.44 | 0.96 | 0.98 | 0.96 | 1.01 | 1.05 | 0.97 | 0.71 | 0.80 | 0.85 |
| P63010 | AP-2 complex subunit beta | AP2B1 | 18 | 39.8 | 0 | 207.7 | 1.12 | 1.00 | 1.01 | 0.87 | 0.94 | 0.95 | 0.95 | 0.97 | 1.03 |
| P63092 | Guanine nucleotide-binding protein G(s) subunit alpha isoforms short;Guanine nucleotide-binding protein G(s) subunit alpha isoforms XLas | GNAS | 4 | 17.5 | 0 | 27 | 1.40 | 0.89 | 1.00 | NaN | 1.04 | NaN | NaN | 1.64 | 1.14 |
| P63104 | 14-3-3 protein zeta/delta | YWHAZ | 16 | 75.9 | 0 | 323.3 | 0.97 | 0.93 | 0.92 | 0.95 | 0.93 | 0.92 | 0.90 | 0.93 | 0.96 |
| P63167 | Dynein light chain 1, cytoplasmic | DYNLL1 | 3 | 58.4 | 0 | 311.6 | 0.70 | 0.82 | 0.77 | 0.87 | 0.96 | 0.75 | 0.44 | 0.89 | 0.91 |
| P63172 | Dynein light chain Tctex-type 1 | DYNLT1 | 3 | 38.9 | 0 | 40.08 | 0.76 | 1.28 | 0.75 | 0.80 | NaN | 0.75 | 0.80 | NaN | 0.76 |
| P63173 | 60S ribosomal protein L38 | RPL38 | 4 | 50 | 0 | 6.379 | 0.59 | 0.56 | 0.77 | NaN | NaN | 0.48 | 0.61 | 0.63 | 0.71 |
| Q8WVC2 | 40S ribosomal protein S21 | RPS21 | 6 | 74.1 | 0 | 105.1 | 0.96 | 0.97 | 1.04 | 0.95 | 0.98 | 1.05 | NaN | 0.99 | 0.80 |
| P63244 | Guanine nucleotide-binding protein subunit beta-2-like 1;Guanine nucleotide-binding protein subunit beta-2-like 1, N-terminally processed | GNB2L1 | 20 | 79.5 | 0 | 193.2 | 0.98 | 0.94 | 0.91 | 0.87 | 1.00 | 0.81 | 0.95 | 0.92 | 0.95 |
| P63261 | Actin, cytoplasmic 2;Actin, cytoplasmic 2, N-terminally processed | ACTG1 | 3 | 80.5 | 0 | 323.3 | 0.98 | 0.96 | 0.98 | 0.95 | 0.95 | 0.94 | 1.05 | 1.03 | 1.06 |
| P63313 | Thymosin beta-10 | TMSB10 | 3 | 54.5 | 0 | 90.42 | 0.58 | 0.69 | 0.77 | 0.94 | 1.02 | 1.08 | NaN | NaN | 0.80 |
| P67775 | Serine/threonine-protein phosphatase 2A catalytic subunit alpha isoform;Serine/threonine-protein phosphatase 2A catalytic subunit beta isoform | PPP2CA | 9 | 53.1 | 0 | 131.7 | 1.13 | 1.21 | 1.20 | 0.90 | 1.13 | 1.16 | 1.15 | 0.90 | 0.98 |
| P67809 | Nuclease-sensitive element-binding protein 1 | YBX1 | 10 | 54 | 0 | 323.3 | 1.25 | 1.22 | 1.19 | 1.11 | 1.09 | 1.09 | 0.98 | 1.03 | 1.01 |
| Q5SRQ6 | Casein kinase II subunit beta | CSNK2B | 6 | 38 | 0 | 8.063 | 1.77 | 1.25 | 1.43 | NaN | 0.86 | 0.90 | NaN | 3.09 | 1.11 |

| | | | | | | | | | | | | | | | |
|---|---|---|---|---|---|---|---|---|---|---|---|---|---|---|---|
| P67936 | Tropomyosin alpha-4 chain | TPM4 | 8 | 73 | 0 | 323.3 | 1.20 | 1.11 | 1.08 | 0.94 | 0.97 | 0.93 | 1.19 | 1.11 | 1.16 |
| P68032 | Actin, alpha cardiac muscle 1;Actin, gamma-enteric smooth muscle;Actin, alpha skeletal muscle;Actin, aortic smooth | ACTC1 | 4 | 45.6 | 0 | 22.92 | 0.91 | NaN | NaN | 0.91 | NaN | 0.99 | 0.62 | 0.64 | 0.84 |
| P68036 | Ubiquitin-conjugating enzyme E2 L3 | UBE2L3 | 7 | 63.6 | 0 | 173.7 | 1.01 | 1.18 | 1.09 | 1.13 | 1.11 | 1.09 | 1.16 | 1.34 | 0.96 |
| P68104 | Elongation factor 1-alpha 1;Putative elongation factor 1-alpha-like 3;Elongation factor 1-alpha | EEF1A1 | 17 | 74.9 | 0 | 323.3 | 0.82 | 0.79 | 0.82 | 0.94 | 0.90 | 0.92 | 0.77 | 0.87 | 0.85 |
| P68363 | Tubulin alpha-1B chain | TUBA1B | 0 | 80.7 | 0 | 323.3 | 0.67 | 0.61 | 0.60 | 0.67 | 0.71 | 0.69 | 1.03 | 0.87 | 0.89 |
| P68366 | Tubulin alpha-4A chain | TUBA4A | 3 | 56.5 | 0 | 17.53 | 1.00 | 0.99 | 1.23 | NaN | 1.01 | 0.97 | NaN | 1.95 | 2.09 |
| P68371 | Tubulin beta-4B chain | TUBB4B | 1 | 67 | 0 | 323.3 | 0.55 | 0.52 | 0.53 | 0.61 | 0.61 | 0.60 | 0.94 | 0.86 | 0.87 |
| P68402 | Platelet-activating factor acetylhydrolase IB subunit beta | PAFAH1B2 | 8 | 58.1 | 0 | 143.5 | 1.11 | 1.01 | 0.96 | 1.01 | 1.07 | 1.04 | 1.08 | 1.04 | 1.10 |
| Q71DI3 | Histone H3.2;Histone H3.1t;Histone H3.1;Histone H3 | HIST2H3A | 3 | 39.7 | 0 | 45.61 | 0.79 | 0.77 | 0.76 | 0.67 | 0.67 | 0.63 | 0.88 | 0.82 | 0.68 |
| P78318 | Immunoglobulin-binding protein 1 | IGBP1 | 3 | 11.5 | 0 | 48.45 | NaN | 0.72 | 1.12 | NaN | NaN | 1.26 | NaN | 0.87 | 1.26 |
| P78344 | Eukaryotic translation initiation factor 4 gamma 2 | EIF4G2 | 10 | 14.6 | 0 | 25.93 | 1.06 | 0.99 | 1.21 | 1.21 | 1.02 | 0.89 | 1.02 | 0.81 | 1.01 |
| P78346 | Ribonuclease P protein subunit p30 | RPP30 | 2 | 9.7 | 0.002 | 1.9 | 0.99 | 1.00 | 1.12 | NaN | 0.85 | 1.01 | NaN | NaN | NaN |
| P78371 | T-complex protein 1 subunit beta | CCT2 | 35 | 80 | 0 | 323.3 | 1.07 | 1.06 | 1.06 | 1.01 | 1.00 | 1.06 | 0.96 | 1.08 | 0.97 |
| P78417 | Glutathione S-transferase omega-1 | GSTO1 | 20 | 66.4 | 0 | 227.5 | 1.22 | 1.17 | 1.22 | 1.22 | 1.17 | 1.21 | 1.08 | 1.01 | 1.16 |
| P78527 | DNA-dependent protein kinase catalytic subunit | PRKDC | 69 | 20.6 | 0 | 226.7 | 0.88 | 0.76 | 0.79 | 1.06 | 0.90 | 0.91 | 0.90 | 1.01 | 0.94 |
| P80723 | Brain acid soluble protein 1 | BASP1 | 14 | 89.9 | 0 | 323.3 | 0.93 | 0.98 | 0.95 | 0.90 | 0.88 | 0.91 | 1.02 | 1.09 | 1.15 |
| P82673 | 28S ribosomal protein S35, mitochondrial | MRPS35 | 3 | 11.1 | 0 | 3.985 | 0.69 | 0.54 | 0.56 | NaN | 1.16 | 0.49 | NaN | NaN | NaN |
| P83111 | Serine beta-lactamase-like protein LACTB, mitochondrial | LACTB | 4 | 10.6 | 0 | 7.36 | 1.05 | 1.02 | 0.97 | NaN | NaN | 0.91 | NaN | NaN | NaN |
| P83916 | Chromobox protein homolog 1 | CBX1 | 5 | 55.7 | 0 | 51.41 | NaN | 0.55 | NaN | 0.70 | NaN | 0.58 | 0.72 | 0.81 | 0.72 |
| P84022 | Mothers against decapentaplegic homolog 3;Mothers against decapentaplegic homolog;Mothers against decapentaplegic homolog 2;Mothers against | SMAD3 | 3 | 8 | 0 | 3.479 | 0.48 | NaN | NaN | 0.55 | NaN | 0.72 | NaN | NaN | NaN |
| P84077 | ADP-ribosylation factor 1;ADP-ribosylation factor 3 | ARF1 | 4 | 59.7 | 0 | 60.34 | 0.75 | 0.77 | 0.88 | 1.03 | 0.95 | 0.92 | 0.66 | 0.71 | 0.67 |
| P84090 | Enhancer of rudimentary | ERH | 5 | 51 | 0 | 323.3 | 0.95 | 1.10 | 1.03 | 1.13 | 0.87 | 1.01 | 0.91 | 0.98 | 1.35 |
| P84095 | Rho-related GTP-binding protein RhoG | RHOG | 6 | 47.6 | 0 | 14.04 | 1.17 | 1.01 | 1.13 | 0.69 | NaN | 1.19 | 4.43 | 5.19 | 1.01 |
| P84103 | Serine/arginine-rich splicing factor 3 | SRSF3 | 8 | 47 | 0 | 23.35 | 1.06 | 1.08 | 1.04 | 1.01 | 0.96 | 0.96 | 1.18 | 1.07 | 1.16 |

| | | | | | | | | | | | | | | | |
|---|---|---|---|---|---|---|---|---|---|---|---|---|---|---|---|
| P98179 | RNA-binding protein 3 | RBM3 | 4 | 47.8 | 0 | 323.3 | 0.80 | 0.75 | 0.80 | 0.94 | 0.85 | 0.92 | 0.81 | 0.85 | 0.88 |
| Q00688 | Peptidyl-prolyl cis-trans isomerase FKBP3 | FKBP3 | 11 | 52.7 | 0 | 47.91 | 0.81 | 1.06 | NaN | 1.19 | 1.11 | 1.15 | NaN | 3.06 | 1.01 |
| Q00765 | Receptor expression-enhancing protein 5 | REEP5 | 5 | 20.1 | 0 | 4.783 | 1.20 | 1.15 | 1.04 | 1.23 | NaN | 1.27 | 1.06 | 0.94 | 0.90 |
| Q00839 | Heterogeneous nuclear ribonucleoprotein U | HNRNPU | 4 | 41.9 | 0 | 323.3 | 0.73 | 0.80 | 0.76 | 0.80 | 0.82 | 0.86 | 0.80 | 0.86 | 0.87 |
| Q01082 | Spectrin beta chain, non-erythrocytic 1 | SPTBN1 | 3 | 60.5 | 0 | 323.3 | 0.72 | 0.73 | 0.74 | 0.77 | 0.76 | 0.77 | 0.92 | 0.92 | 0.92 |
| Q01085 | Nucleolysin TIAR | TIAL1 | 4 | 11.7 | 0 | 11.16 | 0.95 | NaN | 1.10 | NaN | 0.78 | NaN | NaN | NaN | NaN |
| Q01518 | Adenylyl cyclase-associated protein 1 | CAP1 | 23 | 60.2 | 0 | 323.3 | 1.06 | 1.06 | 1.05 | 1.07 | 1.01 | 1.06 | 1.04 | 1.03 | 1.01 |
| Q01650 | Large neutral amino acids transporter small subunit 1 | SLC7A5 | 3 | 9.5 | 0 | 89.18 | 1.58 | 0.90 | 0.94 | 0.88 | 1.18 | 0.71 | 1.00 | 1.01 | 1.74 |
| Q01813 | ATP-dependent 6-phosphofructokinase, platelet | PFKP | 33 | 56.1 | 0 | 323.3 | 0.97 | 0.98 | 0.96 | 1.02 | 1.01 | 1.05 | 0.98 | 1.08 | 1.03 |
| Q01970 | 1-phosphatidylinositol 4,5-bisphosphate phosphodiesterase beta-3 | PLCB3 | 7 | 8.1 | 0 | 11.51 | NaN | NaN | 0.95 | 0.42 | 0.92 | NaN | 0.85 | 0.86 | NaN |
| Q02790 | Peptidyl-prolyl cis-trans isomerase FKBP4;Peptidyl-prolyl cis-trans isomerase FKBP4, N-terminally processed | FKBP4 | 15 | 46 | 0 | 70.57 | 0.81 | 0.83 | 1.21 | 1.09 | 1.07 | 1.17 | 0.69 | 0.84 | 0.51 |
| Q02809 | Procollagen-lysine,2-oxoglutarate 5-dioxygenase 1 | PLOD1 | 11 | 22.3 | 0 | 14.53 | 0.58 | 0.70 | 0.68 | NaN | 0.62 | NaN | NaN | 1.06 | 1.12 |
| Q02878 | 60S ribosomal protein L6 | RPL6 | 13 | 45.1 | 0 | 139.6 | 0.99 | 1.00 | 0.96 | 0.99 | 0.97 | 0.97 | 0.99 | 0.95 | 0.98 |
| Q03135 | Caveolin-1;Caveolin | CAV1 | 3 | 39.9 | 0 | 167.3 | 1.08 | 1.11 | 1.08 | 1.15 | 1.01 | 1.17 | 0.86 | 0.89 | 0.90 |
| Q03252 | Lamin-B2 | LMNB2 | 19 | 35.3 | 0 | 112.4 | 1.07 | 1.10 | 1.45 | 1.11 | 1.10 | 1.26 | 1.29 | 1.11 | 1.21 |
| Q04446 | 1,4-alpha-glucan-branching enzyme | GBE1 | 17 | 32.1 | 0 | 126.4 | 1.09 | 1.09 | 0.95 | 0.96 | 1.36 | 1.59 | 1.00 | 0.85 | 1.04 |
| Q04760 | Lactoylglutathione lyase | GLO1 | 11 | 66.3 | 0 | 279.5 | 0.85 | 0.82 | 0.92 | 0.94 | 0.95 | 0.93 | 0.82 | 0.84 | 0.80 |
| Q04837 | Single-stranded DNA-binding protein, mitochondrial | SSBP1 | 6 | 47.3 | 0 | 113 | 1.13 | 1.17 | 1.22 | 1.37 | 1.15 | 1.16 | 0.73 | 0.74 | 0.81 |
| Q04917 | 14-3-3 protein eta | YWHAH | 13 | 61.4 | 0 | 323.3 | 0.77 | 0.86 | 0.90 | 0.85 | 0.87 | 0.94 | 0.80 | 0.83 | 0.84 |
| Q04941 | Proteolipid protein 2 | PLP2 | 2 | 18.4 | 0 | 31.59 | 1.16 | 1.20 | 1.23 | 1.00 | 1.03 | 1.02 | 0.71 | 0.88 | 1.11 |
| Q5T760 | Serine/arginine-rich splicing factor 11 | SRSF11 | 2 | 6.4 | 0 | 7.365 | 1.11 | 1.03 | 1.13 | 1.03 | 1.05 | 1.11 | 0.52 | 0.75 | 1.08 |
| Q06124 | Tyrosine-protein phosphatase non-receptor type 11 | PTPN11 | 5 | 14.4 | 0 | 26.15 | 1.10 | 0.90 | 0.93 | NaN | NaN | 1.41 | NaN | NaN | NaN |
| Q06210 | Glutamine--fructose-6-phosphate aminotransferase [isomerizing] 1 | GFPT1 | 19 | 37.1 | 0 | 175.3 | 0.76 | 0.94 | 0.97 | 0.84 | 0.88 | 0.85 | 0.92 | 0.93 | 1.07 |
| Q06323 | Proteasome activator complex subunit 1 | PSME1 | 17 | 67.5 | 0 | 126.8 | 1.17 | 1.12 | 1.04 | 1.12 | 1.10 | 1.20 | 1.17 | 1.14 | 1.16 |
| Q06481 | Amyloid-like protein 2 | APLP2 | 2 | 3.7 | 0 | 21.22 | NaN | 1.19 | 0.53 | NaN | 1.06 | NaN | NaN | NaN | NaN |
| Q06830 | Peroxiredoxin-1 | PRDX1 | 15 | 77.4 | 0 | 323.3 | 0.96 | 0.95 | 0.98 | 1.03 | 1.02 | 1.02 | 0.98 | 0.94 | 0.95 |

| | | | | | | | | | | | | | | | | |
|---|---|---|---|---|---|---|---|---|---|---|---|---|---|---|---|---|
| Q07021 | Complement component 1 Q subcomponent-binding protein, mitochondrial | C1QBP | 4 | 22 | 0 | 202.1 | 1.09 | 1.24 | 1.02 | 1.07 | 1.00 | 1.03 | 0.99 | 1.05 | 1.15 |
| Q07065 | Cytoskeleton-associated protein | CKAP4 | 25 | 53 | 0 | 323.3 | 1.09 | 1.08 | 1.08 | 1.10 | 1.06 | 1.01 | 1.00 | 1.03 | 1.11 |
| Q07666 | KH domain-containing, RNA-binding, signal transduction-associated protein 1 | KHDRBS1 | 6 | 16.7 | 0 | 124.2 | 0.92 | 0.99 | 0.92 | 1.38 | 0.93 | 1.02 | NaN | NaN | 0.84 |
| Q07812 | Apoptosis regulator BAX | BAX | 6 | 40.6 | 0 | 42.87 | 1.04 | NaN | 0.68 | 1.03 | 1.63 | 0.96 | 0.54 | NaN | 0.76 |
| Q5QP56 | Bcl-2-like protein 1 | BCL2L1 | 2 | 10.5 | 1E-03 | 2.21 | 0.96 | 1.33 | NaN | NaN | NaN | 1.39 | NaN | NaN | 0.89 |
| Q07960 | Rho GTPase-activating protein 1 | ARHGAP1 | 9 | 33.5 | 0 | 50.74 | 0.97 | 0.97 | 1.04 | 0.77 | 0.85 | 0.89 | 1.88 | 1.02 | 1.08 |
| Q08170 | Serine/arginine-rich splicing factor 4 | SRSF4 | 2 | 11.3 | 0 | 323.3 | 0.96 | 1.22 | 1.20 | 1.31 | 1.10 | 1.25 | 1.59 | 1.33 | 1.24 |
| Q08209 | Serine/threonine-protein phosphatase 2B catalytic subunit alpha isoform;Serine/threonine-protein phosphatase | PPP3CA | 7 | 18 | 0 | 16.17 | 0.44 | NaN | 0.49 | 0.64 | 0.88 | NaN | 1.06 | NaN | 0.53 |
| Q08211 | ATP-dependent RNA helicase A | DHX9 | 31 | 32.3 | 0 | 323.3 | 0.90 | 0.85 | 0.86 | 0.90 | 0.88 | 0.98 | 0.95 | 0.88 | 0.97 |
| Q08380 | Galectin-3-binding protein | LGALS3BF | 7 | 15.9 | 0 | 15.55 | 0.78 | 0.73 | 0.93 | 0.84 | 0.92 | 0.86 | 0.82 | 0.71 | 1.53 |
| Q08752 | Peptidyl-prolyl cis-trans isomerase D | PPID | 3 | 13.5 | 0 | 5.036 | 0.98 | 1.23 | 1.03 | 1.26 | 0.93 | 1.18 | NaN | 0.81 | 1.00 |
| Q08945 | FACT complex subunit SSRP1 | SSRP1 | 15 | 29.3 | 0 | 116.6 | 0.73 | 0.55 | 0.84 | 0.98 | 0.99 | 0.74 | 0.98 | 1.20 | 0.92 |
| Q08J23 | tRNA (cytosine(34)-C(5))-methyltransferase | NSUN2 | 10 | 17.5 | 0 | 30.1 | NaN | NaN | 0.71 | NaN | 0.64 | 0.66 | 3.39 | NaN | 0.94 |
| Q09161 | Nuclear cap-binding protein subunit 1 | NCBP1 | 3 | 5.6 | 5E-04 | 2.832 | 1.11 | NaN | NaN | NaN | 1.43 | 0.79 | NaN | NaN | 1.22 |
| Q09666 | Neuroblast differentiation-associated protein AHNAK | AHNAK | 300 | 74.4 | 0 | 323.3 | 0.71 | 0.74 | 0.74 | 0.88 | 0.89 | 0.88 | 0.82 | 0.77 | 0.83 |
| Q0VF96 | Cingulin-like protein 1 | CGNL1 | 3 | 2.2 | 0 | 3.818 | 1.16 | 1.01 | 1.06 | 0.94 | 1.05 | 0.86 | 1.32 | 1.26 | 1.23 |
| Q10471 | Polypeptide N-acetylgalactosaminyltransferase 2;Polypeptide N-acetylgalactosaminyltransferase 2 soluble form | GALNT2 | 4 | 11.4 | 0 | 13.76 | 1.12 | 1.03 | 1.59 | NaN | 1.23 | 1.16 | NaN | NaN | 1.17 |
| Q10567 | AP-1 complex subunit beta-1 | AP1B1 | 3 | 21.4 | 0.007 | 1.649 | NaN | NaN | NaN | 1.00 | NaN | NaN | 1.65 | 1.39 | NaN |
| Q12765 | Secernin-1 | SCRN1 | 12 | 34.3 | 0 | 33.96 | 0.85 | 0.87 | 0.94 | 0.98 | 1.14 | 1.09 | 0.94 | 0.71 | 0.85 |
| Q12792 | Twinfilin-1 | TWF1 | 5 | 18 | 0 | 75.56 | 1.22 | 1.13 | 1.24 | NaN | NaN | 0.67 | NaN | NaN | NaN |
| Q12797 | Aspartyl/asparaginyl beta-hydroxylase | ASPH | 5 | 13.3 | 0 | 203.2 | 0.98 | 0.91 | 1.27 | 0.88 | 1.01 | 0.94 | 0.89 | 0.92 | 1.33 |
| Q12874 | Splicing factor 3A subunit 3 | SF3A3 | 9 | 21 | 0 | 25.11 | 0.88 | 1.06 | 0.99 | 0.90 | 0.90 | 1.13 | 1.38 | 0.86 | 0.98 |
| Q12904 | Aminoacyl tRNA synthase complex-interacting multifunctional protein 1;Endothelial monocyte- | AIMP1 | 13 | 54.2 | 0 | 124.1 | 1.04 | 0.94 | 0.98 | 1.06 | 0.99 | 1.07 | 0.48 | NaN | 0.83 |
| Q12906 | Interleukin enhancer-binding factor 3 | ILF3 | 25 | 37.9 | 0 | 323.3 | 0.83 | 0.85 | 0.79 | 0.93 | 0.93 | 0.93 | 0.80 | 0.89 | 0.91 |
| Q12931 | Heat shock protein 75 kDa, mitochondrial | TRAP1 | 7 | 14.6 | 0 | 17.92 | 0.85 | 1.02 | 0.75 | NaN | NaN | 0.85 | NaN | NaN | 0.67 |

| | | | | | | | | | | | | | | |
|---|---|---|---|---|---|---|---|---|---|---|---|---|---|---|
| Q13011 | Delta(3,5)-Delta(2,4)-dienoyl-CoA isomerase, mitochondrial | ECH1 | 6 | 36 | 0 | 34.88 | 0.99 | 1.06 | 1.10 | 0.90 | 1.00 | 1.12 | NaN | 1.01 | 1.13 |
| Q13045 | Protein flightless-1 homolog | FLII | 8 | 9.2 | 0 | 18.21 | 1.13 | 1.20 | 1.18 | 1.04 | 0.97 | 0.71 | 2.09 | 2.31 | 1.24 |
| Q13148 | TAR DNA-binding protein 43 | TARDBP | 9 | 36.2 | 0 | 45.36 | 0.78 | 0.81 | 0.99 | 1.14 | 1.03 | 1.08 | NaN | NaN | 0.97 |
| Q13151 | Heterogeneous nuclear ribonucleoprotein A0 | HNRNPA0 | 6 | 28.2 | 0 | 50.73 | 0.84 | 0.82 | 0.66 | 0.95 | 0.62 | 0.92 | 1.03 | 1.06 | 1.09 |
| Q13155 | Aminoacyl tRNA synthase complex-interacting multifunctional protein 2 | AIMP2 | 7 | 28.8 | 0 | 45.71 | NaN | 0.82 | 1.32 | NaN | 0.87 | 1.20 | NaN | NaN | 0.96 |
| Q13158 | FAS-associated death domain protein | FADD | 1 | 8.7 | 0 | 3.118 | 1.09 | 1.15 | 1.13 | 1.51 | 1.26 | 1.40 | NaN | 2.37 | 0.91 |
| Q13162 | Peroxiredoxin-4 | PRDX4 | 9 | 53.9 | 0 | 85.96 | 1.01 | 0.97 | 0.98 | 1.03 | 1.07 | 1.09 | 1.18 | 1.09 | 1.09 |
| Q13177 | Serine/threonine-protein kinase PAK 2;PAK-2p27;PAK-2p34 | PAK2 | 19 | 53.8 | 0 | 237.5 | 1.06 | 1.03 | 0.89 | 1.08 | 0.97 | 0.95 | 0.85 | 0.96 | 0.84 |
| Q13185 | Chromobox protein homolog 3 | CBX3 | 5 | 58.5 | 0 | 309.9 | 0.86 | 0.80 | 0.81 | 1.06 | 0.89 | 0.86 | 1.05 | 0.81 | 0.77 |
| Q13200 | 26S proteasome non-ATPase regulatory subunit 2 | PSMD2 | 31 | 44.9 | 0 | 260.8 | 1.15 | 1.21 | 1.39 | 1.04 | 1.07 | 1.02 | 1.09 | 1.18 | 1.06 |
| Q13242 | Serine/arginine-rich splicing factor 9 | SRSF9 | 5 | 23.5 | 0 | 31.28 | 1.61 | 1.10 | 1.02 | 1.07 | 1.26 | 0.89 | 1.60 | 1.07 | 1.40 |
| Q13243 | Serine/arginine-rich splicing factor 5 | SRSF5 | 2 | 12.9 | 0.002 | 1.893 | NaN | NaN | NaN | NaN | NaN | NaN | 1.34 | 1.22 | NaN |
| Q13247 | Serine/arginine-rich splicing factor 6 | SRSF6 | 2 | 18 | 1E-03 | 2.154 | 2.23 | 2.57 | 2.12 | NaN | 1.01 | NaN | NaN | NaN | 2.10 |
| Q13263 | Transcription intermediary factor 1-beta | TRIM28 | 16 | 33.4 | 0 | 127.3 | 1.02 | 1.08 | 1.00 | 0.90 | 1.01 | 1.12 | 0.69 | 0.76 | 0.92 |
| Q13283 | Ras GTPase-activating protein-binding protein 1 | G3BP1 | 13 | 46.6 | 0 | 148.4 | 1.38 | 1.23 | 1.23 | 0.98 | 0.92 | 1.03 | 0.90 | 1.06 | 1.04 |
| Q13347 | Eukaryotic translation initiation factor 3 subunit I | EIF3I | 10 | 55.4 | 0 | 172.7 | 1.08 | 1.00 | 1.01 | 1.24 | 1.01 | 1.02 | 1.39 | 1.00 | 0.91 |
| Q13404 | Ubiquitin-conjugating enzyme E2 variant 1 | UBE2V1 | 6 | 58.5 | 0 | 46.56 | 1.19 | 1.12 | 1.19 | 1.02 | 1.04 | 1.12 | 1.02 | 1.02 | 1.05 |
| Q13409 | Cytoplasmic dynein 1 intermediate chain 2 | DYNC1I2 | 6 | 31.2 | 0 | 323.3 | 0.89 | 1.04 | 0.68 | 0.82 | 0.67 | 0.82 | 0.83 | 0.90 | 1.06 |
| Q13423 | NAD(P) transhydrogenase, mitochondrial | NNT | 15 | 18.8 | 0 | 98.23 | 0.90 | 0.86 | 0.80 | 0.83 | 0.74 | 0.93 | 0.90 | 0.79 | 0.90 |
| Q13435 | Splicing factor 3B subunit 2 | SF3B2 | 18 | 25.7 | 0 | 91.05 | 1.21 | 0.73 | 0.90 | 1.05 | 1.02 | 1.04 | 1.07 | 0.95 | 0.68 |
| Q13442 | 28 kDa heat- and acid-stable phosphoprotein | PDAP1 | 10 | 38.7 | 0 | 178.3 | 0.99 | 0.96 | 0.99 | 1.01 | 1.14 | 0.97 | 0.80 | 0.89 | 0.96 |
| Q13492 | Phosphatidylinositol-binding clathrin assembly protein | PICALM | 7 | 16.4 | 0 | 25.95 | 1.07 | 0.94 | 0.90 | 1.07 | 1.19 | 0.89 | 1.56 | 1.08 | 0.69 |
| Q13501 | Sequestosome-1 | SQSTM1 | 10 | 38.9 | 0 | 254.7 | 1.22 | 1.35 | 1.30 | 1.63 | 1.41 | 1.42 | NaN | NaN | 0.81 |
| Q13526 | Peptidyl-prolyl cis-trans isomerase NIMA-interacting 1 | PIN1 | 5 | 55.8 | 0 | 12.33 | 0.67 | 0.64 | 0.71 | 0.82 | 0.94 | 0.68 | 0.78 | 0.89 | 0.66 |
| Q13547 | Histone deacetylase 1 | HDAC1 | 4 | 19.9 | 0 | 8.918 | 0.27 | 0.79 | 0.66 | 0.87 | 0.94 | 0.97 | NaN | NaN | 0.81 |
| Q13561 | Dynactin subunit 2 | DCTN2 | 13 | 47.1 | 0 | 194.5 | 0.90 | 0.95 | 0.95 | 1.05 | 0.95 | 0.98 | 0.96 | 1.12 | 1.04 |
| Q13564 | NEDD8-activating enzyme E1 regulatory subunit | NAE1 | 5 | 14 | 0 | 17.11 | NaN | 0.76 | NaN | NaN | NaN | 0.79 | 0.96 | 1.14 | NaN |

| | | | | | | | | | | | | | | | |
|---|---|---|---|---|---|---|---|---|---|---|---|---|---|---|---|
| Q13573 | SNW domain-containing protein | SNW1 | 4 | 15.1 | 0 | 5.545 | 0.84 | NaN | 0.67 | NaN | NaN | NaN | NaN | NaN | NaN |
| Q13596 | Sorting nexin-1 | SNX1 | 4 | 13.6 | 0 | 45.19 | 1.10 | 1.02 | 1.21 | 0.92 | 0.86 | 1.00 | 0.77 | 1.18 | 1.20 |
| Q13630 | GDP-L-fucose synthase | TSTA3 | 4 | 15.3 | 0 | 6.267 | NaN | 0.88 | 1.21 | NaN | 0.79 | 0.91 | NaN | 1.44 | 1.29 |
| Q13813 | Spectrin alpha chain, non-erythrocytic 1 | SPTAN1 | 3 | 66 | 0 | 323.3 | 0.74 | 0.75 | 0.77 | 0.79 | 0.82 | 0.78 | 0.93 | 0.92 | 0.94 |
| Q13838 | Spliceosome RNA helicase DDX39B | DDX39B | 7 | 53.3 | 0 | 323.3 | 0.90 | 0.86 | 0.89 | 0.81 | 0.89 | 0.90 | 0.95 | 0.86 | 0.96 |
| Q9BVA1 | Tubulin beta-2B chain;Tubulin beta-2A chain | TUBB2B | 3 | 57.8 | 0 | 16.04 | 0.72 | 0.56 | 0.59 | 0.75 | 0.65 | 0.74 | NaN | NaN | 0.82 |
| Q13907 | Isopentenyl-diphosphate Delta-isomerase 1 | IDI1 | 6 | 45.8 | 0 | 21.18 | 0.67 | NaN | 0.53 | NaN | 1.47 | NaN | NaN | NaN | NaN |
| Q14008 | Cytoskeleton-associated protein | CKAP5 | 18 | 11.2 | 0 | 29.52 | 1.01 | 1.10 | 0.91 | 0.94 | 1.02 | 1.22 | NaN | 0.56 | 1.13 |
| Q14011 | Cold-inducible RNA-binding protein | CIRBP | 3 | 26.7 | 0 | 47.97 | 2.31 | 1.57 | 0.62 | NaN | NaN | 1.05 | NaN | NaN | 1.48 |
| Q14019 | Coactosin-like protein | COTL1 | 11 | 66.2 | 0 | 167.5 | 1.08 | 1.04 | 1.04 | 1.00 | 0.99 | 1.00 | 1.01 | 1.00 | 0.99 |
| Q14103 | Heterogeneous nuclear ribonucleoprotein D0 | HNRNPD | 3 | 39.7 | 0 | 323.3 | 0.91 | 0.92 | 0.86 | 0.91 | 0.90 | 0.91 | 0.77 | 0.82 | 0.86 |
| Q14152 | Eukaryotic translation initiation factor 3 subunit A | EIF3A | 44 | 36.2 | 0 | 289.6 | 1.08 | 1.26 | 1.26 | 1.26 | 1.20 | 1.18 | 0.88 | 0.86 | 0.98 |
| Q14204 | Cytoplasmic dynein 1 heavy chain 1 | DYNC1H1 | 140 | 38.8 | 0 | 323.3 | 0.91 | 0.92 | 0.92 | 0.91 | 0.89 | 0.96 | 0.96 | 0.93 | 0.86 |
| Q14232 | Translation initiation factor eIF-2B subunit alpha | EIF2B1 | 4 | 16.1 | 0 | 2.982 | 0.69 | 0.58 | 0.56 | NaN | 0.59 | NaN | 0.87 | NaN | 1.56 |
| Q14240 | Eukaryotic initiation factor 4A-II;Eukaryotic initiation factor 4A-II, N-terminally processed | EIF4A2 | 7 | 44 | 0 | 15.29 | 0.83 | NaN | NaN | 1.06 | 0.53 | NaN | NaN | NaN | NaN |
| Q14247 | Src substrate cortactin | CTTN | 23 | 49.5 | 0 | 207.8 | 1.03 | 0.89 | 1.04 | 0.99 | 1.12 | 1.07 | 1.33 | 1.16 | 1.04 |
| Q14258 | E3 ubiquitin/ISG15 ligase | TRIM25 | 19 | 37.6 | 0 | 89.08 | 0.79 | 0.93 | 1.03 | 0.86 | 0.69 | 1.01 | 1.23 | 1.04 | 0.90 |
| Q14315 | Filamin-C | FLNC | 74 | 45.6 | 0 | 323.3 | 1.06 | 1.11 | 1.01 | 0.97 | 0.94 | 0.94 | 1.11 | 1.22 | 1.05 |
| Q14320 | Protein FAM50A;Protein | FAM50A | 4 | 16.5 | 0 | 15.28 | 0.91 | 0.92 | 1.12 | NaN | NaN | 1.63 | NaN | NaN | 1.66 |
| Q14344 | Guanine nucleotide-binding protein subunit alpha-13 | GNA13 | 2 | 5.3 | 0.008 | 1.589 | NaN | 1.02 | 1.38 | NaN | NaN | NaN | NaN | NaN | NaN |
| Q5QPP3 | UDP-glucose 4-epimerase | GALE | 5 | 28.6 | 0 | 23.84 | 0.81 | 1.32 | 0.90 | 0.87 | 0.93 | 1.42 | 1.05 | NaN | 0.56 |
| Q14444 | Caprin-1 | CAPRIN1 | 11 | 22.8 | 0 | 284.6 | 0.99 | 1.01 | 1.30 | 0.99 | 1.01 | 1.06 | 0.83 | 0.83 | 0.73 |
| Q14498 | RNA-binding protein 39 | RBM39 | 11 | 30.8 | 0 | 124.6 | 1.25 | 1.42 | 1.46 | 0.78 | 1.31 | 1.24 | 0.78 | 0.74 | 0.89 |
| Q14566 | DNA replication licensing factor MCM6 | MCM6 | 6 | 8.6 | 0 | 7.33 | NaN | 1.07 | NaN | 6.73 | 0.84 | 0.65 | NaN | NaN | 0.79 |
| Q14573 | Inositol 1,4,5-trisphosphate receptor type 3 | ITPR3 | 13 | 8.9 | 0 | 35.28 | 0.80 | 0.97 | 0.81 | 0.67 | 0.75 | 0.70 | NaN | 0.85 | 0.95 |
| Q14677 | Clathrin interactor 1 | CLINT1 | 2 | 4.8 | 0 | 6.117 | NaN | NaN | NaN | NaN | NaN | 0.75 | 2.04 | NaN | 1.29 |
| Q14697 | Neutral alpha-glucosidase AB | GANAB | 38 | 47.6 | 0 | 323.3 | 1.01 | 0.96 | 1.00 | 0.94 | 1.06 | 0.98 | 1.00 | 0.97 | 1.02 |
| Q14764 | Major vault protein | MVP | 24 | 37.4 | 0 | 126.8 | 0.74 | 0.66 | 0.67 | 0.72 | 0.84 | 0.94 | 0.81 | 0.69 | 1.03 |
| Q14847 | LIM and SH3 domain protein 1 | LASP1 | 17 | 57.5 | 0 | 323.3 | 0.94 | 1.01 | 0.96 | 1.09 | 1.04 | 1.01 | 0.94 | 0.91 | 0.96 |
| Q14974 | Importin subunit beta-1 | KPNB1 | 29 | 42.2 | 0 | 323.3 | 0.96 | 0.96 | 0.96 | 0.94 | 1.00 | 0.95 | 0.93 | 0.89 | 0.97 |
| Q14978 | Nucleolar and coiled-body phosphoprotein 1 | NOLC1 | 9 | 13.3 | 0 | 25.46 | 0.95 | 0.85 | 0.89 | 0.79 | 1.22 | 1.06 | 0.79 | 0.75 | 0.89 |
| Q14980 | Nuclear mitotic apparatus protein | NUMA1 | 36 | 24.3 | 0 | 323.3 | 0.76 | 0.78 | 0.59 | 0.78 | 0.72 | 0.73 | 0.66 | 0.66 | 0.81 |

| | | | | | | | | | | | | | | | |
|---|---|---|---|---|---|---|---|---|---|---|---|---|---|---|---|
| Q14CX7 | N-alpha-acetyltransferase 25, NatB auxiliary subunit | NAA25 | 3 | 3.5 | 0.009 | 1.406 | NaN | NaN | 0.67 | NaN | 0.71 | 1.02 | NaN | NaN | NaN |
| Q15008 | 26S proteasome non-ATPase regulatory subunit 6 | PSMD6 | 10 | 29.3 | 0 | 136.8 | 1.10 | 1.00 | 1.15 | 1.11 | 0.93 | 1.09 | 1.06 | 1.09 | 1.00 |
| Q15019 | Septin-2 | SEPT2 | 12 | 47.9 | 0 | 323.3 | 1.04 | 0.99 | 1.02 | 1.03 | 1.08 | 1.04 | 1.03 | 1.01 | 1.06 |
| Q15021 | Condensin complex subunit 1 | NCAPD2 | 8 | 8.1 | 0 | 11 | 1.13 | 1.30 | NaN | NaN | NaN | 1.15 | NaN | NaN | 1.26 |
| Q15029 | 116 kDa U5 small nuclear ribonucleoprotein component | EFTUD2 | 14 | 23.4 | 0 | 47.84 | 0.81 | 0.78 | 0.74 | NaN | 1.01 | 0.86 | 0.94 | 0.76 | 0.93 |
| Q15046 | Lysine--tRNA ligase | KARS | 15 | 29 | 0 | 35.21 | 1.26 | 1.03 | 1.08 | 0.88 | 1.11 | 1.00 | 1.05 | 1.05 | 1.62 |
| Q15056 | Eukaryotic translation initiation factor 4H | EIF4H | 13 | 45.6 | 0 | 284.7 | 1.17 | 1.05 | 1.23 | 1.12 | 1.10 | 1.14 | 1.11 | 0.98 | 0.87 |
| Q15075 | Early endosome antigen 1 | EEA1 | 21 | 21.2 | 0 | 113.3 | 1.07 | 1.34 | 1.11 | 1.13 | 0.93 | 1.09 | 0.87 | 1.02 | 1.24 |
| Q15084 | Protein disulfide-isomerase A6 | PDIA6 | 17 | 51.4 | 0 | 323.3 | 1.12 | 1.17 | 1.11 | 1.00 | 1.05 | 1.09 | 1.14 | 1.09 | 1.14 |
| Q15102 | Platelet-activating factor acetylhydrolase IB subunit | PAFAH1B3 | 7 | 49.8 | 0 | 323.3 | 1.03 | 0.93 | 0.93 | 1.05 | 0.91 | 0.87 | 1.00 | 1.02 | 0.89 |
| Q15121 | Astrocytic phosphoprotein PEA- | PEA15 | 7 | 45.4 | 0 | 35.98 | 1.30 | 1.28 | 1.41 | NaN | 1.22 | 1.17 | 1.12 | NaN | 1.06 |
| Q15125 | 3-beta-hydroxysteroid-Delta(8),Delta(7)-isomerase | EBP | 3 | 16.5 | 0 | 196.3 | 0.90 | 1.11 | 1.25 | 0.86 | 0.82 | 0.78 | 0.91 | 1.05 | 1.20 |
| Q15149 | Plectin | PLEC | 259 | 59 | 0 | 323.3 | 0.92 | 0.90 | 0.90 | 0.90 | 0.86 | 0.89 | 1.12 | 1.12 | 1.09 |
| Q15181 | Inorganic pyrophosphatase | PPA1 | 18 | 82 | 0 | 293.9 | 0.97 | 0.93 | 0.97 | 1.05 | 0.97 | 0.97 | 0.95 | 0.94 | 0.94 |
| Q15185 | Prostaglandin E synthase 3 | PTGES3 | 7 | 46.2 | 0 | 186.2 | 1.31 | 1.29 | 1.30 | 1.24 | 1.01 | 1.42 | 1.06 | 1.23 | 1.17 |
| Q15233 | Non-POU domain-containing octamer-binding protein | NONO | 21 | 53.9 | 0 | 122.4 | 0.90 | 0.88 | 0.90 | 0.92 | 0.97 | 1.00 | 0.88 | 0.88 | 0.83 |
| Q15293 | Reticulocalbin-1 | RCN1 | 19 | 61.3 | 0 | 323.3 | 0.90 | 0.98 | 1.00 | 1.11 | 1.06 | 1.01 | 0.96 | 1.08 | 0.88 |
| Q15363 | Transmembrane emp24 domain-containing protein 2 | TMED2 | 4 | 24.4 | 0 | 2.92 | 1.12 | 0.78 | NaN | NaN | 1.09 | NaN | 0.49 | NaN | NaN |
| Q15365 | Poly(rC)-binding protein 1 | PCBP1 | 13 | 87.1 | 0 | 323.3 | 0.92 | 0.90 | 0.83 | 0.91 | 0.95 | 0.99 | 0.93 | 0.88 | 0.94 |
| Q15366 | Poly(rC)-binding protein 2 | PCBP2 | 2 | 41.9 | 0 | 94 | 0.93 | 0.98 | 0.92 | 0.90 | 0.88 | 0.91 | 0.95 | 0.96 | 0.90 |
| Q15382 | GTP-binding protein Rheb | RHEB | 6 | 28.8 | 0 | 40.27 | 0.79 | 0.76 | 0.78 | 0.89 | 0.89 | 0.88 | 0.61 | 0.88 | 0.69 |
| Q15393 | Splicing factor 3B subunit 3 | SF3B3 | 9 | 8.4 | 0 | 9.23 | 0.88 | 0.85 | 0.96 | 0.96 | 0.87 | 1.17 | 1.10 | 0.97 | 0.88 |
| Q15417 | Calponin-3 | CNN3 | 7 | 61.7 | 0 | 323.3 | 1.17 | 1.12 | 1.15 | 1.06 | 1.02 | 1.04 | 1.16 | 1.18 | 1.20 |
| Q15424 | Scaffold attachment factor B1 | SAFB | 9 | 24.3 | 0 | 223.6 | 1.02 | 0.93 | 1.03 | 0.93 | 1.12 | 1.17 | 0.83 | 2.66 | 1.09 |
| Q15427 | Splicing factor 3B subunit 4 | SF3B4 | 5 | 20.3 | 0 | 31.36 | 0.66 | 0.87 | NaN | NaN | NaN | 0.88 | NaN | NaN | 1.04 |
| Q15428 | Splicing factor 3A subunit 2 | SF3A2 | 5 | 16.8 | 0 | 323.3 | 0.83 | 0.91 | 0.99 | 1.06 | 0.96 | 0.89 | NaN | 1.39 | 1.03 |
| Q15459 | Splicing factor 3A subunit 1 | SF3A1 | 14 | 23.8 | 0 | 159.5 | 0.90 | 1.00 | 0.92 | 1.69 | 1.15 | 0.90 | 0.85 | 1.58 | 0.85 |
| Q15637 | Splicing factor 1 | SF1 | 10 | 20.2 | 0 | 51.5 | 1.12 | 1.17 | 1.19 | 1.03 | 1.17 | 1.06 | 1.01 | 1.07 | 0.88 |
| Q15642 | Cdc42-interacting protein 4 | TRIP10 | 12 | 31.3 | 0 | 79.14 | 1.25 | 1.21 | 1.26 | 1.75 | 1.16 | 1.51 | NaN | 1.64 | 1.05 |
| Q15645 | Pachytene checkpoint protein 2 homolog | TRIP13 | 4 | 12 | 0 | 4.034 | NaN | NaN | NaN | 0.95 | 0.83 | NaN | NaN | NaN | NaN |
| Q15691 | Microtubule-associated protein RP/EB family member 1 | MAPRE1 | 18 | 76.5 | 0 | 285.3 | 1.20 | 1.19 | 1.08 | 1.05 | 1.21 | 1.02 | 0.98 | 1.00 | 1.04 |
| Q15717 | ELAV-like protein 1 | ELAVL1 | 12 | 45.7 | 0 | 255.3 | 1.00 | 0.92 | 0.99 | 0.84 | 0.92 | 0.94 | 1.02 | 0.80 | 0.94 |
| Q15738 | Sterol-4-alpha-carboxylate 3-dehydrogenase, decarboxylating | NSDHL | 6 | 27.6 | 0 | 17.21 | NaN | NaN | 0.61 | NaN | 0.92 | NaN | 0.83 | NaN | 0.65 |
| Q15758 | Neutral amino acid transporter B(0);Amino acid transporter | SLC1A5 | 6 | 13.3 | 0 | 39.9 | 0.70 | 0.84 | 0.71 | 0.92 | 0.85 | NaN | 0.92 | 0.93 | 0.97 |

| | | | | | | | | | | | | | | |
|---|---|---|---|---|---|---|---|---|---|---|---|---|---|---|
| Q15785 | Mitochondrial import receptor subunit TOM34 | TOMM34 | 3 | 14.2 | 0 | 23.12 | 0.91 | NaN | 1.09 | NaN | 0.75 | NaN | NaN | NaN | NaN |
| Q15819 | Ubiquitin-conjugating enzyme E2 variant 2 | UBE2V2 | 3 | 36.6 | 0.006 | 1.693 | 1.20 | 0.97 | 0.92 | 1.06 | 0.99 | 0.86 | NaN | NaN | NaN |
| Q15836 | Vesicle-associated membrane protein 3 | VAMP3 | 3 | 46 | 0 | 214.8 | NaN | 1.07 | NaN | 1.25 | NaN | 1.07 | 0.88 | 0.94 | 1.42 |
| Q15907 | Ras-related protein Rab-11B | RAB11B | 2 | 58.7 | 0 | 223.5 | 1.27 | 1.19 | 1.15 | 1.22 | 1.19 | 1.12 | 1.38 | 1.26 | 1.30 |
| Q16186 | Proteasomal ubiquitin receptor ADRM1 | ADRM1 | 7 | 21.4 | 0 | 179.9 | 1.25 | 1.17 | 1.01 | 1.17 | 1.07 | 1.18 | 0.80 | NaN | 1.00 |
| Q16222 | UDP-N-acetylhexosamine pyrophosphorylase;UDP-N-acetylgalactosamine pyrophosphorylase;UDP-N-acetylglucosamine pyrophosphorylase | UAP1 | 9 | 25.9 | 0 | 23.39 | 1.23 | 1.23 | 1.42 | 1.17 | 0.90 | 1.36 | NaN | 1.23 | 1.24 |
| Q16270 | Insulin-like growth factor-binding protein 7 | IGFBP7 | 7 | 39.7 | 0 | 223.2 | 1.98 | 1.93 | 2.34 | 1.06 | 1.29 | 1.55 | 2.16 | 2.56 | 2.62 |
| Q16401 | 26S proteasome non-ATPase regulatory subunit 5 | PSMD5 | 10 | 28.2 | 0 | 41.15 | NaN | NaN | NaN | 1.43 | 0.86 | NaN | 0.71 | 0.74 | 0.74 |
| Q16531 | DNA damage-binding protein 1 | DDB1 | 20 | 22.5 | 0 | 227.3 | 0.83 | 0.71 | 0.84 | 0.86 | 0.89 | 0.90 | 0.98 | 0.85 | 0.99 |
| Q16543 | Hsp90 co-chaperone Cdc37;Hsp90 co-chaperone Cdc37, N-terminally processed | CDC37 | 12 | 34.9 | 0 | 302.1 | 0.89 | 0.91 | 0.87 | 0.92 | 0.90 | 0.87 | 0.86 | 0.86 | 0.86 |
| Q16658 | Fascin | FSCN1 | 5 | 14.6 | 0 | 5.275 | 0.60 | 1.09 | 0.72 | NaN | 0.97 | 0.59 | NaN | 0.90 | 0.87 |
| Q16666 | Gamma-interferon-inducible protein 16 | IFI16 | 3 | 5.9 | 0 | 4.128 | 0.43 | NaN | 0.71 | NaN | 0.96 | 0.97 | NaN | NaN | 0.74 |
| Q16698 | 2,4-dienoyl-CoA reductase, mitochondrial | DECR1 | 7 | 28.4 | 0 | 40.33 | 0.86 | 1.15 | 0.65 | NaN | 0.97 | 0.91 | 0.58 | NaN | 0.85 |
| Q16706 | Alpha-mannosidase 2 | MAN2A1 | 2 | 3 | 0.001 | 2.695 | 1.02 | 1.02 | 0.90 | NaN | NaN | 1.29 | NaN | NaN | NaN |
| Q16891 | MICOS complex subunit MIC60 | IMMT | 24 | 43.1 | 0 | 236.6 | 1.01 | 0.91 | 1.05 | 1.00 | 0.93 | 0.94 | 0.91 | 1.09 | 0.99 |
| Q1KMD3 | Heterogeneous nuclear ribonucleoprotein U-like protein 2 | HNRNPUL | 13 | 24.1 | 0 | 178 | 0.78 | 0.90 | 0.99 | 0.82 | 0.83 | 0.98 | 0.90 | 0.57 | 1.05 |
| Q27J81 | Inverted formin-2 | INF2 | 7 | 10.1 | 0 | 35.48 | NaN | 0.92 | 1.18 | NaN | 1.36 | NaN | NaN | 1.10 | NaN |
| Q2NKX8 | DNA excision repair protein ERCC-6-like | ERCC6L | 3 | 3.8 | 0.001 | 2.641 | 0.78 | 0.81 | 1.24 | 0.89 | 0.87 | 0.88 | 0.70 | 0.67 | 1.48 |
| Q2TAY7 | WD40 repeat-containing protein SMU1;WD40 repeat-containing protein SMU1, N-terminally processed | SMU1 | 2 | 3.9 | 0 | 4.617 | 0.97 | 1.04 | 0.92 | 0.73 | 0.99 | 0.99 | 0.76 | 0.85 | 1.17 |
| Q32MZ4 | Leucine-rich repeat flightless-interacting protein 1 | LRRFIP1 | 10 | 19.3 | 0 | 100.6 | 1.20 | 1.00 | 0.97 | 0.75 | 1.05 | 0.75 | 0.91 | NaN | 1.52 |
| Q32P28 | Prolyl 3-hydroxylase 1 | LEPRE1 | 9 | 17.7 | 0 | 14.09 | NaN | 1.12 | 1.09 | 1.05 | 0.86 | 0.92 | NaN | 0.98 | 0.72 |
| Q32Q12 | Nucleoside diphosphate kinase;Nucleoside diphosphate kinase B | NME1-NMI | 1 | 71.9 | 0 | 323.3 | 0.97 | 0.98 | 0.98 | 1.00 | 1.00 | 1.00 | 1.02 | 1.03 | 1.01 |
| Q3BDU5 | | LMNA | 0 | 72.7 | 0 | 144.9 | 1.31 | 1.19 | 1.12 | NaN | 0.95 | 0.95 | NaN | NaN | 1.12 |
| Q3ZCM7 | Tubulin beta-8 chain | TUBB8 | 2 | 25.9 | 0 | 323.3 | 0.39 | 0.38 | 0.43 | 0.54 | 0.51 | 0.45 | 0.59 | 0.53 | 0.50 |

| | | | | | | | | | | | | | | |
|---|---|---|---|---|---|---|---|---|---|---|---|---|---|---|
| Q3ZCQ8 | Mitochondrial import inner membrane translocase subunit TIM50 | TIMM50 | 4 | 15.3 | 0 | 12.29 | 0.96 | 1.23 | 1.04 | NaN | NaN | 1.07 | 1.38 | 1.40 | 1.14 |
| Q52LW3 | Rho GTPase-activating protein | ARHGAP2[illegible] | 7 | 7.1 | 0 | 7.896 | NaN | NaN | 0.54 | 1.06 | 0.84 | 0.89 | 0.71 | NaN | 0.68 |
| Q53GQ0 | Very-long-chain 3-oxoacyl-CoA reductase | HSD17B12 | 7 | 34.6 | 0 | 87.16 | 0.75 | 0.85 | 1.14 | NaN | 1.00 | 0.70 | NaN | 0.52 | 0.81 |
| Q562R1 | Beta-actin-like protein 2 | ACTBL2 | 2 | 25.3 | 0 | 14.68 | 1.04 | 0.99 | NaN | 1.00 | 0.99 | 0.89 | 1.09 | 1.08 | 1.13 |
| Q56VL3 | OCIA domain-containing protein | OCIAD2 | 3 | 19.5 | 0 | 4.814 | 0.67 | 0.87 | 0.67 | NaN | NaN | 0.68 | NaN | NaN | 1.21 |
| Q58FF6 | Putative heat shock protein HSP 90-beta 4 | HSP90AB4 | 1 | 10.1 | 0 | 34 | 0.93 | 0.90 | 0.95 | 0.92 | 0.95 | 0.91 | 0.52 | 0.54 | 0.57 |
| Q58FF8 | Putative heat shock protein HSP 90-beta 2 | HSP90AB2 | 1 | 27 | 0 | 56.76 | 0.92 | 0.87 | 0.91 | 0.88 | 0.98 | 0.91 | 0.70 | NaN | 0.70 |
| Q5HYB6 | | DKFZp686 | 0 | 78.9 | 0 | 126 | 1.07 | 1.07 | 1.05 | 1.01 | 0.98 | 1.11 | 1.14 | 1.04 | 1.11 |
| Q5JPT2 | SH3 domain-containing kinase-binding protein 1 | SH3KBP1 | 13 | 27.4 | 0 | 141.4 | 2.43 | 2.36 | 2.56 | 1.54 | 1.18 | 1.05 | 0.87 | 1.22 | 1.31 |
| Q5QPL9 | RNA-binding protein Raly | RALY | 13 | 54.9 | 0 | 28.45 | 0.86 | 0.88 | 0.81 | 0.94 | 0.85 | 0.88 | 0.87 | 0.81 | 0.88 |
| Q5R3I4 | Tetratricopeptide repeat protein | TTC38 | 6 | 18.8 | 0 | 6.508 | 0.54 | 0.59 | 0.52 | 0.56 | 0.63 | 0.81 | 0.72 | 0.98 | 0.89 |
| Q5STZ8 | | ABCF1 | 1 | 12.7 | 0 | 21.82 | NaN | NaN | NaN | NaN | 0.65 | 0.71 | NaN | NaN | NaN |
| Q5T123 | SH3 domain-binding glutamic acid-rich-like protein 3 | SH3BGRL3 | 4 | 42 | 0 | 47.52 | 1.07 | 0.93 | 1.00 | 0.86 | 0.73 | 1.04 | 1.10 | 0.86 | 0.81 |
| Q9Y6H1 | Coiled-coil-helix-coiled-coil-helix domain-containing protein 2;Putative coiled-coil-helix-coiled-coil-helix domain-containing protein CHCHD2P9 | CHCHD2 | 2 | 27.2 | 0 | 36.59 | 1.05 | 0.86 | 0.91 | 1.00 | 1.00 | 1.46 | 1.09 | 1.15 | 0.87 |
| Q5T4S7 | E3 ubiquitin-protein ligase UBR4 | UBR4 | 8 | 2.7 | 0 | 37.88 | 1.03 | 0.85 | 1.06 | NaN | NaN | 0.96 | NaN | NaN | 0.78 |
| Q5T6H7 | Xaa-Pro aminopeptidase 1 | XPNPEP1 | 6 | 19.9 | 0 | 18.73 | 0.57 | 0.75 | 0.80 | 0.85 | 0.91 | 0.66 | 0.74 | 0.91 | 0.90 |
| Q5T6W2 | | HNRNPK | 1 | 57.5 | 0 | 3.313 | 0.82 | 0.66 | 0.79 | NaN | NaN | NaN | 1.01 | 0.97 | 0.79 |
| Q5TH30 | Protein NDRG3 | NDRG3 | 4 | 15.5 | 0 | 6.676 | NaN | 0.54 | NaN | 0.63 | 0.79 | 1.07 | 0.95 | 0.73 | NaN |
| Q6DD88 | Atlastin-3 | ATL3 | 11 | 28.5 | 0 | 270.9 | 0.97 | 1.00 | 0.98 | 0.89 | 0.92 | 1.07 | 0.60 | 0.73 | 1.03 |
| Q6DKJ4 | Nucleoredoxin | NXN | 10 | 31 | 0 | 21.56 | 0.82 | 0.80 | NaN | NaN | 0.87 | NaN | NaN | 0.71 | NaN |
| Q6NUK1 | Calcium-binding mitochondrial carrier protein SCaMC-1 | SLC25A24 | 24 | 53.7 | 0 | 126.5 | 1.57 | 1.71 | 1.67 | 1.19 | 1.20 | 1.20 | 1.64 | 1.20 | 1.41 |
| Q6NZI2 | Polymerase I and transcript release factor | PTRF | 11 | 34.1 | 0 | 323.3 | 1.00 | 0.98 | 1.05 | 0.97 | 1.04 | 1.06 | 0.84 | 0.96 | 0.94 |
| Q6P2Q9 | Pre-mRNA-processing-splicing factor 8 | PRPF8 | 23 | 13.1 | 0 | 39.36 | 0.64 | 0.77 | 0.92 | 0.78 | 0.85 | 0.90 | 0.80 | 0.95 | 1.07 |
| Q6PKG0 | La-related protein 1 | LARP1 | 2 | 1.9 | 1E-03 | 2.148 | 0.97 | 0.78 | 0.73 | 0.70 | NaN | NaN | 0.67 | 0.75 | 0.46 |
| Q6WKZ4 | Rab11 family-interacting protein | RAB11FIP[illegible] | 1 | 0.6 | 0.009 | 1.431 | 1.36 | 1.41 | 1.54 | 1.19 | 1.29 | 1.37 | NaN | 1.87 | 1.92 |
| Q6YN16 | Hydroxysteroid dehydrogenase-like protein 2 | HSDL2 | 8 | 25.1 | 0 | 74.97 | 1.39 | 1.17 | 1.33 | NaN | 1.17 | 1.27 | 0.98 | 1.72 | 1.52 |
| Q6ZN40 | Tropomyosin alpha-1 chain | TPM1 | 1 | 33.4 | 0 | 7.568 | 1.41 | 1.25 | NaN | NaN | NaN | 1.20 | NaN | NaN | 1.39 |
| Q70J99 | Protein unc-13 homolog D | UNC13D | 5 | 8.3 | 0 | 24.62 | NaN | 0.45 | 0.49 | NaN | NaN | 0.65 | 0.95 | NaN | 1.36 |
| Q70UQ0 | Inhibitor of nuclear factor kappa-B kinase-interacting protein | IKBIP | 4 | 12.3 | 0 | 7.237 | NaN | NaN | 1.61 | 0.58 | 1.50 | 1.31 | NaN | NaN | NaN |
| Q71U36 | Tubulin alpha-1A chain;Tubulin alpha-3E chain | TUBA1A | 1 | 80.7 | 0 | 4.532 | 0.28 | 0.40 | 0.42 | NaN | NaN | 0.46 | NaN | NaN | 0.95 |

| | | | | | | | | | | | | | | | | |
|---|---|---|---|---|---|---|---|---|---|---|---|---|---|---|---|---|
| Q7KZF4 | Staphylococcal nuclease domain-containing protein 1 | SND1 | 34 | 50.7 | 0 | 323.3 | 0.91 | 0.95 | 0.85 | 0.88 | 0.89 | 0.85 | 1.08 | 0.98 | 0.97 |
| Q7L1Q6 | Basic leucine zipper and W2 domain-containing protein 1 | BZW1 | 6 | 28.2 | 0 | 20.36 | 1.34 | 1.29 | 1.27 | 1.39 | 1.12 | 1.25 | 1.05 | 1.03 | 1.07 |
| Q7L2H7 | Eukaryotic translation initiation factor 3 subunit M | EIF3M | 15 | 47.1 | 0 | 120 | 1.03 | 0.83 | 0.89 | 1.10 | 0.95 | 0.90 | 0.54 | 1.10 | 0.87 |
| Q7L576 | Cytoplasmic FMR1-interacting protein 1 | CYFIP1 | 7 | 11.2 | 0 | 15.7 | 1.16 | 0.85 | 0.73 | 0.84 | 0.90 | 0.89 | NaN | 1.25 | 0.83 |
| Q9H8S9 | MOB kinase activator 1A;MOB kinase activator 1B | MOB1A | 2 | 10.6 | 0 | 3.127 | 1.07 | 1.17 | NaN | NaN | 1.21 | NaN | NaN | 0.75 | NaN |
| Q7RTV0 | PHD finger-like domain-containing protein 5A | PHF5A | 2 | 26.4 | 0 | 58.28 | 1.27 | 0.89 | NaN | NaN | 0.88 | NaN | NaN | NaN | 0.93 |
| Q7Z406 | Myosin-14 | MYH14 | 3 | 6.1 | 0 | 7.572 | 1.08 | 0.96 | 0.91 | 1.13 | 1.19 | 1.53 | 1.11 | 1.30 | 1.23 |
| Q7Z6Z7 | E3 ubiquitin-protein ligase | HUWE1 | 41 | 13.3 | 0 | 245.6 | 0.97 | 0.97 | 0.94 | 1.00 | 0.99 | 0.98 | 0.90 | 0.96 | 0.82 |
| Q7Z7H5 | Transmembrane emp24 domain-containing protein 4 | TMED4 | 3 | 20.3 | 0 | 18.54 | 0.87 | 0.99 | 1.07 | 1.37 | 0.98 | 0.97 | NaN | NaN | 0.93 |
| Q86TI2 | Dipeptidyl peptidase 9 | DPP9 | 6 | 8.7 | 0 | 25.64 | NaN | NaN | NaN | NaN | 0.76 | 0.77 | NaN | NaN | 0.90 |
| Q86UE4 | Protein LYRIC | MTDH | 10 | 21 | 0 | 164.3 | 0.75 | 0.98 | 1.26 | 0.97 | 1.04 | 1.00 | 0.97 | 0.89 | 0.86 |
| Q86UP2 | Kinectin | KTN1 | 32 | 27.7 | 0 | 142.3 | 1.09 | 1.17 | 1.14 | 1.20 | 1.15 | 1.33 | 0.82 | 1.01 | 1.34 |
| Q86V81 | THO complex subunit 4 | ALYREF | 5 | 36.2 | 0 | 41.14 | 0.92 | 0.90 | NaN | 1.00 | 0.86 | NaN | 0.86 | 0.87 | 0.81 |
| Q86VP6 | Cullin-associated NEDD8-dissociated protein 1 | CAND1 | 19 | 18.3 | 0 | 39.33 | 1.03 | 0.96 | 0.99 | 0.96 | 1.02 | 1.01 | 0.91 | 0.91 | 1.09 |
| Q86X55 | Histone-arginine methyltransferase CARM1 | CARM1 | 5 | 17.8 | 0 | 22.55 | NaN | 0.56 | 0.61 | NaN | 0.48 | 0.68 | NaN | NaN | NaN |
| Q86Y56 | Dynein assembly factor 5, axonemal | DNAAF5 | 3 | 4.4 | 0.001 | 2.085 | NaN | NaN | 0.92 | 0.76 | 0.68 | NaN | NaN | NaN | 1.00 |
| Q8IUE6 | Histone H2A type 2-B | HIST2H2AI | 1 | 35.4 | 0 | 6.544 | 1.11 | 0.97 | 0.82 | 1.14 | 1.22 | 0.99 | NaN | NaN | 0.85 |
| Q8IVD9 | NudC domain-containing protein | NUDCD3 | 3 | 17.2 | 0 | 2.885 | NaN | NaN | NaN | 0.63 | 0.75 | 0.95 | NaN | NaN | NaN |
| Q8IVF2 | Protein AHNAK2 | AHNAK2 | 4 | 5.3 | 0 | 5.874 | 0.91 | 0.82 | 0.99 | 0.44 | NaN | 0.77 | 1.17 | 1.37 | 0.99 |
| Q8IVM0 | Coiled-coil domain-containing protein 50 | CCDC50 | 3 | 11.8 | 0 | 8.379 | NaN | 1.36 | 1.29 | NaN | NaN | NaN | NaN | NaN | 1.58 |
| Q8IW52 | SLIT and NTRK-like protein 4 | SLITRK4 | 2 | 2.7 | 0.007 | 1.624 | 0.84 | 0.82 | 0.85 | 0.98 | NaN | 0.79 | 0.98 | NaN | 0.93 |
| Q8IWE2 | Protein NOXP20 | FAM114A1 | 9 | 23.8 | 0 | 102 | 0.93 | 0.82 | 1.11 | 0.97 | 1.04 | 0.79 | NaN | 1.01 | 1.06 |
| Q8IY81 | pre-rRNA processing protein FTSJ3 | FTSJ3 | 6 | 12.5 | 0 | 15.97 | 1.16 | 1.00 | 1.35 | NaN | 1.04 | 0.96 | NaN | 1.09 | 0.95 |
| Q8N0X7 | Spartin | SPG20 | 3 | 6.5 | 0 | 5.031 | NaN | 1.99 | 1.02 | NaN | NaN | 0.79 | NaN | NaN | 0.75 |
| Q8N163 | Cell cycle and apoptosis regulator protein 2 | CCAR2 | 5 | 9 | 0 | 11.4 | 0.77 | 0.78 | 0.78 | 1.01 | 0.95 | 0.62 | 1.11 | 0.96 | 0.91 |
| Q8N1F7 | Nuclear pore complex protein Nup93 | NUP93 | 10 | 19.4 | 0 | 43.76 | NaN | NaN | NaN | 0.60 | 0.92 | 0.87 | 0.93 | 0.87 | 1.07 |
| Q8N1G4 | Leucine-rich repeat-containing protein 47 | LRRC47 | 9 | 19.7 | 0 | 30.36 | 0.68 | 1.09 | 0.73 | 1.07 | 1.12 | 0.73 | 1.26 | 1.28 | 0.91 |
| Q8N3U4 | Cohesin subunit SA-2 | STAG2 | 2 | 3.2 | 0.001 | 2.673 | NaN | NaN | 0.76 | NaN | 0.93 | 1.06 | NaN | NaN | 0.76 |
| Q8N4X5 | Actin filament-associated protein 1-like 2 | AFAP1L2 | 4 | 9.2 | 0 | 8.154 | 1.22 | 1.07 | 1.28 | 1.26 | NaN | 1.12 | 1.39 | NaN | NaN |
| Q8N999 | Uncharacterized protein | C12orf29 | 1 | 3.1 | 5E-04 | 2.843 | 1.26 | 1.19 | 1.34 | 1.03 | 1.18 | 1.15 | 1.17 | NaN | 1.15 |
| Q8NBF2 | NHL repeat-containing protein 2 | NHLRC2 | 2 | 4.5 | 0 | 5.7 | NaN | NaN | 0.39 | 0.66 | NaN | NaN | 0.60 | NaN | 0.38 |

| | | | | | | | | | | | | | | |
|---|---|---|---|---|---|---|---|---|---|---|---|---|---|---|
| Q8NBJ5 | Procollagen galactosyltransferase 1 | COLGALT1 | 7 | 13.7 | 0 | 9.849 | 0.72 | 0.74 | 1.78 | 0.95 | 1.13 | 0.85 | NaN | 1.16 | 0.84 |
| Q8NBS9 | Thioredoxin domain-containing protein 5 | TXNDC5 | 17 | 45.1 | 0 | 322.1 | 1.12 | 1.27 | 1.23 | 1.05 | 1.09 | 1.16 | 1.32 | 1.26 | 1.26 |
| Q8NBX0 | Saccharopine dehydrogenase-like oxidoreductase | SCCPDH | 6 | 20.7 | 0 | 9.15 | NaN | NaN | NaN | 0.98 | 0.95 | NaN | 1.34 | 0.21 | NaN |
| Q8NC51 | Plasminogen activator inhibitor 1 RNA-binding protein | SERBP1 | 16 | 47.8 | 0 | 323.3 | 0.95 | 0.90 | 0.89 | 1.02 | 0.96 | 0.91 | 0.76 | 0.86 | 0.82 |
| Q8NCW5 | NAD(P)H-hydrate epimerase | APOA1BP | 5 | 27.8 | 0 | 12.04 | 0.69 | 0.78 | 0.82 | 0.81 | NaN | 0.75 | NaN | NaN | 1.21 |
| Q8NE71 | ATP-binding cassette sub-family F member 1 | ABCF1 | 9 | 17.2 | 0 | 26.38 | 0.90 | 0.80 | 0.93 | 0.89 | 0.89 | 0.80 | NaN | 0.62 | 0.83 |
| Q8NE73 | Ectonucleoside triphosphate diphosphohydrolase 4 | ENTPD4 | 2 | 6.1 | 0.008 | 1.488 | 1.01 | 0.88 | 0.87 | 1.08 | 1.01 | 1.10 | NaN | NaN | NaN |
| Q8NF37 | Lysophosphatidylcholine acyltransferase 1 | LPCAT1 | 4 | 11.4 | 0 | 10.86 | 0.75 | NaN | NaN | 0.78 | 1.95 | 0.92 | NaN | 1.03 | 1.05 |
| Q8NFQ8 | Torsin-1A-interacting protein 2 | TOR1AIP2 | 1 | 5.3 | 5E-04 | 2.814 | 1.18 | 0.94 | 1.37 | NaN | NaN | NaN | NaN | NaN | 0.97 |
| Q8NI27 | THO complex subunit 2 | THOC2 | 3 | 2.6 | 0 | 3.139 | 0.78 | NaN | 0.53 | NaN | NaN | NaN | NaN | NaN | NaN |
| Q8TAT6 | Nuclear protein localization protein 4 homolog | NPLOC4 | 8 | 17.6 | 0 | 23.4 | 1.07 | 1.32 | 1.24 | 0.76 | 0.84 | 0.92 | 1.65 | 1.25 | 1.15 |
| Q8TBC4 | NEDD8-activating enzyme E1 catalytic subunit | UBA3 | 6 | 25.3 | 0 | 12.9 | NaN | 1.74 | NaN | NaN | 0.97 | 0.88 | NaN | NaN | NaN |
| Q8TBQ9 | Protein kish-A | TMEM167A | 1 | 12.5 | 0.002 | 1.914 | 1.15 | 1.14 | 1.11 | NaN | 1.24 | 1.10 | NaN | 1.35 | 1.36 |
| Q8TCJ2 | Dolichyl-diphosphooligosaccharide--protein glycosyltransferase subunit STT3B | STT3B | 3 | 5.7 | 0 | 5.267 | 1.75 | NaN | 1.42 | 1.57 | 1.19 | 1.24 | NaN | NaN | 1.40 |
| Q8TDN6 | Ribosome biogenesis protein BRX1 homolog | BRIX1 | 2 | 5.9 | 1E-03 | 2.243 | 1.15 | 0.96 | 1.26 | 1.07 | 0.90 | 1.23 | 1.17 | 0.77 | 0.92 |
| Q8TEA8 | D-tyrosyl-tRNA(Tyr) deacylase 1;D-tyrosyl-tRNA(Tyr) deacylase | DTD1 | 2 | 20.6 | 0 | 109.7 | 0.94 | 0.86 | 0.97 | 0.81 | 0.88 | 0.88 | NaN | NaN | 0.87 |
| Q8TEX9 | Importin-4 | IPO4 | 6 | 8 | 0 | 12.98 | NaN | 0.89 | NaN | 0.61 | NaN | 0.73 | 0.81 | 0.84 | NaN |
| Q8TF65 | PDZ domain-containing protein GIPC2 | GIPC2 | 1 | 5.4 | 0 | 5.025 | 1.30 | 2.05 | 1.68 | NaN | NaN | 1.10 | NaN | NaN | 1.16 |
| Q8WUH6 | Transmembrane protein 263 | TMEM263 | 2 | 35.3 | 0 | 11.01 | NaN | NaN | 1.68 | 1.00 | 0.96 | 1.07 | NaN | NaN | NaN |
| Q8WUM4 | Programmed cell death 6-interacting protein | PDCD6IP | 33 | 46.4 | 0 | 252.7 | 0.95 | 0.92 | 0.92 | 0.87 | 0.91 | 0.94 | 0.87 | 0.90 | 1.00 |
| Q8WVM8 | Sec1 family domain-containing protein 1 | SCFD1 | 5 | 11.4 | 0 | 10.06 | NaN | 0.96 | 0.98 | NaN | NaN | 0.93 | NaN | NaN | 0.68 |
| Q8WW12 | PEST proteolytic signal-containing nuclear protein | PCNP | 6 | 42.1 | 0 | 43.13 | 1.24 | 1.18 | 1.09 | 0.83 | 1.20 | 1.08 | 1.19 | 1.10 | 1.26 |
| Q8WWM7 | Ataxin-2-like protein | ATXN2L | 6 | 7.3 | 0 | 6.696 | 1.06 | 1.15 | 1.10 | NaN | NaN | NaN | 1.15 | NaN | 0.96 |
| Q8WX92 | Negative elongation factor B | NELFB | 3 | 7.6 | 0 | 9.645 | NaN | NaN | NaN | NaN | 0.84 | 0.79 | NaN | NaN | NaN |
| Q8WX93 | Palladin | PALLD | 8 | 8 | 0 | 7.941 | 0.74 | 0.26 | 0.96 | NaN | 0.94 | 0.38 | NaN | NaN | 0.55 |
| Q8WXF1 | Paraspeckle component 1 | PSPC1 | 7 | 21.8 | 0 | 26.02 | 2.62 | NaN | NaN | 0.79 | 0.94 | NaN | 0.84 | 0.90 | NaN |
| Q8WXX5 | DnaJ homolog subfamily C member 9 | DNAJC9 | 9 | 45.8 | 0 | 32.93 | 0.71 | 0.93 | 0.82 | 0.93 | 0.90 | 0.62 | NaN | 0.80 | 0.88 |
| Q92504 | Zinc transporter SLC39A7 | SLC39A7 | 2 | 5.3 | 0 | 6.335 | 1.06 | 1.02 | NaN | 0.84 | 1.01 | 0.46 | NaN | 1.02 | 1.10 |

| | | | | | | | | | | | | | | | |
|---|---|---|---|---|---|---|---|---|---|---|---|---|---|---|---|
| Q92520 | Protein FAM3C | FAM3C | 7 | 34.8 | 0 | 24.79 | 0.98 | 0.79 | 0.96 | 0.95 | 0.98 | 1.14 | 0.90 | 0.55 | 0.71 |
| Q92597 | Protein NDRG1 | NDRG1 | 9 | 44.7 | 0 | 226.7 | 1.00 | 0.90 | 0.93 | 1.18 | 1.40 | 1.40 | 0.65 | 1.04 | 1.14 |
| Q92598 | Heat shock protein 105 kDa | HSPH1 | 32 | 51.9 | 0 | 277.9 | 1.97 | 1.66 | 1.92 | 1.68 | 1.93 | 1.93 | 0.94 | 0.75 | 0.83 |
| Q92616 | Translational activator GCN1 | GCN1L1 | 30 | 15 | 0 | 52.27 | 0.91 | 0.92 | 0.87 | 0.89 | 0.86 | 0.89 | 1.06 | 0.94 | 0.89 |
| Q92688 | Acidic leucine-rich nuclear phosphoprotein 32 family member B | ANP32B | 6 | 31.9 | 0 | 22.2 | 0.82 | 0.99 | 0.91 | 0.81 | 0.95 | 0.98 | 0.95 | 0.89 | 0.97 |
| Q92734 | Protein TFG | TFG | 6 | 29.5 | 0 | 16.8 | NaN | NaN | NaN | 1.44 | 1.23 | 1.37 | NaN | NaN | NaN |
| Q92743 | Serine protease HTRA1 | HTRA1 | 6 | 17.3 | 0 | 99.7 | 1.16 | 2.14 | 1.12 | NaN | 1.52 | 1.21 | 1.40 | NaN | 0.59 |
| Q92769 | Histone deacetylase 2 | HDAC2 | 2 | 10.9 | 0.001 | 2.742 | 0.80 | 0.67 | 0.78 | NaN | NaN | NaN | NaN | NaN | 0.75 |
| Q92804 | TATA-binding protein-associated factor 2N | TAF15 | 7 | 23.6 | 0 | 323.3 | 0.95 | 0.95 | 1.02 | 1.06 | 0.93 | 0.85 | 1.06 | 0.94 | 0.97 |
| Q92890 | Ubiquitin fusion degradation protein 1 homolog | UFD1L | 3 | 12.4 | 0 | 6.623 | NaN | NaN | NaN | NaN | 0.82 | 0.99 | NaN | NaN | NaN |
| Q92900 | Regulator of nonsense transcripts 1 | UPF1 | 14 | 15.2 | 0 | 35.09 | 1.18 | 1.17 | 1.24 | 1.13 | 1.12 | 1.15 | 0.96 | 1.02 | 0.99 |
| Q92905 | COP9 signalosome complex subunit 5 | COPS5 | 1 | 4.5 | 0 | 3.54 | 0.54 | 0.54 | NaN | NaN | NaN | NaN | NaN | NaN | NaN |
| Q92973 | Transportin-1 | TNPO1 | 14 | 27.6 | 0 | 162.2 | 1.23 | 1.07 | 1.05 | 1.03 | 0.99 | 1.01 | 1.07 | 1.05 | 0.89 |
| Q93008 | Probable ubiquitin carboxyl-terminal hydrolase FAF-X | USP9X | 9 | 5.1 | 0 | 10.16 | 0.64 | 0.96 | 1.63 | 1.08 | 1.18 | 1.12 | NaN | 1.43 | 0.86 |
| Q969E4 | Transcription elongation factor A protein-like 3 | TCEAL3 | 1 | 12 | 0 | 22.63 | 0.99 | 0.83 | 1.05 | NaN | NaN | NaN | 0.89 | NaN | 1.00 |
| Q969G5 | Protein kinase C delta-binding protein | PRKCDBP | 4 | 18.8 | 0 | 5.618 | 1.33 | NaN | 1.09 | NaN | 1.22 | 1.58 | NaN | NaN | 0.80 |
| Q969H8 | Myeloid-derived growth factor | MYDGF | 4 | 27.2 | 0 | 51.99 | 1.07 | 1.07 | 1.10 | 1.02 | 0.97 | 0.88 | 1.15 | 1.06 | 0.84 |
| Q969U7 | Proteasome assembly chaperone 2 | PSMG2 | 4 | 17.4 | 0 | 3.592 | 0.74 | NaN | 0.74 | 0.88 | 0.63 | 0.87 | 1.64 | 0.65 | 1.07 |
| Q96A49 | Synapse-associated protein 1 | SYAP1 | 3 | 11.1 | 0 | 20.85 | NaN | NaN | 0.87 | 0.61 | 3.60 | 0.94 | 1.02 | 0.64 | 0.90 |
| Q96AG4 | Leucine-rich repeat-containing protein 59 | LRRC59 | 10 | 42.7 | 0 | 146.5 | 1.10 | 1.05 | 1.06 | 1.06 | 0.95 | 1.03 | 0.98 | 0.89 | 1.07 |
| Q96B36 | Proline-rich AKT1 substrate 1 | AKT1S1 | 2 | 11.7 | 0 | 3.99 | NaN | NaN | 1.04 | 0.57 | NaN | 0.86 | 0.84 | 0.74 | 0.85 |
| Q9NVJ2 | ADP-ribosylation factor-like protein 8B;ADP-ribosylation factor-like protein 8A | ARL8B | 2 | 14 | 0 | 9.157 | 1.21 | 0.96 | 0.92 | NaN | NaN | 1.03 | NaN | NaN | 0.78 |
| Q96C19 | EF-hand domain-containing protein D2 | EFHD2 | 11 | 42.5 | 0 | 124.5 | 1.18 | 0.98 | 1.15 | 1.26 | 1.20 | 1.01 | 1.24 | 1.06 | 1.04 |
| Q96C90 | Protein phosphatase 1 regulatory subunit 14B | PPP1R14E | 3 | 48.3 | 0 | 51.71 | 1.14 | 1.07 | 1.12 | 0.75 | 0.87 | 0.86 | 1.35 | 1.13 | 1.22 |
| Q96CT7 | Coiled-coil domain-containing protein 124 | CCDC124 | 6 | 30 | 0 | 16.77 | 1.07 | 1.56 | 0.95 | NaN | 1.37 | 1.18 | 0.63 | NaN | 0.92 |
| Q96CV9 | Optineurin | OPTN | 5 | 14.6 | 0 | 29.42 | NaN | 0.97 | 0.98 | NaN | 1.06 | 1.01 | NaN | NaN | 0.93 |
| Q96CX2 | BTB/POZ domain-containing protein KCTD12 | KCTD12 | 3 | 11.4 | 0 | 14.39 | NaN | NaN | NaN | 0.83 | NaN | 0.99 | NaN | NaN | NaN |
| Q96D71 | RalBP1-associated Eps domain-containing protein 1 | REPS1 | 4 | 5.5 | 0.001 | 2.366 | 1.27 | NaN | NaN | NaN | 1.43 | NaN | 0.50 | 1.62 | 1.28 |

| | | | | | | | | | | | | | | |
|---|---|---|---|---|---|---|---|---|---|---|---|---|---|---|
| Q96DG6 | Carboxymethylenebutenolidase homolog | CMBL | 4 | 20.8 | 0 | 5.456 | 1.06 | 0.92 | 0.71 | NaN | 0.90 | 0.78 | 0.73 | 0.72 | 0.50 |
| Q96EL3 | 39S ribosomal protein L53, mitochondrial | MRPL53 | 1 | 13.4 | 1E-03 | 2.269 | 1.08 | 0.72 | 1.08 | NaN | NaN | NaN | NaN | NaN | 1.03 |
| Q96FJ2 | Dynein light chain 2, cytoplasmic | DYNLL2 | 1 | 49.4 | 0 | 14.34 | 0.83 | NaN | NaN | 1.01 | 0.78 | 0.90 | NaN | NaN | NaN |
| Q96FQ6 | Protein S100-A16 | S100A16 | 6 | 54.4 | 0 | 14.67 | 1.83 | 1.70 | 1.72 | NaN | NaN | 1.39 | NaN | 0.33 | 1.70 |
| Q96G03 | Phosphoglucomutase-2 | PGM2 | 9 | 20.1 | 0 | 118.8 | 0.98 | 0.90 | 1.46 | 1.13 | 0.77 | 0.91 | NaN | 1.48 | 0.84 |
| Q96HC4 | PDZ and LIM domain protein 5 | PDLIM5 | 8 | 19.5 | 0 | 17.45 | 1.15 | 1.16 | 1.25 | 1.43 | 1.40 | 1.34 | 1.08 | 1.07 | 0.98 |
| Q96HE7 | ERO1-like protein alpha | ERO1L | 16 | 50.2 | 0 | 199.2 | 1.27 | 1.09 | 1.22 | 1.04 | 1.30 | 1.28 | 1.23 | 1.19 | 1.19 |
| Q96HS1 | Serine/threonine-protein phosphatase PGAM5, mitochondrial | PGAM5 | 2 | 6.6 | 0 | 6.559 | 0.95 | 1.05 | 1.29 | 1.13 | NaN | 1.12 | 1.16 | 1.09 | 0.95 |
| Q96I24 | Far upstream element-binding protein 3 | FUBP3 | 6 | 14.5 | 0 | 5.955 | 0.86 | NaN | NaN | 3.55 | NaN | 0.80 | NaN | 1.99 | NaN |
| Q96I99 | Succinyl-CoA ligase [GDP-forming] subunit beta, | SUCLG2 | 12 | 39.4 | 0 | 41.82 | 0.96 | 0.96 | 1.08 | NaN | 0.99 | 0.94 | NaN | NaN | 0.93 |
| Q96IU4 | Alpha/beta hydrolase domain-containing protein 14B | ABHD14B | 5 | 31.4 | 0 | 10.08 | 0.88 | NaN | 0.80 | 1.47 | 1.00 | 0.75 | 0.97 | 2.10 | 1.15 |
| Q96IZ0 | PRKC apoptosis WT1 regulator protein | PAWR | 8 | 41.2 | 0 | 183.4 | 1.42 | 1.17 | 1.30 | 1.23 | 1.05 | 0.89 | 1.04 | 0.92 | 1.14 |
| Q96K17 | Transcription factor BTF3 homolog 4;Transcription factor | BTF3L4 | 2 | 36.7 | 0 | 41.8 | 0.58 | 0.86 | 0.45 | 1.22 | 1.40 | 1.33 | 1.00 | 0.97 | NaN |
| Q96KP4 | Cytosolic non-specific | CNDP2 | 12 | 39.6 | 0 | 73.86 | 0.90 | 0.94 | 0.88 | 0.71 | 1.00 | 0.88 | NaN | 0.71 | 0.88 |
| Q96N66 | Lysophospholipid acyltransferase 7 | MBOAT7 | 8 | 21.2 | 0 | 217.8 | 0.81 | 0.87 | 0.98 | 0.87 | 0.88 | 0.86 | 1.33 | 1.00 | 0.94 |
| Q96P70 | Importin-9 | IPO9 | 7 | 9.6 | 0 | 34.6 | 1.03 | 0.92 | NaN | NaN | 1.04 | NaN | NaN | NaN | 1.01 |
| Q96PK6 | RNA-binding protein 14 | RBM14 | 3 | 5.7 | 0 | 7.67 | 1.47 | NaN | NaN | 0.82 | NaN | 0.84 | NaN | NaN | 1.05 |
| Q96QK1 | Vacuolar protein sorting-associated protein 35 | VPS35 | 17 | 26.6 | 0 | 92.16 | 1.21 | 1.20 | 1.16 | 1.02 | 1.05 | 1.00 | 1.06 | 1.41 | 1.21 |
| Q96RS6 | NudC domain-containing protein | NUDCD1 | 11 | 24.4 | 0 | 41.26 | 1.06 | 0.84 | 0.91 | 0.93 | 0.95 | 0.92 | NaN | NaN | 0.92 |
| Q96T51 | RUN and FYVE domain-containing protein 1 | RUFY1 | 4 | 11.7 | 0 | 21.31 | NaN | 0.90 | 1.10 | NaN | 1.35 | 0.91 | 1.52 | 1.54 | 1.47 |
| Q96T76 | MMS19 nucleotide excision repair protein homolog | MMS19 | 5 | 7.1 | 0 | 5.006 | 0.39 | NaN | 2.36 | NaN | NaN | NaN | NaN | NaN | NaN |
| Q96TA1 | Niban-like protein 1 | FAM129B | 22 | 42 | 0 | 171 | 1.07 | 1.16 | 1.09 | 0.93 | 1.11 | 0.96 | 1.19 | 1.12 | 0.96 |
| Q99426 | Tubulin-folding cofactor B | TBCB | 7 | 38.9 | 0 | 26.2 | 0.93 | 0.94 | 0.95 | NaN | 0.93 | 0.88 | 1.19 | NaN | 0.92 |
| Q99436 | Proteasome subunit beta type-7;Proteasome subunit beta type | PSMB7 | 6 | 22.7 | 0 | 23.52 | 1.14 | 1.34 | NaN | 1.21 | 1.34 | 1.37 | 1.42 | 1.15 | 0.98 |
| Q99447 | Ethanolamine-phosphate cytidylyltransferase | PCYT2 | 3 | 10.3 | 0 | 42.53 | 0.61 | 0.59 | 0.78 | 0.79 | NaN | NaN | NaN | 0.68 | 0.70 |
| Q99459 | Cell division cycle 5-like protein | CDC5L | 6 | 11.1 | 0 | 5.361 | NaN | 0.80 | 1.22 | 0.60 | 0.69 | NaN | NaN | NaN | NaN |
| Q99460 | 26S proteasome non-ATPase regulatory subunit 1 | PSMD1 | 21 | 29.4 | 0 | 178.6 | 0.99 | 0.96 | 1.08 | 1.14 | 1.06 | 1.37 | 0.81 | 0.96 | 0.93 |
| Q99471 | Prefoldin subunit 5 | PFDN5 | 7 | 59.7 | 0 | 267.6 | 0.96 | 0.80 | 0.90 | 0.74 | 0.99 | 0.93 | 0.62 | 0.78 | 0.83 |
| Q99497 | Protein deglycase DJ-1 | PARK7 | 14 | 79.9 | 0 | 323.3 | 1.02 | 1.04 | 0.99 | 1.08 | 1.07 | 1.07 | 1.00 | 1.02 | 1.05 |
| Q99536 | Synaptic vesicle membrane protein VAT-1 homolog | VAT1 | 12 | 43.3 | 0 | 108.8 | 0.67 | 0.75 | 0.79 | 0.88 | 0.95 | 0.80 | 0.77 | 0.73 | 0.71 |

| | | | | | | | | | | | | | | | |
|---|---|---|---|---|---|---|---|---|---|---|---|---|---|---|---|
| Q99584 | Protein S100-A13 | S100A13 | 7 | 55.1 | 0 | 45.64 | 1.13 | 1.11 | 1.07 | 1.06 | 1.07 | 1.22 | 1.09 | 1.12 | 1.30 |
| Q99613 | Eukaryotic translation initiation factor 3 subunit C;Eukaryotic translation initiation factor 3 subunit C-like protein | EIF3C | 21 | 27.5 | 0 | 120.2 | 1.17 | 1.24 | 1.05 | 0.93 | 1.03 | 1.38 | 0.86 | 0.96 | 1.03 |
| Q99614 | Tetratricopeptide repeat protein 1 | TTC1 | 4 | 14.7 | 0 | 3.972 | NaN | NaN | NaN | 1.09 | NaN | 0.85 | NaN | NaN | 0.85 |
| Q99615 | DnaJ homolog subfamily C member 7 | DNAJC7 | 5 | 12.8 | 0 | 5.994 | 1.51 | 1.31 | 1.66 | NaN | 1.01 | 1.33 | NaN | NaN | 1.40 |
| Q99714 | 3-hydroxyacyl-CoA dehydrogenase type-2 | HSD17B10 | 8 | 49.4 | 0 | 111.2 | 0.98 | 0.96 | 0.98 | 1.18 | 0.97 | 1.11 | 1.22 | 0.98 | 0.93 |
| Q99733 | Nucleosome assembly protein 1-like 4 | NAP1L4 | 7 | 32 | 0 | 146.5 | 0.84 | 1.07 | 1.02 | 1.06 | 0.85 | 0.99 | 0.85 | 0.88 | 1.11 |
| Q99805 | Transmembrane 9 superfamily member 2 | TM9SF2 | 1 | 2.1 | 0.009 | 1.413 | NaN | 1.08 | NaN | NaN | 0.66 | 1.03 | NaN | NaN | 0.97 |
| Q99829 | Copine-1 | CPNE1 | 18 | 37.1 | 0 | 86.01 | 1.02 | 0.92 | 0.92 | 0.91 | 0.91 | 0.89 | 1.03 | 1.13 | 1.09 |
| Q99832 | T-complex protein 1 subunit eta | CCT7 | 24 | 58.7 | 0 | 323.3 | 1.04 | 1.07 | 1.08 | 1.04 | 1.03 | 1.08 | 0.93 | 0.99 | 0.93 |
| Q99961 | Endophilin-A2 | SH3GL1 | 10 | 32.6 | 0 | 27.16 | NaN | NaN | 1.14 | 0.86 | 0.84 | 0.95 | 0.89 | 0.93 | NaN |
| Q9BPX3 | Condensin complex subunit 3 | NCAPG | 5 | 6.6 | 0 | 4.415 | NaN | 1.02 | 0.89 | NaN | 1.09 | NaN | NaN | NaN | 0.59 |
| Q9BPX5 | Actin-related protein 2/3 complex subunit 5-like protein | ARPC5L | 4 | 58.8 | 0 | 13.7 | 1.19 | 1.07 | 1.29 | 1.05 | 0.97 | 1.26 | 0.96 | 0.95 | 1.06 |
| Q9BQG0 | Myb-binding protein 1A | MYBBP1A | 14 | 16 | 0 | 46.76 | 0.97 | 1.05 | 0.96 | 0.78 | 0.91 | 1.15 | 1.17 | 0.71 | 1.13 |
| Q9BR76 | Coronin-1B | CORO1B | 9 | 27.4 | 0 | 40.08 | 1.16 | NaN | 0.78 | 0.71 | 1.03 | 1.19 | 1.05 | 0.72 | 0.81 |
| Q9BRA2 | Thioredoxin domain-containing protein 17 | TXNDC17 | 5 | 43.1 | 0 | 39.19 | 1.18 | 1.11 | 1.19 | 1.11 | 1.08 | 1.02 | 1.23 | 1.18 | 1.20 |
| Q9BRX8 | Redox-regulatory protein FAM213A | FAM213A | 3 | 15.7 | 0.004 | 1.755 | 0.97 | 0.89 | 0.88 | 0.86 | 0.67 | 1.06 | NaN | NaN | NaN |
| Q9BS26 | Endoplasmic reticulum resident protein 44 | ERP44 | 7 | 20.9 | 0 | 39.01 | 1.38 | 1.96 | 1.49 | NaN | 1.17 | 1.36 | NaN | NaN | 1.19 |
| Q9BSJ8 | Extended synaptotagmin-1 | ESYT1 | 18 | 22.1 | 0 | 154.8 | 0.83 | 0.83 | 0.92 | 0.90 | 0.96 | 0.91 | 0.91 | 0.92 | 1.00 |
| Q9BT78 | COP9 signalosome complex subunit 4 | COPS4 | 5 | 18.7 | 0 | 5.045 | NaN | 1.01 | NaN | 6.46 | 0.56 | 1.13 | NaN | NaN | NaN |
| Q9BTT0 | Acidic leucine-rich nuclear phosphoprotein 32 family member E | ANP32E | 5 | 24.6 | 0 | 162.2 | 1.04 | 1.23 | 0.81 | 0.73 | 0.85 | 1.08 | NaN | 1.20 | 1.17 |
| Q9BTV4 | Transmembrane protein 43 | TMEM43 | 7 | 23.8 | 0 | 62.33 | 0.99 | 0.85 | NaN | 1.00 | 0.89 | 1.06 | NaN | NaN | 1.10 |
| Q9BUF5 | Tubulin beta-6 chain | TUBB6 | 8 | 59.9 | 0 | 304.9 | 0.60 | 1.01 | 0.76 | 0.75 | 0.67 | 0.63 | NaN | 1.07 | 1.15 |
| Q9BV40 | Vesicle-associated membrane protein 8 | VAMP8 | 4 | 45 | 0 | 12.99 | 0.72 | 0.68 | 0.76 | 0.69 | 0.90 | 0.77 | 0.58 | 0.58 | 0.87 |
| Q9BV86 | N-terminal Xaa-Pro-Lys N-methyltransferase 1;N-terminal Xaa-Pro-Lys N-methyltransferase 1, N-terminally processed | NTMT1 | 2 | 9.4 | 1E-03 | 2.167 | 0.94 | 1.08 | 1.22 | 0.99 | 0.93 | 0.80 | 1.03 | 0.96 | 1.05 |
| Q9BVC6 | Transmembrane protein 109 | TMEM109 | 3 | 12.3 | 0 | 5.785 | 0.97 | 1.08 | 0.98 | 0.99 | 1.26 | 1.31 | 1.60 | 1.23 | 0.90 |
| Q9BVG4 | Protein PBDC1 | PBDC1 | 4 | 21.9 | 0 | 3.377 | 0.70 | 0.97 | NaN | NaN | 1.05 | 0.57 | 1.16 | 1.03 | 0.77 |
| Q9BVK6 | Transmembrane emp24 domain-containing protein 9 | TMED9 | 3 | 15.7 | 0 | 96.99 | 1.46 | 1.17 | 1.06 | 1.03 | 1.39 | 1.27 | 1.77 | 1.37 | 1.36 |

| | | | | | | | | | | | | | | |
|---|---|---|---|---|---|---|---|---|---|---|---|---|---|---|
| Q9BW60 | Elongation of very long chain fatty acids protein 1 | ELOVL1 | 1 | 4.3 | 0 | 13.64 | 0.72 | NaN | NaN | 0.79 | 0.71 | 0.99 | NaN | NaN | NaN |
| Q9BWD1 | Acetyl-CoA acetyltransferase, cytosolic | ACAT2 | 12 | 50.6 | 0 | 323.3 | 0.74 | 0.74 | 0.66 | 0.80 | 0.87 | 0.82 | 0.98 | 0.99 | 0.98 |
| Q9BX68 | Histidine triad nucleotide-binding protein 2, mitochondrial | HINT2 | 2 | 21.5 | 0 | 4.774 | 0.98 | NaN | 1.19 | 0.73 | 1.01 | NaN | NaN | NaN | NaN |
| Q9BXJ9 | N-alpha-acetyltransferase 15, NatA auxiliary subunit | NAA15 | 6 | 8.3 | 0 | 24.1 | 0.48 | NaN | NaN | 0.93 | 1.12 | 0.94 | NaN | 0.82 | 0.66 |
| Q9BXP5 | Serrate RNA effector molecule homolog | SRRT | 6 | 10.4 | 0 | 9.477 | 0.87 | 0.89 | NaN | 1.25 | 0.90 | 1.02 | 0.68 | 1.31 | 1.04 |
| Q9BY32 | Inosine triphosphate pyrophosphatase | ITPA | 5 | 38.7 | 0 | 20.73 | 1.12 | 0.83 | 0.82 | NaN | 0.68 | 0.98 | NaN | NaN | 1.36 |
| Q9BZE4 | Nucleolar GTP-binding protein 1 | GTPBP4 | 3 | 7.4 | 0 | 5.088 | NaN | 0.51 | 0.50 | NaN | NaN | NaN | NaN | NaN | NaN |
| Q9BZL1 | Ubiquitin-like protein 5 | UBL5 | 2 | 28.8 | 0.002 | 1.903 | 0.74 | NaN | 0.61 | NaN | 1.38 | 2.74 | NaN | NaN | 0.85 |
| Q9BZQ8 | Protein Niban | FAM129A | 5 | 7.8 | 0 | 4.743 | 1.11 | NaN | 1.07 | NaN | NaN | 1.15 | NaN | NaN | 0.99 |
| Q9C002 | Normal mucosa of esophagus-specific gene 1 protein | NMES1 | 2 | 32.5 | 0 | 4.77 | 1.05 | 0.92 | 0.87 | 1.00 | 0.59 | 0.84 | 1.98 | 1.52 | 1.09 |
| Q9C0C2 | 182 kDa tankyrase-1-binding protein | TNKS1BP1 | 6 | 5.1 | 0 | 6.961 | 0.80 | 1.22 | NaN | 1.11 | NaN | NaN | NaN | 1.23 | NaN |
| Q9GZN8 | UPF0687 protein C20orf27 | C20orf27 | 2 | 13.8 | 0.008 | 1.517 | 1.11 | 0.93 | NaN | 1.05 | NaN | 1.22 | NaN | NaN | 0.87 |
| Q9GZP8 | Immortalization up-regulated protein | IMUP | 2 | 17 | 0 | 12.69 | 0.82 | NaN | 1.03 | NaN | NaN | 0.87 | NaN | NaN | NaN |
| Q9H074 | Polyadenylate-binding protein-interacting protein 1 | PAIP1 | 3 | 13.4 | 0 | 4.676 | 1.32 | NaN | 1.27 | 1.12 | 1.48 | 1.06 | NaN | NaN | NaN |
| Q9H0B6 | Kinesin light chain 2;Kinesin light chain 4 | KLC2 | 2 | 7.7 | 0 | 3.969 | 0.93 | 1.31 | 1.11 | NaN | 1.82 | 1.27 | NaN | NaN | 1.29 |
| Q9H0D6 | 5-3 exoribonuclease 2 | XRN2 | 7 | 12.4 | 0 | 13.64 | 1.26 | NaN | 0.92 | NaN | 1.27 | 1.52 | 1.27 | 1.68 | 1.36 |
| Q9H0U4 | Ras-related protein Rab-1B | RAB1B | 3 | 67.7 | 0 | 42.7 | 0.73 | 0.84 | 0.82 | 1.02 | 1.12 | 0.99 | 0.56 | 0.62 | 0.67 |
| Q9H1E3 | Nuclear ubiquitous casein and cyclin-dependent kinase substrate 1 | NUCKS1 | 3 | 13.2 | 0 | 8.121 | 0.55 | 0.58 | 0.59 | 0.69 | 0.88 | 0.69 | 0.97 | 1.14 | 1.10 |
| Q9H223 | EH domain-containing protein 4 | EHD4 | 5 | 14 | 0 | 14.2 | 0.79 | NaN | NaN | NaN | NaN | NaN | 1.32 | 1.48 | NaN |
| Q9H307 | Pinin | PNN | 7 | 14.1 | 0 | 10.58 | NaN | NaN | NaN | 1.14 | NaN | NaN | 1.00 | 1.38 | NaN |
| Q9H3H3 | UPF0696 protein C11orf68 | C11orf68 | 5 | 19.5 | 0 | 6.508 | 1.06 | NaN | NaN | 0.75 | NaN | 1.01 | 0.79 | 1.68 | 0.69 |
| Q9H3K6 | BolA-like protein 2 | BOLA2 | 5 | 72.1 | 0 | 23.71 | 1.22 | 1.18 | 1.19 | 1.25 | 1.20 | 1.04 | 1.14 | NaN | 1.20 |
| Q9H3N1 | Thioredoxin-related transmembrane protein 1 | TMX1 | 6 | 23.6 | 0 | 13.87 | 1.18 | 1.21 | 1.41 | 1.24 | 1.21 | 1.26 | 1.72 | 1.12 | 1.17 |
| Q9H3P7 | Golgi resident protein GCP60 | ACBD3 | 4 | 14 | 0 | 15.6 | NaN | 1.73 | NaN | NaN | 1.17 | 1.35 | 0.76 | NaN | 0.89 |
| Q9H444 | Charged multivesicular body protein 4b | CHMP4B | 6 | 33.9 | 0 | 244.6 | 1.47 | 1.26 | 1.26 | 1.19 | 1.21 | 1.01 | 1.26 | 0.97 | 1.07 |
| Q9H488 | GDP-fucose protein O-fucosyltransferase 1 | POFUT1 | 5 | 23.7 | 0 | 18.73 | 1.13 | 1.33 | 1.22 | 1.01 | 1.34 | 1.31 | NaN | NaN | 0.90 |
| Q9H4A4 | Aminopeptidase B | RNPEP | 14 | 28.8 | 0 | 186.1 | 0.97 | 1.31 | 1.28 | NaN | NaN | NaN | 1.21 | 1.11 | 1.42 |
| Q9H4L5 | Oxysterol-binding protein-related protein 3 | OSBPL3 | 10 | 16 | 0 | 41.37 | 0.93 | 1.00 | 1.06 | NaN | NaN | 0.96 | 0.80 | NaN | 0.91 |
| Q9H4M9 | EH domain-containing protein 1 | EHD1 | 17 | 51.3 | 0 | 258 | 0.92 | 0.98 | 0.89 | 0.96 | 0.97 | 1.05 | 1.03 | 0.92 | 0.93 |

| | | | | | | | | | | | | | | |
|---|---|---|---|---|---|---|---|---|---|---|---|---|---|---|
| Q9H6S3 | Epidermal growth factor receptor kinase substrate 8-like protein 2 | EPS8L2 | 15 | 36.6 | 0 | 307.5 | 1.05 | 0.87 | 0.85 | 0.89 | 0.78 | 0.93 | NaN | NaN | 1.10 |
| Q9H993 | Protein-glutamate O-methyltransferase | ARMT1 | 6 | 21.8 | 0 | 30.22 | 1.05 | 1.12 | 0.96 | NaN | 0.99 | 1.18 | NaN | 1.12 | 1.13 |
| Q9H9B4 | Sideroflexin-1 | SFXN1 | 7 | 36.6 | 0 | 19.15 | 0.80 | 0.98 | 0.67 | 0.99 | 1.66 | 0.91 | 0.49 | 0.53 | 1.02 |
| Q9H9H4 | Vacuolar protein sorting-associated protein 37B | VPS37B | 2 | 10.9 | 0 | 4.058 | NaN | 0.40 | 0.61 | NaN | NaN | NaN | NaN | NaN | NaN |
| Q9HAV4 | Exportin-5 | XPO5 | 9 | 10.6 | 0 | 26.49 | 0.86 | 0.80 | 0.77 | 1.14 | NaN | 0.97 | 1.03 | 1.01 | 1.12 |
| Q9HAV7 | GrpE protein homolog 1, mitochondrial | GRPEL1 | 3 | 15.2 | 0 | 5.862 | 1.72 | 1.46 | 1.24 | NaN | 0.82 | 1.11 | 0.77 | NaN | 0.97 |
| Q9HB71 | Calcyclin-binding protein | CACYBP | 20 | 76.3 | 0 | 206.3 | 1.20 | 1.33 | 1.24 | 1.26 | 1.13 | 1.17 | 0.81 | 0.84 | 1.06 |
| Q9HC07 | Transmembrane protein 165 | TMEM165 | 4 | 20.4 | 0 | 6.481 | 1.02 | 0.75 | 0.99 | NaN | NaN | 1.06 | NaN | NaN | NaN |
| Q9HDC9 | Adipocyte plasma membrane-associated protein | APMAP | 12 | 40.6 | 0 | 84.43 | 0.92 | 0.88 | 0.96 | 1.00 | 1.06 | 0.95 | 0.96 | 0.89 | 0.78 |
| Q9NP97 | Dynein light chain roadblock-type 1;Dynein light chain roadblock-type 2 | DYNLRB1 | 6 | 83.3 | 0 | 32.15 | 1.01 | 1.23 | 0.90 | 0.61 | NaN | 1.12 | NaN | NaN | 1.19 |
| Q9NPQ8 | Synembryn-A | RIC8A | 6 | 13.9 | 0 | 20.89 | 0.81 | 1.01 | NaN | NaN | NaN | 0.94 | NaN | NaN | 0.60 |
| Q9NQ50 | 39S ribosomal protein L40, mitochondrial | MRPL40 | 1 | 7.8 | 0.002 | 1.983 | NaN | 0.81 | 1.13 | NaN | NaN | NaN | NaN | NaN | NaN |
| Q9NQC3 | Reticulon-4 | RTN4 | 2 | 14.8 | 0 | 323.3 | 1.12 | 0.97 | 0.98 | 0.98 | 1.25 | 1.10 | 0.87 | 1.06 | 1.02 |
| Q9NQG5 | Regulation of nuclear pre-mRNA domain-containing protein 1B | RPRD1B | 5 | 22.7 | 0 | 36.25 | 1.22 | 1.03 | NaN | NaN | 1.21 | 1.09 | NaN | NaN | 0.95 |
| Q9NQR4 | Omega-amidase NIT2 | NIT2 | 5 | 21 | 0 | 19.37 | 1.81 | NaN | NaN | NaN | NaN | NaN | 1.51 | NaN | 1.69 |
| Q9NQW6 | Actin-binding protein anillin | ANLN | 15 | 16.5 | 0 | 104.1 | 0.81 | 0.99 | 1.03 | NaN | 1.53 | 1.50 | NaN | NaN | 0.72 |
| Q9NR12 | PDZ and LIM domain protein 7 | PDLIM7 | 7 | 17.3 | 0 | 6.777 | NaN | NaN | 3.16 | NaN | 0.99 | 0.89 | NaN | NaN | 2.03 |
| Q9NR30 | Nucleolar RNA helicase 2 | DDX21 | 6 | 12.4 | 0 | 53.19 | NaN | NaN | NaN | NaN | 1.39 | 1.01 | NaN | 0.60 | 0.96 |
| Q9NR31 | GTP-binding protein SAR1a | SAR1A | 7 | 42.9 | 0 | 47.6 | 1.11 | 0.69 | 0.79 | 1.05 | 1.18 | 1.02 | 0.97 | 1.07 | 0.92 |
| Q9NR45 | Sialic acid synthase | NANS | 9 | 36.5 | 0 | 99.79 | 0.95 | 0.84 | 0.80 | 1.41 | 1.25 | 1.11 | 1.00 | 0.97 | 0.87 |
| Q9NRN7 | L-aminoadipate-semialdehyde dehydrogenase-phosphopantetheinyl transferase | AASDHPP | 3 | 12.6 | 0 | 37.1 | 0.80 | NaN | 0.43 | 0.74 | 0.91 | 0.91 | 0.87 | 0.94 | 0.83 |
| Q9NRX4 | 14 kDa phosphohistidine phosphatase | PHPT1 | 3 | 37.6 | 0 | 59.16 | 1.16 | 1.15 | 1.19 | 1.10 | 1.12 | 1.04 | 1.20 | 0.96 | 1.01 |
| Q9NS69 | Mitochondrial import receptor subunit TOM22 homolog | TOMM22 | 5 | 66.2 | 0 | 31.81 | 0.90 | 0.75 | 0.94 | 0.68 | 1.02 | 0.97 | NaN | 0.40 | 0.72 |
| Q9NSD9 | Phenylalanine--tRNA ligase beta subunit | FARSB | 9 | 15.4 | 0 | 21.1 | 0.81 | 0.94 | 0.98 | 0.93 | 1.03 | 0.93 | 0.64 | 0.92 | 0.99 |
| Q9NSE4 | Isoleucine--tRNA ligase, mitochondrial | IARS2 | 15 | 20.3 | 0 | 26.7 | 0.95 | 0.76 | 0.70 | 1.04 | 0.88 | 0.85 | NaN | NaN | 0.92 |
| Q9NTZ6 | RNA-binding protein 12 | RBM12 | 2 | 2.4 | 0.008 | 1.531 | NaN | 2.03 | NaN | 1.01 | 0.97 | 1.12 | 2.03 | NaN | 0.70 |
| Q9NUQ6 | SPATS2-like protein | SPATS2L | 9 | 25.8 | 0 | 31.94 | 1.08 | 0.56 | 0.65 | 0.59 | 0.54 | 0.41 | 0.96 | 0.81 | 0.77 |
| Q9NUQ9 | Protein FAM49B | FAM49B | 9 | 45.4 | 0 | 53.41 | 1.22 | 1.11 | 1.25 | 1.00 | 0.98 | 1.14 | 0.72 | 0.78 | 1.24 |
| Q9NVI7 | ATPase family AAA domain-containing protein 3A | ATAD3A | 9 | 18.5 | 0 | 49.45 | 1.30 | 0.89 | 1.67 | 1.32 | 1.22 | 1.16 | 1.10 | 1.12 | 0.62 |
| Q9NVZ3 | Adaptin ear-binding coat-associated protein 2 | NECAP2 | 4 | 26.6 | 0 | 11.47 | 1.45 | 1.00 | NaN | NaN | 1.03 | 1.26 | NaN | NaN | 0.87 |

| | | | | | | | | | | | | | | |
|---|---|---|---|---|---|---|---|---|---|---|---|---|---|---|
| Q9NWV4 | UPF0587 protein C1orf123 | C1orf123 | 4 | 40 | 0 | 17.26 | 1.14 | 0.82 | 1.14 | 0.61 | 0.95 | NaN | NaN | 1.05 | 1.50 |
| Q9NX40 | OCIA domain-containing protein | OCIAD1 | 2 | 11.4 | 0 | 8.876 | NaN | 0.77 | NaN | NaN | 1.01 | 1.32 | NaN | NaN | NaN |
| Q9NXG2 | THUMP domain-containing protein 1 | THUMPD1 | 1 | 4.5 | 0 | 4.405 | 0.73 | 0.94 | 1.09 | 0.76 | NaN | NaN | 0.77 | NaN | 1.07 |
| Q9NY12 | H/ACA ribonucleoprotein complex subunit 1 | GAR1 | 1 | 8.3 | 0 | 5.417 | NaN | 0.97 | 0.98 | NaN | 0.95 | 0.92 | 0.95 | 1.00 | NaN |
| Q9NYL9 | Tropomodulin-3 | TMOD3 | 12 | 52 | 0 | 116.8 | 1.09 | 0.89 | 0.96 | 1.19 | 0.91 | 1.15 | NaN | 1.29 | 1.16 |
| Q9NYU2 | UDP-glucose:glycoprotein glucosyltransferase 1 | UGGT1 | 4 | 3.7 | 0 | 3.096 | 0.85 | 1.08 | 0.99 | NaN | NaN | 0.98 | NaN | NaN | 0.58 |
| Q9NZ01 | Very-long-chain enoyl-CoA reductase | TECR | 5 | 14.9 | 0 | 23.66 | 0.80 | 0.82 | 0.88 | 0.95 | 0.82 | 0.89 | 0.74 | NaN | 0.75 |
| Q9NZB2 | Constitutive coactivator of PPAR-gamma-like protein 1 | FAM120A | 9 | 11.9 | 0 | 56.65 | 0.99 | 1.11 | 1.25 | 1.11 | 1.09 | 1.02 | 1.17 | NaN | 1.02 |
| Q9NZL4 | Hsp70-binding protein 1 | HSPBP1 | 2 | 11.7 | 0 | 26.28 | NaN | NaN | NaN | NaN | 1.11 | 1.14 | NaN | NaN | NaN |
| Q9NZL9 | Methionine adenosyltransferase 2 subunit beta | MAT2B | 2 | 6.9 | 0.003 | 1.879 | NaN | NaN | 0.85 | 0.67 | 0.47 | NaN | 0.95 | NaN | 0.92 |
| Q9NZM1 | Myoferlin | MYOF | 84 | 52.6 | 0 | 323.3 | 0.72 | 0.75 | 0.83 | 0.90 | 0.89 | 0.89 | 0.70 | 0.88 | 1.02 |
| Q9NZN4 | EH domain-containing protein 2 | EHD2 | 12 | 35.7 | 0 | 42.68 | 0.70 | 0.89 | 0.59 | 0.94 | 0.85 | 0.78 | 1.08 | 0.70 | 0.94 |
| Q9P035 | Very-long-chain (3R)-3-hydroxyacyl-CoA dehydratase 3 | HACD3 | 4 | 18.8 | 0 | 10.81 | 0.69 | 0.54 | NaN | 1.14 | 0.71 | 0.76 | 1.20 | 0.84 | 1.06 |
| Q9P0L0 | Vesicle-associated membrane protein-associated protein A | VAPA | 13 | 61 | 0 | 144.2 | 0.97 | 0.85 | 1.00 | 0.90 | 1.07 | 0.98 | 0.65 | 0.81 | 0.62 |
| Q9P1F3 | Costars family protein ABRACL | ABRACL | 3 | 43.2 | 0 | 15.93 | 0.89 | 1.03 | NaN | NaN | 1.03 | 1.05 | 1.19 | 1.19 | 1.25 |
| Q9P258 | Protein RCC2 | RCC2 | 2 | 6.1 | 0 | 2.925 | NaN | NaN | 1.41 | NaN | NaN | NaN | 0.98 | 0.83 | NaN |
| Q9P287 | BRCA2 and CDKN1A-interacting protein | BCCIP | 3 | 18.2 | 0 | 11.54 | NaN | 0.98 | NaN | NaN | NaN | 0.81 | 0.84 | NaN | 1.47 |
| Q9P2E9 | Ribosome-binding protein 1 | RRBP1 | 42 | 36 | 0 | 323.3 | 0.92 | 0.87 | 0.92 | 0.93 | 0.81 | 0.86 | 0.63 | 0.90 | 0.86 |
| Q9P2J5 | Leucine--tRNA ligase, | LARS | 23 | 27.7 | 0 | 142.2 | 1.02 | 0.92 | 0.93 | 0.67 | 0.82 | 0.96 | NaN | 0.85 | 1.10 |
| Q9P2X0 | Dolichol-phosphate mannosyltransferase subunit 3 | DPM3 | 2 | 23.9 | 0.002 | 2.029 | 1.34 | 1.36 | 0.85 | NaN | NaN | NaN | NaN | NaN | 1.00 |
| Q9UBB4 | Ataxin-10 | ATXN10 | 7 | 17.5 | 0 | 106.2 | NaN | 0.90 | 1.03 | 1.08 | 1.20 | 1.04 | NaN | 0.61 | 0.76 |
| Q9UBE0 | SUMO-activating enzyme subunit 1;SUMO-activating enzyme subunit 1, N-terminally processed | SAE1 | 10 | 29.8 | 0 | 27.25 | 1.28 | 1.98 | 1.08 | 0.98 | 1.31 | 1.06 | 1.10 | 1.00 | 1.03 |
| Q9UBF2 | Coatomer subunit gamma-2 | COPG2 | 8 | 18.9 | 0 | 19.74 | NaN | NaN | 1.18 | NaN | 0.83 | 0.85 | NaN | NaN | NaN |
| Q9UBI6 | Guanine nucleotide-binding protein G(I)/G(S)/G(O) subunit gamma-12 | GNG12 | 3 | 56.9 | 0 | 96.94 | 0.93 | 1.03 | 0.80 | 1.08 | 1.02 | 0.99 | 1.07 | 0.94 | 1.18 |
| Q9UBM7 | 7-dehydrocholesterol reductase | DHCR7 | 4 | 10.5 | 0 | 8.396 | NaN | NaN | NaN | 0.98 | 1.09 | 0.86 | 1.86 | 1.43 | NaN |
| Q9UBQ7 | Glyoxylate reductase/hydroxypyruvate reductase | GRHPR | 8 | 40.2 | 0 | 100.4 | 0.85 | 0.62 | 0.79 | NaN | 0.94 | 0.71 | NaN | NaN | 0.82 |
| Q9UBR2 | Cathepsin Z | CTSZ | 3 | 12.9 | 0 | 67.04 | 1.38 | 1.05 | 1.49 | 1.14 | 1.97 | 1.21 | 1.26 | 1.15 | 1.04 |
| Q9UBS4 | DnaJ homolog subfamily B member 11 | DNAJB11 | 4 | 17.6 | 0 | 4.818 | NaN | NaN | 1.17 | NaN | NaN | NaN | 1.05 | 0.97 | NaN |
| Q9UBT2 | SUMO-activating enzyme subunit 2 | UBA2 | 9 | 22.8 | 0 | 13.46 | 0.78 | 0.79 | 0.71 | 0.79 | 1.33 | 1.10 | 1.10 | 0.73 | 0.90 |

| | | | | | | | | | | | | | | | |
|---|---|---|---|---|---|---|---|---|---|---|---|---|---|---|---|
| Q9UDY2 | Tight junction protein ZO-2 | TJP2 | 13 | 16.6 | 0 | 93.64 | 1.69 | 1.37 | NaN | 1.29 | 1.13 | NaN | 1.20 | NaN | NaN |
| Q9UG63 | ATP-binding cassette sub-family F member 2 | ABCF2 | 4 | 7.4 | 0 | 6.417 | 1.26 | 1.16 | 1.11 | NaN | 0.74 | 1.46 | NaN | 0.89 | 1.07 |
| Q9UGI8 | Testin | TES | 18 | 51.5 | 0 | 78.51 | 1.10 | 1.35 | 1.10 | NaN | 0.97 | 1.24 | 1.30 | NaN | 1.01 |
| Q9UH65 | Switch-associated protein 70 | SWAP70 | 12 | 20.5 | 0 | 16.95 | 0.89 | 0.97 | 0.83 | 1.27 | 1.18 | 1.02 | 1.12 | 1.30 | 1.05 |
| Q9UH99 | SUN domain-containing protein 2 | SUN2 | 3 | 8.6 | 0 | 5.686 | NaN | 1.92 | 1.77 | NaN | NaN | 1.42 | NaN | NaN | 1.41 |
| Q9UHB9 | Signal recognition particle subunit SRP68 | SRP68 | 11 | 25.2 | 0 | 116.8 | 0.89 | 0.78 | 0.98 | 0.98 | 0.90 | 0.85 | 0.96 | 0.78 | 0.56 |
| Q9UHD1 | Cysteine and histidine-rich domain-containing protein 1 | CHORDC1 | 9 | 47.9 | 0 | 52.51 | 1.64 | 1.86 | 1.65 | 1.42 | 1.70 | 1.65 | 0.88 | 0.69 | 0.82 |
| Q9UHD8 | Septin-9 | 9-Sep | 16 | 43.3 | 0 | 323.3 | 1.01 | 1.06 | 1.04 | 1.03 | 1.05 | 1.00 | 1.10 | 1.03 | 1.05 |
| Q9UHD9 | Ubiquilin-2 | UBQLN2 | 3 | 14.7 | 0 | 126.8 | 1.18 | 0.76 | 0.71 | 0.83 | 1.09 | 1.12 | NaN | NaN | 0.56 |
| Q9UHV9 | Prefoldin subunit 2 | PFDN2 | 7 | 51.9 | 0 | 74.29 | 1.04 | 1.11 | 1.19 | 1.06 | 1.01 | 1.17 | 1.32 | 0.93 | 1.09 |
| Q9UHY7 | Enolase-phosphatase E1 | ENOPH1 | 2 | 13 | 0 | 22.77 | 0.63 | 0.75 | 0.82 | NaN | NaN | 1.23 | 0.97 | NaN | 1.51 |
| Q9UI47 | Catenin alpha-3 | CTNNA3 | 1 | 3.5 | 0 | 4.184 | 0.93 | 0.95 | 0.94 | NaN | 1.06 | 1.02 | 0.97 | NaN | 1.00 |
| Q9UJS0 | Calcium-binding mitochondrial carrier protein Aralar2 | SLC25A13 | 4 | 7.9 | 5E-04 | 2.831 | 1.02 | 1.09 | 0.91 | NaN | NaN | NaN | NaN | NaN | NaN |
| Q9UJU6 | Drebrin-like protein | DBNL | 10 | 32.3 | 0 | 134.9 | 0.95 | 0.86 | 0.89 | 0.93 | 0.92 | 1.02 | 1.25 | NaN | 1.14 |
| Q9UJZ1 | Stomatin-like protein 2, mitochondrial | STOML2 | 9 | 49.4 | 0 | 41.07 | 1.47 | 0.93 | 0.97 | 1.35 | 1.06 | 0.93 | 1.61 | 1.08 | 1.30 |
| Q9UK76 | Hematological and neurological expressed 1 protein;Hematological and neurological expressed 1 protein, | HN1 | 5 | 64.9 | 0 | 290.3 | 1.28 | 1.29 | 1.44 | 0.76 | 1.13 | 0.93 | 1.46 | 1.55 | 1.53 |
| Q9UKY7 | Protein CDV3 homolog | CDV3 | 7 | 54.3 | 0 | 202.5 | 1.24 | 1.20 | 1.27 | 1.52 | 1.17 | 1.00 | 1.13 | 1.06 | 0.81 |
| Q9UL25 | Ras-related protein Rab-21 | RAB21 | 7 | 39.1 | 0 | 33.53 | 1.22 | 1.36 | 1.20 | NaN | 1.08 | 2.39 | NaN | NaN | 0.76 |
| Q9ULC4 | Malignant T-cell-amplified sequence 1 | MCTS1 | 4 | 40.9 | 0 | 23.36 | NaN | 0.70 | 0.68 | 0.67 | 0.81 | 0.66 | 0.92 | 0.50 | NaN |
| Q9ULC5 | Long-chain-fatty-acid--CoA ligase 5 | ACSL5 | 2 | 3.4 | 0 | 3.279 | 0.59 | NaN | NaN | NaN | 1.16 | 1.21 | NaN | NaN | NaN |
| Q9ULV4 | Coronin-1C | CORO1C | 3 | 47.9 | 0 | 225.9 | 0.80 | 0.82 | 0.83 | 0.81 | 0.87 | 0.90 | 0.85 | 0.95 | 0.89 |
| Q9ULW0 | Targeting protein for Xklp2 | TPX2 | 1 | 2.1 | 0 | 17.33 | NaN | NaN | 0.33 | 1.03 | 0.85 | 1.48 | 0.33 | 0.39 | 0.12 |
| Q9UM22 | Mammalian ependymin-related protein 1 | EPDR1 | 1 | 4.9 | 0.008 | 1.495 | NaN | 0.50 | 0.44 | 0.40 | NaN | 0.58 | NaN | NaN | NaN |
| Q9UMS4 | Pre-mRNA-processing factor 19 | PRPF19 | 12 | 35.1 | 0 | 163.5 | 1.24 | 1.00 | 1.09 | 0.95 | 0.87 | 0.87 | 1.01 | 0.97 | 0.90 |
| Q9UMX0 | Ubiquilin-1 | UBQLN1 | 2 | 16.3 | 0 | 10.82 | NaN | NaN | NaN | 1.18 | 1.17 | 1.03 | NaN | NaN | NaN |
| Q9UMX5 | Neudesin | NENF | 4 | 27.9 | 0 | 3.428 | NaN | NaN | NaN | 1.03 | NaN | 1.78 | 0.72 | NaN | NaN |
| Q9UN86 | Ras GTPase-activating protein-binding protein 2 | G3BP2 | 7 | 20.7 | 0 | 8.231 | 1.40 | 1.20 | 1.27 | 1.20 | 1.28 | 1.13 | 1.07 | 0.87 | 0.88 |
| Q9UNF0 | Protein kinase C and casein kinase substrate in neurons protein 2 | PACSIN2 | 9 | 21 | 0 | 6.422 | 1.14 | 1.13 | 0.91 | 1.15 | 1.00 | 1.20 | 1.01 | 1.37 | 0.94 |
| Q9UNF1 | Melanoma-associated antigen D2 | MAGED2 | 10 | 23.8 | 0 | 33.29 | 1.08 | NaN | 0.94 | NaN | NaN | 0.83 | 0.72 | NaN | 1.44 |
| Q9UNM6 | 26S proteasome non-ATPase regulatory subunit 13 | PSMD13 | 16 | 55.9 | 0 | 98.97 | 0.97 | 0.91 | 1.03 | 0.95 | 1.31 | 1.02 | 1.53 | 1.09 | 1.02 |
| Q9UNS2 | COP9 signalosome complex subunit 3 | COPS3 | 4 | 14.2 | 0 | 49.5 | 1.02 | NaN | 1.26 | NaN | 1.09 | 1.52 | NaN | 0.76 | 1.10 |

| | | | | | | | | | | | | | | | | |
|---|---|---|---|---|---|---|---|---|---|---|---|---|---|---|---|---|
| Q9UNW1 | Multiple inositol polyphosphate phosphatase 1 | MINPP1 | 1 | 3.1 | 0.001 | 2.717 | NaN | 1.26 | 1.20 | NaN | 1.28 | NaN | NaN | NaN | NaN |
| Q9UNZ2 | NSFL1 cofactor p47 | NSFL1C | 11 | 40.5 | 0 | 80.02 | 1.06 | 1.23 | 1.19 | 1.06 | 0.94 | 0.98 | 1.18 | 0.99 | 1.11 |
| Q9UQ35 | Serine/arginine repetitive matrix protein 2 | SRRM2 | 8 | 4.3 | 0 | 15.96 | 1.69 | 1.32 | 1.41 | 0.84 | 1.09 | NaN | 0.85 | 0.88 | 1.45 |
| Q9UQ80 | Proliferation-associated protein 2G4 | PA2G4 | 23 | 60.4 | 0 | 198.1 | 0.89 | 0.92 | 0.80 | 1.06 | 0.87 | 0.92 | 0.84 | 1.04 | 0.90 |
| Q9UQE7 | Structural maintenance of chromosomes protein 3 | SMC3 | 14 | 16.3 | 0 | 22.14 | NaN | NaN | NaN | 0.96 | 1.29 | NaN | NaN | NaN | NaN |
| Q9Y224 | UPF0568 protein C14orf166 | C14orf166 | 9 | 44.7 | 0 | 33.88 | 1.07 | 1.05 | 1.10 | 0.98 | 0.94 | 1.12 | 0.84 | 0.77 | 1.01 |
| Q9Y230 | RuvB-like 2 | RUVBL2 | 17 | 44.3 | 0 | 81.45 | 1.01 | 0.83 | 0.77 | 0.98 | 1.07 | 0.94 | 0.82 | 0.83 | 0.77 |
| Q9Y237 | Peptidyl-prolyl cis-trans isomerase NIMA-interacting 4 | PIN4 | 2 | 17.6 | 0 | 4.809 | 1.16 | 1.03 | 1.14 | 0.99 | 1.25 | 1.51 | 1.12 | NaN | NaN |
| Q9Y265 | RuvB-like 1 | RUVBL1 | 13 | 39.3 | 0 | 64.7 | 0.85 | 0.89 | 0.85 | 0.91 | 0.78 | 0.77 | 0.82 | 0.88 | 0.85 |
| Q9Y266 | Nuclear migration protein nudC | NUDC | 15 | 52.6 | 0 | 42.05 | 1.27 | 1.13 | 1.21 | 1.14 | 0.99 | 1.30 | 1.04 | 1.16 | 1.23 |
| Q9Y281 | Cofilin-2 | CFL2 | 5 | 62 | 0 | 15.19 | 0.80 | 0.85 | NaN | 0.80 | NaN | 0.91 | NaN | NaN | NaN |
| Q9Y285 | Phenylalanine--tRNA ligase alpha subunit | FARSA | 6 | 19.3 | 0 | 16.57 | 1.06 | 0.95 | 1.01 | NaN | 0.93 | 1.02 | NaN | NaN | 0.92 |
| Q9Y295 | Developmentally-regulated GTP-binding protein 1 | DRG1 | 3 | 11.2 | 0 | 8.405 | 0.97 | 1.03 | 1.06 | NaN | NaN | 0.97 | 1.33 | 1.21 | 0.95 |
| Q9Y2A7 | Nck-associated protein 1 | NCKAP1 | 11 | 15 | 0 | 28.87 | 0.87 | 0.97 | 0.79 | 0.95 | 0.99 | 0.84 | NaN | 0.84 | 0.99 |
| Q9Y2D5 | A-kinase anchor protein 2 | AKAP2 | 5 | 8.5 | 0 | 3.645 | NaN | NaN | NaN | NaN | 2.38 | 4.45 | 0.86 | NaN | NaN |
| S6FW71 | GDP-fucose protein O-fucosyltransferase 2 | POFUT2 | 1 | 6.6 | 0.008 | 1.543 | 1.35 | 0.98 | 0.85 | NaN | 0.95 | NaN | NaN | NaN | 1.12 |
| Q9Y2Q3 | Glutathione S-transferase kappa | GSTK1 | 5 | 28.8 | 0 | 97.45 | 0.64 | 0.35 | NaN | 0.26 | 0.74 | 0.87 | NaN | 0.67 | 0.72 |
| Q9Y2R0 | Cytochrome c oxidase assembly factor 3 homolog, mitochondrial | COA3 | 2 | 17.9 | 0.001 | 2.758 | 1.63 | 1.38 | 1.32 | NaN | NaN | 1.40 | NaN | NaN | NaN |
| Q9Y2S6 | Translation machinery-associated protein 7 | TMA7 | 1 | 15.6 | 0.001 | 2.087 | 1.18 | 1.30 | 1.26 | 1.40 | 1.13 | 1.19 | 0.85 | 0.94 | NaN |
| Q9Y2T2 | AP-3 complex subunit mu-1 | AP3M1 | 2 | 12.9 | 0 | 6.401 | 0.88 | NaN | 1.00 | NaN | NaN | 1.37 | NaN | NaN | 0.92 |
| Q9Y2V2 | Calcium-regulated heat stable protein 1 | CARHSP1 | 3 | 42.9 | 0 | 14.44 | 1.01 | 0.81 | 0.94 | 0.91 | 1.07 | 0.94 | NaN | 0.98 | 0.96 |
| Q9Y2X3 | Nucleolar protein 58 | NOP58 | 6 | 16.3 | 0 | 8.944 | 1.11 | 1.16 | NaN | NaN | NaN | NaN | NaN | NaN | 1.46 |
| Q9Y2Z0 | Suppressor of G2 allele of SKP1 homolog | SUGT1 | 7 | 28.5 | 0 | 46.75 | NaN | 1.04 | 1.25 | 0.67 | 1.05 | NaN | NaN | 0.86 | NaN |
| Q9Y305 | Acyl-coenzyme A thioesterase 9, mitochondrial | ACOT9 | 14 | 33.9 | 0 | 44.3 | 0.78 | 0.79 | 0.91 | 0.87 | 1.02 | 0.99 | 1.13 | 0.74 | 0.97 |
| Q9Y333 | U6 snRNA-associated Sm-like protein LSm2 | LSM2 | 4 | 67.4 | 0 | 25.05 | 1.17 | 1.36 | 1.31 | NaN | NaN | NaN | NaN | NaN | 1.31 |
| Q9Y376 | Calcium-binding protein 39 | CAB39 | 4 | 14.7 | 0 | 6.151 | 1.53 | NaN | NaN | NaN | 0.98 | 0.80 | NaN | NaN | 0.81 |
| Q9Y383 | Putative RNA-binding protein Luc7-like 2;Putative RNA-binding protein Luc7-like 1 | LUC7L2 | 10 | 28.8 | 0 | 56.28 | 0.81 | 0.91 | 0.82 | 0.88 | 0.76 | 0.94 | 0.76 | 0.82 | 0.77 |
| Q9Y3A6 | Transmembrane emp24 domain-containing protein 5 | TMED5 | 2 | 10 | 5E-04 | 2.87 | NaN | NaN | NaN | 1.04 | NaN | NaN | 0.99 | 0.88 | NaN |
| Q9Y3B4 | Splicing factor 3B subunit 6 | SF3B6 | 4 | 38.4 | 0 | 4.67 | NaN | NaN | 0.66 | 0.98 | 0.99 | 1.02 | NaN | 0.47 | NaN |
| Q9Y3C1 | Nucleolar protein 16 | NOP16 | 1 | 8.4 | 0.007 | 1.672 | 1.30 | 1.17 | NaN | NaN | 1.64 | 1.39 | NaN | NaN | NaN |

| | | | | | | | | | | | | | | | | |
|---|---|---|---|---|---|---|---|---|---|---|---|---|---|---|---|---|
| Q9Y3C6 | Peptidyl-prolyl cis-trans isomerase-like 1 | PPIL1 | 5 | 35.5 | 0 | 12.29 | 1.02 | 0.95 | 1.01 | 1.31 | 0.99 | 0.88 | 0.98 | 1.31 | 0.78 |
| Q9Y3D0 | Mitotic spindle-associated MMXD complex subunit MIP18 | FAM96B | 3 | 39.3 | 0 | 20.23 | NaN | NaN | 0.83 | NaN | NaN | 1.28 | 2.19 | NaN | 1.71 |
| Q9Y3D6 | Mitochondrial fission 1 protein | FIS1 | 2 | 15.8 | 0 | 37.27 | NaN | NaN | 1.03 | NaN | 1.15 | 0.83 | 1.01 | 0.96 | NaN |
| Q9Y3F4 | Serine-threonine kinase receptor-associated protein | STRAP | 17 | 63.4 | 0 | 56.24 | 0.97 | 1.03 | 0.93 | NaN | 1.20 | 1.22 | 1.03 | 1.01 | 0.98 |
| Q9Y3I0 | tRNA-splicing ligase RtcB | RTCB | 18 | 46.3 | 0 | 110.9 | 0.87 | 0.66 | 0.97 | NaN | 1.11 | 0.61 | 1.60 | 0.95 | 0.65 |
| Q9Y3U8 | 60S ribosomal protein L36 | RPL36 | 4 | 27.6 | 0 | 8.096 | 0.71 | 0.81 | 0.85 | 0.86 | 0.85 | 0.86 | NaN | 1.16 | 0.72 |
| Q9Y490 | Talin-1 | TLN1 | 107 | 63.3 | 0 | 323.3 | 1.05 | 1.05 | 1.06 | 1.07 | 1.02 | 1.04 | 1.01 | 0.99 | 1.00 |
| V9GZ56 | U6 snRNA-associated Sm-like protein LSm4 | LSM4 | 2 | 11.8 | 0 | 3.31 | 0.61 | 0.69 | 0.69 | 1.08 | 0.69 | 0.92 | NaN | NaN | 0.49 |
| Q9Y512 | Sorting and assembly machinery component 50 homolog | SAMM50 | 3 | 8.3 | 0.001 | 2.451 | NaN | 1.17 | 0.97 | NaN | 1.66 | 1.13 | NaN | NaN | 1.51 |
| Q9Y570 | Protein phosphatase methylesterase 1 | PPME1 | 13 | 44.6 | 0 | 102.6 | 1.28 | 1.00 | 1.26 | 1.20 | 1.13 | 1.20 | 0.88 | 0.73 | NaN |
| Q9Y5B9 | FACT complex subunit SPT16 | SUPT16H | 20 | 23 | 0 | 32.52 | 0.85 | 1.10 | 0.87 | 0.69 | 0.99 | 0.88 | 1.36 | 0.94 | 0.90 |
| Q9Y5J1 | U3 small nucleolar RNA-associated protein 18 homolog | UTP18 | 3 | 9 | 0 | 2.962 | NaN | 1.41 | NaN | NaN | 0.99 | NaN | 0.77 | NaN | 1.84 |
| Q9Y5K6 | CD2-associated protein | CD2AP | 7 | 16.6 | 0 | 17.97 | NaN | 1.00 | 1.26 | 0.55 | 0.61 | 0.90 | 1.05 | 0.65 | 1.21 |
| Q9Y5L4 | Mitochondrial import inner membrane translocase subunit Tim13 | TIMM13 | 4 | 52.6 | 0 | 12.83 | 1.56 | 1.05 | 0.85 | 1.15 | 1.27 | NaN | 0.99 | 0.85 | 0.39 |
| Q9Y5S9 | RNA-binding protein 8A | RBM8A | 4 | 27.6 | 0 | 37.23 | 1.05 | 1.13 | 1.17 | 1.08 | 0.97 | 1.17 | 0.81 | 0.86 | 1.15 |
| Q9Y5X3 | Sorting nexin-5 | SNX5 | 2 | 6.7 | 0.008 | 1.559 | 1.18 | 1.03 | 1.05 | NaN | 0.92 | 1.05 | NaN | NaN | 0.88 |
| Q9Y617 | Phosphoserine aminotransferase | PSAT1 | 8 | 25.9 | 0 | 7.437 | NaN | NaN | 0.71 | 0.72 | 0.39 | 0.52 | 0.52 | 0.62 | 0.69 |
| Q9Y678 | Coatomer subunit gamma-1 | COPG1 | 20 | 35.5 | 0 | 112.3 | 1.05 | 1.03 | 1.08 | 0.95 | 1.01 | 1.11 | 1.13 | 1.08 | 1.00 |
| Q9Y696 | Chloride intracellular channel protein 4 | CLIC4 | 12 | 62.5 | 0 | 228.5 | 1.02 | 0.88 | 1.23 | 0.96 | 0.95 | 1.01 | 0.97 | 1.00 | 1.16 |
| Q9Y6C9 | Mitochondrial carrier homolog 2 | MTCH2 | 6 | 25.4 | 0 | 30.75 | 1.54 | 1.32 | 1.07 | 0.82 | 1.03 | 1.09 | 0.75 | 0.90 | NaN |
| Q9Y6G9 | Cytoplasmic dynein 1 light intermediate chain 1 | DYNC1LI1 | 7 | 23.9 | 0 | 15.48 | 1.15 | 0.94 | 1.24 | 0.65 | 0.90 | 0.91 | 1.05 | 1.07 | 0.93 |
| Q9Y6N5 | Sulfide:quinone oxidoreductase, mitochondrial | SQRDL | 8 | 22.4 | 0 | 13.52 | NaN | NaN | NaN | 0.96 | NaN | 1.06 | NaN | NaN | NaN |
| R4GNH3 | 26S protease regulatory subunit 6A | PSMC3 | 17 | 51.8 | 0 | 310.2 | 0.97 | 1.00 | 1.17 | 0.95 | 0.97 | 1.01 | 1.05 | 0.99 | 0.78 |

Supplementary Table S2 The results of phosphoproteomic analysis

| Protein ID | Protein names | Gene names | Phospho-site positions | Amino acid | Localization prob | Score | Ratio of TGF-β 1+SFN / Con_1 | Ratio of TGF-β 1+SFN / Con_2 | Ratio of TGF-β 1+SFN / Con_3 | Ratio of SFN / Con_1 | Ratio of SFN / Con_2 | Ratio of SFN / Con_3 | Ratio of TGF-β1/ Con_1 | Ratio of TGF-β1/ Con_2 | Ratio of TGF-β1/ Con_3 |
|---|---|---|---|---|---|---|---|---|---|---|---|---|---|---|---|
| A0A1B0GWH8 | Battenin | CLN3 | 12 | S | 1 | 112 | 0.79 | NaN | NaN | NaN | NaN | 0.90 | 1.50 | 1.06 | 1.88 |
| A0A024R4E5 | Vigilin | HDLBP | 31 | S | 1 | 228.5 | 1.41 | 1.32 | 1.48 | 1.73 | 1.36 | 1.24 | 1.28 | 1.42 | 1.17 |
| A0A0G2JL47 | Large proline-rich protein | BAT3 | 113 | S | 0.997 | 149.4 | NaN | 0.73 | NaN | 0.92 | 0.96 | NaN | 1.05 | 0.82 | 0.90 |
| A0A0G2JL47 | Large proline-rich protein | BAT3 | 958 | S | 1 | 123.1 | NaN | 0.85 | 0.72 | 0.98 | NaN | NaN | 0.94 | NaN | 0.59 |
| A0A0G2JL47 | Large proline-rich protein | BAT3 | 967 | S | 1 | 261 | 0.80 | 0.90 | 0.99 | 1.13 | 1.21 | 1.10 | 0.88 | 0.87 | 0.73 |
| A0A075B784 | Ubiquitin carboxyl-terminal hydrolase;Ubiquitin carboxyl-terminal hydrolase 36 | USP36 | 742 | S | 0.998 | 47.06 | 1.36 | 1.32 | 1.04 | 0.67 | 1.03 | 1.01 | NaN | 1.19 | 1.00 |
| A0A075B784 | Ubiquitin carboxyl-terminal hydrolase;Ubiquitin carboxyl-terminal hydrolase 36 | USP36 | 807 | S | 0.838 | 122.7 | 0.64 | 0.72 | NaN | NaN | NaN | NaN | NaN | NaN | NaN |
| A0A087WXA6 | GRIP1-associated protein 1 | GRIPAP1 | 271 | S | 0.968 | 181.6 | 1.75 | 1.47 | 1.30 | 1.52 | NaN | 0.98 | 1.01 | NaN | 0.80 |
| H0YL34 | Synemin | SYNM | 758 | S | 1 | 78.91 | 1.35 | 0.99 | 1.43 | 0.81 | 1.67 | 0.49 | 1.90 | NaN | NaN |
| H0YL34 | Synemin | SYNM | 763 | S | 1 | 78.91 | 1.35 | 1.95 | 1.43 | 1.14 | 0.87 | NaN | 0.65 | 0.76 | NaN |
| G3V2E8 | Alpha-actinin-1;Alpha-actinin-2;Alpha-actinin-3;Alpha-actinin-4 | ACTN1 | 75 | S | 0.996 | 98.63 | 0.35 | 0.36 | NaN | 0.88 | 0.91 | 0.79 | 1.33 | 1.29 | 1.41 |
| A0A087WT18 | Putative monooxygenase p33MONOX | KIAA1191 | 102 | S | 0.633 | 95.52 | NaN | NaN | NaN | NaN | 0.63 | 0.59 | NaN | NaN | NaN |
| A0A087WTB8 | Ubiquitin carboxyl-terminal hydrolase;Ubiquitin carboxyl-terminal hydrolase isozyme L3 | UCHL3 | 90 | S | 0.666 | 60.35 | NaN | NaN | NaN | 1.22 | NaN | 1.56 | NaN | NaN | NaN |
| A0A087WTB8 | Ubiquitin carboxyl-terminal hydrolase;Ubiquitin carboxyl-terminal hydrolase isozyme L3 | UCHL3 | 94 | S | 0.883 | 108.7 | 0.96 | 0.87 | NaN | NaN | NaN | NaN | NaN | NaN | NaN |
| A0A087WTP3 | Far upstream element-binding protein 2 | KHSRP | 480 | S | 1 | 105.1 | 1.78 | 1.47 | 1.67 | 2.73 | 2.38 | 2.67 | NaN | 0.62 | 0.83 |
| A0A087WTP3 | Far upstream element-binding protein 2 | KHSRP | 181 | S | 0.998 | 121.4 | NaN | NaN | 1.46 | 1.79 | 1.85 | 1.69 | 0.95 | 1.11 | 0.96 |
| A0A087WTQ7 | DNA damage-binding protein | DDB2 | 26 | S | 0.851 | 73.27 | NaN | NaN | 0.57 | 0.83 | 0.46 | NaN | 1.72 | 1.62 | NaN |
| Q1JUQ3 | Peptidyl-prolyl cis-trans isomerase;Peptidyl-prolyl cis-trans isomerase FKBP1A | FKBP12-Exin | 9 | S | 0.999 | 153.9 | NaN | 1.58 | NaN | 2.63 | 2.17 | 2.53 | 0.84 | NaN | NaN |
| A0A087WTS8 | Heat shock 70 kDa protein 4 | HSPA4 | 76 | S | 1 | 61.42 | 1.32 | 1.09 | 1.38 | 1.34 | 1.23 | NaN | NaN | NaN | NaN |
| Q5JWB9 | Transmembrane protein 230 | TMEM230 | 24 | S | 0.758 | 75.02 | NaN | 3.61 | NaN | NaN | NaN | NaN | 2.24 | 3.27 | NaN |
| A0A087WTU3 | Testis-expressed sequence 264 protein | TEX264 | 170 | S | 0.993 | 172.6 | NaN | NaN | 0.36 | 0.68 | 0.63 | NaN | 0.33 | 0.33 | 0.32 |
| A0A087WVR3 | E3 ubiquitin-protein ligase UHRF1 | UHRF1 | 104 | S | 0.856 | 126.4 | 0.68 | NaN | 0.38 | NaN | NaN | NaN | 0.59 | NaN | NaN |

| | | | | | | | | | | | | | | | |
|---|---|---|---|---|---|---|---|---|---|---|---|---|---|---|---|
| E9PS08 | UBX domain-containing protein 1 | UBXN1 | 58 | S | 0.703 | 163.7 | NaN | NaN | 1.50 | NaN | NaN | 1.70 | 1.11 | 1.29 | 1.18 |
| E7ER32 | MKL/myocardin-like protein 1 | MKL1 | 454 | S | 0.994 | 172.9 | 1.26 | 1.45 | NaN | NaN | NaN | NaN | NaN | NaN | NaN |
| A0A0G2JNN3 | Leukocyte receptor cluster member 8 | LENG8 | 203 | S | 1 | 79.71 | NaN | 0.94 | 0.87 | 1.12 | 1.24 | 1.20 | 0.74 | 0.71 | 0.74 |
| A0A087WUT6 | Eukaryotic translation initiation factor 5B | EIF5B | 1168 | S | 1 | 83.05 | 1.11 | 1.12 | NaN | 2.18 | 1.34 | 1.56 | NaN | 0.85 | 0.61 |
| A0A087WUT6 | Eukaryotic translation initiation factor 5B | EIF5B | 107 | S | 1 | 122.2 | 0.63 | 0.72 | 0.71 | 1.07 | 0.93 | 0.76 | 0.85 | 0.81 | 0.84 |
| A0A087WUT6 | Eukaryotic translation initiation factor 5B | EIF5B | 113 | S | 1 | 430.8 | 1.01 | 0.98 | 0.90 | 0.96 | 0.86 | 0.94 | 1.08 | 1.00 | 1.07 |
| A0A087WUT6 | Eukaryotic translation initiation factor 5B | EIF5B | 164 | S | 1 | 113.7 | 0.77 | 0.76 | 1.05 | 0.93 | 0.95 | 0.98 | 0.73 | 0.85 | NaN |
| A0A087WUT6 | Eukaryotic translation initiation factor 5B | EIF5B | 214 | S | 1 | 202.2 | 1.28 | 1.34 | 1.31 | 1.30 | 1.33 | 1.27 | 1.46 | 1.38 | 1.35 |
| A0A087WUT6 | Eukaryotic translation initiation factor 5B | EIF5B | 135 | S | 0.999 | 186.9 | 0.70 | 0.71 | 0.68 | 0.35 | 0.44 | 0.36 | 0.43 | 0.62 | 0.61 |
| A0A087WUT6 | Eukaryotic translation initiation factor 5B | EIF5B | 137 | S | 1 | 186.9 | 0.70 | 0.71 | 0.68 | 0.97 | 1.02 | 0.94 | 0.64 | 0.47 | 0.61 |
| A0A087X0G7 | NF-kappa-B essential modulator | IKBKG | 379 | S | 1 | 99.53 | 2.27 | NaN | 1.29 | 1.97 | NaN | 1.45 | NaN | NaN | NaN |
| A0A087WUZ3 | Spectrin beta chain, non-erythrocytic 1 | SPTBN1 | 2342 | S | 0.77 | 240.2 | 1.12 | NaN | NaN | NaN | NaN | NaN | 1.15 | NaN | 1.11 |
| A0A087WUZ3 | Spectrin beta chain, non-erythrocytic 1 | SPTBN1 | 2343 | S | 0.973 | 268.1 | 0.81 | 1.02 | 1.10 | 1.62 | 1.20 | 1.60 | NaN | NaN | NaN |
| A0A087WUZ3 | Spectrin beta chain, non-erythrocytic 1 | SPTBN1 | 2166 | S | 0.993 | 118.2 | NaN | 0.65 | NaN | 0.79 | 0.65 | 0.76 | 0.97 | 1.00 | 0.96 |
| A0A087WUZ3 | Spectrin beta chain, non-erythrocytic 1 | SPTBN1 | 2167 | S | 0.993 | 181.2 | 0.92 | 0.76 | 0.75 | 0.76 | 0.78 | 0.75 | 0.63 | 0.67 | 0.64 |
| A0A087WUZ3 | Spectrin beta chain, non-erythrocytic 1 | SPTBN1 | 2171 | S | 1 | 168.8 | 0.80 | 0.76 | 0.77 | 0.76 | 0.78 | 0.75 | 0.61 | 0.61 | 0.64 |
| A0A087WUZ3 | Spectrin beta chain, non-erythrocytic 1 | SPTBN1 | 2162 | S | 0.851 | 143.3 | NaN | 0.63 | NaN | 0.70 | 0.75 | 0.70 | 1.12 | 1.11 | 0.96 |
| A0A087WUZ3 | Spectrin beta chain, non-erythrocytic 1 | SPTBN1 | 2163 | S | 0.971 | 181.2 | 0.63 | 0.61 | 0.60 | 0.70 | 0.63 | 0.76 | 1.07 | 1.02 | 0.96 |
| A0A087WUZ3 | Spectrin beta chain, non-erythrocytic 1 | SPTBN1 | 2104 | S | 1 | 174.7 | 0.67 | 0.72 | 0.61 | 0.72 | 0.73 | 0.70 | 0.89 | 0.86 | 0.91 |
| A0A087WVA8 | Testis-expressed sequence 2 protein | TEX2 | 266 | S | 0.992 | 116.8 | NaN | NaN | 1.22 | 1.39 | NaN | 1.35 | 0.83 | 0.90 | NaN |
| K7EK53 | Eukaryotic translation initiation factor 3 subunit K | EIF3K | 130 | S | 0.989 | 124.3 | 1.24 | 1.50 | NaN | 0.96 | 1.36 | NaN | 0.71 | 0.90 | NaN |
| A0A087WVF1 | Uncharacterized protein C18orf25 | C18orf25 | 66 | S | 0.985 | 88.29 | 1.35 | 1.38 | 1.33 | 1.04 | 1.01 | 1.00 | 1.85 | 1.74 | 1.99 |
| A0A087WVF1 | Uncharacterized protein C18orf25 | C18orf25 | 145 | S | 0.919 | 49.07 | NaN | NaN | 2.28 | 2.00 | NaN | NaN | 1.91 | 2.04 | 1.93 |
| A0A087WVF7 | Intersectin-2 | ITSN2 | 867 | S | 0.988 | 69.58 | NaN | NaN | 1.39 | 1.70 | NaN | 1.49 | NaN | 1.33 | NaN |
| A0A087WVF7 | Intersectin-2 | ITSN2 | 872 | S | 0.955 | 139.2 | 1.06 | 1.05 | 0.95 | 0.94 | 1.00 | 0.70 | 0.96 | 1.12 | NaN |

| | | | | | | | | | | | | | | | |
|---|---|---|---|---|---|---|---|---|---|---|---|---|---|---|---|
| H7C4X9 | Protein kinase C-binding protein 1 | ZMYND8 | 595 | S | 1 | 60.03 | NaN | 0.70 | 0.53 | NaN | NaN | NaN | 1.01 | NaN | 0.89 |
| H7C4X9 | Protein kinase C-binding protein 1 | ZMYND8 | 474 | S | 1 | 78.84 | 1.01 | 1.33 | 1.16 | NaN | 1.35 | 2.10 | 0.87 | 0.87 | 0.98 |
| H7C4X9 | Protein kinase C-binding protein 1 | ZMYND8 | 417 | S | 0.973 | 111.6 | 0.45 | 0.46 | 0.48 | 0.57 | NaN | 0.56 | 0.97 | 1.19 | 1.13 |
| H7C4X9 | Protein kinase C-binding protein 1 | ZMYND8 | 683 | S | 0.991 | 82.87 | 0.72 | 0.65 | NaN | 0.81 | NaN | 0.83 | 0.97 | 0.96 | 0.98 |
| E7ET14 | Lysine-specific demethylase PHF2 | PHF2 | 95 | S | 0.998 | 76.21 | 1.02 | 0.81 | NaN | 1.05 | 1.55 | NaN | 0.89 | 1.30 | 0.97 |
| E7ET14 | Lysine-specific demethylase PHF2 | PHF2 | 269 | S | 0.667 | 75.51 | NaN | NaN | 0.23 | 0.72 | 0.93 | 0.76 | 0.78 | 0.38 | NaN |
| E7ET14 | Lysine-specific demethylase PHF2 | PHF2 | 271 | S | 0.667 | 75.51 | NaN | NaN | 0.23 | 0.72 | 0.93 | 0.76 | 0.78 | 0.38 | NaN |
| E7ET14 | Lysine-specific demethylase PHF2 | PHF2 | 272 | S | 0.667 | 75.51 | NaN | NaN | 0.23 | 0.72 | 0.93 | 0.76 | 0.78 | 0.38 | NaN |
| A0A087WW66 | 26S proteasome non-ATPase regulatory subunit 1 | PSMD1 | 315 | S | 1 | 84.95 | NaN | 0.95 | 0.95 | 1.04 | 1.08 | 1.22 | 1.01 | 0.98 | 1.26 |
| A0A087WWA3 | Kinesin-like protein;Kinesin-like protein KIF1B | KIF1B | 1428 | S | 0.94 | 71.56 | 0.48 | NaN | NaN | 0.75 | 0.79 | 1.06 | 0.51 | 0.54 | NaN |
| A0A087WWA3 | Kinesin-like protein;Kinesin-like protein KIF1B | KIF1B | 1031 | S | 1 | 102.4 | 1.19 | 0.94 | NaN | NaN | NaN | NaN | 1.67 | 1.20 | 1.38 |
| A0A087WWC8 | Myelin expression factor 2 | MYEF2 | 17 | S | 0.986 | 152.1 | 0.85 | 1.03 | 0.93 | 1.09 | NaN | 1.00 | 1.20 | 1.37 | 1.14 |
| A0A087WWF4 | Kanadaptin | SLC4A1AP | 82 | S | 1 | 238 | 0.54 | 0.88 | 0.57 | 0.98 | 0.86 | 0.87 | 0.35 | 0.43 | 0.47 |
| M0R2H0 | Leucine-rich repeat-containing protein C10orf11 | C10orf11 | 9 | S | 1 | 66.54 | NaN | NaN | 1.21 | 1.66 | 1.61 | 1.92 | 1.52 | 1.41 | 1.65 |
| M0R2H0 | Leucine-rich repeat-containing protein C10orf11 | C10orf11 | 10 | S | 1 | 66.54 | NaN | NaN | 1.21 | 1.66 | 1.61 | 1.92 | 1.52 | 1.41 | 1.65 |
| A0A087WWJ1 | DNA mismatch repair protein Msh6 | MSH6 | 251 | S | 0.996 | 129.6 | NaN | 0.40 | 0.28 | 0.49 | 0.53 | 0.45 | 0.63 | 0.63 | 0.61 |
| A0A499FJY3 | Putative RNA-binding protein 15 | RBM15 | 626 | S | 1 | 189.6 | NaN | 0.87 | 1.08 | 0.84 | 0.91 | NaN | 1.11 | 1.10 | 0.97 |
| A0A499FJY3 | Putative RNA-binding protein 15 | RBM15 | 630 | S | 1 | 189.6 | NaN | 0.87 | 1.08 | 0.84 | 0.91 | NaN | 1.11 | 1.10 | 0.97 |
| A0A499FJY3 | Putative RNA-binding protein 15 | RBM15 | 656 | S | 1 | 77.32 | 1.11 | 0.91 | 1.03 | 0.83 | 0.89 | 0.57 | 0.76 | 1.27 | 0.69 |
| A0A499FJY3 | Putative RNA-binding protein 15 | RBM15 | 250 | S | 1 | 126.7 | 0.87 | 0.88 | 0.90 | NaN | NaN | 0.78 | 1.04 | 1.05 | 1.05 |
| A0A499FJY3 | Putative RNA-binding protein 15 | RBM15 | 213 | S | 0.985 | 82.41 | 0.80 | 0.77 | NaN | NaN | NaN | NaN | NaN | NaN | NaN |
| A0A499FJY3 | Putative RNA-binding protein 15 | RBM15 | 215 | S | 0.986 | 82.41 | 0.80 | 0.77 | NaN | NaN | NaN | NaN | NaN | NaN | NaN |
| A0A499FJY3 | Putative RNA-binding protein 15 | RBM15 | 697 | S | 0.998 | 56.29 | 0.67 | 0.62 | NaN | 0.59 | 0.65 | 0.96 | 0.66 | NaN | 0.87 |
| A0A087WWW9 | B-cell lymphoma/leukemia 10 | BCL10 | 127 | S | 1 | 151.2 | NaN | NaN | 1.60 | 1.60 | NaN | NaN | 1.13 | 0.74 | 1.64 |
| A0A087WWY2 | | FOLR2 | 139 | S | 0.999 | 48.11 | 0.80 | 0.67 | NaN | NaN | 0.73 | NaN | 0.81 | 0.98 | NaN |

| | | | | | | | | | | | | | | | |
|---|---|---|---|---|---|---|---|---|---|---|---|---|---|---|---|
| A0A087WXE2 | RNA pseudouridylate synthase domain-containing | RPUSD2 | 35 | S | 1 | 60.92 | 0.89 | 0.74 | 0.82 | 1.06 | 1.05 | 1.31 | 0.93 | 1.09 | 0.91 |
| A0A087WXN9 | MTSS1-like protein | MTSS1L | 611 | S | 1 | 90.88 | 1.00 | 1.02 | 1.06 | 0.92 | 0.96 | 0.87 | 1.03 | 1.02 | 1.44 |
| A0A499FIX8 | Extended synaptotagmin-2 | ESYT2 | 615 | S | 0.997 | 160.6 | 0.45 | 0.56 | NaN | 0.84 | 1.00 | 0.84 | 0.50 | NaN | 0.32 |
| A0A499FIX8 | Extended synaptotagmin-2 | ESYT2 | 617 | S | 1 | 160.6 | 0.45 | 0.56 | NaN | 0.84 | 1.00 | 0.81 | 0.50 | NaN | 0.32 |
| A0A499FIX8 | Extended synaptotagmin-2 | ESYT2 | 667 | S | 1 | 156.7 | 2.35 | 2.76 | 2.59 | 3.31 | 2.87 | 2.53 | 0.98 | 0.86 | 0.95 |
| A0A087WXZ7 | HEAT repeat-containing protein 6 | HEATR6 | 490 | S | 0.917 | 101.9 | NaN | NaN | NaN | NaN | NaN | NaN | 0.98 | 2.48 | 0.74 |
| A0A087WY28 | Optineurin | OPTN | 64 | S | 0.996 | 51.14 | 1.19 | NaN | 1.23 | 1.10 | 1.39 | NaN | 1.18 | 1.28 | 1.26 |
| A0A087WY61 | Nuclear mitotic apparatus protein 1 | NUMA1 | 169 | S | 0.999 | 241.2 | 0.87 | 0.79 | 0.86 | 1.10 | 1.14 | 1.14 | 0.86 | 0.90 | 0.92 |
| A0A087WY61 | Nuclear mitotic apparatus protein 1 | NUMA1 | 1225 | S | 0.78 | 62.66 | 0.71 | 0.69 | NaN | 0.99 | 1.07 | NaN | NaN | NaN | NaN |
| A0A087WY61 | Nuclear mitotic apparatus protein 1 | NUMA1 | 271 | S | 1 | 69.34 | 2.49 | NaN | NaN | NaN | NaN | NaN | 1.90 | 1.47 | NaN |
| A0A087WY61 | Nuclear mitotic apparatus protein 1 | NUMA1 | 1741 | S | 1 | 239.8 | 1.08 | 1.03 | 1.12 | 1.09 | 1.11 | 1.21 | 1.06 | 1.03 | 1.04 |
| A0A087WYN9 | ATP-dependent RNA helicase DHX29 | DHX29 | 200 | S | 1 | 114.4 | 0.95 | 0.88 | 0.90 | 0.83 | 0.74 | 0.81 | 0.94 | 1.10 | 1.13 |
| A0A087WYW3 | NK-tumor recognition protein;Putative peptidyl-prolyl cis-trans isomerase | NKTR | 163 | S | 0.958 | 62.45 | 1.41 | NaN | NaN | 1.19 | 1.09 | NaN | 1.90 | 2.17 | 1.95 |
| A0A087WZ06 | Serine/threonine-protein kinase 3;Serine/threonine-protein kinase 3 36kDa subunit;Serine/threonine-protein kinase 3 20kDa | STK3 | 274 | S | 0.717 | 40.99 | 0.66 | 0.52 | NaN | NaN | NaN | NaN | NaN | 1.15 | NaN |
| A0A087WZ13 | | RAVER1 | 617 | S | 1 | 67.45 | 0.68 | NaN | 0.79 | 0.96 | 0.93 | 0.99 | 0.77 | 0.65 | 0.64 |
| A0A087WZ13 | | RAVER1 | 567 | S | 1 | 96.11 | 2.75 | 3.42 | 2.77 | 3.99 | 3.29 | 3.39 | 1.51 | 1.55 | NaN |
| B7Z385 | Adenylyl cyclase-associated protein;Adenylyl cyclase-associated protein 2 | CAP2 | 197 | S | 0.998 | 116.8 | 0.72 | 0.48 | 0.48 | 0.35 | 0.66 | 0.41 | 0.68 | 0.54 | 0.66 |
| B7Z385 | Adenylyl cyclase-associated protein;Adenylyl cyclase-associated protein 2 | CAP2 | 200 | S | 0.854 | 139.8 | 0.96 | NaN | 0.92 | 0.88 | 0.87 | 0.64 | 0.81 | 1.02 | 1.09 |
| M0QZS0 | Rho guanine nucleotide exchange factor 18 | ARHGEF18 | 405 | S | 1 | 53.6 | NaN | 1.41 | 1.88 | 1.89 | 2.31 | 1.63 | 1.13 | 2.86 | 1.03 |
| M0QZS0 | Rho guanine nucleotide exchange factor 18 | ARHGEF18 | 742 | S | 1 | 150.1 | 1.92 | 1.75 | 1.71 | 1.03 | 1.06 | 1.12 | 1.86 | 2.04 | 1.80 |
| A0A087WZU8 | Cellular tumor antigen p53 | TP53 | 304 | S | 0.917 | 174.4 | 0.34 | 0.36 | 0.34 | 0.60 | 0.64 | 0.63 | 0.82 | 0.83 | 0.79 |
| F8VQD8 | CCR4-NOT transcription complex subunit 2 | CNOT2 | 80 | S | 0.999 | 143 | 0.87 | 0.67 | NaN | 0.82 | NaN | 0.29 | NaN | 0.73 | 0.68 |
| A0A087X055 | Ras-responsive element-binding protein 1 | RREB1 | 288 | S | 0.861 | 83.11 | 0.82 | 1.19 | NaN | NaN | NaN | NaN | NaN | NaN | NaN |
| A0A087X055 | Ras-responsive element-binding protein 1 | RREB1 | 389 | S | 0.804 | 70.54 | 0.58 | NaN | 0.49 | NaN | NaN | NaN | NaN | 0.58 | 0.64 |
| G3V2W9 | E2F-associated | EAPP | 88 | S | 0.999 | 134.4 | 0.69 | 0.83 | NaN | NaN | NaN | NaN | NaN | NaN | NaN |

| | | | | | | | | | | | | | | |
|---|---|---|---|---|---|---|---|---|---|---|---|---|---|---|
| G3V2W9 | E2F-associated | EAPP | 90 | S | 1 | 134.4 | 0.69 | 0.83 | NaN | NaN | NaN | NaN | NaN | NaN | NaN |
| A0A087X0H9 | RNA-binding protein 26 | RBM26 | 127 | S | 0.974 | 58.75 | 0.85 | 1.20 | NaN | 1.29 | NaN | NaN | NaN | 1.26 | NaN |
| A0A087X0K9 | Tight junction protein ZO-1 | TJP1 | 1525;161[illegible] | S | 0.996 | 173 | 3.08 | 3.16 | 2.60 | 2.08 | 1.82 | 2.12 | 1.28 | 1.12 | 1.20 |
| A0A087X0K9 | Tight junction protein ZO-1 | TJP1 | 113;125 | S | 0.999 | 104.5 | 1.88 | 1.13 | 1.92 | 0.73 | 1.42 | 1.44 | 1.52 | NaN | 1.11 |
| A0A087X0K9 | Tight junction protein ZO-1 | TJP1 | 119;131 | S | 0.974 | 93.66 | 1.88 | NaN | 1.92 | NaN | NaN | 1.50 | NaN | NaN | NaN |
| C9JZJ7 | E3 ubiquitin-protein ligase Midline-1 | MID1 | 96 | S | 0.992 | 135.9 | 1.03 | 0.86 | 0.96 | 0.98 | 1.06 | 0.88 | 1.01 | 1.23 | NaN |
| A0A087X1M6 | Fibronectin type-III domain-containing protein 3A | FNDC3A | 203 | S | 0.961 | 84.31 | 0.57 | NaN | 0.57 | 0.68 | NaN | 0.62 | NaN | 0.59 | 0.52 |
| A0A087X1N7 | Nebulin | NEB | 308 | S | 0.855 | 60.44 | 1.07 | 1.21 | NaN | NaN | 1.44 | NaN | NaN | NaN | NaN |
| E9PL71 | Elongation factor 1-delta | EEF1D | 109 | S | 1 | 238.1 | 0.87 | 0.88 | 0.80 | 1.02 | 1.10 | 1.04 | 0.77 | 0.75 | 0.75 |
| E9PL71 | Elongation factor 1-delta | EEF1D | 138 | S | 1 | 407.8 | 1.02 | 1.05 | 1.05 | 0.97 | 1.04 | 1.04 | 0.94 | 0.91 | 1.03 |
| A0A087X2B6 | Cell cycle and apoptosis regulator protein 2 | CCAR2 | 138 | S | 0.994 | 100.3 | NaN | 1.96 | NaN | NaN | NaN | 1.75 | 1.57 | 2.08 | NaN |
| A0A087X2B6 | Cell cycle and apoptosis regulator protein 2 | CCAR2 | 141 | S | 0.962 | 100.3 | NaN | 1.96 | NaN | NaN | NaN | 1.75 | 3.68 | 2.15 | NaN |
| G3V4V5 | E3 ubiquitin-protein ligase HECTD1 | HECTD1 | 632 | S | 0.828 | 164.3 | 0.88 | 0.90 | 0.87 | 0.95 | NaN | 0.89 | 0.90 | NaN | 0.96 |
| A0A088AWL3 | Nuclear receptor corepressor | NCOR1 | 115 | S | 1 | 145 | NaN | 0.73 | 0.70 | 0.87 | 0.95 | 0.83 | 0.99 | 0.89 | 0.91 |
| A0A088AWL3 | Nuclear receptor corepressor | NCOR1 | 2088 | S | 0.995 | 115.7 | 1.04 | 0.82 | 0.92 | 1.03 | 0.91 | 0.80 | 0.88 | 0.91 | 0.87 |
| A0A088AWL3 | Nuclear receptor corepressor | NCOR1 | 2024 | S | 1 | 83.05 | 0.76 | 0.69 | 0.79 | NaN | 0.83 | 0.83 | 1.03 | 0.87 | 1.16 |
| A0A088AWL3 | Nuclear receptor corepressor | NCOR1 | 1881 | S | 1 | 94.19 | 0.87 | NaN | 0.92 | 0.64 | NaN | 0.74 | 1.23 | NaN | NaN |
| A0A088AWL3 | Nuclear receptor corepressor | NCOR1 | 1884 | S | 0.702 | 94.19 | 0.87 | NaN | 0.92 | 0.64 | NaN | 0.74 | NaN | NaN | NaN |
| A0A494C0S2 | DNA repair protein complementing XP-G cells | ERCC5-201 | 384 | S | 0.759 | 129.1 | 0.81 | NaN | NaN | 0.77 | 0.65 | 0.86 | NaN | 1.02 | 1.02 |
| A0A096LP07 | COP9 signalosome complex subunit 1 | GPS1 | 469 | S | 1 | 187.6 | 1.01 | 0.92 | 0.90 | 0.98 | 1.22 | 1.11 | 1.01 | 1.04 | 0.87 |
| A0A096LP07 | | GPS1 | 212 | S | 0.742 | 47.13 | NaN | NaN | 1.60 | 1.42 | 1.41 | 1.36 | 1.54 | 1.52 | NaN |
| A0A096LP07 | | GPS1 | 219 | S | 0.719 | 47.13 | NaN | NaN | 1.60 | 1.42 | 1.41 | 1.36 | 1.54 | 1.52 | NaN |
| A0A096LP25 | Uncharacterized protein FLJ45252 | AAK1 | 249 | S | 1 | 105 | 0.58 | 0.48 | 0.57 | 0.86 | 0.88 | 0.82 | 0.97 | 1.00 | 0.81 |
| F8W7U3 | WASH complex subunit FAM21C;WASH complex subunit FAM21A | FAM21C | 498;410 | S | 1 | 131 | 0.85 | 1.15 | 0.67 | 1.53 | 1.22 | 1.74 | 0.74 | 0.78 | 0.58 |
| F8W7U3 | WASH complex subunit FAM21C;WASH complex subunit FAM21A | FAM21C | 333;245 | S | 1 | 117.8 | 1.17 | 1.09 | NaN | 0.87 | NaN | NaN | 1.12 | 0.97 | 1.10 |
| F8W7U3 | WASH complex subunit FAM21C;WASH complex subunit FAM21A | FAM21C | 728;640 | S | 1 | 115.5 | NaN | NaN | 1.74 | 1.65 | NaN | NaN | 0.83 | 0.89 | 0.93 |
| A0A0A0MR88 | WASH complex subunit FAM21C | FAM21C | 539 | S | 1 | 158.4 | 1.17 | 1.26 | 1.15 | 1.24 | NaN | 1.18 | 1.12 | 1.07 | 1.05 |
| F5H0Q6 | CLK4-associating serine/arginine rich protein | CLASRP | 285 | S | 0.996 | 49.08 | 0.87 | NaN | NaN | 0.92 | 0.88 | NaN | NaN | 0.91 | 0.96 |
| Q5JSD2 | Voltage-dependent anion-selective channel protein 2 | VDAC2 | 115 | S | 0.973 | 104.9 | 0.85 | 0.72 | 0.86 | 1.02 | 1.15 | NaN | 1.04 | 0.88 | NaN |
| C9JW01 | Pumilio homolog 2 | PUM2 | 136 | S | 1 | 111.1 | 1.16 | NaN | 1.15 | 1.14 | 1.07 | 0.99 | NaN | NaN | 0.92 |

| | | | | | | | | | | | | | | | |
|---|---|---|---|---|---|---|---|---|---|---|---|---|---|---|---|
| C9JW01 | Pumilio homolog 2 | PUM2 | 182 | S | 1 | 130.2 | 1.34 | 1.25 | 1.27 | 1.14 | 1.12 | 1.06 | 1.03 | 1.03 | 1.16 |
| A0A0A0MSK4 | G-protein-signaling modulator | GPSM1 | 492 | S | 0.5 | 111.8 | 0.80 | 0.96 | NaN | 1.21 | 0.96 | 1.16 | NaN | 1.44 | 1.31 |
| A0A0A0MSK4 | G-protein-signaling modulator | GPSM1 | 493 | S | 0.545 | 111.8 | 0.80 | 0.96 | NaN | 1.21 | 0.96 | 1.16 | 1.00 | 1.44 | 1.31 |
| H0YBF7 | Arf-GAP with SH3 domain, ANK repeat and PH domain-containing protein 1 | ASAP1 | 949 | S | 1 | 115.1 | 1.23 | 1.19 | NaN | 1.03 | 1.25 | NaN | 1.07 | 0.86 | NaN |
| H0YBF7 | Arf-GAP with SH3 domain, ANK repeat and PH domain-containing protein 1 | ASAP1 | 538 | S | 1 | 221.3 | 0.74 | 0.83 | 0.88 | 0.79 | 0.76 | 0.70 | 0.95 | 1.04 | 0.95 |
| H0YBF7 | Arf-GAP with SH3 domain, ANK repeat and PH domain-containing protein 1 | ASAP1 | 547 | S | 1 | 144.4 | 0.78 | 0.95 | 0.93 | 1.59 | 1.57 | 1.28 | 0.75 | NaN | 0.61 |
| H0YBF7 | Arf-GAP with SH3 domain, ANK repeat and PH domain-containing protein 1 | ASAP1 | 829 | S | 1 | 95.09 | 1.61 | 1.41 | 1.35 | 1.32 | 1.44 | 1.39 | 1.31 | NaN | 1.19 |
| H0YBF7 | Arf-GAP with SH3 domain, ANK repeat and PH domain-containing protein 1 | ASAP1 | 660 | S | 0.998 | 107.2 | 3.41 | NaN | 2.68 | 3.24 | 3.05 | NaN | 0.83 | 1.28 | 0.72 |
| H0YBF7 | Arf-GAP with SH3 domain, ANK repeat and PH domain-containing protein 1 | ASAP1 | 664 | S | 1 | 78.67 | 3.41 | 1.70 | 2.30 | 1.85 | 3.05 | NaN | 1.55 | 1.68 | NaN |
| A0A0A0MRI1 | GPALPP motifs-containing protein 1 | GPALPP1 | 138 | S | 1 | 110.3 | NaN | NaN | NaN | NaN | 0.83 | 0.58 | 0.50 | 0.54 | NaN |
| A0A0A0MRI1 | GPALPP motifs-containing protein 1 | GPALPP1 | 139 | S | 1 | 110.3 | NaN | NaN | NaN | NaN | 0.83 | 0.58 | 0.50 | 0.54 | NaN |
| A0A0A0MRI1 | GPALPP motifs-containing protein 1 | GPALPP1 | 103 | S | 1 | 172.8 | 0.92 | 0.82 | NaN | 0.86 | 0.86 | NaN | 0.99 | 0.99 | 1.00 |
| A0A0A0MRJ3 | Neuron navigator 1 | NAV1 | 773 | S | 1 | 100.9 | NaN | 1.45 | 1.88 | 1.09 | 1.18 | 1.15 | NaN | 2.50 | 2.15 |
| J3QSX6 | Actin-binding LIM protein 1 | ABLIM1 | 329 | S | 1 | 119.6 | 1.18 | 1.01 | NaN | 1.71 | 1.49 | 1.87 | 0.44 | NaN | 0.43 |
| J3QSX6 | Actin-binding LIM protein 1 | ABLIM1 | 89 | S | 1 | 211.7 | 1.85 | 1.74 | 1.75 | 1.51 | 1.53 | 1.40 | 1.83 | 1.60 | 1.76 |
| A0A0A0MRM9 | Nucleolar and coiled-body phosphoprotein 1 | NOLC1 | 572 | S | 1 | 156.1 | 1.01 | 1.02 | 0.87 | 1.01 | 0.86 | 0.91 | 0.95 | 0.91 | 0.98 |
| A0A0A0MRM9 | Nucleolar and coiled-body phosphoprotein 1 | NOLC1 | 547 | S | 1 | 95.85 | 0.66 | 0.65 | 0.59 | 0.81 | NaN | 0.82 | 0.73 | 0.72 | 0.77 |
| A0A0A0MRN0 | Pre-mRNA-splicing factor 38B | PRPF38B | 416 | S | 1 | 76.05 | 1.57 | 1.66 | 1.25 | 1.48 | 1.66 | 1.39 | 1.32 | 1.18 | 1.21 |
| A0A0A0MRN0 | Pre-mRNA-splicing factor 38B | PRPF38B | 418 | S | 1 | 110.1 | 1.15 | 1.00 | 0.95 | 0.91 | 0.77 | 1.18 | 1.07 | 0.82 | 1.40 |
| A0A0A0MRN5 | Opioid growth factor receptor | OGFR | 485 | S | 0.988 | 107.4 | 0.95 | NaN | 0.88 | 0.96 | 1.03 | 1.42 | 0.78 | 0.66 | NaN |
| A0A0A0MRN5 | Opioid growth factor receptor | OGFR | 465 | S | 0.794 | 142.6 | NaN | NaN | 1.20 | NaN | NaN | NaN | 1.08 | NaN | 1.30 |
| A0A0A0MRN5 | Opioid growth factor receptor | OGFR | 263 | S | 1 | 76.11 | NaN | NaN | NaN | 1.01 | NaN | 1.16 | 1.05 | 1.17 | 0.96 |
| A0A0A0MRN5 | Opioid growth factor receptor | OGFR | 326 | S | 1 | 185 | 1.01 | 1.01 | 1.04 | 1.04 | 1.02 | 1.01 | 1.05 | 1.08 | 1.10 |
| A0A0A0MRP0 | MAP7 domain-containing protein 3 | MAP7D3 | 183 | S | 0.604 | 79.31 | NaN | NaN | NaN | 0.98 | 1.07 | NaN | NaN | NaN | NaN |
| A0A0A0MRP0 | MAP7 domain-containing protein 3 | MAP7D3 | 185 | S | 0.806 | 84.57 | 0.64 | 0.88 | 1.04 | NaN | NaN | 1.02 | 0.54 | NaN | NaN |
| A0A0A0MRP6 | Probable global transcription activator SNF2L1 | SMARCA 1 | 119 | S | 0.96 | 51.77 | 0.49 | 0.51 | 0.63 | NaN | 0.97 | 0.87 | NaN | 0.54 | NaN |

| | | | | | | | | | | | | | | | |
|---|---|---|---|---|---|---|---|---|---|---|---|---|---|---|---|
| A0A0A0MRT2 | WW domain-containing adapter protein with coiled-coil | WAC | 8 | S | 1 | 78.1 | 0.71 | 0.73 | 1.00 | 1.15 | 0.99 | 1.31 | NaN | NaN | 1.62 |
| A0A0A0MRT2 | WW domain-containing adapter protein with coiled-coil | WAC | 359 | S | 1 | 155.6 | 1.21 | 1.01 | 0.86 | 1.14 | 0.95 | 1.02 | 0.72 | 1.03 | 1.00 |
| B6VEX4 | Abl interactor 1 | ABI1 | 225 | S | 1 | 81.38 | 1.45 | 1.44 | 1.29 | 1.26 | 1.24 | 1.12 | NaN | 1.45 | NaN |
| B6VEX4 | Abl interactor 1 | ABI1 | 183 | S | 1 | 132.7 | 0.73 | 0.79 | 0.79 | 1.01 | 1.00 | 0.93 | 0.77 | 0.78 | 0.79 |
| A0A0A0MRW1 | Protein FAM219A | FAM219A | 86 | S | 1 | 189.9 | 1.06 | 1.46 | NaN | 1.84 | NaN | 1.26 | NaN | 0.82 | 1.11 |
| A0A0A0MSA7 | Eukaryotic translation initiation factor 4 gamma 3 | EIF4G3 | 303 | S | 0.5 | 58.23 | 0.87 | 1.14 | 0.95 | NaN | NaN | 0.96 | NaN | NaN | NaN |
| A0A0A0MSA7 | Eukaryotic translation initiation factor 4 gamma 3 | EIF4G3 | 304 | S | 0.5 | 58.23 | 0.87 | 1.14 | 0.95 | NaN | NaN | 0.96 | NaN | NaN | NaN |
| A0A0A0MSA7 | Eukaryotic translation initiation factor 4 gamma 3 | EIF4G3 | 494 | S | 1 | 105 | 1.13 | 1.20 | 1.24 | 1.22 | 1.21 | 1.25 | 1.29 | 1.32 | 1.27 |
| B3KPJ4 | Polyhomeotic-like protein 2 | PHC2 | 357 | S | 0.768 | 61.35 | NaN | NaN | NaN | 1.21 | 0.92 | NaN | NaN | NaN | NaN |
| A0A0A0MSK5 | Torsin-1A-interacting protein 1 | TOR1AIP 1 | 33 | S | 1 | 148.9 | 1.42 | 1.24 | 1.81 | 3.30 | 2.60 | 2.74 | 2.35 | 3.16 | 1.75 |
| A0A0A0MSK5 | Torsin-1A-interacting protein 1 | TOR1AIP 1 | 35 | S | 1 | 187.8 | 1.30 | 1.24 | 1.81 | 3.30 | 2.60 | 2.74 | 2.35 | 3.16 | 1.47 |
| A0A0A0MSK5 | Torsin-1A-interacting protein 1 | TOR1AIP 1 | 36 | S | 1 | 187.8 | 1.30 | 1.24 | 1.81 | 3.30 | 2.60 | 2.74 | 2.35 | 3.16 | 1.47 |
| A0A0A0MSK5 | Torsin-1A-interacting protein 1 | TOR1AIP 1 | 22 | S | 0.908 | 65.2 | NaN | 4.14 | 3.93 | 3.28 | NaN | NaN | NaN | NaN | NaN |
| A0A0A0MSL3 | Ubiquitin-conjugating enzyme E2 variant 1 | UBE2V1 | 102 | S | 1 | 99.83 | 0.77 | 0.83 | 0.63 | 0.96 | 0.93 | 0.70 | 0.84 | 0.88 | 1.17 |
| Q5TBM3 | Heat shock protein 105 kDa | HSPH1 | 73 | S | 1 | 95.62 | NaN | 1.42 | 1.33 | 1.11 | 1.12 | 1.12 | 0.95 | 0.89 | 0.97 |
| A0A0G2JI50 | Negative elongation factor E | NELFE | 174 | S | 0.747 | 117.2 | NaN | 0.83 | 0.73 | NaN | NaN | 0.91 | 0.88 | NaN | 0.52 |
| A0A0G2JI50 | Negative elongation factor E | NELFE | 176 | S | 0.861 | 122.2 | 0.97 | 0.66 | NaN | 0.84 | NaN | 0.54 | 0.80 | 0.81 | 0.92 |
| A0A0G2JI50 | Negative elongation factor E | NELFE | 115 | S | 1 | 154.2 | 1.65 | 1.66 | NaN | NaN | NaN | NaN | NaN | NaN | NaN |
| A0A0A0MSU2 | ELM2 and SANT domain-containing protein 1 | ELMSAN1 | 461 | S | 1 | 88.22 | 0.56 | 0.40 | NaN | 0.64 | 0.86 | NaN | 0.68 | NaN | NaN |
| Q5TCC6 | Isoleucine--tRNA ligase, cytoplasmic | IARS | 100 | S | 0.964 | 77.22 | NaN | 1.11 | 1.25 | NaN | NaN | 1.73 | NaN | 0.94 | NaN |
| A0A0A0MSY4 | | DOCK9 | 37 | S | 1 | 131.4 | 1.72 | 1.84 | NaN | NaN | NaN | NaN | NaN | NaN | NaN |
| A0A0A0MT33 | Protein SCAF8 | SCAF8 | 695 | S | 0.999 | 203.2 | 0.93 | 0.91 | 0.99 | 1.00 | 1.33 | 1.08 | 1.01 | 1.19 | 1.05 |
| A0A2R8YF38 | Transcription activator BRG1 | SMARCA 4 | 18 | S | 1 | 62.65 | NaN | NaN | NaN | 1.00 | 1.26 | NaN | NaN | 0.87 | NaN |
| A0A0A0MT60 | Peptidyl-prolyl cis-trans isomerase;FK506-binding protein 15 | FKBP15 | 1139 | S | 1 | 91.59 | 0.95 | 1.02 | 0.80 | 0.74 | 0.73 | NaN | 0.95 | 1.00 | 0.89 |
| A0A0A0MTR7 | E3 ubiquitin-protein ligase RNF213 | RNF213 | 1258 | S | 1 | 158.5 | 0.92 | 0.81 | 0.92 | 0.97 | NaN | 0.58 | 1.08 | 1.03 | 1.07 |
| H3BMG9 | Protein FAM65A | FAM65A | 310 | S | 1 | 246.3 | 1.09 | 0.99 | 1.04 | 0.77 | 0.83 | 0.77 | 1.53 | 1.47 | 1.52 |
| A0A0A0MTP7 | Protein SCAF11 | SCAF11 | 278 | S | 0.605 | 148.3 | NaN | 0.74 | NaN | NaN | NaN | NaN | 0.87 | 0.94 | NaN |
| A0A0A0MTP7 | Protein SCAF11 | SCAF11 | 548 | S | 1 | 175.8 | 0.86 | 0.84 | NaN | 0.94 | 1.03 | 0.82 | 0.99 | 0.83 | 0.99 |
| A0A0A0MTP7 | Protein SCAF11 | SCAF11 | 841 | S | 0.5 | 65.59 | 0.73 | NaN | NaN | 0.86 | NaN | 0.67 | 0.69 | NaN | NaN |
| A0A0A0MTP7 | Protein SCAF11 | SCAF11 | 842 | S | 0.5 | 65.59 | 0.73 | NaN | NaN | 0.86 | NaN | 0.67 | 0.69 | NaN | NaN |

| | | | | | | | | | | | | | | | |
|---|---|---|---|---|---|---|---|---|---|---|---|---|---|---|---|
| A0A0A0MTQ8 | Coiled-coil domain-containing protein 175 | CCDC175 | 583 | S | 1 | 48.95 | 0.42 | 0.44 | NaN | 0.25 | NaN | NaN | 0.35 | 0.33 | NaN |
| A0A0A6YYC0 | Ribosomal protein S6 kinase;Ribosomal protein S6 kinase alpha-4 | RPS6KA4 | 331 | S | 0.999 | 108.7 | 0.55 | 0.43 | NaN | NaN | NaN | NaN | NaN | 0.99 | 0.79 |
| A0A0A6YYC7 | E3 ubiquitin-protein ligase ZFP91 | ZFP91-CNTF | 83 | S | 0.823 | 93.45 | 0.76 | NaN | NaN | 0.66 | 0.69 | NaN | NaN | 0.89 | NaN |
| A8MV53 | Suppressor of SWI4 1 | PPAN | 306 | S | 1 | 148.9 | 1.21 | 1.30 | 1.34 | 1.29 | 1.28 | 1.31 | 1.08 | 1.05 | 1.06 |
| A0A0A6YYJ8 | Putative RNA-binding protein Luc7-like 2 | LUC7L2 | 449 | S | 1 | 106.9 | 0.95 | 0.99 | 1.01 | 0.89 | 0.90 | 0.91 | 0.93 | 0.95 | 0.99 |
| A0A0A6YYJ8 | Putative RNA-binding protein Luc7-like 2 | LUC7L2 | 450 | S | 1 | 106.9 | 0.95 | 0.99 | 1.01 | 0.89 | 0.90 | 0.91 | 0.93 | 0.95 | 0.99 |
| A0A0B4J1S8 | Phosphatidylinositol 4-kinase beta | PI4KB | 440 | S | 1 | 118.5 | 1.77 | 1.91 | NaN | 1.16 | 1.43 | NaN | 1.82 | 1.84 | 2.33 |
| H0YEG1 | Mediator of RNA polymerase II transcription subunit 24 | MED24 | 49 | S | 0.83 | 156.1 | 1.46 | 0.95 | 0.91 | 1.29 | NaN | NaN | NaN | 0.81 | 0.68 |
| A0A0C4DFS6 | Protein sprouty homolog 4 | SPRY4 | 148 | S | 1 | 82.48 | 2.62 | 2.24 | 2.75 | 2.47 | 2.27 | 2.41 | 1.49 | 1.25 | 1.98 |
| E7EQD7 | Disks large homolog 1 | DLG1 | 102 | S | 0.666 | 137 | 1.72 | 1.95 | 2.06 | 1.79 | 1.86 | NaN | NaN | NaN | NaN |
| H0Y3X6 | Negative elongation factor A | NELFA | 367 | S | 0.962 | 49.09 | 0.97 | 0.92 | NaN | NaN | 1.46 | NaN | NaN | 1.27 | 1.28 |
| A0A0C4DG07 | Nibrin | NBN | 350 | S | 0.968 | 49.09 | 0.74 | 0.78 | NaN | NaN | 1.10 | 1.01 | 0.69 | 0.59 | NaN |
| H7C3Q7 | Abl interactor 2 | ABI2 | 93 | S | 0.87 | 69.35 | 1.13 | 1.43 | NaN | 1.59 | NaN | NaN | NaN | NaN | NaN |
| H7C3Q7 | Abl interactor 2 | ABI2 | 49 | S | 0.965 | 82.19 | 0.70 | NaN | 0.68 | 0.84 | 0.83 | 0.81 | 0.69 | 0.67 | 0.63 |
| H7BZV4 | ADP-ribosylation factor-like protein 6-interacting protein 4 | ARL6IP4 | 130 | S | 1 | 276.8 | 1.66 | 1.54 | 1.74 | 1.45 | 1.52 | 1.44 | 1.70 | 1.68 | 1.69 |
| A0A0C4DG89 | Probable ATP-dependent RNA helicase DDX46 | DDX46 | 804 | S | 1 | 225.1 | 0.59 | 0.61 | 0.61 | 0.63 | 0.69 | 0.72 | 0.48 | 0.48 | 0.53 |
| H7C477 | THO complex subunit 2 | THOC2 | 153 | S | 0.967 | 67.17 | 0.73 | 0.63 | NaN | NaN | NaN | NaN | NaN | NaN | NaN |
| A0A0C4DGA7 | Actin-binding LIM protein 3 | ABLIM3 | 470 | S | 1 | 40.32 | NaN | 1.99 | NaN | NaN | 1.37 | NaN | 2.24 | 2.38 | 2.75 |
| A0A0C4DGA7 | Actin-binding LIM protein 3 | ABLIM3 | 471 | S | 1 | 40.32 | NaN | 1.99 | NaN | NaN | 1.37 | NaN | 2.24 | 2.38 | 2.75 |
| A0A0C4DGB5 | Calpastatin | CAST | 289 | S | 0.987 | 309 | 1.15 | 0.90 | 0.96 | 0.76 | 1.76 | 1.32 | 0.81 | 0.46 | 1.03 |
| A0A0C4DGB5 | Calpastatin | CAST | 412 | S | 0.62 | 99.11 | 1.11 | 1.27 | NaN | 1.21 | NaN | NaN | NaN | 1.32 | NaN |
| A0A0C4DGG1 | Protein kinase C and casein kinase substrate in neurons protein 3 | PACSIN3 | 354 | S | 0.998 | 150.3 | 0.37 | 0.59 | 0.38 | NaN | NaN | NaN | 1.42 | NaN | 1.58 |
| A0A0C4DGG1 | Protein kinase C and casein kinase substrate in neurons protein 3 | PACSIN3 | 319 | S | 0.995 | 68.82 | 1.07 | 1.21 | NaN | 0.94 | NaN | NaN | 1.10 | 1.40 | NaN |
| A0A2R8YE38 | Chromodomain-helicase-DNA-binding protein 4 | CHD4 | 286 | S | 0.997 | 152.4 | 0.45 | 0.50 | 0.53 | 0.63 | 0.62 | 0.63 | 0.44 | 0.44 | 0.44 |
| A0A0C4DGH6 | UHRF1-binding protein 1-like | UHRF1BP1L | 639 | S | 0.947 | 97.9 | NaN | NaN | NaN | 0.54 | 0.59 | NaN | 0.77 | NaN | NaN |
| H0YHL2 | Bromodomain adjacent to zinc finger domain protein 2A | BAZ2A | 66 | S | 0.894 | 47.06 | NaN | NaN | NaN | NaN | NaN | 0.94 | NaN | 0.51 | 0.87 |
| J3QRU8 | ARF GTPase-activating protein GIT1 | GIT1 | 592 | S | 0.676 | 64.55 | NaN | NaN | NaN | NaN | NaN | NaN | 0.87 | 0.97 | NaN |
| J3QRU8 | ARF GTPase-activating protein GIT1 | GIT1 | 362 | S | 0.953 | 101.8 | 0.92 | NaN | NaN | NaN | 1.20 | 1.28 | 0.73 | 0.66 | 0.79 |

| | | | | | | | | | | | | | | | |
|---|---|---|---|---|---|---|---|---|---|---|---|---|---|---|---|
| J3QRU8 | ARF GTPase-activating protein GIT1 | GIT1 | 388 | S | 1 | 317.9 | NaN | NaN | NaN | NaN | NaN | NaN | 0.70 | NaN | 0.63 |
| H7BXT7 | BET1-like protein | BET1L | 9 | S | 1 | 130.6 | 2.21 | 2.46 | 2.82 | NaN | NaN | NaN | 2.38 | NaN | NaN |
| A0A0C4DH22 | Band 4.1-like protein 1 | EPB41L1 | 783 | S | 1 | 108 | 1.90 | 2.12 | NaN | 1.29 | 1.25 | 1.40 | 1.54 | 1.66 | 1.56 |
| Q5QPR4 | Cyclin-dependent kinase 11A;Cyclin-dependent kinase 11B | CDK11A | 234 | S | 0.982 | 190.8 | 0.61 | 0.66 | NaN | 0.84 | 0.81 | 0.80 | 0.86 | NaN | NaN |
| Q5QPR4 | Cyclin-dependent kinase 11A;Cyclin-dependent kinase 11B | CDK11A | 540 | S | 1 | 81.26 | 0.87 | NaN | 0.90 | 0.81 | 0.77 | 0.83 | 0.96 | 1.02 | 1.07 |
| Q5QPR4 | Cyclin-dependent kinase 11A;Cyclin-dependent kinase 11B | CDK11A | 13 | S | 1 | 88.77 | 0.97 | 0.97 | NaN | 0.82 | 1.04 | 0.89 | NaN | 0.94 | 0.86 |
| H7C3Z6 | Mitogen-activated protein kinase kinase kinase kinase 4 | MAP4K4 | 243 | S | 0.946 | 50.9 | 1.36 | 1.42 | NaN | NaN | NaN | 1.02 | NaN | NaN | NaN |
| E7EXA6 | Chromosome transmission fidelity protein 18 homolog | CHTF18 | 871 | S | 1 | 108.4 | 0.53 | 0.43 | 0.64 | 0.62 | 0.65 | 0.53 | 0.83 | NaN | 0.76 |
| A0A0D9SFE4 | | DNM1 | 847 | S | 0.99 | 63.8 | 0.85 | 0.70 | 0.90 | 0.84 | NaN | 0.83 | 0.92 | 1.11 | 0.85 |
| A0A0G2JHC2 | Phostensin | PPP1R18 | 125 | S | 1 | 47.56 | 1.01 | NaN | NaN | 1.20 | 0.91 | 1.15 | NaN | NaN | 0.84 |
| A0A0G2JHC2 | Phostensin | PPP1R18 | 224 | S | 1 | 103.9 | 1.02 | NaN | 1.26 | 1.51 | NaN | NaN | NaN | NaN | 1.27 |
| A0A0G2JHN8 | Bromodomain-containing protein 2 | BRD2 | 160 | S | 0.995 | 123.8 | NaN | 0.81 | NaN | 0.97 | 0.74 | 0.90 | NaN | NaN | NaN |
| A0A0G2JMS7 | Protein scribble homolog | SCRIB | 1405 | S | 1 | 120.3 | 1.80 | 2.40 | NaN | NaN | 2.31 | 2.36 | NaN | 0.97 | 1.46 |
| A0A0G2JMS7 | Protein scribble homolog | SCRIB | 1485 | S | 0.971 | 101.5 | 1.19 | NaN | NaN | 1.34 | 1.05 | NaN | 1.08 | NaN | 0.60 |
| A0A0G2JMS7 | Protein scribble homolog | SCRIB | 1394 | S | 1 | 62.34 | 2.88 | NaN | NaN | 1.90 | 1.94 | NaN | 1.07 | 1.20 | NaN |
| A0A0G2JMS7 | Protein scribble homolog | SCRIB | 1225 | S | 0.997 | 88.54 | 0.60 | 0.57 | 0.62 | 0.86 | NaN | 0.67 | 0.90 | 0.76 | 0.74 |
| A0A0G2JMS7 | Protein scribble homolog | SCRIB | 1228 | S | 1 | 88.54 | 0.60 | 0.57 | 0.62 | 0.86 | NaN | 0.67 | 0.90 | 0.76 | 0.74 |
| A0A0G2JMS7 | Protein scribble homolog | SCRIB | 754 | S | 0.998 | 117.5 | 0.93 | NaN | NaN | 0.94 | 0.99 | NaN | 0.99 | NaN | 1.12 |
| A0A0G2JMS7 | Protein scribble homolog | SCRIB | 1367 | S | 1 | 119.5 | NaN | 1.39 | 1.43 | 1.31 | 1.16 | 1.21 | 1.19 | 1.21 | 1.14 |
| A0A0G2JNJ7 | CCR4-NOT transcription complex subunit 3 | CNOT3 | 221 | S | 0.757 | 71.73 | 0.85 | NaN | NaN | NaN | 1.31 | 1.48 | 0.76 | 0.79 | NaN |
| F6RP06 | BCL2/adenovirus E1B 19 kDa protein-interacting protein 3 | BNIP3 | 105 | S | 1 | 59.27 | NaN | 1.14 | NaN | 0.85 | NaN | 0.93 | 0.96 | NaN | 1.12 |
| A0A0J9YX88 | PHD and RING finger domain-containing protein 1 | PHRF1 | 1360 | S | 1 | 153 | 0.58 | NaN | 0.90 | 0.77 | 0.73 | 0.74 | 1.02 | 1.39 | 0.91 |
| A0A0J9YX88 | PHD and RING finger domain-containing protein 1 | PHRF1 | 1371 | S | 0.974 | 92.96 | 0.58 | NaN | 0.79 | 0.64 | NaN | 0.74 | NaN | 0.99 | 0.99 |
| A0A0J9YX88 | PHD and RING finger domain-containing protein 1 | PHRF1 | 1202 | S | 1 | 98.3 | 1.65 | 1.68 | 1.68 | NaN | NaN | 1.12 | NaN | NaN | 1.60 |
| A0A0J9YX88 | PHD and RING finger domain-containing protein 1 | PHRF1 | 915 | S | 0.989 | 169.9 | NaN | 1.52 | 1.51 | NaN | NaN | 0.95 | NaN | NaN | 0.91 |
| A0A0J9YX88 | PHD and RING finger domain-containing protein 1 | PHRF1 | 1229 | S | 0.969 | 44.18 | NaN | NaN | 0.53 | NaN | 0.69 | NaN | 0.95 | 1.04 | NaN |
| A0A0J9YX88 | PHD and RING finger domain-containing protein 1 | PHRF1 | 973 | S | 1 | 108 | 1.36 | 1.20 | NaN | 1.86 | 1.30 | 1.18 | 1.31 | 1.15 | 1.19 |
| A0A0J9YWL0 | Absent in melanoma 1 protein | AIM1 | 941 | S | 0.692 | 96.55 | 0.58 | NaN | 0.69 | NaN | NaN | NaN | 0.75 | NaN | 1.01 |

| | | | | | | | | | | | | | | | |
|---|---|---|---|---|---|---|---|---|---|---|---|---|---|---|---|
| E9PH18 | DnaJ homolog subfamily B member 6 | DNAJB6 | 162 | S | 1 | 179.6 | 1.97 | NaN | NaN | 2.11 | 2.04 | NaN | NaN | NaN | NaN |
| A0A0J9YXZ5 | Ras GTPase-activating-like protein IQGAP1 | IQGAP1 | 330 | S | 1 | 120.9 | 1.95 | 2.08 | 2.09 | 3.11 | 2.99 | 2.91 | 1.10 | 1.02 | 1.18 |
| A0A0J9YYJ3 | Transmembrane protein 184B | TMEM184 B | 343 | S | 0.997 | 82.75 | 1.27 | 1.29 | 0.67 | 1.22 | 1.24 | 1.10 | 1.14 | 1.16 | 1.14 |
| A0A0J9YYJ3 | Transmembrane protein 184B | TMEM184 B | 344 | S | 0.999 | 92.05 | 0.56 | 1.29 | NaN | 1.22 | 1.24 | 0.82 | 1.14 | 1.16 | 1.04 |
| D6REM6 | Matrin-3 | MATR3 | 188 | S | 1 | 80.46 | 2.23 | NaN | NaN | 1.27 | 1.35 | NaN | NaN | NaN | NaN |
| D6REM6 | Matrin-3 | MATR3 | 596 | S | 0.949 | 55.71 | 0.59 | 0.65 | 0.75 | 0.74 | NaN | NaN | NaN | 1.03 | 1.00 |
| D6REM6 | Matrin-3 | MATR3 | 598 | S | 1 | 216.4 | 0.83 | 0.99 | 0.78 | 0.80 | 0.79 | 0.81 | 0.79 | 0.79 | 0.82 |
| D6REM6 | Matrin-3 | MATR3 | 604 | S | 0.984 | 127 | 0.46 | 0.45 | 3.55 | 0.55 | 0.51 | 0.52 | 0.82 | 0.86 | 0.91 |
| A0A0U1RQF0 | Fatty acid synthase;[Acyl-carrier-protein] S-acetyltransferase;[Acyl-carrier-protein] S-malonyltransferase;3-oxoacyl-[acyl-carrier-protein] synthase;3-oxoacyl-[acyl-carrier-protein] reductase;3-hydroxyacyl-[acyl-carrier-protein] dehydratase;Enoyl-[acyl-carrier-protein] | FASN | 207 | S | 0.994 | 84.17 | NaN | NaN | 0.63 | 0.95 | 0.74 | 0.87 | 0.45 | 0.44 | 0.55 |
| A0A0U1RRM7 | ATR-interacting protein | ATRIP | 131 | S | 0.997 | 80.05 | NaN | NaN | 0.96 | 0.92 | NaN | 0.99 | 1.16 | 0.94 | 0.83 |
| A0A0U1RQU3 | Calcium-transporting ATPase;Plasma membrane calcium-transporting ATPase | ATP2B1 | 258 | S | 0.986 | 84.19 | 0.43 | 0.35 | 0.37 | 0.47 | 0.52 | 0.51 | 0.49 | 0.44 | 0.58 |
| A0A0U1RRD7 | InaD-like protein | INADL | 28 | S | 0.986 | 102.2 | NaN | NaN | NaN | 0.62 | 0.78 | NaN | NaN | 0.46 | NaN |
| A0A1B0GW38 | E3 ubiquitin-protein ligase | CBL | 481 | S | 1 | 89.77 | 1.11 | 1.05 | 1.19 | 1.18 | 1.34 | 1.10 | 0.88 | 0.75 | NaN |
| A0A140T9I0 | HLA class I histocompatibility antigen, A-80 alpha chain;HLA class I histocompatibility antigen, A-23 alpha chain;HLA class I histocompatibility antigen, A-69 alpha chain;HLA class I histocompatibility antigen, A-24 alpha chain;HLA class I histocompatibility antigen, A-2 alpha chain;HLA class I histocompatibility | HLA-A | 356 | S | 0.997 | 134.1 | 3.07 | 3.16 | NaN | 3.39 | 1.93 | NaN | NaN | NaN | NaN |
| A2AB15 | Putative pre-mRNA-splicing factor ATP-dependent RNA helicase DHX16 | DHX16 | 43 | S | 1 | 219.5 | 0.42 | 0.49 | 1.19 | 1.16 | 0.82 | 0.80 | 1.41 | 0.62 | 1.35 |

| | | | | | | | | | | | | | | | |
|---|---|---|---|---|---|---|---|---|---|---|---|---|---|---|---|
| A2AB15 | Putative pre-mRNA-splicing factor ATP-dependent RNA helicase DHX16 | DHX16 | 46 | S | 1 | 219.5 | 1.23 | 1.15 | 1.19 | 1.16 | 1.19 | 1.14 | 1.41 | 1.37 | 1.35 |
| A2AB15 | Putative pre-mRNA-splicing factor ATP-dependent RNA helicase DHX16 | DHX16 | 100 | S | 1 | 207.6 | 0.71 | 0.80 | 0.80 | 0.91 | 0.75 | 0.65 | 0.83 | 0.89 | NaN |
| A0A1B0GTJ6 | | | 248 | S | 0.955 | 71.15 | 1.10 | 1.08 | 1.23 | 1.08 | NaN | 1.01 | 1.19 | NaN | 1.36 |
| A0A1B0GTL5 | | | 634 | S | 0.998 | 66.92 | 1.34 | 1.29 | NaN | NaN | NaN | NaN | NaN | 1.50 | 1.59 |
| A0A1B0GTL5 | | | 640 | S | 0.997 | 77.81 | 1.05 | 1.16 | 1.25 | 1.05 | 1.13 | 1.11 | 1.27 | 1.27 | 1.25 |
| A0A1B0GTL5 | | | 866 | S | 0.999 | 150.7 | 0.76 | 0.72 | 1.20 | 0.94 | 1.04 | 0.93 | 1.20 | 1.28 | 1.30 |
| A0A1B0GTL5 | | | 871 | S | 0.951 | 69.58 | NaN | NaN | NaN | 0.34 | NaN | 1.52 | NaN | NaN | NaN |
| A0A1B0GTL5 | | | 938 | S | 0.988 | 102.7 | NaN | NaN | NaN | 1.05 | 0.96 | 0.91 | 1.01 | 1.08 | NaN |
| A0A1B0GTL5 | | | 911 | S | 0.802 | 91.65 | NaN | NaN | NaN | 1.27 | NaN | 1.26 | NaN | NaN | NaN |
| A0A1B0GTL5 | | | 913 | S | 0.987 | 166.4 | 1.47 | 1.97 | 1.76 | NaN | 1.26 | 1.22 | NaN | 1.93 | 1.55 |
| A0A1B0GTL5 | Rab11 family-interacting protein 5 | RAB11FIP5 | 482;482 | S | 0.977 | 64.91 | 3.33 | NaN | 2.33 | 1.77 | NaN | NaN | 2.84 | NaN | NaN |
| A0A1B0GTL5 | Rab11 family-interacting protein 5 | RAB11FIP5 | 307;307 | S | 1 | 120 | 1.08 | 1.15 | 1.16 | 1.12 | 1.19 | 1.11 | 0.78 | 0.82 | NaN |
| F5GZ78 | Paxillin | PXN | 300 | S | 0.639 | 136.4 | 1.37 | 1.47 | NaN | 1.19 | 1.23 | NaN | 1.09 | 0.85 | NaN |
| F5GZ78 | Paxillin | PXN | 301 | S | 0.978 | 115.7 | 1.62 | 1.38 | 1.32 | 1.19 | 1.08 | 1.40 | NaN | 1.01 | 1.25 |
| F5GZ78 | Paxillin | PXN | 83 | S | 0.911 | 250.3 | 1.70 | 1.81 | 1.80 | 1.74 | 2.15 | 1.73 | 1.20 | 1.11 | 1.28 |
| F5GZ78 | Paxillin | PXN | 256 | S | 0.971 | 42.21 | NaN | 2.20 | 2.53 | 2.54 | 2.15 | 2.52 | 2.77 | 2.01 | NaN |
| F5GZ78 | Paxillin | PXN | 320 | S | 0.999 | 396.1 | 0.81 | 0.80 | 0.77 | 0.90 | NaN | 1.00 | 0.83 | 0.79 | 0.83 |
| F5GZ78 | Paxillin | PXN | 104 | S | 1 | 378.2 | 4.54 | 5.48 | 4.12 | 3.79 | 3.86 | 3.12 | 1.57 | 1.41 | 1.63 |
| F5GZ78 | Paxillin | PXN | 117 | S | 0.676 | 60.36 | NaN | NaN | 2.14 | NaN | NaN | NaN | NaN | 1.03 | 2.47 |
| A0A3B3ITE1 | Tight junction protein ZO-2 | TJP2 | 979 | S | 0.994 | 91.47 | 1.24 | 1.12 | 1.13 | 0.96 | 1.04 | 1.17 | 1.68 | 1.57 | 1.67 |
| A0A3B3ITE1 | Tight junction protein ZO-2 | TJP2 | 267 | S | 1 | 115.7 | 1.19 | 1.21 | 1.21 | 1.16 | 1.14 | 1.08 | 1.29 | 1.26 | 1.34 |
| A0A3B3ITE1 | Tight junction protein ZO-2 | TJP2 | 987 | S | 1 | 128.9 | 0.85 | 0.84 | 0.79 | 0.83 | 0.85 | 0.82 | 0.91 | 0.93 | 0.94 |
| A0A3B3ITE1 | Tight junction protein ZO-2 | TJP2 | 395 | S | 0.963 | 79.9 | 2.12 | 0.94 | NaN | NaN | NaN | NaN | NaN | NaN | NaN |
| A0A3B3ITE1 | Tight junction protein ZO-2 | TJP2 | 399 | S | 0.973 | 172.3 | 2.12 | 0.94 | NaN | NaN | NaN | NaN | 1.26 | NaN | NaN |
| A0A3B3ITE1 | Tight junction protein ZO-2 | TJP2 | 401 | S | 0.718 | 94.5 | 2.12 | 0.94 | NaN | NaN | NaN | NaN | NaN | NaN | NaN |
| A0A3B3ITE1 | Tight junction protein ZO-2 | TJP2 | 416 | S | 1 | 112.4 | 1.16 | 0.60 | 0.63 | 0.59 | 0.60 | 0.60 | 0.76 | 0.75 | 0.77 |
| A0A3B3ITE1 | Tight junction protein ZO-2 | TJP2 | 1160 | S | 1 | 255.9 | 1.69 | 1.78 | 1.53 | 1.42 | NaN | 1.33 | 1.78 | 1.93 | 1.93 |
| A0A3B3ITE1 | Tight junction protein ZO-2 | TJP2 | 967 | S | 1 | 87 | 1.13 | 1.34 | NaN | NaN | 1.05 | NaN | NaN | 1.18 | NaN |
| A0A3B3ITE1 | Tight junction protein ZO-2 | TJP2 | 131 | S | 1 | 145.8 | 0.92 | 1.08 | 1.02 | 1.08 | 1.26 | 1.13 | 0.93 | 0.84 | 0.89 |
| A0A3B3ITE1 | Tight junction protein ZO-2 | TJP2 | 1068 | S | 0.832 | 90.3 | 1.58 | 1.32 | 1.39 | 1.68 | NaN | 1.36 | 1.66 | 1.18 | 1.56 |
| A0A3B3ITE1 | Tight junction protein ZO-2 | TJP2 | 1069 | S | 0.545 | 75.05 | 0.82 | NaN | NaN | 1.44 | 1.41 | 1.36 | NaN | 1.18 | NaN |
| A0A3B3ITE1 | Tight junction protein ZO-2 | TJP2 | 171 | S | 1 | 102.9 | 0.46 | 0.47 | 1.38 | 1.27 | 1.25 | 1.25 | 1.66 | 1.61 | 1.62 |
| A0A3B3ITE1 | Tight junction protein ZO-2 | TJP2 | 175 | S | 1 | 104.8 | 1.17 | 1.23 | 1.02 | 0.92 | 1.07 | 1.01 | 1.06 | 1.08 | 1.02 |
| A0A1B0GUM1 | Pleckstrin homology domain-containing family A member 7 | PLEKHA7 | 88 | S | 0.983 | 92.73 | 0.51 | 1.04 | 0.69 | NaN | NaN | NaN | NaN | NaN | NaN |
| Q5VTI5 | Pleckstrin homology domain-containing family A member 6 | PLEKHA6 | 797 | S | 0.947 | 155.2 | 0.70 | NaN | NaN | 0.88 | NaN | NaN | 0.72 | 0.79 | NaN |
| A0A1B0GV68 | FERM, RhoGEF and pleckstrin domain-containing | FARP1 | 427 | S | 0.978 | 47.08 | NaN | NaN | 3.04 | NaN | NaN | NaN | 3.90 | 3.44 | NaN |
| E7EUR8 | Protein FAM193A | FAM193A | 147 | S | 0.919 | 82.48 | NaN | 0.95 | NaN | 1.31 | 1.20 | NaN | 0.86 | NaN | NaN |

| | | | | | | | | | | | | | | | |
|---|---|---|---|---|---|---|---|---|---|---|---|---|---|---|---|
| A0A1B0GVY1 | E3 ubiquitin-protein ligase NEDD4-like | NEDD4L | 582 | S | 0.976 | 79.61 | 0.75 | 0.70 | NaN | 0.66 | 0.67 | NaN | NaN | NaN | NaN |
| R4GMM6 | Ubiquitin-conjugating enzyme E2 E3 | UBE2E3 | 8 | S | 1 | 169.1 | 0.87 | 0.86 | NaN | NaN | NaN | NaN | NaN | NaN | NaN |
| A0A1C7CYX9 | | | 27 | S | 0.997 | 92.79 | 1.72 | 1.98 | 1.71 | 1.33 | NaN | 1.34 | 1.69 | 1.80 | 1.87 |
| A0A1W2PNZ9 | Probable JmjC domain-containing histone demethylation protein 2C | JMJD1C | 1825 | S | 0.815 | 75.33 | NaN | 0.45 | NaN | 0.70 | 0.50 | NaN | NaN | NaN | NaN |
| A0A1W2PP04 | RNA-binding motif protein, X-linked 2 | RBMX2 | 187 | S | 0.674 | 209.2 | NaN | NaN | NaN | NaN | 0.82 | NaN | 0.95 | 0.97 | NaN |
| A0A1W2PRI6 | Heterogeneous nuclear ribonucleoprotein U | HNRNPU | 53 | S | 1 | 184.4 | 0.63 | 0.60 | 0.74 | 0.51 | 0.64 | 0.81 | NaN | NaN | NaN |
| E5RIS7 | Transcription elongation factor A protein 1 | TCEA1 | 97 | S | 0.829 | 91.93 | NaN | 0.94 | NaN | 0.83 | NaN | 0.79 | NaN | NaN | 1.00 |
| E5RIS7 | Transcription elongation factor A protein 1 | TCEA1 | 100 | S | 1 | 183.1 | 0.76 | 0.79 | 0.83 | 0.85 | 0.89 | 0.85 | 0.87 | 1.02 | 0.91 |
| B4DP61 | Survival motor neuron protein | SMN2 | 28 | S | 1 | 291.7 | 1.03 | 0.98 | 0.92 | 1.10 | 1.05 | 1.03 | 0.92 | 0.86 | 0.95 |
| B4DP61 | Survival motor neuron protein | SMN2 | 31 | S | 1 | 133.7 | 3.19 | 2.68 | 3.72 | 2.50 | 2.87 | 2.39 | 2.28 | 0.96 | 1.90 |
| A0A286YEX4 | Nuclear factor 1 C-type | NFIC | 345 | S | 0.944 | 219.3 | NaN | 0.55 | 0.80 | 0.92 | 0.84 | 0.92 | 0.64 | NaN | NaN |
| A0A286YEX4 | Nuclear factor 1 C-type | NFIC | 361 | S | 0.96 | 86.51 | 0.50 | 0.57 | 0.55 | 0.82 | 0.67 | 0.79 | NaN | 0.57 | 0.55 |
| H0Y9X0 | Protein furry homolog-like | FRYL | 1142 | S | 0.86 | 89.23 | NaN | NaN | NaN | 1.51 | NaN | 1.33 | NaN | NaN | NaN |
| A0A286YFD6 | Ribonucleoside-diphosphate reductase subunit M2 | RRM2 | 20 | S | 1 | 122.2 | 1.13 | 1.09 | 1.01 | 0.98 | 1.13 | 1.18 | 1.03 | 1.07 | 1.00 |
| A0A286YFG8 | | | 128 | S | 1 | 41.09 | NaN | NaN | NaN | 0.91 | NaN | 0.92 | 1.36 | 1.37 | NaN |
| A0A2R8Y5E8 | Probable histidine--tRNA ligase, mitochondrial | HARS2 | 56 | S | 1 | 42.29 | 0.59 | 0.95 | NaN | NaN | NaN | NaN | NaN | NaN | NaN |
| A0A2R8Y5T7 | Glutathione synthetase | GSS | 301 | S | 1 | 52.06 | NaN | 0.64 | NaN | 1.27 | 1.07 | NaN | 1.27 | 1.11 | NaN |
| A0A2R8Y443 | Dual specificity tyrosine-phosphorylation-regulated kinase 1B;Dual specificity tyrosine-phosphorylation-regulated kinase 1A | DYRK1B | 286 | S | 0.788 | 45.72 | 0.71 | NaN | 0.91 | NaN | 0.84 | NaN | 0.90 | 0.93 | 0.91 |
| A0A2R8Y679 | Serine/threonine-protein kinase B-raf | BRAF | 128 | S | 0.6 | 50.95 | 1.09 | 0.77 | NaN | NaN | NaN | NaN | NaN | NaN | NaN |
| F5H777 | Coiled-coil domain-containing protein 82 | CCDC82 | 154 | S | 1 | 157.3 | 0.88 | 0.60 | NaN | NaN | NaN | NaN | NaN | NaN | NaN |
| A0A2R8Y4N5 | DnaJ homolog subfamily C member 21 | DNAJC21 | 254 | S | 1 | 339.3 | 0.58 | 0.53 | 0.70 | 0.95 | 0.87 | 0.91 | 0.73 | 0.67 | 0.71 |
| A0A2R8YF61 | Cyclin-dependent kinase 13 | CDK13 | 102 | S | 0.843 | 103.4 | NaN | 0.50 | 1.89 | NaN | NaN | NaN | NaN | NaN | NaN |
| A0A2R8Y4Z8 | Coiled-coil domain-containing protein 9 | CCDC9 | 386 | S | 0.882 | 165.7 | 1.05 | 1.17 | 1.01 | 0.86 | 0.85 | 0.86 | 0.91 | 1.00 | 1.18 |
| A0A2R8Y4Z8 | | | 521 | S | 1 | 123.7 | 1.89 | NaN | 2.12 | 1.74 | 1.90 | 1.77 | 1.12 | 1.24 | 1.22 |
| A0A2R8Y6I5 | Cytoplasmic dynein 1 heavy chain 1 | DYNC1H1 | 209 | S | 0.667 | 50.66 | 0.76 | NaN | NaN | 1.17 | 0.96 | NaN | 1.15 | NaN | NaN |
| A0A2R8Y7R5 | Deoxynucleoside triphosphate triphosphohydrolase SAMHD1 | SAMHD1 | 33 | S | 1 | 211.2 | 0.53 | 0.53 | 0.46 | 0.48 | 0.50 | 0.47 | 0.57 | NaN | 0.65 |
| H0YH87 | Ataxin-2 | ATXN2 | 701 | S | 0.934 | 166.1 | 1.13 | NaN | 1.09 | 1.33 | 1.35 | NaN | 1.25 | NaN | 1.45 |

| | | | | | | | | | | | | | | |
|---|---|---|---|---|---|---|---|---|---|---|---|---|---|---|
| H0YH87 | Ataxin-2 | ATXN2 | 705 | S | 0.979 | 166.1 | 1.13 | NaN | 1.09 | 1.33 | 1.35 | 1.37 | 1.25 | NaN | 1.45 |
| H0YH87 | Ataxin-2 | ATXN2 | 612 | S | 0.953 | 89.51 | 1.14 | 0.50 | 0.53 | NaN | NaN | 0.76 | NaN | 0.88 | 0.95 |
| H0YH87 | Ataxin-2 | ATXN2 | 598 | S | 0.86 | 63.1 | NaN | NaN | 0.65 | 1.11 | 1.06 | 0.75 | 0.62 | 0.67 | 0.94 |
| H3BQK4 | Tuberin | TSC2 | 157 | S | 1 | 138.1 | 0.33 | NaN | NaN | 0.48 | 0.62 | NaN | 0.44 | 1.09 | 1.42 |
| A0A494C1R1 | Putative Polycomb group protein ASXL1 | ASXH1 | 503 | S | 0.535 | 74.91 | NaN | NaN | NaN | NaN | NaN | NaN | 0.91 | 0.78 | NaN |
| A0A2R8Y611 | AP-3 complex subunit delta-1 | AP3D1 | 374 | S | 1 | 107.7 | 0.44 | 0.43 | NaN | NaN | NaN | NaN | NaN | NaN | NaN |
| A0A2R8Y611 | AP-3 complex subunit delta-1 | AP3D1 | 376 | S | 1 | 107.7 | 0.59 | 0.43 | NaN | NaN | NaN | NaN | NaN | NaN | NaN |
| A0A2R8Y611 | AP-3 complex subunit delta-1 | AP3D1 | 378 | S | 1 | 103.8 | 0.33 | 0.43 | NaN | NaN | NaN | NaN | NaN | NaN | NaN |
| A0A2R8Y611 | AP-3 complex subunit delta-1 | AP3D1 | 569 | S | 0.806 | 135.7 | 0.71 | NaN | NaN | 1.16 | 1.08 | NaN | NaN | 0.64 | NaN |
| A0A2R8Y611 | AP-3 complex subunit delta-1 | AP3D1 | 571 | S | 0.79 | 89.63 | NaN | NaN | 0.73 | NaN | NaN | NaN | 0.65 | NaN | 0.55 |
| H0Y8T6 | Rap guanine nucleotide exchange factor 2 | RAPGEF2 | 128 | S | 0.958 | 119.5 | 0.85 | NaN | 0.86 | 0.67 | 0.60 | 0.84 | 1.12 | 1.14 | NaN |
| Q5JS72 | | CASK | 192 | S | 0.991 | 284.3 | 1.13 | 1.09 | 1.33 | 1.01 | 0.88 | 1.03 | 1.14 | 1.21 | 1.39 |
| A0A2R8Y6G6 | Alpha-enolase | ENO1 | 419 | S | 1 | 48.66 | NaN | NaN | NaN | NaN | NaN | NaN | 0.49 | NaN | 0.72 |
| H0Y470 | Girdin | CCDC88A | 65 | S | 0.834 | 88.04 | NaN | NaN | NaN | 2.75 | 1.79 | NaN | NaN | NaN | 2.37 |
| H0Y470 | Girdin | CCDC88A | 670 | S | 0.994 | 66.32 | NaN | NaN | 1.02 | 0.85 | 0.86 | 0.77 | 1.14 | 1.16 | NaN |
| H0Y470 | Girdin | CCDC88A | 719 | S | 1 | 213.8 | 0.64 | NaN | 0.65 | NaN | NaN | 0.83 | 0.95 | 1.10 | 1.09 |
| F8WF45 | TATA element modulatory factor | TMF1 | 399 | S | 0.556 | 139.8 | 1.37 | 1.39 | 1.72 | 1.19 | 1.08 | 1.29 | 0.99 | 1.29 | NaN |
| F8WF45 | TATA element modulatory factor | TMF1 | 344 | S | 1 | 231.4 | 1.16 | 0.97 | 1.01 | 1.03 | 0.93 | 1.02 | 0.83 | 0.86 | 0.91 |
| A0A2R8Y7R9 | Liprin-alpha-1;Liprin-alpha-4 | PPFIA1 | 733 | S | 0.925 | 62.09 | 1.66 | 0.47 | 1.40 | 1.22 | 1.21 | 0.51 | 1.47 | NaN | NaN |
| A0A2R8Y7R9 | Liprin-alpha-1;Liprin-alpha-4 | PPFIA1 | 736 | S | 0.575 | 54.02 | 1.66 | 1.35 | 1.40 | 1.22 | 1.21 | NaN | 1.47 | NaN | NaN |
| A0A2R8Y7R9 | Liprin-alpha-1 | PPFIA1 | 224 | S | 0.995 | 73.14 | 2.05 | 1.55 | NaN | NaN | NaN | NaN | NaN | NaN | NaN |
| A0A2R8Y880 | E3 ubiquitin-protein ligase RBBP6 | RBBP6 | 282 | S | 1 | 57.3 | 0.84 | 0.70 | NaN | NaN | NaN | NaN | NaN | NaN | NaN |
| A0A2R8Y880 | E3 ubiquitin-protein ligase RBBP6 | RBBP6 | 283 | S | 1 | 57.3 | 0.84 | 0.70 | NaN | NaN | NaN | NaN | NaN | NaN | NaN |
| A0A2R8Y880 | E3 ubiquitin-protein ligase RBBP6 | RBBP6 | 284 | S | 1 | 57.3 | 0.84 | 0.70 | NaN | NaN | NaN | NaN | NaN | NaN | NaN |
| A0A2R8Y880 | E3 ubiquitin-protein ligase RBBP6 | RBBP6 | 285 | S | 1 | 57.3 | 0.84 | 0.70 | NaN | NaN | NaN | NaN | NaN | NaN | NaN |
| A0A2R8YCL1 | Growth factor receptor-bound protein 10 | GRB10 | 188 | S | 0.974 | 54.63 | NaN | 1.38 | NaN | NaN | 0.93 | NaN | 1.99 | NaN | 1.60 |
| C9JEF4 | Histone deacetylase 6 | HDAC6 | 22 | S | 1 | 127.7 | 0.45 | 0.35 | NaN | NaN | NaN | 0.65 | 1.14 | 1.12 | 1.00 |
| A0A2R8YDS4 | Nitric oxide synthase, | NOS2 | 887 | S | 0.958 | 42.52 | NaN | NaN | 0.59 | 1.19 | 1.15 | NaN | NaN | 0.49 | NaN |
| E9PDQ5 | Rho guanine nucleotide exchange factor 7 | ARHGEF 7 | 420 | S | 0.907 | 63.09 | NaN | 1.05 | 1.10 | 1.10 | 0.97 | 1.08 | NaN | 1.27 | NaN |
| E9PDQ5 | Rho guanine nucleotide exchange factor 7;Rho guanine nucleotide exchange | ARHGEF 7 | 262 | S | 1 | 92.44 | 1.76 | 1.53 | 1.79 | 2.27 | 2.05 | 1.97 | NaN | 0.60 | 0.67 |
| A0A2U3TZL8 | Kinesin-like protein;Kinesin-like protein KIF23 | KIF23 | 717 | S | 1 | 123.3 | 0.23 | 0.20 | NaN | 0.36 | NaN | 0.34 | 0.38 | 0.40 | 0.50 |
| C9J7T7 | Nuclear receptor corepressor | NCOR2 | 496 | S | 1 | 56.6 | NaN | NaN | 2.30 | NaN | NaN | NaN | 1.86 | 2.60 | 2.31 |
| Q5JU47 | TBC1 domain family member | TBC1D4 | 103 | S | 0.998 | 62.34 | 0.73 | NaN | 1.23 | NaN | 0.89 | NaN | 2.33 | 2.47 | NaN |
| Q5JU47 | TBC1 domain family member | TBC1D4 | 200 | S | 0.838 | 177.1 | NaN | 2.15 | NaN | NaN | NaN | NaN | 1.92 | NaN | 2.26 |

| | | | | | | | | | | | | | | |
|---|---|---|---|---|---|---|---|---|---|---|---|---|---|---|
| Q5JU47 | TBC1 domain family member | TBC1D4 | 203 | S | 1 | 249.3 | 1.57 | 0.69 | 0.72 | 2.17 | 0.89 | NaN | 0.97 | 0.70 | 2.26 |
| C9JBX5 | Translocon-associated protein subunit alpha | SSR1 | 200 | S | 1 | 163.9 | 1.91 | 1.66 | NaN | 1.91 | NaN | 1.93 | NaN | 0.98 | 0.61 |
| A0A3B3IS71 | Retinoblastoma-associated protein | RB1 | 807 | S | 1 | 86.54 | 1.41 | NaN | 0.83 | 0.77 | 0.91 | 0.81 | 0.54 | 0.54 | 0.54 |
| A0A3B3IS71 | Retinoblastoma-associated protein | RB1 | 37 | S | 1 | 123.6 | 0.78 | 0.77 | 0.84 | 0.90 | 1.01 | 0.89 | 0.77 | 0.67 | 0.70 |
| A0A3B3IS71 | Retinoblastoma-associated protein | RB1 | 249 | S | 1 | 71.51 | 1.10 | 1.51 | NaN | NaN | NaN | NaN | NaN | NaN | NaN |
| A0A3B3IU78 | Coatomer subunit alpha;Xenin;Proxenin | COPA | 43 | S | 1 | 66.57 | 1.75 | 1.88 | 1.51 | NaN | NaN | NaN | 0.67 | NaN | NaN |
| A0A3B3ISN0 | | | 190 | S | 0.99 | 81.77 | 2.13 | 2.28 | 2.43 | NaN | 1.10 | 1.03 | 3.04 | 3.11 | 3.13 |
| A0A3B3ISQ4 | Epidermal growth factor receptor kinase substrate 8-like protein 2 | EPS8L2 | 479 | S | 0.785 | 114.7 | 1.01 | NaN | NaN | NaN | NaN | 1.00 | 1.06 | 1.04 | NaN |
| A0A3B3ISQ4 | Epidermal growth factor receptor kinase substrate 8-like protein 2 | EPS8L2 | 459 | S | 0.794 | 114.7 | NaN | NaN | NaN | NaN | NaN | NaN | 0.74 | 1.04 | NaN |
| A0A3B3ISQ4 | Epidermal growth factor receptor kinase substrate 8-like protein 2 | EPS8L2 | 570 | S | 0.993 | 121.9 | 0.91 | 0.89 | 0.95 | 1.06 | 0.95 | 1.00 | 1.10 | 1.05 | 1.16 |
| A0A3B3ISZ4 | Polycomb group RING finger protein 6 | PCGF6 | 115 | S | 1 | 103.7 | NaN | NaN | NaN | 0.75 | NaN | NaN | 0.89 | 0.79 | 0.77 |
| A0A3B3ITD8 | Nuclear pore complex protein Nup98-Nup96;Nuclear pore complex protein Nup98;Nuclear pore complex | NUP98 | 606 | S | 1 | 312.6 | 1.80 | 1.60 | 2.08 | 1.55 | 1.52 | 1.43 | 1.80 | 1.89 | 1.49 |
| A0A3B3ITD8 | Nuclear pore complex protein Nup98-Nup96;Nuclear pore complex protein Nup98;Nuclear pore complex | NUP98 | 871 | S | 1 | 235.5 | 0.88 | 0.81 | 0.86 | 0.91 | 0.86 | 0.76 | 1.01 | 0.99 | 1.05 |
| A0A3B3ITE3 | Gamma-tubulin complex component 3 | TUBGCP 3 | 515 | S | 0.851 | 211.6 | 0.59 | 1.62 | NaN | NaN | 0.61 | NaN | NaN | 0.68 | NaN |
| H0Y3A3 | Disabled homolog 2-interacting protein | DAB2IP | 887 | S | 1 | 118.8 | NaN | NaN | NaN | 0.21 | 0.34 | NaN | 0.38 | 0.48 | 0.66 |
| A0A3B3ITG6 | Translation initiation factor eIF-2B subunit epsilon | EIF2B5 | 500 | S | 1 | 128.2 | 0.53 | 0.56 | NaN | 0.80 | 0.79 | NaN | NaN | 0.65 | 0.65 |
| A0A3B3ITK7 | Phosphoglucomutase-1 | PGM1 | 117 | S | 1 | 156.1 | 1.09 | 1.07 | NaN | NaN | NaN | NaN | NaN | 1.23 | NaN |
| A0A3B3IU46 | RNMT-activating mini protein | FAM103A 1 | 36 | S | 1 | 90.02 | 1.23 | 1.16 | NaN | 1.42 | 1.35 | NaN | 0.82 | 0.89 | NaN |
| A0A3B3IUA7 | E3 ubiquitin/ISG15 ligase TRIM25 | TRIM25 | 100 | S | 1 | 171.5 | 1.11 | 1.09 | 1.27 | 1.75 | 1.72 | 1.32 | 0.96 | 0.88 | 0.43 |
| A0A3F2YNX0 | Protein transport protein Sec16A | SEC16A | 1905 | S | 0.967 | 55.34 | NaN | 1.84 | 1.85 | 1.76 | 1.53 | NaN | 1.27 | 1.33 | NaN |
| A0A3F2YNX0 | Protein transport protein Sec16A | SEC16A | 891 | S | 1 | 86.08 | 0.72 | 0.72 | 0.72 | 0.83 | 0.83 | 0.88 | 0.92 | 0.94 | 0.87 |

| | | | | | | | | | | | | | | | |
|---|---|---|---|---|---|---|---|---|---|---|---|---|---|---|---|
| A0A3F2YNX0 | Protein transport protein Sec16A | SEC16A | 417 | S | 1 | 130.9 | 0.73 | 0.54 | 0.66 | 0.63 | 1.04 | NaN | 1.10 | NaN | NaN |
| A0A3F2YNX0 | Protein transport protein Sec16A | SEC16A | 1786 | S | 1 | 88.32 | NaN | 3.42 | 3.56 | 2.48 | 3.00 | 2.77 | 1.89 | NaN | 2.68 |
| A0A3F2YNX0 | Protein transport protein Sec16A | SEC16A | 390 | S | 0.635 | 118.8 | NaN | 1.09 | NaN | NaN | NaN | NaN | 1.03 | 1.14 | NaN |
| A0A3F2YNX0 | Protein transport protein Sec16A | SEC16A | 391 | S | 0.971 | 225.1 | NaN | NaN | 0.78 | 0.98 | 1.10 | 0.92 | NaN | 0.87 | NaN |
| A0A3F2YNY6 | Pre-mRNA-processing factor 40 homolog A | PRPF40A | 975 | S | 0.999 | 169.4 | NaN | NaN | NaN | 0.48 | NaN | 0.51 | 1.41 | NaN | 0.36 |
| A0A3F2YNY6 | Pre-mRNA-processing factor 40 homolog A | PRPF40A | 920 | S | 1 | 93.92 | 0.94 | 0.89 | 0.88 | 0.93 | 0.89 | 0.87 | 0.91 | 0.88 | 0.93 |
| A0A3F2YNY6 | Pre-mRNA-processing factor 40 homolog A | PRPF40A | 922 | S | 1 | 93.92 | 0.94 | 0.89 | 0.88 | 0.93 | 0.89 | 0.87 | 0.91 | 0.88 | 0.93 |
| A0A3F2YNY6 | Pre-mRNA-processing factor 40 homolog A | PRPF40A | 925 | S | 1 | 93.92 | 0.94 | 1.78 | 2.13 | 0.93 | 0.89 | 0.87 | 0.91 | 0.88 | 0.93 |
| A0A494BZT8 | Transforming acidic coiled-coil-containing protein 3 | TACC3 | 250 | S | 0.745 | 81.14 | 2.07 | NaN | NaN | 3.66 | 3.19 | 3.22 | NaN | NaN | NaN |
| A0A494BZT8 | Transforming acidic coiled-coil-containing protein 3 | TACC3 | 434 | S | 0.991 | 149.9 | 3.12 | 2.83 | NaN | NaN | 3.24 | NaN | NaN | NaN | NaN |
| A0A494BZV2 | Myosin phosphatase Rho-interacting protein | MPRIP | 2364 | S | 1 | 194.1 | 0.77 | 0.77 | 0.98 | 1.28 | 1.59 | 1.19 | 0.44 | 0.53 | 0.71 |
| A0A494BZV2 | Myosin phosphatase Rho-interacting protein | MPRIP | 289 | S | 0.999 | 81.53 | 1.37 | 1.42 | 1.40 | 1.30 | NaN | 1.15 | 1.45 | 1.44 | 1.12 |
| A0A494BZV2 | Myosin phosphatase Rho-interacting protein | MPRIP | 292 | S | 0.819 | 81.53 | 1.37 | 1.42 | 1.40 | 1.30 | NaN | NaN | 1.45 | NaN | NaN |
| A0A494BZV2 | Myosin phosphatase Rho-interacting protein | MPRIP | 483 | S | 0.672 | 197.4 | 2.00 | 1.97 | NaN | NaN | 2.09 | 1.14 | NaN | NaN | NaN |
| A0A494BZV2 | Myosin phosphatase Rho-interacting protein | MPRIP | 220 | S | 0.969 | 166.1 | 1.45 | 1.15 | 1.57 | 1.69 | 1.19 | 1.65 | 1.11 | 1.76 | 1.06 |
| A0A494BZV2 | Myosin phosphatase Rho-interacting protein | MPRIP | 224 | S | 0.998 | 166.1 | 1.45 | 1.15 | 1.54 | 1.57 | 1.22 | 1.65 | 1.00 | 1.46 | 1.04 |
| A0A494BZV2 | Myosin phosphatase Rho-interacting protein | MPRIP | 737 | S | 1 | 121.6 | 1.37 | 1.29 | 1.24 | NaN | 1.80 | 1.69 | 0.84 | 0.81 | 0.74 |
| A0A494BZV2 | Myosin phosphatase Rho-interacting protein | MPRIP | 2380 | S | 0.998 | 84.76 | NaN | 1.29 | 1.33 | 1.43 | 1.41 | 1.34 | 2.00 | 1.76 | 1.74 |
| A0A494C050 | NF-kappa-B-activating protein | NKAP | 149 | S | 1 | 111.7 | 0.99 | 1.05 | 1.07 | 0.86 | 1.01 | 1.13 | 0.96 | 1.00 | 0.95 |
| H0Y6K5 | Transcription factor Sp3 | SP3 | 5 | S | 1 | 128.3 | 0.94 | NaN | NaN | 0.76 | NaN | 0.89 | NaN | NaN | 1.47 |
| D6RC77 | Phosphoacetylglucosamine mutase | PGM3 | 92 | S | 0.751 | 203.3 | 0.47 | 0.48 | NaN | 0.72 | NaN | NaN | NaN | 0.47 | NaN |
| A0A494C0B4 | UV excision repair protein RAD23 homolog A | RAD23A | 128 | S | 0.761 | 117.4 | NaN | NaN | NaN | 0.76 | NaN | 0.76 | NaN | NaN | NaN |
| A0A494C0B4 | UV excision repair protein RAD23 homolog A | RAD23A | 133 | S | 0.86 | 82.51 | 0.50 | NaN | 0.57 | NaN | NaN | NaN | NaN | NaN | NaN |
| A0A494C0Q6 | Poly(A)-specific ribonuclease PARN | PARN | 561 | S | 1 | 73.35 | 1.05 | NaN | 1.60 | NaN | 1.12 | 1.66 | 1.30 | NaN | 1.61 |

| | | | | | | | | | | | | | | | |
|---|---|---|---|---|---|---|---|---|---|---|---|---|---|---|---|
| H7BZ50 | Mitotic-spindle organizing protein 2B | MZT2B | 115 | S | 0.949 | 86.26 | 0.87 | NaN | 0.96 | NaN | NaN | NaN | 1.01 | 0.91 | NaN |
| A0A494C108 | Protein KRI1 homolog | KRI1 | 177 | S | 1 | 327.6 | 1.14 | 1.13 | 1.14 | 1.10 | 1.23 | 1.22 | 1.13 | 1.27 | 1.20 |
| A0A494C108 | Protein KRI1 homolog | KRI1 | 634 | S | 1 | 173.6 | 0.51 | 0.43 | 0.65 | 0.44 | 0.52 | 1.06 | 0.61 | 0.75 | 0.88 |
| A0A494C108 | Protein KRI1 homolog | KRI1 | 645 | S | 1 | 148.4 | NaN | 0.77 | 1.11 | 1.22 | NaN | 1.06 | 0.66 | 0.61 | NaN |
| A0A494C108 | Protein KRI1 homolog | KRI1 | 142 | S | 1 | 78.09 | 1.29 | NaN | 1.40 | 1.04 | 1.17 | NaN | NaN | 1.01 | NaN |
| A0A494C198 | Protein DEK | DEK | 229 | S | 1 | 124.1 | 0.75 | 0.83 | 0.80 | 0.89 | 0.90 | 0.86 | 0.84 | 0.89 | 0.84 |
| A0A494C198 | Protein DEK | DEK | 231 | S | 1 | 124.1 | 0.75 | 0.83 | 0.80 | 0.89 | 0.90 | 0.86 | 0.84 | 0.82 | 0.81 |
| A0A494C198 | Protein DEK | DEK | 234 | S | 1 | 124.1 | 0.75 | 0.83 | 0.80 | 0.89 | 0.91 | 0.85 | 0.82 | 0.82 | 0.81 |
| A0A494C198 | Protein DEK | DEK | 235 | S | 1 | 124.1 | 0.75 | 0.83 | 0.80 | 0.89 | 0.91 | 0.85 | 0.82 | 0.82 | 0.81 |
| A0A494C1A5 | U5 small nuclear ribonucleoprotein 200 kDa helicase | SNRNP200 | 225 | S | 1 | 440.6 | 0.62 | 0.64 | 0.62 | 0.83 | 0.70 | 0.77 | 0.67 | 0.66 | 0.67 |
| A0A494C1F2 | Nuclear pore complex protein Nup214 | NUP214 | 666 | S | 1 | 85.62 | 0.89 | 0.80 | 0.82 | 0.94 | 0.96 | 0.95 | 0.84 | 0.85 | 0.87 |
| A0A494C1F2 | Nuclear pore complex protein Nup214 | NUP214 | 430 | S | 0.983 | 132.4 | 0.46 | 0.42 | 1.58 | 0.60 | 1.38 | 1.27 | NaN | 0.63 | NaN |
| A0A494C1F2 | Nuclear pore complex protein Nup214 | NUP214 | 433 | S | 0.829 | 71.4 | NaN | NaN | NaN | NaN | NaN | NaN | 0.66 | NaN | 0.64 |
| C9J8U1 | Cytospin-A | SPECC1L | 384 | S | 1 | 110.6 | 0.71 | NaN | NaN | NaN | 0.91 | 0.46 | 1.00 | 1.14 | 1.36 |
| C9J8U1 | Cytospin-A | SPECC1L | 385 | S | 1 | 110.6 | 0.71 | NaN | NaN | NaN | 0.91 | 0.46 | 1.00 | 1.14 | 1.36 |
| A0A494C1K3 | General transcription factor II-I | GTF2I | 984 | S | 0.999 | 154.9 | 0.59 | 0.63 | 0.61 | 0.74 | 0.73 | 0.69 | 0.88 | 0.83 | 0.85 |
| C9JXK0 | Lamin-B receptor | LBR | 84 | S | 1 | 94.69 | NaN | 0.95 | 1.05 | NaN | 0.99 | 0.99 | NaN | NaN | NaN |
| C9JXK0 | Lamin-B receptor | LBR | 86 | S | 1 | 87.22 | 0.35 | 0.95 | 1.05 | NaN | 0.99 | 0.99 | NaN | NaN | NaN |
| Q7Z6P5 | DNA replication licensing factor MCM3 | MCM3 | 206 | S | 0.991 | 246.4 | 0.59 | 0.60 | 0.62 | 0.85 | 0.88 | 0.78 | 0.46 | 0.46 | 0.48 |
| Q7Z6P5 | DNA replication licensing factor MCM3 | MCM3 | 167 | S | 1 | 196.8 | 0.82 | 0.83 | NaN | NaN | NaN | NaN | 3.06 | 2.94 | 1.06 |
| H0Y917 | WD repeat-containing protein 26 | WDR26 | 113 | S | 0.999 | 82.65 | 0.76 | 1.08 | NaN | NaN | 1.26 | NaN | NaN | NaN | NaN |
| A0A499FJI4 | E3 ubiquitin-protein ligase MYCBP2 | MYCBP2 | 2751 | S | 0.972 | 64.24 | 0.57 | 0.78 | NaN | 0.68 | 0.63 | NaN | NaN | NaN | NaN |
| A0A499FJI4 | E3 ubiquitin-protein ligase MYCBP2 | MYCBP2 | 3467 | S | 0.965 | 47.64 | NaN | NaN | NaN | NaN | 1.49 | NaN | 1.65 | 1.41 | NaN |
| A0A499FJS7 | Inhibitor of nuclear factor kappa-B kinase subunit beta | IKBKB | 608 | S | 0.932 | 66.51 | NaN | NaN | 0.78 | 1.09 | 0.95 | 1.04 | NaN | 0.65 | 0.66 |
| A0A4W8VX11 | Pericentriolar material 1 | PCM1 | 1676 | S | 1 | 167.6 | 0.50 | 0.60 | NaN | 0.43 | NaN | 0.84 | NaN | 0.72 | NaN |
| A0A4W8VX11 | Pericentriolar material 1 | PCM1 | 1711 | S | 1 | 156.6 | 0.62 | 1.46 | 1.15 | 0.73 | 1.34 | 0.69 | 1.41 | 1.35 | 1.73 |
| A0A4W8VX11 | Pericentriolar material 1 | PCM1 | 1714 | S | 1 | 123.2 | 1.52 | 1.46 | 0.62 | 1.38 | 1.34 | 1.04 | 0.67 | 1.35 | 0.62 |
| A0A4W8VX11 | Pericentriolar material 1 | PCM1 | 862 | S | 0.999 | 150 | NaN | NaN | 0.41 | 0.46 | NaN | NaN | 0.55 | NaN | 0.79 |
| A0A4W8VX11 | Pericentriolar material 1 | PCM1 | 65 | S | 0.998 | 172.6 | 0.76 | 0.80 | 0.79 | 0.76 | 0.73 | 0.75 | 0.89 | 0.95 | 0.97 |
| H0Y8W5 | Rho GTPase-activating protein 10 | ARHGAP10 | 269 | S | 0.981 | 67.08 | NaN | NaN | 1.29 | NaN | NaN | 1.14 | 1.09 | NaN | 1.15 |
| A1L170 | Uncharacterized protein C1orf226 | C1orf226 | 223 | S | 0.989 | 78.34 | 1.15 | 1.21 | NaN | 1.23 | 1.37 | 1.27 | NaN | 1.34 | 1.30 |
| A1L170 | Uncharacterized protein C1orf226 | C1orf226 | 249 | S | 1 | 150 | 1.18 | 1.22 | 1.34 | 1.20 | 1.07 | 1.03 | 1.05 | 1.14 | 0.89 |

| | | | | | | | | | | | | | | | |
|---|---|---|---|---|---|---|---|---|---|---|---|---|---|---|---|
| A2A2D0 | Stathmin | STMN1 | 16 | S | 1 | 222.4 | 2.01 | 2.10 | 2.12 | 2.50 | 2.50 | NaN | 1.50 | 1.40 | 1.59 |
| A2A2D0 | Stathmin | STMN1 | 25 | S | 1 | 455.1 | 5.68 | 5.70 | 5.91 | 6.11 | 6.27 | 6.48 | 1.66 | 1.64 | 1.63 |
| A2A2D0 | Stathmin;Stathmin-2 | STMN1 | 46 | S | 1 | 96.24 | 1.77 | NaN | NaN | 2.56 | 2.68 | NaN | 0.76 | 0.79 | NaN |
| A2A2D0 | Stathmin | STMN1 | 38 | S | 1 | 200.8 | 1.38 | 1.36 | 1.37 | 2.14 | 2.18 | 2.01 | 0.73 | 0.68 | 0.75 |
| A2A2D0 | Stathmin | STMN1 | 63 | S | 1 | 130.7 | 2.48 | 2.29 | 2.66 | 4.42 | 3.31 | 3.89 | 0.66 | 0.59 | 0.71 |
| H7C5M1 | RNA-binding protein 34 | RBM34 | 31 | S | 1 | 110 | NaN | NaN | NaN | 0.79 | 0.55 | NaN | NaN | NaN | NaN |
| A2AAT0 | Zinc transporter SLC39A7 | SLC39A7 | 185 | S | 1 | 132.7 | 0.93 | 0.99 | 0.92 | 0.97 | 0.97 | 0.97 | 0.95 | 0.99 | 0.99 |
| A2AAT0 | Zinc transporter SLC39A7 | SLC39A7 | 186 | S | 1 | 132.7 | 0.93 | 0.96 | 0.92 | 0.97 | 0.97 | 0.97 | 0.95 | 0.99 | 0.99 |
| A2RU67 | Uncharacterized protein KIAA1467 | KIAA1467 | 30 | S | 1 | 104.3 | 1.90 | 1.44 | NaN | 1.59 | NaN | NaN | NaN | NaN | NaN |
| A2RU67 | Uncharacterized protein KIAA1467 | KIAA1467 | 33 | S | 0.999 | 104.3 | 1.90 | 1.44 | NaN | 1.59 | NaN | NaN | NaN | NaN | NaN |
| A6NL93 | Non-histone chromosomal protein HMG-14 | HMGN1 | 76 | S | 0.999 | 170.9 | 0.67 | 0.66 | 0.72 | 0.69 | 0.52 | 0.79 | 0.84 | 1.01 | 0.63 |
| A6NL93 | Non-histone chromosomal protein HMG-14 | HMGN1 | 79 | S | 1 | 152.6 | 0.59 | 0.64 | 0.69 | 0.60 | 0.55 | 0.58 | 0.72 | 0.83 | 0.77 |
| A6NL93 | Non-histone chromosomal protein HMG-14 | HMGN1 | 89 | S | 1 | 129.8 | 0.80 | 0.72 | NaN | 0.81 | 0.71 | 0.68 | 0.64 | 0.72 | 0.71 |
| A6NGP5 | Hematological and neurological expressed 1-like | HN1L | 18 | S | 0.998 | 156.3 | 2.37 | NaN | 2.34 | 5.13 | 4.25 | 6.13 | 0.81 | 0.61 | NaN |
| P07355 | Annexin A2;Putative annexin A2-like protein;Annexin | ANXA2 | 26 | S | 0.774 | 154.1 | NaN | NaN | 0.74 | NaN | 0.90 | NaN | NaN | 0.80 | 0.85 |
| A6NNK5 | Tumor suppressor p53-binding protein 1 | TP53BP1 | 1381 | S | 1 | 92.9 | 0.73 | 0.61 | NaN | 0.95 | 1.22 | 0.99 | 0.99 | 1.21 | NaN |
| A6NNK5 | Tumor suppressor p53-binding protein 1 | TP53BP1 | 1385 | S | 1 | 92.9 | 0.53 | 0.61 | NaN | 0.71 | 1.22 | 0.99 | 0.64 | 0.96 | 1.02 |
| A6NNK5 | Tumor suppressor p53-binding protein 1 | TP53BP1 | 585 | S | 0.979 | 135.9 | 0.61 | 0.52 | NaN | 0.96 | NaN | 0.91 | 1.03 | NaN | 0.97 |
| A6NNK5 | Tumor suppressor p53-binding protein 1 | TP53BP1 | 557 | S | 0.998 | 121.7 | 0.48 | 0.46 | 0.50 | 0.62 | 0.63 | 0.76 | 0.62 | 0.59 | 0.63 |
| A6NNK5 | Tumor suppressor p53-binding protein 1 | TP53BP1 | 1118 | S | 0.572 | 106.2 | 0.38 | 0.37 | NaN | 0.61 | 0.61 | 0.43 | NaN | 0.47 | NaN |
| A6NNK5 | Tumor suppressor p53-binding protein 1 | TP53BP1 | 1119 | S | 0.994 | 212.4 | 0.38 | 0.32 | 0.36 | 0.54 | 0.61 | 0.59 | 0.42 | 0.43 | 0.42 |
| A6NNK5 | Tumor suppressor p53-binding protein 1 | TP53BP1 | 505 | S | 1 | 160.8 | 0.54 | 0.49 | 0.66 | 0.95 | 0.86 | 0.69 | 0.98 | 1.04 | 0.98 |
| A6NNK5 | Tumor suppressor p53-binding protein 1 | TP53BP1 | 1099 | S | 1 | 150.6 | 0.44 | 0.47 | NaN | 0.54 | 0.57 | 0.49 | 0.50 | 0.50 | 0.49 |
| A6NNK5 | Tumor suppressor p53-binding protein 1 | TP53BP1 | 1106 | S | 0.975 | 125 | 0.44 | 0.46 | NaN | 0.84 | 0.72 | 0.49 | NaN | NaN | 0.49 |
| A6NNK5 | Tumor suppressor p53-binding protein 1 | TP53BP1 | 1109 | S | 0.836 | 150.6 | NaN | NaN | NaN | NaN | NaN | NaN | 0.52 | 0.55 | NaN |
| A6NNK5 | Tumor suppressor p53-binding protein 1 | TP53BP1 | 270 | S | 1 | 97.91 | 0.64 | 0.62 | NaN | 0.99 | 0.89 | 0.93 | 0.89 | 0.94 | 0.86 |
| A6NNK5 | Tumor suppressor p53-binding protein 1 | TP53BP1 | 1713 | S | 0.963 | 141.1 | NaN | 0.59 | 0.90 | 0.87 | NaN | 0.84 | 1.11 | 1.04 | 1.07 |

| | | | | | | | | | | | | | | |
|---|---|---|---|---|---|---|---|---|---|---|---|---|---|---|
| A6NNK5 | Tumor suppressor p53-binding protein 1 | TP53BP1 | 1714 | S | 0.988 | 141.1 | NaN | 0.59 | 0.90 | 0.87 | NaN | 0.84 | 1.11 | 1.04 | 1.07 |
| A6NNK5 | Tumor suppressor p53-binding protein 1 | TP53BP1 | 385 | S | 0.938 | 166.5 | 0.68 | NaN | 0.75 | 0.67 | 0.85 | NaN | 1.01 | 0.82 | 1.37 |
| G3V174 | Rho GTPase-activating protein 32 | ARHGAP 32 | 782 | S | 1 | 169.9 | NaN | NaN | NaN | NaN | NaN | NaN | 1.87 | 1.82 | 2.67 |
| G3V174 | Rho GTPase-activating protein 32 | ARHGAP 32 | 797 | S | 0.999 | 204 | 1.08 | 0.92 | 0.90 | 0.91 | 0.93 | 0.59 | 1.16 | 1.39 | 1.18 |
| A8MQ02 | Afadin | MLLT4 | 1736 | S | 1 | 208.5 | 0.61 | 0.62 | 0.68 | 0.80 | NaN | 0.79 | 1.03 | 1.04 | 0.88 |
| A8MQ02 | Afadin | MLLT4 | 1141 | S | 1 | 100.4 | NaN | 0.66 | NaN | NaN | NaN | 0.93 | NaN | 0.99 | 1.27 |
| A8MQ02 | Afadin | MLLT4 | 1042 | S | 0.991 | 107.2 | 3.79 | NaN | NaN | 2.99 | NaN | 3.33 | 0.86 | NaN | NaN |
| H7BY10 | 60S ribosomal protein L23a | RPL23A | 45 | S | 0.715 | 60.75 | 1.26 | 1.25 | NaN | NaN | NaN | NaN | NaN | NaN | NaN |
| A8MZ87 | Kinesin light chain 2 | KLC2 | 306 | S | 0.991 | 63.91 | NaN | 0.95 | 1.13 | 1.24 | NaN | 1.38 | 1.20 | 0.96 | 2.00 |
| A9Z1X7 | Serine/arginine repetitive matrix protein 1 | SRRM1 | 747 | S | 1 | 120.2 | 1.04 | 1.18 | 1.02 | 0.80 | 1.04 | 0.75 | 1.01 | 1.13 | 0.85 |
| A9Z1X7 | Serine/arginine repetitive matrix protein 1 | SRRM1 | 749 | S | 1 | 98.44 | 1.09 | 1.11 | 1.16 | 1.07 | 1.04 | 1.03 | 1.26 | 1.13 | 1.19 |
| A9Z1X7 | Serine/arginine repetitive matrix protein 1 | SRRM1 | 800 | S | 1 | 61.1 | 0.67 | 0.70 | NaN | NaN | 0.82 | 0.85 | NaN | 1.06 | NaN |
| A9Z1X7 | Serine/arginine repetitive matrix protein 1 | SRRM1 | 804 | S | 1 | 61.1 | 0.67 | 0.70 | NaN | NaN | 0.82 | 0.85 | NaN | 1.06 | NaN |
| A9Z1X7 | Serine/arginine repetitive matrix protein 1 | SRRM1 | 806 | S | 1 | 61.1 | 0.67 | 0.70 | NaN | NaN | 0.82 | 0.85 | NaN | 1.06 | NaN |
| A9Z1X7 | Serine/arginine repetitive matrix protein 1 | SRRM1 | 703 | S | 0.733 | 53.4 | NaN | NaN | NaN | 1.08 | 0.98 | 0.96 | NaN | 1.23 | 1.18 |
| A9Z1X7 | Serine/arginine repetitive matrix protein 1 | SRRM1 | 704 | S | 0.965 | 121.6 | NaN | NaN | 1.11 | 1.08 | 1.01 | 0.96 | 0.96 | 0.93 | 0.85 |
| A9Z1X7 | Serine/arginine repetitive matrix protein 1 | SRRM1 | 705 | S | 0.948 | 191 | 1.15 | 1.10 | NaN | 1.04 | NaN | NaN | 1.15 | 1.12 | NaN |
| A9Z1X7 | Serine/arginine repetitive matrix protein 1 | SRRM1 | 684 | S | 1 | 95.48 | 1.20 | 1.24 | 1.27 | 1.04 | 1.17 | 1.05 | NaN | 1.02 | NaN |
| A9Z1X7 | Serine/arginine repetitive matrix protein 1 | SRRM1 | 397 | S | 1 | 152 | 0.41 | 0.39 | 0.43 | 0.59 | 0.56 | 0.57 | 0.50 | 0.50 | 0.51 |
| A9Z1X7 | Serine/arginine repetitive matrix protein 1 | SRRM1 | 883 | S | 1 | 180 | 1.38 | 1.48 | 1.59 | 1.42 | 1.67 | 1.53 | 1.29 | 1.77 | 1.50 |
| A9Z1X7 | Serine/arginine repetitive matrix protein 1 | SRRM1 | 778 | S | 1 | 203.3 | 0.67 | 0.76 | 0.74 | 0.88 | 0.79 | 0.93 | 0.52 | 0.59 | 0.60 |
| A9Z1X7 | Serine/arginine repetitive matrix protein 1 | SRRM1 | 784 | S | 1 | 203.3 | 0.89 | 0.84 | 0.86 | 0.96 | 1.03 | 0.94 | 0.86 | 0.85 | 0.87 |
| A9Z1X7 | Serine/arginine repetitive matrix protein 1 | SRRM1 | 790 | S | 0.849 | 88.84 | NaN | NaN | 1.23 | 1.24 | 0.83 | 1.32 | 1.03 | 1.07 | 1.56 |
| A9Z1X7 | Serine/arginine repetitive matrix protein 1 | SRRM1 | 458 | S | 1 | 104.3 | 1.43 | 1.48 | 0.65 | 1.25 | 1.32 | 1.27 | 1.07 | 1.24 | 0.77 |
| A9Z1X7 | Serine/arginine repetitive matrix protein 1 | SRRM1 | 460 | S | 1 | 104.3 | 1.43 | 1.48 | 1.13 | 1.25 | 1.32 | 1.27 | 1.07 | 1.24 | 1.31 |
| A9Z1X7 | Serine/arginine repetitive matrix protein 1 | SRRM1 | 654 | S | 0.755 | 59.37 | NaN | 1.01 | NaN | 1.01 | 1.09 | NaN | 1.09 | NaN | 1.15 |

| | | | | | | | | | | | | | | | |
|---|---|---|---|---|---|---|---|---|---|---|---|---|---|---|---|
| A9Z1X7 | Serine/arginine repetitive matrix protein 1 | SRRM1 | 635 | S | 1 | 65.3 | 0.90 | 0.96 | 0.97 | 0.92 | 0.79 | 0.89 | 0.99 | 0.91 | 0.85 |
| A9Z1X7 | Serine/arginine repetitive matrix protein 1 | SRRM1 | 637 | S | 1 | 65.3 | 0.90 | 0.96 | 0.97 | 0.92 | 0.79 | 0.89 | 0.99 | 0.91 | 0.85 |
| A9Z1X7 | Serine/arginine repetitive matrix protein 1 | SRRM1 | 445 | S | 0.999 | 131.9 | 1.21 | 1.11 | 1.18 | 1.08 | 1.08 | 1.07 | 1.11 | 1.10 | 1.13 |
| A9Z1X7 | Serine/arginine repetitive matrix protein 1 | SRRM1 | 384 | S | 1 | 128.1 | 0.70 | 0.70 | 0.72 | 1.38 | 0.74 | 0.71 | 0.76 | 0.78 | 0.80 |
| A9Z1X7 | Serine/arginine repetitive matrix protein 1 | SRRM1 | 386 | S | 1 | 117.2 | 0.70 | 0.70 | 0.72 | 0.72 | 0.76 | 0.71 | NaN | 0.78 | NaN |
| A9Z1X7 | Serine/arginine repetitive matrix protein 1 | SRRM1 | 388 | S | 1 | 128.1 | 1.65 | 1.91 | 1.76 | 0.72 | 0.76 | NaN | 0.76 | 0.78 | 0.80 |
| A9Z1X7 | Serine/arginine repetitive matrix protein 1 | SRRM1 | 722 | S | 1 | 78.69 | 0.97 | 1.23 | 1.11 | 1.00 | NaN | 0.98 | 0.97 | 0.99 | 0.93 |
| A9Z1X7 | Serine/arginine repetitive matrix protein 1 | SRRM1 | 724 | S | 0.996 | 68.28 | 0.87 | NaN | 1.15 | 1.06 | 1.09 | 1.01 | NaN | 0.98 | NaN |
| A9Z1X7 | Serine/arginine repetitive matrix protein 1 | SRRM1 | 726 | S | 0.75 | 65.04 | NaN | NaN | 1.02 | 1.08 | NaN | NaN | 1.21 | 0.93 | 0.96 |
| A9Z1X7 | Serine/arginine repetitive matrix protein 1 | SRRM1 | 662 | S | 1 | 115.3 | 1.00 | 0.99 | 1.02 | 0.96 | 0.99 | 0.97 | 0.95 | NaN | 0.96 |
| A9Z1X7 | Serine/arginine repetitive matrix protein 1 | SRRM1 | 569 | S | 1 | 100.8 | 0.52 | 0.57 | 0.54 | 0.78 | 0.78 | 0.68 | 0.61 | 0.61 | 0.55 |
| A9Z1X7 | Serine/arginine repetitive matrix protein 1 | SRRM1 | 571 | S | 1 | 100.8 | 1.41 | 1.48 | 1.45 | 1.25 | 1.22 | 1.17 | 1.37 | 1.46 | 1.38 |
| A9Z1X7 | Serine/arginine repetitive matrix protein 1 | SRRM1 | 558 | S | 1 | 62.68 | 1.05 | 1.08 | NaN | NaN | 0.99 | 0.96 | NaN | NaN | NaN |
| A9Z1X7 | Serine/arginine repetitive matrix protein 1 | SRRM1 | 560 | S | 1 | 62.68 | 1.05 | 1.08 | NaN | NaN | 0.99 | 0.96 | NaN | NaN | NaN |
| A9Z1X7 | Serine/arginine repetitive matrix protein 1 | SRRM1 | 592 | S | 1 | 65.88 | 1.15 | 1.15 | 1.31 | 1.14 | 1.19 | 1.24 | 1.24 | 1.27 | 1.32 |
| A9Z1X7 | Serine/arginine repetitive matrix protein 1 | SRRM1 | 645 | S | 1 | 64.5 | 1.05 | 1.08 | 0.79 | 1.04 | NaN | NaN | 1.04 | 1.00 | 1.01 |
| A9Z1X7 | Serine/arginine repetitive matrix protein 1 | SRRM1 | 647 | S | 1 | 77.38 | 0.92 | 1.08 | 0.79 | 0.78 | NaN | 0.69 | 1.04 | 0.79 | 0.74 |
| A9Z1X7 | Serine/arginine repetitive matrix protein 1 | SRRM1 | 763 | S | 1 | 190.2 | 0.54 | 0.88 | 0.56 | 0.63 | 0.64 | 0.62 | 0.56 | 0.74 | 0.69 |
| A9Z1X7 | Serine/arginine repetitive matrix protein 1 | SRRM1 | 765 | S | 1 | 190.2 | 0.57 | 0.61 | 0.56 | 0.60 | 0.59 | 0.55 | 0.51 | 0.49 | 0.45 |
| A9Z1X7 | | SRRM1 | 547 | S | 0.997 | 50.83 | 2.00 | 2.08 | 2.29 | NaN | NaN | 1.60 | 1.61 | 1.54 | 1.76 |
| A9Z1X7 | Serine/arginine repetitive matrix protein 1 | SRRM1 | 606 | S | 1 | 101.5 | 1.49 | 1.56 | 1.81 | 1.46 | 0.98 | 1.45 | 1.63 | 1.02 | 1.71 |
| A9Z1X7 | Serine/arginine repetitive matrix protein 1 | SRRM1 | 614 | S | 0.919 | 79.89 | NaN | 1.07 | 1.05 | 0.99 | 0.97 | 0.97 | 1.05 | 1.02 | 1.02 |
| A9Z1X7 | Serine/arginine repetitive matrix protein 1 | SRRM1 | 616 | S | 1 | 79.89 | NaN | 1.07 | 1.05 | 0.99 | 0.97 | 0.97 | 1.05 | 1.02 | 1.02 |
| A9Z1X7 | Serine/arginine repetitive matrix protein 1 | SRRM1 | 424 | S | 0.998 | 79.8 | 1.52 | 1.58 | 1.59 | 1.37 | 1.35 | 1.37 | 1.47 | 1.48 | NaN |

| | | | | | | | | | | | | | | | |
|---|---|---|---|---|---|---|---|---|---|---|---|---|---|---|---|
| A9Z1X7 | Serine/arginine repetitive matrix protein 1 | SRRM1 | 426 | S | 1 | 79.8 | 0.69 | 0.71 | 0.74 | 0.66 | 0.69 | 0.68 | 0.71 | 0.74 | NaN |
| A9Z1X7 | Serine/arginine repetitive matrix protein 1 | SRRM1 | 625 | S | 1 | 129 | 0.90 | 0.95 | 0.96 | 1.36 | 1.00 | 0.91 | 1.24 | 0.91 | 0.94 |
| A9Z1X7 | Serine/arginine repetitive matrix protein 1 | SRRM1 | 409 | S | 0.996 | 81.43 | NaN | 1.28 | 1.08 | 0.95 | NaN | 0.97 | NaN | 1.14 | 1.12 |
| A9Z1X7 | Serine/arginine repetitive matrix protein 1 | SRRM1 | 375 | S | 0.993 | 78.15 | 1.08 | 1.06 | 1.07 | 1.00 | NaN | NaN | 1.02 | NaN | NaN |
| A9Z1X7 | Serine/arginine repetitive matrix protein 1 | SRRM1 | 260 | S | 1 | 108.3 | 0.90 | 0.90 | 0.95 | 0.93 | 0.95 | 0.96 | 0.96 | 0.99 | 0.98 |
| B7Z2E6 | 14-3-3 protein zeta/delta | YWHAZ | 87 | S | 0.994 | 134.5 | 0.84 | 0.72 | 0.74 | 0.73 | NaN | 0.73 | 1.23 | NaN | 0.97 |
| B0YIW6 | Coatomer subunit delta | ARCN1 | 534 | S | 1 | 84.89 | 2.00 | 2.12 | 1.72 | 2.37 | 2.39 | 2.34 | 0.80 | 0.90 | 1.06 |
| F5H7V1 | Nardilysin | NRD1 | 94 | S | 0.998 | 115.6 | 1.18 | 1.17 | 1.20 | 1.22 | 1.30 | NaN | 1.11 | 0.93 | 1.18 |
| F5H7V1 | Nardilysin | NRD1 | 96 | S | 0.565 | 82.93 | NaN | NaN | NaN | NaN | 1.25 | NaN | 0.99 | 1.09 | NaN |
| B1AKL4 | Eukaryotic translation initiation factor 4E transporter | EIF4ENIF 1 | 563 | S | 0.996 | 84.69 | 2.23 | 2.32 | NaN | NaN | NaN | NaN | 1.03 | 1.88 | 1.05 |
| B1AKL4 | Eukaryotic translation initiation factor 4E transporter | EIF4ENIF 1 | 927 | S | 0.921 | 44.57 | NaN | 1.97 | NaN | NaN | 1.61 | NaN | 1.16 | NaN | 1.31 |
| B1AKN3 | Arginine-glutamic acid dipeptide repeats protein | RERE | 838 | S | 1 | 126.6 | 1.36 | 1.00 | NaN | NaN | NaN | NaN | 0.88 | NaN | NaN |
| B1AKN3 | Arginine-glutamic acid dipeptide repeats protein | RERE | 845 | S | 0.992 | 126.6 | 1.36 | 1.00 | NaN | NaN | NaN | NaN | 0.88 | NaN | NaN |
| B1AKN3 | Arginine-glutamic acid dipeptide repeats protein | RERE | 847 | S | 0.992 | 126.6 | 1.36 | 1.00 | NaN | NaN | NaN | NaN | 0.88 | NaN | NaN |
| B1AKZ5 | Astrocytic phosphoprotein PEA-15 | PEA15 | 94 | S | 1 | 172.7 | 1.42 | 1.41 | 1.39 | 1.23 | 1.27 | 1.30 | 1.37 | 1.41 | 1.42 |
| E5RHT1 | Presenilin;Presenilin-2;Presenilin-2 NTF subunit;Presenilin-2 CTF subunit | PSEN2 | 25 | S | 0.998 | 181.9 | 0.51 | 0.50 | NaN | NaN | NaN | NaN | 0.49 | NaN | NaN |
| B1AUU8 | Epidermal growth factor receptor substrate 15 | EPS15 | 662 | S | 1 | 78.66 | 1.45 | 1.59 | 1.73 | 1.43 | 1.32 | NaN | 1.34 | 1.53 | 1.81 |
| B1AUU8 | Epidermal growth factor receptor substrate 15 | EPS15 | 680 | S | 0.998 | 45.55 | 0.73 | 1.80 | NaN | NaN | NaN | NaN | NaN | NaN | NaN |
| B1AUU8 | Epidermal growth factor receptor substrate 15 | EPS15 | 428 | S | 0.5 | 111.2 | NaN | 0.97 | NaN | NaN | NaN | NaN | 0.93 | NaN | 0.86 |
| B1AUU8 | Epidermal growth factor receptor substrate 15 | EPS15 | 429 | S | 0.5 | 111.2 | NaN | 0.97 | NaN | NaN | NaN | NaN | 0.93 | NaN | 0.86 |
| H0YCM0 | Non-specific serine/threonine protein kinase;Serine/threonine- | PAK1 | 65 | S | 1 | 166.5 | 0.96 | 1.09 | NaN | 0.94 | 1.00 | 0.86 | NaN | 1.07 | 0.97 |
| B3KR00 | AMSH-like protease | STAMBPL 1 | 76 | S | 0.983 | 71.39 | NaN | NaN | 1.05 | 0.99 | 0.66 | 0.93 | NaN | NaN | NaN |
| H3BN34 | Pyruvate kinase;Pyruvate kinase PKM | PKM | 37 | S | 1 | 151.5 | NaN | 1.36 | 1.35 | 1.35 | NaN | 1.24 | 1.14 | 1.20 | 1.13 |
| C9JUN4 | Phosphoribosyl pyrophosphate synthase- | PRPSAP1 | 141 | S | 1 | 226.1 | 0.57 | 0.58 | 0.66 | 0.65 | 0.75 | 0.72 | NaN | 0.50 | NaN |

| | | | | | | | | | | | | | | |
|---|---|---|---|---|---|---|---|---|---|---|---|---|---|---|
| C9JEN3 | Protein lifeguard 3 | TMBIM1 | 81 | S | 0.999 | 85.52 | NaN | 1.56 | 1.01 | NaN | NaN | NaN | NaN | 1.90 | NaN |
| I3L0Y9 | Brain-specific angiogenesis inhibitor 1-associated protein 2 | BAIAP2 | 82 | S | 1 | 89.3 | NaN | NaN | 1.36 | 1.23 | 1.26 | 1.28 | 0.84 | 0.90 | 0.89 |
| B4DWJ1 | Focal adhesion kinase 1 | PTK2 | 220 | S | 0.999 | 79.51 | 2.73 | 2.23 | 2.53 | 1.97 | 2.06 | 2.23 | 1.02 | 1.23 | 1.32 |
| G3V2D6 | Heterogeneous nuclear ribonucleoproteins C1/C2 | HNRNPC | 220;141 | S | 1 | 144.3 | 0.80 | 0.80 | 0.86 | 0.84 | 0.83 | 0.82 | 0.79 | 0.81 | 0.83 |
| B4DYP1 | N(G),N(G)-dimethylarginine dimethylaminohydrolase 1 | DDAH1 | 105 | S | 1 | 44.3 | NaN | NaN | 1.77 | 1.01 | NaN | 1.11 | NaN | 0.70 | NaN |
| B4E1E0 | CTP synthase;CTP synthase | CTPS1 | 415 | S | 0.571 | 46.5 | NaN | NaN | 1.62 | NaN | NaN | 0.99 | 2.21 | 1.88 | NaN |
| B4E1E0 | CTP synthase;CTP synthase | CTPS1 | 418 | S | 0.983 | 169.8 | 0.71 | 0.62 | NaN | 0.53 | 0.51 | 0.46 | 1.23 | 1.47 | 0.88 |
| B4E1E0 | CTP synthase;CTP synthase | CTPS1 | 419 | S | 1 | 169.8 | 1.19 | 1.06 | 1.10 | 0.66 | 0.72 | 0.58 | 0.96 | 1.22 | 0.94 |
| H7C118 | Protein phosphatase 1 regulatory subunit 7 | PPP1R7 | 33 | S | 1 | 95.62 | 0.51 | 0.55 | 0.59 | 0.71 | 0.58 | 1.08 | 0.86 | 0.76 | 0.79 |
| H7C118 | Protein phosphatase 1 regulatory subunit 7 | PPP1R7 | 36 | S | 1 | 95.62 | 0.59 | 1.05 | 0.61 | 1.12 | 0.62 | 1.08 | 1.24 | 0.86 | 0.97 |
| H7C2Y0 | Septin-2 | 2-Sep | 73 | S | 1 | 288.9 | 0.95 | 0.93 | 0.94 | 0.88 | 0.87 | 0.94 | 0.97 | 0.99 | 1.07 |
| B5MDQ0 | DNA excision repair protein ERCC-6-like | ERCC6L | 823 | S | 0.833 | 117.5 | 1.11 | NaN | 1.12 | NaN | NaN | NaN | NaN | 1.42 | NaN |
| B5MDQ0 | DNA excision repair protein ERCC-6-like | ERCC6L | 946 | S | 1 | 73.5 | 0.76 | 0.77 | NaN | 0.97 | 0.90 | 0.95 | 1.03 | 1.04 | NaN |
| H3BPE4 | Eukaryotic translation initiation factor 3 subunit C;Eukaryotic translation initiation factor 3 subunit C-like protein | EIF3C | 13 | S | 0.705 | 85.24 | NaN | NaN | NaN | NaN | NaN | NaN | 0.42 | NaN | 0.49 |
| H3BPE4 | Eukaryotic translation initiation factor 3 subunit C;Eukaryotic translation initiation factor 3 subunit C-like protein | EIF3C | 15 | S | 0.722 | 85.24 | NaN | NaN | NaN | NaN | NaN | NaN | 0.42 | NaN | 0.49 |
| H3BPE4 | Eukaryotic translation initiation factor 3 subunit C;Eukaryotic translation initiation factor 3 subunit C-like protein | EIF3C | 16 | S | 0.495 | 83.19 | NaN | NaN | NaN | NaN | NaN | NaN | 0.42 | NaN | 0.49 |
| H3BPE4 | Eukaryotic translation initiation factor 3 subunit C;Eukaryotic translation initiation factor 3 subunit C-like protein | EIF3C | 18 | S | 0.894 | 85.24 | NaN | NaN | NaN | NaN | NaN | NaN | 0.42 | NaN | 0.49 |
| H3BPE4 | Eukaryotic translation initiation factor 3 subunit C;Eukaryotic translation initiation factor 3 subunit C-like protein | EIF3C | 39 | S | 1 | 168 | 1.62 | 1.68 | 1.61 | 1.33 | 1.37 | 1.47 | 1.36 | 1.33 | 1.43 |
| B7Z4K4 | Putative tRNA (cytidine(32)/guanosine(34)-2'-O)-methyltransferase | FTSJ1 | 134 | S | 0.983 | 62.61 | 1.16 | 1.38 | NaN | NaN | NaN | NaN | NaN | NaN | NaN |
| B7Z7F3 | Ran-binding protein 3 | RANBP3 | 32 | S | 1 | 171.2 | 0.77 | 0.80 | 0.73 | 0.77 | NaN | 0.81 | 0.64 | 0.68 | 0.63 |
| B7Z7F3 | Ran-binding protein 3 | RANBP3 | 33 | S | 1 | 171.9 | 0.66 | 0.60 | 1.34 | 0.77 | 0.68 | 0.83 | 0.61 | 0.68 | 0.61 |
| B7Z7F3 | Ran-binding protein 3 | RANBP3 | 40 | S | 1 | 134.6 | NaN | 0.27 | 1.00 | NaN | 0.66 | 0.85 | NaN | 1.38 | 1.19 |
| B7Z7F3 | Ran-binding protein 3 | RANBP3 | 260 | S | 1 | 160.3 | 1.02 | 1.09 | 1.06 | 1.13 | 1.20 | 1.10 | 1.02 | 1.01 | 1.04 |

| | | | | | | | | | | | | | | | |
|---|---|---|---|---|---|---|---|---|---|---|---|---|---|---|---|
| F8WF16 | Periphilin-1 | PPHLN1 | 138 | S | 0.996 | 110.6 | 0.75 | 0.76 | 0.75 | 0.72 | 0.79 | 0.74 | 0.82 | 0.82 | 0.83 |
| B7ZBM3 | Forkhead box protein P4 | FOXP4 | 542 | S | 0.873 | 46.84 | NaN | NaN | NaN | NaN | NaN | 1.20 | 0.67 | NaN | 0.65 |
| B7ZKW8 | CapZ-interacting protein | RCSD1 | 90;120 | S | 1 | 153.1 | 0.36 | 0.37 | NaN | NaN | 0.34 | NaN | NaN | NaN | 0.34 |
| B7ZKW8 | CapZ-interacting protein | RCSD1 | 186;216 | S | 0.999 | 128.4 | 0.56 | 0.58 | 0.55 | 0.71 | 0.61 | 0.57 | 0.57 | 0.63 | 0.60 |
| B7ZKW8 | CapZ-interacting protein | RCSD1 | 321;351 | S | 1 | 97.78 | 0.49 | 0.52 | 0.58 | 0.68 | 0.72 | 0.70 | 0.73 | 0.68 | 0.69 |
| B7ZKW8 | CapZ-interacting protein | RCSD1 | 237;267 | S | 0.619 | 223.3 | 0.47 | NaN | NaN | 0.79 | 1.48 | NaN | NaN | NaN | NaN |
| B7ZKW8 | CapZ-interacting protein | RCSD1 | 238;268 | S | 0.834 | 190 | 0.47 | NaN | NaN | 0.72 | 1.48 | NaN | NaN | NaN | NaN |
| B7ZKW8 | CapZ-interacting protein | RCSD1 | 53;83 | S | 0.796 | 75.74 | 0.58 | 0.55 | NaN | NaN | NaN | NaN | NaN | NaN | NaN |
| E5RJM0 | Cytoplasmic polyadenylation element-binding protein 4 | CPEB4 | 97 | S | 0.701 | 44.75 | 2.11 | NaN | 0.82 | NaN | NaN | 1.77 | NaN | NaN | NaN |
| B8ZZ08 | Putative RNA-binding protein Luc7-like 1 | LUC7L | 162 | S | 1 | 72.32 | NaN | NaN | 1.23 | 1.04 | NaN | NaN | 1.16 | 1.10 | NaN |
| B8ZZF5 | Protein eva-1 homolog A | EVA1A | 102 | S | 0.939 | 90.86 | NaN | NaN | 1.06 | NaN | NaN | NaN | 1.73 | NaN | 1.97 |
| B8ZZI9 | Aftiphilin | AFTPH | 37 | S | 0.997 | 70.96 | 1.16 | NaN | 1.43 | NaN | NaN | NaN | 1.22 | 1.23 | 1.13 |
| C9J2I0 | Arf-GAP domain and FG repeat-containing protein 1 | AGFG1 | 103 | S | 0.995 | 65.63 | 2.93 | 1.78 | NaN | 1.64 | 1.71 | 1.30 | 1.87 | 1.75 | NaN |
| B9ZVT1 | RNA-binding protein 12B | RBM12B | 575 | S | 1 | 53.45 | 0.69 | 0.59 | NaN | 0.72 | 0.73 | NaN | NaN | 0.87 | NaN |
| B9ZVT1 | RNA-binding protein 12B | RBM12B | 719 | S | 0.998 | 42.94 | NaN | NaN | NaN | NaN | NaN | NaN | 0.99 | 1.00 | 0.98 |
| C9JWF0 | Structural maintenance of chromosomes protein 4;Structural maintenance of chromosomes protein | SMC4 | 22 | S | 1 | 190.4 | 1.20 | 1.10 | 0.84 | 0.96 | 0.83 | 0.84 | 1.17 | 1.18 | 0.90 |
| C9JWF0 | Structural maintenance of chromosomes protein 4;Structural maintenance of chromosomes protein | SMC4 | 27 | S | 0.914 | 190.4 | 0.86 | NaN | NaN | 1.11 | 1.20 | 0.96 | 0.68 | NaN | 0.82 |
| C9JWF0 | Structural maintenance of chromosomes protein 4;Structural maintenance of chromosomes protein | SMC4 | 28 | S | 0.841 | 159.5 | NaN | 1.10 | 1.03 | NaN | NaN | 0.97 | NaN | NaN | NaN |
| C9JWF0 | Structural maintenance of chromosomes protein 4 | SMC4 | 41 | S | 0.982 | 220.5 | NaN | 1.10 | NaN | 0.97 | NaN | 0.97 | NaN | 1.27 | NaN |
| C9JIY9 | Nucleoporin NUP53 | NUP35 | 49 | S | 0.727 | 55.01 | 0.88 | 0.89 | NaN | 1.61 | 1.49 | NaN | NaN | NaN | NaN |
| H7C559 | Serine/threonine-protein phosphatase 4 regulatory subunit 2 | PPP4R2 | 58 | S | 0.98 | 75.53 | 0.65 | 0.70 | 1.00 | 1.23 | 1.67 | 0.99 | 0.59 | 0.66 | NaN |
| C9J3R1 | Zinc finger protein 639 | ZNF639 | 60 | S | 0.995 | 96.53 | NaN | NaN | 0.98 | 1.39 | 1.25 | 0.95 | NaN | 1.01 | NaN |
| C9J0A7 | Charged multivesicular body protein 2b | CHMP2B | 169 | S | 1 | 135 | 1.05 | 1.13 | 1.12 | 1.00 | 1.11 | 1.04 | 1.17 | 1.01 | 1.12 |
| C9J0I9 | Nuclear-interacting partner of ALK | ZC3HC1 | 24 | S | 0.999 | 127.4 | 0.62 | 0.75 | NaN | 1.28 | NaN | NaN | 0.91 | 0.74 | NaN |
| C9J0I9 | Nuclear-interacting partner of ALK | ZC3HC1 | 351 | S | 0.5 | 162.4 | NaN | 0.98 | NaN | 1.62 | 1.64 | NaN | NaN | 0.84 | NaN |
| C9J0I9 | Nuclear-interacting partner of ALK | ZC3HC1 | 352 | S | 0.932 | 162.8 | 1.01 | 1.03 | 0.87 | 1.32 | 1.34 | 1.12 | 0.76 | 0.84 | 0.78 |
| C9J0I9 | Nuclear-interacting partner of ALK | ZC3HC1 | 292 | S | 0.98 | 77.73 | 1.26 | 0.94 | NaN | NaN | 1.17 | NaN | NaN | NaN | NaN |

| | | | | | | | | | | | | | | | |
|---|---|---|---|---|---|---|---|---|---|---|---|---|---|---|---|
| C9J0I9 | Nuclear-interacting partner of ALK | ZC3HC1 | 295 | S | 0.591 | 97.35 | 1.26 | 1.28 | NaN | NaN | 1.17 | NaN | NaN | NaN | NaN |
| C9J0I9 | Nuclear-interacting partner of ALK | ZC3HC1 | 301 | S | 0.98 | 153.9 | 1.56 | NaN | NaN | 1.41 | 1.32 | NaN | 1.25 | 1.18 | NaN |
| C9J0I9 | Nuclear-interacting partner of ALK | ZC3HC1 | 316 | S | 0.771 | 89.98 | 1.04 | 1.31 | NaN | 0.94 | NaN | 1.08 | NaN | NaN | NaN |
| C9J1H7 | Nucleolin | NCL | 51 | S | 0.998 | 114.9 | 0.87 | 0.89 | 0.85 | 1.26 | 1.29 | 1.28 | 0.57 | 0.55 | 0.54 |
| F5H5B7 | Cleavage and polyadenylation specificity factor subunit 7 | CPSF7 | 60 | S | 1 | 56.95 | 1.13 | 1.19 | NaN | NaN | 1.26 | NaN | 0.87 | NaN | NaN |
| C9J3F6 | TBC1 domain family member | TBC1D5 | 528 | S | 0.981 | 77.66 | 0.96 | 1.01 | 1.12 | NaN | 1.12 | 1.02 | 0.87 | 1.02 | 0.81 |
| M0R1Q8 | Regulator of chromosome condensation | RCC1 | 113 | S | 1 | 122.7 | 0.79 | 0.84 | 0.82 | 0.87 | 0.87 | 0.89 | 0.89 | 0.92 | 0.95 |
| C9J4C4 | Folliculin | FLCN | 9 | S | 0.998 | 90.26 | 0.85 | 0.76 | 0.80 | 0.73 | 0.61 | 0.70 | 0.84 | 0.88 | 0.68 |
| C9J4K0 | Ashwin | C2orf49 | 151 | S | 0.95 | 51.52 | NaN | NaN | 2.49 | NaN | 2.66 | 2.43 | 0.89 | 1.00 | 1.24 |
| C9J542 | PDZ and LIM domain protein 4 | PDLIM4 | 53 | S | 0.98 | 183.8 | 2.29 | 2.41 | 2.24 | 0.97 | 1.06 | 0.93 | 2.76 | 2.83 | 2.87 |
| E9PNJ7 | Nucleosome assembly protein 1-like 4 | NAP1L4 | 125 | S | 1 | 457.2 | 0.87 | 0.88 | 0.89 | 0.94 | 0.96 | 0.98 | 0.75 | 0.77 | 0.81 |
| C9JM54 | Chromobox protein homolog 8 | CBX8 | 165 | S | 0.702 | 72.8 | 0.46 | 0.45 | 0.56 | 0.59 | 0.88 | 0.78 | NaN | 0.56 | 0.57 |
| C9JM54 | Chromobox protein homolog 8 | CBX8 | 166 | S | 0.565 | 49.17 | NaN | NaN | NaN | NaN | NaN | NaN | 0.54 | NaN | 0.31 |
| C9J6P4 | Zinc finger CCCH-type antiviral protein 1 | ZC3HAV1 | 284 | S | 1 | 113.3 | 1.44 | 1.23 | NaN | 1.39 | NaN | NaN | 0.97 | NaN | 1.16 |
| C9J6P4 | Zinc finger CCCH-type antiviral protein 1 | ZC3HAV1 | 275 | S | 1 | 94.02 | NaN | 1.15 | NaN | 1.16 | NaN | 0.80 | NaN | NaN | NaN |
| C9J813 | | CALD1 | 202 | S | 1 | 104.3 | 0.99 | 1.01 | 1.02 | 1.12 | NaN | 1.09 | 1.34 | 1.20 | 1.42 |
| C9J9H5 | Mitotic spindle assembly checkpoint protein MAD1 | MAD1L1 | 18 | S | 0.668 | 53.21 | 0.75 | 0.65 | NaN | NaN | NaN | NaN | NaN | NaN | NaN |
| C9J9W2 | LIM and SH3 domain protein 1 | LASP1 | 110 | S | 1 | 190.2 | 1.48 | 1.18 | 1.17 | 2.29 | 2.25 | 2.30 | NaN | 0.65 | 1.68 |
| C9JA69 | HMG box transcription factor BBX | BBX | 794 | S | 0.996 | 84.94 | 0.57 | 0.46 | 0.55 | 0.54 | 0.69 | 0.58 | 1.14 | 1.18 | NaN |
| C9JA93 | | TBC1D15 | 106 | S | 0.973 | 168.6 | 0.97 | 1.00 | 0.86 | 0.81 | 0.87 | 0.66 | 1.02 | NaN | 1.02 |
| C9JAE6 | Multidrug and toxin extrusion protein 2 | SLC47A2 | 338 | S | 0.885 | 45.86 | NaN | NaN | NaN | 0.80 | 0.74 | NaN | NaN | 0.53 | NaN |
| C9JAE6 | Multidrug and toxin extrusion protein 2 | SLC47A2 | 343 | S | 0.824 | 45.86 | NaN | NaN | NaN | 0.80 | 0.74 | NaN | NaN | 0.53 | NaN |
| C9JL19 | 60 kDa heat shock protein, mitochondrial | HSPD1 | 70 | S | 0.985 | 150.1 | 2.79 | 2.85 | 2.26 | 1.77 | NaN | NaN | NaN | 1.73 | NaN |
| C9JED2 | SH3 domain-binding protein 4 | SH3BP4 | 131 | S | 1 | 102.6 | 1.15 | 1.15 | 1.16 | 1.72 | 1.57 | 1.49 | 0.55 | 0.58 | 0.78 |
| C9JEZ4 | Cdc42 effector protein 3 | CDC42EP3 | 100 | S | 0.999 | 298.8 | 5.12 | 5.23 | 6.80 | 5.84 | NaN | 5.74 | 1.91 | 1.45 | 1.99 |
| E7EV54 | Proline-, glutamic acid- and leucine-rich protein 1 | PELP1 | 819 | S | 1 | 86.51 | 0.81 | NaN | NaN | NaN | NaN | NaN | 0.92 | NaN | 1.11 |
| E7EV54 | Proline-, glutamic acid- and leucine-rich protein 1 | PELP1 | 334 | S | 1 | 156.1 | 0.82 | 0.82 | 0.70 | 0.88 | 0.94 | 0.87 | 0.85 | 0.95 | 0.86 |
| C9JHG2 | Raftlin | RFTN1 | 220 | S | 0.999 | 60.47 | 2.93 | 2.79 | 3.55 | NaN | NaN | 1.74 | NaN | 1.34 | NaN |
| C9JI87 | Voltage-dependent anion-selective channel protein 1 | VDAC1 | 104 | S | 0.997 | 138.7 | 0.85 | 0.81 | 0.95 | 1.08 | 1.08 | 1.04 | 1.07 | NaN | 1.07 |

| | | | | | | | | | | | | | | | |
|---|---|---|---|---|---|---|---|---|---|---|---|---|---|---|---|
| C9JIG9 | Serine/threonine-protein kinase OSR1 | OXSR1 | 339 | S | 1 | 308.4 | 1.13 | NaN | 1.06 | NaN | 2.39 | 1.00 | 0.79 | NaN | 0.94 |
| C9JIG9 | Serine/threonine-protein kinase OSR1 | OXSR1 | 427 | S | 0.853 | 154.3 | NaN | NaN | NaN | 1.62 | 1.78 | 1.53 | NaN | NaN | NaN |
| C9JKH7 | Fibronectin type III domain-containing protein 3B | FNDC3B | 181 | S | 0.995 | 84.82 | 2.04 | 1.84 | 1.97 | 1.14 | 1.06 | 1.00 | 1.87 | 1.96 | 1.98 |
| C9JKL2 | MLN64 N-terminal domain homolog | STARD3NL | 192 | S | 0.973 | 180.2 | 0.72 | 0.60 | 0.64 | 0.66 | NaN | 0.63 | 0.81 | NaN | 0.96 |
| C9JKL2 | MLN64 N-terminal domain homolog | STARD3NL | 200 | S | 1 | 180.2 | 0.64 | 0.60 | 0.64 | 0.75 | NaN | 0.56 | 0.81 | NaN | 0.72 |
| I3L3A7 | Protein FAM195B | FAM195B | 21 | S | 0.993 | 98.93 | 0.99 | 0.98 | 1.16 | 1.03 | 1.01 | NaN | 1.25 | NaN | 1.14 |
| C9JN71 | Zinc finger protein 878 | ZNF878 | 209 | S | 0.92 | 62.24 | NaN | NaN | 0.55 | 1.02 | 0.67 | 0.53 | 0.62 | 0.90 | 0.67 |
| C9JNP9 | Ubiquitin fusion degradation protein 1 homolog | UFD1L | 151 | S | 0.804 | 67.58 | 1.70 | 1.51 | 1.47 | 2.53 | 2.73 | 2.65 | 0.93 | NaN | 1.37 |
| F8WBS8 | 26S proteasome non-ATPase regulatory subunit 2 | PSMD2 | 16 | S | 1 | 174.9 | 1.21 | 1.28 | 1.18 | 1.65 | 1.73 | 1.81 | 0.96 | 0.96 | 0.99 |
| C9JRJ5 | LIM domain-containing protein | LIMD1 | 384 | S | 0.982 | 90.69 | NaN | 0.85 | NaN | 0.91 | 1.03 | NaN | NaN | 0.67 | NaN |
| C9JRM9 | Zinc finger protein with KRAB and SCAN domains 1 | ZKSCAN1 | 13 | S | 1 | 82.53 | NaN | NaN | NaN | NaN | NaN | 0.43 | NaN | 0.61 | 1.03 |
| C9JWC4 | Holliday junction recognition protein | HJURP | 388 | S | 1 | 81.59 | 1.39 | 1.16 | NaN | NaN | NaN | NaN | NaN | NaN | NaN |
| C9JZG1 | Eukaryotic translation initiation factor 3 subunit B | EIF3B | 115 | S | 1 | 287.9 | 1.07 | 1.09 | 1.09 | 1.06 | 1.00 | 0.97 | 1.06 | 1.05 | 1.08 |
| C9JZG1 | Eukaryotic translation initiation factor 3 subunit B | EIF3B | 125 | S | 1 | 287.9 | 1.09 | 1.09 | 1.09 | 1.01 | 0.91 | 0.97 | 1.15 | 1.16 | 1.15 |
| C9JZW3 | Elongation factor 1-beta | EEF1B2 | 106 | S | 1 | 478.2 | 1.13 | 1.07 | 1.09 | 1.05 | 0.92 | 1.08 | NaN | 1.06 | 1.29 |
| C9K0J5 | Ras-associated and pleckstrin homology domains-containing protein 1 | RAPH1 | 1206 | S | 0.736 | 98.99 | 1.61 | NaN | 1.67 | 2.12 | 1.83 | 1.85 | NaN | 0.98 | NaN |
| C9K0J5 | Ras-associated and pleckstrin homology domains-containing protein 1 | RAPH1 | 662 | S | 1 | 56.95 | 2.73 | 2.01 | NaN | 2.71 | NaN | NaN | NaN | NaN | NaN |
| C9K0K7 | Phosphoribosyl pyrophosphate synthase- | PRPSAP2 | 227 | S | 0.999 | 183.9 | NaN | 0.67 | 0.89 | 0.74 | 0.79 | NaN | 0.70 | 0.78 | NaN |
| D6R9B6 | 40S ribosomal protein S3a | RPS3A | 144 | S | 1 | 66.49 | NaN | 1.47 | 1.49 | 1.45 | 1.75 | 1.60 | 1.02 | 0.99 | 0.93 |
| D6REC6 | Cyclin-dependent kinase 7 | CDK7 | 71 | S | 1 | 86.87 | 0.54 | NaN | 0.49 | 0.56 | 0.56 | 0.55 | NaN | 0.90 | 0.83 |
| D6R9K7 | RNA-binding protein 4 | RBM4 | 86 | S | 0.963 | 100.2 | NaN | NaN | 1.66 | 1.58 | 1.69 | NaN | NaN | NaN | NaN |
| D6RAF8 | Heterogeneous nuclear ribonucleoprotein D0 | HNRNPD | 80 | S | 1 | 100.8 | 0.71 | 0.79 | 0.72 | 0.76 | NaN | NaN | NaN | NaN | NaN |
| D6RAF8 | Heterogeneous nuclear ribonucleoprotein D0 | HNRNPD | 82 | S | 0.792 | 319.9 | NaN | 0.82 | 0.81 | 0.76 | 0.90 | NaN | NaN | NaN | NaN |
| D6RAF8 | Heterogeneous nuclear ribonucleoprotein D0 | HNRNPD | 83 | S | 0.996 | 362.1 | 0.76 | 0.79 | 0.76 | 0.88 | 0.91 | 0.87 | 0.91 | 0.91 | 0.93 |
| D6RAY8 | SWI/SNF-related matrix-associated actin-dependent regulator of chromatin subfamily A containing | SMARCAD1 | 211 | S | 0.989 | 87.74 | 1.09 | 1.19 | NaN | 1.92 | 1.24 | NaN | NaN | 3.22 | NaN |

| | | | | | | | | | | | | | | | |
|---|---|---|---|---|---|---|---|---|---|---|---|---|---|---|---|
| D6RAY8 | SWI/SNF-related matrix-associated actin-dependent regulator of chromatin subfamily A containing | SMARCAD1 | 212 | S | 0.989 | 87.74 | 1.09 | 1.19 | NaN | 1.92 | 1.24 | NaN | NaN | 3.22 | NaN |
| D6RAY8 | SWI/SNF-related matrix-associated actin-dependent regulator of chromatin subfamily A containing | SMARCAD1 | 214 | S | 0.826 | 87.74 | 1.09 | 1.19 | NaN | 1.92 | 1.24 | NaN | NaN | 3.22 | NaN |
| D6RB55 | Amyloid beta A4 precursor protein-binding family B member 2 | APBB2 | 106 | S | 0.985 | 43.52 | NaN | 0.90 | NaN | 1.03 | 1.18 | NaN | NaN | NaN | NaN |
| H7BXG7 | Protein transport protein Sec31A | SEC31A | 532 | S | 1 | 153.9 | 1.95 | 1.84 | 2.06 | 1.59 | 1.48 | 1.60 | 1.98 | 2.09 | 1.76 |
| H7BXG7 | Protein transport protein Sec31A | SEC31A | 912 | S | 0.998 | 161 | 1.47 | 1.38 | 1.24 | 1.50 | 1.29 | 1.51 | 1.11 | 1.01 | NaN |
| E3W994 | CLIP-associating protein 2 | CLASP2 | 575 | S | 0.984 | 146.7 | 2.35 | 1.68 | NaN | NaN | NaN | NaN | 2.41 | 1.45 | NaN |
| E5RHJ2 | Aspartyl/asparaginyl beta-hydroxylase | ASPH | 29 | S | 0.827 | 104.8 | 0.87 | NaN | 1.04 | NaN | NaN | 1.21 | 0.70 | NaN | 0.68 |
| E5RGC1 | F-box/WD repeat-containing protein 11 | FBXW11 | 5 | S | 0.995 | 46.62 | NaN | NaN | 0.69 | 0.78 | 0.74 | NaN | 1.00 | 0.87 | NaN |
| E5RGC1 | F-box/WD repeat-containing protein 11 | FBXW11 | 17 | S | 0.991 | 46.62 | NaN | NaN | 0.69 | 0.78 | 0.74 | NaN | 1.00 | 0.87 | NaN |
| E5RHA3 | Glutamate-rich protein 1 | ERICH1 | 144 | S | 1 | 137.2 | 0.71 | 0.84 | 0.75 | 0.82 | 1.15 | NaN | NaN | NaN | 1.21 |
| E5RJU9 | Protein LYRIC | MTDH | 370 | S | 1 | 234.8 | 0.70 | 0.88 | 7.83 | 0.81 | 0.78 | 0.85 | 0.99 | 1.00 | 1.01 |
| E7EMD6 | A-kinase anchor protein 10, mitochondrial | AKAP10 | 187 | S | 0.981 | 134 | 0.97 | 0.89 | 1.12 | 1.11 | 0.97 | 1.14 | 1.02 | NaN | NaN |
| E7EMN6 | Protein phosphatase inhibitor 2-like protein 3;Protein phosphatase inhibitor 2 | PPP1R2 | 95 | S | 1 | 235.1 | 0.85 | 0.89 | 0.54 | 0.60 | 0.52 | NaN | 1.03 | 0.77 | 1.08 |
| E7EMN6 | Protein phosphatase inhibitor 2-like protein 3;Protein phosphatase inhibitor 2 | PPP1R2 | 96 | S | 1 | 235.1 | 0.85 | 0.62 | 0.54 | NaN | NaN | NaN | 1.10 | 1.06 | 1.08 |
| E7EMW7 | E3 ubiquitin-protein ligase UBR5 | UBR5 | 446 | S | 0.992 | 51.82 | 0.59 | NaN | NaN | 1.00 | 1.06 | NaN | NaN | NaN | NaN |
| E7EMW7 | E3 ubiquitin-protein ligase UBR5 | UBR5 | 447 | S | 0.999 | 51.82 | 0.59 | NaN | NaN | 1.00 | 1.06 | NaN | NaN | NaN | NaN |
| E7EMW7 | E3 ubiquitin-protein ligase UBR5 | UBR5 | 450 | S | 1 | 51.82 | 0.59 | NaN | NaN | 1.00 | 1.06 | NaN | NaN | NaN | NaN |
| E7EMZ9 | Transforming acidic coiled-coil-containing protein 2 | TACC2 | 2467 | S | 0.967 | 128.1 | 0.86 | NaN | 1.14 | 0.94 | NaN | 1.07 | 1.33 | NaN | 1.50 |
| E7EMZ9 | Transforming acidic coiled-coil-containing protein 2 | TACC2 | 2272 | S | 0.994 | 76.51 | 1.45 | 1.48 | 1.13 | 1.35 | 1.44 | 1.37 | 1.25 | 0.40 | 1.26 |
| E7EMZ9 | Transforming acidic coiled-coil-containing protein 2 | TACC2 | 2276 | S | 1 | 77.07 | 1.45 | 1.48 | 1.20 | 1.35 | 1.44 | 1.30 | 1.25 | 1.33 | 1.26 |
| E7EMZ9 | Transforming acidic coiled-coil-containing protein 2 | TACC2 | 2524 | S | 0.748 | 190.7 | 0.98 | 0.96 | NaN | NaN | NaN | 1.18 | 0.86 | NaN | NaN |
| E7EN95 | Filamin-B | FLNB | 1914 | S | 1 | 113.5 | 3.26 | 2.66 | 2.77 | 3.36 | NaN | 3.83 | 1.03 | 0.86 | 0.83 |

| | | | | | | | | | | | | | | | |
|---|---|---|---|---|---|---|---|---|---|---|---|---|---|---|---|
| E7EN95 | Filamin-B | FLNB | 814 | S | 0.999 | 163.5 | 9.87 | 8.62 | 10.61 | 16.86 | 16.68 | 15.30 | 1.01 | 0.80 | 0.98 |
| E7EN95 | Filamin-B | FLNB | 2285 | S | 1 | 204.5 | 0.70 | 0.73 | 0.75 | 0.98 | 1.01 | 1.00 | 0.74 | 0.76 | 0.76 |
| E7EN95 | Filamin-B | FLNB | 2288 | S | 0.571 | 63.18 | 0.70 | 0.76 | 0.88 | 1.09 | NaN | 1.08 | NaN | NaN | NaN |
| E7EN95 | Filamin-B | FLNB | 1336 | S | 1 | 130.3 | 3.02 | 3.10 | 2.59 | 3.60 | 3.92 | 3.46 | 1.20 | 1.36 | 1.13 |
| F5H3K2 | Uncharacterized protein C12orf43 | C12orf43 | 134 | S | 0.926 | 98.19 | 1.23 | 1.27 | NaN | 1.29 | NaN | 0.85 | 1.19 | 1.44 | NaN |
| F5H3K2 | Uncharacterized protein C12orf43 | C12orf43 | 92 | S | 1 | 41.6 | NaN | NaN | NaN | 0.59 | NaN | NaN | 0.89 | 0.72 | NaN |
| E7EV99 | Alpha-adducin | ADD1 | 12 | S | 0.98 | 90.85 | NaN | 2.66 | NaN | 3.03 | 2.74 | NaN | NaN | 0.76 | NaN |
| E7EV99 | Alpha-adducin | ADD1 | 358 | S | 1 | 244 | 0.87 | 1.00 | 0.84 | 0.77 | 0.83 | 0.86 | 0.97 | 0.97 | 0.91 |
| E7EPJ7 | Triple functional domain | TRIO | 2188 | S | 0.999 | 59.47 | 0.79 | 0.88 | NaN | NaN | 0.78 | 1.78 | 1.15 | 0.99 | 1.40 |
| E7EPJ7 | Triple functional domain | TRIO | 2192 | S | 0.987 | 42.92 | 1.87 | 1.51 | NaN | NaN | 2.11 | 1.64 | 2.58 | 2.31 | 1.29 |
| E7EPJ7 | Triple functional domain | TRIO | 2162 | S | 0.774 | 48.9 | NaN | 0.69 | 0.84 | NaN | NaN | NaN | 1.07 | 1.18 | 0.91 |
| E7EPK0 | LIM and calponin homology domains-containing protein 1 | LIMCH1 | 511 | S | 0.999 | 164 | NaN | 0.64 | NaN | 1.03 | NaN | 0.81 | 0.71 | 0.82 | 0.88 |
| E7EPK0 | LIM and calponin homology domains-containing protein 1 | LIMCH1 | 522 | S | 0.728 | 64.38 | NaN | NaN | NaN | NaN | NaN | NaN | 1.08 | 1.16 | NaN |
| E7EPK0 | LIM and calponin homology domains-containing protein 1 | LIMCH1 | 84 | S | 0.956 | 85.81 | 0.80 | 0.70 | 0.72 | 0.85 | 0.84 | 0.82 | NaN | NaN | NaN |
| E7EPK0 | LIM and calponin homology domains-containing protein 1 | LIMCH1 | 70 | S | 1 | 80.51 | 0.70 | 0.86 | 1.03 | NaN | 1.00 | 0.87 | NaN | 1.03 | 1.01 |
| E7EPK0 | LIM and calponin homology domains-containing protein 1 | LIMCH1 | 813 | S | 1 | 162 | 1.48 | 1.44 | 1.45 | 1.35 | 1.36 | 1.31 | 2.24 | 2.16 | NaN |
| E7EPK0 | LIM and calponin homology domains-containing protein 1 | LIMCH1 | 559 | S | 1 | 189.1 | 1.08 | 1.09 | 1.04 | 1.06 | 1.10 | 1.15 | 1.10 | 1.17 | 0.97 |
| E7EPK0 | LIM and calponin homology domains-containing protein 1 | LIMCH1 | 156 | S | 0.781 | 70.91 | 2.13 | 2.27 | NaN | NaN | NaN | NaN | NaN | NaN | NaN |
| E7EPN9 | Protein PRRC2C | PRRC2C | 189 | S | 0.983 | 150.2 | NaN | 1.44 | 1.03 | 2.07 | NaN | NaN | NaN | NaN | 0.75 |
| E7EPN9 | Protein PRRC2C | PRRC2C | 2107 | S | 1 | 121.8 | 1.13 | 0.99 | 1.00 | 1.43 | 1.57 | 1.27 | 0.71 | 0.72 | NaN |
| E7EPN9 | Protein PRRC2C | PRRC2C | 880 | S | 0.834 | 63.72 | NaN | NaN | 1.83 | 1.59 | 2.15 | NaN | 1.82 | 1.65 | NaN |
| E9PGG1 | | DYNC1I2 | 81 | S | 0.768 | 146.5 | 0.82 | 0.89 | 0.83 | 1.37 | 1.35 | 1.33 | 0.54 | 0.58 | 0.52 |
| S4R3H4 | Apoptotic chromatin condensation inducer in the nucleus | ACIN1 | 432 | S | 1 | 221.6 | 0.67 | 0.68 | 0.66 | 0.67 | 0.74 | 0.72 | 1.18 | 1.17 | 1.18 |
| S4R3H4 | Apoptotic chromatin condensation inducer in the nucleus | ACIN1 | 352 | S | 0.952 | 112.6 | NaN | 0.97 | 1.00 | 1.07 | 1.19 | 1.14 | NaN | 1.11 | 0.97 |
| S4R3H4 | Apoptotic chromatin condensation inducer in the nucleus | ACIN1 | 671 | S | 1 | 84.9 | 0.79 | NaN | 0.93 | 0.94 | NaN | 0.92 | 0.95 | NaN | 1.04 |
| S4R3H4 | Apoptotic chromatin condensation inducer in the nucleus | ACIN1 | 652 | S | 1 | 78.53 | 1.70 | 1.79 | 1.62 | 1.45 | 1.38 | 0.83 | 1.56 | 1.42 | 2.03 |
| S4R3H4 | Apoptotic chromatin condensation inducer in the nucleus | ACIN1 | 326 | S | 0.968 | 45.09 | NaN | NaN | NaN | 0.73 | 0.79 | NaN | NaN | NaN | 0.80 |

| | | | | | | | | | | | | | | |
|---|---|---|---|---|---|---|---|---|---|---|---|---|---|---|
| S4R3H4 | Apoptotic chromatin condensation inducer in the nucleus | ACIN1 | 328 | S | 0.97 | 69.03 | NaN | NaN | NaN | NaN | NaN | NaN | 0.85 | 0.82 | NaN |
| S4R3H4 | Apoptotic chromatin condensation inducer in the nucleus | ACIN1 | 330 | S | 0.982 | 69.03 | NaN | NaN | NaN | 0.73 | 0.79 | NaN | 0.85 | 0.82 | 0.80 |
| S4R3H4 | Apoptotic chromatin condensation inducer in the nucleus | ACIN1 | 597 | S | 1 | 92.43 | 0.78 | NaN | NaN | 0.79 | 0.85 | NaN | 0.84 | 0.80 | NaN |
| S4R3H4 | Apoptotic chromatin condensation inducer in the nucleus | ACIN1 | 599 | S | 1 | 92.43 | 0.78 | NaN | NaN | 0.34 | 0.37 | NaN | 0.54 | 0.53 | NaN |
| S4R3H4 | Apoptotic chromatin condensation inducer in the nucleus | ACIN1 | 158 | S | 1 | 284.4 | 0.79 | 0.72 | 0.76 | 0.83 | 0.81 | 0.76 | 0.94 | 0.95 | 0.96 |
| S4R3H4 | Apoptotic chromatin condensation inducer in the nucleus | ACIN1 | 946 | S | 1 | 140.2 | 0.96 | 1.02 | 0.94 | 0.85 | 0.89 | 0.89 | 0.77 | 0.77 | 1.05 |
| S4R3H4 | Apoptotic chromatin condensation inducer in the nucleus | ACIN1 | 307 | S | 0.999 | 106.5 | NaN | 0.98 | NaN | 1.20 | 1.30 | NaN | NaN | NaN | NaN |
| E7ERS3 | Zinc finger CCCH domain-containing protein 18 | ZC3H18 | 46 | S | 1 | 86.8 | 1.61 | 2.04 | NaN | 1.72 | 2.03 | NaN | NaN | NaN | NaN |
| E7ERS3 | Zinc finger CCCH domain-containing protein 18 | ZC3H18 | 67 | S | 1 | 148.3 | 0.74 | 0.68 | NaN | 0.86 | NaN | 0.88 | NaN | NaN | NaN |
| E7ERS3 | Zinc finger CCCH domain-containing protein 18 | ZC3H18 | 95 | S | 0.891 | 83.32 | 0.89 | 0.72 | NaN | NaN | 1.35 | 1.12 | NaN | NaN | NaN |
| E7ERS3 | Zinc finger CCCH domain-containing protein 18 | ZC3H18 | 558 | S | 0.959 | 77.74 | 0.95 | 1.00 | 1.10 | 0.99 | 1.06 | 1.06 | 1.11 | 1.12 | 1.13 |
| E7ERS3 | Zinc finger CCCH domain-containing protein 18 | ZC3H18 | 917 | S | 1 | 115.8 | 1.06 | 1.05 | 1.08 | 1.00 | 1.01 | 1.05 | 1.05 | 1.06 | 0.99 |
| E7ERS3 | Zinc finger CCCH domain-containing protein 18 | ZC3H18 | 866 | S | 1 | 79.57 | 0.98 | 0.75 | 0.98 | 0.63 | 0.91 | NaN | 0.74 | 0.97 | NaN |
| F8W7U3 | WASH complex subunit FAM21A | FAM21A | 451 | S | 1 | 303.5 | 1.32 | 1.19 | 1.21 | 1.51 | 0.49 | 1.25 | 1.21 | 1.04 | 1.08 |
| E7ETM8 | Nexilin | NEXN | 16 | S | 1 | 306.3 | 0.95 | 1.00 | 0.91 | 1.03 | 1.12 | 1.09 | 0.77 | 0.81 | 0.85 |
| E7ETM8 | Nexilin | NEXN | 179 | S | 0.995 | 65.56 | 0.71 | 0.84 | 0.80 | 1.02 | 0.96 | 1.00 | 0.88 | 0.91 | 0.92 |
| Q13428 | Treacle protein | TCOF1 | 906;906 | S | 1 | 73.44 | 3.68 | 4.07 | 3.96 | 3.29 | 2.88 | 2.78 | 2.25 | 2.43 | 2.17 |
| Q13428 | Treacle protein | TCOF1 | 375;375 | S | 0.774 | 99.6 | 1.24 | 1.27 | NaN | NaN | NaN | NaN | 1.68 | NaN | NaN |
| Q13428 | Treacle protein | TCOF1 | 381;381 | S | 1 | 200 | 2.13 | 1.63 | 1.59 | 1.44 | 1.74 | 1.62 | 1.23 | 1.23 | 1.13 |
| Q13428 | Treacle protein | TCOF1 | 233;233 | S | 0.944 | 64.21 | NaN | 3.91 | 0.53 | 0.88 | 0.92 | 0.86 | 0.79 | 0.82 | NaN |
| Q13428 | Treacle protein | TCOF1 | 1219;125 | S | 1 | 95.43 | 0.86 | 1.00 | 0.81 | 1.52 | 1.37 | 1.46 | 0.63 | 0.71 | 0.57 |
| Q13428 | Treacle protein | TCOF1 | 1312;135 | S | 1 | 94.02 | 1.18 | 1.22 | 1.02 | 1.07 | 1.13 | 1.00 | 1.05 | 0.91 | 0.90 |
| J3KQ96 | | TCOF1 | 1073 | S | 0.845 | 103.8 | NaN | 0.69 | NaN | 1.04 | NaN | NaN | 0.80 | NaN | 1.49 |
| Q13428 | Treacle protein | TCOF1 | 171;171 | S | 0.962 | 140.2 | 0.97 | 1.51 | NaN | 1.60 | NaN | 1.50 | 2.32 | NaN | 2.36 |
| Q13428 | Treacle protein | TCOF1 | 1190;122 | S | 0.918 | 109.1 | 0.78 | 0.97 | NaN | NaN | 0.76 | NaN | NaN | NaN | NaN |
| Q13428 | Treacle protein | TCOF1 | 1340;137 | S | 0.857 | 73.75 | 0.79 | 0.81 | NaN | NaN | NaN | NaN | NaN | NaN | NaN |

| | | | | | | | | | | | | | | | |
|---|---|---|---|---|---|---|---|---|---|---|---|---|---|---|---|
| Q13428 | Treacle protein | TCOF1 | 156;156 | S | 1 | 138.3 | 1.05 | 1.03 | 1.13 | 1.53 | 1.47 | 1.42 | 0.91 | 0.96 | 1.02 |
| E7ETY4 | Serine/threonine-protein kinase MARK2 | MARK2 | 452 | S | 0.952 | 65.33 | 1.34 | 1.73 | NaN | NaN | NaN | NaN | NaN | NaN | NaN |
| E7EUU4 | Eukaryotic translation initiation factor 4 gamma 1 | EIF4G1 | 1192 | S | 1 | 148.7 | 2.00 | 2.09 | 2.12 | 2.52 | 2.39 | 2.38 | 1.18 | 1.16 | 1.10 |
| E7EUU4 | Eukaryotic translation initiation factor 4 gamma 1 | EIF4G1 | 1053 | S | 1 | 96.46 | 1.59 | 1.91 | 2.06 | NaN | NaN | 1.82 | 1.58 | NaN | 1.28 |
| E7EUU4 | Eukaryotic translation initiation factor 4 gamma 1 | EIF4G1 | 1148 | S | 1 | 78.16 | 1.97 | 1.84 | 1.99 | 1.52 | 1.44 | NaN | NaN | 1.94 | NaN |
| E7EUV9 | Histone RNA hairpin-binding protein | SLBP | 20 | S | 1 | 61.27 | NaN | 0.69 | 0.79 | 0.97 | 1.04 | NaN | 0.88 | 0.64 | 0.67 |
| E7EUV9 | Histone RNA hairpin-binding protein | SLBP | 23 | S | 1 | 61.27 | NaN | 0.69 | 0.79 | 0.97 | 1.12 | NaN | 0.53 | 0.64 | 0.67 |
| E7EVX2 | Golgin subfamily A member 4 | GOLGA4 | 271 | S | 1 | 50.87 | NaN | NaN | 0.97 | NaN | NaN | NaN | NaN | 0.90 | 1.26 |
| E7EX17 | Eukaryotic translation initiation factor 4B | EIF4B | 464 | S | 0.935 | 78.1 | 1.01 | 0.90 | NaN | NaN | NaN | NaN | NaN | NaN | NaN |
| E7EX17 | Eukaryotic translation initiation factor 4B | EIF4B | 93 | S | 0.878 | 77.24 | 1.60 | NaN | NaN | 1.63 | NaN | NaN | 1.08 | NaN | 0.97 |
| E7EX17 | Eukaryotic translation initiation factor 4B | EIF4B | 500 | S | 0.663 | 112 | NaN | NaN | NaN | 2.23 | NaN | 1.69 | NaN | 1.26 | NaN |
| E7EX17 | Eukaryotic translation initiation factor 4B | EIF4B | 502 | S | 0.99 | 313.6 | 2.83 | 1.85 | 2.01 | 2.23 | 1.60 | 1.69 | NaN | NaN | 1.30 |
| E7EX17 | Eukaryotic translation initiation factor 4B | EIF4B | 503 | S | 0.997 | 287.5 | 1.93 | NaN | NaN | 1.55 | 1.64 | 1.75 | 1.30 | NaN | NaN |
| E7EX17 | Eukaryotic translation initiation factor 4B | EIF4B | 509 | S | 1 | 302.9 | 0.63 | 0.60 | 0.67 | 0.80 | 0.77 | 0.76 | 0.75 | 0.76 | 0.75 |
| E7EX40 | Rab11 family-interacting protein 1 | RAB11FIP1 | 51 | S | 0.568 | 98.75 | NaN | NaN | NaN | NaN | 0.74 | NaN | 0.71 | 0.91 | NaN |
| E7EX40 | Rab11 family-interacting protein 1 | RAB11FIP1 | 54 | S | 0.985 | 141.2 | 0.79 | NaN | 0.73 | 0.76 | 0.81 | NaN | 0.84 | NaN | 0.72 |
| E7EX40 | Rab11 family-interacting protein 1 | RAB11FIP1 | 190 | S | 0.559 | 70.33 | 0.95 | 0.86 | NaN | NaN | NaN | 0.92 | 0.85 | NaN | NaN |
| E7EX40 | Rab11 family-interacting protein 1 | RAB11FIP1 | 191 | S | 0.998 | 116.5 | NaN | NaN | NaN | 0.45 | 0.57 | NaN | 0.98 | NaN | 0.39 |
| E7EX40 | Rab11 family-interacting protein 1 | RAB11FIP1 | 193 | S | 0.787 | 110.2 | 0.95 | NaN | NaN | NaN | 0.75 | 0.92 | NaN | 0.85 | 0.78 |
| E7EX40 | Rab11 family-interacting protein 1 | RAB11FIP1 | 195 | S | 0.669 | 110.2 | NaN | NaN | NaN | NaN | 0.75 | NaN | NaN | 0.85 | 0.78 |
| E7EX40 | Rab11 family-interacting protein 1 | RAB11FIP1 | 197 | S | 1 | 110.2 | 0.95 | 0.86 | NaN | 1.01 | 0.75 | 0.92 | 0.85 | 0.85 | 0.78 |
| E7EX40 | Rab11 family-interacting protein 1 | RAB11FIP1 | 380 | S | 0.617 | 64.22 | NaN | 2.58 | 2.47 | 2.39 | 1.80 | NaN | NaN | 1.78 | 1.78 |
| E7EX40 | Rab11 family-interacting protein 1 | RAB11FIP1 | 381 | S | 0.5 | 63.73 | NaN | 2.58 | 2.47 | 2.39 | 1.80 | NaN | NaN | 1.78 | 1.78 |
| E7EX44 | Caldesmon | CALD1 | 553 | S | 0.918 | 105.8 | 1.05 | 1.13 | NaN | 1.30 | 1.40 | NaN | 1.00 | 0.97 | 1.00 |
| E7EX44 | | CALD1 | 196 | S | 1 | 60.6 | 0.82 | NaN | 1.00 | NaN | NaN | NaN | NaN | NaN | 0.90 |

| | | | | | | | | | | | | | | | |
|---|---|---|---|---|---|---|---|---|---|---|---|---|---|---|---|
| F8VZJ2 | Nascent polypeptide-associated complex subunit alpha;Nascent polypeptide-associated complex subunit alpha, muscle-specific form | NACA | 87 | S | 1 | 301.7 | 1.15 | 1.04 | 1.03 | 1.05 | 1.04 | 1.01 | 1.08 | 1.06 | 1.00 |
| E9PCY7 | Heterogeneous nuclear ribonucleoprotein H;Heterogeneous nuclear ribonucleoprotein H, N-terminally processed;Heterogeneous nuclear ribonucleoprotein F;Heterogeneous nuclear ribonucleoprotein F, N- | HNRNPH1 | 310 | S | 1 | 81.88 | NaN | 1.60 | NaN | 1.99 | 1.67 | NaN | NaN | NaN | NaN |
| E9PCY7 | Heterogeneous nuclear ribonucleoprotein H;Heterogeneous nuclear ribonucleoprotein H, N-terminally processed;Heterogeneous nuclear ribonucleoprotein H2 | HNRNPH1 | 104;104 | S | 1 | 235.3 | 1.69 | 1.76 | 1.68 | 2.03 | 2.08 | 2.03 | 1.05 | 1.03 | 0.98 |
| H0YF64 | Calcipressin-3;Calcipressin-1 | RCAN3 | 15 | S | 1 | 81.35 | 0.52 | 0.62 | 0.59 | NaN | 0.54 | 0.63 | 0.78 | 0.70 | 0.76 |
| H0YF64 | Calcipressin-3;Calcipressin-1 | RCAN3 | 19 | S | 1 | 81.35 | 0.72 | 0.99 | 0.59 | NaN | 0.54 | 0.63 | 0.78 | 0.70 | 0.77 |
| E9PER6 | Putative 3-phosphoinositide-dependent protein kinase 2;3-phosphoinositide-dependent protein kinase 1 | PDPK1 | 214 | S | 0.822 | 49.19 | 0.88 | 1.06 | NaN | 1.05 | 2.65 | NaN | 2.30 | 1.45 | NaN |
| E9PF10 | Nuclear pore complex protein Nup155 | NUP155 | 928 | S | 0.989 | 125.4 | 1.49 | 1.61 | 1.57 | 1.54 | 1.59 | 1.51 | 1.23 | 1.28 | 1.27 |
| E9PFP1 | Phosphatidylinositol 3-kinase regulatory subunit beta | PIK3R2 | 262 | S | 0.537 | 85.2 | NaN | 0.94 | 0.93 | 1.05 | 1.00 | 1.00 | 1.09 | NaN | 1.01 |
| E9PFP1 | Phosphatidylinositol 3-kinase regulatory subunit beta | PIK3R2 | 263 | S | 0.5 | 85.2 | NaN | NaN | 0.93 | 1.05 | 1.00 | NaN | NaN | NaN | NaN |
| G5E9V3 | Pleckstrin homology-like domain family B member 2 | PHLDB2 | 489 | S | 1 | 152.2 | 0.69 | 0.81 | 0.78 | 0.78 | 0.64 | 0.88 | 0.80 | NaN | 0.86 |
| G5E9V3 | Pleckstrin homology-like domain family B member 2 | PHLDB2 | 204 | S | 0.96 | 184.3 | 1.53 | 1.48 | 1.33 | 1.62 | 1.51 | 1.29 | 1.62 | NaN | 1.84 |
| E9PFQ8 | Serine/threonine-protein kinase RIO1 | RIOK1 | 21 | S | 0.708 | 134.2 | 1.07 | NaN | 1.13 | 0.97 | NaN | 0.89 | 1.34 | NaN | NaN |
| E9PFQ8 | Serine/threonine-protein kinase RIO1 | RIOK1 | 22 | S | 0.742 | 167.1 | NaN | 0.87 | 0.75 | NaN | 1.19 | 0.88 | NaN | 1.43 | 1.26 |
| E9PG73 | Peptidyl-prolyl cis-trans isomerase G | PPIG | 672 | S | 0.999 | 106.2 | 1.25 | NaN | NaN | 0.91 | 0.98 | NaN | 1.07 | 1.08 | 1.12 |
| E9PG73 | Peptidyl-prolyl cis-trans isomerase G | PPIG | 702 | S | 0.909 | 84.84 | NaN | 0.90 | NaN | 1.04 | 1.01 | 1.01 | 1.07 | 0.91 | NaN |

| | | | | | | | | | | | | | | | |
|---|---|---|---|---|---|---|---|---|---|---|---|---|---|---|---|
| E9PG73 | Peptidyl-prolyl cis-trans isomerase G | PPIG | 275 | S | 1 | 55.26 | NaN | NaN | 0.76 | 0.96 | 0.88 | NaN | 0.72 | NaN | NaN |
| E9PG73 | Peptidyl-prolyl cis-trans isomerase G | PPIG | 239 | S | 0.917 | 128.6 | 1.15 | 1.15 | 1.11 | 1.08 | NaN | 1.03 | NaN | 1.16 | 1.36 |
| E9PG73 | Peptidyl-prolyl cis-trans isomerase G | PPIG | 241 | S | 0.997 | 128.6 | 1.15 | 1.15 | 1.11 | 1.08 | NaN | 1.03 | NaN | 1.16 | 1.36 |
| E9PG73 | Peptidyl-prolyl cis-trans isomerase G | PPIG | 242 | S | 0.999 | 128.6 | 1.15 | 1.15 | 1.11 | 1.08 | NaN | 1.03 | NaN | 1.16 | 1.36 |
| E9PG73 | Peptidyl-prolyl cis-trans isomerase G | PPIG | 244 | S | 1 | 128.6 | 1.15 | 1.15 | 1.11 | 1.08 | NaN | 1.03 | NaN | 1.16 | 1.36 |
| E9PGC0 | Ras GTPase-activating protein 1 | RASA1 | 665 | S | 0.944 | 56.34 | NaN | NaN | NaN | NaN | 1.44 | 1.56 | NaN | 0.96 | NaN |
| E9PHI6 | Cytoplasmic dynein 1 light intermediate chain 1 | DYNC1LI1 | 91 | S | 1 | 265.6 | 1.14 | 1.15 | 1.23 | 1.23 | 1.25 | 1.18 | 0.95 | 1.00 | 1.01 |
| E9PHI6 | Cytoplasmic dynein 1 light intermediate chain 1 | DYNC1LI1 | 394 | S | 0.981 | 160.6 | 1.18 | 1.03 | 1.57 | 1.70 | 0.68 | 2.29 | NaN | 0.93 | 0.82 |
| E9PHI6 | Cytoplasmic dynein 1 light intermediate chain 1 | DYNC1LI1 | 400 | S | 0.999 | 176.1 | 0.98 | 0.98 | 1.03 | 0.81 | 1.03 | 0.80 | 0.89 | 0.93 | 0.94 |
| E9PHI6 | Cytoplasmic dynein 1 light intermediate chain 1 | DYNC1LI1 | 305 | S | 1 | 231.6 | 1.55 | NaN | NaN | 5.06 | 5.31 | NaN | NaN | NaN | NaN |
| E9PHV6 | Nuclear autoantigen Sp-100 | SP100 | 132 | S | 1 | 133.2 | 1.13 | 1.14 | NaN | NaN | 1.23 | 1.22 | 1.05 | 1.28 | 1.06 |
| E9PHV6 | Nuclear autoantigen Sp-100 | SP100 | 426 | S | 0.647 | 77.69 | 0.90 | 1.33 | NaN | 1.57 | 1.25 | NaN | NaN | NaN | NaN |
| E9PHV6 | Nuclear autoantigen Sp-100 | SP100 | 427 | S | 0.648 | 77.69 | 0.90 | 1.33 | NaN | 1.57 | 1.25 | NaN | NaN | NaN | NaN |
| E9PHV6 | Nuclear autoantigen Sp-100 | SP100 | 428 | S | 0.65 | 77.69 | 0.90 | 1.33 | NaN | 1.57 | 1.25 | NaN | NaN | NaN | NaN |
| E9PHV6 | Nuclear autoantigen Sp-100 | SP100 | 382 | S | 1 | 130.5 | 0.27 | NaN | NaN | NaN | 0.38 | NaN | 1.07 | 0.86 | 0.82 |
| E9PHV6 | Nuclear autoantigen Sp-100 | SP100 | 384 | S | 1 | 130.5 | 0.27 | NaN | NaN | NaN | 0.38 | NaN | 0.84 | 0.86 | 0.82 |
| E9PHV6 | Nuclear autoantigen Sp-100 | SP100 | 385 | S | 1 | 130.5 | 0.27 | NaN | NaN | NaN | 0.38 | NaN | 0.84 | 0.86 | 0.82 |
| E9PHV6 | Nuclear autoantigen Sp-100 | SP100 | 337 | S | 1 | 167.6 | 1.09 | 1.20 | 1.55 | 1.40 | NaN | 1.62 | 1.02 | NaN | 1.18 |
| E9PK52 | Band 4.1-like protein 2 | EPB41L2 | 645 | S | 1 | 110.4 | NaN | NaN | 1.13 | 1.57 | 1.54 | 1.41 | 0.97 | 0.95 | NaN |
| E9PK52 | Band 4.1-like protein 2 | EPB41L2 | 38 | S | 0.762 | 190.1 | 1.07 | NaN | 1.15 | 1.00 | 1.25 | 1.23 | NaN | 1.43 | NaN |
| E9PK52 | Band 4.1-like protein 2 | EPB41L2 | 39 | S | 0.938 | 213.4 | NaN | 1.18 | 1.05 | 0.99 | 1.27 | 1.23 | 0.87 | 1.10 | 1.20 |
| E9PK52 | Band 4.1-like protein 2 | EPB41L2 | 598 | S | 0.868 | 80.98 | 1.72 | 1.77 | NaN | 2.07 | 2.09 | 1.84 | NaN | NaN | 0.83 |
| E9PI52 | Arginine/serine-rich coiled-coil protein 2 | RSRC2 | 17 | S | 0.751 | 92.45 | 0.83 | 0.95 | 1.04 | 0.97 | 1.10 | 0.81 | NaN | 0.88 | 1.41 |
| E9PI52 | Arginine/serine-rich coiled-coil protein 2 | RSRC2 | 32 | S | 0.999 | 159.6 | 1.18 | 1.19 | 1.23 | 1.23 | 1.32 | 1.07 | 0.99 | 0.96 | 0.82 |
| E9PIE3 | Protein kinase C delta-binding protein | PRKCDBP | 197 | S | 0.998 | 67.2 | 1.63 | 1.49 | 1.45 | 1.34 | NaN | 1.33 | 1.66 | 1.61 | 1.72 |
| E9PIE3 | Protein kinase C delta-binding protein | PRKCDBP | 198 | S | 0.998 | 67.2 | 1.63 | 1.49 | 1.45 | 1.34 | NaN | 1.33 | 1.66 | 1.61 | 1.72 |
| E9PJ04 | Splicing factor 3B subunit 2 | SF3B2 | 300 | S | 0.761 | 67.19 | 1.20 | 1.21 | 1.26 | NaN | 1.16 | NaN | 1.04 | 1.43 | 1.38 |
| E9PJ04 | Splicing factor 3B subunit 2 | SF3B2 | 301 | S | 0.775 | 67.19 | 1.20 | 1.21 | 1.26 | NaN | 1.16 | NaN | NaN | 1.43 | 1.38 |
| E9PJ04 | Splicing factor 3B subunit 2 | SF3B2 | 305 | S | 1 | 115.1 | 1.10 | 1.09 | 1.11 | 1.20 | 1.12 | 1.13 | 0.90 | 0.93 | 0.86 |
| E9PJ04 | Splicing factor 3B subunit 2 | SF3B2 | 307 | S | 0.995 | 115.1 | 1.16 | 1.10 | 1.11 | 1.20 | 1.12 | 1.13 | 0.90 | 0.93 | 0.86 |
| E9PJ38 | Receptor-binding cancer antigen expressed on SiSo | EBAG9 | 36 | S | 1 | 107.7 | 0.84 | 0.88 | 0.82 | 0.86 | NaN | 0.91 | 0.80 | 0.86 | 0.85 |

| | | | | | | | | | | | | | | | |
|---|---|---|---|---|---|---|---|---|---|---|---|---|---|---|---|
| E9PJ60 | Probable ATP-dependent RNA helicase DDX20 | DDX20 | 285 | S | 1 | 116.3 | 0.96 | 0.91 | 0.93 | 0.83 | 0.95 | 0.93 | 1.06 | 1.02 | 0.99 |
| E9PJ60 | Probable ATP-dependent RNA helicase DDX20 | DDX20 | 286 | S | 1 | 116.3 | 0.96 | 0.91 | 0.93 | 0.83 | 0.95 | 0.93 | 1.06 | 1.02 | 0.99 |
| E9PJF4 | Methylosome subunit pICln | CLNS1A | 102 | S | 1 | 330 | 1.06 | 1.11 | 1.09 | 1.06 | 1.10 | 1.07 | 0.98 | 1.00 | 1.10 |
| E9PK91 | Bcl-2-associated transcription factor 1 | BCLAF1 | 177 | S | 1 | 193.6 | 0.99 | 0.93 | 0.84 | 0.97 | 1.01 | 0.96 | 0.91 | 0.81 | 1.01 |
| E9PK91 | Bcl-2-associated transcription factor 1 | BCLAF1 | 385 | S | 0.988 | 169.7 | 2.28 | 2.78 | NaN | 1.71 | 1.29 | NaN | 2.13 | 2.47 | 2.58 |
| E9PK91 | Bcl-2-associated transcription factor 1 | BCLAF1 | 512 | S | 1 | 158.6 | 0.89 | 0.88 | 0.90 | 0.94 | 0.92 | 0.96 | 0.93 | 0.93 | 1.00 |
| E9PK91 | Bcl-2-associated transcription factor 1 | BCLAF1 | 496 | S | 1 | 196.2 | 0.85 | 0.92 | 0.87 | 0.96 | 0.96 | 0.90 | 1.02 | 0.87 | 0.94 |
| E9PK91 | Bcl-2-associated transcription factor 1 | BCLAF1 | 397 | S | 1 | 338.7 | 1.06 | 1.07 | 1.09 | 1.02 | 1.03 | 0.97 | 1.14 | 1.21 | 1.04 |
| E9PK91 | Bcl-2-associated transcription factor 1 | BCLAF1 | 658 | S | 1 | 149.2 | 1.01 | 1.01 | 1.16 | 1.01 | 0.99 | 0.96 | 1.08 | 0.93 | 1.07 |
| E9PK91 | Bcl-2-associated transcription factor 1 | BCLAF1 | 268 | S | 0.999 | 181.6 | 1.06 | 1.07 | 1.00 | 1.03 | 0.99 | 0.93 | 0.88 | 0.97 | 0.92 |
| E9PK91 | Bcl-2-associated transcription factor 1 | BCLAF1 | 648 | S | 1 | 96.95 | 1.04 | 1.15 | 1.20 | 1.03 | 1.12 | 1.04 | 0.93 | 1.02 | 1.19 |
| E9PK91 | Bcl-2-associated transcription factor 1 | BCLAF1 | 183 | S | 0.997 | 101.3 | 1.20 | 1.36 | NaN | 2.09 | NaN | NaN | 4.56 | NaN | NaN |
| E9PK91 | Bcl-2-associated transcription factor 1 | BCLAF1 | 222 | S | 1 | 193.7 | 0.56 | 0.64 | 0.80 | 0.71 | 0.82 | 0.68 | 1.00 | 0.94 | 0.94 |
| E9PK91 | Bcl-2-associated transcription factor 1 | BCLAF1 | 531 | S | 1 | 113.9 | 2.19 | 2.19 | 2.21 | 1.87 | 1.48 | 1.61 | 2.12 | 2.13 | 2.21 |
| E9PK91 | Bcl-2-associated transcription factor 1 | BCLAF1 | 285 | S | 0.978 | 105.5 | 0.59 | 0.72 | NaN | 0.86 | 0.83 | 1.30 | NaN | 1.61 | 1.77 |
| E9PK91 | Bcl-2-associated transcription factor 1 | BCLAF1 | 287 | S | 0.931 | 89.44 | NaN | 1.45 | NaN | 1.44 | 1.55 | 1.30 | NaN | 1.27 | NaN |
| F5H0D8 | | CELF1 | 18 | S | 0.726 | 134 | 0.93 | 0.74 | NaN | NaN | NaN | NaN | NaN | NaN | NaN |
| E9PS47 | Phosphatidylserine synthase 2 | PTDSS2 | 16 | S | 1 | 57.29 | 1.65 | 1.66 | NaN | NaN | NaN | NaN | NaN | NaN | NaN |
| H0YE74 | Cleavage stimulation factor subunit 3 | CSTF3 | 65 | S | 1 | 197.8 | 1.10 | 1.07 | 0.98 | 1.08 | 1.02 | 1.09 | 0.87 | 0.80 | 0.97 |
| E9PQA1 | Small acidic protein | C11orf58 | 15 | S | 0.992 | 281.5 | NaN | 1.29 | 1.36 | 1.21 | 1.14 | 1.20 | 1.33 | 1.25 | NaN |
| E9PQA1 | Small acidic protein | C11orf58 | 17 | S | 1 | 663 | 1.27 | 1.25 | 1.16 | 1.13 | 0.91 | 1.08 | 1.05 | 1.07 | 1.35 |
| E9PMG1 | RalBP1-associated Eps domain-containing protein 1 | REPS1 | 650 | S | 1 | 171.8 | 1.26 | 1.25 | 1.24 | 1.03 | 0.98 | 1.51 | 1.32 | 1.31 | 1.29 |
| E9PMG1 | RalBP1-associated Eps domain-containing protein 1 | REPS1 | 535 | S | 0.965 | 64.1 | NaN | 1.59 | 1.82 | 1.80 | 1.72 | 1.76 | 1.17 | 1.21 | NaN |
| E9PMG1 | RalBP1-associated Eps domain-containing protein 1 | REPS1 | 455 | S | 0.746 | 49.09 | 1.32 | NaN | NaN | NaN | NaN | NaN | 1.12 | 1.38 | NaN |
| E9PMG1 | RalBP1-associated Eps domain-containing protein 1 | REPS1 | 162 | S | 1 | 93.79 | NaN | 1.62 | NaN | 1.51 | 1.70 | NaN | NaN | NaN | NaN |
| E9PMG1 | RalBP1-associated Eps domain-containing protein 1 | REPS1 | 166 | S | 1 | 93.79 | NaN | 1.62 | NaN | 1.51 | 1.70 | NaN | NaN | NaN | NaN |

| | | | | | | | | | | | | | | | |
|---|---|---|---|---|---|---|---|---|---|---|---|---|---|---|---|
| E9PMG1 | RalBP1-associated Eps domain-containing protein 1 | REPS1 | 170 | S | 0.995 | 177.1 | NaN | 1.62 | 0.75 | 0.82 | 1.70 | 0.77 | NaN | NaN | 0.84 |
| E9PMG4 | Telomerase Cajal body protein 1 | WRAP53 | 85 | S | 0.951 | 82.49 | NaN | NaN | 0.67 | 0.43 | 0.55 | NaN | 1.19 | 1.18 | 1.17 |
| E9PMG4 | Telomerase Cajal body protein 1 | WRAP53 | 90 | S | 0.974 | 82.49 | NaN | NaN | 0.67 | 0.43 | 0.55 | NaN | 1.19 | 1.18 | 1.17 |
| E9PMG4 | Telomerase Cajal body protein 1 | WRAP53 | 54 | S | 1 | 85.19 | 0.71 | 0.68 | NaN | 0.76 | 0.78 | NaN | 0.97 | 1.20 | NaN |
| E9PMG4 | Telomerase Cajal body protein 1 | WRAP53 | 458 | S | 0.985 | 182.3 | 0.51 | 0.61 | 0.60 | 0.53 | NaN | 0.67 | 1.39 | NaN | 1.43 |
| E9PMW5 | Tripartite motif-containing protein 3 | TRIM3 | 7 | S | 1 | 100.6 | 0.54 | 0.53 | 0.70 | 0.71 | NaN | 0.81 | 0.57 | 0.66 | 0.62 |
| E9PMQ6 | Heat shock factor protein 1 | HSF1 | 363 | S | 0.999 | 127.3 | 0.75 | 0.65 | 0.69 | 0.57 | NaN | 0.58 | 1.13 | 1.08 | 1.05 |
| E9PMQ6 | Heat shock factor protein 1 | HSF1 | 314 | S | 0.714 | 81.13 | NaN | NaN | NaN | 0.69 | 0.78 | NaN | NaN | NaN | NaN |
| E9PMR6 | Rho guanine nucleotide exchange factor 12 | ARHGEF 12 | 1286 | S | 1 | 75.1 | 0.57 | 1.45 | NaN | NaN | NaN | NaN | NaN | NaN | NaN |
| E9PMR6 | Rho guanine nucleotide exchange factor 12 | ARHGEF 12 | 206 | S | 0.995 | 71.3 | 0.92 | 0.77 | NaN | 1.16 | 0.87 | 1.16 | NaN | 1.06 | 1.23 |
| E9PMS6 | LIM domain only protein 7 | LMO7 | 591 | S | 1 | 130.8 | 0.69 | 0.83 | 0.66 | 0.87 | 0.99 | 0.98 | 0.78 | 0.75 | 0.78 |
| E9PMS6 | LIM domain only protein 7 | LMO7 | 155 | S | 1 | 89.99 | 1.95 | 2.02 | NaN | NaN | 0.80 | NaN | 1.86 | NaN | 1.37 |
| E9PMS6 | LIM domain only protein 7 | LMO7 | 553 | S | 0.999 | 243.8 | 0.72 | 0.67 | 0.69 | 0.56 | 0.71 | 0.65 | 0.64 | 0.65 | 0.65 |
| E9PMS6 | LIM domain only protein 7 | LMO7 | 556 | S | 0.95 | 218.7 | 2.14 | 1.62 | 1.78 | 2.06 | NaN | 1.85 | 1.29 | NaN | 1.31 |
| E9PMS6 | LIM domain only protein 7 | LMO7 | 1102 | S | 1 | 96.33 | 5.41 | 3.75 | NaN | NaN | NaN | NaN | NaN | NaN | NaN |
| E9PMS6 | LIM domain only protein 7 | LMO7 | 1085 | S | 0.983 | 60.03 | 1.41 | 2.46 | NaN | NaN | NaN | NaN | NaN | NaN | NaN |
| E9PMS6 | LIM domain only protein 7 | LMO7 | 1185 | S | 0.999 | 80.47 | NaN | 2.87 | 2.39 | 1.93 | 1.64 | 1.72 | 1.19 | 1.36 | 1.15 |
| E9PMS6 | LIM domain only protein 7 | LMO7 | 1015 | S | 0.859 | 103.4 | NaN | NaN | NaN | NaN | NaN | NaN | 0.86 | 0.89 | NaN |
| E9PMS6 | LIM domain only protein 7 | LMO7 | 432 | S | 0.998 | 169.7 | NaN | 1.31 | 1.40 | 1.23 | 1.24 | 1.20 | 1.09 | NaN | 1.01 |
| E9PNJ4 | Stromal interaction molecule 1 | STIM1 | 348 | S | 0.533 | 66.64 | NaN | NaN | NaN | NaN | 2.21 | NaN | 2.28 | NaN | 1.77 |
| E9PNJ4 | Stromal interaction molecule 1 | STIM1 | 445 | S | 0.956 | 242.1 | 1.11 | NaN | NaN | 0.98 | 0.99 | NaN | 1.11 | 1.45 | NaN |
| H0YDN6 | Diacylglycerol kinase;Diacylglycerol kinase zeta | DGKZ | 19 | S | 0.5 | 54.07 | 0.96 | 1.01 | NaN | 1.07 | 1.09 | NaN | 1.01 | 0.96 | NaN |
| H0YDN6 | Diacylglycerol kinase;Diacylglycerol kinase zeta | DGKZ | 20 | S | 0.5 | 54.07 | 0.96 | 1.01 | NaN | 1.07 | 1.09 | NaN | 1.01 | 0.96 | NaN |
| E9PP21 | Cysteine and glycine-rich protein 1 | CSRP1 | 159 | S | 1 | 202.8 | NaN | 1.03 | 1.18 | NaN | NaN | 1.23 | NaN | 0.81 | NaN |
| E9PPY3 | Ribosomal RNA-processing protein 8 | RRP8 | 62 | S | 0.99 | 109.6 | NaN | NaN | 1.28 | 1.03 | 1.10 | 0.78 | NaN | NaN | 1.19 |
| E9PPY3 | Ribosomal RNA-processing protein 8 | RRP8 | 64 | S | 0.999 | 109.6 | NaN | NaN | 1.28 | 1.03 | 1.10 | 0.78 | NaN | NaN | 1.19 |
| E9PPY3 | Ribosomal RNA-processing protein 8 | RRP8 | 104 | S | 1 | 80.41 | NaN | NaN | NaN | 0.90 | 0.87 | 1.00 | NaN | 1.16 | 1.29 |
| E9PPY3 | Ribosomal RNA-processing protein 8 | RRP8 | 106 | S | 1 | 80.41 | NaN | NaN | NaN | 0.90 | 0.87 | 1.00 | NaN | 1.16 | 1.29 |
| E9PQC4 | Kinetochore protein Nuf2 | NUF2 | 247 | S | 1 | 67.39 | 0.52 | 0.59 | 0.68 | 0.99 | 0.96 | 0.88 | 0.72 | 0.71 | 0.69 |

| | | | | | | | | | | | | | | |
|---|---|---|---|---|---|---|---|---|---|---|---|---|---|---|
| E9PQP7 | Serine/threonine-protein phosphatase 6 regulatory subunit 3 | PPP6R3 | 619 | S | 0.663 | 84.75 | 0.97 | 0.96 | NaN | NaN | NaN | NaN | NaN | NaN | NaN |
| E9PQP7 | Serine/threonine-protein phosphatase 6 regulatory subunit 3 | PPP6R3 | 385 | S | 1 | 299 | 0.86 | 0.87 | 0.86 | 0.89 | 0.91 | 0.83 | 0.98 | 0.93 | 0.93 |
| E9PRB3 | Deoxynucleotidyltransferase terminal-interacting protein 2 | DNTTIP2 | 124 | S | 0.972 | 132.4 | 1.24 | 1.27 | NaN | 1.61 | 1.65 | 1.53 | 0.89 | 0.75 | NaN |
| F2Z2E2 | Ras GTPase-activating-like protein IQGAP3 | IQGAP3 | 496 | S | 1 | 116.5 | 1.01 | NaN | NaN | 2.11 | 2.26 | 1.96 | NaN | 0.75 | NaN |
| F2Z2T1 | Nuclear cap-binding protein subunit 1 | NCBP1 | 22 | S | 0.729 | 90.4 | NaN | NaN | NaN | 0.95 | NaN | 0.85 | NaN | NaN | NaN |
| F2Z3H6 | B-cell CLL/lymphoma 7 protein family member B | BCL7B | 133 | S | 0.969 | 144.3 | NaN | NaN | NaN | 0.99 | 0.80 | NaN | 1.03 | NaN | NaN |
| F5GWT4 | Serine/threonine-protein kinase WNK1 | WNK1 | 1781 | S | 0.932 | 90.08 | 0.92 | NaN | 0.92 | 1.00 | 1.05 | 1.04 | 0.77 | 0.85 | 0.98 |
| F5GWT4 | Serine/threonine-protein kinase WNK1 | WNK1 | 1784 | S | 0.997 | 90.08 | 0.92 | 1.05 | 0.92 | 1.00 | 1.06 | 1.13 | 0.77 | 0.85 | 0.88 |
| F5GWT4 | Serine/threonine-protein kinase WNK1 | WNK1 | 1730 | S | 1 | 131.9 | 1.13 | 1.05 | 1.08 | 1.21 | 1.37 | 1.32 | 0.72 | 0.70 | 0.85 |
| F5GWT4 | Serine/threonine-protein kinase WNK1 | WNK1 | 185 | S | 0.746 | 67.65 | 0.71 | NaN | NaN | 0.91 | 0.69 | 0.84 | 0.82 | NaN | 0.75 |
| F5GX09 | Protein FAM76B | FAM76B | 193 | S | 1 | 118.5 | 1.41 | NaN | NaN | 0.59 | 0.48 | NaN | NaN | NaN | 0.54 |
| F5GX58 | Cell division cycle-associated protein 3 | CDCA3 | 87 | S | 0.998 | 47.53 | 1.61 | 1.23 | 1.76 | 2.84 | NaN | 2.58 | NaN | 0.88 | NaN |
| F5GYF8 | Signal-induced proliferation-associated 1-like protein 1 | SIPA1L1 | 1003 | S | 0.997 | 156.6 | NaN | 1.67 | NaN | 1.84 | 1.68 | NaN | 2.62 | 2.41 | NaN |
| F5GYF8 | Signal-induced proliferation-associated 1-like protein 1 | SIPA1L1 | 1039 | S | 0.998 | 133 | 1.31 | NaN | 1.63 | 1.21 | 1.29 | 1.53 | 1.84 | 2.08 | 1.84 |
| F5H3F0 | Ubiquitin thioesterase OTUB1 | OTUB1 | 16 | S | 0.847 | 89.68 | 0.67 | NaN | NaN | 0.92 | NaN | 1.10 | NaN | NaN | 0.80 |
| R4GN16 | Striatin-4 | STRN4 | 126 | S | 1 | 215.7 | 0.95 | 1.11 | 1.16 | NaN | 1.31 | 0.93 | 0.99 | 1.18 | 1.34 |
| F5H1K3 | tRNA 2'-phosphotransferase 1 | TRPT1 | 98 | S | 0.938 | 95.21 | NaN | NaN | 0.98 | 0.82 | NaN | NaN | 0.83 | 1.10 | 1.18 |
| F5H1U9 | Multiple PDZ domain protein | MPDZ | 483 | S | 0.998 | 77.37 | NaN | NaN | NaN | NaN | NaN | NaN | 0.95 | 0.73 | NaN |
| F5H1U9 | Multiple PDZ domain protein | MPDZ | 354 | S | 0.783 | 107.2 | 1.06 | 1.07 | NaN | 0.99 | NaN | NaN | 1.20 | 0.92 | 0.86 |
| F5H452 | Transcription factor Dp-1 | TFDP1 | 23 | S | 1 | 60.3 | 0.49 | 0.57 | NaN | 0.88 | 1.15 | NaN | 0.69 | 0.72 | NaN |
| F5H658 | ATP-dependent RNA helicase DHX8 | DHX8 | 460 | S | 1 | 60.3 | 0.43 | 0.45 | 0.49 | NaN | 0.69 | 0.78 | 0.38 | 0.41 | 0.38 |
| F5H6E2 | Unconventional myosin-Ic | MYO1C | 384 | S | 0.989 | 109.8 | 1.17 | 1.14 | 1.34 | 1.74 | 1.84 | 1.63 | 0.92 | 0.98 | 1.04 |
| F5H7D6 | Transducin-like enhancer protein 3 | TLE3 | 230 | S | 0.997 | 136.7 | NaN | NaN | NaN | 0.55 | NaN | NaN | 1.62 | 1.17 | 1.47 |
| F5H7D6 | Transducin-like enhancer protein 3 | TLE3 | 184 | S | 0.899 | 96.69 | NaN | 0.27 | 0.38 | 0.30 | 0.39 | NaN | 0.92 | NaN | 0.86 |
| F5H7D6 | Transducin-like enhancer protein 3 | TLE3 | 189 | S | 0.994 | 120.3 | NaN | NaN | 0.48 | 0.44 | 0.37 | 0.38 | 1.09 | NaN | 1.14 |
| F5H8D7 | DNA repair protein XRCC1 | XRCC1 | 415 | S | 0.626 | 115.3 | 0.50 | NaN | NaN | 0.75 | NaN | 0.69 | NaN | NaN | NaN |
| F5H8D7 | DNA repair protein XRCC1 | XRCC1 | 416 | S | 0.845 | 85.73 | 0.52 | NaN | NaN | 0.65 | 0.71 | 0.72 | 0.68 | 0.76 | NaN |

| | | | | | | | | | | | | | | |
|---|---|---|---|---|---|---|---|---|---|---|---|---|---|---|
| H0YAQ0 | Set1/Ash2 histone methyltransferase complex subunit ASH2 | ASH2L | 85 | S | 1 | 53.76 | 0.79 | 0.80 | 0.97 | 0.77 | 0.76 | 0.71 | NaN | 1.02 | 0.97 |
| F6PQP6 | | EPN2 | 192 | S | 1 | 61.73 | 0.51 | 0.68 | NaN | NaN | NaN | NaN | 0.78 | NaN | NaN |
| F6RY50 | Signal-induced proliferation-associated protein 1 | SIPA1 | 737 | S | 0.843 | 78.7 | NaN | NaN | NaN | 0.96 | 0.98 | NaN | NaN | 1.21 | NaN |
| F6VDH7 | Hsc70-interacting protein | ST13 | 38 | S | 1 | 50.24 | 0.56 | NaN | NaN | NaN | 0.77 | NaN | 0.83 | 0.74 | NaN |
| F6VDH7 | Hsc70-interacting protein | ST13 | 39 | S | 1 | 50.24 | 0.56 | NaN | NaN | NaN | 0.77 | NaN | 0.83 | 0.74 | NaN |
| F6VDH7 | Hsc70-interacting protein | ST13 | 42 | S | 1 | 50.24 | 0.56 | NaN | NaN | NaN | 0.77 | NaN | 0.83 | 0.74 | NaN |
| F8VPD4 | CAD protein;Glutamine-dependent carbamoyl-phosphate synthase;Aspartate carbamoyltransferase;Dihydroorotase | CAD | 1796 | S | 1 | 59.92 | 0.86 | 1.17 | 0.86 | 0.97 | 1.03 | NaN | 1.59 | 1.55 | 1.65 |
| H3BV98 | RISC-loading complex subunit TARBP2 | TARBP2 | 53 | S | 1 | 156.5 | 0.91 | 1.12 | 0.79 | NaN | NaN | 0.75 | 0.78 | 0.49 | NaN |
| F8VTQ5 | Heterogeneous nuclear ribonucleoprotein A1;Heterogeneous nuclear ribonucleoprotein A1, N-terminally processed | HNRNPA1 | 6 | S | 1 | 257.2 | 1.48 | 1.42 | 1.32 | 1.46 | 1.42 | 1.43 | 1.11 | 1.14 | 1.16 |
| F8VVL1 | Density-regulated protein | DENR | 73 | S | 1 | 250.5 | 0.63 | 0.61 | 0.62 | 0.91 | 0.90 | 0.97 | 0.91 | 1.02 | 1.04 |
| F8VXC8 | SWI/SNF complex subunit SMARCC2 | SMARCC2 | 347 | S | 1 | 127.3 | 0.79 | 0.76 | 0.70 | 1.17 | 1.12 | 1.02 | 0.77 | 0.67 | 0.79 |
| F8VXC8 | SWI/SNF complex subunit SMARCC2 | SMARCC2 | 302 | S | 1 | 118.7 | 0.87 | 0.87 | 0.88 | 0.89 | 0.93 | 0.92 | 0.95 | 0.97 | 0.98 |
| F8VXC8 | SWI/SNF complex subunit SMARCC2 | SMARCC2 | 304 | S | 1 | 118.7 | 0.85 | 0.86 | 0.88 | 0.93 | 0.93 | 0.92 | 0.94 | 0.97 | 0.97 |
| F8VXC8 | SWI/SNF complex subunit SMARCC2 | SMARCC2 | 306 | S | 0.978 | 72.52 | 1.67 | 1.61 | NaN | NaN | 0.87 | NaN | 0.95 | 1.17 | 0.99 |
| F8VXC8 | SWI/SNF complex subunit SMARCC2 | SMARCC2 | 283 | S | 0.999 | 153 | 0.80 | 0.74 | 0.90 | 1.09 | 1.09 | 1.12 | 1.11 | 1.22 | 1.04 |
| F8VY01 | FYVE, RhoGEF and PH domain-containing protein 6 | FGD6 | 721 | S | 1 | 71.81 | NaN | NaN | NaN | NaN | 0.76 | NaN | 0.88 | 0.67 | NaN |
| F8W038 | Chromatin complexes subunit BAP18 | C17orf49 | 96 | S | 1 | 154.5 | 1.31 | 1.29 | 1.48 | 1.43 | 1.50 | 1.49 | 0.93 | 0.92 | 0.95 |
| F8W038 | Chromatin complexes subunit BAP18 | C17orf49 | 35 | S | 0.5 | 97.12 | 2.62 | 2.36 | NaN | NaN | NaN | NaN | NaN | NaN | NaN |
| F8W038 | Chromatin complexes subunit BAP18 | C17orf49 | 36 | S | 0.5 | 97.12 | 2.62 | 2.36 | NaN | NaN | NaN | NaN | NaN | NaN | NaN |
| F8W726 | Ubiquitin-associated protein 2-like | UBAP2L | 620 | S | 0.885 | 155.9 | 1.07 | 1.17 | 1.25 | 1.35 | 1.30 | 1.23 | 0.85 | 0.88 | 0.92 |
| F8W726 | Ubiquitin-associated protein 2-like | UBAP2L | 464 | S | 0.622 | 168.9 | 3.68 | 3.56 | 4.11 | NaN | NaN | NaN | NaN | NaN | NaN |
| F8W726 | Ubiquitin-associated protein 2-like | UBAP2L | 465 | S | 0.906 | 129.3 | NaN | 4.41 | 3.84 | 3.19 | NaN | 2.97 | 2.56 | 3.39 | 2.39 |

| | | | | | | | | | | | | | | |
|---|---|---|---|---|---|---|---|---|---|---|---|---|---|---|
| F8W726 | Ubiquitin-associated protein 2-like | UBAP2L | 478 | S | 1 | 243.2 | 1.04 | 1.43 | 0.64 | 0.79 | NaN | 0.78 | 0.57 | NaN | 0.63 |
| F8W726 | Ubiquitin-associated protein 2-like | UBAP2L | 488 | S | 0.906 | 155.6 | 1.04 | 1.43 | 1.18 | 1.79 | 1.90 | 1.94 | 0.57 | 0.53 | 0.68 |
| F8W914 | Reticulon | RTN4 | 153 | S | 0.994 | 244.7 | 0.96 | 1.14 | NaN | NaN | 1.28 | NaN | 1.02 | 1.08 | NaN |
| F8W930 | Insulin-like growth factor 2 mRNA-binding protein 2 | IGF2BP2 | 168 | S | 0.984 | 142.4 | 1.36 | 1.39 | 1.30 | 1.39 | 1.47 | 1.23 | NaN | 1.10 | 1.21 |
| F8W930 | Insulin-like growth factor 2 mRNA-binding protein 2 | IGF2BP2 | 170 | S | 0.999 | 149.2 | 0.82 | 0.89 | 1.67 | 0.69 | 0.75 | 2.00 | 1.06 | 1.02 | 0.95 |
| F8W9J4 | Dystonin | DST | 1378 | S | 0.77 | 64.1 | NaN | 0.91 | 0.78 | 1.54 | 1.58 | 1.39 | 0.45 | NaN | 0.52 |
| F8W9J4 | Dystonin | DST | 1382 | S | 0.999 | 81.33 | 0.73 | 0.74 | NaN | 1.53 | 1.58 | 1.46 | 0.49 | 0.55 | 0.50 |
| F8W9J4 | Dystonin | DST | 2919 | S | 1 | 78.43 | 0.92 | 0.85 | NaN | 0.91 | 1.34 | 1.55 | 1.06 | 1.34 | 1.32 |
| F8W9J4 | Dystonin | DST | 2671 | S | 0.997 | 85.88 | NaN | NaN | NaN | NaN | 2.49 | 1.25 | NaN | NaN | NaN |
| F8WA39 | Myotubularin-related protein 1 | MTMR1 | 43 | S | 0.993 | 87.49 | NaN | 0.81 | 1.30 | NaN | 0.93 | NaN | 1.08 | 1.03 | 1.63 |
| F8WAE5 | Eukaryotic translation initiation factor 2A;Eukaryotic translation initiation factor 2A, N-terminally processed | EIF2A | 498 | S | 0.591 | 88.95 | 0.73 | NaN | 0.68 | 0.69 | 0.72 | 0.73 | 0.89 | 0.81 | 0.93 |
| F8WAE5 | Eukaryotic translation initiation factor 2A;Eukaryotic translation initiation factor 2A, N-terminally processed | EIF2A | 501 | S | 1 | 233.5 | 0.77 | 0.66 | 0.76 | 0.72 | 0.66 | 0.73 | 0.96 | 0.96 | 0.88 |
| F8WAZ4 | GTP-binding protein 1 | GTPBP1 | 44 | S | 1 | 79.88 | NaN | 1.10 | 1.30 | 1.43 | 1.25 | 1.42 | 1.35 | 1.71 | 1.88 |
| F8WAZ4 | GTP-binding protein 1 | GTPBP1 | 47 | S | 1 | 79.88 | NaN | 1.10 | 1.30 | 1.43 | 1.25 | 1.42 | 1.35 | 1.71 | 1.88 |
| F8WAZ4 | GTP-binding protein 1 | GTPBP1 | 25 | S | 0.677 | 78.94 | 0.90 | 0.84 | NaN | NaN | NaN | NaN | NaN | NaN | NaN |
| F8WBF9 | Protein NDRG3 | NDRG3 | 266 | S | 1 | 104.2 | NaN | NaN | 1.44 | NaN | NaN | 0.99 | 2.07 | 1.29 | 1.24 |
| F8WBL2 | Bystin | BYSL | 98 | S | 1 | 184.2 | 0.74 | 0.89 | 0.75 | 0.95 | 0.90 | 1.02 | 0.59 | 0.59 | 0.58 |
| F8WCX2 | Zinc finger protein 687 | ZNF687 | 253 | S | 0.96 | 113.7 | 0.65 | NaN | NaN | NaN | 0.55 | 0.73 | 0.80 | 0.79 | NaN |
| F8WCX2 | Zinc finger protein 687 | ZNF687 | 183 | S | 0.685 | 49.91 | 0.67 | 0.94 | NaN | 0.62 | 0.87 | NaN | NaN | NaN | NaN |
| F8WD88 | Histone H3-like centromeric protein A | CENPA | 17 | S | 0.996 | 61.86 | 0.96 | 1.08 | 0.96 | 1.04 | 1.00 | NaN | 0.89 | 1.01 | 0.91 |
| F8WD88 | Histone H3-like centromeric protein A | CENPA | 19 | S | 0.958 | 61.86 | 0.96 | 1.08 | 0.96 | 1.04 | 1.00 | NaN | 0.89 | 1.01 | 0.91 |
| F8WDB4 | Leucine-rich repeat and WD repeat-containing protein 1 | LRWD1 | 99 | S | 0.952 | 125.6 | NaN | 0.92 | 0.89 | NaN | NaN | NaN | 1.30 | NaN | NaN |
| F8WDB4 | Leucine-rich repeat and WD repeat-containing protein 1 | LRWD1 | 60 | S | 1 | 93.8 | 0.60 | 0.66 | 0.69 | NaN | 0.74 | 0.84 | 0.79 | NaN | NaN |
| F8WF93 | Dol-P-Man:Man(5)GlcNAc(2)-PP-Dol alpha-1,3-mannosyltransferase | ALG3 | 13 | S | 0.996 | 86.09 | 2.10 | 2.02 | 2.15 | 1.92 | 2.45 | 2.03 | 1.74 | 1.24 | 1.41 |
| G3V1D1 | Ferritin;Ferritin heavy chain;Ferritin heavy chain, N-terminally processed | FTH1 | 109 | S | 0.997 | 175.1 | 0.69 | 0.67 | 0.61 | 0.79 | 0.98 | 0.88 | 0.40 | 0.58 | NaN |
| G3V198 | Nuclear pore complex protein Nup160 | NUP160 | 1122 | S | 0.999 | 102.4 | 0.57 | 0.66 | 0.60 | 0.65 | 0.70 | 0.67 | 0.85 | 0.90 | 0.90 |
| H0YER7 | Apoptosis inhibitor 5 | API5 | 277 | S | 0.797 | 166 | NaN | NaN | 0.87 | NaN | 1.04 | 1.03 | 1.09 | NaN | NaN |
| H0YER7 | Apoptosis inhibitor 5 | API5 | 279 | S | 0.97 | 225.1 | 0.81 | 0.81 | 0.81 | 1.01 | 0.95 | 0.99 | 0.79 | 0.76 | 0.86 |

| | | | | | | | | | | | | | | |
|---|---|---|---|---|---|---|---|---|---|---|---|---|---|---|
| G3V2D6 | Heterogeneous nuclear ribonucleoproteins C1/C2 | HNRNPC | 207 | S | 1 | 243 | 0.57 | NaN | NaN | NaN | 0.71 | NaN | 0.90 | 0.88 | NaN |
| G3V2R1 | Protein Smaug homolog 1 | SAMD4A | 11 | S | 0.995 | 117.3 | 1.01 | 1.01 | 1.12 | NaN | NaN | NaN | NaN | NaN | NaN |
| G3V340 | Striatin-3 | STRN3 | 257 | S | 1 | 170.3 | 0.70 | 0.81 | 0.57 | 0.98 | 1.07 | 0.84 | 0.67 | 0.78 | 0.61 |
| G3V4X8 | SNW domain-containing protein 1 | SNW1 | 62 | S | 1 | 129.9 | 0.58 | 0.49 | 0.41 | 0.62 | 0.61 | 0.62 | 0.30 | 0.32 | 0.32 |
| G3V4X8 | SNW domain-containing protein 1 | SNW1 | 70 | S | 0.993 | 129.9 | 0.83 | 0.79 | 0.84 | 1.03 | 0.83 | 0.97 | 0.71 | 0.74 | 0.74 |
| G3V4X8 | SNW domain-containing protein 1 | SNW1 | 72 | S | 0.5 | 91.97 | NaN | NaN | NaN | NaN | 0.96 | NaN | 0.67 | 0.60 | NaN |
| Q5TAQ8 | DDB1- and CUL4-associated factor 8 | DCAF8 | 99 | S | 1 | 131 | NaN | NaN | NaN | NaN | NaN | NaN | 1.03 | 1.09 | 1.12 |
| G3V411 | Zinc finger CCCH domain-containing protein 14 | ZC3H14 | 217 | S | 1 | 113.6 | 1.70 | 1.75 | 1.37 | 1.58 | 1.63 | 1.46 | 1.78 | 1.56 | 1.43 |
| H0YJ03 | Proteasome subunit alpha type-3 | PSMA3 | 78 | S | 1 | 227.4 | 0.63 | 0.60 | 0.63 | 0.88 | 1.01 | 0.91 | 0.48 | 0.47 | 0.57 |
| G3V529 | ATP-dependent RNA helicase DDX24 | DDX24 | 39 | S | 1 | 162.8 | 1.00 | 1.28 | 1.29 | 0.79 | 0.86 | 0.84 | 0.77 | 0.80 | 0.67 |
| G3V529 | ATP-dependent RNA helicase DDX24 | DDX24 | 252 | S | 0.677 | 79.02 | 1.05 | NaN | 1.21 | NaN | NaN | 1.14 | NaN | NaN | NaN |
| G3V599 | Melanoma inhibitory activity protein 2 | MIA2 | 1071 | S | 1 | 124.6 | 3.24 | 2.89 | 2.93 | NaN | NaN | 2.47 | NaN | NaN | NaN |
| G3V5E1 | Cyclin-K | CCNK | 340 | S | 1 | 101.5 | 0.99 | 0.79 | 0.81 | 0.88 | 0.83 | 0.82 | 0.88 | 0.95 | 0.88 |
| G3V5E1 | Cyclin-K | CCNK | 324 | S | 1 | 79.47 | 0.92 | 0.81 | 0.82 | 0.88 | 0.78 | 0.92 | 0.92 | 1.01 | 1.09 |
| G3V5E1 | Cyclin-K | CCNK | 328 | S | 0.639 | 79.47 | 0.92 | 0.81 | 0.82 | 0.88 | 0.93 | 0.92 | 1.04 | 1.01 | 1.09 |
| H0YG16 | Kinesin light chain 1 | KLC1 | 152 | S | 0.967 | 77.75 | 1.19 | 1.16 | NaN | 0.89 | 1.17 | NaN | NaN | NaN | 1.04 |
| H0YG16 | Kinesin light chain 1 | KLC1 | 155 | S | 0.883 | 72.89 | NaN | NaN | 1.27 | NaN | 1.17 | 1.11 | 0.93 | 0.93 | NaN |
| H0YG16 | Kinesin light chain 1 | KLC1 | 66 | S | 0.985 | 43.72 | 2.74 | NaN | 2.71 | 5.54 | 4.79 | NaN | 1.59 | 1.05 | NaN |
| G3V5V3 | Nuclear export mediator factor NEMF | NEMF | 705 | S | 1 | 102.7 | 0.46 | 0.57 | NaN | 0.60 | 0.60 | 0.66 | NaN | NaN | 0.78 |
| G3V5V3 | Nuclear export mediator factor NEMF | NEMF | 706 | S | 1 | 102.7 | 0.46 | 0.57 | NaN | 0.60 | 0.60 | 0.66 | NaN | NaN | 0.78 |
| Q5QP22 | RNA-binding protein 39 | RBM39 | 136 | S | 1 | 142.4 | 1.07 | 1.10 | 1.09 | 1.15 | 1.13 | 1.10 | 0.89 | 0.92 | 0.85 |
| Q5QP22 | RNA-binding protein 39 | RBM39 | 97 | S | 0.996 | 70.4 | 1.30 | NaN | 1.99 | NaN | 1.32 | 1.34 | 1.37 | 1.28 | 1.38 |
| G3XAM7 | Catenin alpha-1 | CTNNA1 | 641 | S | 1 | 299.9 | 1.36 | 1.46 | 1.42 | 1.28 | 1.28 | 1.27 | 1.16 | 1.08 | 1.18 |
| P42166 | Lamina-associated polypeptide 2, isoforms beta/gamma;Thymopoietin;Thymopentin;Lamina-associated polypeptide 2, isoform alpha;Thymopoietin;Thymopentin | TMPO | 66;66 | S | 1 | 311.2 | 1.28 | 1.29 | 0.62 | 1.27 | 0.67 | 1.38 | 0.94 | 0.72 | 0.94 |

| | | | | | | | | | | | | | | |
|---|---|---|---|---|---|---|---|---|---|---|---|---|---|---|
| P42166 | Lamina-associated polypeptide 2, isoforms beta/gamma;Thymopoietin;Thymopentin;Lamina-associated polypeptide 2, isoform alpha;Thymopoietin;Thymopentin | TMPO | 67;67 | S | 1 | 312.7 | 0.62 | 0.71 | 1.28 | 0.69 | 0.67 | 0.70 | 0.69 | 0.72 | 0.83 |
| G5E9A6 | Ubiquitin carboxyl-terminal hydrolase;Ubiquitin carboxyl-terminal hydrolase 11 | USP11 | 905 | S | 0.98 | 67.83 | NaN | NaN | 0.33 | NaN | NaN | NaN | 0.75 | 0.96 | NaN |
| G5E9A6 | Ubiquitin carboxyl-terminal hydrolase;Ubiquitin carboxyl-terminal hydrolase 11 | USP11 | 605 | S | 0.997 | 115.3 | 0.49 | NaN | NaN | NaN | NaN | NaN | 1.25 | 1.92 | 0.91 |
| G5E9C0 | Sp110 nuclear body protein | SP110 | 256 | S | 1 | 86.54 | 0.34 | 0.48 | NaN | 0.78 | NaN | NaN | NaN | NaN | NaN |
| G5E9C8 | Son of sevenless homolog 1 | SOS1 | 1195 | S | 0.998 | 56.91 | 1.10 | NaN | NaN | 0.95 | 0.95 | NaN | 0.92 | 0.83 | NaN |
| G8JLD5 | Dynamin-1-like protein | DNM1L | 592 | S | 1 | 73.98 | 0.97 | 0.99 | 1.09 | NaN | 1.65 | 1.50 | 0.75 | 0.77 | 0.66 |
| G8JLH9 | Signal transducer and activator of transcription;Signal transducer and activator of transcription 3 | STAT3 | 629 | S | 1 | 131.9 | 1.73 | 1.73 | 1.63 | 1.83 | 1.90 | 2.01 | 0.88 | 0.81 | NaN |
| H0Y2P0 | CD44 antigen | CD44 | 254 | S | 1 | 108.8 | 1.01 | 1.03 | 0.99 | 0.84 | 0.88 | 0.82 | 1.09 | 1.20 | 1.19 |
| H0Y2V6 | Centrosomal protein of 170 | CEP170 | 935 | S | 0.97 | 97.22 | 3.01 | 3.48 | 3.89 | 1.63 | 1.46 | 3.63 | 2.51 | 1.89 | 2.10 |
| H0Y2V6 | Centrosomal protein of 170 | CEP170 | 410 | S | 0.949 | 78.79 | 3.25 | 1.83 | NaN | NaN | NaN | NaN | NaN | NaN | NaN |
| H0Y2V6 | Centrosomal protein of 170 | CEP170 | 1043 | S | 0.693 | 43.37 | NaN | NaN | NaN | 1.83 | 2.23 | NaN | NaN | NaN | 0.81 |
| H0Y2V6 | Centrosomal protein of 170 kDa;Cep170-like protein | CEP170 | 1495 | S | 0.769 | 97.61 | NaN | NaN | NaN | NaN | 1.14 | NaN | 2.37 | 1.79 | NaN |
| H0Y2V6 | Centrosomal protein of 170 kDa;Cep170-like protein | CEP170 | 1496 | S | 0.505 | 42.33 | NaN | NaN | NaN | NaN | 1.14 | NaN | 2.37 | 1.79 | NaN |
| H0Y2V6 | Centrosomal protein of 170 kDa;Cep170-like protein | CEP170 | 1503 | S | 1 | 124 | 1.11 | 1.07 | 0.74 | 0.88 | 0.87 | 0.64 | 2.37 | 0.98 | 0.92 |
| H0Y589 | Histone lysine demethylase PHF8 | PHF8 | 585 | S | 0.968 | 80.06 | NaN | 1.09 | 0.89 | NaN | NaN | NaN | NaN | 1.95 | NaN |
| H0Y449 | Nuclease-sensitive element-binding protein 1 | YBX1 | 364 | S | 0.886 | 188.1 | 1.19 | 1.31 | 1.33 | 1.32 | 1.17 | 1.31 | 1.16 | 1.24 | 1.18 |
| H0Y449 | Nuclease-sensitive element-binding protein 1 | YBX1 | 215 | S | 0.997 | 273.4 | 3.27 | NaN | NaN | 1.64 | NaN | 1.48 | 2.98 | 2.09 | NaN |
| H0Y449 | Nuclease-sensitive element-binding protein 1 | YBX1 | 224 | S | 0.991 | 315 | 1.58 | 1.59 | NaN | 1.40 | 1.20 | 1.15 | 1.85 | 1.64 | 1.56 |
| H0Y4R1 | Inosine-5'-monophosphate dehydrogenase 2 | IMPDH2 | 372 | S | 1 | 74.39 | 0.92 | NaN | NaN | 1.04 | 1.06 | NaN | NaN | NaN | NaN |
| H0Y4V9 | La-related protein 4B | LARP4B | 165 | S | 0.827 | 89.68 | NaN | 1.18 | NaN | 1.35 | 1.42 | 1.21 | NaN | 0.99 | 1.21 |
| H0Y4V9 | La-related protein 4B | LARP4B | 167 | S | 0.997 | 104.5 | 0.92 | 0.59 | 1.60 | 1.26 | 1.18 | 1.34 | NaN | 1.12 | 0.52 |
| H0Y4V9 | La-related protein 4B | LARP4B | 337 | S | 1 | 137.9 | 1.62 | 1.56 | 1.77 | 1.43 | 1.59 | 1.62 | 0.87 | 0.79 | 0.91 |
| H0Y4V9 | La-related protein 4B | LARP4B | 82 | S | 0.557 | 59.3 | NaN | NaN | NaN | NaN | NaN | 0.74 | 1.09 | 1.45 | NaN |
| H0Y4V9 | La-related protein 4B | LARP4B | 90 | S | 0.899 | 143.5 | 0.97 | 1.07 | 1.22 | 1.03 | 1.26 | 1.15 | 1.50 | 1.11 | 1.68 |
| H0Y4Z8 | Rab-like protein 6 | RABL6 | 426 | S | 1 | 93.1 | NaN | NaN | NaN | NaN | NaN | NaN | NaN | 0.65 | 0.95 |
| H0Y4Z8 | Rab-like protein 6 | RABL6 | 597 | S | 1 | 150.5 | 1.19 | 1.15 | 1.46 | 1.34 | 1.06 | 1.09 | 1.17 | 1.36 | 1.28 |

| | | | | | | | | | | | | | | | |
|---|---|---|---|---|---|---|---|---|---|---|---|---|---|---|---|
| H0Y4Z8 | Rab-like protein 6 | RABL6 | 455 | S | 1 | 74.84 | 1.51 | 0.54 | 1.09 | 0.65 | 0.89 | 1.10 | 1.18 | 0.80 | 1.47 |
| H0Y4Z8 | Rab-like protein 6 | RABL6 | 465 | S | 0.981 | 60.62 | 1.51 | 0.54 | NaN | 0.65 | 0.89 | 1.58 | NaN | NaN | 1.23 |
| H0Y4Z8 | Rab-like protein 6 | RABL6 | 471 | S | 0.926 | 74.84 | 1.51 | NaN | 1.09 | NaN | 1.35 | 1.10 | 1.27 | 0.49 | 1.09 |
| H0Y4Z8 | Rab-like protein 6 | RABL6 | 472 | S | 0.943 | 74.84 | 1.51 | NaN | 1.09 | NaN | 1.35 | 1.32 | 1.18 | 0.49 | 1.09 |
| H0Y503 | Basic leucine zipper and W2 domain-containing protein 1 | BZW1 | 127 | S | 1 | 173.1 | NaN | 0.72 | 0.77 | 0.81 | NaN | 0.71 | 0.68 | 0.58 | 0.63 |
| H0Y507 | SH3 and PX domain-containing protein 2A | SH3PXD2A | 474 | S | 1 | 211.1 | 0.76 | NaN | NaN | 0.94 | 0.83 | 0.97 | NaN | NaN | 0.81 |
| H0Y555 | Proteasome inhibitor PI31 subunit | PSMF1 | 13 | S | 0.757 | 72.92 | NaN | 1.12 | NaN | 1.27 | 1.18 | NaN | NaN | NaN | NaN |
| H0Y564 | Anaphase-promoting complex subunit 1 | ANAPC1 | 221 | S | 0.5 | 48.44 | NaN | NaN | NaN | NaN | NaN | NaN | 0.80 | 0.95 | NaN |
| H0Y564 | Anaphase-promoting complex subunit 1 | ANAPC1 | 223 | S | 0.994 | 83.09 | 0.77 | NaN | NaN | NaN | NaN | 1.07 | 0.74 | 0.83 | NaN |
| H0Y579 | UV excision repair protein RAD23 homolog B | RAD23B | 30 | S | 0.954 | 251.7 | 1.41 | 1.46 | 1.34 | 1.58 | 1.61 | 1.65 | 0.85 | 0.91 | 0.92 |
| H0Y7H7 | Dedicator of cytokinesis protein 4 | DOCK4 | 1161 | S | 1 | 75.2 | 1.42 | 1.59 | NaN | 0.89 | NaN | NaN | 1.13 | 1.29 | NaN |
| H0Y5S9 | Casein kinase I isoform | CSNK1E | 66 | S | 0.5 | 88.16 | 0.79 | NaN | NaN | 0.97 | 0.79 | NaN | 1.06 | 1.00 | 1.01 |
| H0Y626 | Tripartite motif-containing protein 16 | TRIM16 | 60 | S | 1 | 314.3 | 0.93 | 0.89 | 0.92 | 1.09 | 1.01 | 1.21 | 0.79 | 0.78 | 0.92 |
| H0Y6F5 | Zinc fingers and homeoboxes protein 3 | ZHX3 | 655 | S | 0.968 | 52.11 | 1.05 | 1.06 | NaN | NaN | NaN | NaN | 0.97 | 1.15 | NaN |
| H7BZT5 | Zinc finger protein 185 | ZNF185 | 205;449 | S | 0.656 | 158.5 | NaN | NaN | NaN | NaN | 0.90 | NaN | NaN | 1.33 | 1.24 |
| H7BZT5 | Zinc finger protein 185 | ZNF185 | 212;456 | S | 1 | 138 | 1.12 | 1.21 | 1.16 | 1.20 | 1.05 | 0.97 | 1.20 | 1.18 | 1.13 |
| H7BZT5 | Zinc finger protein 185 | ZNF185 | 278;522 | S | 0.995 | 199.6 | NaN | 0.71 | 0.89 | NaN | 0.94 | 1.26 | 0.78 | NaN | 1.51 |
| H7BZT5 | Zinc finger protein 185 | ZNF185 | 224;468 | S | 0.996 | 113.2 | 2.53 | 2.78 | 3.18 | NaN | NaN | 2.66 | 4.37 | 5.96 | 3.81 |
| H0Y704 | | ZNF185 | 23 | S | 1 | 160.5 | NaN | 0.84 | 0.93 | 0.62 | 0.59 | 0.62 | 0.67 | NaN | NaN |
| H0Y7Q2 | Coiled-coil domain-containing protein 132 | CCDC132 | 346 | S | 0.998 | 150.1 | 0.82 | 0.74 | NaN | 0.91 | NaN | NaN | NaN | NaN | 0.78 |
| H0Y7W6 | | FNBP1 | 311 | S | 0.997 | 57.35 | 1.10 | 0.93 | NaN | 1.02 | 0.87 | 1.05 | 1.20 | 1.17 | 1.27 |
| Q96RU3 | Formin-binding protein 1 | FNBP1 | 258;296 | S | 0.998 | 85.4 | 1.74 | 1.77 | 1.70 | 1.68 | 1.85 | 1.87 | 2.48 | 2.30 | 2.32 |
| H0Y7Y8 | AT-rich interactive domain-containing protein 4B | ARID4B | 75 | S | 0.976 | 61.04 | NaN | 0.30 | NaN | 0.68 | 0.68 | NaN | NaN | 0.43 | NaN |
| H0Y7Z1 | Fibronectin;Anastellin;Ugl-Y1;Ugl-Y2;Ugl-Y3 | FN1 | 1101 | S | 1 | 188.5 | NaN | 1.55 | 1.57 | NaN | NaN | 1.21 | NaN | 1.05 | NaN |
| H0Y8X4 | 2'-deoxynucleoside 5'-phosphate N-hydrolase 1 | DNPH1 | 238 | S | 0.998 | 140.7 | NaN | 1.25 | 1.25 | 1.76 | 1.80 | 1.35 | 1.10 | 1.00 | 1.05 |
| H0Y9P1 | BMP-2-inducible protein | BMP2K | 722 | S | 0.842 | 40.01 | 1.53 | 1.26 | NaN | NaN | NaN | NaN | 1.00 | NaN | NaN |
| H0Y9P1 | BMP-2-inducible protein | BMP2K | 724 | S | 0.856 | 59.51 | 1.53 | 1.26 | NaN | NaN | NaN | NaN | 1.00 | 1.06 | NaN |
| H0Y9P1 | BMP-2-inducible protein | BMP2K | 725 | S | 0.856 | 59.51 | 1.53 | 1.26 | NaN | NaN | NaN | NaN | 1.00 | 1.06 | NaN |
| H0Y9P1 | BMP-2-inducible protein | BMP2K | 640 | S | 0.645 | 99.96 | 1.55 | 1.69 | 1.92 | NaN | NaN | NaN | 1.80 | 1.98 | 2.64 |
| H0Y9T5 | m7GpppN-mRNA hydrolase | DCP2 | 228 | S | 1 | 80.85 | 1.07 | 0.89 | 1.24 | 1.09 | 0.90 | 1.07 | 0.96 | 0.80 | 1.15 |
| H0Y9T5 | m7GpppN-mRNA hydrolase | DCP2 | 229 | S | 1 | 80.85 | 1.07 | 0.89 | 1.24 | 1.09 | 0.90 | 1.07 | 0.96 | 0.80 | 1.15 |
| H0Y9T5 | m7GpppN-mRNA hydrolase | DCP2 | 231 | S | 1 | 80.85 | 1.07 | 0.89 | 1.24 | 1.09 | 0.90 | 1.07 | 0.96 | 0.80 | 1.15 |
| H0YA93 | NEDD4-binding protein 2 | N4BP2 | 313 | S | 0.542 | 42.35 | 1.18 | 0.90 | 1.96 | 1.40 | 1.88 | 0.83 | 1.45 | NaN | NaN |
| H0YA93 | NEDD4-binding protein 2 | N4BP2 | 333 | S | 0.826 | 42.35 | 1.18 | 0.90 | 1.96 | 1.40 | 1.88 | 0.83 | 1.45 | NaN | NaN |

| | | | | | | | | | | | | | | |
|---|---|---|---|---|---|---|---|---|---|---|---|---|---|---|
| H0YA93 | NEDD4-binding protein 2 | N4BP2 | 335 | S | 0.826 | 42.35 | 1.18 | 0.90 | 1.96 | 1.40 | 1.88 | 0.83 | 1.45 | NaN | NaN |
| H0YA93 | NEDD4-binding protein 2 | N4BP2 | 337 | S | 0.826 | 42.35 | 1.18 | 0.90 | 1.96 | 1.40 | 1.88 | 0.83 | 1.45 | NaN | NaN |
| H0YAC7 | Coatomer subunit beta' | COPB2 | 73 | S | 1 | 351.3 | 0.88 | 0.86 | 0.85 | 0.86 | 0.97 | 0.91 | 0.80 | 0.88 | 0.97 |
| H0YAD9 | SH3 domain-binding protein 2 | SH3BP2 | 120 | S | 1 | 41.54 | 1.26 | NaN | 1.12 | NaN | 0.89 | 1.10 | NaN | NaN | NaN |
| H0YAZ3 | Vinexin | SORBS3 | 42 | S | 0.975 | 184.9 | NaN | NaN | 1.91 | 1.70 | 1.72 | 1.77 | 2.10 | 2.22 | 2.38 |
| H0YD46 | DNA polymerase delta subunit 3 | POLD3 | 31 | S | 1 | 164.5 | 0.51 | 0.56 | NaN | 0.68 | 0.71 | NaN | 0.51 | 0.59 | NaN |
| H0YDM2 | Protein SOGA1;N-terminal form;C-terminal 80 kDa form | SOGA1 | 869 | S | 1 | 60.34 | NaN | 0.40 | NaN | 0.67 | 0.70 | 0.57 | NaN | 0.87 | NaN |
| H0YE29 | Rho GTPase-activating protein 1 | ARHGAP 1 | 48 | S | 0.73 | 80.91 | NaN | NaN | NaN | 1.38 | 1.42 | 1.32 | 1.25 | NaN | NaN |
| H0YE29 | Rho GTPase-activating protein 1 | ARHGAP 1 | 49 | S | 0.981 | 169.2 | 1.63 | 1.64 | 1.80 | 1.44 | NaN | 1.38 | 1.20 | 1.20 | 1.24 |
| H0YE35 | Synembryn-A | RIC8A | 44 | S | 1 | 290.8 | 0.63 | 0.64 | 0.60 | 0.76 | 0.76 | 0.76 | 0.43 | 0.51 | 0.53 |
| H0YE35 | Synembryn-A | RIC8A | 118 | S | 1 | 51.57 | NaN | 1.75 | 1.78 | 2.89 | NaN | NaN | 1.02 | 1.01 | NaN |
| H0YGG4 | | EIF2B1 | 7 | S | 1 | 47.81 | NaN | NaN | NaN | NaN | NaN | NaN | 0.55 | 0.38 | NaN |
| H0YGI8 | Stress-induced-phosphoprotein 1 | STIP1 | 80 | S | 1 | 131.1 | 1.60 | 1.59 | 1.45 | 2.64 | 2.61 | 2.43 | 1.02 | 0.99 | 1.02 |
| H0YK04 | Rho GTPase-activating protein 5 | ARHGAP 5 | 96 | S | 0.965 | 186.1 | 1.73 | 1.15 | NaN | 1.84 | NaN | NaN | 1.68 | 1.82 | 1.88 |
| J3KPH4 | Sorting nexin-1 | SNX1 | 32 | S | 1 | 161.4 | 1.04 | 1.13 | 1.02 | 1.09 | 1.09 | 1.04 | 1.13 | 1.80 | 1.17 |
| J3KPH4 | Sorting nexin-1 | SNX1 | 39 | S | 1 | 198.4 | 1.04 | 1.12 | 1.02 | 1.05 | 1.09 | 1.04 | 0.82 | 0.69 | 1.10 |
| H0YLQ7 | DnaJ homolog subfamily C member 17 | DNAJC17 | 112 | S | 1 | 218.4 | 0.96 | 0.89 | 0.94 | 0.86 | 0.87 | NaN | 0.81 | 1.00 | 1.04 |
| H0YM23 | Ankyrin repeat domain-containing protein 17 | ANKRD17 | 1931 | S | 0.982 | 59.98 | NaN | 0.72 | NaN | 0.70 | 0.79 | 0.87 | NaN | 0.98 | NaN |
| H0YNU8 | Tropomodulin-3 | TMOD3 | 25 | S | 0.972 | 91.11 | NaN | 2.15 | NaN | 2.26 | 2.15 | NaN | 1.87 | 1.40 | NaN |
| H3BLV9 | SRSF protein kinase 1 | SRPK1 | 327 | S | 0.96 | 85.93 | 0.99 | 0.84 | NaN | NaN | NaN | NaN | 0.94 | 0.97 | 0.95 |
| H3BLZ2 | | DBNDD1 | 7 | S | 0.939 | 81.01 | 1.20 | 1.14 | 1.11 | 1.04 | 1.07 | 1.08 | 1.03 | 1.03 | 1.09 |
| H3BM18 | Gamma-interferon-inducible protein 16 | IFI16 | 106 | S | 1 | 238.8 | NaN | 1.47 | 1.16 | 2.15 | 2.07 | 2.16 | 1.20 | 1.16 | 1.33 |
| H3BM18 | Gamma-interferon-inducible protein 16 | IFI16 | 153 | S | 1 | 183.7 | 0.60 | 0.58 | 0.64 | 0.88 | 0.80 | 0.91 | NaN | NaN | 0.55 |
| H3BM38 | Synaptosomal-associated protein;Synaptosomal-associated protein 23 | SNAP23 | 110 | S | 1 | 257.8 | 2.61 | 3.01 | 2.90 | 2.66 | NaN | 1.78 | 2.20 | 2.07 | 1.78 |
| H3BM89 | 60S ribosomal protein L4 | RPL4 | 201 | S | 1 | 71.22 | 1.25 | 1.42 | NaN | NaN | 1.42 | 1.22 | 0.87 | 0.84 | 1.09 |
| H3BTD3 | cAMP-regulated phosphoprotein 19 | ARPP19 | 7 | S | 0.765 | 43.3 | 0.73 | 0.67 | NaN | NaN | NaN | NaN | NaN | NaN | NaN |
| H3BMF6 | Ubiquitin carboxyl-terminal hydrolase 7 | USP7 | 18 | S | 1 | 118.7 | 0.90 | NaN | 0.77 | NaN | 1.02 | 1.04 | NaN | NaN | NaN |
| H3BMI8 | Telomeric repeat-binding factor 2-interacting protein 1 | TERF2IP | 32 | S | 0.966 | 183.3 | NaN | NaN | NaN | 2.02 | NaN | NaN | 1.15 | 1.08 | NaN |
| H3BUL0 | RNA-binding protein with serine-rich domain 1 | RNPS1 | 155 | S | 1 | 61.17 | 0.80 | 0.84 | NaN | 0.90 | 0.89 | 0.91 | 0.77 | 0.86 | 0.89 |
| H3BUL0 | RNA-binding protein with serine-rich domain 1 | RNPS1 | 157 | S | 0.996 | 61.17 | 0.80 | 0.84 | NaN | 0.90 | 0.89 | 0.91 | 0.77 | 0.86 | 0.89 |

| | | | | | | | | | | | | | | |
|---|---|---|---|---|---|---|---|---|---|---|---|---|---|---|
| H3BN50 | | SLC9A3R2 | 20 | S | 1 | 67.87 | 1.61 | 1.65 | 1.64 | 1.34 | 1.39 | 1.39 | 1.40 | 1.34 | 1.46 |
| H3BTK3 | Calcium-regulated heat stable protein 1 | CARHSP1 | 30 | S | 1 | 154.1 | 0.77 | 0.81 | 0.60 | 0.85 | 0.67 | 0.63 | 0.99 | 0.81 | 0.77 |
| H3BTK3 | Calcium-regulated heat stable protein 1 | CARHSP1 | 32 | S | 1 | 154.1 | 0.91 | 0.95 | 0.71 | 0.88 | 0.67 | 0.92 | 1.09 | 0.81 | 1.19 |
| H3BTK3 | Calcium-regulated heat stable protein 1 | CARHSP1 | 41 | S | 0.999 | 119.4 | 2.33 | 2.19 | 2.12 | 2.08 | 2.12 | 2.05 | 2.47 | 2.69 | 2.21 |
| H3BNW0 | THUMP domain-containing protein 1 | THUMPD1 | 86 | S | 1 | 147.7 | 0.56 | 0.89 | 0.87 | 0.90 | 0.95 | 0.92 | 0.98 | 0.99 | 0.90 |
| H3BNW0 | THUMP domain-containing protein 1 | THUMPD1 | 88 | S | 1 | 147.7 | 0.81 | 0.89 | 0.49 | 0.90 | 0.95 | 0.92 | 0.65 | 0.96 | 0.90 |
| H3BPG4 | Cytoplasmic tRNA 2-thiolation protein 2 | CTU2 | 101 | S | 1 | 84.82 | 1.15 | 1.08 | 1.15 | 0.85 | 1.12 | 0.89 | 1.10 | 1.18 | 1.22 |
| H3BUH7 | Fructose-bisphosphate aldolase;Fructose-bisphosphate aldolase A | ALDOA | 36 | S | 0.921 | 127.7 | 1.12 | 1.46 | NaN | NaN | NaN | NaN | 1.94 | NaN | NaN |
| H3BTD6 | Enhancer of mRNA-decapping protein 3 | EDC3 | 18 | S | 1 | 173 | 1.14 | 1.23 | 1.06 | 1.39 | 1.16 | 1.24 | 1.26 | 1.06 | 1.33 |
| H3BPZ1 | Very-long-chain (3R)-3-hydroxyacyl-CoA dehydratase | HACD3 | 89 | S | 1 | 123 | 1.35 | 1.59 | 1.43 | 1.36 | 1.19 | 1.35 | 0.79 | 0.79 | 0.62 |
| H3BRT8 | Dual specificity protein kinase CLK3 | CLK3 | 76 | S | 0.958 | 57.07 | 0.69 | 0.64 | NaN | 0.65 | 0.98 | NaN | NaN | 0.93 | NaN |
| H3BRT8 | Dual specificity protein kinase CLK3 | CLK3 | 9 | S | 0.975 | 55.84 | 1.13 | 1.13 | NaN | NaN | NaN | NaN | 1.14 | NaN | NaN |
| H3BQK9 | Microtubule-actin cross-linking factor 1, isoforms 1/2/3/5 | MACF1 | 4553 | S | 1 | 157.4 | 0.63 | 0.68 | 0.69 | 1.24 | 0.98 | 1.03 | 0.98 | 0.92 | 0.91 |
| H3BQK9 | Microtubule-actin cross-linking factor 1, isoforms 1/2/3/5 | MACF1 | 4527 | S | 0.564 | 108.5 | NaN | NaN | NaN | NaN | NaN | NaN | 0.49 | NaN | 0.42 |
| H3BQK9 | Microtubule-actin cross-linking factor 1, isoforms 1/2/3/5 | MACF1 | 4528 | S | 0.995 | 195.4 | 0.38 | NaN | NaN | 0.83 | 0.86 | 0.68 | 0.36 | 0.35 | NaN |
| H3BQZ7 | Heterogeneous nuclear ribonucleoprotein U-like protein 2 | HNRNPUL2-BSCL2 | 161 | S | 1 | 304.8 | 0.98 | 0.92 | 0.92 | 0.99 | 0.93 | 0.97 | 1.09 | 0.98 | 1.10 |
| H3BQZ7 | Heterogeneous nuclear ribonucleoprotein U-like protein 2 | HNRNPUL2-BSCL2 | 226 | S | 0.696 | 220.2 | 0.45 | 0.45 | NaN | 0.56 | 0.63 | NaN | NaN | 0.71 | 0.55 |
| H3BQZ7 | Heterogeneous nuclear ribonucleoprotein U-like protein 2 | HNRNPUL2-BSCL2 | 228 | S | 0.832 | 200.5 | 0.45 | 0.45 | NaN | NaN | NaN | NaN | 0.67 | NaN | NaN |
| H3BRM1 | Abscission/NoCut checkpoint regulator | ZFYVE19 | 262 | S | 1 | 213 | 0.93 | 1.05 | 0.84 | 0.97 | 0.88 | 0.80 | 1.09 | 1.22 | 0.99 |
| H3BRM1 | Abscission/NoCut checkpoint regulator | ZFYVE19 | 115 | S | 1 | 74.46 | 0.83 | 0.69 | 0.82 | NaN | 0.79 | 0.76 | 0.87 | 0.97 | 1.11 |
| H3BRG5 | StAR-related lipid transfer protein 13 | STARD13 | 299 | S | 1 | 43.52 | NaN | 0.84 | 1.17 | NaN | NaN | NaN | NaN | NaN | NaN |

| | | | | | | | | | | | | | | | | |
|---|---|---|---|---|---|---|---|---|---|---|---|---|---|---|---|---|
| H3BRG5 | StAR-related lipid transfer protein 13 | STARD13 | 118 | S | 1 | 109.1 | 1.25 | 1.07 | 1.04 | 0.93 | 0.89 | 0.93 | 1.00 | 1.17 | NaN |
| H3BS42 | Zinc finger protein 768 | ZNF768 | 52 | S | 0.782 | 42 | NaN | NaN | NaN | NaN | 0.84 | NaN | 1.05 | 0.90 | 1.04 |
| H3BS86 | E3 ubiquitin-protein ligase | STUB1 | 19 | S | 1 | 183 | 0.68 | 0.72 | 0.68 | 0.71 | 0.81 | 0.78 | 0.77 | 0.79 | 0.83 |
| H3BS86 | E3 ubiquitin-protein ligase | STUB1 | 23 | S | 0.981 | 81.32 | 0.90 | 1.29 | 1.52 | 1.24 | NaN | 1.59 | 1.03 | NaN | NaN |
| H3BTX0 | PAXIP1-associated glutamate-rich protein 1 | PAGR1 | 67 | S | 1 | 194.9 | 0.96 | 1.16 | 1.29 | 1.09 | 1.20 | NaN | 1.24 | 1.20 | 1.48 |
| J3QLT7 | Nucleolar protein 3 | NOL3 | 70 | S | 0.572 | 99.89 | NaN | 0.80 | 0.85 | NaN | NaN | NaN | 0.83 | NaN | NaN |
| H3BUV9 | Ubiquitin-like protein 7 | UBL7 | 174 | S | 1 | 260.8 | 0.80 | 0.97 | NaN | 0.83 | NaN | 0.10 | 0.95 | 0.88 | NaN |
| P29590 | Protein PML | PML | 162;518 | S | 1 | 154.8 | 0.50 | 0.46 | 0.57 | 0.61 | 0.51 | 0.51 | 0.74 | 0.82 | 0.92 |
| P29590 | Protein PML | PML | 171;527 | S | 1 | 154.8 | 0.63 | 0.54 | 0.82 | 0.69 | 0.79 | 0.82 | 1.07 | 1.25 | 1.44 |
| P29590 | Protein PML | PML | 174;530 | S | 1 | 169.7 | 1.47 | NaN | 1.41 | NaN | 1.16 | 1.23 | 1.63 | NaN | 1.64 |
| P29590 | Protein PML | PML | 47;403 | S | 0.999 | 88.67 | 0.94 | 1.04 | 1.03 | 1.60 | NaN | 1.28 | 1.25 | 1.15 | 1.05 |
| P29590 | Protein PML | PML | 149;505 | S | 0.987 | 116.9 | 0.82 | 0.85 | 0.88 | 0.87 | 0.90 | 0.89 | 1.22 | 1.16 | 1.20 |
| H7BZX1 | Sorbin and SH3 domain-containing protein 2 | SORBS2 | 224 | S | 0.989 | 71.14 | 0.37 | 0.36 | 0.52 | 0.61 | NaN | NaN | NaN | NaN | NaN |
| H7BZ88 | Signal transducer and activator of transcription 1- | STAT1 | 17 | S | 1 | 96.05 | 1.34 | 1.34 | 1.48 | 1.96 | 1.96 | 1.63 | 0.67 | 0.69 | NaN |
| H7BZT5 | Zinc finger protein 185 | ZNF185 | 153 | S | 0.77 | 138.1 | 1.69 | NaN | NaN | NaN | NaN | NaN | 1.79 | 1.85 | NaN |
| H7BZZ1 | HCLS1-binding protein 3 | HS1BP3 | 13 | S | 1 | 190.7 | 0.80 | 0.87 | 1.20 | 1.25 | 1.38 | 0.81 | 1.03 | 0.99 | 1.04 |
| H7BZZ1 | HCLS1-binding protein 3 | HS1BP3 | 68 | S | 1 | 115 | 0.72 | 0.85 | 0.76 | 0.85 | 0.91 | 0.71 | 0.81 | 0.75 | 0.81 |
| H7BZZ6 | Serine/arginine-rich splicing factor 7 | SRSF7 | 103 | S | 1 | 81.88 | 0.95 | 0.98 | 0.99 | 1.04 | 1.05 | 1.10 | NaN | 0.84 | 0.87 |
| H7BZZ6 | Serine/arginine-rich splicing factor 7 | SRSF7 | 105 | S | 1 | 81.88 | 0.26 | 0.25 | 0.27 | 0.39 | 0.40 | 0.38 | NaN | 0.17 | 0.87 |
| H7BZZ6 | Serine/arginine-rich splicing factor 7 | SRSF7 | 76 | S | 0.994 | 97.74 | 1.03 | 1.17 | 0.31 | 0.28 | 1.00 | 1.12 | 0.29 | 1.03 | 0.30 |
| H7BZZ6 | Serine/arginine-rich splicing factor 7 | SRSF7 | 78 | S | 0.993 | 66.96 | 1.03 | 1.17 | 1.07 | 1.06 | 1.00 | 1.12 | 1.11 | 0.30 | 1.14 |
| H7C072 | THO complex subunit 5 homolog | THOC5 | 183 | S | 1 | 187.3 | 0.86 | 0.77 | 0.76 | 0.91 | 0.76 | 0.81 | 1.23 | 0.82 | 1.03 |
| H7C072 | THO complex subunit 5 homolog | THOC5 | 185 | S | 1 | 187.3 | 0.86 | 0.77 | 0.76 | 0.91 | 0.76 | 0.81 | 1.23 | 0.82 | 1.03 |
| H7C0X8 | Macrophage-capping protein | CAPG | 92 | S | 0.999 | 55.16 | 0.93 | 1.26 | NaN | 2.28 | NaN | NaN | 0.68 | NaN | NaN |
| H7C113 | Phospholipid-transporting ATPase IG | ATP11C | 159 | S | 0.845 | 56.21 | 1.20 | 1.63 | NaN | NaN | NaN | NaN | 0.92 | 0.87 | NaN |
| H7C1Q8 | DIS3-like exonuclease 2 | DIS3L2 | 156 | S | 1 | 116.8 | 0.86 | NaN | 0.75 | 0.78 | NaN | 0.66 | NaN | NaN | 0.89 |
| H7C2H2 | Ras-specific guanine nucleotide-releasing factor RalGPS2 | RALGPS2 | 67 | S | 1 | 115 | 0.69 | 0.58 | 0.68 | 0.95 | 1.13 | NaN | 0.95 | 0.86 | NaN |
| J3KQW3 | Transmembrane protein 44 | TMEM44 | 194 | S | 0.994 | 60.02 | 0.85 | 0.83 | NaN | 1.03 | 0.99 | 0.98 | 0.93 | 0.96 | 1.17 |
| H7C409 | Bromodomain and WD repeat-containing protein 1 | BRWD1 | 413 | S | 0.813 | 47.6 | NaN | NaN | NaN | 0.89 | NaN | 0.68 | 1.14 | NaN | NaN |
| H7C4B6 | Transcription regulator protein BACH1 | BACH1 | 11 | S | 0.981 | 47.56 | NaN | NaN | 1.53 | NaN | 1.65 | 1.56 | NaN | NaN | NaN |

| | | | | | | | | | | | | | | |
|---|---|---|---|---|---|---|---|---|---|---|---|---|---|---|
| I1E4Y6 | PERQ amino acid-rich with GYF domain-containing protein 2 | GIGYF2 | 26 | S | 0.966 | 146.9 | 1.85 | 1.69 | 1.62 | 1.69 | 2.08 | 1.87 | 1.40 | 1.44 | 1.67 |
| I1E4Y6 | PERQ amino acid-rich with GYF domain-containing protein 2 | GIGYF2 | 258 | S | 1 | 55.98 | 1.27 | 1.21 | NaN | 1.08 | 1.26 | NaN | NaN | NaN | NaN |
| I3L0L0 | | RUNX2 | 419 | S | 0.682 | 141.5 | NaN | NaN | NaN | 1.00 | NaN | NaN | 0.82 | 0.83 | NaN |
| I3L4F6 | Coiled-coil domain-containing protein 137 | CCDC137 | 18 | S | 1 | 69.28 | NaN | 1.44 | 1.51 | NaN | NaN | NaN | 0.99 | NaN | NaN |
| I3L467 | Unconventional prefoldin RPB5 interactor 1 | URI1 | 16 | S | 0.842 | 46.98 | NaN | NaN | NaN | 0.89 | NaN | NaN | 0.60 | 0.82 | NaN |
| I3L1Q2 | B-cell CLL/lymphoma 7 protein family member C | BCL7C | 114 | S | 0.985 | 84.66 | 0.82 | 1.07 | 1.18 | 0.71 | 0.81 | NaN | NaN | NaN | NaN |
| I3L1Q2 | B-cell CLL/lymphoma 7 protein family member C | BCL7C | 122 | S | 0.561 | 98.74 | 0.94 | NaN | 1.30 | NaN | NaN | NaN | NaN | NaN | NaN |
| I3L1Q2 | B-cell CLL/lymphoma 7 protein family member C | BCL7C | 126 | S | 0.993 | 172.2 | 0.82 | 1.18 | 1.18 | 1.63 | 0.81 | NaN | 1.10 | 1.05 | 0.80 |
| I3L4I0 | FLYWCH family member 2 | FLYWCH 2 | 21 | S | 0.985 | 56.3 | 1.18 | NaN | 0.72 | NaN | 1.58 | 1.32 | NaN | NaN | 0.98 |
| I3L436 | Regulatory-associated protein of mTOR | RPTOR | 37 | S | 0.984 | 106.8 | 0.57 | 0.52 | 0.61 | NaN | NaN | 0.62 | NaN | NaN | 0.83 |
| I3L436 | Regulatory-associated protein of mTOR | RPTOR | 23 | S | 0.998 | 254.8 | 0.69 | 0.62 | 0.70 | 0.69 | 0.70 | 0.71 | 0.88 | 1.05 | 1.21 |
| I3L4J1 | Vacuolar protein sorting-associated protein 4A | VPS4A | 119 | S | 0.839 | 53.2 | NaN | NaN | 0.96 | 0.97 | 0.85 | 0.84 | 0.94 | 1.11 | 1.09 |
| I3L4J1 | Vacuolar protein sorting-associated protein 4A | VPS4A | 121 | S | 0.97 | 53.2 | NaN | NaN | 0.96 | 0.97 | 0.85 | 0.84 | 0.94 | 1.11 | 1.09 |
| J3KNG6 | Protein spire homolog 1 | SPIRE1 | 467 | S | 0.936 | 63.71 | NaN | NaN | 0.87 | NaN | NaN | 1.07 | 1.03 | 0.97 | NaN |
| J3KNN7 | BRCA1-associated protein | BRAP | 87 | S | 0.994 | 151.4 | 0.88 | NaN | 1.04 | 1.12 | 1.03 | 0.95 | 0.96 | 1.01 | 0.93 |
| J3KR72 | Transcription initiation factor TFIID subunit 6 | TAF6 | 710 | S | 1 | 99.71 | NaN | NaN | NaN | NaN | 0.93 | NaN | 0.94 | 0.77 | NaN |
| J3KRY8 | Tether containing UBX domain for GLUT4 | ASPSCR1 | 331 | S | 0.727 | 71.55 | NaN | NaN | NaN | 3.30 | 2.26 | NaN | NaN | NaN | NaN |
| J3KRY8 | Tether containing UBX domain for GLUT4 | ASPSCR1 | 104 | S | 0.933 | 65.41 | 1.50 | 1.50 | 1.69 | 2.66 | 1.97 | 2.65 | 0.92 | 0.92 | NaN |
| J3QLL0 | Importin subunit alpha-1 | KPNA2 | 62 | S | 0.966 | 214.5 | 2.32 | 2.27 | 2.46 | 3.99 | 3.97 | 4.44 | 0.84 | 0.93 | NaN |
| J3KSG3 | ADP-ribosylation factor-binding protein GGA3 | GGA3 | 37 | S | 1 | 77.19 | 1.29 | 1.05 | 1.29 | 0.91 | 1.08 | 1.29 | 0.90 | 1.09 | 1.04 |
| J3KSI3 | E3 ubiquitin-protein ligase rififylin | RFFL | 89 | S | 0.967 | 83.22 | 1.23 | NaN | NaN | NaN | 0.89 | NaN | 1.23 | NaN | 1.24 |
| J3KSI3 | E3 ubiquitin-protein ligase rififylin | RFFL | 92 | S | 0.942 | 83.22 | 1.23 | NaN | NaN | NaN | NaN | NaN | 1.23 | NaN | 1.24 |
| J3KSR8 | Serine/arginine-rich splicing factor 1 | SRSF1 | 94 | S | 1 | 158.5 | 0.75 | 0.76 | 0.78 | 0.75 | 0.75 | 0.75 | 0.70 | 0.67 | 0.75 |
| J3KSR8 | Serine/arginine-rich splicing factor 1 | SRSF1 | 96 | S | 0.983 | 65.63 | 1.26 | 1.38 | 1.27 | 1.48 | 1.34 | 1.27 | 1.45 | 1.29 | 1.54 |

| | | | | | | | | | | | | | | | |
|---|---|---|---|---|---|---|---|---|---|---|---|---|---|---|---|
| J3KSR8 | Serine/arginine-rich splicing factor 1 | SRSF1 | 100 | S | 0.993 | 65.63 | NaN | NaN | 1.36 | NaN | 1.22 | 1.23 | 1.74 | 1.39 | 1.54 |
| J3KSY7 | Protein CASC3 | CASC3 | 35 | S | 1 | 270.2 | 0.82 | 0.81 | 0.80 | 0.71 | 0.73 | 0.72 | 1.00 | 0.95 | 1.04 |
| J3KTD8 | | CBFB | 46 | S | 0.992 | 78.76 | NaN | 1.34 | 1.19 | NaN | NaN | 1.08 | 1.42 | 1.31 | 1.42 |
| J3QK89 | Calcium homeostasis endoplasmic reticulum protein | CHERP | 826 | S | 0.995 | 107 | 0.85 | 1.03 | 1.03 | NaN | 0.99 | NaN | 1.13 | 1.07 | 1.13 |
| J3QK89 | Calcium homeostasis endoplasmic reticulum protein | CHERP | 828 | S | 0.996 | 107 | 0.85 | 1.03 | 1.03 | NaN | 0.99 | NaN | 1.13 | 1.07 | 1.13 |
| J3QK89 | Calcium homeostasis endoplasmic reticulum protein | CHERP | 824 | S | 0.998 | 82.77 | NaN | 1.00 | 1.06 | NaN | 0.87 | NaN | NaN | NaN | 1.08 |
| J3QQN1 | Proteasome subunit beta type-10 | PSMB10 | 110 | S | 0.917 | 61.42 | 1.23 | NaN | NaN | NaN | 1.08 | 0.78 | NaN | NaN | 0.91 |
| J3QQQ3 | Zinc finger protein 830 | ZNF830 | 85 | S | 1 | 161.7 | 1.07 | 1.03 | NaN | 1.33 | 1.22 | NaN | 1.19 | 1.41 | 1.70 |
| J3QR07 | YTH domain-containing protein 1 | YTHDC1 | 308 | S | 1 | 165.6 | NaN | NaN | 1.15 | 0.67 | 0.68 | NaN | 0.77 | 0.84 | NaN |
| J3QRP6 | Na(+)/H(+) exchange regulatory cofactor NHE-RF1 | SLC9A3R1 | 161 | S | 1 | 163.8 | 4.92 | 4.60 | 4.90 | 4.45 | 4.74 | 4.85 | 2.65 | NaN | 2.93 |
| J3QRP6 | Na(+)/H(+) exchange regulatory cofactor NHE-RF1 | SLC9A3R1 | 169 | S | 0.609 | 114.9 | 0.83 | NaN | NaN | NaN | NaN | NaN | 0.94 | 0.89 | NaN |
| J3QRP6 | Na(+)/H(+) exchange regulatory cofactor NHE-RF1 | SLC9A3R1 | 171 | S | 0.999 | 350.6 | 0.79 | 0.90 | 0.70 | 0.89 | 0.80 | 0.93 | 0.88 | 1.29 | 0.77 |
| J3QSD7 | Cyclin-dependent kinase 12 | CDK12 | 680 | S | 1 | 78.41 | 0.92 | 0.54 | 0.80 | 1.08 | 1.13 | NaN | 0.80 | 0.90 | 0.87 |
| J3QSD7 | Cyclin-dependent kinase 12 | CDK12 | 684 | S | 1 | 78.41 | 0.92 | 0.87 | 0.80 | 1.08 | 1.13 | NaN | 1.12 | 0.90 | 0.87 |
| K7EMU2 | cAMP-dependent protein kinase type I-alpha regulatory subunit;cAMP-dependent protein kinase type I-alpha regulatory subunit, N-terminally processed | PRKAR1A | 83 | S | 1 | 150.1 | 0.85 | 0.86 | 0.72 | 0.87 | 0.87 | 0.85 | 0.94 | 0.96 | 1.02 |
| K7ERL1 | Formin-like protein 1 | FMNL1 | 64 | S | 1 | 180.9 | 0.87 | 0.85 | 0.87 | 0.87 | 0.94 | 0.88 | 1.00 | 1.00 | 0.90 |
| K7EQ06 | | HDGFRP2 | 12 | S | 0.936 | 109.6 | 1.36 | 1.28 | 0.85 | NaN | NaN | 0.88 | NaN | NaN | 1.16 |
| K7EKZ3 | Vacuolar protein sorting-associated protein 4B | VPS4B | 102 | S | 1 | 195.1 | 0.94 | 0.89 | 0.85 | 0.90 | 0.81 | 0.88 | 0.99 | 0.94 | 0.85 |
| K7EL01 | F-box/WD repeat-containing protein 9 | FBXW9 | 18 | S | 1 | 188.9 | NaN | 0.50 | 0.64 | 0.40 | NaN | 0.68 | 0.82 | NaN | 1.26 |
| K7EL60 | Eukaryotic translation initiation factor 3 subunit G | EIF3G | 42 | S | 0.897 | 174.4 | 1.32 | 1.37 | 1.42 | 1.24 | 2.15 | 1.23 | 1.04 | 1.33 | 1.04 |
| K7ENR9 | DNA ligase 3 | LIG3 | 123 | S | 0.763 | 130.2 | 0.66 | 0.62 | 0.57 | 1.03 | 1.08 | NaN | 0.59 | 0.69 | 0.68 |
| K7EQA1 | Programmed cell death protein 5 | PDCD5 | 81 | S | 1 | 179.6 | 1.05 | 1.27 | 1.06 | 1.26 | 1.32 | 1.24 | 0.74 | 0.70 | 0.73 |
| K7EQI9 | Protein FAM134C | FAM134C | 216 | S | 0.995 | 54.33 | NaN | NaN | 1.24 | 1.14 | 1.20 | NaN | 1.12 | 1.17 | NaN |
| K7EQI9 | Protein FAM134C | FAM134C | 223 | S | 1 | 267 | 1.03 | 0.97 | 0.92 | 0.86 | 1.20 | 0.82 | 0.98 | 1.02 | 1.00 |
| K7EQI9 | Protein FAM134C | FAM134C | 336 | S | 0.942 | 126.2 | 2.25 | 2.32 | NaN | NaN | 1.89 | NaN | NaN | 1.64 | NaN |
| K7EQI9 | Protein FAM134C | FAM134C | 338 | S | 0.942 | 177.1 | 2.25 | 2.32 | NaN | NaN | 1.89 | NaN | NaN | 1.64 | NaN |
| K7EQI9 | Protein FAM134C | FAM134C | 339 | S | 0.672 | 177.1 | 2.25 | 2.32 | NaN | NaN | NaN | NaN | NaN | 1.64 | NaN |

| | | | | | | | | | | | | | | | |
|---|---|---|---|---|---|---|---|---|---|---|---|---|---|---|---|
| K7ER94 | FH1/FH2 domain-containing protein 3 | FHOD3 | 541 | S | 1 | 166.6 | 0.61 | 0.37 | 0.47 | 0.62 | NaN | 0.53 | 1.66 | 0.71 | 0.76 |
| K7ERB6 | Homologous-pairing protein 2 homolog | PSMC3IP | 131 | S | 0.908 | 61.99 | NaN | NaN | NaN | NaN | NaN | NaN | 1.56 | 1.08 | NaN |
| K7ERU2 | KN motif and ankyrin repeat domain-containing protein 2 | KANK2 | 98 | S | 1 | 88.55 | 1.72 | 1.45 | 1.70 | 1.47 | 1.64 | 1.50 | 1.11 | 1.09 | 1.07 |
| K7ERU2 | KN motif and ankyrin repeat domain-containing protein 2 | KANK2 | 46 | S | 0.999 | 119.8 | 1.57 | 2.02 | NaN | 1.83 | 1.68 | 1.61 | NaN | 0.97 | NaN |
| M0QXN5 | Nuclear pore glycoprotein p62 | NUP62 | 332 | S | 1 | 179.2 | 1.35 | NaN | NaN | 1.59 | 1.72 | NaN | 1.07 | 0.83 | NaN |
| M0QYF9 | Homer protein homolog 3 | HOMER3 | 47 | S | 1 | 69.15 | NaN | NaN | NaN | NaN | NaN | NaN | 1.39 | 1.48 | 2.12 |
| M0QZ09 | NAD-dependent protein deacetylase sirtuin-6 | SIRT6 | 264 | S | 0.823 | 53.98 | 1.24 | 1.11 | 1.39 | 1.05 | NaN | NaN | NaN | NaN | NaN |
| M0QZR4 | Rho guanine nucleotide exchange factor 1 | ARHGEF 1 | 919 | S | 1 | 100.8 | NaN | NaN | NaN | 2.71 | 2.86 | NaN | 0.98 | 0.90 | NaN |
| M0R2H7 | Cdc42-interacting protein 4 | TRIP10 | 296 | S | 0.999 | 164.8 | 0.97 | 0.98 | 0.95 | 0.94 | 0.94 | 0.80 | 1.70 | 1.70 | 1.49 |
| M0R0P8 | Unconventional myosin-IXb | MYO9B | 717 | S | 0.898 | 115.5 | 0.84 | 0.89 | 0.87 | 1.28 | 1.36 | 1.08 | 0.71 | 0.60 | 0.73 |
| M0R0P8 | Unconventional myosin-IXb | MYO9B | 1122 | S | 1 | 76.21 | 0.68 | NaN | 0.54 | 0.82 | 0.60 | 0.55 | 0.78 | 0.85 | 0.74 |
| M0R0P8 | Unconventional myosin-IXb | MYO9B | 1405 | S | 0.993 | 89.4 | NaN | 0.45 | NaN | 0.62 | 0.58 | 0.54 | NaN | NaN | NaN |
| M0R0P8 | Unconventional myosin-IXb | MYO9B | 1935 | S | 1 | 124.1 | 0.83 | 0.82 | 1.25 | 1.25 | 1.25 | 1.31 | 0.72 | 0.86 | 0.83 |
| M0R0P8 | Unconventional myosin-IXb | MYO9B | 1115 | S | 0.538 | 76.21 | NaN | NaN | 0.54 | 0.71 | 0.60 | 0.55 | NaN | 0.85 | 0.74 |
| M0R0P8 | Unconventional myosin-IXb | MYO9B | 1290 | S | 1 | 214.3 | 0.47 | 0.48 | 0.51 | 0.59 | 0.47 | 0.62 | 0.85 | 0.80 | 0.77 |
| M0R0W6 | Receptor protein-tyrosine kinase;Tyrosine-protein kinase receptor UFO | AXL | 616 | S | 0.983 | 72.88 | NaN | 0.61 | NaN | NaN | NaN | NaN | 1.30 | NaN | 1.03 |
| M0R2S2 | Epidermal growth factor receptor substrate 15-like 1 | EPS15L1 | 101 | S | 0.999 | 317.3 | 0.99 | 1.08 | 1.27 | 1.13 | 0.98 | 1.06 | 1.10 | 1.11 | 1.02 |
| M0R2S2 | Epidermal growth factor receptor substrate 15-like 1 | EPS15L1 | 75 | S | 1 | 129.3 | 1.64 | 1.68 | 1.72 | 1.65 | 1.72 | 1.69 | 1.00 | 0.98 | 1.11 |
| O00203 | AP-3 complex subunit beta-1 | AP3B1 | 276 | S | 1 | 229.7 | 1.00 | 1.01 | 1.05 | 1.15 | 1.18 | 1.00 | 1.08 | 1.15 | 1.08 |
| O00264 | Membrane-associated progesterone receptor component 1 | PGRMC1 | 181 | S | 1 | 414.9 | 0.95 | 0.98 | 1.00 | 0.93 | 0.95 | 1.05 | 1.04 | 1.07 | 1.16 |
| O00264 | Membrane-associated progesterone receptor component 1 | PGRMC1 | 54 | S | 0.949 | 118.2 | NaN | NaN | NaN | 1.00 | 0.96 | NaN | NaN | 1.10 | 1.14 |
| O00264 | Membrane-associated progesterone receptor component 1 | PGRMC1 | 57 | S | 1 | 322.5 | 1.00 | 1.26 | 0.99 | 1.13 | 1.12 | 0.98 | 1.14 | 1.06 | 1.12 |
| O00273 | DNA fragmentation factor subunit alpha | DFFA | 315 | S | 0.998 | 134.8 | 0.76 | 0.81 | NaN | 1.49 | 1.33 | 1.05 | 1.38 | 1.42 | 0.98 |
| O00303 | Eukaryotic translation initiation factor 3 subunit F | EIF3F | 258 | S | 1 | 106.9 | 1.03 | 1.03 | 0.99 | 1.28 | 1.37 | 1.20 | 1.14 | 0.97 | 0.98 |
| O00308 | NEDD4-like E3 ubiquitin-protein ligase WWP2 | WWP2 | 211 | S | 0.991 | 84.95 | 1.00 | 0.86 | 1.06 | 0.84 | NaN | 3.13 | 4.01 | 4.20 | 1.44 |
| O00425 | Insulin-like growth factor 2 mRNA-binding protein 3 | IGF2BP3 | 184 | S | 0.999 | 49.03 | 0.95 | NaN | 0.98 | 0.89 | NaN | NaN | 1.05 | NaN | NaN |

| | | | | | | | | | | | | | | | |
|---|---|---|---|---|---|---|---|---|---|---|---|---|---|---|---|
| O00499 | Myc box-dependent-interacting protein 1 | BIN1 | 296 | S | 1 | 102.6 | 1.23 | 2.38 | 1.01 | 0.80 | 0.85 | 0.84 | 1.53 | 0.87 | 0.87 |
| O00499 | Myc box-dependent-interacting protein 1 | BIN1 | 298 | S | 1 | 182.2 | 1.05 | 0.97 | 0.93 | 1.04 | 0.92 | 0.94 | 1.12 | 1.20 | 1.13 |
| O00499 | Myc box-dependent-interacting protein 1 | BIN1 | 303 | S | 1 | 136.4 | 1.23 | 2.38 | 1.58 | 1.32 | 0.85 | NaN | 1.46 | 1.08 | 0.78 |
| O00505 | Importin subunit alpha-4 | KPNA3 | 60 | S | 1 | 226.2 | 1.73 | 1.81 | 1.81 | 1.36 | 1.36 | 1.40 | 1.91 | 2.09 | 2.03 |
| U3KQ48 | U3 small nucleolar ribonucleoprotein protein MPP10 | MPHOSPH10 | 163 | S | 1 | 160.1 | 0.76 | 0.68 | 0.83 | 0.76 | 0.90 | 0.97 | 0.65 | 0.48 | 1.17 |
| U3KQ48 | U3 small nucleolar ribonucleoprotein protein MPP10 | MPHOSPH10 | 167 | S | 1 | 160.1 | 0.76 | 0.68 | 0.83 | 0.76 | 0.90 | 0.97 | 0.65 | 0.48 | 1.17 |
| U3KQ48 | U3 small nucleolar ribonucleoprotein protein MPP10 | MPHOSPH10 | 171 | S | 1 | 122.5 | 1.05 | 1.03 | 0.83 | 1.02 | 1.01 | 0.97 | 1.19 | 1.14 | 1.17 |
| O00567 | Nucleolar protein 56 | NOP56 | 519 | S | 1 | 142.3 | 0.93 | 0.99 | 0.93 | 1.22 | NaN | 0.92 | 0.70 | 1.05 | 1.14 |
| O00567 | Nucleolar protein 56 | NOP56 | 520 | S | 1 | 178.5 | 0.74 | 0.96 | 0.82 | 0.97 | 1.23 | 0.96 | 0.85 | 0.92 | 0.87 |
| O00567 | Nucleolar protein 56 | NOP56 | 569 | S | 1 | 63.73 | 0.76 | 1.22 | NaN | NaN | NaN | NaN | NaN | NaN | NaN |
| O00567 | Nucleolar protein 56 | NOP56 | 570 | S | 1 | 106.4 | 1.57 | NaN | NaN | 1.44 | NaN | NaN | 0.98 | NaN | 1.24 |
| O14497 | AT-rich interactive domain-containing protein 1A | ARID1A | 696 | S | 0.998 | 104.2 | 1.21 | 1.09 | NaN | 1.92 | 1.44 | 1.14 | NaN | 1.29 | NaN |
| O14497 | AT-rich interactive domain-containing protein 1A | ARID1A | 1600 | S | 0.896 | 78.79 | NaN | NaN | NaN | NaN | NaN | NaN | 0.33 | 0.51 | 0.44 |
| O14523 | C2 domain-containing protein 2-like | C2CD2L | 662 | S | 0.799 | 106.3 | 1.63 | NaN | NaN | 1.22 | NaN | 1.34 | 1.17 | 1.23 | 1.26 |
| O14545 | TRAF-type zinc finger domain-containing protein 1 | TRAFD1 | 415 | S | 0.966 | 159.4 | 0.99 | 0.92 | 0.82 | 0.92 | 0.78 | 0.96 | 0.94 | 1.01 | NaN |
| O14613 | Cdc42 effector protein 2 | CDC42EP2 | 141 | S | 0.991 | 61.52 | 4.09 | 2.62 | NaN | NaN | NaN | NaN | NaN | NaN | NaN |
| O14686 | Histone-lysine N-methyltransferase 2D | KMT2D | 4738 | S | 1 | 51.01 | 1.05 | 1.05 | NaN | NaN | NaN | NaN | 1.23 | NaN | 1.20 |
| O14686 | Histone-lysine N-methyltransferase 2D | KMT2D | 1671 | S | 1 | 66.02 | NaN | 0.62 | NaN | 1.12 | 1.35 | NaN | NaN | NaN | NaN |
| O14828 | Secretory carrier-associated membrane protein 3 | SCAMP3 | 76 | S | 0.997 | 96.67 | 0.91 | 0.95 | 0.98 | 1.15 | 1.13 | 1.10 | 0.70 | 0.69 | 0.71 |
| O14879 | Interferon-induced protein with tetratricopeptide repeats 3 | IFIT3 | 478 | S | 0.888 | 69.02 | NaN | NaN | NaN | NaN | 1.14 | 1.49 | NaN | NaN | NaN |
| O14974 | Protein phosphatase 1 regulatory subunit 12A | PPP1R12A | 299 | S | 1 | 107 | 0.52 | 0.33 | NaN | 0.83 | NaN | NaN | NaN | 0.49 | NaN |
| O14974 | Protein phosphatase 1 regulatory subunit 12A | PPP1R12A | 862 | S | 1 | 227.4 | NaN | 0.87 | NaN | NaN | NaN | NaN | 1.49 | NaN | 0.83 |
| O14974 | Protein phosphatase 1 regulatory subunit 12A | PPP1R12A | 871 | S | 0.986 | 326.5 | 1.02 | NaN | 1.02 | 0.88 | NaN | 1.24 | 1.26 | 1.04 | 1.04 |
| O15173 | Membrane-associated progesterone receptor component 2 | PGRMC2 | 90 | S | 0.997 | 218.1 | NaN | NaN | NaN | 3.14 | 3.40 | NaN | 1.19 | NaN | 1.08 |

| | | | | | | | | | | | | | | | |
|---|---|---|---|---|---|---|---|---|---|---|---|---|---|---|---|
| O15173 | Membrane-associated progesterone receptor component 2 | PGRMC2 | 208 | S | 0.998 | 125.7 | 1.70 | 1.85 | 1.91 | 2.20 | 1.99 | 2.08 | 1.98 | 1.23 | 1.67 |
| O15446 | DNA-directed RNA polymerase I subunit RPA34 | CD3EAP | 136 | S | 1 | 85.02 | NaN | 2.21 | 2.23 | NaN | 2.63 | NaN | 1.26 | 1.40 | 1.75 |
| O15446 | DNA-directed RNA polymerase I subunit RPA34 | CD3EAP | 285 | S | 0.94 | 153.4 | 1.42 | 1.54 | 1.34 | 1.41 | 1.51 | 1.58 | 0.64 | 0.60 | 0.75 |
| O15541 | RING finger protein 113A | RNF113A | 84 | S | 0.997 | 121.3 | 1.14 | 1.25 | 1.02 | 0.85 | 0.93 | 0.78 | 0.85 | 0.83 | 0.77 |
| O15541 | RING finger protein 113A | RNF113A | 85 | S | 0.995 | 121.3 | 1.14 | 1.25 | 1.02 | 0.85 | 0.93 | 0.78 | 0.85 | 0.83 | 0.77 |
| O15541 | RING finger protein 113A | RNF113A | 253 | S | 1 | 201.7 | 1.47 | 1.56 | 1.58 | 1.34 | 1.39 | 1.25 | 1.14 | 1.36 | 1.34 |
| O43150 | Arf-GAP with SH3 domain, ANK repeat and PH domain-containing protein 2 | ASAP2 | 701 | S | 1 | 316.6 | 0.90 | 0.87 | 0.77 | 1.08 | 1.13 | 0.98 | 0.62 | 0.66 | 0.58 |
| O43237 | Cytoplasmic dynein 1 light intermediate chain 2 | DYNC1LI2 | 194 | S | 1 | 201.2 | 0.66 | 0.63 | 0.74 | 0.85 | 0.83 | 0.86 | 0.71 | 0.67 | 0.68 |
| O43237 | Cytoplasmic dynein 1 light intermediate chain 2 | DYNC1LI2 | 407 | S | 0.719 | 111 | 1.96 | 2.92 | NaN | NaN | NaN | NaN | NaN | NaN | NaN |
| O43290 | U4/U6.U5 tri-snRNP-associated protein 1 | SART1 | 448 | S | 1 | 114.1 | 1.74 | 1.85 | 1.39 | 1.66 | 1.49 | 1.47 | 2.20 | 2.17 | 1.84 |
| O43290 | U4/U6.U5 tri-snRNP-associated protein 1 | SART1 | 598 | S | 0.894 | 83.56 | 0.70 | 0.59 | NaN | 0.71 | NaN | NaN | NaN | 0.82 | NaN |
| O43290 | U4/U6.U5 tri-snRNP-associated protein 1 | SART1 | 474 | S | 1 | 137.7 | 0.60 | 0.51 | 0.58 | 0.91 | 1.10 | 0.70 | 0.77 | 0.77 | 0.67 |
| O43290 | U4/U6.U5 tri-snRNP-associated protein 1 | SART1 | 486 | S | 1 | 137.7 | 0.60 | 0.51 | 0.58 | 0.91 | 1.10 | 0.70 | 0.77 | 0.77 | 0.67 |
| O43294 | Transforming growth factor beta-1-induced transcript 1 protein | TGFB1I1 | 137 | S | 1 | 87.33 | 2.47 | 2.36 | 2.61 | 0.74 | 1.30 | 2.91 | 1.40 | 1.53 | NaN |
| O43294 | Transforming growth factor beta-1-induced transcript 1 protein | TGFB1I1 | 140 | S | 0.612 | 87.33 | 2.47 | 2.36 | 2.61 | NaN | NaN | 2.91 | 2.13 | NaN | NaN |
| O43294 | Transforming growth factor beta-1-induced transcript 1 protein | TGFB1I1 | 141 | S | 0.934 | 79.88 | NaN | NaN | NaN | 2.49 | 2.71 | NaN | NaN | 2.22 | NaN |
| O43294 | Transforming growth factor beta-1-induced transcript 1 protein | TGFB1I1 | 68 | S | 1 | 145.1 | 1.47 | 1.21 | 1.39 | 1.39 | 1.37 | 1.50 | 1.39 | 1.45 | 1.40 |
| O43294 | Transforming growth factor beta-1-induced transcript 1 protein | TGFB1I1 | 194 | S | 0.945 | 97.08 | 0.96 | 0.64 | 1.07 | 1.34 | 1.15 | NaN | 1.39 | 1.17 | 1.30 |
| Q9UG54 | Mitogen-activated protein kinase kinase kinase 7 | DKFZp586F0420 | 93 | S | 1 | 177.6 | 1.91 | 1.78 | 1.48 | 1.46 | 1.44 | NaN | 1.86 | 1.92 | 1.86 |
| O43379 | WD repeat-containing protein 62 | WDR62 | 1144 | S | 0.881 | 83.6 | 0.35 | 0.41 | NaN | NaN | NaN | NaN | 0.62 | NaN | NaN |
| O43379 | WD repeat-containing protein 62 | WDR62 | 32 | S | 0.64 | 80.91 | 0.70 | NaN | 0.78 | NaN | 0.57 | 0.94 | 1.17 | NaN | 1.02 |

| | | | | | | | | | | | | | | | |
|---|---|---|---|---|---|---|---|---|---|---|---|---|---|---|---|
| O43379 | WD repeat-containing protein 62 | WDR62 | 33 | S | 0.941 | 78.89 | NaN | 0.97 | 0.78 | NaN | 0.57 | NaN | 1.17 | 0.79 | 1.02 |
| O43379 | WD repeat-containing protein 62 | WDR62 | 1228 | S | 0.99 | 139.8 | 1.04 | 1.14 | 0.77 | NaN | NaN | NaN | NaN | NaN | NaN |
| O43395 | U4/U6 small nuclear ribonucleoprotein Prp3 | PRPF3 | 619 | S | 1 | 47.73 | NaN | NaN | 0.44 | NaN | 0.70 | 0.88 | NaN | 0.74 | 0.85 |
| O43399 | Tumor protein D54 | TPD52L2 | 166 | S | 1 | 190.8 | 1.46 | 1.65 | 1.58 | 1.28 | 1.27 | 1.31 | 3.55 | 3.45 | 3.46 |
| O43463 | Histone-lysine N-methyltransferase SUV39H1 | SUV39H1 | 391 | S | 1 | 79.49 | NaN | NaN | NaN | 0.54 | 0.75 | NaN | NaN | NaN | NaN |
| O43493 | Trans-Golgi network integral membrane protein 2 | TGOLN2 | 71 | S | 0.991 | 210.7 | 0.71 | 0.94 | 0.91 | 0.92 | NaN | 0.97 | 0.74 | 0.94 | 0.82 |
| O43493 | Trans-Golgi network integral membrane protein 2 | TGOLN2 | 298 | S | 1 | 257 | NaN | 1.13 | NaN | 1.31 | NaN | NaN | 1.17 | NaN | 1.98 |
| O43719 | HIV Tat-specific factor 1 | HTATSF1 | 579 | S | 1 | 104.8 | NaN | 1.11 | 0.97 | 1.02 | 1.06 | NaN | 1.17 | NaN | 1.26 |
| O43719 | HIV Tat-specific factor 1 | HTATSF1 | 624 | S | 1 | 251.7 | 0.73 | 0.70 | 0.71 | 0.82 | 0.80 | 0.78 | 0.82 | 0.90 | 0.73 |
| O43719 | HIV Tat-specific factor 1 | HTATSF1 | 498 | S | 1 | 145.4 | 0.76 | 0.82 | 1.18 | NaN | NaN | 1.39 | 0.87 | NaN | 0.99 |
| O43719 | HIV Tat-specific factor 1 | HTATSF1 | 453 | S | 0.985 | 157.3 | 0.93 | 0.92 | 0.93 | 1.04 | 1.04 | 1.13 | 0.95 | 1.02 | 1.29 |
| O43719 | HIV Tat-specific factor 1 | HTATSF1 | 676 | S | 1 | 311.8 | 0.80 | 0.83 | 0.82 | 0.91 | 0.96 | 0.87 | 0.89 | 0.87 | 0.91 |
| O43719 | HIV Tat-specific factor 1 | HTATSF1 | 642 | S | 1 | 252.1 | 0.90 | 0.84 | 0.88 | 0.98 | 1.24 | 0.88 | 1.14 | 1.15 | 1.05 |
| O43719 | HIV Tat-specific factor 1 | HTATSF1 | 616 | S | 1 | 155.9 | 0.75 | 0.75 | 0.77 | 0.99 | 1.01 | 1.01 | 0.97 | 1.01 | 1.05 |
| O43765 | Small glutamine-rich tetratricopeptide repeat-containing protein alpha | SGTA | 77 | S | 1 | 121.9 | 1.35 | 1.57 | 1.43 | 2.40 | 2.39 | 2.43 | 0.83 | 0.80 | 0.85 |
| O43765 | Small glutamine-rich tetratricopeptide repeat-containing protein alpha | SGTA | 305 | S | 0.977 | 237.1 | NaN | NaN | 1.24 | 1.60 | NaN | 1.51 | NaN | 1.36 | NaN |
| O43815 | Striatin | STRN | 245 | S | 1 | 176.4 | 0.96 | NaN | NaN | NaN | NaN | NaN | 1.11 | NaN | 1.29 |
| O43815 | Striatin | STRN | 137 | S | 0.998 | 204.2 | 0.36 | 0.44 | 0.34 | 0.76 | 0.70 | 0.75 | 0.36 | 0.34 | 0.49 |
| O60244 | Mediator of RNA polymerase II transcription subunit 14 | MED14 | 986 | S | 1 | 140.5 | 0.37 | 0.36 | 0.42 | 0.63 | NaN | 0.55 | 0.34 | 0.42 | 0.50 |
| O60264 | SWI/SNF-related matrix-associated actin-dependent regulator of chromatin subfamily A member 5 | SMARCA5 | 66 | S | 1 | 231.5 | NaN | 0.50 | NaN | 0.75 | 0.86 | 0.88 | 0.67 | NaN | 0.46 |
| O60271 | C-Jun-amino-terminal kinase-interacting protein 4 | SPAG9 | 329 | S | 0.936 | 66.57 | NaN | 0.54 | NaN | 1.00 | 0.92 | 0.72 | 0.95 | 1.09 | NaN |
| O60271 | C-Jun-amino-terminal kinase-interacting protein 4 | SPAG9 | 332 | S | 0.997 | 79.61 | 0.46 | 0.47 | 0.68 | 0.57 | 0.67 | 0.59 | 1.08 | 1.15 | 1.17 |
| O60271 | C-Jun-amino-terminal kinase-interacting protein 4 | SPAG9 | 730 | S | 0.999 | 125.2 | 0.93 | 1.06 | 1.41 | 1.31 | 1.37 | NaN | 0.96 | 1.00 | 1.21 |
| O60271 | C-Jun-amino-terminal kinase-interacting protein 4 | SPAG9 | 732 | S | 0.688 | 66.78 | NaN | NaN | 1.41 | NaN | 1.34 | NaN | 1.38 | NaN | 0.90 |
| O60271 | C-Jun-amino-terminal kinase-interacting protein 4 | SPAG9 | 733 | S | 0.985 | 192.9 | 0.86 | 0.68 | 0.66 | 0.82 | 0.81 | 0.64 | 0.79 | 0.82 | 0.66 |
| O60271 | C-Jun-amino-terminal kinase-interacting protein 4 | SPAG9 | 251 | S | 1 | 105.9 | 0.72 | 0.73 | 0.79 | 0.98 | 0.90 | 0.90 | 1.03 | 0.92 | 0.96 |

| | | | | | | | | | | | | | | | |
|---|---|---|---|---|---|---|---|---|---|---|---|---|---|---|---|
| O60292 | Signal-induced proliferation-associated 1-like protein 3 | SIPA1L3 | 1364 | S | 1 | 65.57 | NaN | NaN | NaN | NaN | NaN | NaN | 2.21 | 3.49 | NaN |
| O60292 | Signal-induced proliferation-associated 1-like protein 3 | SIPA1L3 | 1534 | S | 0.5 | 48.33 | NaN | 0.97 | 0.81 | 1.09 | NaN | NaN | NaN | NaN | NaN |
| O60292 | Signal-induced proliferation-associated 1-like protein 3 | SIPA1L3 | 1544 | S | 0.996 | 88.1 | NaN | 1.28 | 1.24 | NaN | 0.89 | NaN | 1.81 | 1.81 | 1.70 |
| O60502 | Protein O-GlcNAcase | MGEA5 | 364 | S | 1 | 101 | 0.67 | 0.61 | 0.64 | 0.93 | 0.95 | 0.82 | 0.65 | 0.61 | NaN |
| O60551 | Glycylpeptide N-tetradecanoyltransferase 2 | NMT2 | 66 | S | 0.959 | 78.78 | 0.51 | 0.50 | 0.52 | 0.72 | 0.62 | 0.55 | 0.59 | 0.55 | 1.00 |
| O60551 | Glycylpeptide N-tetradecanoyltransferase 2 | NMT2 | 68 | S | 0.997 | 78.78 | 0.51 | 0.50 | 1.54 | 1.03 | 1.16 | 1.00 | 0.94 | 1.33 | 1.05 |
| O60551 | Glycylpeptide N-tetradecanoyltransferase 2 | NMT2 | 70 | S | 0.98 | 78.78 | NaN | NaN | 0.84 | NaN | 0.90 | NaN | NaN | 0.96 | 1.00 |
| O60551 | Glycylpeptide N-tetradecanoyltransferase 2 | NMT2 | 38 | S | 1 | 91.75 | NaN | 0.78 | 0.76 | 0.98 | NaN | 0.93 | 1.01 | 0.98 | 0.96 |
| O60566 | Mitotic checkpoint serine/threonine-protein kinase BUB1 beta | BUB1B | 670 | S | 1 | 43.54 | NaN | NaN | NaN | 1.15 | 1.25 | NaN | NaN | NaN | NaN |
| O60763 | General vesicular transport factor p115 | USO1 | 942 | S | 1 | 269.8 | 1.24 | 1.32 | 1.32 | 1.30 | 1.09 | NaN | 0.89 | NaN | 1.07 |
| O60784 | Target of Myb protein 1 | TOM1 | 462 | S | 0.8 | 86.48 | 1.97 | NaN | NaN | 1.62 | 1.85 | NaN | 1.22 | NaN | 1.39 |
| O60832 | H/ACA ribonucleoprotein complex subunit 4 | DKC1 | 485 | S | 1 | 129.5 | NaN | NaN | 1.43 | 1.54 | 0.60 | 1.55 | 1.40 | 1.17 | NaN |
| O60832 | H/ACA ribonucleoprotein complex subunit 4 | DKC1 | 494 | S | 0.998 | 310.1 | 0.89 | 0.86 | 0.97 | 0.92 | 0.96 | 0.96 | 0.96 | 0.78 | 0.90 |
| O60832 | H/ACA ribonucleoprotein complex subunit 4 | DKC1 | 513 | S | 1 | 118.9 | 1.21 | 1.17 | 1.30 | 1.11 | NaN | 1.11 | 1.04 | 0.96 | 1.10 |
| O60832 | H/ACA ribonucleoprotein complex subunit 4 | DKC1 | 451 | S | 1 | 99.37 | 1.00 | 1.06 | 1.30 | 1.00 | 1.03 | 0.87 | 0.98 | 0.96 | 0.56 |
| O60832 | H/ACA ribonucleoprotein complex subunit 4 | DKC1 | 453 | S | 1 | 99.37 | 1.00 | 1.06 | 1.30 | 1.00 | 1.03 | 1.00 | 0.98 | 0.96 | 0.97 |
| O60832 | H/ACA ribonucleoprotein complex subunit 4 | DKC1 | 455 | S | 1 | 98.63 | 1.17 | 1.26 | 1.30 | 0.93 | 1.78 | 1.49 | 1.26 | 0.95 | 1.09 |
| O75379 | Vesicle-associated membrane protein 4 | VAMP4 | 30 | S | 1 | 221.9 | 1.01 | 1.11 | 1.08 | 1.12 | 0.95 | 0.95 | 1.03 | 1.14 | 1.01 |
| O75436 | Vacuolar protein sorting-associated protein 26A | VPS26A | 315 | S | 1 | 229.5 | NaN | NaN | 2.03 | 2.43 | 2.60 | 2.76 | NaN | 0.40 | 0.41 |
| O75475 | PC4 and SFRS1-interacting protein | PSIP1 | 106 | S | 0.985 | 179 | 0.69 | NaN | NaN | 0.76 | 0.73 | 0.77 | 0.82 | 0.81 | 0.89 |
| O75475 | PC4 and SFRS1-interacting protein | PSIP1 | 273 | S | 0.977 | 287 | 0.53 | 0.56 | 0.50 | 0.56 | NaN | 0.52 | 0.62 | NaN | 0.63 |
| O75475 | PC4 and SFRS1-interacting protein | PSIP1 | 275 | S | 1 | 287 | 0.53 | 0.56 | 0.50 | 0.56 | 0.57 | 0.52 | 0.62 | 0.73 | 0.63 |
| Q5JRI1 | Serine/arginine-rich splicing factor 10 | SRSF10 | 133 | S | 1 | 84.06 | 1.00 | 1.08 | 1.08 | 1.01 | 1.07 | 0.97 | 1.38 | 1.14 | 1.34 |
| Q5JRI1 | Serine/arginine-rich splicing factor 10 | SRSF10 | 131 | S | 1 | 61.11 | 1.02 | 1.06 | 1.01 | 0.95 | 0.97 | 0.93 | 1.24 | 1.25 | 1.24 |

| | | | | | | | | | | | | | | | |
|---|---|---|---|---|---|---|---|---|---|---|---|---|---|---|---|
| Q5JRI1 | Serine/arginine-rich splicing factor 10 | SRSF10 | 129 | S | 1 | 61.11 | NaN | 1.09 | 1.08 | 0.96 | 0.96 | NaN | 1.25 | 1.28 | NaN |
| O75533 | Splicing factor 3B subunit 1 | SF3B1 | 488 | S | 0.993 | 128.9 | 2.04 | 2.18 | 2.43 | 3.80 | 3.69 | 3.07 | NaN | 0.66 | NaN |
| O75533 | Splicing factor 3B subunit 1 | SF3B1 | 129 | S | 1 | 129 | 0.90 | 0.85 | 0.82 | 1.12 | 0.93 | 0.98 | 0.63 | 0.71 | 0.74 |
| O75683 | Surfeit locus protein 6 | SURF6 | 138 | S | 1 | 97.16 | 0.95 | NaN | 0.90 | 0.93 | 0.99 | 0.93 | 0.92 | 0.97 | 0.92 |
| S4R419 | Transcription elongation factor A protein 3 | TCEA3 | 78 | S | 1 | 107.6 | 0.41 | 0.40 | 0.67 | 0.52 | 0.55 | 0.63 | 0.42 | 0.57 | 0.62 |
| O76021 | Ribosomal L1 domain-containing protein 1 | RSL1D1 | 427 | S | 0.952 | 67.93 | NaN | NaN | 0.83 | 1.65 | NaN | 1.31 | 0.62 | NaN | 0.75 |
| O76021 | Ribosomal L1 domain-containing protein 1 | RSL1D1 | 361 | S | 1 | 220.1 | 1.65 | 1.64 | 1.63 | 1.73 | 1.68 | 1.73 | 1.03 | 0.86 | 1.09 |
| O76021 | Ribosomal L1 domain-containing protein 1 | RSL1D1 | 443 | S | 0.997 | 48.57 | 1.46 | 1.58 | NaN | 2.14 | 2.37 | NaN | NaN | 0.60 | 0.80 |
| Q5QPB1 | AN1-type zinc finger protein 5 | ZFAND5 | 100 | S | 0.958 | 105.1 | NaN | 1.43 | NaN | 1.20 | 1.80 | NaN | NaN | NaN | NaN |
| O76094 | Signal recognition particle subunit SRP72 | SRP72 | 620 | S | 0.772 | 146.6 | 0.54 | 1.07 | NaN | 1.20 | NaN | NaN | NaN | NaN | NaN |
| O76094 | Signal recognition particle subunit SRP72 | SRP72 | 621 | S | 0.976 | 264.8 | 0.64 | 0.81 | 0.52 | 0.53 | NaN | 0.57 | 0.81 | NaN | 1.00 |
| O76094 | Signal recognition particle subunit SRP72 | SRP72 | 625 | S | 0.994 | 264.8 | 1.09 | 1.08 | 1.13 | 1.10 | NaN | 1.09 | 0.87 | 0.88 | 0.94 |
| O94804 | Serine/threonine-protein kinase 10 | STK10 | 438 | S | 1 | 304.7 | 1.13 | 1.16 | 1.13 | 1.03 | 1.05 | 1.01 | 1.18 | 1.16 | 1.10 |
| O94806 | Serine/threonine-protein kinase D3 | PRKD3 | 27 | S | 0.705 | 80.24 | 0.94 | 1.21 | 1.76 | 1.07 | NaN | NaN | NaN | NaN | NaN |
| O94806 | Serine/threonine-protein kinase D3 | PRKD3 | 30 | S | 0.844 | 80.24 | 0.94 | 1.21 | 1.76 | 1.07 | NaN | NaN | 1.55 | 2.10 | NaN |
| O94806 | Serine/threonine-protein kinase D3 | PRKD3 | 31 | S | 0.844 | 73.06 | 0.94 | 1.21 | 1.76 | 1.07 | NaN | NaN | 1.55 | 2.10 | NaN |
| O94826 | Mitochondrial import receptor subunit TOM70 | TOMM70 A | 91 | S | 1 | 184.3 | 1.01 | 1.07 | 1.09 | 1.07 | 1.23 | 1.06 | 0.88 | 0.89 | 0.90 |
| O94874 | E3 UFM1-protein ligase 1 | UFL1 | 458 | S | 1 | 171.4 | 0.71 | 0.81 | 0.84 | 0.83 | 0.82 | 0.80 | 0.89 | 0.94 | 0.89 |
| O95104 | Splicing factor, arginine/serine-rich 15 | SCAF4 | 154 | S | 1 | 191.8 | 0.80 | 0.81 | 0.80 | 1.05 | NaN | 0.93 | 0.72 | NaN | 0.72 |
| O95155 | Ubiquitin conjugation factor E4 B | UBE4B | 88 | S | 0.949 | 175.2 | 0.55 | NaN | NaN | 0.63 | NaN | 0.75 | 0.98 | NaN | NaN |
| O95218 | Zinc finger Ran-binding domain-containing protein 2 | ZRANB2 | 120 | S | 1 | 209.7 | NaN | NaN | NaN | 1.01 | NaN | NaN | 1.44 | 1.51 | NaN |
| O95218 | Zinc finger Ran-binding domain-containing protein 2 | ZRANB2 | 153 | S | 1 | 178.9 | 1.17 | NaN | 1.09 | NaN | NaN | NaN | NaN | NaN | 1.56 |
| O95218 | Zinc finger Ran-binding domain-containing protein 2 | ZRANB2 | 188 | S | 1 | 103.4 | 1.10 | NaN | 0.98 | 0.81 | NaN | NaN | 1.01 | 1.06 | 2.19 |
| O95297 | Myelin protein zero-like protein 1 | MPZL1 | 210 | S | 0.996 | 192.2 | 2.11 | 2.13 | NaN | 2.14 | 1.75 | 1.65 | 0.75 | 0.84 | 0.87 |
| O95297 | Myelin protein zero-like protein 1 | MPZL1 | 219 | S | 0.992 | 57.28 | NaN | 1.59 | NaN | 2.28 | 2.02 | NaN | 1.01 | 0.99 | NaN |
| O95365 | Zinc finger and BTB domain-containing protein 7A | ZBTB7A | 337 | S | 1 | 172.6 | 1.01 | 1.02 | 0.87 | 0.91 | 0.91 | 0.82 | 1.00 | 0.93 | 1.09 |

| | | | | | | | | | | | | | | |
|---|---|---|---|---|---|---|---|---|---|---|---|---|---|---|
| O95365 | Zinc finger and BTB domain-containing protein 7A | ZBTB7A | 525 | S | 0.652 | 148.4 | 0.80 | 0.89 | 0.92 | NaN | NaN | 1.02 | 0.96 | 0.93 | NaN |
| O95365 | Zinc finger and BTB domain-containing protein 7A | ZBTB7A | 526 | S | 0.573 | 148.4 | NaN | NaN | NaN | NaN | NaN | NaN | NaN | 0.93 | 1.04 |
| O95365 | Zinc finger and BTB domain-containing protein 7A | ZBTB7A | 549 | S | 1 | 55.66 | 1.07 | 0.79 | NaN | NaN | NaN | NaN | NaN | NaN | NaN |
| O95391 | Pre-mRNA-splicing factor | SLU7 | 215 | S | 1 | 108.2 | 0.99 | 1.00 | 1.04 | 1.01 | 0.99 | 0.94 | 1.17 | 1.14 | 1.11 |
| O95400 | CD2 antigen cytoplasmic tail-binding protein 2 | CD2BP2 | 195 | S | 0.836 | 54.02 | 0.74 | NaN | NaN | NaN | 0.89 | NaN | 0.91 | 0.79 | NaN |
| O95425 | Supervillin | SVIL | 245 | S | 1 | 161 | 0.79 | 0.88 | 0.91 | 1.01 | 0.93 | 1.08 | 1.06 | 1.05 | 0.96 |
| O95425 | Supervillin | SVIL | 1322 | S | 1 | 70.45 | 0.85 | 0.79 | 2.57 | 1.58 | NaN | NaN | NaN | NaN | NaN |
| O95425 | Supervillin | SVIL | 968 | S | 1 | 215.5 | 1.10 | 0.98 | 1.10 | 1.33 | 1.49 | 1.39 | 1.27 | 1.45 | 1.39 |
| O95613 | Pericentrin | PCNT | 2177 | S | 1 | 88.49 | NaN | NaN | NaN | 1.43 | 1.52 | NaN | 0.65 | 0.70 | NaN |
| O95628 | CCR4-NOT transcription complex subunit 4 | CNOT4 | 430 | S | 0.674 | 136 | 1.83 | 1.58 | NaN | NaN | NaN | NaN | NaN | NaN | NaN |
| O95628 | CCR4-NOT transcription complex subunit 4 | CNOT4 | 432 | S | 0.938 | 195.9 | NaN | NaN | 1.77 | 1.96 | 2.11 | 1.77 | 1.48 | NaN | NaN |
| O95674 | Phosphatidate cytidylyltransferase 2 | CDS2 | 23 | S | 0.902 | 74.81 | 1.52 | 1.29 | NaN | NaN | NaN | NaN | NaN | NaN | NaN |
| O95674 | Phosphatidate cytidylyltransferase 2 | CDS2 | 33 | S | 0.672 | 135.9 | 0.95 | 1.29 | 1.00 | 0.97 | 1.00 | 1.04 | NaN | 1.01 | 0.94 |
| O95684 | FGFR1 oncogene partner | FGFR1OP | 156 | S | 1 | 124.4 | 0.80 | 0.99 | 0.84 | 0.84 | 0.95 | 0.89 | 0.83 | 0.72 | 0.69 |
| O95684 | FGFR1 oncogene partner | FGFR1OP | 160 | S | 1 | 124.4 | 0.80 | 0.77 | 0.84 | 0.84 | 0.95 | 0.89 | 0.83 | 0.72 | 0.77 |
| O95810 | Serum deprivation-response protein | SDPR | 25 | S | 0.974 | 157.3 | 0.73 | 0.71 | 0.67 | 1.05 | 1.10 | 1.04 | 0.33 | 0.34 | 0.39 |
| O95810 | Serum deprivation-response protein | SDPR | 27 | S | 0.952 | 104.1 | 1.00 | 0.87 | NaN | 1.16 | NaN | 0.69 | NaN | 1.33 | NaN |
| O95810 | Serum deprivation-response protein | SDPR | 293 | S | 0.995 | 78.07 | 0.56 | 0.60 | 0.88 | 0.53 | 0.50 | 1.45 | 0.84 | NaN | NaN |
| O95817 | BAG family molecular chaperone regulator 3 | BAG3 | 171 | S | 0.614 | 145.8 | 2.08 | 2.10 | 2.39 | 1.73 | 1.91 | 1.70 | 1.17 | 1.08 | 1.19 |
| O95817 | BAG family molecular chaperone regulator 3 | BAG3 | 173 | S | 1 | 359.9 | 2.40 | 2.31 | 1.98 | 2.00 | 1.79 | 1.94 | 1.11 | 1.19 | 1.21 |
| O95817 | BAG family molecular chaperone regulator 3 | BAG3 | 275 | S | 0.874 | 86.25 | 5.71 | 6.01 | 5.48 | 3.44 | 3.60 | 2.93 | 3.32 | 3.33 | 3.29 |
| O95817 | BAG family molecular chaperone regulator 3 | BAG3 | 279 | S | 1 | 86.25 | 6.19 | 6.20 | 5.48 | 3.44 | 3.60 | 2.93 | 3.32 | 3.33 | 3.29 |
| O95817 | BAG family molecular chaperone regulator 3 | BAG3 | 377 | S | 1 | 151.3 | 1.49 | 1.63 | 1.69 | 1.42 | 2.82 | 1.45 | 1.27 | 1.33 | 1.30 |
| O95817 | BAG family molecular chaperone regulator 3 | BAG3 | 385 | S | 0.656 | 133.5 | 1.41 | 1.57 | 2.61 | 1.49 | 2.82 | 1.83 | 0.68 | 0.79 | 0.71 |
| O95817 | BAG family molecular chaperone regulator 3 | BAG3 | 386 | S | 0.836 | 151.3 | 1.69 | 1.64 | 1.63 | 1.53 | 1.18 | 1.57 | 0.69 | 1.24 | 1.29 |
| O95835 | Serine/threonine-protein kinase LATS1 | LATS1 | 613 | S | 0.971 | 114.9 | NaN | NaN | 1.37 | NaN | 1.31 | 1.30 | 0.82 | 0.88 | NaN |

| | | | | | | | | | | | | | | | |
|---|---|---|---|---|---|---|---|---|---|---|---|---|---|---|---|
| Q9NZH5 | Securin-2;Securin | PTTG2 | 165 | S | 1 | 122.5 | 0.83 | NaN | NaN | 1.57 | 1.50 | NaN | 0.99 | NaN | 1.07 |
| O96007 | Molybdopterin synthase catalytic subunit | MOCS2 | 20 | S | 0.984 | 45.01 | NaN | 2.69 | NaN | 2.52 | 3.01 | NaN | 0.91 | NaN | NaN |
| O96013 | Serine/threonine-protein kinase PAK 4 | PAK4 | 181 | S | 0.99 | 53.1 | 0.76 | 0.73 | NaN | 0.73 | 0.78 | NaN | NaN | 1.12 | NaN |
| O96013 | Serine/threonine-protein kinase PAK 4 | PAK4 | 104 | S | 1 | 81.57 | 0.75 | 0.73 | 0.73 | NaN | 0.70 | 0.66 | NaN | NaN | 0.85 |
| P00558 | Phosphoglycerate kinase 1 | PGK1 | 203 | S | 1 | 228.6 | 1.05 | 1.09 | 1.05 | 1.14 | 1.13 | 1.08 | 1.17 | 1.19 | 1.22 |
| P02545 | Prelamin-A/C;Lamin-A/C | LMNA | 390 | S | 1 | 209.8 | 1.36 | 1.42 | 1.38 | 1.40 | 1.32 | 1.41 | 1.34 | 1.34 | 1.40 |
| P02545 | Prelamin-A/C;Lamin-A/C | LMNA | 392 | S | 1 | 201.8 | 1.02 | 1.02 | 0.93 | 1.25 | 1.24 | 1.27 | 1.23 | 1.17 | 1.20 |
| P02545 | Prelamin-A/C;Lamin-A/C | LMNA | 458 | S | 1 | 148.5 | 0.63 | 0.58 | 0.61 | 1.00 | 0.85 | 0.88 | 0.56 | 0.60 | 0.62 |
| P02545 | Prelamin-A/C;Lamin-A/C | LMNA | 22 | S | 0.998 | 295.7 | 2.65 | 2.74 | 2.49 | 4.48 | 4.30 | 4.11 | 0.87 | 0.89 | 0.92 |
| P04406 | Glyceraldehyde-3-phosphate dehydrogenase | GAPDH | 83 | S | 1 | 76.21 | 1.20 | 1.39 | NaN | NaN | NaN | NaN | NaN | NaN | NaN |
| P05387 | 60S acidic ribosomal protein P2;60S acidic ribosomal protein P1 | RPLP2 | 102 | S | 1 | 315.9 | 0.30 | 0.27 | 0.99 | 0.98 | 0.37 | 0.98 | 0.92 | 0.67 | 0.84 |
| P05387 | 60S acidic ribosomal protein P2;60S acidic ribosomal protein P1 | RPLP2 | 105 | S | 1 | 315.9 | 0.52 | 0.43 | 0.28 | 0.35 | 0.35 | 0.35 | 0.68 | 0.83 | 0.70 |
| P05387 | 60S acidic ribosomal protein | RPLP2 | 17 | S | 0.853 | 116.3 | 1.06 | NaN | 1.33 | 1.46 | 1.55 | 1.67 | 0.28 | NaN | NaN |
| P17535 | Transcription factor AP-1;Transcription factor jun-D | JUN | 73;100 | S | 1 | 98.63 | 1.09 | 1.01 | 1.04 | 1.21 | 1.22 | 1.17 | 1.18 | 1.20 | 1.24 |
| P05455 | Lupus La protein | SSB | 366 | S | 1 | 325.3 | 0.84 | 0.84 | 0.87 | 0.90 | 0.88 | 0.89 | 1.00 | 0.95 | 0.98 |
| P06748 | Nucleophosmin | NPM1 | 125 | S | 1 | 359.6 | 0.97 | 1.00 | 0.96 | 0.85 | 0.90 | 0.85 | 0.94 | 0.93 | 0.94 |
| P06748 | Nucleophosmin | NPM1 | 70 | S | 0.994 | 73.77 | NaN | NaN | 0.63 | 0.84 | 0.78 | NaN | 0.63 | 0.91 | 1.14 |
| P06748 | Nucleophosmin | NPM1 | 243 | S | 0.903 | 60.26 | 3.72 | 3.21 | 3.37 | 5.67 | 6.53 | NaN | NaN | NaN | 0.76 |
| V9GYZ6 | Bifunctional glutamate/proline--tRNA ligase;Glutamate--tRNA ligase;Proline--tRNA ligase | EPRS | 889 | S | 0.739 | 110.6 | 0.96 | 1.01 | NaN | NaN | NaN | NaN | 1.38 | NaN | NaN |
| V9GYZ6 | Bifunctional glutamate/proline--tRNA ligase;Glutamate--tRNA ligase;Proline--tRNA ligase | EPRS | 892 | S | 0.808 | 250.9 | 0.76 | 1.01 | 0.78 | NaN | 0.82 | NaN | 1.38 | NaN | NaN |
| V9GYZ6 | Bifunctional glutamate/proline--tRNA ligase;Glutamate--tRNA ligase;Proline--tRNA ligase | EPRS | 893 | S | 0.89 | 312.5 | 0.71 | 0.74 | 0.73 | 0.81 | 0.79 | 0.80 | 0.93 | 0.91 | 0.96 |
| P07900 | Heat shock protein HSP 90-alpha | HSP90AA1 | 231 | S | 1 | 173.2 | 0.95 | 0.94 | NaN | 0.86 | 0.94 | 0.96 | 0.87 | 0.92 | 0.73 |
| P07900 | Heat shock protein HSP 90-alpha | HSP90AA1 | 263 | S | 1 | 340 | 0.91 | 0.94 | 0.91 | 1.02 | 1.00 | 1.05 | 0.98 | 0.94 | 0.99 |
| P08238 | Heat shock protein HSP 90-beta;Putative heat shock protein HSP 90-beta-3 | HSP90AB1 | 226 | S | 1 | 157.4 | 0.89 | 0.84 | 0.99 | 0.76 | 0.83 | 0.87 | 1.10 | 1.07 | 1.36 |
| P08238 | Heat shock protein HSP 90-beta;Putative heat shock protein HSP 90-beta 2 | HSP90AB1 | 255 | S | 1 | 332.4 | 0.91 | 0.95 | 0.86 | 0.89 | 0.90 | 0.89 | 0.95 | 0.93 | 0.99 |

| | | | | | | | | | | | | | | |
|---|---|---|---|---|---|---|---|---|---|---|---|---|---|---|
| P08238 | Heat shock protein HSP 90-beta;Putative heat shock protein HSP 90-beta 2 | HSP90AB 1 | 261 | S | 1 | 306.1 | 0.87 | 1.16 | NaN | 0.87 | 1.04 | 0.88 | 0.86 | 1.01 | NaN |
| P08240 | Signal recognition particle receptor subunit alpha | SRPR | 296 | S | 0.987 | 118.5 | NaN | NaN | 0.49 | NaN | NaN | NaN | 0.90 | NaN | 1.01 |
| P08240 | Signal recognition particle receptor subunit alpha | SRPR | 297 | S | 0.842 | 118.5 | NaN | NaN | 0.49 | NaN | NaN | NaN | 0.90 | NaN | 1.01 |
| P08240 | Signal recognition particle receptor subunit alpha | SRPR | 298 | S | 0.667 | 115.7 | NaN | NaN | 0.49 | NaN | NaN | NaN | 0.90 | NaN | 1.01 |
| P08621 | U1 small nuclear ribonucleoprotein 70 kDa | SNRNP70 | 226 | S | 0.973 | 45.68 | NaN | NaN | NaN | NaN | 0.97 | NaN | 1.43 | 1.50 | 1.84 |
| P08670 | Vimentin | VIM | 26 | S | 0.809 | 109 | NaN | NaN | NaN | NaN | NaN | NaN | NaN | 0.87 | 0.84 |
| P08670 | Vimentin | VIM | 214 | S | 1 | 59.98 | NaN | NaN | NaN | 1.39 | 1.47 | NaN | NaN | NaN | NaN |
| P08670 | Vimentin | VIM | 325 | S | 1 | 134.9 | 1.32 | 1.48 | NaN | 1.91 | 1.19 | NaN | 2.12 | 1.51 | 1.30 |
| P08670 | Vimentin | VIM | 339 | S | 0.901 | 170 | 1.08 | 1.09 | NaN | NaN | NaN | 0.88 | NaN | NaN | NaN |
| P08670 | Vimentin | VIM | 55 | S | 0.792 | 147.1 | NaN | 1.20 | 1.20 | NaN | NaN | NaN | NaN | NaN | NaN |
| P08670 | Vimentin | VIM | 56 | S | 0.991 | 157.4 | 1.08 | NaN | NaN | 0.99 | 0.97 | 1.12 | 0.54 | 0.46 | 0.54 |
| P0C1Z6 | TCF3 fusion partner | TFPT | 249 | S | 1 | 104.6 | 0.82 | 0.76 | NaN | 1.18 | 0.92 | 0.72 | 1.14 | 1.05 | 1.01 |
| P0C1Z6 | TCF3 fusion partner | TFPT | 252 | S | 1 | 104.6 | 0.82 | 0.79 | NaN | 1.18 | 0.92 | 0.72 | 1.14 | 1.05 | 1.01 |
| Q96II5 | Serine/threonine-protein kinase A-Raf | ARAF | 260 | S | 1 | 77.64 | NaN | NaN | 1.03 | NaN | NaN | 1.03 | 1.10 | 1.23 | 1.58 |
| Q96II5 | Serine/threonine-protein kinase A-Raf;RAF proto-oncogene serine/threonine- | ARAF | 585 | S | 1 | 85.82 | 0.91 | 0.93 | NaN | NaN | 1.19 | 1.16 | NaN | 2.19 | 2.07 |
| P11166 | Solute carrier family 2, facilitated glucose transporter member 1 | SLC2A1 | 473 | S | 0.688 | 64.25 | NaN | NaN | NaN | NaN | NaN | NaN | 3.04 | 2.97 | NaN |
| P11274 | Breakpoint cluster region protein | BCR | 122 | S | 1 | 107.7 | 0.75 | 0.79 | 0.72 | 0.86 | 0.88 | NaN | 0.73 | NaN | 0.74 |
| P11388 | DNA topoisomerase 2-alpha | TOP2A | 1106 | S | 1 | 220.9 | 0.83 | 0.84 | 0.84 | 1.35 | 1.78 | 1.59 | 1.01 | 1.02 | 1.04 |
| P11388 | DNA topoisomerase 2-alpha | TOP2A | 1393 | S | 0.968 | 222.6 | 1.07 | 1.08 | 1.12 | 2.04 | 2.17 | 2.05 | 1.01 | NaN | 0.96 |
| P11388 | DNA topoisomerase 2-alpha | TOP2A | 1247 | S | 1 | 178.2 | 1.77 | 2.47 | 2.43 | 5.01 | 4.62 | 3.56 | 1.08 | 0.88 | NaN |
| P11388 | DNA topoisomerase 2-alpha | TOP2A | 1377 | S | 1 | 173.4 | 1.69 | NaN | 1.68 | 2.54 | 2.23 | 2.56 | 1.33 | 1.29 | 1.34 |
| P11388 | DNA topoisomerase 2-alpha | TOP2A | 1525 | S | 1 | 173.3 | 1.21 | 1.22 | 1.27 | 1.95 | NaN | 1.99 | 1.00 | 1.06 | 1.00 |
| P11717 | Cation-independent mannose-6-phosphate receptor | IGF2R | 2409 | S | 1 | 224.4 | 1.22 | 1.28 | 1.32 | 1.31 | 1.39 | 1.20 | 1.10 | 1.09 | 1.11 |
| P11717 | Cation-independent mannose-6-phosphate receptor | IGF2R | 2484 | S | 1 | 331.7 | 1.05 | 1.06 | 1.10 | 1.07 | 1.09 | 1.03 | 1.00 | 0.98 | 1.02 |
| P11831 | Serum response factor | SRF | 224 | S | 0.998 | 275.9 | 1.23 | 1.42 | 0.80 | 1.03 | 1.09 | 1.07 | 1.22 | 1.30 | 1.27 |
| P12931 | Proto-oncogene tyrosine-protein kinase Src | SRC | 17 | S | 1 | 185.2 | NaN | 1.08 | 0.81 | 1.04 | 1.05 | 0.53 | 1.11 | 1.17 | 1.00 |
| P13051 | Uracil-DNA glycosylase | UNG | 23 | S | 1 | 107.2 | 0.42 | NaN | NaN | NaN | NaN | NaN | 0.65 | 0.61 | NaN |
| P13861 | cAMP-dependent protein kinase type II-alpha regulatory | PRKAR2A | 78 | S | 1 | 254.9 | 1.00 | 0.34 | 0.35 | 0.33 | 0.30 | 0.51 | 0.67 | 1.20 | 1.22 |
| P13861 | cAMP-dependent protein kinase type II-alpha regulatory | PRKAR2A | 80 | S | 1 | 254.9 | 1.00 | 1.02 | 1.01 | 0.86 | 0.86 | 0.92 | 1.12 | 0.61 | 0.78 |

| | | | | | | | | | | | | | | | |
|---|---|---|---|---|---|---|---|---|---|---|---|---|---|---|---|
| P13861 | cAMP-dependent protein kinase type II-alpha regulatory | PRKAR2A | 99 | S | 1 | 123.6 | 1.60 | 1.37 | NaN | 2.34 | NaN | 2.06 | 1.64 | 1.48 | 1.31 |
| P13994 | Coiled-coil domain-containing protein 130 | CCDC130 | 362 | S | 0.995 | 88.87 | 0.56 | 0.54 | 0.45 | 0.65 | 0.66 | NaN | 0.67 | 0.89 | 0.81 |
| P14625 | Endoplasmin | HSP90B1 | 306 | S | 1 | 235.2 | 1.07 | 0.52 | 0.80 | 0.82 | 0.84 | 0.80 | NaN | NaN | 0.52 |
| Q86XA6 | Beta-1,4-galactosyltransferase 1;Lactose synthase A protein;N-acetyllactosamine synthase;Beta-N-acetylglucosaminylglycopeptide beta-1,4-galactosyltransferase;Beta-N-acetylglucosaminyl-glycolipid beta-1,4-galactosyltransferase;Processed beta-1,4- | B4GALT1 | 74 | S | 0.882 | 149 | NaN | NaN | NaN | NaN | NaN | NaN | NaN | 0.81 | 0.94 |
| P15407 | Fos-related antigen 1 | FOSL1 | 101 | S | 1 | 76 | NaN | NaN | NaN | 4.13 | 4.30 | NaN | NaN | NaN | NaN |
| P16383 | GC-rich sequence DNA-binding factor 2 | GCFC2 | 429 | S | 0.898 | 106.2 | 0.81 | 0.79 | 0.81 | NaN | 0.89 | 0.85 | 0.53 | 0.56 | 0.58 |
| P16383 | GC-rich sequence DNA-binding factor 2 | GCFC2 | 430 | S | 0.944 | 106.2 | 0.81 | 0.79 | 0.81 | NaN | 0.78 | 0.76 | 0.53 | 0.56 | 0.58 |
| P16989 | Y-box-binding protein 3 | YBX3 | 38 | S | 0.603 | 178.8 | 1.07 | 1.71 | 1.25 | NaN | NaN | 1.45 | NaN | NaN | 1.24 |
| P17096 | High mobility group protein HMG-I/HMG-Y | HMGA1 | 99 | S | 1 | 255.6 | 1.18 | 1.15 | 1.06 | 1.01 | 0.62 | 1.01 | 0.94 | 0.63 | 0.97 |
| P17096 | High mobility group protein HMG-I/HMG-Y | HMGA1 | 102 | S | 1 | 255.6 | 0.59 | 0.56 | 0.57 | 0.68 | 0.62 | 0.37 | 0.92 | 0.91 | 0.99 |
| P17096 | High mobility group protein HMG-I/HMG-Y | HMGA1 | 103 | S | 1 | 255.6 | 0.59 | 0.56 | 0.57 | 0.68 | 0.63 | 0.59 | 0.92 | 0.90 | 0.58 |
| P17096 | High mobility group protein HMG-I/HMG-Y | HMGA1 | 36 | S | 0.999 | 129.1 | 3.37 | 3.42 | 3.15 | 6.22 | 6.04 | 5.84 | 0.95 | 0.96 | 0.91 |
| P17252 | Protein kinase C alpha type | PRKCA | 226 | S | 0.908 | 170 | 1.78 | 2.11 | NaN | 1.62 | 1.60 | NaN | 0.76 | 0.68 | 0.60 |
| P17252 | Protein kinase C alpha type | PRKCA | 319 | S | 0.985 | 81.53 | NaN | NaN | 1.82 | 2.29 | 2.28 | NaN | 1.28 | 1.40 | 1.44 |
| P17275 | Transcription factor jun-B | JUNB | 259 | S | 1 | 140.3 | 4.31 | NaN | NaN | 0.99 | NaN | 1.22 | NaN | 5.63 | 5.63 |
| P17535 | Transcription factor jun-D | JUND | 255 | S | 0.997 | 111.6 | 0.13 | 0.40 | 0.32 | 0.50 | 0.52 | 0.39 | 0.36 | 0.45 | 0.19 |
| P17535 | Transcription factor jun-D | JUND | 259 | S | 1 | 107 | 0.39 | 0.40 | 0.32 | 0.95 | 0.94 | 0.82 | 0.36 | 0.35 | 0.36 |
| P18031 | Tyrosine-protein phosphatase non-receptor type 1 | PTPN1 | 50 | S | 1 | 68.11 | 1.74 | 1.32 | 1.69 | NaN | 1.30 | 1.10 | 1.11 | 1.42 | 1.37 |
| P18206 | Vinculin | VCL | 290 | S | 1 | 126.2 | 0.95 | 1.01 | 0.89 | 1.07 | 1.04 | 0.99 | 0.96 | 1.05 | 1.00 |
| P18206 | Vinculin | VCL | 346 | S | 0.999 | 177.4 | 0.80 | 2.57 | 1.13 | 2.13 | 2.10 | NaN | NaN | 1.09 | NaN |
| P18206 | Vinculin | VCL | 721 | S | 1 | 99.07 | 2.83 | 3.23 | 3.29 | 3.08 | 3.19 | 2.98 | 1.91 | 1.63 | 1.92 |
| P18206 | Vinculin | VCL | 820 | S | 0.92 | 81.55 | 1.18 | NaN | 1.28 | 1.31 | 1.34 | 1.21 | 1.06 | NaN | 1.08 |
| P18583 | Protein SON | SON | 1697 | S | 1 | 152.3 | 1.75 | 1.62 | 1.64 | 1.27 | 1.45 | 1.31 | 1.18 | 1.26 | 1.22 |
| P18583 | Protein SON | SON | 1556 | S | 0.998 | 219.8 | 1.02 | 1.00 | 0.92 | 1.00 | 0.92 | 1.17 | 0.78 | 0.76 | 0.80 |
| P18583 | Protein SON | SON | 2129 | S | 0.905 | 43.35 | 1.08 | 1.30 | NaN | NaN | NaN | NaN | NaN | NaN | NaN |
| P18583 | Protein SON | SON | 2011 | S | 1 | 59.29 | 0.57 | 1.50 | NaN | NaN | 1.14 | NaN | NaN | NaN | NaN |
| P18583 | Protein SON | SON | 2013 | S | 1 | 74.53 | NaN | 0.46 | NaN | 0.60 | 0.66 | 0.82 | NaN | 0.41 | NaN |

| | | | | | | | | | | | | | | | |
|---|---|---|---|---|---|---|---|---|---|---|---|---|---|---|---|
| P18583 | Protein SON | SON | 283 | S | 0.998 | 187.1 | 1.27 | NaN | 1.18 | NaN | 1.26 | 1.24 | 1.01 | 1.05 | NaN |
| P18669 | Phosphoglycerate mutase 1 | PGAM1 | 31 | S | 1 | 89.51 | 1.06 | 0.90 | NaN | 1.15 | 1.27 | NaN | 0.93 | NaN | NaN |
| P18669 | Phosphoglycerate mutase 1;Probable phosphoglycerate mutase 4 | PGAM1 | 118 | S | 1 | 124.3 | 0.81 | 0.79 | 0.82 | 0.95 | 1.00 | 0.98 | 0.69 | 0.67 | 0.71 |
| P18858 | DNA ligase 1;DNA ligase | LIG1 | 91 | S | 0.975 | 87.9 | 0.71 | NaN | NaN | NaN | 0.71 | 0.69 | NaN | NaN | NaN |
| P18858 | DNA ligase 1;DNA ligase | LIG1 | 98 | S | 0.816 | 78.65 | 0.71 | NaN | NaN | NaN | 0.71 | 0.69 | NaN | NaN | NaN |
| P18858 | DNA ligase 1;DNA ligase | LIG1 | 911 | S | 0.994 | 94.06 | NaN | NaN | NaN | 1.28 | 1.04 | NaN | NaN | NaN | NaN |
| P18858 | DNA ligase 1;DNA ligase | LIG1 | 913 | S | 0.994 | 94.06 | NaN | NaN | NaN | 1.28 | 1.04 | NaN | NaN | NaN | NaN |
| P18858 | DNA ligase 1;DNA ligase | LIG1 | 141 | S | 1 | 165.5 | 0.94 | NaN | 6.77 | NaN | NaN | NaN | NaN | NaN | NaN |
| P18858 | DNA ligase 1;DNA ligase | LIG1 | 66 | S | 1 | 244.7 | 0.77 | 0.69 | 0.72 | 0.48 | 0.47 | 0.45 | 0.77 | 0.82 | 0.76 |
| P18858 | DNA ligase 1;DNA ligase | LIG1 | 76 | S | 1 | 244.7 | 0.35 | 0.45 | 0.41 | 0.76 | 0.60 | 0.59 | 0.48 | 0.52 | 0.58 |
| P19634 | Sodium/hydrogen exchanger 1 | SLC9A1 | 703 | S | 1 | 102.7 | NaN | NaN | NaN | NaN | NaN | NaN | NaN | 1.42 | 1.51 |
| P19634 | Sodium/hydrogen exchanger 1 | SLC9A1 | 693 | S | 0.996 | 54.54 | 0.98 | 1.07 | NaN | 0.88 | NaN | 1.09 | 1.19 | 1.10 | 1.14 |
| P19634 | Sodium/hydrogen exchanger 1 | SLC9A1 | 785 | S | 0.864 | 111.8 | 0.82 | 0.83 | 0.79 | NaN | NaN | 0.71 | 1.05 | 1.16 | 0.83 |
| P19838 | Nuclear factor NF-kappa-B p105 subunit;Nuclear factor NF-kappa-B p50 subunit | NFKB1 | 893 | S | 0.974 | 77.89 | 0.97 | 0.85 | NaN | NaN | 1.30 | NaN | NaN | 0.57 | NaN |
| P20700 | Lamin-B1 | LMNB1 | 23 | S | 0.937 | 107.5 | NaN | 1.96 | 1.69 | NaN | NaN | NaN | NaN | NaN | 0.75 |
| Q03252 | Lamin-B1;Lamin-B2 | LMNB1 | 391;405 | S | 0.998 | 84.37 | 0.53 | 0.59 | 0.62 | 0.90 | 0.96 | 0.89 | 0.92 | 0.86 | 1.16 |
| Q03252 | Lamin-B1;Lamin-B2 | LMNB1 | 393;407 | S | 0.993 | 84.37 | 0.42 | 0.45 | NaN | 0.66 | 0.64 | 0.64 | 1.11 | 0.83 | 1.12 |
| Q03252 | Lamin-B1;Lamin-B2 | LMNB1 | 396;410 | S | 0.999 | 77.63 | 1.79 | 1.30 | 1.49 | 2.43 | 2.64 | NaN | NaN | 0.69 | 1.09 |
| Q60FE5 | Filamin-A | FLNA | 2125 | S | 1 | 146.3 | 1.30 | 1.33 | 1.31 | 1.29 | 1.31 | NaN | 1.19 | 1.12 | 1.08 |
| Q60FE5 | Filamin-A | FLNA | 1432 | S | 1 | 159 | 0.75 | 0.79 | 0.71 | 1.32 | 1.32 | 1.26 | 0.84 | 0.83 | 0.83 |
| Q60FE5 | Filamin-A | FLNA | 2300 | S | 0.961 | 78.31 | 1.64 | 1.78 | NaN | NaN | 2.11 | NaN | NaN | 0.90 | NaN |
| Q60FE5 | Filamin-A | FLNA | 2153 | S | 0.938 | 141.9 | 1.16 | 1.17 | 1.22 | 1.51 | 1.30 | 1.39 | 0.78 | 0.84 | 0.81 |
| P22059 | Oxysterol-binding protein 1 | OSBP | 351 | S | 1 | 270.8 | 0.48 | 0.48 | 0.51 | 0.85 | 0.92 | 0.89 | 0.45 | 0.36 | 0.47 |
| P22059 | Oxysterol-binding protein 1 | OSBP | 190 | S | 1 | 211 | 0.85 | 0.80 | 0.80 | 0.90 | 1.12 | 0.90 | 0.75 | 0.84 | 0.77 |
| P22059 | Oxysterol-binding protein 1 | OSBP | 193 | S | 1 | 211 | 0.85 | 0.80 | 0.80 | 0.90 | 1.12 | 0.90 | 0.75 | 0.84 | 0.77 |
| P22059 | Oxysterol-binding protein 1 | OSBP | 240 | S | 0.998 | 76.42 | 3.25 | 3.21 | NaN | NaN | NaN | NaN | NaN | NaN | NaN |
| Q5JRR6 | Ubiquitin-like modifier-activating enzyme 1 | UBA1 | 283 | S | 1 | 96.67 | 0.94 | 0.94 | 0.84 | 1.19 | 1.08 | 1.20 | 0.75 | 1.08 | 1.02 |
| P22626 | Heterogeneous nuclear ribonucleoproteins A2/B1 | HNRNPA2B1 | 259 | S | 1 | 131.4 | 1.60 | NaN | 1.75 | 2.04 | 2.34 | 2.21 | NaN | 1.17 | 1.10 |
| P22626 | Heterogeneous nuclear ribonucleoproteins A2/B1 | HNRNPA2B1 | 344 | S | 0.872 | 78.32 | NaN | NaN | 1.62 | 2.08 | 2.73 | NaN | NaN | NaN | NaN |
| P23381 | Tryptophan--tRNA ligase, cytoplasmic;T1-TrpRS;T2-TrpRS | WARS | 467 | S | 1 | 76.83 | 0.89 | 0.79 | 1.23 | 0.82 | 0.80 | NaN | 1.04 | 0.70 | NaN |
| P24928 | DNA-directed RNA polymerase II subunit RPB1 | POLR2A | 1913 | S | 0.922 | 83.69 | 0.52 | 0.54 | 0.63 | NaN | NaN | NaN | NaN | NaN | 0.70 |
| P24928 | DNA-directed RNA polymerase II subunit RPB1 | POLR2A | 1920 | S | 0.767 | 101.5 | NaN | 0.84 | 0.63 | NaN | NaN | NaN | NaN | NaN | NaN |
| P24928 | DNA-directed RNA polymerase II subunit RPB1 | POLR2A | 1878 | S | 0.994 | 110.5 | 0.94 | 0.80 | NaN | NaN | 0.92 | NaN | NaN | 0.98 | 0.90 |
| P26038 | Moesin | MSN | 576 | S | 1 | 105 | 0.77 | 0.63 | NaN | 1.34 | 1.17 | 1.00 | NaN | NaN | NaN |

| | | | | | | | | | | | | | | | |
|---|---|---|---|---|---|---|---|---|---|---|---|---|---|---|---|
| P26358 | DNA (cytosine-5)-methyltransferase 1 | DNMT1 | 714 | S | 1 | 259.6 | 0.26 | 0.25 | 0.27 | 0.52 | 0.55 | 0.49 | 0.34 | 0.33 | 0.34 |
| P26358 | DNA (cytosine-5)-methyltransferase 1 | DNMT1 | 127 | S | 1 | 72.17 | NaN | NaN | NaN | 0.87 | 0.68 | 0.96 | NaN | NaN | NaN |
| P26368 | Splicing factor U2AF 65 kDa subunit | U2AF2 | 79 | S | 1 | 111.4 | 0.95 | 1.00 | 1.04 | 1.08 | 1.08 | 1.01 | 0.88 | 0.91 | 0.90 |
| P27816 | Microtubule-associated protein 4;Microtubule-associated protein | MAP4 | 358 | S | 1 | 127.2 | 0.58 | 0.56 | 0.60 | 1.04 | 1.06 | 1.05 | 0.62 | 0.64 | 0.67 |
| P27816 | Microtubule-associated protein 4;Microtubule-associated protein | MAP4 | 787 | S | 1 | 95.03 | 1.07 | 1.09 | 1.06 | 1.84 | 1.79 | 1.88 | 0.84 | 0.82 | 0.83 |
| P27816 | Microtubule-associated protein 4;Microtubule-associated protein | MAP4 | 789 | S | 0.842 | 74.85 | NaN | NaN | NaN | 2.07 | 1.81 | 1.99 | 0.82 | 0.74 | 0.71 |
| P27816 | Microtubule-associated protein 4;Microtubule-associated protein | MAP4 | 280 | S | 1 | 223.3 | 0.54 | 0.53 | 0.49 | 0.73 | 0.75 | 0.76 | 0.58 | 0.53 | 0.56 |
| P27816 | Microtubule-associated protein 4;Microtubule-associated protein | MAP4 | 507 | S | 1 | 279.1 | 0.30 | 0.32 | 0.36 | 0.64 | 0.70 | 0.69 | 0.27 | 0.31 | 0.26 |
| P27816 | Microtubule-associated protein 4;Microtubule-associated protein | MAP4 | 510 | S | 0.998 | 150.1 | 0.74 | 0.74 | 0.94 | 1.70 | 1.86 | 1.50 | 0.67 | 0.33 | 0.62 |
| P27816 | Microtubule-associated protein 4;Microtubule-associated protein | MAP4 | 696 | S | 1 | 87.48 | 1.01 | 1.07 | 1.08 | 1.58 | 1.58 | 1.73 | 0.64 | 0.62 | 0.61 |
| P27816 | Microtubule-associated protein 4;Microtubule-associated protein | MAP4 | 624 | S | 0.919 | 106.8 | NaN | NaN | NaN | 0.91 | 1.13 | 1.52 | 0.66 | NaN | 0.77 |
| P27816 | Microtubule-associated protein 4;Microtubule-associated protein | MAP4 | 636 | S | 1 | 128.4 | 0.70 | 0.79 | 0.80 | 2.03 | 1.75 | 1.76 | 1.05 | 0.91 | 0.87 |
| P27816 | Microtubule-associated protein 4;Microtubule-associated protein | MAP4 | 1073 | S | 1 | 133.9 | 0.65 | 0.63 | 0.64 | 0.64 | 0.58 | 0.58 | 2.06 | 2.09 | 1.92 |
| P27824 | Calnexin | CANX | 583 | S | 1 | 367.3 | 1.21 | 1.19 | 1.01 | 1.08 | 1.10 | 1.08 | 1.01 | 0.97 | 1.06 |
| P27824 | Calnexin | CANX | 554 | S | 1 | 430.6 | 0.82 | 0.84 | 0.79 | 0.86 | 0.82 | 0.81 | 0.91 | 0.87 | 0.95 |
| P27824 | Calnexin | CANX | 564 | S | 1 | 298.7 | 0.91 | 0.98 | 0.95 | 0.80 | 0.81 | 0.75 | 0.97 | 1.04 | 1.52 |
| P28066 | Proteasome subunit alpha type-5 | PSMA5 | 16 | S | 0.998 | 111 | 4.12 | 4.16 | 3.85 | 6.45 | 4.84 | 4.89 | 1.17 | 0.90 | 1.05 |
| P28066 | Proteasome subunit alpha type-5 | PSMA5 | 56 | S | 0.974 | 70.63 | NaN | NaN | NaN | NaN | NaN | NaN | NaN | 0.54 | 1.06 |
| P28290 | Sperm-specific antigen 2 | SSFA2 | 641 | S | 1 | 122.9 | 0.74 | 0.81 | 0.85 | 0.89 | 0.88 | 0.83 | 1.14 | 1.25 | 1.17 |
| P28290 | Sperm-specific antigen 2 | SSFA2 | 591 | S | 0.543 | 119.3 | 1.04 | 0.78 | 0.93 | NaN | 0.90 | 0.79 | 1.23 | NaN | 1.33 |
| P28290 | Sperm-specific antigen 2 | SSFA2 | 593 | S | 0.764 | 119.3 | 1.15 | 0.92 | 0.93 | 1.10 | 1.12 | 1.16 | 1.23 | 1.14 | 1.33 |
| P28290 | Sperm-specific antigen 2 | SSFA2 | 668 | S | 0.998 | 97.73 | 1.37 | 1.56 | 1.50 | 1.19 | NaN | 1.22 | 2.69 | 2.03 | 2.42 |

| | | | | | | | | | | | | | | | |
|---|---|---|---|---|---|---|---|---|---|---|---|---|---|---|---|
| P28290 | Sperm-specific antigen 2 | SSFA2 | 739 | S | 1 | 142.5 | 2.39 | NaN | 0.90 | 0.79 | 0.55 | NaN | 2.01 | NaN | 1.86 |
| P29317 | Ephrin type-A receptor 2 | EPHA2 | 901 | S | 0.913 | 48.28 | NaN | NaN | 1.49 | NaN | NaN | 1.31 | 1.23 | 1.14 | NaN |
| P29590 | Protein PML | PML | 36 | S | 0.998 | 112.3 | 0.70 | 0.68 | 0.95 | NaN | 0.68 | NaN | NaN | 1.36 | 1.34 |
| P29966 | Myristoylated alanine-rich C-kinase substrate | MARCKS | 145 | S | 1 | 289.1 | 0.63 | 0.63 | 0.67 | 0.68 | 0.67 | 0.68 | 1.12 | 1.15 | 1.09 |
| P29966 | Myristoylated alanine-rich C-kinase substrate | MARCKS | 147 | S | 0.879 | 204.5 | NaN | 3.23 | 2.16 | 1.74 | 1.70 | 3.55 | 2.15 | 1.83 | 3.63 |
| P29966 | Myristoylated alanine-rich C-kinase substrate | MARCKS | 118 | S | 0.997 | 206.1 | 2.30 | 2.36 | 2.21 | 2.23 | 2.44 | 2.22 | 1.06 | 1.05 | 1.04 |
| P29966 | Myristoylated alanine-rich C-kinase substrate | MARCKS | 128 | S | 0.993 | 94.15 | 0.68 | 1.14 | 0.78 | 0.68 | NaN | NaN | NaN | NaN | NaN |
| P29966 | Myristoylated alanine-rich C-kinase substrate | MARCKS | 131 | S | 0.909 | 94.15 | 0.68 | 1.14 | 0.78 | 0.68 | NaN | NaN | NaN | 2.05 | NaN |
| P29966 | Myristoylated alanine-rich C-kinase substrate | MARCKS | 132 | S | 0.751 | 94.15 | NaN | 1.14 | 1.39 | 0.68 | NaN | NaN | NaN | 0.94 | NaN |
| P29966 | Myristoylated alanine-rich C-kinase substrate | MARCKS | 135 | S | 0.953 | 206.1 | 0.88 | 0.75 | 0.76 | 0.87 | 0.74 | 0.82 | 0.84 | 1.06 | 1.05 |
| P29966 | Myristoylated alanine-rich C-kinase substrate | MARCKS | 81 | S | 0.876 | 248.8 | 2.33 | 2.06 | NaN | 3.13 | NaN | 4.03 | NaN | NaN | 1.05 |
| P29966 | Myristoylated alanine-rich C-kinase substrate | MARCKS | 26 | S | 0.748 | 138.2 | NaN | NaN | 1.12 | NaN | 1.46 | 1.28 | 1.12 | 1.09 | NaN |
| P29966 | Myristoylated alanine-rich C-kinase substrate | MARCKS | 27 | S | 0.988 | 226.7 | 1.54 | 1.45 | 1.28 | 1.30 | NaN | 1.32 | 1.04 | NaN | 1.20 |
| P29966 | Myristoylated alanine-rich C-kinase substrate | MARCKS | 101 | S | 1 | 208.4 | 2.53 | 2.73 | 3.00 | 3.94 | 4.04 | 3.81 | 1.12 | 1.02 | 1.01 |
| P29966 | Myristoylated alanine-rich C-kinase substrate | MARCKS | 46 | S | 1 | 158.9 | 1.39 | 1.44 | 1.50 | 2.08 | 2.02 | 1.96 | 1.26 | 1.23 | 1.22 |
| P30050 | 60S ribosomal protein L12 | RPL12 | 38 | S | 1 | 72.29 | 1.37 | 1.43 | 1.57 | 2.30 | 2.08 | 2.02 | 0.50 | 0.45 | 0.46 |
| P30419 | Glycylpeptide N-tetradecanoyltransferase 1 | NMT1 | 47 | S | 1 | 160.9 | NaN | NaN | NaN | 1.15 | 1.09 | 1.06 | NaN | NaN | NaN |
| P31629 | Transcription factor HIVEP2 | HIVEP2 | 1443 | S | 0.998 | 83.69 | 0.40 | 0.49 | NaN | NaN | NaN | NaN | NaN | 0.41 | NaN |
| P31689 | DnaJ homolog subfamily A member 1 | DNAJA1 | 335 | S | 1 | 149.7 | 3.65 | 4.35 | 5.08 | 5.72 | 5.64 | 5.50 | 0.68 | 0.73 | 0.88 |
| P31749 | RAC-alpha serine/threonine-protein kinase | AKT1 | 122 | S | 0.544 | 74.4 | NaN | NaN | 0.99 | 0.86 | NaN | 0.95 | NaN | 0.85 | NaN |
| P31749 | RAC-alpha serine/threonine-protein kinase | AKT1 | 124 | S | 0.997 | 149.9 | 0.53 | 0.46 | 0.47 | 0.60 | 1.04 | 0.60 | 0.53 | 0.53 | 0.59 |
| P31749 | RAC-alpha serine/threonine-protein kinase | AKT1 | 126 | S | 0.989 | 74.4 | NaN | NaN | 0.99 | 0.86 | 1.04 | 0.95 | 0.83 | 0.85 | 0.84 |
| P31749 | RAC-alpha serine/threonine-protein kinase | AKT1 | 129 | S | 0.999 | 74.4 | NaN | NaN | 0.99 | 0.86 | 1.04 | 0.95 | 0.83 | 0.85 | 0.84 |
| P31942 | Heterogeneous nuclear ribonucleoprotein H3 | HNRNPH3 | 216 | S | 1 | 61.54 | 2.35 | 2.26 | NaN | NaN | 2.49 | NaN | NaN | NaN | NaN |
| P33981 | Dual specificity protein kinase TTK | TTK | 821 | S | 0.999 | 118.2 | 2.61 | 1.38 | NaN | 4.46 | 3.17 | 3.84 | NaN | NaN | NaN |
| P35251 | Replication factor C subunit 1 | RFC1 | 69 | S | 0.999 | 92.1 | 0.72 | NaN | NaN | NaN | 0.94 | 0.72 | 1.08 | NaN | 1.12 |
| P35251 | Replication factor C subunit 1 | RFC1 | 71 | S | 0.951 | 92.1 | 0.72 | NaN | NaN | NaN | 0.94 | 0.72 | 1.08 | NaN | 1.12 |

| | | | | | | | | | | | | | | |
|---|---|---|---|---|---|---|---|---|---|---|---|---|---|---|
| P35367 | Histamine H1 receptor | HRH1 | 275 | S | 0.825 | 78.38 | 0.66 | 4.14 | 4.02 | NaN | NaN | 2.47 | NaN | NaN | NaN |
| P35568 | Insulin receptor substrate 1 | IRS1 | 1078 | S | 1 | 83.78 | 0.77 | 0.78 | 0.64 | 0.97 | NaN | 1.04 | 0.86 | NaN | 0.83 |
| P35579 | Myosin-9 | MYH9 | 1943 | S | 1 | 362.9 | 0.81 | 0.83 | 0.76 | 0.82 | 0.81 | 0.80 | 1.02 | 1.04 | 1.03 |
| P35579 | Myosin-9 | MYH9 | 1713 | S | 0.5 | 75.54 | 1.10 | 1.14 | NaN | NaN | NaN | NaN | NaN | NaN | 2.16 |
| P35579 | Myosin-9 | MYH9 | 1714 | S | 0.5 | 75.54 | 1.10 | 1.14 | NaN | NaN | NaN | NaN | NaN | NaN | 2.16 |
| P35580 | Myosin-10 | MYH10 | 1952 | S | 1 | 98.8 | 0.73 | 0.74 | NaN | 0.77 | 0.82 | NaN | 1.30 | NaN | NaN |
| P35580 | Myosin-10 | MYH10 | 1956 | S | 1 | 282.6 | 0.80 | 0.85 | 0.81 | 0.81 | 0.84 | 0.77 | 0.93 | 0.92 | 0.87 |
| P36507 | Dual specificity mitogen-activated protein kinase kinase 2 | MAP2K2 | 293 | S | 0.746 | 134.6 | NaN | NaN | 1.68 | 1.78 | 1.87 | 1.81 | 0.93 | 0.99 | NaN |
| P36507 | Dual specificity mitogen-activated protein kinase kinase 2 | MAP2K2 | 295 | S | 0.994 | 207.4 | 1.74 | 1.90 | NaN | 1.89 | NaN | 1.81 | 0.99 | 1.08 | NaN |
| P38159 | RNA-binding motif protein, X chromosome;RNA-binding motif protein, X chromosome, N-terminally processed;RNA binding motif protein, X-linked-like-1 | RBMX | 208 | S | 1 | 112.1 | 0.81 | 0.80 | 0.80 | 0.93 | 0.96 | 0.95 | 0.85 | 0.85 | 0.87 |
| P38159 | RNA-binding motif protein, X chromosome;RNA-binding motif protein, X chromosome, N-terminally processed | RBMX | 352 | S | 1 | 66.6 | NaN | 0.78 | NaN | 0.93 | NaN | 0.81 | NaN | 1.05 | 0.87 |
| P38432 | Coilin | COIL | 566 | S | 0.934 | 82.2 | NaN | NaN | NaN | 1.54 | NaN | 1.05 | 0.98 | 0.83 | 0.97 |
| P40222 | Alpha-taxilin | TXLNA | 515 | S | 0.993 | 124.5 | 1.53 | 1.65 | 1.63 | 1.48 | 1.46 | 1.53 | 1.06 | 1.05 | 1.11 |
| P40222 | Alpha-taxilin | TXLNA | 19 | S | 0.788 | 119.8 | 1.44 | 1.10 | NaN | NaN | 2.06 | NaN | NaN | NaN | NaN |
| P40818 | Ubiquitin carboxyl-terminal hydrolase 8 | USP8 | 718 | S | 0.99 | 115.4 | NaN | 2.28 | 1.92 | 2.07 | 2.10 | 1.86 | 1.77 | 2.10 | 2.29 |
| P41208 | Centrin-2 | CETN2 | 20 | S | 1 | 62.53 | 1.04 | 0.78 | NaN | 1.11 | 1.18 | NaN | NaN | NaN | NaN |
| P41227 | N-alpha-acetyltransferase 10 | NAA10 | 205 | S | 0.999 | 197.6 | 0.80 | 0.95 | 0.95 | 2.05 | 1.30 | 0.96 | NaN | NaN | 0.83 |
| P41227 | N-alpha-acetyltransferase 10 | NAA10 | 209 | S | 0.585 | 169.2 | NaN | NaN | NaN | NaN | NaN | NaN | 0.95 | 1.00 | NaN |
| P41227 | N-alpha-acetyltransferase 10 | NAA10 | 186 | S | 1 | 85.36 | 0.97 | 0.90 | 1.02 | 1.16 | 1.29 | 1.21 | 0.88 | 0.77 | 0.91 |
| P42684 | Abelson tyrosine-protein kinase 2 | ABL2 | 631 | S | 0.972 | 51.01 | 0.80 | 0.85 | 0.94 | 1.09 | 1.00 | NaN | 0.89 | 0.89 | NaN |
| P42684 | Abelson tyrosine-protein kinase 2 | ABL2 | 936 | S | 0.987 | 70.49 | 1.60 | 1.58 | NaN | NaN | NaN | NaN | NaN | NaN | NaN |
| P42858 | Huntingtin | HTT | 1199 | S | 0.996 | 44.42 | 1.00 | NaN | NaN | NaN | 1.04 | NaN | 0.82 | 0.75 | 0.77 |
| P42858 | Huntingtin | HTT | 432 | S | 0.977 | 123.5 | 0.92 | 0.81 | 0.96 | 0.83 | 0.82 | 0.93 | 1.13 | 1.20 | 1.39 |
| P43490 | Nicotinamide phosphoribosyltransferase | NAMPT | 472 | S | 1 | 90.15 | 1.77 | 1.35 | 1.39 | 2.45 | 2.00 | 2.05 | 1.09 | 1.09 | 1.03 |
| P46013 | Antigen KI-67 | MKI67 | 827 | S | 0.998 | 70.94 | NaN | NaN | 0.58 | 1.27 | 1.48 | 1.66 | NaN | NaN | NaN |
| P46013 | Antigen KI-67 | MKI67 | 2105 | S | 1 | 62.66 | NaN | NaN | NaN | NaN | 1.68 | 2.16 | NaN | NaN | NaN |
| P46013 | Antigen KI-67 | MKI67 | 1131 | S | 1 | 84.13 | 0.54 | 0.54 | 0.75 | 1.95 | 1.92 | NaN | 0.62 | 0.55 | 0.58 |
| P46013 | Antigen KI-67 | MKI67 | 1861 | S | 0.998 | 71.69 | NaN | NaN | NaN | 1.75 | 2.04 | 1.59 | NaN | NaN | NaN |
| P46013 | Antigen KI-67 | MKI67 | 1376 | S | 0.826 | 133.9 | NaN | NaN | NaN | NaN | 1.52 | 2.07 | NaN | NaN | NaN |
| P46013 | Antigen KI-67 | MKI67 | 308 | S | 1 | 117.1 | 0.49 | 0.45 | 0.47 | 1.02 | 1.04 | 1.13 | 0.68 | 0.72 | 0.66 |
| P46013 | Antigen KI-67 | MKI67 | 357 | S | 1 | 80.6 | NaN | NaN | NaN | NaN | 1.25 | 1.60 | NaN | 0.59 | NaN |

| | | | | | | | | | | | | | | | |
|---|---|---|---|---|---|---|---|---|---|---|---|---|---|---|---|
| P46060 | Ran GTPase-activating protein 1 | RANGAP1 | 428 | S | 0.851 | 73.03 | 1.58 | NaN | 1.40 | NaN | 3.07 | 2.97 | 0.77 | 0.82 | 0.68 |
| P46060 | Ran GTPase-activating protein 1 | RANGAP1 | 442 | S | 1 | 92.82 | 0.55 | NaN | 0.44 | 0.86 | 0.80 | 0.86 | 0.73 | 0.82 | 0.88 |
| P46087 | Probable 28S rRNA (cytosine(4447)-C(5))-methyltransferase | NOP2 | 786 | S | 0.849 | 85.4 | NaN | 1.42 | 1.33 | NaN | NaN | 1.97 | NaN | NaN | NaN |
| P46087 | Probable 28S rRNA (cytosine(4447)-C(5))-methyltransferase | NOP2 | 732 | S | 0.975 | 106.5 | 1.23 | 0.85 | 1.37 | 1.39 | 1.55 | 1.57 | NaN | NaN | NaN |
| P46100 | Transcriptional regulator | ATRX | 849 | S | 1 | 77.53 | 0.51 | 0.49 | 0.57 | 0.65 | 0.64 | 0.65 | 0.82 | 0.91 | 0.90 |
| P46100 | Transcriptional regulator | ATRX | 850 | S | 1 | 77.53 | 0.51 | 0.49 | 0.57 | 0.65 | 0.64 | 0.65 | 0.82 | 0.91 | 0.90 |
| P46100 | Transcriptional regulator | ATRX | 1527 | S | 0.984 | 109.9 | 0.25 | NaN | NaN | 0.83 | 0.79 | 0.74 | 0.53 | 0.51 | 0.57 |
| P46100 | Transcriptional regulator | ATRX | 594 | S | 0.631 | 59.16 | NaN | NaN | NaN | 0.91 | 1.10 | NaN | 0.67 | 0.72 | NaN |
| P46100 | Transcriptional regulator | ATRX | 598 | S | 0.741 | 72.79 | 0.54 | 0.59 | NaN | NaN | NaN | NaN | 0.86 | NaN | NaN |
| P46100 | Transcriptional regulator | ATRX | 729 | S | 1 | 179.1 | 0.45 | 0.76 | 0.47 | 0.62 | 0.67 | 0.63 | 0.58 | 0.60 | 0.69 |
| P46100 | Transcriptional regulator | ATRX | 731 | S | 1 | 179.1 | 0.45 | 0.38 | 0.47 | 0.62 | 0.67 | 0.63 | 0.58 | 0.60 | 0.69 |
| P46100 | Transcriptional regulator | ATRX | 675 | S | 0.869 | 108.5 | NaN | NaN | NaN | NaN | NaN | NaN | NaN | 0.66 | 0.63 |
| P46100 | Transcriptional regulator | ATRX | 677 | S | 0.999 | 108.5 | 0.61 | 0.55 | NaN | NaN | 0.71 | NaN | 0.81 | NaN | 0.63 |
| P46100 | Transcriptional regulator | ATRX | 92 | S | 1 | 250.1 | 0.53 | 0.29 | 0.64 | 0.51 | 0.71 | 0.63 | 0.53 | 0.61 | 0.48 |
| P46821 | Microtubule-associated protein 1B;MAP1B heavy chain;MAP1 light chain LC1 | MAP1B | 541 | S | 0.971 | 78.72 | NaN | 0.48 | 0.55 | 0.45 | 0.37 | 0.44 | 1.27 | 1.23 | 1.28 |
| P46821 | Microtubule-associated protein 1B;MAP1B heavy chain;MAP1 light chain LC1 | MAP1B | 1016 | S | 1 | 157.4 | NaN | 0.45 | 0.55 | 0.82 | 0.64 | 0.64 | 1.04 | 0.93 | 0.93 |
| P46821 | Microtubule-associated protein 1B;MAP1B heavy chain;MAP1 light chain LC1 | MAP1B | 1376 | S | 0.561 | 116.4 | 0.49 | 0.47 | 0.56 | 0.54 | 0.60 | 0.67 | 0.37 | 0.34 | 0.49 |
| P46821 | Microtubule-associated protein 1B;MAP1B heavy chain;MAP1 light chain LC1 | MAP1B | 1378 | S | 1 | 272.3 | 0.54 | 0.50 | 0.42 | 0.53 | 0.55 | 0.56 | 0.48 | 0.43 | 0.60 |
| P46821 | Microtubule-associated protein 1B;MAP1B heavy chain;MAP1 light chain LC1 | MAP1B | 1387 | S | 0.95 | 253.7 | 0.96 | 0.72 | NaN | NaN | 1.77 | 1.10 | NaN | NaN | NaN |
| P46821 | Microtubule-associated protein 1B;MAP1B heavy chain;MAP1 light chain LC1 | MAP1B | 1389 | S | 1 | 265.4 | 0.78 | 0.87 | 0.93 | 0.94 | 0.98 | 1.72 | 0.67 | 0.62 | 0.68 |
| P46821 | Microtubule-associated protein 1B;MAP1B heavy chain;MAP1 light chain LC1 | MAP1B | 1252 | S | 0.9 | 114.8 | 0.28 | 0.45 | 0.46 | 0.13 | 0.64 | 0.31 | 0.89 | 0.69 | 0.53 |
| P46821 | Microtubule-associated protein 1B;MAP1B heavy chain;MAP1 light chain LC1 | MAP1B | 1254 | S | 0.677 | 114.8 | 0.44 | 0.47 | 0.77 | NaN | 1.08 | 0.66 | 0.97 | 0.69 | 0.83 |
| P46821 | Microtubule-associated protein 1B;MAP1B heavy chain;MAP1 light chain LC1 | MAP1B | 1256 | S | 0.963 | 114.8 | 0.28 | 1.05 | 1.09 | 1.11 | 1.08 | 0.95 | 0.96 | 1.02 | 0.87 |

| | | | | | | | | | | | | | | | | |
|---|---|---|---|---|---|---|---|---|---|---|---|---|---|---|---|---|
| P46821 | Microtubule-associated protein 1B;MAP1B heavy chain;MAP1 light chain LC1 | MAP1B | 1258 | S | 0.826 | 114.6 | 0.28 | 0.74 | 1.09 | 1.08 | NaN | 0.75 | 0.96 | NaN | 0.91 |
| P46821 | Microtubule-associated protein 1B;MAP1B heavy chain;MAP1 light chain LC1 | MAP1B | 1260 | S | 0.98 | 114.7 | 1.14 | NaN | 0.50 | 0.44 | 1.08 | 0.92 | 1.09 | 1.12 | 0.75 |
| P46821 | Microtubule-associated protein 1B;MAP1B heavy chain;MAP1 light chain LC1 | MAP1B | 1265 | S | 1 | 217.8 | 1.03 | 1.03 | 1.05 | 0.83 | 0.85 | 0.88 | 1.05 | 1.02 | 0.27 |
| P46821 | Microtubule-associated protein 1B;MAP1B heavy chain;MAP1 light chain LC1 | MAP1B | 1154 | S | 0.993 | 204.9 | 0.62 | 0.62 | 0.58 | 0.66 | 0.86 | 0.85 | 0.43 | 0.48 | 0.54 |
| P46821 | Microtubule-associated protein 1B;MAP1B heavy chain;MAP1 light chain LC1 | MAP1B | 1208 | S | 0.981 | 243.8 | 0.44 | 0.46 | NaN | 0.74 | 0.86 | 0.75 | NaN | 0.56 | 0.50 |
| P46821 | Microtubule-associated protein 1B;MAP1B heavy chain;MAP1 light chain LC1 | MAP1B | 614 | S | 1 | 110 | 0.38 | 0.37 | 0.41 | 0.63 | 0.55 | 0.66 | 0.76 | 0.80 | 0.77 |
| P46821 | Microtubule-associated protein 1B;MAP1B heavy chain;MAP1 light chain LC1 | MAP1B | 1792 | S | 0.872 | 246.1 | 0.60 | 0.59 | 0.70 | NaN | 0.52 | 0.54 | 1.02 | 1.02 | 0.94 |
| P46821 | Microtubule-associated protein 1B;MAP1B heavy chain;MAP1 light chain LC1 | MAP1B | 1793 | S | 0.989 | 239.2 | 0.60 | 0.58 | 0.64 | 0.50 | 0.50 | 0.49 | 0.93 | 1.01 | 1.02 |
| P46821 | Microtubule-associated protein 1B;MAP1B heavy chain;MAP1 light chain LC1 | MAP1B | 1797 | S | 1 | 246.1 | 1.24 | 1.23 | 1.57 | 1.34 | 1.26 | 1.43 | 1.11 | 0.84 | 1.05 |
| P46821 | Microtubule-associated protein 1B;MAP1B heavy chain;MAP1 light chain LC1 | MAP1B | 995 | S | 1 | 101.6 | 0.43 | 0.43 | NaN | NaN | NaN | NaN | 1.57 | NaN | NaN |
| P46821 | Microtubule-associated protein 1B;MAP1B heavy chain;MAP1 light chain LC1 | MAP1B | 1427 | S | 1 | 204.2 | 1.18 | 1.21 | 1.21 | 1.19 | 1.22 | 1.20 | 1.31 | 1.25 | 1.35 |
| P46821 | Microtubule-associated protein 1B;MAP1B heavy chain;MAP1 light chain LC1 | MAP1B | 891 | S | 0.995 | 90.34 | 1.04 | 0.61 | NaN | NaN | NaN | NaN | NaN | NaN | NaN |
| P46821 | Microtubule-associated protein 1B;MAP1B heavy chain;MAP1 light chain LC1 | MAP1B | 2209 | S | 1 | 108.6 | 0.77 | 0.80 | 0.85 | 0.95 | 0.90 | 0.90 | 0.78 | 0.79 | NaN |
| P46821 | Microtubule-associated protein 1B;MAP1B heavy chain;MAP1 light chain LC1 | MAP1B | 2211 | S | 1 | 108.6 | 0.77 | 0.80 | 0.85 | 0.95 | 0.90 | 0.90 | 0.78 | 0.79 | NaN |
| P46821 | Microtubule-associated protein 1B;MAP1B heavy chain;MAP1 light chain LC1 | MAP1B | 561 | S | 0.985 | 63.68 | 0.32 | NaN | NaN | 0.24 | NaN | NaN | 0.26 | 0.25 | 0.65 |

| | | | | | | | | | | | | | | | |
|---|---|---|---|---|---|---|---|---|---|---|---|---|---|---|---|
| P46821 | Microtubule-associated protein 1B;MAP1B heavy chain;MAP1 light chain LC1 | MAP1B | 1501 | S | 1 | 199.4 | 0.89 | 0.88 | 0.64 | 0.85 | 0.85 | 0.81 | 0.90 | 0.56 | 0.92 |
| P46821 | Microtubule-associated protein 1B;MAP1B heavy chain;MAP1 light chain LC1 | MAP1B | 1438 | S | 1 | 241.1 | 0.31 | 0.32 | 0.34 | 0.41 | 0.59 | 0.46 | 0.47 | 0.62 | 0.86 |
| P46821 | Microtubule-associated protein 1B;MAP1B heavy chain;MAP1 light chain LC1 | MAP1B | 1443 | S | 1 | 241.1 | 0.63 | 0.68 | 0.67 | 0.83 | 0.77 | 0.73 | 0.66 | 0.51 | 0.58 |
| P46821 | Microtubule-associated protein 1B;MAP1B heavy chain;MAP1 light chain LC1 | MAP1B | 937 | S | 0.784 | 221.1 | 0.71 | 0.55 | NaN | 0.52 | NaN | 0.66 | 0.89 | 0.82 | NaN |
| P46821 | Microtubule-associated protein 1B;MAP1B heavy chain;MAP1 light chain LC1 | MAP1B | 1666 | S | 0.999 | 179.2 | NaN | 0.27 | 0.54 | 0.69 | 0.62 | NaN | 0.83 | NaN | 0.74 |
| P46821 | Microtubule-associated protein 1B;MAP1B heavy chain;MAP1 light chain LC1 | MAP1B | 1782 | S | 1 | 120.4 | 1.42 | 1.65 | 1.17 | 0.81 | 1.14 | 1.15 | 1.55 | 1.49 | 1.69 |
| P46821 | Microtubule-associated protein 1B;MAP1B heavy chain;MAP1 light chain LC1 | MAP1B | 1785 | S | 1 | 146.8 | 0.37 | 0.34 | 0.36 | 0.36 | 0.36 | 0.38 | 0.41 | 0.37 | 0.36 |
| P46821 | Microtubule-associated protein 1B;MAP1B heavy chain;MAP1 light chain LC1 | MAP1B | 831 | S | 1 | 209.4 | 0.58 | 0.59 | 0.63 | 0.30 | 0.77 | 0.76 | 0.31 | 0.19 | 0.69 |
| P46821 | Microtubule-associated protein 1B;MAP1B heavy chain;MAP1 light chain LC1 | MAP1B | 832 | S | 1 | 209.4 | 0.21 | 0.28 | 0.63 | 0.32 | 0.32 | 0.76 | 0.24 | 0.66 | 0.69 |
| P46821 | Microtubule-associated protein 1B;MAP1B heavy chain;MAP1 light chain LC1 | MAP1B | 1915 | S | 0.999 | 205 | 0.82 | 0.67 | 0.70 | 0.91 | 0.90 | 0.80 | 0.97 | 0.93 | 0.91 |
| P46821 | Microtubule-associated protein 1B;MAP1B heavy chain;MAP1 light chain LC1 | MAP1B | 2256 | S | 0.989 | 84.62 | 0.76 | NaN | 1.06 | NaN | NaN | NaN | 0.83 | NaN | 0.65 |
| P46821 | Microtubule-associated protein 1B;MAP1B heavy chain;MAP1 light chain LC1 | MAP1B | 1322 | S | 1 | 215.4 | 0.27 | 0.30 | 0.33 | 0.47 | 0.44 | 0.48 | 0.61 | 0.65 | 0.57 |
| P46821 | Microtubule-associated protein 1B;MAP1B heavy chain;MAP1 light chain LC1 | MAP1B | 1326 | S | 0.613 | 106.3 | 0.37 | 0.42 | NaN | NaN | NaN | NaN | 1.28 | NaN | NaN |
| P46821 | Microtubule-associated protein 1B;MAP1B heavy chain;MAP1 light chain LC1 | MAP1B | 1339 | S | 0.938 | 224 | 0.51 | 0.30 | 0.49 | 0.47 | 0.44 | 0.59 | 0.47 | 1.78 | 0.52 |
| P46821 | Microtubule-associated protein 1B;MAP1B heavy chain;MAP1 light chain LC1 | MAP1B | 1881 | S | 0.989 | 237.6 | 0.44 | NaN | NaN | NaN | 0.64 | 0.62 | 0.58 | 0.55 | 0.53 |

| | | | | | | | | | | | | | | | |
|---|---|---|---|---|---|---|---|---|---|---|---|---|---|---|---|
| P46821 | Microtubule-associated protein 1B;MAP1B heavy chain;MAP1 light chain LC1 | MAP1B | 1396 | S | 1 | 375.4 | 1.07 | 0.81 | 1.03 | 0.99 | 0.96 | 0.91 | 0.80 | 1.10 | 0.89 |
| P46821 | Microtubule-associated protein 1B;MAP1B heavy chain;MAP1 light chain LC1 | MAP1B | 1400 | S | 1 | 375.4 | 0.52 | 0.63 | 1.19 | 0.62 | 0.57 | 0.58 | 1.00 | 1.11 | 1.16 |
| P46821 | Microtubule-associated protein 1B;MAP1B heavy chain;MAP1 light chain LC1 | MAP1B | 1779 | S | 1 | 154.5 | 0.44 | 0.46 | 0.43 | 0.45 | 0.46 | 0.44 | 0.64 | 0.64 | 0.62 |
| P46821 | Microtubule-associated protein 1B;MAP1B heavy chain;MAP1 light chain LC1 | MAP1B | 1298 | S | 1 | 183.6 | 1.29 | 1.33 | 1.36 | 0.73 | 1.02 | 1.26 | 1.04 | 0.89 | 0.98 |
| P46821 | Microtubule-associated protein 1B;MAP1B heavy chain;MAP1 light chain LC1 | MAP1B | 1312 | S | 1 | 205.6 | 0.67 | 0.65 | 0.55 | 0.70 | 0.62 | 0.69 | 0.78 | 0.93 | 1.57 |
| P46937 | Transcriptional coactivator YAP1 | YAP1 | 61 | S | 1 | 222.8 | 0.71 | 0.75 | 0.87 | 0.84 | 0.75 | 0.86 | 2.26 | 1.67 | 0.66 |
| P46937 | Transcriptional coactivator YAP1 | YAP1 | 289 | S | 1 | 116.5 | NaN | NaN | NaN | NaN | 2.01 | 2.28 | NaN | NaN | NaN |
| P46937 | Transcriptional coactivator YAP1 | YAP1 | 163 | S | 0.969 | 93.88 | NaN | 1.03 | NaN | NaN | 1.11 | NaN | NaN | 1.05 | 1.04 |
| P46937 | Transcriptional coactivator YAP1 | YAP1 | 164 | S | 0.966 | 114.1 | 0.99 | 0.97 | 1.07 | 0.97 | 0.96 | 1.10 | 1.08 | 0.88 | 1.17 |
| P46937 | Transcriptional coactivator YAP1 | YAP1 | 367 | S | 0.982 | 140.9 | 1.05 | NaN | NaN | 1.61 | 1.62 | NaN | NaN | NaN | 1.04 |
| P48634 | Protein PRRC2A | PRRC2A | 380 | S | 0.99 | 52.07 | 0.70 | 0.68 | NaN | NaN | 0.97 | NaN | NaN | NaN | NaN |
| P48634 | Protein PRRC2A | PRRC2A | 1219 | S | 1 | 86.29 | 2.23 | 2.23 | 2.13 | 2.15 | 2.24 | 2.09 | 1.31 | 1.28 | NaN |
| P48634 | Protein PRRC2A | PRRC2A | 342 | S | 1 | 106.1 | 1.77 | NaN | 1.16 | NaN | NaN | NaN | 1.04 | 0.98 | 1.11 |
| P48634 | Protein PRRC2A | PRRC2A | 350 | S | 1 | 106.1 | 1.77 | NaN | 1.16 | NaN | NaN | NaN | 1.04 | 0.98 | 1.11 |
| P48651 | Phosphatidylserine synthase 1 | PTDSS1 | 442 | S | 1 | 77.19 | 0.74 | 0.63 | NaN | 0.94 | 0.78 | 0.88 | 0.96 | NaN | 0.76 |
| P48681 | Nestin | NES | 680 | S | 1 | 85.75 | 1.18 | 1.05 | NaN | NaN | NaN | NaN | 1.03 | 0.93 | 1.04 |
| P48681 | Nestin | NES | 471 | S | 0.995 | 176.5 | 0.84 | 0.75 | 1.16 | 0.77 | NaN | 1.16 | 1.53 | NaN | NaN |
| P48681 | Nestin | NES | 768 | S | 1 | 50.87 | NaN | NaN | NaN | NaN | NaN | NaN | 1.35 | 1.27 | NaN |
| P48960 | CD97 antigen;CD97 antigen subunit alpha;CD97 antigen subunit beta | CD97 | 831 | S | 0.86 | 63.69 | NaN | NaN | 1.65 | 1.88 | NaN | 1.81 | 3.86 | 3.63 | NaN |
| P49321 | Nuclear autoantigenic sperm protein | NASP | 480 | S | 1 | 248.2 | NaN | 0.33 | 0.31 | 0.56 | 0.62 | 0.57 | 0.36 | 0.38 | NaN |
| P49321 | Nuclear autoantigenic sperm protein | NASP | 726 | S | 1 | 85.36 | 0.78 | 0.83 | 0.80 | 1.02 | NaN | 0.95 | 0.74 | NaN | 0.73 |
| P49321 | Nuclear autoantigenic sperm protein | NASP | 421 | S | 0.999 | 67.93 | 2.19 | 2.34 | 2.98 | 1.61 | 1.23 | 3.42 | 1.13 | 1.23 | 3.20 |
| P49321 | Nuclear autoantigenic sperm protein | NASP | 451 | S | 0.528 | 41.32 | NaN | NaN | NaN | 1.33 | 1.47 | NaN | NaN | 1.03 | 0.96 |
| P49454 | Centromere protein F | CENPF | 3079 | S | 0.832 | 70.99 | 0.54 | 0.63 | NaN | NaN | NaN | 1.51 | NaN | NaN | 0.52 |
| P49585 | Choline-phosphate cytidylyltransferase A | PCYT1A | 362 | S | 1 | 116.8 | 2.16 | 2.24 | 2.32 | 2.34 | 2.04 | 2.06 | 1.30 | 1.26 | 1.29 |

| | | | | | | | | | | | | | | | |
|---|---|---|---|---|---|---|---|---|---|---|---|---|---|---|---|
| P49585 | Choline-phosphate cytidylyltransferase A | PCYT1A | 329 | S | 1 | 71.15 | NaN | NaN | 0.82 | 0.93 | NaN | 0.87 | 0.90 | 0.80 | 0.87 |
| P49585 | Choline-phosphate cytidylyltransferase A | PCYT1A | 331 | S | 0.996 | 71.15 | NaN | NaN | 0.82 | 0.93 | 1.09 | 0.87 | 0.90 | 0.80 | 0.87 |
| P49585 | Choline-phosphate cytidylyltransferase A | PCYT1A | 315 | S | 1 | 120.8 | 0.72 | 0.59 | 0.70 | 0.89 | 1.00 | 0.75 | 0.57 | 0.54 | NaN |
| P49585 | Choline-phosphate cytidylyltransferase A | PCYT1A | 319 | S | 0.999 | 120.8 | 0.72 | 0.59 | 0.70 | 0.89 | 1.00 | 0.75 | 0.57 | 0.52 | NaN |
| P49585 | Choline-phosphate cytidylyltransferase A | PCYT1A | 321 | S | 0.569 | 63.46 | NaN | 0.47 | NaN | 0.93 | 1.04 | NaN | 0.55 | 0.54 | NaN |
| P49585 | Choline-phosphate cytidylyltransferase A | PCYT1A | 322 | S | 0.884 | 118 | 0.72 | 0.47 | NaN | 0.93 | 1.04 | NaN | 0.55 | 0.52 | NaN |
| P49585 | Choline-phosphate cytidylyltransferase A | PCYT1A | 323 | S | 0.872 | 120.8 | NaN | 0.53 | 0.70 | 0.88 | 1.00 | 0.75 | 0.57 | 0.52 | NaN |
| P49736 | DNA replication licensing factor MCM2 | MCM2 | 139 | S | 1 | 343.7 | 0.80 | 0.81 | 0.76 | 0.85 | 0.88 | 0.84 | 0.85 | 0.82 | 0.84 |
| P49736 | DNA replication licensing factor MCM2 | MCM2 | 26 | S | 0.588 | 229.8 | NaN | 0.86 | NaN | 0.96 | 1.00 | NaN | 0.80 | NaN | 0.69 |
| P49736 | DNA replication licensing factor MCM2 | MCM2 | 27 | S | 0.977 | 229.8 | 0.85 | NaN | 0.90 | 0.96 | 0.95 | 0.99 | NaN | NaN | NaN |
| P49736 | DNA replication licensing factor MCM2 | MCM2 | 41 | S | 0.957 | 150.4 | 0.70 | 0.69 | NaN | NaN | NaN | NaN | 0.66 | NaN | NaN |
| P49756 | RNA-binding protein 25 | RBM25 | 583 | S | 1 | 181.8 | 1.07 | 0.96 | 1.53 | 1.48 | 1.43 | 1.45 | 1.66 | 1.52 | 0.93 |
| P49790 | Nuclear pore complex protein Nup153 | NUP153 | 687 | S | 1 | 112.2 | NaN | NaN | 1.16 | 1.55 | 1.59 | 1.32 | 0.57 | NaN | 0.56 |
| P49792 | E3 SUMO-protein ligase RanBP2;RanBP2-like and GRIP domain-containing protein 4;RanBP2-like and GRIP domain-containing protein 3;RANBP2-like and GRIP domain-containing protein 1;RANBP2-like and GRIP domain-containing protein 2;RANBP2-like and GRIP domain-containing protein 5/6;RANBP2-like and | RANBP2 | 2510 | S | 1 | 61.18 | NaN | 0.46 | 0.54 | 0.84 | 0.88 | 1.10 | 0.84 | 0.82 | 1.19 |
| P49792 | E3 SUMO-protein ligase RanBP2;RanBP2-like and GRIP domain-containing protein 4;RanBP2-like and GRIP domain-containing protein 3;RANBP2-like and GRIP domain-containing protein 5/6;RANBP2-like and GRIP domain-containing | RANBP2 | 2246 | S | 0.989 | 152.2 | 0.98 | 1.32 | 1.62 | 1.27 | NaN | 1.25 | 1.31 | 1.27 | 1.04 |

| | | | | | | | | | | | | | | | |
|---|---|---|---|---|---|---|---|---|---|---|---|---|---|---|---|
| P49792 | E3 SUMO-protein ligase RanBP2;RanBP2-like and GRIP domain-containing protein 4;RanBP2-like and GRIP domain-containing protein 3;RANBP2-like and GRIP domain-containing protein 5/6;RANBP2-like and GRIP domain-containing | RANBP2 | 2250 | S | 0.902 | 217.6 | 0.98 | 2.06 | 1.09 | 1.27 | NaN | 1.25 | 1.31 | 1.27 | NaN |
| P49792 | E3 SUMO-protein ligase RanBP2;RanBP2-like and GRIP domain-containing protein 4;RanBP2-like and GRIP domain-containing protein 3;RANBP2-like and GRIP domain-containing protein 5/6;RANBP2-like and GRIP domain-containing | RANBP2 | 2251 | S | 0.835 | 160.5 | NaN | NaN | NaN | NaN | NaN | NaN | NaN | 1.27 | 1.04 |
| P49792 | E3 SUMO-protein ligase RanBP2 | RANBP2 | 1400 | S | 0.953 | 62.56 | 0.64 | 0.91 | 0.82 | 1.07 | 1.00 | NaN | 1.04 | 1.02 | NaN |
| P49792 | E3 SUMO-protein ligase RanBP2 | RANBP2 | 781 | S | 0.697 | 45.16 | NaN | NaN | 0.87 | 0.83 | 0.79 | NaN | NaN | 0.78 | NaN |
| P49792 | E3 SUMO-protein ligase RanBP2;RanBP2-like and GRIP domain-containing | RANBP2 | 2276 | S | 0.988 | 56.08 | 0.75 | 0.86 | 0.78 | NaN | 1.41 | 1.64 | 0.76 | 0.81 | NaN |
| P49792 | E3 SUMO-protein ligase RanBP2;RanBP2-like and GRIP domain-containing | RANBP2 | 2280 | S | 0.934 | 82.77 | 0.75 | 0.84 | 0.78 | NaN | 1.00 | 1.64 | NaN | 0.87 | NaN |
| P49792 | E3 SUMO-protein ligase RanBP2 | RANBP2 | 2900 | S | 1 | 164.5 | 0.91 | 0.79 | 0.94 | NaN | NaN | 0.86 | 0.90 | 0.96 | 1.21 |
| P49915 | GMP synthase [glutamine-hydrolyzing] | GMPS | 332 | S | 0.722 | 87.73 | NaN | 1.00 | 0.60 | NaN | NaN | NaN | NaN | NaN | NaN |
| P50914 | 60S ribosomal protein L14 | RPL14 | 139 | S | 1 | 80.56 | 1.47 | 1.57 | NaN | 2.00 | 2.04 | 1.99 | 0.54 | 0.56 | 0.54 |
| P51116 | Fragile X mental retardation syndrome-related protein 2 | FXR2 | 601 | S | 0.998 | 142.3 | 1.61 | 1.69 | 0.87 | 1.15 | 1.29 | NaN | 1.03 | 1.05 | 1.91 |
| P51116 | Fragile X mental retardation syndrome-related protein 2 | FXR2 | 603 | S | 1 | 142.3 | 1.59 | 1.59 | 1.65 | 1.05 | 1.29 | NaN | 1.75 | 1.81 | 1.91 |
| P51608 | Methyl-CpG-binding protein 2 | MECP2 | 80 | S | 0.765 | 64.78 | 0.42 | 0.40 | NaN | NaN | NaN | NaN | NaN | NaN | NaN |

| | | | | | | | | | | | | | | | |
|---|---|---|---|---|---|---|---|---|---|---|---|---|---|---|---|
| P51610 | Host cell factor 1;HCF N-terminal chain 1;HCF N-terminal chain 2;HCF N-terminal chain 3;HCF N-terminal chain 4;HCF N-terminal chain 5;HCF N-terminal chain 6;HCF C-terminal chain 1;HCF C-terminal chain 2;HCF C-terminal chain 3;HCF C- | HCFC1 | 1507 | S | 1 | 89.68 | 1.73 | 2.03 | 2.15 | NaN | NaN | 2.07 | 1.60 | 1.35 | NaN |
| P51812 | Ribosomal protein S6 kinase alpha-3 | RPS6KA3 | 715 | S | 1 | 57.43 | NaN | NaN | NaN | 1.78 | NaN | NaN | 1.24 | 1.08 | 1.18 |
| P51812 | Ribosomal protein S6 kinase alpha-3 | RPS6KA3 | 369 | S | 0.987 | 51 | NaN | NaN | NaN | NaN | NaN | 1.24 | 0.80 | 0.83 | 0.87 |
| P51858 | Hepatoma-derived growth factor | HDGF | 165 | S | 1 | 191.9 | 0.98 | 0.94 | 0.98 | 0.99 | 0.99 | 1.00 | 1.09 | 1.10 | 1.11 |
| P51858 | Hepatoma-derived growth factor | HDGF | 132 | S | 1 | 219 | 1.03 | 1.00 | 0.66 | 0.94 | 0.92 | 0.75 | 1.16 | 1.14 | 0.98 |
| P51858 | Hepatoma-derived growth factor | HDGF | 133 | S | 1 | 219 | 0.84 | 0.66 | 0.72 | 0.62 | 0.73 | 0.80 | 0.73 | 0.65 | 0.83 |
| P51991 | Heterogeneous nuclear ribonucleoprotein A3 | HNRNPA3 | 358 | S | 1 | 277.5 | 1.01 | 1.03 | 0.94 | 0.91 | 0.99 | 0.96 | 1.19 | 1.10 | 0.97 |
| P52756 | RNA-binding protein 5 | RBM5 | 59 | S | 0.852 | 40.62 | 1.02 | 0.86 | NaN | NaN | NaN | NaN | NaN | NaN | NaN |
| P52756 | RNA-binding protein 5 | RBM5 | 624 | S | 1 | 176.9 | 1.33 | 1.12 | NaN | NaN | 1.11 | 0.90 | 1.31 | 1.42 | NaN |
| P52926 | High mobility group protein HMGI-C | HMGA2 | 44 | S | 1 | 108.4 | 0.37 | NaN | 0.61 | 1.11 | 1.03 | 0.48 | NaN | 0.78 | 0.79 |
| P53396 | ATP-citrate synthase | ACLY | 481 | S | 1 | 92.77 | 1.65 | 1.59 | 1.68 | 2.08 | 2.24 | 2.07 | 0.99 | 1.02 | 0.91 |
| P54198 | Protein HIRA | HIRA | 610 | S | 1 | 57.41 | 0.66 | NaN | NaN | NaN | 0.60 | NaN | NaN | 0.96 | 0.86 |
| P54198 | Protein HIRA | HIRA | 611 | S | 1 | 57.41 | 0.66 | NaN | NaN | NaN | 0.60 | NaN | NaN | 0.96 | 0.86 |
| P54198 | Protein HIRA | HIRA | 612 | S | 1 | 57.41 | 0.66 | NaN | NaN | NaN | 0.60 | NaN | NaN | 0.96 | 0.86 |
| P54198 | Protein HIRA | HIRA | 614 | S | 1 | 57.41 | 0.66 | NaN | NaN | NaN | 0.60 | NaN | NaN | 0.96 | 0.86 |
| P54259 | Atrophin-1 | ATN1 | 661 | S | 0.926 | 77.63 | NaN | 1.05 | 1.07 | 1.02 | NaN | 0.96 | 0.95 | 0.81 | 0.94 |
| P54259 | Atrophin-1 | ATN1 | 101 | S | 0.958 | 62.65 | NaN | 0.58 | 0.80 | 0.60 | 0.57 | NaN | NaN | NaN | NaN |
| P55081 | Microfibrillar-associated protein 1 | MFAP1 | 116 | S | 1 | 155.9 | 1.10 | 1.00 | 1.11 | 1.08 | 1.04 | 1.11 | 1.05 | 0.96 | 1.16 |
| P55081 | Microfibrillar-associated protein 1 | MFAP1 | 118 | S | 1 | 155.9 | 1.27 | 1.29 | 1.27 | 1.15 | 1.02 | 1.05 | 1.29 | 0.99 | 1.24 |
| P55081 | Microfibrillar-associated protein 1 | MFAP1 | 52 | S | 1 | 134.1 | 0.62 | 0.66 | 0.66 | 0.84 | 0.80 | 0.72 | 0.52 | 0.50 | 0.59 |
| P55081 | Microfibrillar-associated protein 1 | MFAP1 | 53 | S | 1 | 134.1 | 0.62 | 0.66 | 0.66 | 0.84 | 0.80 | 0.72 | 0.52 | 0.50 | 0.59 |
| P55327 | Tumor protein D52 | TPD52 | 171 | S | 0.955 | 66.39 | 1.64 | NaN | NaN | 2.65 | 2.90 | 3.05 | 0.78 | 0.87 | 1.20 |
| P55327 | Tumor protein D52 | TPD52 | 176 | S | 1 | 76.07 | 0.66 | 0.59 | 0.83 | 0.49 | 0.56 | 0.63 | 3.08 | 2.88 | 3.51 |
| P56524 | Histone deacetylase 4 | HDAC4 | 565 | S | 1 | 140 | 0.90 | 1.02 | 0.89 | 0.73 | 0.66 | 0.93 | 0.98 | 1.17 | 1.06 |
| P60174 | Triosephosphate isomerase | TPI1 | 58 | S | 1 | 257.5 | 1.00 | 1.09 | 1.00 | 1.18 | 1.16 | 1.11 | 1.06 | 0.96 | 1.13 |
| P61006 | Ras-related protein Rab-8A | RAB8A | 181 | S | 1 | 158.7 | NaN | NaN | 8.22 | 20.18 | NaN | 11.42 | 1.97 | NaN | NaN |

| | | | | | | | | | | | | | | | |
|---|---|---|---|---|---|---|---|---|---|---|---|---|---|---|---|
| P61978 | Heterogeneous nuclear ribonucleoprotein K | HNRNPK | 284 | S | 1 | 113.6 | 1.22 | 1.32 | 1.26 | 1.24 | NaN | 1.18 | 1.19 | 1.18 | 1.31 |
| P61978 | Heterogeneous nuclear ribonucleoprotein K | HNRNPK | 379 | S | 0.952 | 92.43 | 1.93 | NaN | NaN | NaN | 2.54 | 2.87 | 1.62 | NaN | NaN |
| P61978 | Heterogeneous nuclear ribonucleoprotein K | HNRNPK | 214 | S | 0.979 | 106.3 | 1.98 | 2.14 | 2.66 | NaN | NaN | NaN | 0.90 | 0.86 | 0.85 |
| P61978 | Heterogeneous nuclear ribonucleoprotein K | HNRNPK | 216 | S | 0.985 | 139 | 1.90 | 2.20 | 2.32 | 3.68 | 3.59 | NaN | NaN | NaN | 0.95 |
| P61978 | Heterogeneous nuclear ribonucleoprotein K | HNRNPK | 116 | S | 1 | 252.8 | 1.05 | 1.02 | 0.95 | 0.98 | 1.09 | 1.02 | 0.49 | 0.48 | 0.45 |
| P62070 | Ras-related protein R-Ras2 | RRAS2 | 186 | S | 1 | 181.7 | 1.39 | 1.42 | 1.34 | 1.48 | 1.49 | 1.49 | 1.01 | 0.94 | 1.03 |
| P62258 | 14-3-3 protein epsilon | YWHAE | 210 | S | 0.989 | 125.5 | 1.01 | 1.06 | 1.18 | 0.99 | 1.01 | 1.02 | 1.03 | 0.98 | 0.99 |
| P62328 | Thymosin beta-4;Hematopoietic system | TMSB4X | 44 | S | 1 | 145.5 | 1.03 | 1.27 | 1.40 | 1.62 | 1.66 | 1.80 | 1.02 | 1.01 | NaN |
| P62995 | Transformer-2 protein homolog beta | TRA2B | 95 | S | 0.999 | 53.74 | 0.77 | 0.80 | NaN | NaN | NaN | NaN | NaN | NaN | NaN |
| P62995 | Transformer-2 protein homolog beta | TRA2B | 97 | S | 0.994 | 53.74 | 0.77 | 0.80 | NaN | NaN | NaN | NaN | NaN | NaN | NaN |
| P62995 | Transformer-2 protein homolog beta | TRA2B | 99 | S | 0.757 | 53.74 | 0.77 | 0.80 | NaN | NaN | NaN | NaN | NaN | NaN | NaN |
| Q13595 | Transformer-2 protein homolog beta;Transformer-2 protein homolog alpha | TRA2B | 264;260 | S | 1 | 105.8 | 0.63 | 0.66 | 0.56 | 0.64 | 0.60 | 0.90 | 1.14 | 1.14 | 0.61 |
| Q13595 | Transformer-2 protein homolog beta;Transformer-2 protein homolog alpha | TRA2B | 266;262 | S | 0.999 | 103.1 | 0.92 | 0.75 | 1.00 | 0.91 | 0.95 | 0.90 | 1.14 | 1.10 | 1.19 |
| Q13595 | Transformer-2 protein homolog beta;Transformer-2 protein homolog alpha | TRA2B | 270;266 | S | 0.894 | 105.8 | 0.93 | NaN | NaN | NaN | NaN | NaN | 1.09 | NaN | 1.14 |
| Q5VU10 | Ribonuclease P protein subunit p30 | RPP30 | 195 | S | 1 | 59.56 | 0.83 | 0.78 | NaN | 1.17 | 1.06 | NaN | NaN | NaN | NaN |
| P78527 | DNA-dependent protein kinase catalytic subunit | PRKDC | 2612 | S | 0.992 | 159.8 | 0.91 | 0.98 | 0.93 | 1.27 | 1.22 | 1.13 | 0.65 | 0.73 | NaN |
| P78536 | Disintegrin and metalloproteinase domain-containing protein 17 | ADAM17 | 791 | S | 1 | 137 | 2.04 | 2.33 | 2.60 | 2.44 | 2.30 | 1.96 | 1.66 | 1.77 | 1.86 |
| P80723 | Brain acid soluble protein 1 | BASP1 | 164 | S | 0.99 | 60.62 | NaN | 0.91 | NaN | 0.80 | 0.85 | NaN | NaN | 1.38 | NaN |
| P80723 | Brain acid soluble protein 1 | BASP1 | 170 | S | 0.888 | 51.62 | 1.04 | NaN | 1.11 | NaN | NaN | 0.92 | NaN | NaN | NaN |
| P84103 | Serine/arginine-rich splicing factor 3 | SRSF3 | 138 | S | 1 | 67.19 | 1.31 | 1.36 | 1.27 | 1.17 | 1.16 | 1.10 | 1.51 | 1.48 | 1.43 |
| P84103 | Serine/arginine-rich splicing factor 3 | SRSF3 | 140 | S | 1 | 67.19 | 1.31 | 1.36 | 1.27 | 1.17 | 1.16 | 1.10 | 1.51 | 1.48 | 1.43 |
| P85037 | Forkhead box protein K1 | FOXK1 | 299 | S | 0.695 | 197 | NaN | NaN | NaN | NaN | NaN | NaN | 0.78 | 0.61 | NaN |
| P85037 | Forkhead box protein K1 | FOXK1 | 213 | S | 0.999 | 75.18 | NaN | 0.93 | NaN | 0.74 | NaN | 0.88 | 0.73 | 0.88 | NaN |
| P85037 | Forkhead box protein K1 | FOXK1 | 445 | S | 1 | 158.9 | 0.96 | 0.99 | 1.00 | 0.92 | 0.91 | 0.89 | 0.97 | 0.98 | 0.95 |
| P85037 | Forkhead box protein K1 | FOXK1 | 420 | S | 0.987 | 142.3 | 0.79 | 0.74 | 0.74 | 0.76 | 0.81 | 0.72 | 0.68 | 0.63 | 0.73 |
| P85037 | Forkhead box protein K1 | FOXK1 | 243 | S | 0.976 | 103.9 | 0.83 | 0.77 | 0.67 | 1.33 | 0.97 | 0.92 | NaN | 1.19 | NaN |

| | | | | | | | | | | | | | | |
|---|---|---|---|---|---|---|---|---|---|---|---|---|---|---|
| P85037 | Forkhead box protein K1 | FOXK1 | 253 | S | 0.684 | 62.61 | NaN | NaN | NaN | NaN | 0.97 | 0.79 | NaN | 1.19 | NaN |
| P85037 | Forkhead box protein K1 | FOXK1 | 257 | S | 0.972 | 90.32 | 0.83 | 0.77 | 0.67 | NaN | NaN | NaN | 0.97 | 0.67 | NaN |
| P98082 | Disabled homolog 2 | DAB2 | 401 | S | 1 | 62.69 | 0.87 | 1.00 | NaN | 0.86 | 1.15 | NaN | NaN | NaN | NaN |
| P98175 | RNA-binding protein 10 | RBM10 | 736 | S | 1 | 120.9 | 0.99 | 0.95 | 1.04 | 0.88 | 0.94 | 0.86 | 1.09 | 1.17 | 1.04 |
| P98175 | RNA-binding protein 10 | RBM10 | 738 | S | 1 | 136.6 | 0.99 | 0.95 | 1.04 | 0.88 | 0.94 | 0.86 | 1.09 | 1.17 | 1.04 |
| P98175 | RNA-binding protein 10 | RBM10 | 723 | S | 1 | 121.5 | 1.21 | 1.30 | 1.22 | 1.14 | 1.13 | 1.11 | 1.15 | 1.15 | 1.18 |
| Q00587 | Cdc42 effector protein 1 | CDC42EP 1 | 350 | S | 1 | 92.3 | NaN | 0.93 | 0.96 | NaN | 0.78 | 0.84 | 1.36 | 1.28 | NaN |
| Q00587 | Cdc42 effector protein 1 | CDC42EP 1 | 101 | S | 0.985 | 41.67 | NaN | 1.21 | NaN | 1.51 | 1.64 | NaN | NaN | 0.61 | NaN |
| Q00587 | Cdc42 effector protein 1 | CDC42EP 1 | 303 | S | 0.998 | 81.19 | 1.32 | 1.25 | 1.26 | NaN | NaN | 0.94 | NaN | 1.73 | 1.83 |
| Q01130 | Serine/arginine-rich splicing factor 2 | SRSF2 | 206 | S | 1 | 60.74 | NaN | 0.54 | 1.08 | 0.76 | 0.57 | NaN | 1.14 | 0.97 | NaN |
| Q01130 | Serine/arginine-rich splicing factor 2 | SRSF2 | 208 | S | 1 | 60.74 | NaN | 0.54 | 1.08 | 0.76 | 0.57 | NaN | 1.14 | 0.97 | NaN |
| Q01130 | Serine/arginine-rich splicing factor 2 | SRSF2 | 212 | S | 1 | 58.69 | NaN | 0.54 | 1.08 | 1.06 | NaN | NaN | 1.14 | 1.27 | NaN |
| Q01130 | Serine/arginine-rich splicing factor 2 | SRSF2 | 220 | S | 0.545 | 60.74 | NaN | NaN | 1.08 | 0.76 | 0.57 | NaN | 1.14 | 0.97 | NaN |
| Q01130 | Serine/arginine-rich splicing factor 2 | SRSF2 | 189 | S | 1 | 73.44 | 1.07 | 1.06 | 1.01 | 0.95 | 0.97 | 0.97 | 1.15 | 1.12 | 1.16 |
| Q01130 | Serine/arginine-rich splicing factor 2 | SRSF2 | 191 | S | 1 | 73.44 | 1.07 | 1.06 | 1.01 | 0.30 | 0.97 | 0.97 | 0.29 | 0.28 | 1.16 |
| Q01130 | Serine/arginine-rich splicing factor 2 | SRSF2 | 187 | S | 1 | 60.55 | 0.97 | 0.98 | NaN | NaN | 0.88 | 0.90 | 1.07 | 1.13 | 1.04 |
| Q01130 | Serine/arginine-rich splicing factor 2 | SRSF2 | 26 | S | 0.999 | 157 | 1.00 | 0.97 | 0.98 | 1.22 | 1.15 | 1.21 | 1.05 | 0.85 | 1.13 |
| Q01167 | Forkhead box protein K2 | FOXK2 | 398 | S | 1 | 182.1 | 0.59 | 0.42 | NaN | NaN | NaN | NaN | 0.87 | 0.73 | NaN |
| Q01167 | Forkhead box protein K2 | FOXK2 | 170 | S | 0.732 | 47.31 | NaN | NaN | 0.57 | NaN | 1.01 | 0.98 | 0.68 | 0.68 | NaN |
| Q01518 | Adenylyl cyclase-associated protein 1;Adenylyl cyclase-associated protein | CAP1 | 34 | S | 0.999 | 157.8 | 3.02 | 2.54 | 2.96 | 4.15 | 4.26 | 4.46 | 0.96 | 0.87 | 1.00 |
| Q01518 | Adenylyl cyclase-associated protein 1 | CAP1 | 308 | S | 0.882 | 78.35 | NaN | NaN | 0.35 | 0.36 | 0.36 | 0.32 | 0.20 | 0.24 | 0.21 |
| Q01804 | OTU domain-containing protein 4 | OTUD4 | 1006 | S | 0.999 | 88.49 | 1.65 | 1.61 | NaN | NaN | NaN | NaN | 0.82 | NaN | NaN |
| Q01804 | OTU domain-containing protein 4 | OTUD4 | 1023 | S | 0.999 | 117.2 | 1.08 | 1.06 | NaN | 0.75 | NaN | NaN | NaN | 1.01 | NaN |
| Q01804 | OTU domain-containing protein 4 | OTUD4 | 1024 | S | 0.999 | 117.2 | 1.08 | 1.06 | NaN | 0.75 | NaN | NaN | NaN | 1.01 | NaN |
| Q02040 | A-kinase anchor protein 17A | AKAP17A | 537 | S | 1 | 77.59 | NaN | 1.81 | 0.80 | 0.85 | 0.88 | 0.96 | 0.93 | 0.92 | 0.95 |
| Q02880 | DNA topoisomerase 2-beta | TOP2B | 1336 | S | 0.998 | 52.35 | 0.63 | 0.81 | 0.78 | 0.67 | NaN | NaN | NaN | 1.50 | 0.89 |
| Q02880 | DNA topoisomerase 2-beta | TOP2B | 1340 | S | 0.999 | 52.35 | 0.63 | 0.81 | 0.78 | 0.67 | NaN | NaN | NaN | 1.50 | 0.89 |
| Q02880 | DNA topoisomerase 2-beta | TOP2B | 1342 | S | 0.996 | 52.35 | 0.63 | 0.81 | 0.78 | 0.67 | NaN | NaN | NaN | 1.50 | 0.89 |
| Q02880 | DNA topoisomerase 2-beta | TOP2B | 1344 | S | 0.995 | 52.35 | 0.63 | 0.81 | 0.78 | 0.67 | NaN | NaN | NaN | 1.50 | 0.89 |
| Q02880 | DNA topoisomerase 2-beta | TOP2B | 1581 | S | 0.995 | 110.3 | 0.77 | 0.78 | 0.76 | 0.80 | 0.80 | 0.71 | 1.00 | 1.04 | 1.03 |

| | | | | | | | | | | | | | | | |
|---|---|---|---|---|---|---|---|---|---|---|---|---|---|---|---|
| Q02880 | DNA topoisomerase 2-beta | TOP2B | 1522 | S | 1 | 173 | 0.72 | 0.37 | 0.81 | 0.93 | 0.69 | 0.88 | 0.99 | 1.05 | 0.87 |
| Q02880 | DNA topoisomerase 2-beta | TOP2B | 1524 | S | 0.998 | 173 | 0.72 | 0.69 | 0.81 | 0.93 | 0.58 | 0.88 | 0.99 | 1.05 | 0.87 |
| Q02880 | DNA topoisomerase 2-beta | TOP2B | 1526 | S | 0.958 | 89.14 | NaN | NaN | NaN | NaN | 0.84 | 0.94 | NaN | 0.88 | NaN |
| Q02880 | DNA topoisomerase 2-beta | TOP2B | 1400 | S | 0.952 | 93.86 | NaN | 0.57 | NaN | NaN | 0.88 | 0.67 | NaN | 0.37 | NaN |
| Q02880 | DNA topoisomerase 2-beta | TOP2B | 1413 | S | 0.995 | 93.86 | NaN | 0.50 | NaN | NaN | 0.88 | 0.67 | NaN | 0.37 | NaN |
| Q02952 | A-kinase anchor protein 12 | AKAP12 | 1331 | S | 0.999 | 55.31 | 1.66 | 1.87 | NaN | NaN | 0.79 | 0.92 | 2.46 | NaN | NaN |
| Q03111 | Protein ENL | MLLT1 | 475 | S | 0.993 | 47.54 | NaN | NaN | NaN | 1.03 | 1.16 | 0.68 | NaN | NaN | 0.74 |
| Q03111 | Protein ENL | MLLT1 | 315 | S | 0.5 | 84.82 | NaN | NaN | NaN | 0.93 | NaN | NaN | 0.97 | NaN | 0.85 |
| Q03164 | Histone-lysine N-methyltransferase 2A;MLL cleavage product N320;MLL cleavage product C180 | KMT2A | 1837 | S | 0.973 | 93.15 | 0.59 | 0.58 | 0.80 | 0.64 | NaN | NaN | NaN | NaN | 0.74 |
| Q03164 | Histone-lysine N-methyltransferase 2A;MLL cleavage product N320;MLL cleavage product C180 | KMT2A | 3527 | S | 0.949 | 50.4 | 0.69 | 0.83 | NaN | NaN | NaN | NaN | NaN | NaN | NaN |
| Q03164 | Histone-lysine N-methyltransferase 2A;MLL cleavage product N320;MLL cleavage product C180 | KMT2A | 2098 | S | 0.98 | 83.72 | NaN | 0.54 | 0.51 | NaN | 0.72 | NaN | 0.51 | NaN | NaN |
| Q03252 | Lamin-B2 | LMNB2 | 37 | S | 0.999 | 165 | 3.48 | 3.56 | 3.37 | NaN | NaN | 5.36 | 0.88 | NaN | 0.98 |
| Q04721 | Neurogenic locus notch homolog protein 2;Notch 2 extracellular truncation;Notch 2 intracellular domain | NOTCH2 | 1845 | S | 0.992 | 206.4 | 1.01 | 1.40 | NaN | NaN | NaN | 1.14 | 1.05 | NaN | 0.87 |
| Q05209 | Tyrosine-protein phosphatase non-receptor type 12 | PTPN12 | 603 | S | 1 | 121.6 | 1.13 | 1.07 | 1.25 | 0.81 | 0.90 | 0.86 | 1.09 | 0.89 | 1.10 |
| Q05209 | Tyrosine-protein phosphatase non-receptor type 12 | PTPN12 | 606 | S | 0.999 | 121.6 | 1.13 | 1.07 | 1.11 | 0.49 | 0.90 | 0.86 | 0.76 | 1.31 | 1.00 |
| Q5T757 | Serine/arginine-rich splicing factor 11 | SRSF11 | 374 | S | 1 | 237.9 | 1.09 | 1.15 | 1.08 | 1.05 | 1.15 | 8.01 | 1.56 | 1.31 | 1.36 |
| Q5T757 | Serine/arginine-rich splicing factor 11 | SRSF11 | 389 | S | 1 | 115.9 | NaN | 0.90 | 0.87 | 0.98 | 1.01 | 0.96 | 0.92 | NaN | 0.90 |
| Q5T757 | Serine/arginine-rich splicing factor 11 | SRSF11 | 147 | S | 0.994 | 113.5 | 2.82 | 2.73 | 3.13 | 3.72 | NaN | 3.27 | 1.18 | 1.24 | 1.65 |
| Q96PN7 | Transcriptional-regulating factor 1 | BCAR2 | 491;491 | S | 0.991 | 78.4 | 0.43 | 0.44 | 0.35 | 0.62 | 0.61 | 0.54 | 0.67 | 0.60 | 0.62 |
| Q06210 | Glutamine--fructose-6-phosphate aminotransferase [isomerizing] 1 | GFPT1 | 261 | S | 0.905 | 100.2 | 22.02 | 20.07 | NaN | NaN | NaN | NaN | NaN | NaN | NaN |
| Q07065 | Cytoskeleton-associated protein 4 | CKAP4 | 17 | S | 0.987 | 129.8 | 2.84 | NaN | NaN | 4.15 | 5.10 | NaN | NaN | NaN | NaN |
| Q08211 | ATP-dependent RNA helicase | DHX9 | 87 | S | 1 | 247.7 | 0.95 | 0.97 | 0.98 | 1.42 | 1.16 | 1.34 | 0.74 | 0.83 | 0.87 |
| Q08AD1 | Calmodulin-regulated spectrin-associated protein 2 | CAMSAP 2 | 599 | S | 0.733 | 56.47 | NaN | NaN | NaN | 0.75 | 0.65 | 1.04 | 0.86 | 0.88 | NaN |
| Q08AD1 | Calmodulin-regulated spectrin-associated protein 2 | CAMSAP 2 | 464 | S | 0.999 | 103.6 | NaN | 2.38 | 2.50 | 1.91 | NaN | NaN | 1.80 | 2.10 | 2.17 |

| | | | | | | | | | | | | | | | |
|---|---|---|---|---|---|---|---|---|---|---|---|---|---|---|---|
| Q08J23 | tRNA (cytosine(34)-C(5))-methyltransferase | NSUN2 | 456 | S | 0.982 | 61.49 | 0.39 | NaN | 0.58 | 0.52 | NaN | NaN | 0.94 | 1.21 | 0.97 |
| Q09472 | Histone acetyltransferase | EP300 | 1038 | S | 0.953 | 146.8 | NaN | NaN | NaN | 0.98 | NaN | 0.93 | NaN | 0.94 | NaN |
| Q09666 | Neuroblast differentiation-associated protein AHNAK | AHNAK | 5110 | S | 1 | 128.9 | 1.84 | 2.00 | 1.92 | 2.17 | 2.14 | 2.18 | 1.34 | 1.36 | 1.41 |
| Q09666 | Neuroblast differentiation-associated protein AHNAK | AHNAK | 5749 | S | 1 | 181.3 | 0.79 | 0.65 | 0.59 | 0.70 | 0.84 | 0.66 | 0.88 | 0.83 | 0.81 |
| Q09666 | Neuroblast differentiation-associated protein AHNAK | AHNAK | 5752 | S | 1 | 318 | 0.60 | 0.62 | 0.66 | 0.49 | 0.55 | 0.53 | 0.86 | 0.83 | 0.87 |
| Q09666 | Neuroblast differentiation-associated protein AHNAK | AHNAK | 5762 | S | 0.889 | 283.8 | 1.19 | 1.24 | 0.90 | 0.95 | NaN | 0.66 | 1.03 | NaN | 0.94 |
| Q09666 | Neuroblast differentiation-associated protein AHNAK | AHNAK | 5763 | S | 0.99 | 387.5 | 0.87 | 0.87 | 1.17 | 1.48 | 1.40 | 1.34 | 1.01 | 1.00 | 0.99 |
| Q09666 | Neuroblast differentiation-associated protein AHNAK | AHNAK | 41 | S | 1 | 202.8 | 2.71 | NaN | 2.93 | 4.37 | 3.90 | 4.23 | 1.09 | 0.94 | NaN |
| Q09666 | Neuroblast differentiation-associated protein AHNAK | AHNAK | 177 | S | 0.996 | 196.3 | 1.07 | 1.12 | NaN | 1.27 | 1.28 | 1.22 | 0.99 | 1.00 | 1.03 |
| Q09666 | Neuroblast differentiation-associated protein AHNAK | AHNAK | 4986 | S | 1 | 58.74 | 1.73 | 1.76 | 1.77 | 1.96 | 1.83 | 1.70 | 1.61 | 1.68 | 1.40 |
| Q09666 | Neuroblast differentiation-associated protein AHNAK | AHNAK | 5731 | S | 1 | 207.9 | 0.79 | 0.86 | 0.81 | 1.01 | 0.99 | 1.01 | 0.91 | 0.89 | 0.89 |
| Q09666 | Neuroblast differentiation-associated protein AHNAK | AHNAK | 5841 | S | 1 | 239.5 | 0.86 | 0.85 | 0.83 | 0.82 | 0.85 | 0.87 | 1.10 | 1.04 | 0.89 |
| Q09666 | Neuroblast differentiation-associated protein AHNAK | AHNAK | 5857 | S | 0.725 | 41.76 | NaN | NaN | NaN | NaN | 0.82 | 1.23 | NaN | NaN | NaN |
| Q09666 | Neuroblast differentiation-associated protein AHNAK | AHNAK | 5552 | S | 1 | 237.3 | 2.19 | 1.96 | 1.93 | 4.22 | 5.55 | 5.11 | 0.43 | NaN | 0.49 |
| Q09666 | Neuroblast differentiation-associated protein AHNAK | AHNAK | 5448 | S | 1 | 105.6 | 0.87 | 0.90 | 0.58 | 0.64 | 0.74 | 0.65 | 1.64 | 1.87 | 1.83 |
| Q09666 | Neuroblast differentiation-associated protein AHNAK | AHNAK | 2397 | S | 1 | 168.3 | 2.06 | 2.25 | 2.16 | 3.25 | 3.34 | 3.05 | 0.84 | 0.93 | 0.95 |
| Q09666 | Neuroblast differentiation-associated protein AHNAK | AHNAK | 511 | S | 1 | 178.1 | 2.40 | 2.40 | 2.35 | 3.63 | 4.11 | 3.34 | 1.23 | 1.28 | 1.08 |
| Q09666 | Neuroblast differentiation-associated protein AHNAK | AHNAK | 93 | S | 1 | 118.7 | 0.31 | 0.34 | NaN | NaN | 0.35 | 0.37 | NaN | NaN | 0.23 |
| Q09666 | Neuroblast differentiation-associated protein AHNAK | AHNAK | 559 | S | 1 | 106.6 | 0.84 | 0.44 | 0.58 | 1.03 | 0.87 | 1.01 | 0.77 | NaN | 0.76 |
| Q09666 | Neuroblast differentiation-associated protein AHNAK | AHNAK | 135 | S | 1 | 260 | 0.80 | 0.80 | 0.84 | 1.29 | 1.30 | 1.32 | 1.36 | 1.34 | 1.43 |
| Q09666 | Neuroblast differentiation-associated protein AHNAK | AHNAK | 210 | S | 0.946 | 119.6 | 1.99 | 1.92 | 1.24 | 1.41 | 2.13 | NaN | 1.28 | NaN | NaN |
| Q09666 | Neuroblast differentiation-associated protein AHNAK | AHNAK | 212 | S | 0.758 | 111.1 | 4.51 | 3.33 | NaN | 2.66 | NaN | 1.77 | NaN | 0.99 | 1.87 |
| Q09666 | Neuroblast differentiation-associated protein AHNAK | AHNAK | 216 | S | 0.999 | 293.1 | 0.81 | 1.05 | 0.79 | 0.95 | 0.92 | 0.96 | 0.95 | 0.96 | 0.99 |
| Q09666 | Neuroblast differentiation-associated protein AHNAK | AHNAK | 5099 | S | 1 | 104.8 | 5.17 | 4.84 | NaN | NaN | NaN | NaN | NaN | NaN | NaN |

| | | | | | | | | | | | | | | | |
|---|---|---|---|---|---|---|---|---|---|---|---|---|---|---|---|
| Q09666 | Neuroblast differentiation-associated protein AHNAK | AHNAK | 5782 | S | 0.999 | 147.3 | 1.09 | 1.14 | 1.17 | 0.93 | 0.93 | 0.94 | 1.43 | 1.43 | 1.45 |
| Q09666 | Neuroblast differentiation-associated protein AHNAK | AHNAK | 5745 | S | 0.62 | 118.4 | 0.42 | NaN | 0.90 | NaN | NaN | 0.33 | NaN | NaN | NaN |
| Q09666 | Neuroblast differentiation-associated protein AHNAK | AHNAK | 5746 | S | 0.659 | 118.4 | 0.42 | 0.44 | 0.90 | NaN | NaN | 0.33 | NaN | NaN | NaN |
| Q09666 | Neuroblast differentiation-associated protein AHNAK | AHNAK | 3426 | S | 1 | 107.8 | 3.73 | 3.94 | 4.23 | 3.51 | 3.86 | 3.23 | 1.51 | 1.68 | 1.40 |
| Q0JRZ9 | F-BAR domain only protein 2 | FCHO2 | 403 | S | 1 | 80.78 | 1.15 | 1.22 | 1.23 | 0.91 | 0.98 | 0.98 | 1.24 | 1.29 | 1.29 |
| Q12802 | A-kinase anchor protein 13 | AKAP13 | 2398 | S | 0.728 | 61.11 | NaN | 0.76 | 1.23 | 1.17 | NaN | NaN | NaN | NaN | NaN |
| Q12872 | Splicing factor, suppressor of white-apricot homolog | SFSWAP | 604 | S | 0.992 | 174.9 | 0.93 | 0.93 | 0.87 | 0.91 | 0.96 | 0.92 | 0.86 | NaN | 0.85 |
| Q12906 | Interleukin enhancer-binding factor 3 | ILF3 | 482 | S | 1 | 156.2 | 0.73 | 0.69 | NaN | 0.85 | 0.88 | NaN | 0.85 | NaN | 0.84 |
| Q12906 | Interleukin enhancer-binding factor 3 | ILF3 | 382 | S | 1 | 226.1 | 0.55 | 0.55 | 0.55 | 1.06 | 1.07 | 0.95 | 0.36 | 0.37 | 0.35 |
| Q12906 | Interleukin enhancer-binding factor 3 | ILF3 | 384 | S | 0.888 | 96.39 | 0.55 | NaN | 0.55 | 1.01 | 1.06 | NaN | 0.56 | 0.44 | 0.43 |
| Q5T8C6 | Cell division cycle protein 16 homolog | CDC16 | 415 | S | 1 | 48.57 | 1.41 | 1.65 | NaN | NaN | NaN | NaN | NaN | NaN | NaN |
| Q13045 | Protein flightless-1 homolog | FLII | 856 | S | 1 | 154.5 | NaN | 1.02 | 1.05 | NaN | 1.21 | 1.25 | 1.36 | 1.33 | 1.43 |
| Q13085 | Acetyl-CoA carboxylase 1;Biotin carboxylase | ACACA | 23 | S | 0.641 | 109.7 | 0.75 | 0.68 | NaN | NaN | 1.31 | NaN | NaN | NaN | NaN |
| Q13085 | Acetyl-CoA carboxylase 1;Biotin carboxylase | ACACA | 25 | S | 0.88 | 110.6 | NaN | NaN | NaN | 0.92 | 1.31 | 0.96 | 1.93 | NaN | 1.62 |
| Q13085 | Acetyl-CoA carboxylase 1;Biotin carboxylase | ACACA | 29 | S | 1 | 263.7 | 0.68 | 0.64 | 0.70 | 0.79 | 0.68 | 0.78 | 0.78 | 0.73 | 0.83 |
| Q13085 | Acetyl-CoA carboxylase 1;Biotin carboxylase | ACACA | 1259 | S | 0.999 | 210.1 | 0.71 | 0.69 | 0.73 | 0.98 | NaN | 0.85 | 0.75 | NaN | 0.71 |
| Q13085 | Acetyl-CoA carboxylase 1;Biotin carboxylase | ACACA | 1263 | S | 0.976 | 210.1 | 0.71 | 0.69 | 0.73 | 0.98 | NaN | 0.85 | 0.75 | 0.78 | 0.71 |
| Q13111 | Chromatin assembly factor 1 subunit A | CHAF1A | 951 | S | 0.951 | 69.67 | 0.53 | 0.58 | NaN | 0.90 | 0.79 | NaN | 0.70 | 0.65 | 0.87 |
| Q13112 | Chromatin assembly factor 1 subunit B | CHAF1B | 409 | S | 0.564 | 61.28 | NaN | 0.68 | 0.86 | 1.04 | 1.13 | 0.93 | NaN | NaN | NaN |
| Q13112 | Chromatin assembly factor 1 subunit B | CHAF1B | 410 | S | 0.956 | 98.42 | 0.78 | 0.76 | 0.89 | 1.04 | 0.89 | 1.00 | 0.85 | 0.77 | 1.05 |
| Q13112 | Chromatin assembly factor 1 subunit B | CHAF1B | 538 | S | 0.992 | 77.45 | 0.59 | 0.69 | NaN | NaN | NaN | 0.96 | 0.54 | NaN | 0.85 |
| Q13112 | Chromatin assembly factor 1 subunit B | CHAF1B | 429 | S | 0.97 | 160.8 | 0.64 | 0.52 | NaN | 0.65 | NaN | 1.23 | 0.64 | NaN | 0.61 |
| Q13177 | Serine/threonine-protein kinase PAK 2;PAK-2p27;PAK- | PAK2 | 141 | S | 1 | 197.7 | 2.05 | 1.98 | 2.23 | 2.83 | 2.94 | 2.96 | 1.04 | 1.01 | 1.06 |
| Q13185 | Chromobox protein homolog 3 | CBX3 | 93 | S | 1 | 104.4 | 0.63 | 0.93 | 0.66 | 1.04 | 1.44 | 1.32 | 0.74 | 0.79 | 0.93 |
| Q13185 | Chromobox protein homolog 3 | CBX3 | 95 | S | 1 | 309 | 1.03 | 1.06 | 1.08 | 1.10 | 1.05 | 1.01 | 0.86 | 0.85 | 0.64 |
| Q13185 | Chromobox protein homolog 3 | CBX3 | 97 | S | 1 | 107.2 | 1.18 | 1.03 | 0.71 | 1.03 | 0.91 | 1.04 | 0.83 | 0.87 | 0.75 |
| Q13185 | Chromobox protein homolog 3 | CBX3 | 99 | S | 1 | 103.3 | 1.27 | 1.04 | 1.47 | 0.95 | 0.94 | 0.85 | 0.72 | 0.87 | 0.75 |

| | | | | | | | | | | | | | | | |
|---|---|---|---|---|---|---|---|---|---|---|---|---|---|---|---|
| Q13185 | Chromobox protein homolog 3 | CBX3 | 176 | S | 0.966 | 124.2 | 1.06 | 1.25 | 1.35 | 1.06 | 1.17 | 0.93 | 1.07 | 0.99 | 1.15 |
| Q13242 | Serine/arginine-rich splicing factor 9 | SRSF9 | 204 | S | 1 | 108.7 | 1.45 | 1.33 | 1.29 | 1.14 | NaN | 1.00 | 1.37 | 1.35 | NaN |
| Q13242 | Serine/arginine-rich splicing factor 9 | SRSF9 | 211 | S | 1 | 150.5 | 1.02 | 1.06 | 1.03 | 0.93 | 0.90 | 0.96 | 0.97 | 0.97 | 0.99 |
| Q13242 | Serine/arginine-rich splicing factor 9 | SRSF9 | 216 | S | 1 | 119.5 | 0.90 | 1.03 | 1.00 | 1.10 | 0.92 | 0.82 | 1.13 | 1.13 | 1.07 |
| Q13242 | Serine/arginine-rich splicing factor 9 | SRSF9 | 189 | S | 0.928 | 42.16 | 1.15 | 1.03 | NaN | NaN | 0.90 | NaN | NaN | NaN | NaN |
| Q13247 | Serine/arginine-rich splicing factor 6 | SRSF6 | 314 | S | 1 | 78.49 | 1.16 | 1.14 | 1.12 | 1.03 | 1.05 | 1.08 | 1.11 | 1.00 | 1.12 |
| Q13247 | Serine/arginine-rich splicing factor 6 | SRSF6 | 316 | S | 1 | 86.9 | 1.16 | 1.14 | 0.27 | 0.30 | 0.28 | 0.31 | 0.40 | 0.29 | 0.29 |
| Q13247 | Serine/arginine-rich splicing factor 6 | SRSF6 | 303 | S | 1 | 172.3 | 1.05 | 1.02 | 1.15 | 0.98 | 1.00 | 1.01 | 0.91 | 0.91 | 0.95 |
| Q13263 | Transcription intermediary factor 1-beta | TRIM28 | 50 | S | 0.995 | 169.4 | 1.02 | 0.97 | 0.82 | 1.08 | NaN | NaN | 1.08 | NaN | NaN |
| Q13283 | Ras GTPase-activating protein-binding protein 1 | G3BP1 | 232 | S | 1 | 494 | 1.26 | 1.29 | 1.43 | 1.09 | NaN | 1.11 | 1.17 | 1.04 | 1.21 |
| Q13283 | Ras GTPase-activating protein-binding protein 1 | G3BP1 | 149 | S | 1 | 148.2 | 1.20 | 1.32 | 2.34 | 1.64 | NaN | 1.16 | 1.15 | 1.25 | NaN |
| Q13425 | Beta-2-syntrophin | SNTB2 | 95 | S | 1 | 160.4 | NaN | 1.01 | 0.92 | NaN | NaN | 0.95 | NaN | NaN | 0.99 |
| Q13425 | Beta-2-syntrophin | SNTB2 | 110 | S | 1 | 112.7 | 0.68 | 0.58 | NaN | 0.63 | 0.70 | 0.75 | 0.82 | 0.66 | 0.83 |
| Q13425 | Beta-2-syntrophin | SNTB2 | 233 | S | 0.879 | 125.4 | 0.70 | 0.61 | 0.60 | 0.64 | 0.61 | 0.55 | 0.89 | 0.90 | 0.83 |
| Q13428 | Treacle protein | TCOF1 | 1111 | S | 0.965 | 119.4 | NaN | NaN | NaN | 0.90 | 0.87 | NaN | NaN | 0.76 | 0.99 |
| Q13442 | 28 kDa heat- and acid-stable phosphoprotein | PDAP1 | 60 | S | 1 | 571.9 | 0.19 | 0.20 | 0.22 | 0.27 | 0.27 | 0.25 | 0.58 | 0.49 | 0.53 |
| Q13442 | 28 kDa heat- and acid-stable phosphoprotein | PDAP1 | 63 | S | 1 | 398.3 | NaN | NaN | 0.19 | 0.21 | NaN | 0.23 | 0.50 | 0.55 | 0.45 |
| Q13443 | Disintegrin and metalloproteinase domain-containing protein 9 | ADAM9 | 758 | S | 1 | 99.86 | 1.10 | 1.20 | 1.31 | 0.93 | 1.02 | 1.11 | 1.04 | 0.90 | 1.19 |
| Q13469 | Nuclear factor of activated T-cells, cytoplasmic 2 | NFATC2 | 326 | S | 0.983 | 105.1 | NaN | NaN | 1.28 | 0.76 | 0.68 | 0.75 | NaN | 1.71 | NaN |
| Q13501 | Sequestosome-1 | SQSTM1 | 366 | S | 0.951 | 215.3 | 1.56 | 1.48 | 1.60 | 1.95 | NaN | 2.01 | 0.83 | NaN | 0.90 |
| Q13501 | Sequestosome-1 | SQSTM1 | 332 | S | 0.998 | 117 | 0.82 | 0.99 | NaN | NaN | NaN | NaN | NaN | NaN | NaN |
| Q13501 | Sequestosome-1 | SQSTM1 | 272 | S | 1 | 205.3 | 1.14 | 1.70 | 1.12 | 2.61 | 2.65 | 1.45 | 0.64 | 0.61 | 0.61 |
| Q13501 | Sequestosome-1 | SQSTM1 | 24 | S | 0.961 | 145.8 | 3.27 | 2.86 | NaN | 3.83 | 4.80 | 4.30 | NaN | NaN | NaN |
| Q13501 | Sequestosome-1 | SQSTM1 | 28 | S | 0.688 | 100.9 | NaN | NaN | 3.13 | NaN | 4.80 | NaN | 0.86 | 0.77 | NaN |
| Q13501 | Sequestosome-1 | SQSTM1 | 284 | S | 0.95 | 161.7 | 1.09 | 1.26 | NaN | 1.75 | NaN | NaN | NaN | NaN | NaN |
| Q13523 | Serine/threonine-protein kinase PRP4 homolog | PRPF4B | 257 | S | 0.998 | 100.4 | 0.88 | 0.88 | 0.91 | 0.89 | 0.87 | 0.92 | 0.90 | 0.89 | 0.90 |
| Q13523 | Serine/threonine-protein kinase PRP4 homolog | PRPF4B | 431 | S | 1 | 83.82 | NaN | 1.19 | 1.40 | 0.85 | 1.17 | 0.94 | 0.68 | 0.59 | 0.96 |
| Q13523 | Serine/threonine-protein kinase PRP4 homolog | PRPF4B | 437 | S | 0.943 | 72.32 | NaN | 1.19 | 1.40 | 0.95 | 1.17 | 1.23 | 0.68 | 0.59 | 1.55 |

| | | | | | | | | | | | | | | |
|---|---|---|---|---|---|---|---|---|---|---|---|---|---|---|
| Q13523 | Serine/threonine-protein kinase PRP4 homolog | PRPF4B | 20 | S | 1 | 123.6 | 0.52 | 0.45 | 0.40 | NaN | 0.62 | 0.66 | 0.39 | 0.42 | 0.42 |
| Q13523 | Serine/threonine-protein kinase PRP4 homolog | PRPF4B | 277 | S | 1 | 85.99 | 0.88 | 0.91 | 0.84 | NaN | NaN | 0.94 | 0.84 | 0.89 | 0.92 |
| Q13523 | Serine/threonine-protein kinase PRP4 homolog | PRPF4B | 427 | S | 1 | 45.83 | NaN | 1.19 | NaN | NaN | NaN | NaN | NaN | 1.36 | 1.61 |
| Q13523 | Serine/threonine-protein kinase PRP4 homolog | PRPF4B | 368 | S | 1 | 75.48 | 1.05 | 0.48 | NaN | NaN | NaN | NaN | NaN | NaN | NaN |
| Q13523 | Serine/threonine-protein kinase PRP4 homolog | PRPF4B | 578 | S | 0.975 | 98.42 | 0.36 | 0.36 | NaN | NaN | 0.49 | 0.49 | NaN | NaN | NaN |
| Q13523 | Serine/threonine-protein kinase PRP4 homolog | PRPF4B | 580 | S | 1 | 98.42 | 0.36 | 0.36 | NaN | NaN | 0.49 | 0.49 | NaN | NaN | NaN |
| Q13541 | Eukaryotic translation initiation factor 4E-binding protein 1 | EIF4EBP1 | 96 | S | 0.515 | 176.9 | NaN | NaN | NaN | NaN | NaN | NaN | 0.81 | NaN | 0.55 |
| Q13541 | Eukaryotic translation initiation factor 4E-binding protein 1 | EIF4EBP1 | 65 | S | 0.991 | 152.9 | 2.90 | 2.76 | 2.72 | 2.12 | 0.86 | 2.39 | 2.18 | 2.00 | 2.30 |
| Q13547 | Histone deacetylase 1 | HDAC1 | 393 | S | 1 | 144.3 | 0.63 | 0.57 | NaN | 0.92 | 0.77 | 0.67 | 0.54 | 0.56 | 0.55 |
| Q13595 | Transformer-2 protein homolog alpha | TRA2A | 96 | S | 1 | 70.56 | 0.70 | 0.64 | NaN | NaN | NaN | 0.83 | NaN | 0.68 | 0.67 |
| Q13595 | Transformer-2 protein homolog alpha | TRA2A | 98 | S | 1 | 70.56 | 0.70 | 0.64 | NaN | NaN | NaN | 0.83 | NaN | 0.68 | 0.67 |
| Q13595 | Transformer-2 protein homolog alpha | TRA2A | 100 | S | 0.996 | 70.56 | 0.70 | 0.64 | NaN | NaN | NaN | 0.83 | NaN | 0.68 | 0.67 |
| Q13595 | Transformer-2 protein homolog alpha | TRA2A | 84 | S | 0.962 | 61.44 | 1.08 | NaN | 0.97 | 0.90 | 0.83 | 0.94 | 1.32 | 1.24 | 1.25 |
| Q13610 | Periodic tryptophan protein 1 homolog | PWP1 | 50 | S | 1 | 151.5 | 0.55 | 0.49 | 0.50 | 0.61 | 0.66 | 0.64 | 0.69 | 0.62 | 0.72 |
| Q13614 | Myotubularin-related protein 2 | MTMR2 | 630 | S | 0.562 | 54.65 | 1.27 | 1.03 | NaN | 0.70 | 0.60 | NaN | NaN | NaN | NaN |
| Q13625 | Apoptosis-stimulating of p53 protein 2 | TP53BP2 | 698 | S | 0.969 | 65.84 | 0.88 | 0.90 | NaN | 0.96 | NaN | 0.94 | NaN | 0.93 | NaN |
| Q13905 | Rap guanine nucleotide exchange factor 1 | RAPGEF1 | 296 | S | 0.94 | 87.57 | 0.86 | 0.81 | 0.92 | 0.73 | 0.78 | 0.84 | NaN | NaN | 1.08 |
| Q14166 | Tubulin--tyrosine ligase-like protein 12 | TTLL12 | 16 | S | 0.994 | 305.8 | 0.70 | 0.47 | NaN | 0.88 | 0.79 | 0.85 | 0.79 | 0.85 | NaN |
| Q14247 | Src substrate cortactin | CTTN | 405 | S | 1 | 252.6 | 1.08 | 1.02 | 0.68 | 1.04 | 2.76 | 1.90 | 0.83 | 0.89 | 1.44 |
| Q14247 | Src substrate cortactin | CTTN | 417 | S | 0.726 | 196.4 | 0.79 | 0.79 | 3.10 | 1.32 | 1.48 | 0.68 | 1.08 | 0.86 | 0.83 |
| Q14247 | Src substrate cortactin | CTTN | 418 | S | 0.999 | 246.6 | 1.31 | 1.33 | 0.99 | 1.35 | 1.44 | 1.34 | 0.95 | 0.96 | 0.92 |
| Q14315 | Filamin-C | FLNC | 2233 | S | 0.999 | 70.41 | 2.61 | 2.32 | NaN | NaN | NaN | NaN | 3.56 | 4.24 | NaN |
| Q14566 | DNA replication licensing factor MCM6 | MCM6 | 762 | S | 1 | 194.6 | NaN | 1.31 | NaN | NaN | NaN | NaN | 0.74 | 0.66 | 0.95 |
| Q14573 | Inositol 1,4,5-trisphosphate receptor type 3 | ITPR3 | 2670 | S | 1 | 88.51 | 0.93 | 1.05 | 0.88 | NaN | NaN | NaN | 2.32 | 2.23 | 2.45 |
| Q14669 | E3 ubiquitin-protein ligase TRIP12 | TRIP12 | 77 | S | 0.935 | 46.02 | NaN | NaN | NaN | 1.27 | 1.00 | 1.23 | 0.81 | 0.86 | NaN |
| Q14669 | E3 ubiquitin-protein ligase TRIP12 | TRIP12 | 312 | S | 1 | 84.73 | 1.15 | 1.08 | 1.29 | 1.03 | 1.20 | NaN | 1.13 | 1.01 | NaN |

| | | | | | | | | | | | | | | | | |
|---|---|---|---|---|---|---|---|---|---|---|---|---|---|---|---|---|
| Q14669 | E3 ubiquitin-protein ligase TRIP12 | TRIP12 | 1016 | S | 0.934 | 47.02 | NaN | NaN | 0.55 | NaN | 0.60 | NaN | 0.72 | 0.71 | 0.67 |
| Q14676 | Mediator of DNA damage checkpoint protein 1 | MDC1 | 329 | S | 0.995 | 233.6 | 0.75 | 0.84 | 0.83 | 1.25 | NaN | 0.99 | 0.68 | 0.56 | 0.55 |
| Q14676 | Mediator of DNA damage checkpoint protein 1 | MDC1 | 376 | S | 0.998 | 296.4 | 1.04 | 1.01 | 1.03 | 0.95 | 1.43 | 0.99 | 0.88 | 0.79 | 0.93 |
| Q14676 | Mediator of DNA damage checkpoint protein 1 | MDC1 | 168 | S | 1 | 114.6 | 1.01 | 1.04 | 0.95 | NaN | 0.99 | 1.01 | 0.92 | 0.98 | 0.99 |
| Q14676 | Mediator of DNA damage checkpoint protein 1 | MDC1 | 453 | S | 0.953 | 337.1 | 0.98 | 0.95 | 0.91 | 0.97 | NaN | NaN | NaN | NaN | NaN |
| Q14677 | Clathrin interactor 1 | CLINT1 | 299 | S | 0.797 | 106.2 | 0.78 | NaN | NaN | 1.18 | NaN | NaN | 0.83 | 0.77 | 0.54 |
| Q14684 | Ribosomal RNA processing protein 1 homolog B | RRP1B | 732 | S | 0.922 | 91.71 | NaN | NaN | 0.81 | 0.63 | NaN | NaN | NaN | 0.57 | 0.70 |
| Q14684 | Ribosomal RNA processing protein 1 homolog B | RRP1B | 245 | S | 1 | 188.6 | 1.70 | 1.42 | 1.71 | 1.79 | 1.74 | 1.72 | 1.41 | 1.41 | 1.42 |
| Q14764 | Major vault protein | MVP | 873 | S | 1 | 149.7 | 0.86 | NaN | 0.85 | NaN | 1.15 | 1.93 | 1.36 | NaN | 0.97 |
| Q14814 | Myocyte-specific enhancer factor 2D | MEF2D | 251 | S | 0.999 | 75.72 | 1.73 | 1.48 | 1.78 | 1.67 | 1.77 | 1.97 | 1.28 | 1.51 | NaN |
| Q14966 | Zinc finger protein 638 | ZNF638 | 1400 | S | 0.716 | 91.87 | NaN | 2.37 | 1.84 | 2.42 | 2.05 | 2.30 | 1.50 | 1.11 | 1.49 |
| Q14966 | Zinc finger protein 638 | ZNF638 | 1401 | S | 0.5 | 85.73 | NaN | NaN | 1.84 | NaN | 2.05 | NaN | NaN | 1.11 | 1.49 |
| Q14993 | Collagen alpha-1(XIX) chain | COL19A1 | 81 | S | 1 | 50.37 | NaN | 0.68 | 0.77 | NaN | NaN | NaN | NaN | 0.72 | NaN |
| Q14C86 | GTPase-activating protein and VPS9 domain-containing protein 1 | GAPVD1 | 946 | S | 0.649 | 259 | NaN | NaN | NaN | 1.29 | 1.27 | NaN | NaN | NaN | NaN |
| Q15007 | Pre-mRNA-splicing regulator WTAP | WTAP | 306 | S | 0.99 | 288 | 1.06 | 0.99 | NaN | NaN | NaN | NaN | 1.36 | NaN | NaN |
| Q15036 | Sorting nexin-17 | SNX17 | 333 | S | 0.551 | 121.3 | NaN | NaN | NaN | NaN | NaN | NaN | NaN | 0.78 | 0.90 |
| Q15059 | Bromodomain-containing protein 3 | BRD3 | 263 | S | 0.994 | 100.9 | 0.79 | 0.99 | 0.67 | 0.75 | NaN | 0.78 | 0.84 | 0.80 | 0.79 |
| Q15061 | WD repeat-containing protein 43 | WDR43 | 77 | S | 1 | 117.2 | 0.90 | 0.99 | 0.92 | 0.98 | 0.96 | 0.98 | 0.85 | 0.85 | 0.82 |
| Q15084 | Protein disulfide-isomerase A6 | PDIA6 | 428 | S | 1 | 344.7 | 1.11 | 1.19 | 1.28 | 0.98 | 0.96 | 0.98 | 1.41 | 1.11 | 1.00 |
| Q15149 | Plectin | PLEC | 1435 | S | 1 | 108.4 | 1.31 | 1.25 | 1.22 | 1.89 | 2.07 | 1.77 | 0.86 | 1.08 | 0.85 |
| Q15149 | Plectin | PLEC | 720 | S | 1 | 160.5 | 1.40 | 1.39 | 1.42 | 1.73 | 1.75 | 1.61 | NaN | 1.33 | 1.16 |
| Q15149 | Plectin | PLEC | 4613 | S | 1 | 247.4 | 1.26 | 1.35 | 1.16 | 1.26 | 1.36 | 1.34 | 1.96 | 1.86 | 1.94 |
| Q15149 | Plectin | PLEC | 4386 | S | 1 | 366.2 | 1.69 | 1.72 | 1.76 | 1.15 | 1.15 | 1.26 | 2.20 | 2.25 | 2.41 |
| Q15149 | Plectin | PLEC | 4396 | S | 0.999 | 267.6 | 2.80 | 2.67 | 2.36 | 1.87 | 2.23 | 2.18 | 2.31 | 2.08 | 2.26 |
| Q15149 | Plectin | PLEC | 125 | S | 1 | 78.78 | 0.99 | 0.81 | 0.96 | NaN | 1.21 | NaN | NaN | 1.28 | NaN |
| Q15149 | Plectin | PLEC | 4406 | S | 0.896 | 199.2 | 1.10 | 1.30 | 0.97 | 1.30 | NaN | 1.99 | 1.13 | NaN | 1.20 |
| Q15311 | RalA-binding protein 1 | RALBP1 | 645 | S | 0.964 | 59.71 | 0.63 | 0.76 | 0.81 | NaN | 0.80 | 0.79 | 1.04 | 1.00 | 1.00 |
| Q15311 | RalA-binding protein 1 | RALBP1 | 92 | S | 0.999 | 115.4 | 0.72 | 0.67 | 0.86 | 0.65 | 0.67 | 0.67 | 0.97 | 0.93 | 1.00 |
| Q15311 | RalA-binding protein 1 | RALBP1 | 93 | S | 0.999 | 115.4 | 0.72 | 0.67 | 0.86 | 0.65 | 0.67 | 0.67 | 0.97 | 0.93 | 1.00 |
| Q15311 | RalA-binding protein 1 | RALBP1 | 48 | S | 0.988 | 91.44 | 1.44 | 1.55 | NaN | NaN | NaN | NaN | NaN | NaN | NaN |
| Q15311 | RalA-binding protein 1 | RALBP1 | 62 | S | 1 | 91.44 | 1.44 | 1.55 | NaN | NaN | NaN | NaN | NaN | NaN | NaN |
| Q15311 | RalA-binding protein 1 | RALBP1 | 29 | S | 0.716 | 41.52 | NaN | 1.96 | NaN | NaN | NaN | NaN | 1.61 | 1.39 | NaN |
| Q15365 | Poly(rC)-binding protein 1 | PCBP1 | 171 | S | 0.937 | 284.2 | 0.68 | 0.79 | NaN | NaN | NaN | NaN | NaN | NaN | NaN |
| Q15365 | Poly(rC)-binding protein 1 | PCBP1 | 173 | S | 1 | 340.6 | 1.01 | 1.12 | 1.04 | 1.37 | 1.38 | 1.39 | 0.75 | 0.79 | 1.31 |

| | | | | | | | | | | | | | | | |
|---|---|---|---|---|---|---|---|---|---|---|---|---|---|---|---|
| Q15365 | Poly(rC)-binding protein 1 | PCBP1 | 190 | S | 0.961 | 122.5 | 1.59 | 1.75 | 1.67 | 2.95 | NaN | 2.91 | 1.03 | 0.97 | 1.09 |
| Q15366 | Poly(rC)-binding protein 2 | PCBP2 | 189 | S | 0.652 | 127.2 | NaN | 2.06 | 1.67 | NaN | NaN | NaN | NaN | 1.06 | NaN |
| Q15366 | Poly(rC)-binding protein 2 | PCBP2 | 173 | S | 0.996 | 93.5 | 0.60 | 0.43 | NaN | 0.72 | 0.88 | NaN | 0.61 | 0.53 | NaN |
| Q15398 | Disks large-associated protein 5 | DLGAP5 | 662 | S | 0.987 | 79.46 | NaN | 0.77 | NaN | NaN | 1.76 | NaN | 0.47 | 0.38 | 0.52 |
| Q15398 | Disks large-associated protein 5 | DLGAP5 | 690 | S | 0.775 | 108.7 | 4.96 | 3.27 | NaN | NaN | NaN | NaN | NaN | NaN | NaN |
| Q15424 | Scaffold attachment factor B1 | SAFB | 383 | S | 1 | 222 | 0.65 | 0.66 | 0.65 | 0.99 | 0.94 | 0.98 | 0.52 | 0.55 | 0.57 |
| Q15424 | Scaffold attachment factor B1 | SAFB | 384 | S | 0.998 | 222 | 0.65 | 0.66 | 0.65 | 0.99 | 0.99 | 0.98 | 0.52 | 0.55 | 0.57 |
| Q15424 | Scaffold attachment factor B1 | SAFB | 604 | S | 1 | 113.8 | 1.07 | 1.12 | 0.99 | 1.14 | 1.05 | 1.03 | 1.17 | 1.43 | NaN |
| Q15424 | Scaffold attachment factor B1;Scaffold attachment factor B2 | SAFB | 344 | S | 0.969 | 101.2 | NaN | 1.53 | NaN | NaN | 1.74 | NaN | 1.55 | 1.75 | NaN |
| Q15459 | Splicing factor 3A subunit 1 | SF3A1 | 451 | S | 0.994 | 55.03 | 1.58 | 0.94 | NaN | 1.51 | NaN | NaN | NaN | NaN | NaN |
| Q15459 | Splicing factor 3A subunit 1 | SF3A1 | 329 | S | 1 | 211.1 | 0.98 | 0.78 | 0.93 | 0.91 | 1.05 | 0.95 | 1.04 | 0.59 | 0.61 |
| Q15477 | Helicase SKI2W | SKIV2L | 256 | S | 0.999 | 145.1 | 0.17 | 0.14 | NaN | 0.22 | 0.19 | 0.19 | 1.01 | 1.00 | 0.91 |
| Q15545 | Transcription initiation factor TFIID subunit 7 | TAF7 | 201 | S | 0.8 | 65.75 | 1.45 | NaN | NaN | 1.33 | 1.03 | 1.46 | 1.05 | 0.88 | 1.03 |
| Q15637 | Splicing factor 1 | SF1 | 80 | S | 1 | 188.3 | 1.02 | 1.06 | 0.95 | 1.00 | 0.98 | 0.96 | 0.84 | 0.87 | 0.91 |
| Q15637 | Splicing factor 1 | SF1 | 82 | S | 1 | 188.3 | 1.02 | 1.06 | 0.95 | 1.00 | 1.01 | 0.96 | 0.84 | 0.87 | 0.91 |
| Q15648 | Mediator of RNA polymerase II transcription subunit 1 | MED1 | 1049 | S | 0.544 | 44.2 | 0.64 | 0.79 | NaN | NaN | NaN | 0.81 | NaN | NaN | NaN |
| Q15648 | Mediator of RNA polymerase II transcription subunit 1 | MED1 | 1479 | S | 0.998 | 85 | 0.80 | 0.65 | 0.74 | 0.87 | 0.89 | 0.91 | NaN | 0.78 | 0.69 |
| Q15648 | Mediator of RNA polymerase II transcription subunit 1 | MED1 | 1481 | S | 0.566 | 59.66 | NaN | NaN | 0.74 | NaN | 0.89 | 0.91 | NaN | NaN | NaN |
| Q15648 | Mediator of RNA polymerase II transcription subunit 1 | MED1 | 1482 | S | 0.805 | 85 | 0.80 | 0.65 | NaN | 0.87 | NaN | NaN | NaN | 0.78 | 0.69 |
| Q15678 | Tyrosine-protein phosphatase non-receptor type 14 | PTPN14 | 314 | S | 0.995 | 85.74 | 0.98 | NaN | 1.01 | NaN | NaN | 0.77 | 1.25 | 1.06 | 1.26 |
| Q15678 | Tyrosine-protein phosphatase non-receptor type 14 | PTPN14 | 593 | S | 0.998 | 97.2 | NaN | 1.41 | 1.26 | 0.90 | NaN | 0.89 | 1.30 | 1.72 | 1.67 |
| Q15678 | Tyrosine-protein phosphatase non-receptor type 14 | PTPN14 | 594 | S | 0.999 | 175.9 | 0.95 | 0.93 | 1.26 | 0.87 | 0.91 | 0.89 | 1.65 | 1.72 | 1.67 |
| Q15942 | Zyxin | ZYX | 281 | S | 1 | 105.6 | NaN | NaN | 1.73 | 1.81 | 2.30 | 2.75 | NaN | 1.23 | NaN |
| Q15942 | Zyxin | ZYX | 267 | S | 1 | 93.7 | 1.57 | 1.47 | 1.77 | 1.99 | 2.03 | 1.92 | 1.33 | 1.28 | 1.28 |
| Q15942 | Zyxin | ZYX | 259 | S | 0.994 | 87.21 | 7.05 | 9.04 | NaN | 5.03 | NaN | NaN | 4.90 | 3.94 | 4.06 |
| Q15942 | Zyxin | ZYX | 344 | S | 1 | 57.57 | 1.94 | 2.04 | NaN | 2.13 | 2.12 | NaN | 1.01 | 1.04 | 1.04 |
| Q16204 | Coiled-coil domain-containing protein 6 | CCDC6 | 244 | S | 1 | 176.3 | 1.74 | 1.69 | 1.62 | 1.87 | 1.89 | 1.76 | 1.10 | 1.09 | 1.05 |
| Q16204 | Coiled-coil domain-containing protein 6 | CCDC6 | 419 | S | 0.999 | 85.54 | 0.89 | 0.93 | 0.85 | 0.82 | 0.80 | 0.88 | 0.99 | 0.88 | 0.91 |
| Q16512 | Serine/threonine-protein kinase N1 | PKN1 | 562 | S | 0.74 | 118 | NaN | NaN | NaN | 0.75 | 0.99 | NaN | NaN | NaN | NaN |
| Q16512 | Serine/threonine-protein kinase N1 | PKN1 | 916 | S | 0.999 | 273.3 | NaN | 1.02 | 0.98 | 0.74 | 0.89 | NaN | 0.98 | 1.17 | 1.04 |

| | | | | | | | | | | | | | | | |
|---|---|---|---|---|---|---|---|---|---|---|---|---|---|---|---|
| Q16513 | Serine/threonine-protein kinase N2 | PKN2 | 583 | S | 1 | 208.5 | 0.84 | 1.00 | 0.98 | 0.93 | 0.99 | 1.14 | 0.95 | 0.93 | 1.03 |
| Q16514 | Transcription initiation factor TFIID subunit 12 | TAF12 | 51 | S | 0.838 | 59.28 | NaN | 0.99 | NaN | NaN | NaN | NaN | 0.91 | 0.93 | NaN |
| Q16643 | Drebrin | DBN1 | 142 | S | 0.998 | 99.26 | 0.91 | 1.00 | 0.97 | 0.78 | 0.79 | 0.79 | 1.31 | 1.39 | 1.44 |
| Q1RLN5 | Rho GTPase-activating protein 12 | ARHGAP 12 | 240 | S | 1 | 117.9 | 0.59 | 0.60 | 0.65 | 0.84 | 0.87 | 0.70 | 0.86 | 0.78 | 0.80 |
| Q27J81 | Inverted formin-2 | INF2 | 1149 | S | 0.876 | 88.74 | 1.27 | 1.13 | NaN | 0.66 | NaN | NaN | NaN | 1.19 | NaN |
| Q2M2I8 | AP2-associated protein kinase 1 | AAK1 | 623 | S | 0.866 | 131.1 | 1.29 | NaN | 1.41 | 1.26 | 1.28 | 1.34 | 1.53 | 1.48 | 1.49 |
| Q32MZ4 | Leucine-rich repeat flightless-interacting protein 1 | LRRFIP1 | 733 | S | 0.999 | 327.2 | NaN | NaN | 0.72 | 0.87 | 0.85 | 0.90 | NaN | 1.23 | 1.24 |
| Q32MZ4 | Leucine-rich repeat flightless-interacting protein 1 | LRRFIP1 | 714 | S | 0.988 | 205.5 | 0.50 | 0.51 | NaN | 0.80 | 0.84 | 0.80 | NaN | 0.93 | 0.93 |
| Q32MZ4 | Leucine-rich repeat flightless-interacting protein 1 | LRRFIP1 | 120 | S | 0.928 | 114.5 | 1.30 | NaN | NaN | NaN | NaN | 1.14 | 1.25 | NaN | 1.28 |
| Q32MZ4 | Leucine-rich repeat flightless-interacting protein 1 | LRRFIP1 | 521 | S | 0.984 | 155.3 | NaN | NaN | NaN | 1.58 | NaN | 0.87 | 0.85 | NaN | NaN |
| Q3KQU3 | MAP7 domain-containing protein 1 | MAP7D1 | 741 | S | 0.791 | 127.9 | NaN | 0.56 | 0.54 | NaN | NaN | NaN | NaN | 0.68 | NaN |
| Q3KQU3 | MAP7 domain-containing protein 1 | MAP7D1 | 544 | S | 0.998 | 96.56 | 1.32 | 1.02 | 2.08 | 1.16 | NaN | 1.68 | 1.12 | 1.63 | 0.88 |
| Q3KQU3 | MAP7 domain-containing protein 1 | MAP7D1 | 552 | S | 0.981 | 96.56 | 1.32 | 2.32 | 2.08 | NaN | NaN | 1.68 | 1.55 | 1.63 | 1.36 |
| Q3KQU3 | MAP7 domain-containing protein 1 | MAP7D1 | 460 | S | 0.965 | 79.27 | NaN | 2.01 | 2.21 | 1.93 | 2.05 | 1.95 | 1.30 | 1.15 | 1.17 |
| Q3KQU3 | MAP7 domain-containing protein 1 | MAP7D1 | 86 | S | 0.862 | 147.4 | NaN | 1.27 | 1.11 | 0.54 | NaN | 1.47 | 0.90 | NaN | 1.22 |
| Q3KQU3 | MAP7 domain-containing protein 1 | MAP7D1 | 113 | S | 0.774 | 59.05 | 1.44 | 1.42 | 1.28 | 1.17 | 1.17 | 1.03 | 0.90 | 0.82 | 0.89 |
| Q3KQU3 | MAP7 domain-containing protein 1 | MAP7D1 | 116 | S | 0.837 | 59.05 | 1.44 | 1.42 | 1.28 | 1.17 | 1.17 | 1.31 | 0.90 | 0.82 | 0.89 |
| Q4G0J3 | La-related protein 7 | LARP7 | 337 | S | 1 | 115.3 | 1.07 | 1.03 | 1.12 | NaN | 1.20 | 0.91 | 1.31 | 1.14 | 0.97 |
| Q4G0J3 | La-related protein 7 | LARP7 | 261 | S | 1 | 144.3 | 0.88 | 0.89 | NaN | 1.31 | NaN | NaN | NaN | 0.94 | NaN |
| Q4KMP7 | TBC1 domain family member 10B | TBC1D10 B | 678 | S | 1 | 59.25 | 1.24 | 1.19 | 1.37 | 1.07 | NaN | 1.14 | NaN | 1.20 | 1.22 |
| Q4KMP7 | TBC1 domain family member 10B | TBC1D10 B | 132 | S | 1 | 120 | NaN | 0.38 | 0.96 | 0.64 | 0.87 | 0.65 | NaN | 1.19 | 1.21 |
| Q4L180 | Filamin A-interacting protein 1-like | FILIP1L | 791 | S | 1 | 68.17 | 0.84 | 0.87 | NaN | NaN | NaN | NaN | NaN | NaN | NaN |
| Q4VCS5 | Angiomotin | AMOT | 4 | S | 1 | 61.17 | 0.83 | NaN | 0.87 | 0.70 | 0.78 | 0.74 | 0.90 | 0.94 | NaN |
| Q4VCS5 | Angiomotin | AMOT | 9 | S | 0.691 | 61.17 | 0.83 | NaN | 0.87 | 0.70 | 0.78 | 0.74 | 0.90 | 0.94 | NaN |
| Q52LW3 | Rho GTPase-activating protein 29 | ARHGAP 29 | 949 | S | 1 | 151.1 | 1.20 | 1.23 | 1.22 | 1.33 | 1.42 | 1.34 | 1.30 | 1.41 | 1.31 |
| Q52LW3 | Rho GTPase-activating protein 29 | ARHGAP 29 | 589 | S | 0.999 | 132.1 | NaN | NaN | NaN | NaN | 0.49 | 0.88 | 0.46 | 0.79 | 0.79 |

| | | | | | | | | | | | | | | |
|---|---|---|---|---|---|---|---|---|---|---|---|---|---|---|
| Q52LW3 | Rho GTPase-activating protein 29 | ARHGAP 29 | 1185 | S | 0.985 | 106.9 | NaN | 1.52 | 1.67 | 2.09 | 2.18 | 1.70 | 1.08 | 1.00 | 1.05 |
| Q52LW3 | Rho GTPase-activating protein 29 | ARHGAP 29 | 190 | S | 1 | 259.2 | 1.09 | 0.98 | 1.60 | 1.73 | 1.83 | 1.94 | 0.48 | 0.30 | 0.72 |
| Q52LW3 | Rho GTPase-activating protein 29 | ARHGAP 29 | 499 | S | 1 | 106.4 | 0.87 | NaN | 1.13 | NaN | 0.88 | NaN | 1.46 | 1.54 | NaN |
| Q52LW3 | Rho GTPase-activating protein 29 | ARHGAP 29 | 913 | S | 1 | 210.1 | 0.95 | 1.02 | 0.84 | 1.61 | 1.54 | 1.37 | 0.55 | NaN | 0.48 |
| Q52LW3 | Rho GTPase-activating protein 29 | ARHGAP 29 | 1029 | S | 1 | 142.1 | NaN | 1.05 | 1.01 | 1.31 | 1.33 | 1.34 | 0.92 | 0.93 | 0.94 |
| Q52LW3 | Rho GTPase-activating protein 29 | ARHGAP 29 | 171 | S | 1 | 161.9 | NaN | NaN | 0.72 | 0.70 | 0.63 | NaN | 1.10 | 1.28 | 1.34 |
| Q52LW3 | Rho GTPase-activating protein 29 | ARHGAP 29 | 559 | S | 0.976 | 173 | 0.60 | NaN | 0.67 | 0.96 | 0.87 | 0.88 | 0.68 | 0.74 | NaN |
| Q5VZS7 | Programmed cell death protein 4 | PDCD4 | 80 | S | 0.752 | 155.2 | 0.76 | 0.75 | NaN | NaN | NaN | 0.87 | NaN | NaN | 1.08 |
| Q53EZ4 | Centrosomal protein of 55 kDa | CEP55 | 428 | S | 0.978 | 171 | 1.58 | 1.61 | 1.59 | 2.32 | 1.03 | 1.81 | 0.66 | 0.75 | 0.66 |
| Q53EZ4 | Centrosomal protein of 55 kDa | CEP55 | 425 | S | 1 | 171 | 1.68 | 1.65 | 2.04 | 2.32 | 2.70 | 2.77 | 0.76 | NaN | 0.86 |
| Q53F19 | Uncharacterized protein C17orf85 | C17orf85 | 415 | S | 0.993 | 77.74 | NaN | NaN | NaN | 1.09 | 1.19 | 0.99 | 0.44 | NaN | NaN |
| Q58WW2 | DDB1- and CUL4-associated factor 6 | DCAF6 | 657 | S | 0.999 | 76.2 | 0.61 | 0.62 | 0.62 | NaN | NaN | NaN | NaN | NaN | NaN |
| Q5C9Z4 | Nucleolar MIF4G domain-containing protein 1 | NOM1 | 139 | S | 0.978 | 47.92 | NaN | 0.86 | 1.03 | NaN | 0.88 | 0.98 | NaN | 0.96 | NaN |
| Q5H909 | Melanoma-associated antigen D2 | MAGED2 | 173 | S | 0.999 | 233.3 | 0.63 | 0.70 | 0.66 | 0.64 | NaN | 0.65 | 1.02 | 1.10 | 1.02 |
| Q5JPT2 | SH3 domain-containing kinase-binding protein 1 | SH3KBP1 | 567 | S | 0.999 | 133.2 | 3.33 | 4.27 | 3.89 | 2.43 | 2.83 | 2.41 | 2.93 | 2.95 | 2.80 |
| Q5JSH3 | WD repeat-containing protein 44 | WDR44 | 96 | S | 0.985 | 172.1 | NaN | 2.11 | 2.20 | NaN | NaN | 1.83 | 2.90 | 2.44 | 1.71 |
| Q5JSH3 | WD repeat-containing protein 44 | WDR44 | 403 | S | 1 | 367 | 0.71 | 0.77 | 0.84 | 0.87 | 0.83 | 0.90 | 1.04 | 1.10 | 1.10 |
| Q5JSH3 | WD repeat-containing protein 44 | WDR44 | 162 | S | 0.702 | 184 | 0.82 | NaN | 0.99 | NaN | 0.94 | NaN | 1.25 | NaN | NaN |
| Q5JSH3 | WD repeat-containing protein 44 | WDR44 | 50 | S | 1 | 111.7 | 0.96 | NaN | NaN | 0.96 | 0.96 | 0.99 | 1.14 | 1.19 | NaN |
| Q5JSZ5 | Protein PRRC2B | PRRC2B | 613 | S | 1 | 59.12 | 1.30 | 1.37 | 1.70 | 1.27 | 1.48 | 1.25 | 0.96 | 1.02 | 0.81 |
| Q5JSZ5 | Protein PRRC2B | PRRC2B | 556 | S | 0.977 | 82.36 | NaN | 2.54 | 1.97 | 2.44 | 2.07 | 2.61 | 1.39 | 1.23 | 1.74 |
| Q5JTD0 | Tight junction-associated protein 1 | TJAP1 | 300 | S | 1 | 281.5 | 0.86 | 0.89 | 0.85 | 0.94 | 0.97 | 0.92 | 0.80 | 0.96 | 0.92 |
| Q5JTH9 | RRP12-like protein | RRP12 | 1080 | S | 1 | 223.4 | 0.89 | 0.69 | 0.88 | 0.95 | 0.94 | 0.81 | 1.06 | 1.03 | 0.96 |
| Q5M775 | Cytospin-B | SPECC1 | 134 | S | 0.991 | 134.9 | 2.50 | 2.50 | 2.06 | NaN | 1.25 | 1.46 | NaN | NaN | 1.49 |
| Q5M775 | Cytospin-B | SPECC1 | 810 | S | 0.955 | 66.92 | NaN | 1.54 | 1.55 | NaN | 1.22 | 1.15 | NaN | 1.54 | 1.43 |
| Q5S007 | Leucine-rich repeat serine/threonine-protein kinase 2 | LRRK2 | 954 | S | 0.998 | 72.73 | NaN | NaN | NaN | 1.05 | 1.10 | NaN | NaN | 1.13 | NaN |

| | | | | | | | | | | | | | | | |
|---|---|---|---|---|---|---|---|---|---|---|---|---|---|---|---|
| Q5S007 | Leucine-rich repeat serine/threonine-protein kinase 2 | LRRK2 | 955 | S | 0.992 | 72.73 | NaN | NaN | NaN | 1.05 | 1.10 | NaN | NaN | 1.13 | NaN |
| Q5SQI0 | Alpha-tubulin N-acetyltransferase 1 | ATAT1 | 276 | S | 1 | 71.22 | NaN | NaN | NaN | 0.61 | 0.68 | 0.41 | 0.98 | 0.72 | NaN |
| Q5SRE5 | Nucleoporin NUP188 homolog | NUP188 | 1709 | S | 0.966 | 68.49 | 1.39 | 1.37 | NaN | NaN | NaN | NaN | NaN | NaN | NaN |
| Q5STZ8 | ATP-binding cassette sub-family F member 1 | ABCF1 | 229 | S | 1 | 225.4 | 0.64 | 0.63 | 0.79 | 0.84 | 0.76 | 0.88 | 0.77 | 0.79 | 0.84 |
| Q5STZ8 | ATP-binding cassette sub-family F member 1 | ABCF1 | 106 | S | 1 | 108 | 0.53 | 0.45 | 0.48 | 0.65 | 0.75 | 0.70 | 1.09 | 1.08 | 1.03 |
| Q5STZ8 | ATP-binding cassette sub-family F member 1 | ABCF1 | 110 | S | 1 | 230.7 | 0.77 | 0.91 | 0.76 | 0.88 | 0.94 | 0.89 | 0.57 | 0.50 | 0.59 |
| Q5STZ8 | ATP-binding cassette sub-family F member 1 | ABCF1 | 22 | S | 0.961 | 146.1 | 0.83 | 0.88 | 0.73 | 1.19 | 1.20 | 1.13 | 0.85 | 0.86 | 0.69 |
| Q5T200 | Zinc finger CCCH domain-containing protein 13 | ZC3H13 | 198 | S | 1 | 93.62 | 0.81 | 0.89 | NaN | 1.05 | 0.95 | NaN | NaN | 0.87 | 0.97 |
| Q5T200 | Zinc finger CCCH domain-containing protein 13 | ZC3H13 | 993 | S | 1 | 129.9 | 1.73 | 1.11 | NaN | 1.33 | 1.38 | NaN | NaN | NaN | NaN |
| Q5T200 | Zinc finger CCCH domain-containing protein 13 | ZC3H13 | 265 | S | 1 | 78.99 | 0.82 | 0.84 | 0.91 | 0.82 | 0.88 | 1.04 | 0.81 | 0.98 | 0.75 |
| Q5T200 | Zinc finger CCCH domain-containing protein 13 | ZC3H13 | 110 | S | 0.732 | 49.15 | 1.03 | NaN | 0.91 | NaN | 1.03 | 0.92 | 1.02 | 0.83 | NaN |
| Q5T200 | Zinc finger CCCH domain-containing protein 13 | ZC3H13 | 77 | S | 1 | 56.87 | 0.73 | NaN | NaN | 0.74 | 0.85 | NaN | NaN | NaN | NaN |
| Q5T2Z0 | Proline/serine-rich coiled-coil protein 1 | PSRC1 | 189 | S | 0.635 | 56.71 | NaN | NaN | 0.71 | 0.67 | NaN | 0.74 | 0.90 | 1.05 | 0.96 |
| Q5T4S7 | E3 ubiquitin-protein ligase UBR4 | UBR4 | 619 | S | 0.911 | 124.4 | NaN | NaN | NaN | 1.14 | 1.08 | 1.30 | 1.06 | 1.12 | 0.96 |
| Q5T4S7 | E3 ubiquitin-protein ligase UBR4 | UBR4 | 620 | S | 0.621 | 124.4 | NaN | 0.77 | 0.80 | 1.14 | NaN | 1.66 | 1.20 | 1.12 | 0.96 |
| Q5T4S7 | E3 ubiquitin-protein ligase UBR4 | UBR4 | 178 | S | 0.978 | 77.24 | 1.25 | 1.35 | 1.67 | 0.48 | NaN | NaN | 0.77 | NaN | NaN |
| Q5T4S7 | E3 ubiquitin-protein ligase UBR4 | UBR4 | 2719 | S | 0.983 | 131.5 | 1.02 | NaN | 0.98 | NaN | 0.96 | 0.95 | 0.95 | 0.94 | 0.99 |
| Q5T4S7 | E3 ubiquitin-protein ligase UBR4 | UBR4 | 362 | S | 0.97 | 215.5 | 0.92 | 0.89 | 0.91 | 1.19 | NaN | 1.27 | 1.08 | NaN | 1.49 |
| Q5T5P2 | Sickle tail protein homolog | KIAA1217 | 1044 | S | 1 | 76.53 | 0.90 | 1.03 | NaN | 1.43 | 1.44 | NaN | NaN | NaN | NaN |
| Q5T5U3 | Rho GTPase-activating protein 21 | ARHGAP 21 | 1504 | S | 0.94 | 56.71 | NaN | 1.00 | 0.77 | 1.07 | NaN | 0.86 | NaN | 0.75 | NaN |
| Q5T5Y3 | Calmodulin-regulated spectrin-associated protein 1 | CAMSAP 1 | 563 | S | 1 | 81.43 | NaN | 1.33 | 1.63 | 1.00 | 1.14 | 1.13 | 1.37 | 1.24 | NaN |
| Q5TBR0 | Sialic acid synthase | NANS | 83 | S | 0.994 | 91.32 | 1.04 | 0.77 | 0.81 | 0.91 | 0.77 | 1.17 | 1.14 | 0.95 | 0.98 |
| Q5UIP0 | Telomere-associated protein RIF1 | RIF1 | 2393 | S | 0.998 | 75.65 | 0.75 | 0.65 | 0.68 | 0.93 | 0.94 | 0.77 | 0.55 | 0.77 | NaN |
| Q5VSL9 | Striatin-interacting protein 1 | STRIP1 | 335 | S | 1 | 170.1 | 0.80 | 0.70 | 0.85 | 0.78 | 1.00 | 0.83 | 1.05 | NaN | 0.98 |

| | | | | | | | | | | | | | | | |
|---|---|---|---|---|---|---|---|---|---|---|---|---|---|---|---|
| Q5VT52 | Regulation of nuclear pre-mRNA domain-containing protein 2 | RPRD2 | 374 | S | 1 | 193 | 0.87 | 1.00 | 1.23 | 1.23 | 1.07 | 1.34 | 0.88 | 0.91 | NaN |
| Q5VT52 | Regulation of nuclear pre-mRNA domain-containing protein 2 | RPRD2 | 485 | S | 0.632 | 103.1 | 1.03 | 0.94 | NaN | 1.11 | NaN | NaN | 0.99 | NaN | NaN |
| Q5VTR2 | E3 ubiquitin-protein ligase BRE1A | RNF20 | 138 | S | 0.969 | 97.9 | NaN | NaN | NaN | NaN | NaN | NaN | 1.42 | 1.64 | 1.10 |
| Q5VUA4 | Zinc finger protein 318 | ZNF318 | 173 | S | 1 | 73.77 | 0.61 | 0.59 | NaN | 0.71 | 0.72 | NaN | NaN | 0.57 | NaN |
| Q5VUB5 | Protein FAM171A1 | FAM171A1 | 849 | S | 0.999 | 51.69 | NaN | NaN | NaN | 1.25 | 0.99 | 2.28 | NaN | 0.90 | 1.57 |
| Q5VWV2 | Partitioning defective 3 | PARD3 | 792 | S | 1 | 47.62 | 0.69 | NaN | NaN | NaN | NaN | NaN | 0.72 | 0.68 | 0.64 |
| Q5VWV2 | Partitioning defective 3 | PARD3 | 635 | S | 0.557 | 67.25 | 0.89 | 1.25 | NaN | NaN | 1.38 | NaN | NaN | 1.09 | 1.13 |
| Q5VWV2 | Partitioning defective 3 | PARD3 | 638 | S | 0.526 | 58.95 | 0.89 | 1.25 | NaN | NaN | NaN | NaN | 1.03 | NaN | 1.13 |
| Q5VY60 | HAUS augmin-like complex subunit 6 | HAUS6 | 416 | S | 0.997 | 106.4 | NaN | NaN | 1.28 | NaN | 1.56 | 2.65 | 1.08 | 1.51 | 0.96 |
| Q5VZ89 | DENN domain-containing protein 4C | DENND4C | 1125 | S | 0.5 | 135.9 | 0.59 | 0.68 | 0.90 | 0.69 | NaN | NaN | NaN | NaN | NaN |
| Q5VZ89 | DENN domain-containing protein 4C | DENND4C | 1126 | S | 0.5 | 135.9 | 0.59 | 0.68 | 0.90 | 0.69 | NaN | NaN | NaN | NaN | NaN |
| Q5VZL5 | Zinc finger MYM-type protein 4 | ZMYM4 | 122 | S | 0.993 | 135.3 | 0.18 | NaN | NaN | NaN | 0.54 | 0.66 | 0.89 | NaN | 1.49 |
| Q63ZY6 | Putative methyltransferase NSUN5C | NSUN5P2 | 260 | S | 0.999 | 42.63 | NaN | NaN | 0.59 | NaN | 1.12 | 0.72 | NaN | NaN | 0.60 |
| Q66K14 | TBC1 domain family member 9B | TBC1D9B | 435 | S | 0.956 | 128.2 | 1.04 | 0.92 | 0.92 | 1.06 | 0.65 | 1.14 | 1.26 | 1.17 | 1.43 |
| Q66K14 | TBC1 domain family member 9B | TBC1D9B | 994 | S | 0.693 | 66.96 | NaN | NaN | 1.06 | NaN | 1.21 | NaN | 0.86 | 0.88 | NaN |
| Q66K74 | Microtubule-associated protein 1S;MAP1S heavy chain;MAP1S light chain | MAP1S | 631 | S | 0.567 | 97.06 | NaN | NaN | NaN | 0.82 | 0.62 | NaN | NaN | NaN | NaN |
| Q66K74 | Microtubule-associated protein 1S;MAP1S heavy chain;MAP1S light chain | MAP1S | 632 | S | 0.7 | 162 | NaN | NaN | NaN | 0.84 | 0.62 | NaN | 1.15 | NaN | NaN |
| Q66K74 | Microtubule-associated protein 1S;MAP1S heavy chain;MAP1S light chain | MAP1S | 640 | S | 0.997 | 176 | 0.94 | 0.94 | 0.91 | 0.86 | 0.93 | 0.87 | 1.08 | 1.04 | 1.08 |
| Q66K74 | Microtubule-associated protein 1S;MAP1S heavy chain;MAP1S light chain | MAP1S | 643 | S | 0.511 | 54.98 | NaN | NaN | NaN | 0.99 | 0.56 | NaN | NaN | NaN | NaN |
| Q66K74 | Microtubule-associated protein 1S;MAP1S heavy chain;MAP1S light chain | MAP1S | 759 | S | 1 | 237.3 | 0.56 | 0.59 | 0.62 | 0.87 | 0.83 | 0.84 | 0.66 | 0.68 | 0.69 |
| Q66K74 | Microtubule-associated protein 1S;MAP1S heavy chain;MAP1S light chain | MAP1S | 592 | S | 0.899 | 64.38 | 0.60 | NaN | 0.41 | 0.98 | NaN | 0.71 | 1.06 | NaN | 0.93 |

| | | | | | | | | | | | | | | |
|---|---|---|---|---|---|---|---|---|---|---|---|---|---|---|
| Q66K74 | Microtubule-associated protein 1S;MAP1S heavy chain;MAP1S light chain | MAP1S | 582 | S | 0.986 | 66.08 | NaN | NaN | 0.55 | 0.77 | NaN | 0.85 | 0.89 | 0.86 | NaN |
| Q66K74 | Microtubule-associated protein 1S;MAP1S heavy chain;MAP1S light chain | MAP1S | 731 | S | 0.845 | 111.7 | 1.33 | 1.15 | NaN | NaN | NaN | NaN | 1.19 | 1.35 | NaN |
| Q68CZ2 | Tensin-3 | TNS3 | 1154 | S | 1 | 65.74 | 0.65 | 0.90 | NaN | NaN | 1.25 | 1.21 | 0.55 | 0.80 | 0.71 |
| Q68CZ2 | Tensin-3 | TNS3 | 690 | S | 0.937 | 169.8 | 0.85 | NaN | NaN | 0.92 | 0.97 | 0.87 | NaN | NaN | NaN |
| Q68CZ2 | Tensin-3 | TNS3 | 660 | S | 0.995 | 148.3 | 0.74 | 0.84 | 0.81 | 1.20 | 1.32 | 1.44 | 0.62 | 0.57 | 0.58 |
| Q68CZ2 | Tensin-3 | TNS3 | 776 | S | 0.972 | 60.1 | 1.11 | 0.87 | NaN | NaN | 1.60 | NaN | NaN | NaN | NaN |
| Q68CZ2 | Tensin-3 | TNS3 | 1441 | S | 1 | 64.71 | 0.46 | 0.41 | 0.47 | 0.90 | NaN | 0.70 | 0.61 | 0.52 | NaN |
| Q68DQ2 | Very large A-kinase anchor protein | CRYBG3 | 1280 | S | 0.97 | 65.11 | 1.03 | 0.89 | NaN | NaN | NaN | NaN | NaN | NaN | NaN |
| Q68E01 | Integrator complex subunit 3 | INTS3 | 502 | S | 0.999 | 65.65 | NaN | NaN | NaN | 0.72 | NaN | NaN | 0.95 | 0.93 | NaN |
| Q68EM7 | Rho GTPase-activating protein 17 | ARHGAP 17 | 575 | S | 1 | 208.1 | 0.57 | 0.62 | NaN | 0.71 | 0.75 | 0.90 | NaN | NaN | 0.79 |
| Q68EM7 | Rho GTPase-activating protein 17 | ARHGAP 17 | 676 | S | 0.997 | 188.3 | 0.85 | 0.90 | 0.96 | 0.89 | 0.96 | 0.87 | 1.00 | 1.05 | 1.09 |
| Q69YN4 | Protein virilizer homolog | KIAA1429 | 133 | S | 0.544 | 67.31 | 1.03 | 0.95 | 1.01 | 0.95 | NaN | NaN | NaN | NaN | NaN |
| Q69YN4 | Protein virilizer homolog | KIAA1429 | 138 | S | 0.883 | 105.4 | 1.03 | 0.95 | NaN | 0.95 | 1.06 | NaN | 0.96 | 0.89 | 1.04 |
| Q6GQQ9 | OTU domain-containing protein 7B | OTUD7B | 464 | S | 0.998 | 84.74 | 1.23 | 1.35 | NaN | 1.32 | 1.77 | NaN | NaN | 1.55 | NaN |
| Q6GQQ9 | OTU domain-containing protein 7B | OTUD7B | 467 | S | 0.999 | 84.74 | 2.15 | 1.35 | NaN | 1.32 | 1.77 | NaN | NaN | 1.55 | NaN |
| Q6IQ49 | Protein SDE2 homolog | SDE2 | 278 | S | 0.651 | 90.13 | 1.27 | 1.08 | NaN | 1.15 | NaN | 0.92 | 0.97 | 0.99 | NaN |
| Q6KC79 | Nipped-B-like protein | NIPBL | 350 | S | 0.627 | 124.7 | 1.07 | 0.83 | 1.24 | 1.99 | 2.13 | NaN | 0.84 | NaN | NaN |
| Q6KC79 | Nipped-B-like protein | NIPBL | 2658 | S | 1 | 165.6 | 0.74 | 0.72 | 0.75 | 0.74 | 0.86 | 0.81 | 0.85 | 0.94 | 0.84 |
| Q6KC79 | Nipped-B-like protein | NIPBL | 318 | S | 1 | 59.59 | 0.85 | 0.96 | NaN | 0.86 | 0.80 | NaN | 0.82 | 0.73 | NaN |
| Q6NZI2 | Polymerase I and transcript release factor | PTRF | 202 | S | 1 | 183.7 | 1.10 | 1.07 | 1.08 | NaN | 0.98 | 1.04 | 1.19 | NaN | NaN |
| Q6NZI2 | Polymerase I and transcript release factor | PTRF | 203 | S | 1 | 183.7 | 1.10 | 1.07 | 1.08 | NaN | 0.98 | 1.04 | 1.19 | NaN | NaN |
| Q6NZI2 | Polymerase I and transcript release factor | PTRF | 365 | S | 1 | 200 | 1.38 | 1.31 | 1.18 | 0.95 | 0.83 | 1.04 | 1.00 | 0.86 | 0.96 |
| Q6NZI2 | Polymerase I and transcript release factor | PTRF | 366 | S | 1 | 200 | 1.38 | 1.35 | 1.18 | 0.95 | 1.00 | 1.04 | 1.00 | 1.03 | 0.96 |
| Q6NZI2 | Polymerase I and transcript release factor | PTRF | 379 | S | 0.991 | 173 | 0.76 | 0.69 | NaN | 0.58 | NaN | NaN | 0.74 | 0.72 | NaN |
| Q6NZI2 | Polymerase I and transcript release factor | PTRF | 387 | S | 1 | 200 | 0.63 | 0.55 | 0.60 | 0.61 | 0.57 | 0.68 | 0.67 | 0.87 | 1.05 |
| Q6NZI2 | Polymerase I and transcript release factor | PTRF | 389 | S | 1 | 200 | 0.63 | 0.55 | 0.60 | 0.61 | 0.57 | 0.68 | 0.67 | 0.87 | 1.05 |
| Q6P2E9 | Enhancer of mRNA-decapping protein 4 | EDC4 | 879 | S | 0.998 | 161 | 1.47 | 1.67 | 1.01 | 1.02 | NaN | 0.86 | 1.45 | NaN | NaN |
| Q6P2E9 | Enhancer of mRNA-decapping protein 4 | EDC4 | 723 | S | 0.981 | 139 | 1.49 | NaN | 0.85 | NaN | 1.93 | 1.55 | 1.19 | 1.40 | 1.51 |
| Q6P597 | Kinesin light chain 3 | KLC3 | 502 | S | 0.994 | 94.51 | 1.04 | 0.91 | NaN | NaN | NaN | 1.04 | NaN | 1.07 | NaN |

| | | | | | | | | | | | | | | |
|---|---|---|---|---|---|---|---|---|---|---|---|---|---|---|
| Q6PD62 | RNA polymerase-associated protein CTR9 homolog | CTR9 | 970 | S | 1 | 313.9 | 0.62 | 0.57 | 0.63 | 0.58 | 0.62 | NaN | 0.69 | 0.91 | 0.74 |
| Q6PKG0 | La-related protein 1 | LARP1 | 90 | S | 1 | 99.79 | NaN | NaN | 1.09 | NaN | 0.92 | NaN | NaN | 1.05 | 0.52 |
| Q6PKG0 | La-related protein 1 | LARP1 | 521 | S | 1 | 157 | 1.18 | 1.14 | 1.12 | 1.08 | 1.11 | 1.09 | 1.04 | 1.03 | 1.09 |
| Q6PKG0 | La-related protein 1 | LARP1 | 546 | S | 0.5 | 209.6 | 3.14 | 3.44 | NaN | NaN | NaN | 2.28 | 2.55 | NaN | NaN |
| Q6PKG0 | La-related protein 1 | LARP1 | 548 | S | 1 | 276 | 3.36 | 3.44 | NaN | 3.76 | 3.04 | 2.28 | 2.55 | NaN | NaN |
| Q6PKG0 | La-related protein 1 | LARP1 | 627 | S | 0.983 | 78.92 | NaN | NaN | NaN | NaN | NaN | NaN | 2.14 | 2.38 | NaN |
| Q6PKG0 | La-related protein 1 | LARP1 | 143 | S | 0.99 | 90.3 | 0.87 | NaN | 1.01 | 0.93 | NaN | 0.93 | 0.81 | 0.79 | 0.86 |
| Q6PKG0 | La-related protein 1 | LARP1 | 774 | S | 1 | 109.1 | NaN | NaN | 2.66 | 2.32 | 2.39 | 2.48 | 2.23 | 2.17 | 2.48 |
| Q6PKG0 | La-related protein 1 | LARP1 | 851 | S | 0.744 | 162.8 | NaN | NaN | NaN | 0.93 | 0.79 | NaN | NaN | NaN | NaN |
| Q6QNY0 | Biogenesis of lysosome-related organelles complex 1 | BLOC1S3 | 65 | S | 1 | 128.9 | 1.15 | 1.16 | 1.25 | 1.09 | 1.11 | NaN | 1.09 | 1.11 | 1.16 |
| Q6SPF0 | Atherin | SAMD1 | 161 | S | 0.993 | 67.36 | NaN | 0.75 | 1.66 | 1.55 | 1.17 | NaN | 2.09 | 2.13 | NaN |
| Q6UN15 | Pre-mRNA 3'-end-processing factor FIP1 | FIP1L1 | 259 | S | 0.976 | 108.7 | 1.29 | 1.31 | 1.21 | 1.96 | 2.09 | 1.88 | NaN | NaN | 0.91 |
| Q6UN15 | Pre-mRNA 3'-end-processing factor FIP1 | FIP1L1 | 304 | S | 1 | 75.04 | NaN | NaN | NaN | NaN | NaN | NaN | 3.28 | NaN | 3.83 |
| Q6UN15 | Pre-mRNA 3'-end-processing factor FIP1 | FIP1L1 | 492 | S | 1 | 128 | 0.89 | 0.94 | 0.93 | 0.92 | 0.86 | 0.90 | 1.03 | 1.01 | 0.96 |
| Q6UN15 | Pre-mRNA 3'-end-processing factor FIP1 | FIP1L1 | 500 | S | 0.999 | 128 | 1.21 | 1.10 | 1.49 | 0.85 | 1.00 | 1.24 | 1.12 | 1.06 | 1.03 |
| Q6VMQ6 | Activating transcription factor 7-interacting protein 1 | ATF7IP | 557 | S | 0.5 | 41.3 | NaN | NaN | NaN | NaN | 0.96 | 1.12 | 0.91 | 0.88 | NaN |
| Q6VMQ6 | Activating transcription factor 7-interacting protein 1 | ATF7IP | 559 | S | 0.976 | 67.1 | 0.43 | NaN | NaN | 0.81 | 0.85 | 1.12 | 0.91 | 0.75 | 0.79 |
| Q6VY07 | Phosphofurin acidic cluster sorting protein 1 | PACS1 | 495 | S | 0.917 | 112.6 | 2.53 | 2.16 | 1.71 | NaN | NaN | NaN | 1.38 | NaN | 1.50 |
| Q6VY07 | Phosphofurin acidic cluster sorting protein 1 | PACS1 | 529 | S | 0.778 | 106.4 | NaN | NaN | 1.21 | 1.07 | 1.27 | 0.95 | 2.12 | NaN | NaN |
| Q6VY07 | Phosphofurin acidic cluster sorting protein 1 | PACS1 | 534 | S | 0.95 | 106.4 | 1.01 | 0.43 | 0.74 | 1.07 | 1.27 | 0.95 | 1.13 | NaN | 1.73 |
| Q6ZRP7 | Sulfhydryl oxidase 2 | QSOX2 | 578 | S | 0.854 | 342.2 | 1.01 | 0.95 | 0.78 | 0.88 | 0.78 | 0.87 | 0.99 | 0.80 | 1.06 |
| Q6ZTN6 | Ankyrin repeat domain-containing protein 13D | ANKRD13 D | 465 | S | 1 | 66.08 | 0.81 | 0.71 | NaN | 1.00 | 1.13 | 0.81 | 0.94 | 1.20 | 0.91 |
| Q6ZUT6 | Uncharacterized protein C15orf52 | C15orf52 | 201 | S | 0.943 | 99.81 | NaN | NaN | 0.81 | 0.97 | 0.78 | 0.91 | 0.94 | 1.01 | 1.08 |
| Q71RC2 | La-related protein 4 | LARP4 | 597 | S | 0.718 | 134.5 | 1.72 | 1.93 | NaN | NaN | NaN | NaN | NaN | NaN | NaN |
| Q71RC2 | La-related protein 4 | LARP4 | 583 | S | 0.979 | 158.7 | 1.23 | 1.28 | 1.28 | 1.44 | 1.44 | 1.50 | 0.82 | 0.75 | 0.82 |
| Q71RC2 | La-related protein 4 | LARP4 | 722 | S | 1 | 81.24 | 2.13 | 1.94 | NaN | NaN | 1.70 | 1.43 | 0.93 | 1.25 | 1.06 |
| Q7KZ85 | Transcription elongation factor SPT6 | SUPT6H | 125 | S | 1 | 106 | 0.59 | NaN | 0.53 | 0.87 | 0.88 | 0.91 | 0.47 | NaN | 0.72 |
| Q7L7X3 | Serine/threonine-protein kinase TAO1 | TAOK1 | 421 | S | 0.995 | 84.09 | NaN | 0.97 | NaN | NaN | NaN | NaN | 1.25 | 1.38 | NaN |
| Q7L8J4 | SH3 domain-binding protein 5-like | SH3BP5L | 30 | S | 1 | 173 | 1.03 | 1.16 | 1.49 | 1.11 | NaN | 1.21 | 1.26 | 1.52 | NaN |
| Q7LBC6 | Lysine-specific demethylase | KDM3B | 744 | S | 0.964 | 230.4 | NaN | 0.81 | 0.65 | 0.87 | 0.99 | 0.79 | 0.78 | NaN | 0.80 |

| | | | | | | | | | | | | | | | | |
|---|---|---|---|---|---|---|---|---|---|---|---|---|---|---|---|---|
| Q7RTP6 | Protein-methionine sulfoxide oxidase MICAL3 | MICAL3 | 1649 | S | 0.867 | 43.86 | NaN | NaN | NaN | NaN | NaN | NaN | 1.31 | 1.23 | NaN |
| Q7Z333 | Probable helicase senataxin | SETX | 1017 | S | 1 | 127.3 | NaN | NaN | NaN | NaN | NaN | NaN | 1.12 | 0.95 | NaN |
| Q7Z333 | Probable helicase senataxin | SETX | 1019 | S | 1 | 127.3 | NaN | NaN | NaN | NaN | NaN | NaN | 1.12 | 0.95 | NaN |
| Q7Z3C6 | Autophagy-related protein 9A | ATG9A | 18 | S | 0.973 | 117.3 | 0.82 | 1.01 | NaN | 0.83 | NaN | NaN | 0.97 | 0.69 | 1.04 |
| Q7Z3K3 | Pogo transposable element with ZNF domain | POGZ | 1338 | S | 0.838 | 63.3 | NaN | 0.73 | 1.37 | NaN | NaN | 1.07 | NaN | NaN | NaN |
| Q7Z3K3 | Pogo transposable element with ZNF domain | POGZ | 425 | S | 0.969 | 112.3 | 0.62 | 0.48 | 0.50 | 0.72 | 0.67 | 0.65 | 0.83 | 0.76 | 0.79 |
| Q7Z417 | Nuclear fragile X mental retardation-interacting protein | NUFIP2 | 629 | S | 1 | 276.3 | 2.91 | 2.88 | 3.05 | 2.52 | NaN | 2.66 | 1.10 | 1.18 | 1.12 |
| Q7Z417 | Nuclear fragile X mental retardation-interacting protein | NUFIP2 | 212 | S | 1 | 253.3 | 2.51 | 3.41 | 3.11 | 2.38 | 2.14 | 2.14 | 1.69 | 1.74 | 1.64 |
| Q7Z417 | Nuclear fragile X mental retardation-interacting protein | NUFIP2 | 652 | S | 1 | 73.88 | 2.39 | 2.73 | 2.83 | 2.18 | 2.13 | NaN | 0.95 | 0.99 | NaN |
| Q7Z417 | Nuclear fragile X mental retardation-interacting protein | NUFIP2 | 572 | S | 0.98 | 90.86 | 1.73 | 1.84 | 1.90 | 2.05 | 1.94 | 1.82 | 0.90 | 1.17 | NaN |
| Q7Z422 | SUZ domain-containing protein 1 | SZRD1 | 107 | S | 0.997 | 95.52 | 0.66 | 0.63 | 0.67 | 0.70 | 0.70 | 0.72 | 0.89 | 0.81 | 0.82 |
| Q7Z422 | SUZ domain-containing protein 1 | SZRD1 | 39 | S | 0.997 | 175 | NaN | 0.67 | NaN | 0.80 | 0.72 | NaN | 0.83 | 0.74 | NaN |
| Q7Z434 | Mitochondrial antiviral-signaling protein | MAVS | 152 | S | 0.988 | 124.5 | 1.37 | 0.94 | 1.88 | 2.10 | 1.91 | 1.98 | 0.51 | NaN | 0.79 |
| Q7Z434 | Mitochondrial antiviral-signaling protein | MAVS | 165 | S | 0.989 | 156.9 | 1.47 | 1.08 | 1.33 | 1.51 | 1.44 | 1.43 | 0.66 | 0.69 | 0.77 |
| Q7Z434 | Mitochondrial antiviral-signaling protein | MAVS | 222 | S | 0.981 | 106.7 | 2.71 | 2.55 | 2.26 | NaN | 3.00 | NaN | 1.63 | NaN | 1.50 |
| Q7Z460 | CLIP-associating protein 1 | CLASP1 | 1091 | S | 0.99 | 120.5 | NaN | 1.41 | 1.51 | 1.31 | 1.77 | 1.63 | 1.19 | 1.26 | 1.65 |
| Q7Z4V5 | Hepatoma-derived growth factor-related protein 2 | HDGFRP 2 | 366 | S | 1 | 99.45 | 0.89 | 1.01 | 1.00 | 0.96 | 0.98 | 0.95 | 1.06 | 0.98 | 1.01 |
| Q7Z4V5 | Hepatoma-derived growth factor-related protein 2 | HDGFRP 2 | 369 | S | 1 | 99.45 | 0.89 | 1.01 | 1.00 | 0.96 | 0.98 | 0.95 | 1.06 | 0.98 | 1.01 |
| Q7Z4V5 | Hepatoma-derived growth factor-related protein 2 | HDGFRP 2 | 370 | S | 1 | 99.45 | 0.89 | 1.01 | 1.00 | 0.96 | 0.98 | 0.95 | 1.06 | 0.98 | 1.01 |
| Q7Z4V5 | Hepatoma-derived growth factor-related protein 2 | HDGFRP 2 | 664 | S | 1 | 113.8 | 1.57 | 1.54 | 1.53 | 1.43 | 1.45 | 1.66 | 1.57 | 1.85 | 1.53 |
| Q7Z4V5 | Hepatoma-derived growth factor-related protein 2 | HDGFRP 2 | 652 | S | 1 | 67.4 | 1.17 | NaN | NaN | 1.33 | 1.15 | NaN | NaN | NaN | NaN |
| Q7Z4V5 | Hepatoma-derived growth factor-related protein 2 | HDGFRP 2 | 230 | S | 0.999 | 54.31 | NaN | NaN | 0.45 | 0.66 | 0.67 | 0.64 | NaN | 0.44 | 0.58 |
| Q7Z4V5 | Hepatoma-derived growth factor-related protein 2 | HDGFRP 2 | 232 | S | 0.996 | 72.14 | NaN | 0.46 | 0.47 | 0.66 | 0.67 | 0.59 | NaN | 0.44 | 0.58 |
| Q7Z4V5 | Hepatoma-derived growth factor-related protein 2 | HDGFRP 2 | 234 | S | 0.982 | 72.14 | NaN | 0.46 | 0.47 | 0.66 | 0.64 | 0.59 | NaN | 0.43 | 0.58 |
| Q7Z4V5 | Hepatoma-derived growth factor-related protein 2 | HDGFRP 2 | 240 | S | 0.989 | 72.14 | NaN | 0.46 | 0.47 | 0.66 | 0.67 | 0.59 | NaN | 0.43 | 0.58 |

| | | | | | | | | | | | | | | | |
|---|---|---|---|---|---|---|---|---|---|---|---|---|---|---|---|
| Q7Z5K2 | Wings apart-like protein homolog | WAPAL | 221 | S | 0.996 | 91.66 | 0.80 | 1.02 | 0.77 | 0.66 | NaN | 1.37 | 1.14 | 0.90 | 1.13 |
| Q7Z5K2 | Wings apart-like protein homolog | WAPAL | 223 | S | 0.957 | 77.29 | NaN | NaN | NaN | NaN | 0.69 | 0.72 | NaN | NaN | NaN |
| Q7Z5K2 | Wings apart-like protein homolog | WAPAL | 77 | S | 0.999 | 164.4 | 1.02 | 1.07 | 1.04 | 0.86 | 0.93 | 1.05 | 0.93 | 1.35 | 0.98 |
| Q7Z5L9 | Interferon regulatory factor 2-binding protein 2 | IRF2BP2 | 360 | S | 1 | 143.6 | 0.52 | 0.49 | 0.58 | 0.50 | 0.42 | 0.50 | 0.83 | 0.94 | 0.96 |
| Q7Z5L9 | Interferon regulatory factor 2-binding protein 2 | IRF2BP2 | 175 | S | 1 | 129.8 | NaN | 1.38 | NaN | NaN | 0.83 | NaN | 2.33 | 2.26 | 2.69 |
| Q7Z5L9 | Interferon regulatory factor 2-binding protein 2 | IRF2BP2 | 457 | S | 0.969 | 77.36 | 0.49 | 0.47 | NaN | NaN | NaN | 0.40 | 0.56 | 0.64 | 0.80 |
| Q7Z5L9 | Interferon regulatory factor 2-binding protein 2 | IRF2BP2 | 460 | S | 0.959 | 109.1 | 0.49 | 0.47 | NaN | 0.40 | NaN | 0.40 | 0.59 | 0.59 | NaN |
| Q7Z6Z7 | E3 ubiquitin-protein ligase HUWE1 | HUWE1 | 3373 | S | 0.983 | 163.6 | 1.00 | 0.97 | 0.89 | 1.43 | NaN | 1.41 | 1.34 | NaN | 1.34 |
| Q7Z6Z7 | E3 ubiquitin-protein ligase HUWE1 | HUWE1 | 1395 | S | 1 | 106.4 | NaN | NaN | 1.63 | 1.47 | 1.40 | 1.46 | 1.37 | 1.36 | 1.39 |
| Q7Z6Z7 | E3 ubiquitin-protein ligase HUWE1 | HUWE1 | 2887 | S | 0.872 | 138.3 | NaN | NaN | NaN | 1.25 | 1.60 | NaN | NaN | 0.71 | NaN |
| Q7Z6Z7 | E3 ubiquitin-protein ligase HUWE1 | HUWE1 | 3662 | S | 0.991 | 93.23 | 2.36 | NaN | 2.00 | 2.60 | 2.76 | 2.21 | 0.98 | 1.00 | 0.89 |
| Q7Z6Z7 | E3 ubiquitin-protein ligase HUWE1 | HUWE1 | 3919 | S | 0.999 | 90.66 | 4.90 | 5.39 | 3.39 | 3.85 | NaN | 3.60 | 1.76 | NaN | NaN |
| Q7Z6Z7 | E3 ubiquitin-protein ligase HUWE1 | HUWE1 | 2362 | S | 1 | 80.47 | 0.38 | 0.44 | NaN | NaN | NaN | NaN | NaN | NaN | NaN |
| Q7Z6Z7 | E3 ubiquitin-protein ligase HUWE1 | HUWE1 | 1907 | S | 1 | 216.5 | 1.12 | 1.11 | 1.17 | 1.05 | 1.03 | 1.07 | 1.14 | 1.08 | 1.11 |
| Q7Z6Z7 | E3 ubiquitin-protein ligase HUWE1 | HUWE1 | 3816 | S | 0.997 | 153.9 | 0.81 | 0.76 | 0.73 | 1.16 | 1.04 | 1.04 | 0.56 | NaN | 0.54 |
| Q7Z6Z7 | E3 ubiquitin-protein ligase HUWE1 | HUWE1 | 2918 | S | 0.915 | 120.3 | NaN | 1.03 | 1.03 | NaN | 1.35 | 1.40 | 1.02 | 1.08 | 1.23 |
| Q86TB9 | Protein PAT1 homolog 1 | PATL1 | 179 | S | 0.995 | 97.46 | 1.01 | 1.37 | NaN | NaN | NaN | 0.90 | NaN | 0.98 | 1.12 |
| Q86TB9 | Protein PAT1 homolog 1 | PATL1 | 184 | S | 1 | 88.28 | 1.29 | 1.37 | NaN | NaN | NaN | NaN | NaN | 1.37 | NaN |
| Q86U06 | Probable RNA-binding protein 23 | RBM23 | 149 | S | 1 | 73.57 | 0.84 | NaN | 0.93 | 1.03 | 1.02 | 0.89 | 0.83 | 0.66 | NaN |
| Q86U42 | Polyadenylate-binding protein | PABPN1 | 95 | S | 0.955 | 73.24 | 1.52 | 1.26 | 1.38 | 1.20 | 1.05 | 1.43 | 1.57 | NaN | 1.10 |
| Q86U42 | Polyadenylate-binding protein | PABPN1 | 150 | S | 1 | 91.66 | 0.70 | 0.81 | 0.76 | 1.31 | 1.75 | 1.17 | NaN | 0.36 | NaN |
| Q86U86 | Protein polybromo-1 | PBRM1 | 10 | S | 0.976 | 129.9 | NaN | 0.74 | 0.66 | 0.84 | 0.95 | NaN | 1.01 | 0.92 | NaN |
| Q86U90 | YrdC domain-containing protein, mitochondrial | YRDC | 37 | S | 1 | 60.62 | NaN | 1.13 | NaN | 1.04 | 1.00 | NaN | NaN | 1.11 | 0.96 |
| Q86UP2 | Kinectin | KTN1 | 75 | S | 0.994 | 97.93 | 2.66 | 1.94 | NaN | NaN | 1.92 | NaN | NaN | 2.16 | NaN |
| Q86UU0 | B-cell CLL/lymphoma 9-like protein | BCL9L | 21 | S | 1 | 100.2 | 2.49 | NaN | 2.64 | NaN | NaN | 0.65 | NaN | 0.97 | 1.11 |
| Q86UU0 | B-cell CLL/lymphoma 9-like protein | BCL9L | 25 | S | 1 | 64.22 | 2.49 | NaN | 2.64 | NaN | NaN | NaN | NaN | NaN | NaN |

| | | | | | | | | | | | | | | |
|---|---|---|---|---|---|---|---|---|---|---|---|---|---|---|
| Q86UU1 | Pleckstrin homology-like domain family B member 1 | PHLDB1 | 518 | S | 0.838 | 97.35 | 3.37 | 2.96 | NaN | NaN | NaN | NaN | NaN | NaN | NaN |
| Q86UX6 | Serine/threonine-protein kinase 32C | STK32C | 18 | S | 0.979 | 112.3 | 0.59 | 0.72 | NaN | 0.69 | 0.70 | NaN | 0.78 | 0.94 | NaN |
| Q86V15 | Zinc finger protein castor homolog 1 | CASZ1 | 1758 | S | 0.959 | 60.1 | 0.28 | NaN | NaN | NaN | NaN | NaN | NaN | 0.43 | 0.84 |
| Q86V48 | Leucine zipper protein 1 | LUZP1 | 659 | S | 1 | 179.2 | 1.84 | 1.60 | 2.05 | 1.17 | 1.27 | 1.25 | 1.18 | 1.42 | 1.56 |
| Q86V48 | Leucine zipper protein 1 | LUZP1 | 394 | S | 1 | 63.18 | 1.61 | 1.34 | 1.52 | 1.08 | 1.16 | 1.07 | 1.39 | 1.66 | 1.37 |
| Q86VQ1 | Glucocorticoid-induced transcript 1 protein | GLCCI1 | 105 | S | 0.983 | 147.5 | NaN | NaN | 0.45 | 0.57 | 0.55 | NaN | 0.82 | 0.87 | NaN |
| Q86VQ1 | Glucocorticoid-induced transcript 1 protein | GLCCI1 | 107 | S | 0.693 | 71.42 | NaN | NaN | NaN | NaN | 0.55 | NaN | 0.82 | 0.87 | NaN |
| Q86VQ1 | Glucocorticoid-induced transcript 1 protein | GLCCI1 | 223 | S | 1 | 86.29 | 0.66 | 0.46 | NaN | NaN | NaN | NaN | NaN | NaN | NaN |
| Q86VQ1 | Glucocorticoid-induced transcript 1 protein | GLCCI1 | 303 | S | 1 | 56.77 | NaN | NaN | NaN | 0.62 | NaN | NaN | 0.70 | 0.74 | 0.70 |
| Q86W56 | Poly(ADP-ribose) glycohydrolase | PARG | 130 | S | 0.776 | 66.64 | NaN | NaN | 1.14 | NaN | 1.21 | 1.03 | NaN | 1.03 | NaN |
| Q86W56 | Poly(ADP-ribose) glycohydrolase | PARG | 137 | S | 0.704 | 57.77 | 0.94 | 1.01 | NaN | NaN | NaN | NaN | 1.05 | NaN | 1.17 |
| Q86W56 | Poly(ADP-ribose) glycohydrolase | PARG | 448 | S | 1 | 74.79 | NaN | 0.75 | NaN | 0.99 | 0.89 | NaN | NaN | NaN | NaN |
| Q86W92 | Liprin-beta-1 | PPFIBP1 | 466 | S | 0.938 | 51.77 | 1.81 | 1.87 | NaN | NaN | NaN | NaN | NaN | NaN | NaN |
| Q86WR7 | Proline and serine-rich protein 2 | PROSER 2 | 179 | S | 1 | 99.5 | 1.89 | 2.10 | 2.01 | 0.92 | 0.94 | 0.91 | 1.97 | 2.03 | 1.97 |
| Q86WR7 | Proline and serine-rich protein 2 | PROSER 2 | 212 | S | 0.907 | 94.13 | NaN | 4.02 | 4.54 | NaN | 2.82 | NaN | NaN | NaN | NaN |
| Q86WR7 | Proline and serine-rich protein 2 | PROSER 2 | 43 | S | 0.959 | 86.11 | NaN | 5.29 | 3.40 | NaN | NaN | NaN | 3.73 | NaN | NaN |
| Q86X95 | Corepressor interacting with RBPJ 1 | CIR1 | 202 | S | 1 | 204.9 | 1.33 | 1.50 | 0.83 | 0.99 | 0.92 | 0.80 | 1.13 | 0.95 | 0.98 |
| Q86XP3 | ATP-dependent RNA helicase DDX42 | DDX42 | 109 | S | 1 | 272.3 | 0.77 | 0.87 | 0.86 | 0.99 | 1.10 | NaN | NaN | NaN | NaN |
| Q86XP3 | ATP-dependent RNA helicase DDX42 | DDX42 | 111 | S | 1 | 272.3 | 0.77 | 0.87 | 0.86 | 0.99 | 1.10 | NaN | NaN | NaN | NaN |
| Q86XP3 | ATP-dependent RNA helicase DDX42 | DDX42 | 185 | S | 1 | 341.6 | 0.64 | 0.68 | 0.71 | 0.80 | 0.88 | 0.76 | 0.60 | 0.59 | 0.65 |
| Q86YD1 | Prostate tumor-overexpressed gene 1 protein | PTOV1 | 34 | S | 0.96 | 78.15 | 0.98 | 1.29 | NaN | NaN | NaN | 1.03 | NaN | NaN | NaN |
| Q86YP4 | Transcriptional repressor p66-alpha | GATAD2A | 100 | S | 1 | 123.4 | 0.70 | 0.64 | 1.30 | 0.74 | 1.13 | NaN | 0.77 | 0.76 | 1.26 |
| Q86YP4 | Transcriptional repressor p66-alpha | GATAD2A | 107 | S | 1 | 113.8 | NaN | 1.17 | 1.17 | 1.12 | 1.13 | NaN | 1.19 | 1.21 | 1.26 |
| Q86YP4 | Transcriptional repressor p66-alpha | GATAD2A | 113 | S | 0.668 | 78.66 | NaN | 0.55 | 1.17 | NaN | NaN | NaN | NaN | NaN | NaN |
| Q86YP4 | Transcriptional repressor p66-alpha | GATAD2A | 114 | S | 0.952 | 123.4 | NaN | 1.17 | NaN | 0.74 | 1.13 | NaN | 1.19 | 0.74 | 1.26 |

| | | | | | | | | | | | | | | | |
|---|---|---|---|---|---|---|---|---|---|---|---|---|---|---|---|
| Q86YS7 | C2 domain-containing protein | C2CD5 | 295 | S | 0.851 | 49.36 | NaN | NaN | NaN | 0.94 | 0.65 | NaN | NaN | NaN | NaN |
| Q86YV5 | Tyrosine-protein kinase SgK223 | SGK223 | 263 | S | 1 | 95.11 | NaN | NaN | NaN | 0.88 | NaN | 0.64 | 0.62 | NaN | NaN |
| Q86YV5 | Tyrosine-protein kinase SgK223 | SGK223 | 148 | S | 0.874 | 112.9 | 0.54 | 0.81 | NaN | 1.21 | 1.05 | 1.11 | NaN | NaN | 0.45 |
| Q8IU81 | Interferon regulatory factor 2-binding protein 1 | IRF2BP1 | 384 | S | 1 | 105.2 | 0.84 | 0.59 | 0.84 | 0.86 | 0.84 | 0.83 | 0.91 | 0.94 | 0.91 |
| Q8IVF2 | Protein AHNAK2 | AHNAK2 | 294 | S | 0.883 | 125.1 | NaN | 1.40 | 1.30 | 2.21 | NaN | NaN | 1.51 | 1.71 | 3.12 |
| Q8IVF2 | Protein AHNAK2 | AHNAK2 | 5175 | S | 1 | 96.02 | NaN | 0.53 | NaN | 0.43 | 0.63 | NaN | 0.80 | 0.83 | NaN |
| Q8IWE2 | Protein NOXP20 | FAM114A1 | 196 | S | 0.972 | 76.2 | 1.09 | NaN | 1.25 | NaN | NaN | NaN | 1.98 | NaN | NaN |
| Q8IWE2 | Protein NOXP20 | FAM114A1 | 120 | S | 0.991 | 94.8 | 0.80 | NaN | NaN | 1.16 | 1.18 | NaN | NaN | NaN | NaN |
| Q8IWZ3 | Ankyrin repeat and KH domain-containing protein 1 | ANKHD1 | 93 | S | 0.925 | 154.4 | 0.83 | 0.70 | NaN | 0.68 | 0.86 | 1.13 | 0.87 | 0.71 | 0.59 |
| Q8IWZ3 | Ankyrin repeat and KH domain-containing protein 1 | ANKHD1 | 95 | S | 0.994 | 154.4 | 0.83 | 0.70 | NaN | 0.68 | 0.81 | 1.13 | 0.87 | 0.71 | 0.59 |
| Q8IY81 | pre-rRNA processing protein FTSJ3 | FTSJ3 | 335 | S | 1 | 219.2 | 1.15 | 1.12 | 1.14 | NaN | 0.66 | 0.92 | 1.30 | 1.04 | 1.15 |
| Q8IY81 | pre-rRNA processing protein FTSJ3 | FTSJ3 | 336 | S | 1 | 219.2 | 1.15 | 1.12 | 1.14 | NaN | 0.66 | 0.92 | 1.30 | 1.04 | 1.15 |
| Q8N1G2 | Cap-specific mRNA (nucleoside-2'-O-)-methyltransferase 1 | CMTR1 | 49 | S | 0.895 | 100.6 | NaN | NaN | 0.55 | 0.61 | NaN | 0.52 | 0.43 | 0.50 | 0.68 |
| Q8N1G2 | Cap-specific mRNA (nucleoside-2'-O-)-methyltransferase 1 | CMTR1 | 51 | S | 0.988 | 133.3 | NaN | 0.98 | 0.59 | 0.38 | NaN | 0.69 | 0.75 | 0.50 | 0.88 |
| Q8N1G2 | Cap-specific mRNA (nucleoside-2'-O-)-methyltransferase 1 | CMTR1 | 53 | S | 0.981 | 192.4 | 0.32 | 0.98 | 0.45 | 0.34 | NaN | 0.39 | 0.75 | 0.56 | 0.55 |
| Q8N1G4 | Leucine-rich repeat-containing protein 47 | LRRC47 | 520 | S | 0.942 | 151.7 | 0.83 | 0.86 | 0.68 | 0.89 | NaN | 0.89 | 1.06 | NaN | 1.13 |
| Q8N392 | Rho GTPase-activating protein 18 | ARHGAP18 | 64 | S | 0.692 | 145.2 | NaN | NaN | NaN | NaN | NaN | NaN | NaN | 1.08 | 1.57 |
| Q8N392 | Rho GTPase-activating protein 18 | ARHGAP18 | 66 | S | 0.761 | 145.2 | 1.87 | 2.14 | NaN | 2.36 | NaN | NaN | 1.38 | 1.08 | 1.57 |
| Q8N392 | Rho GTPase-activating protein 18 | ARHGAP18 | 69 | S | 0.986 | 133.4 | 1.87 | 2.14 | NaN | 2.36 | NaN | NaN | 1.38 | 1.08 | 1.57 |
| Q8N3D4 | EH domain-binding protein 1-like protein 1 | EHBP1L1 | 285 | S | 0.5 | 152.3 | NaN | NaN | 0.98 | 1.15 | 1.13 | 1.03 | 0.96 | 0.83 | 0.82 |
| Q8N3D4 | EH domain-binding protein 1-like protein 1 | EHBP1L1 | 964 | S | 1 | 65.8 | NaN | 0.75 | NaN | 1.14 | 1.04 | 0.99 | 1.00 | 0.89 | 0.96 |
| Q8N3F8 | MICAL-like protein 1 | MICALL1 | 621 | S | 0.908 | 77.63 | NaN | 1.23 | NaN | 0.86 | 1.16 | NaN | 1.27 | 1.06 | 1.28 |
| Q8N3V7 | Synaptopodin | SYNPO | 833 | S | 0.998 | 106.4 | 0.98 | 0.96 | NaN | 0.78 | 1.08 | NaN | 1.45 | 1.32 | NaN |
| Q8N3V7 | Synaptopodin | SYNPO | 685 | S | 1 | 65.18 | 0.95 | 0.86 | NaN | 0.70 | NaN | NaN | 1.30 | 1.53 | NaN |
| Q8N3X1 | Formin-binding protein 4 | FNBP4 | 116 | S | 0.927 | 51.46 | NaN | NaN | NaN | 0.98 | 0.98 | NaN | 1.35 | 1.28 | NaN |
| Q8N3X1 | Formin-binding protein 4 | FNBP4 | 18 | S | 1 | 54.86 | 0.99 | NaN | 1.02 | NaN | 1.11 | 1.12 | 1.13 | 1.15 | 0.98 |

| | | | | | | | | | | | | | | | |
|---|---|---|---|---|---|---|---|---|---|---|---|---|---|---|---|
| Q8N4X5 | Actin filament-associated protein 1-like 2 | AFAP1L2 | 640 | S | 0.999 | 66.91 | NaN | NaN | NaN | 0.59 | NaN | NaN | 1.30 | 1.31 | 1.79 |
| Q8N4X5 | Actin filament-associated protein 1-like 2 | AFAP1L2 | 414 | S | 0.62 | 107.2 | 0.43 | NaN | NaN | NaN | 0.64 | NaN | 1.87 | 1.26 | NaN |
| Q8N556 | Actin filament-associated protein 1 | AFAP1 | 264 | S | 0.594 | 167.1 | NaN | NaN | 1.42 | NaN | 1.36 | 1.59 | 0.87 | NaN | 0.96 |
| Q8N556 | Actin filament-associated protein 1 | AFAP1 | 265 | S | 0.876 | 167.1 | 1.35 | 1.43 | NaN | 1.34 | 1.36 | NaN | NaN | 0.96 | NaN |
| Q8N556 | Actin filament-associated protein 1 | AFAP1 | 665 | S | 0.978 | 101.4 | 0.20 | 1.50 | 1.12 | 1.38 | NaN | NaN | 1.11 | NaN | NaN |
| Q8N556 | Actin filament-associated protein 1 | AFAP1 | 668 | S | 1 | 133 | 1.47 | 1.93 | 2.05 | 1.38 | 1.48 | 1.47 | 1.79 | NaN | 1.65 |
| Q8N556 | Actin filament-associated protein 1 | AFAP1 | 679 | S | 0.919 | 111.4 | NaN | NaN | NaN | 0.47 | 0.43 | NaN | NaN | NaN | NaN |
| Q8N6N3 | UPF0690 protein C1orf52 | C1orf52 | 158 | S | 1 | 261.5 | 1.22 | 1.26 | 1.18 | 1.14 | 1.16 | 1.14 | 1.00 | 0.89 | 0.97 |
| Q8N8A6 | ATP-dependent RNA helicase DDX51 | DDX51 | 83 | S | 1 | 125.4 | 0.60 | 0.63 | 0.71 | 0.74 | 0.64 | 0.61 | 0.85 | 0.84 | 0.93 |
| Q8N9M5 | Transmembrane protein 102 | TMEM102 | 246 | S | 1 | 52.73 | NaN | NaN | 2.37 | 1.92 | 2.04 | NaN | 1.12 | 1.25 | 1.74 |
| Q8NAV1 | Pre-mRNA-splicing factor 38A | PRPF38A | 209 | S | 1 | 78.15 | 1.00 | 1.00 | NaN | NaN | 0.96 | NaN | NaN | 1.12 | NaN |
| Q8NBN3 | Transmembrane protein 87A | TMEM87 A | 540 | S | 1 | 145.1 | 1.37 | 1.62 | 1.11 | 1.17 | 1.38 | 0.87 | 1.11 | 0.84 | 1.28 |
| Q8NC44 | Protein FAM134A | FAM134A | 281 | S | 0.966 | 118.3 | NaN | 0.99 | 0.95 | NaN | 2.01 | 1.01 | 1.32 | NaN | 1.65 |
| Q8NC44 | Protein FAM134A | FAM134A | 283 | S | 0.769 | 118.3 | NaN | NaN | 0.95 | NaN | NaN | NaN | NaN | 1.34 | 1.65 |
| Q8NC44 | Protein FAM134A | FAM134A | 385 | S | 1 | 266.4 | 1.20 | 1.15 | 1.15 | 1.28 | 2.06 | 1.23 | 1.18 | 1.18 | 1.17 |
| Q8NC56 | LEM domain-containing protein 2 | LEMD2 | 134 | S | 1 | 81.64 | NaN | 2.20 | NaN | NaN | 1.86 | 10.75 | NaN | 2.41 | NaN |
| Q8NC56 | LEM domain-containing protein 2 | LEMD2 | 138 | S | 1 | 131.1 | 0.81 | 0.83 | 0.87 | 0.81 | 0.90 | 0.84 | 0.64 | 0.68 | 0.71 |
| Q8NC56 | LEM domain-containing protein 2 | LEMD2 | 139 | S | 1 | 81.64 | 0.86 | 0.88 | 0.82 | 0.81 | 0.90 | 0.82 | 0.64 | 0.68 | 0.71 |
| Q8ND56 | Protein LSM14 homolog A | LSM14A | 216 | S | 1 | 56.6 | 1.17 | 1.12 | 1.23 | 1.09 | 1.13 | 1.06 | NaN | 1.02 | 1.08 |
| Q8ND56 | Protein LSM14 homolog A | LSM14A | 192 | S | 0.986 | 116.1 | 1.24 | NaN | 1.25 | 1.28 | 1.24 | 1.21 | 0.91 | 0.94 | 0.85 |
| Q8ND56 | Protein LSM14 homolog A | LSM14A | 182 | S | 0.896 | 110 | 1.38 | 1.30 | NaN | 1.09 | NaN | 1.25 | 1.26 | NaN | 1.37 |
| Q8ND56 | Protein LSM14 homolog A | LSM14A | 183 | S | 0.942 | 116.8 | 1.16 | 1.23 | 1.33 | 1.25 | 1.10 | 1.12 | 1.47 | 1.18 | 1.45 |
| Q8ND76 | Cyclin-Y | CCNY | 326 | S | 1 | 149.7 | 1.59 | 1.20 | 1.43 | 1.18 | 0.96 | 1.13 | 1.70 | 1.92 | 1.68 |
| Q8NDI1 | EH domain-binding protein 1 | EHBP1 | 428 | S | 0.874 | 130.1 | 0.58 | 0.63 | 0.59 | 1.59 | 0.87 | 0.52 | 0.43 | 2.00 | 0.56 |
| Q8NDI1 | EH domain-binding protein 1 | EHBP1 | 432 | S | 1 | 101.5 | 1.84 | 1.85 | 2.00 | 1.59 | 1.62 | 1.64 | 2.14 | 2.00 | 2.11 |
| Q8NDI1 | EH domain-binding protein 1 | EHBP1 | 436 | S | 1 | 130.1 | 0.91 | 0.82 | 0.92 | 0.84 | 0.87 | 0.92 | 0.91 | 0.80 | 0.87 |
| Q8NDI1 | EH domain-binding protein 1 | EHBP1 | 335 | S | 0.953 | 244.9 | 0.70 | 0.92 | 0.91 | 0.98 | 0.86 | 0.96 | 1.13 | NaN | 0.93 |
| Q8NDI1 | EH domain-binding protein 1 | EHBP1 | 1058 | S | 1 | 98.9 | NaN | 0.80 | 1.06 | 1.67 | 1.36 | 1.19 | 1.81 | 1.65 | 1.06 |
| Q8NDI1 | EH domain-binding protein 1 | EHBP1 | 751 | S | 1 | 67.93 | 1.21 | 1.17 | 1.28 | 1.28 | 1.26 | 1.28 | 1.05 | 1.02 | 1.05 |
| Q8NDI1 | EH domain-binding protein 1 | EHBP1 | 759 | S | 0.962 | 58.7 | NaN | NaN | 2.16 | 2.11 | 2.35 | 2.04 | 0.93 | 1.00 | NaN |
| Q8NDX6 | Zinc finger protein 740 | ZNF740 | 44 | S | 1 | 57.53 | NaN | NaN | NaN | NaN | NaN | NaN | 0.91 | 1.00 | 0.89 |
| Q8NE01 | Metal transporter CNNM3 | CNNM3 | 700 | S | 0.983 | 77.49 | 1.39 | 1.20 | NaN | NaN | NaN | NaN | NaN | NaN | NaN |
| Q8NEF9 | Serum response factor-binding protein 1 | SRFBP1 | 203 | S | 0.956 | 85.36 | 1.68 | NaN | 1.62 | NaN | 1.56 | NaN | 1.21 | 0.82 | NaN |

| | | | | | | | | | | | | | | | |
|---|---|---|---|---|---|---|---|---|---|---|---|---|---|---|---|
| Q8NFC6 | Biorientation of chromosomes in cell division protein 1-like 1 | BOD1L1 | 3029 | S | 1 | 64.19 | NaN | 0.79 | 0.93 | 0.83 | 0.89 | 0.91 | 1.13 | NaN | NaN |
| Q8NFC6 | Biorientation of chromosomes in cell division protein 1-like 1 | BOD1L1 | 2986 | S | 1 | 274.6 | 0.68 | 0.79 | 0.86 | NaN | NaN | 0.72 | 0.52 | 0.92 | 0.77 |
| Q8NFJ5 | Retinoic acid-induced protein | GPRC5A | 345 | S | 1 | 79.39 | 0.78 | NaN | NaN | 0.61 | 0.68 | NaN | NaN | NaN | NaN |
| Q8NHG8 | E3 ubiquitin-protein ligase ZNRF2 | ZNRF2 | 135 | S | 1 | 50.11 | 1.27 | 1.17 | NaN | NaN | NaN | NaN | NaN | NaN | NaN |
| Q8NHV4 | Protein NEDD1 | NEDD1 | 516 | S | 0.99 | 93.22 | 0.67 | 0.53 | NaN | NaN | 0.70 | NaN | NaN | 1.01 | 1.39 |
| Q8NI08 | Nuclear receptor coactivator 7 | NCOA7 | 211 | S | 0.82 | 130.5 | NaN | 0.94 | 0.67 | NaN | NaN | NaN | NaN | 1.10 | 0.90 |
| Q8TAD8 | Smad nuclear-interacting protein 1 | SNIP1 | 35 | S | 1 | 103 | 0.90 | NaN | 1.15 | 0.93 | 0.95 | 0.88 | 1.02 | 0.99 | 1.01 |
| Q8TAP8 | Protein phosphatase 1 regulatory subunit 35 | PPP1R35 | 52 | S | 0.98 | 82.34 | NaN | 2.04 | 1.85 | 1.40 | 1.27 | NaN | 1.56 | 1.51 | NaN |
| Q8TB61 | Adenosine 3'-phospho 5'-phosphosulfate transporter 1 | SLC35B2 | 427 | S | 1 | 103 | 1.64 | 1.30 | 1.36 | 3.49 | 2.27 | 2.02 | 0.83 | 0.99 | 0.80 |
| Q8TCJ2 | Dolichyl-diphosphooligosaccharide--protein glycosyltransferase subunit STT3B | STT3B | 498 | S | 1 | 199 | 1.06 | 1.01 | 0.94 | 0.79 | 0.76 | 0.96 | 0.81 | 0.67 | 1.12 |
| Q8TCJ2 | Dolichyl-diphosphooligosaccharide--protein glycosyltransferase subunit STT3B | STT3B | 499 | S | 1 | 133.8 | 0.93 | 1.01 | 0.99 | 1.02 | 0.73 | 0.99 | 1.09 | 0.82 | 1.02 |
| Q8TD16 | Protein bicaudal D homolog 2 | BICD2 | 582 | S | 1 | 43 | 1.06 | 1.04 | 1.08 | 0.97 | 0.98 | 1.12 | 1.08 | 1.10 | 1.08 |
| Q8TD19 | Serine/threonine-protein kinase Nek9 | NEK9 | 868 | S | 0.5 | 127.2 | 1.54 | 1.56 | 1.45 | 1.81 | NaN | 1.89 | 0.95 | 1.04 | 0.94 |
| Q8TD19 | Serine/threonine-protein kinase Nek9 | NEK9 | 869 | S | 0.619 | 127.2 | 1.43 | 1.56 | 1.45 | 1.81 | NaN | 1.89 | 0.95 | 1.04 | 0.94 |
| Q8TDB6 | E3 ubiquitin-protein ligase DTX3L | DTX3L | 202 | S | 0.994 | 77.26 | NaN | NaN | NaN | 1.10 | 1.14 | 0.90 | NaN | NaN | NaN |
| Q8TDD1 | ATP-dependent RNA helicase DDX54 | DDX54 | 782 | S | 1 | 180.7 | NaN | NaN | 1.87 | 1.44 | 1.62 | 1.34 | NaN | 1.21 | 1.11 |
| Q8TDY2 | RB1-inducible coiled-coil protein 1 | RB1CC1 | 647 | S | 0.896 | 101.3 | NaN | NaN | 1.20 | NaN | 0.60 | 0.52 | NaN | NaN | NaN |
| Q8TDY2 | RB1-inducible coiled-coil protein 1 | RB1CC1 | 237 | S | 0.862 | 75.89 | 0.43 | 0.59 | NaN | NaN | NaN | NaN | NaN | NaN | NaN |
| Q8TEA8 | D-tyrosyl-tRNA(Tyr) deacylase 1 | DTD1 | 194 | S | 0.751 | 182.2 | 1.49 | NaN | NaN | 1.38 | NaN | 1.13 | NaN | 1.91 | 1.38 |
| Q8TEA8 | D-tyrosyl-tRNA(Tyr) deacylase 1 | DTD1 | 196 | S | 0.993 | 261.9 | 1.49 | 2.03 | 1.63 | 1.38 | 1.23 | NaN | 1.82 | 1.44 | NaN |
| Q8TEA8 | D-tyrosyl-tRNA(Tyr) deacylase 1 | DTD1 | 197 | S | 1 | 372.7 | 1.49 | 1.82 | 1.79 | 1.38 | 1.40 | 1.48 | 1.39 | 1.89 | 1.93 |
| Q8TF01 | Arginine/serine-rich protein PNISR | PNISR | 290 | S | 0.941 | 196.8 | 0.86 | NaN | 0.85 | NaN | NaN | 0.70 | 0.77 | NaN | 0.59 |
| Q8WUA2 | Peptidyl-prolyl cis-trans isomerase-like 4 | PPIL4 | 178 | S | 0.97 | 235.1 | NaN | 1.63 | 1.26 | 1.25 | 1.02 | 1.02 | 1.09 | NaN | NaN |

| | | | | | | | | | | | | | | | |
|---|---|---|---|---|---|---|---|---|---|---|---|---|---|---|---|
| Q8WUF5 | RelA-associated inhibitor | PPP1R13L | 567 | S | 0.973 | 49.89 | NaN | NaN | NaN | NaN | NaN | NaN | 0.85 | 1.03 | NaN |
| Q8WUX9 | Charged multivesicular body protein 7 | CHMP7 | 417 | S | 1 | 163.2 | 1.41 | 1.28 | 1.40 | NaN | 0.81 | 1.46 | NaN | NaN | NaN |
| Q8WVC0 | RNA polymerase-associated protein LEO1 | LEO1 | 171 | S | 1 | 97.3 | 0.64 | 0.66 | NaN | 0.99 | NaN | NaN | NaN | 0.80 | NaN |
| Q8WVC0 | RNA polymerase-associated protein LEO1 | LEO1 | 179 | S | 1 | 97.3 | 0.64 | 0.66 | NaN | 0.99 | NaN | NaN | NaN | 0.80 | NaN |
| Q8WVC0 | RNA polymerase-associated protein LEO1 | LEO1 | 630 | S | 0.93 | 106.9 | 0.85 | 0.89 | 0.83 | 1.07 | 0.83 | 0.68 | 0.98 | 1.01 | 0.94 |
| Q8WVC0 | RNA polymerase-associated protein LEO1 | LEO1 | 294 | S | 1 | 121.8 | 0.67 | 1.18 | NaN | 1.13 | 0.49 | 1.00 | 0.79 | 1.53 | 1.04 |
| Q8WVC0 | RNA polymerase-associated protein LEO1 | LEO1 | 296 | S | 1 | 121.8 | 1.10 | 1.18 | NaN | 1.13 | 1.03 | 1.00 | 1.42 | 1.53 | 1.12 |
| Q8WVC0 | RNA polymerase-associated protein LEO1 | LEO1 | 300 | S | 0.99 | 121.8 | 0.67 | 1.18 | NaN | 1.13 | 0.49 | 1.00 | 0.79 | 1.53 | 1.04 |
| Q8WVC0 | RNA polymerase-associated protein LEO1 | LEO1 | 658 | S | 1 | 212.3 | 0.94 | 0.98 | 0.82 | 0.98 | 0.88 | 0.87 | 1.08 | 1.03 | 1.00 |
| Q8WVC0 | RNA polymerase-associated protein LEO1 | LEO1 | 197 | S | 1 | 56.47 | 0.68 | 0.46 | NaN | NaN | NaN | NaN | 0.66 | NaN | NaN |
| Q8WW12 | PEST proteolytic signal-containing nuclear protein | PCNP | 119 | S | 1 | 178.9 | 1.15 | 1.16 | 1.05 | 0.99 | 1.12 | 1.06 | 0.73 | 0.72 | 0.93 |
| Q8WWM7 | Ataxin-2-like protein | ATXN2L | 594 | S | 0.992 | 278.3 | 1.14 | 0.97 | 0.98 | 1.23 | 1.37 | 1.19 | 0.55 | 0.50 | 0.50 |
| Q8WWM7 | Ataxin-2-like protein | ATXN2L | 111 | S | 1 | 138.7 | 2.34 | 2.58 | 2.31 | 2.09 | 2.01 | 1.99 | 0.95 | 0.93 | 0.92 |
| Q8WWM7 | Ataxin-2-like protein | ATXN2L | 449 | S | 1 | 68.89 | 2.18 | 2.09 | 2.17 | 2.25 | 2.02 | 2.18 | 1.17 | 1.19 | 1.23 |
| Q8WWM7 | Ataxin-2-like protein | ATXN2L | 339 | S | 0.987 | 85.47 | 3.00 | 3.50 | 3.73 | NaN | NaN | NaN | NaN | 2.80 | 4.07 |
| Q8WWM7 | Ataxin-2-like protein | ATXN2L | 634 | S | 0.977 | 124 | 0.92 | 0.99 | 0.79 | 0.96 | 0.95 | 1.04 | 0.93 | 0.90 | 0.92 |
| Q8WWQ0 | PH-interacting protein | PHIP | 1281 | S | 1 | 96.53 | 1.45 | NaN | 0.85 | 0.78 | 0.46 | 0.83 | 0.85 | 0.88 | 0.88 |
| Q8WWQ0 | PH-interacting protein | PHIP | 1283 | S | 1 | 96.53 | 1.45 | NaN | 0.85 | 0.78 | 0.46 | 0.83 | 0.85 | 0.88 | 0.88 |
| Q8WWQ0 | PH-interacting protein | PHIP | 1783 | S | 1 | 253.1 | 1.16 | 1.04 | 1.22 | 1.30 | 1.03 | 1.25 | 0.98 | 0.95 | 1.02 |
| Q8WX92 | Negative elongation factor B | NELFB | 557 | S | 0.999 | 125.6 | 0.81 | 0.89 | 0.84 | NaN | 0.99 | 0.75 | 1.17 | NaN | 0.94 |
| Q8WX93 | Palladin | PALLD | 893 | S | 1 | 176.3 | 1.00 | NaN | 0.98 | 1.59 | 1.52 | 1.63 | 1.34 | 1.35 | 1.46 |
| Q8WX93 | Palladin | PALLD | 1118 | S | 1 | 117.3 | 0.56 | 0.43 | NaN | 0.53 | 1.51 | 0.61 | 0.54 | 1.96 | 0.49 |
| Q8WX93 | Palladin | PALLD | 1121 | S | 1 | 214.3 | 0.55 | 0.53 | 0.51 | 0.58 | 0.64 | 0.55 | 0.62 | 0.55 | 0.49 |
| Q8WX93 | Palladin | PALLD | 1116 | S | 1 | 117.3 | 1.57 | 1.49 | NaN | 1.52 | 1.51 | 1.55 | 1.90 | 1.96 | 1.94 |
| Q8WXH0 | Nesprin-2 | SYNE2 | 6361 | S | 0.99 | 108.7 | 0.65 | 0.63 | 0.85 | 1.14 | 1.12 | 1.12 | NaN | 0.78 | 0.78 |
| Q8WYL5 | Protein phosphatase Slingshot homolog 1 | SSH1 | 897 | S | 0.962 | 92.41 | NaN | NaN | 1.24 | NaN | NaN | NaN | NaN | 0.98 | 1.18 |
| Q8WYP5 | Protein ELYS | AHCTF1 | 528 | S | 1 | 78.69 | 1.16 | 0.87 | NaN | 1.65 | 1.99 | NaN | NaN | NaN | NaN |
| Q8WYP5 | Protein ELYS | AHCTF1 | 1944 | S | 0.986 | 54.54 | 0.48 | NaN | 0.52 | 0.53 | NaN | NaN | 0.56 | NaN | 0.76 |
| Q8WYP5 | Protein ELYS | AHCTF1 | 1222 | S | 0.924 | 94.38 | NaN | NaN | NaN | NaN | 1.66 | 1.99 | 1.75 | 1.38 | NaN |
| Q8WYQ5 | Microprocessor complex subunit DGCR8 | DGCR8 | 377 | S | 0.982 | 98.84 | 2.76 | 2.19 | 2.45 | NaN | 2.50 | 2.38 | 0.67 | 1.01 | 0.96 |
| Q8WYQ5 | Microprocessor complex subunit DGCR8 | DGCR8 | 275 | S | 0.995 | 74.28 | NaN | NaN | NaN | 1.63 | 1.81 | NaN | NaN | NaN | NaN |
| Q92508 | Piezo-type mechanosensitive ion channel component 1 | PIEZO1 | 1621 | S | 0.999 | 177.4 | NaN | 0.74 | 0.90 | 0.68 | NaN | NaN | 0.99 | 0.99 | 1.25 |

| | | | | | | | | | | | | | | | |
|---|---|---|---|---|---|---|---|---|---|---|---|---|---|---|---|
| Q92522 | Histone H1x | H1FX | 31 | S | 0.98 | 83.53 | 0.65 | NaN | 0.59 | 1.22 | 1.24 | 1.03 | NaN | 0.61 | NaN |
| Q92538 | Golgi-specific brefeldin A-resistance guanine nucleotide exchange factor 1 | GBF1 | 1780 | S | 0.83 | 44.83 | NaN | NaN | NaN | NaN | NaN | NaN | 10.22 | 4.30 | NaN |
| Q92538 | Golgi-specific brefeldin A-resistance guanine nucleotide exchange factor 1 | GBF1 | 1784 | S | 0.918 | 44.83 | NaN | NaN | NaN | NaN | NaN | NaN | 10.22 | 4.30 | NaN |
| Q92538 | Golgi-specific brefeldin A-resistance guanine nucleotide exchange factor 1 | GBF1 | 1298 | S | 1 | 350.1 | 1.12 | 1.28 | 1.28 | 1.13 | 1.12 | 1.51 | 1.91 | 1.87 | 1.67 |
| Q92538 | Golgi-specific brefeldin A-resistance guanine nucleotide exchange factor 1 | GBF1 | 1318 | S | 0.994 | 130.3 | 1.60 | 1.70 | 1.52 | 1.55 | 1.52 | 1.53 | 2.48 | 2.44 | 2.36 |
| Q92538 | Golgi-specific brefeldin A-resistance guanine nucleotide exchange factor 1 | GBF1 | 1773 | S | 1 | 144.2 | 1.22 | 1.37 | 2.85 | 2.90 | NaN | 2.49 | NaN | NaN | 1.04 |
| Q92576 | PHD finger protein 3 | PHF3 | 1133 | S | 1 | 84.3 | NaN | 0.55 | 0.62 | 0.95 | 1.06 | 1.13 | 0.57 | NaN | 0.62 |
| Q92576 | PHD finger protein 3 | PHF3 | 1642 | S | 1 | 44.72 | 0.90 | 0.76 | NaN | NaN | NaN | NaN | NaN | NaN | NaN |
| Q92614 | Unconventional myosin-XVIIIa | MYO18A | 1998 | S | 1 | 72.82 | 0.61 | 0.59 | NaN | 0.75 | NaN | 0.71 | NaN | NaN | 0.60 |
| Q92614 | Unconventional myosin-XVIIIa | MYO18A | 102 | S | 0.905 | 77.44 | 1.01 | 0.69 | NaN | NaN | NaN | NaN | NaN | NaN | NaN |
| Q92614 | Unconventional myosin-XVIIIa | MYO18A | 1942 | S | 1 | 123.4 | NaN | NaN | NaN | 0.69 | 0.78 | 1.05 | NaN | NaN | NaN |
| Q92614 | Unconventional myosin-XVIIIa | MYO18A | 1970 | S | 1 | 91.1 | 0.75 | 0.74 | NaN | 0.97 | 1.51 | NaN | NaN | NaN | NaN |
| Q92614 | Unconventional myosin-XVIIIa | MYO18A | 2020 | S | 1 | 184.4 | 0.97 | 0.97 | 0.95 | 1.03 | 1.07 | 0.88 | 1.01 | 1.03 | 0.92 |
| Q92614 | Unconventional myosin-XVIIIa | MYO18A | 2007 | S | 0.999 | 115.8 | NaN | 0.78 | 0.62 | 0.75 | 0.73 | 0.76 | 0.61 | 0.60 | 0.54 |
| Q92614 | Unconventional myosin-XVIIIa | MYO18A | 2041 | S | 1 | 185 | 0.94 | 0.71 | 0.93 | 0.86 | 0.92 | 0.90 | 1.02 | 0.93 | 0.84 |
| Q92614 | Unconventional myosin-XVIIIa | MYO18A | 2043 | S | 0.993 | 145.7 | 1.03 | 0.96 | 0.88 | 0.89 | 0.85 | 0.90 | 0.99 | 0.80 | 0.92 |
| Q92619 | Minor histocompatibility protein HA-1;Minor histocompatibility antigen HA- | HMHA1 | 23 | S | 0.996 | 103.7 | NaN | 4.72 | 1.35 | 1.04 | 1.09 | 0.88 | 1.68 | 1.75 | 1.44 |
| Q92667 | A-kinase anchor protein 1, mitochondrial | AKAP1 | 150 | S | 0.699 | 139.5 | 1.36 | 1.37 | NaN | 2.07 | 2.04 | NaN | NaN | NaN | NaN |
| Q92667 | A-kinase anchor protein 1, mitochondrial | AKAP1 | 151 | S | 0.5 | 122.5 | 1.36 | NaN | NaN | 2.07 | 2.04 | NaN | NaN | NaN | NaN |
| Q92733 | Proline-rich protein PRCC | PRCC | 157 | S | 1 | 104.8 | 1.01 | 1.04 | NaN | 0.98 | 1.02 | 1.00 | 1.05 | 0.92 | 1.03 |
| Q92733 | Proline-rich protein PRCC | PRCC | 159 | S | 1 | 104.8 | 1.01 | 1.04 | NaN | 0.98 | 1.02 | 1.00 | 1.05 | 0.92 | 1.03 |
| Q92733 | Proline-rich protein PRCC | PRCC | 267 | S | 1 | 128.7 | 0.76 | 0.73 | 0.66 | 0.63 | 0.68 | 0.72 | 0.59 | 0.66 | 0.62 |
| X6RAB3 | USP6 N-terminal-like protein | USP6NL | 739 | S | 0.948 | 83.88 | 1.62 | 1.41 | 1.28 | 1.42 | 1.40 | NaN | 1.53 | NaN | 1.17 |
| Q92769 | Histone deacetylase 2 | HDAC2 | 422 | S | 1 | 171.3 | 0.47 | NaN | 0.46 | NaN | 0.53 | 0.48 | NaN | 0.45 | NaN |
| Q92769 | Histone deacetylase 2 | HDAC2 | 394 | S | 1 | 243.5 | 0.53 | 0.53 | NaN | 0.71 | 1.39 | NaN | 0.47 | 0.37 | 0.31 |
| Q92797 | Symplekin | SYMPK | 1171 | S | 0.895 | 76.97 | NaN | 0.91 | NaN | NaN | 1.05 | NaN | 1.27 | NaN | 1.76 |
| Q92797 | Symplekin | SYMPK | 1259 | S | 1 | 120.7 | 0.67 | 0.85 | 0.62 | 1.11 | NaN | 1.07 | 0.78 | 0.67 | 0.84 |
| Q92882 | Osteoclast-stimulating factor 1 | OSTF1 | 213 | S | 1 | 281.5 | 0.92 | 0.94 | 0.92 | 0.82 | 0.86 | 0.86 | 1.03 | 1.01 | 0.98 |
| Q92917 | G patch domain and KOW motifs-containing protein | GPKOW | 42 | S | 0.999 | 62.1 | 1.49 | 1.65 | NaN | NaN | NaN | NaN | NaN | NaN | NaN |
| Q92922 | SWI/SNF complex subunit SMARCC1 | SMARCC 1 | 328 | S | 1 | 101.2 | 0.90 | 0.97 | 0.93 | NaN | 1.16 | NaN | 1.01 | 1.02 | 1.23 |

| | | | | | | | | | | | | | | | |
|---|---|---|---|---|---|---|---|---|---|---|---|---|---|---|---|
| Q92922 | SWI/SNF complex subunit SMARCC1 | SMARCC 1 | 330 | S | 1 | 101.2 | 0.90 | 0.97 | 0.93 | NaN | 1.16 | NaN | 1.01 | 1.02 | 1.23 |
| V9GYM8 | Rho guanine nucleotide exchange factor 2 | ARHGEF 2 | 741 | S | 1 | 111.2 | NaN | NaN | NaN | 1.21 | 1.42 | 1.30 | 0.84 | 0.90 | 1.05 |
| V9GYM8 | Rho guanine nucleotide exchange factor 2 | ARHGEF 2 | 977 | S | 1 | 139.9 | 0.78 | 0.72 | 0.61 | 0.85 | 0.76 | 0.81 | 0.92 | 0.89 | NaN |
| V9GYM8 | Rho guanine nucleotide exchange factor 2 | ARHGEF 2 | 1005 | S | 0.988 | 70.18 | NaN | 1.05 | 0.90 | NaN | 0.87 | 1.12 | NaN | 0.91 | 1.00 |
| V9GYM8 | Rho guanine nucleotide exchange factor 2 | ARHGEF 2 | 690 | S | 0.99 | 76.28 | 5.38 | 5.97 | 5.34 | 4.20 | 4.83 | NaN | NaN | NaN | NaN |
| Q93008 | Probable ubiquitin carboxyl-terminal hydrolase FAF-X | USP9X | 2563 | S | 0.997 | 70.7 | NaN | NaN | 0.81 | 1.54 | 1.66 | NaN | 0.88 | 0.96 | NaN |
| Q969E4 | Transcription elongation factor A protein-like 3 | TCEAL3 | 65 | S | 1 | 242.2 | 1.32 | 1.25 | 1.21 | 1.04 | 1.17 | 1.09 | 1.26 | 1.32 | 1.11 |
| Q969E4 | Transcription elongation factor A protein-like 3;Transcription elongation factor A protein-like 6;Transcription elongation factor A protein-like 5 | TCEAL3 | 121 | S | 1 | 276 | 0.80 | 0.80 | 0.89 | 1.11 | 1.06 | 1.15 | 1.16 | 1.26 | 1.14 |
| Q969E4 | Transcription elongation factor A protein-like 3;Transcription elongation factor A protein-like 6;Transcription elongation factor A protein-like 5 | TCEAL3 | 125 | S | 1 | 130.7 | 2.49 | 2.01 | NaN | NaN | NaN | NaN | 2.80 | 2.25 | NaN |
| Q969S3 | Zinc finger protein 622 | ZNF622 | 141 | S | 0.794 | 80.69 | NaN | 1.11 | 1.05 | 1.61 | 1.65 | 1.66 | NaN | 0.49 | 0.43 |
| Q969S3 | Zinc finger protein 622 | ZNF622 | 143 | S | 0.76 | 71.35 | 1.12 | NaN | 1.05 | NaN | NaN | NaN | 0.51 | NaN | NaN |
| Q96AE4 | Far upstream element-binding protein 1 | FUBP1 | 630 | S | 0.906 | 109.9 | 1.03 | NaN | NaN | 1.44 | 1.49 | NaN | NaN | NaN | NaN |
| Q96AT1 | Uncharacterized protein KIAA1143 | KIAA1143 | 50 | S | 1 | 178.9 | 0.67 | 0.77 | 0.63 | 0.74 | 0.68 | 0.76 | 0.76 | 0.81 | 0.80 |
| Q96B36 | Proline-rich AKT1 substrate 1 | AKT1S1 | 88 | S | 0.994 | 119.7 | 1.17 | NaN | 1.10 | 1.30 | 1.14 | 1.11 | NaN | NaN | 1.00 |
| Q96B36 | Proline-rich AKT1 substrate 1 | AKT1S1 | 92 | S | 0.879 | 119.7 | 1.36 | NaN | 1.07 | 1.30 | 1.14 | 1.11 | NaN | NaN | NaN |
| Q96B36 | Proline-rich AKT1 substrate 1 | AKT1S1 | 203 | S | 0.999 | 221.9 | 1.03 | 0.90 | 1.12 | 0.98 | 0.93 | 0.93 | 1.21 | 1.33 | 1.14 |
| Q96B36 | Proline-rich AKT1 substrate 1 | AKT1S1 | 211 | S | 0.839 | 211.1 | 1.05 | 0.99 | 1.03 | NaN | 0.96 | 0.96 | 1.21 | 1.33 | NaN |
| Q96B36 | Proline-rich AKT1 substrate 1 | AKT1S1 | 212 | S | 0.9 | 229.8 | 0.93 | 0.90 | 1.22 | 0.98 | 0.99 | 0.90 | NaN | 1.22 | 1.14 |
| Q96C19 | EF-hand domain-containing protein D2 | EFHD2 | 74 | S | 0.959 | 126 | 3.70 | 3.35 | 3.21 | 2.32 | 2.48 | 3.22 | NaN | 1.92 | NaN |
| Q96C19 | EF-hand domain-containing protein D2 | EFHD2 | 76 | S | 0.5 | 65.44 | NaN | NaN | NaN | 2.64 | 3.39 | NaN | NaN | NaN | NaN |
| Q96C90 | Protein phosphatase 1 regulatory subunit 14B | PPP1R14 B | 32 | S | 1 | 76.82 | 5.00 | 3.75 | NaN | 4.39 | 3.29 | NaN | NaN | NaN | NaN |
| Q96CW6 | Probable RNA polymerase II nuclear localization protein SLC7A6OS | SLC7A6O S | 308 | S | 1 | 178.4 | 0.70 | 0.56 | 0.66 | 0.96 | 0.98 | 0.87 | 0.71 | 0.70 | 0.75 |
| Q96CX2 | BTB/POZ domain-containing protein KCTD12 | KCTD12 | 176 | S | 0.807 | 137.5 | 0.91 | 0.98 | 0.88 | NaN | 1.40 | 1.19 | NaN | NaN | NaN |

| | | | | | | | | | | | | | | | |
|---|---|---|---|---|---|---|---|---|---|---|---|---|---|---|---|
| Q96CX2 | BTB/POZ domain-containing protein KCTD12 | KCTD12 | 185 | S | 0.887 | 105.7 | NaN | 0.91 | 0.76 | 0.96 | 0.90 | 1.12 | 0.78 | 0.72 | 1.07 |
| Q96CX2 | BTB/POZ domain-containing protein KCTD12 | KCTD12 | 187 | S | 0.54 | 97.49 | 0.94 | 0.91 | NaN | NaN | 0.90 | 1.12 | NaN | NaN | NaN |
| Q96E09 | Protein FAM122A | FAM122A | 270 | S | 0.942 | 87.2 | 0.81 | 0.96 | NaN | 0.91 | 0.81 | 1.00 | 1.21 | 1.20 | 1.30 |
| Q96EB6 | NAD-dependent protein deacetylase sirtuin-1;SirtT1 75 kDa fragment | SIRT1 | 27 | S | 0.936 | 97.96 | NaN | NaN | 0.84 | 0.89 | NaN | 1.25 | 1.22 | 1.13 | 1.17 |
| Q96EU6 | Ribosomal RNA processing protein 36 homolog | RRP36 | 73 | S | 1 | 88.19 | 1.19 | 0.98 | 0.78 | 1.14 | 1.12 | 1.15 | 0.58 | 0.55 | NaN |
| Q96EV2 | RNA-binding protein 33 | RBM33 | 41 | S | 1 | 139.7 | 2.02 | 2.02 | NaN | NaN | NaN | NaN | 2.35 | 2.07 | 0.76 |
| Q96EV2 | RNA-binding protein 33 | RBM33 | 205 | S | 1 | 248.7 | 0.85 | 1.13 | 1.03 | 0.99 | 1.02 | 1.00 | 0.94 | 0.95 | 1.02 |
| Q96EV2 | RNA-binding protein 33 | RBM33 | 765 | S | 1 | 79.99 | NaN | 0.81 | 0.83 | 1.00 | 0.91 | 0.95 | 0.76 | 0.86 | 0.82 |
| Q96G46 | tRNA-dihydrouridine(47) synthase [NAD(P)(+)]-like | DUS3L | 276 | S | 0.629 | 101 | NaN | NaN | NaN | 0.94 | 0.65 | NaN | NaN | 1.32 | 1.16 |
| Q96GM8 | Target of EGR1 protein 1 | TOE1 | 5 | S | 1 | 241.9 | 0.69 | 0.62 | NaN | NaN | NaN | 0.69 | 0.69 | NaN | 0.89 |
| S4R3D6 | Methylthioribulose-1-phosphate dehydratase | APIP | 40 | S | 0.974 | 114.7 | 1.02 | 1.11 | 1.18 | 0.97 | 1.01 | 1.06 | 1.17 | 0.86 | 1.08 |
| Q96HR8 | H/ACA ribonucleoprotein complex non-core subunit | NAF1 | 315 | S | 1 | 94 | 0.96 | 1.14 | 1.14 | 1.25 | 1.18 | 1.45 | 1.00 | 1.11 | 1.07 |
| Q96I25 | Splicing factor 45 | RBM17 | 222 | S | 0.859 | 179.7 | 0.70 | 0.57 | 0.69 | NaN | NaN | 0.88 | 0.61 | NaN | NaN |
| Q96I25 | Splicing factor 45 | RBM17 | 155 | S | 1 | 188.3 | 0.99 | 1.01 | 0.90 | 0.92 | 0.93 | 1.04 | 1.06 | 1.03 | 0.82 |
| Q96IF1 | LIM domain-containing protein ajuba | AJUBA | 119 | S | 1 | 144.6 | NaN | 1.68 | 1.49 | 1.49 | 1.54 | NaN | 1.12 | 1.08 | NaN |
| Q96IF1 | LIM domain-containing protein ajuba | AJUBA | 137 | S | 0.999 | 401.3 | 1.47 | 1.15 | NaN | 1.13 | 1.07 | 0.83 | 0.84 | 0.73 | 0.70 |
| Q96JM3 | Chromosome alignment-maintaining phosphoprotein 1 | CHAMP1 | 416 | S | 1 | 85.81 | 1.03 | 0.87 | 1.13 | NaN | NaN | 1.58 | 1.04 | 0.97 | NaN |
| Q96JM3 | Chromosome alignment-maintaining phosphoprotein 1 | CHAMP1 | 476 | S | 1 | 135.6 | 0.84 | 0.88 | 0.86 | 0.95 | 1.04 | 1.08 | 1.09 | 1.03 | 1.17 |
| Q96JM3 | Chromosome alignment-maintaining phosphoprotein 1 | CHAMP1 | 651 | S | 0.911 | 106.9 | NaN | 0.70 | 0.80 | 0.82 | 0.68 | 0.82 | 0.91 | 1.06 | 0.72 |
| Q96JM3 | Chromosome alignment-maintaining phosphoprotein 1 | CHAMP1 | 652 | S | 0.787 | 73.68 | NaN | NaN | NaN | NaN | 0.68 | 0.82 | 0.91 | 1.06 | 0.72 |
| Q96JM3 | Chromosome alignment-maintaining phosphoprotein 1 | CHAMP1 | 653 | S | 0.818 | 106.9 | NaN | 0.70 | 0.80 | 0.82 | 0.68 | NaN | NaN | NaN | 0.72 |
| Q96JM3 | Chromosome alignment-maintaining phosphoprotein 1 | CHAMP1 | 427 | S | 1 | 58.78 | 1.53 | NaN | 1.54 | 1.99 | 2.21 | 1.76 | 0.82 | 1.78 | 1.30 |
| Q96JM3 | Chromosome alignment-maintaining phosphoprotein 1 | CHAMP1 | 432 | S | 1 | 58.78 | 1.53 | NaN | 1.54 | 1.99 | 2.21 | 1.76 | NaN | 1.78 | 1.78 |
| Q96JM3 | Chromosome alignment-maintaining phosphoprotein 1 | CHAMP1 | 436 | S | 1 | 58.78 | 1.53 | NaN | 1.54 | 1.99 | 2.21 | 1.76 | NaN | 1.78 | 1.78 |
| Q96JM3 | Chromosome alignment-maintaining phosphoprotein 1 | CHAMP1 | 443 | S | 0.737 | 56.47 | NaN | NaN | NaN | 0.98 | 0.96 | 0.88 | 1.04 | 0.91 | NaN |
| Q96JM3 | Chromosome alignment-maintaining phosphoprotein 1 | CHAMP1 | 445 | S | 0.598 | 83.25 | 0.46 | 0.58 | NaN | 0.98 | 0.96 | 0.88 | 0.59 | NaN | 0.40 |

| | | | | | | | | | | | | | | |
|---|---|---|---|---|---|---|---|---|---|---|---|---|---|---|
| Q96JM3 | Chromosome alignment-maintaining phosphoprotein 1 | CHAMP1 | 452 | S | 1 | 83.25 | 0.46 | 0.58 | NaN | 1.10 | 0.94 | 0.88 | 0.59 | NaN | 0.40 |
| Q96JM3 | Chromosome alignment-maintaining phosphoprotein 1 | CHAMP1 | 282 | S | 0.983 | 86.3 | 0.44 | 0.92 | 0.53 | 0.48 | NaN | 0.46 | 0.86 | 0.87 | 1.24 |
| Q96JM3 | Chromosome alignment-maintaining phosphoprotein 1 | CHAMP1 | 405 | S | 0.993 | 79.35 | 1.19 | 0.71 | NaN | 0.96 | 1.13 | 0.92 | 0.49 | 0.58 | 0.81 |
| Q96JM3 | Chromosome alignment-maintaining phosphoprotein 1 | CHAMP1 | 204 | S | 1 | 115.1 | 0.59 | 0.64 | 0.63 | 0.75 | 0.75 | 0.64 | 0.81 | 0.90 | 0.84 |
| Q96JM3 | Chromosome alignment-maintaining phosphoprotein 1 | CHAMP1 | 214 | S | 0.996 | 115.1 | 0.77 | 0.60 | 0.68 | 0.98 | 0.99 | 0.91 | 0.85 | 0.82 | 0.83 |
| Q96JM3 | Chromosome alignment-maintaining phosphoprotein 1 | CHAMP1 | 382 | S | 0.715 | 69.47 | NaN | NaN | 1.29 | 2.05 | 1.73 | NaN | 1.17 | 1.22 | NaN |
| Q96JM3 | Chromosome alignment-maintaining phosphoprotein 1 | CHAMP1 | 386 | S | 0.964 | 69.47 | NaN | NaN | 1.29 | 2.05 | 1.73 | NaN | 1.17 | 1.22 | NaN |
| Q96JM3 | Chromosome alignment-maintaining phosphoprotein 1 | CHAMP1 | 275 | S | 0.993 | 114.2 | 0.70 | NaN | NaN | NaN | NaN | NaN | 1.06 | NaN | 0.79 |
| Q96K76 | Ubiquitin carboxyl-terminal hydrolase 47 | USP47 | 910 | S | 1 | 220.1 | 1.09 | NaN | NaN | NaN | NaN | NaN | 0.72 | 1.07 | NaN |
| Q96KR1 | Zinc finger RNA-binding | ZFR | 1054 | S | 1 | 115.6 | 1.07 | 0.99 | 1.05 | 0.91 | 0.98 | 0.95 | 1.14 | 1.17 | 1.51 |
| Q96N66 | Lysophospholipid acyltransferase 7 | MBOAT7 | 284 | S | 0.933 | 157.4 | 1.00 | 0.99 | 1.21 | 1.74 | 1.69 | 1.67 | 1.00 | 0.92 | NaN |
| Q96N66 | Lysophospholipid acyltransferase 7 | MBOAT7 | 285 | S | 0.529 | 76.03 | 1.09 | NaN | 1.21 | 1.59 | NaN | 1.55 | 1.00 | 0.92 | NaN |
| Q96N67 | Dedicator of cytokinesis protein 7 | DOCK7 | 900 | S | 0.998 | 268.4 | 0.89 | NaN | 1.04 | 1.18 | 1.44 | NaN | 3.18 | NaN | NaN |
| Q96N67 | Dedicator of cytokinesis protein 7 | DOCK7 | 910 | S | 0.873 | 207 | NaN | NaN | 1.04 | 1.18 | 0.98 | NaN | 3.18 | 0.88 | NaN |
| Q96NE9 | FERM domain-containing protein 6 | FRMD6 | 522 | S | 0.997 | 151.6 | NaN | NaN | 1.82 | 1.13 | 1.10 | NaN | NaN | 2.94 | 2.65 |
| Q96NE9 | FERM domain-containing protein 6 | FRMD6 | 544 | S | 1 | 127.2 | 2.31 | 1.98 | NaN | 1.17 | 1.12 | 1.30 | 2.06 | 2.40 | 1.91 |
| Q96NY7 | Chloride intracellular channel protein 6 | CLIC6 | 293 | S | 1 | 156.2 | 2.04 | 2.11 | 1.92 | 0.99 | 1.68 | NaN | NaN | 1.20 | NaN |
| Q96NY7 | Chloride intracellular channel protein 6 | CLIC6 | 305 | S | 0.999 | 156.2 | 2.04 | 2.11 | 1.92 | 1.96 | 1.68 | NaN | NaN | 1.20 | NaN |
| Q96P48 | Arf-GAP with Rho-GAP domain, ANK repeat and PH domain-containing protein 1 | ARAP1 | 229 | S | 1 | 102.6 | 0.76 | 0.63 | 0.59 | 0.77 | 0.71 | NaN | 1.36 | 1.33 | 1.30 |
| Q96PK6 | RNA-binding protein 14 | RBM14 | 215 | S | 1 | 68.17 | 1.41 | NaN | 1.24 | 1.09 | 1.19 | 1.19 | 1.30 | 1.27 | 1.25 |
| Q96PK6 | RNA-binding protein 14 | RBM14 | 220 | S | 1 | 68.17 | 1.41 | NaN | 1.24 | 1.09 | 1.19 | 1.19 | 1.30 | 1.27 | 1.25 |
| Q96PX8 | SLIT and NTRK-like protein 1 | SLITRK1 | 586 | S | 0.986 | 79.04 | NaN | NaN | 1.05 | 0.91 | 0.70 | NaN | 1.17 | 1.14 | NaN |
| Q96QB1 | Rho GTPase-activating protein 7 | DLC1 | 523 | S | 1 | 91.73 | 1.42 | NaN | 2.02 | 1.04 | 1.10 | NaN | 1.88 | 1.76 | 2.03 |
| Q96QB1 | Rho GTPase-activating protein 7 | DLC1 | 526 | S | 1 | 180.7 | 1.42 | NaN | 2.02 | 1.04 | 1.10 | NaN | 1.88 | 1.76 | 2.03 |

| | | | | | | | | | | | | | | |
|---|---|---|---|---|---|---|---|---|---|---|---|---|---|---|
| Q96QC0 | Serine/threonine-protein phosphatase 1 regulatory subunit 10 | PPP1R10 | 545 | S | 0.93 | 88.72 | 0.40 | 0.59 | NaN | NaN | 0.96 | 0.95 | 0.49 | NaN | NaN |
| Q96QC0 | Serine/threonine-protein phosphatase 1 regulatory subunit 10 | PPP1R10 | 313 | S | 0.997 | 78.92 | 1.74 | 2.44 | 2.34 | 1.99 | 1.93 | 2.23 | 1.46 | 1.55 | NaN |
| Q96QR8 | Transcriptional activator protein Pur-beta | PURB | 304 | S | 1 | 165.2 | 1.36 | 1.43 | 1.59 | 1.22 | 1.17 | 1.33 | 1.38 | 1.51 | 1.61 |
| Q96RL1 | BRCA1-A complex subunit RAP80 | UIMC1 | 677 | S | 1 | 57.2 | NaN | NaN | NaN | NaN | 1.27 | 1.14 | 1.21 | NaN | 1.42 |
| Q96RT1 | Protein LAP2 | ERBB2IP | 852 | S | 0.877 | 68.36 | 1.07 | NaN | NaN | NaN | 0.60 | NaN | 1.35 | 0.97 | NaN |
| Q96RT1 | Protein LAP2 | ERBB2IP | 857 | S | 1 | 97.45 | 1.18 | 0.27 | NaN | 1.44 | 1.56 | 0.95 | 1.02 | NaN | 1.07 |
| Q96RU3 | Formin-binding protein 1 | FNBP1 | 359 | S | 0.999 | 94.73 | 0.74 | 0.73 | 0.66 | 0.79 | 0.80 | 0.74 | 0.74 | 0.75 | 0.71 |
| Q96S55 | ATPase WRNIP1 | WRNIP1 | 65 | S | 1 | 78.52 | NaN | NaN | 0.73 | 0.80 | 0.85 | 0.87 | 0.88 | 0.85 | 0.82 |
| Q96S94 | Cyclin-L2 | CCNL2 | 369 | S | 1 | 70.52 | NaN | NaN | 1.08 | NaN | NaN | NaN | 1.08 | 0.99 | NaN |
| Q96S94 | Cyclin-L2 | CCNL2 | 348 | S | 0.933 | 40.73 | NaN | NaN | 0.92 | NaN | 0.78 | NaN | 1.02 | 1.15 | NaN |
| Q96ST2 | Protein IWS1 homolog | IWS1 | 398 | S | 1 | 105.5 | 0.96 | 1.01 | 0.97 | 0.94 | 0.93 | 0.99 | 1.09 | 1.11 | 1.15 |
| Q96ST2 | Protein IWS1 homolog | IWS1 | 400 | S | 1 | 105.5 | 0.96 | 1.01 | 0.97 | 0.94 | 0.93 | 0.99 | 1.09 | 1.11 | 1.15 |
| Q96ST2 | Protein IWS1 homolog | IWS1 | 511 | S | 1 | 127.1 | 0.74 | 0.87 | 0.89 | 0.93 | 0.89 | 1.14 | 0.97 | 1.00 | 1.45 |
| Q96ST2 | Protein IWS1 homolog | IWS1 | 513 | S | 1 | 127.1 | 0.74 | 0.87 | 0.89 | 0.93 | 0.89 | 1.14 | 0.97 | 1.00 | 1.45 |
| Q96ST2 | Protein IWS1 homolog | IWS1 | 235 | S | 1 | 52.27 | NaN | NaN | NaN | NaN | 0.89 | 0.88 | 1.21 | 1.00 | 1.17 |
| Q96ST2 | Protein IWS1 homolog | IWS1 | 237 | S | 1 | 52.27 | NaN | NaN | NaN | NaN | 0.89 | 0.88 | 1.21 | 1.00 | 1.17 |
| Q96ST2 | Protein IWS1 homolog | IWS1 | 196 | S | 1 | 46.57 | NaN | NaN | 1.71 | 1.44 | 1.34 | 0.81 | NaN | 1.50 | 1.66 |
| Q96ST2 | Protein IWS1 homolog | IWS1 | 198 | S | 1 | 46.57 | NaN | NaN | 1.71 | 1.44 | 1.34 | NaN | NaN | 1.50 | 1.66 |
| Q96ST2 | Protein IWS1 homolog | IWS1 | 248 | S | 1 | 74.78 | 1.32 | 1.47 | 1.46 | 1.68 | 1.60 | 0.76 | 1.87 | 1.50 | 0.98 |
| Q96ST2 | Protein IWS1 homolog | IWS1 | 250 | S | 0.996 | 74.78 | 1.32 | 1.47 | 1.46 | 1.68 | 1.60 | NaN | 1.87 | 1.50 | NaN |
| Q96ST2 | Protein IWS1 homolog | IWS1 | 261 | S | 0.894 | 49 | 0.85 | 0.94 | 0.93 | NaN | 0.62 | 0.76 | NaN | 0.98 | 0.98 |
| Q96ST2 | Protein IWS1 homolog | IWS1 | 263 | S | 0.925 | 49 | 0.85 | 0.94 | 0.93 | NaN | 0.62 | 0.76 | NaN | 0.98 | 0.98 |
| Q96ST2 | Protein IWS1 homolog | IWS1 | 274 | S | 0.977 | 59.36 | NaN | 0.91 | 0.97 | NaN | 0.85 | NaN | NaN | 0.94 | 0.70 |
| Q96ST2 | Protein IWS1 homolog | IWS1 | 287 | S | 1 | 92.68 | NaN | 0.91 | 0.97 | NaN | 0.85 | NaN | NaN | 0.94 | NaN |
| Q96ST2 | Protein IWS1 homolog | IWS1 | 289 | S | 1 | 92.68 | NaN | 0.91 | 0.97 | NaN | 0.85 | NaN | NaN | 0.94 | 0.70 |
| Q96ST2 | Protein IWS1 homolog | IWS1 | 377 | S | 1 | 131.7 | 0.74 | 0.70 | 0.70 | 0.70 | 0.87 | 0.80 | 0.72 | 0.63 | 0.80 |
| Q96ST2 | Protein IWS1 homolog | IWS1 | 438 | S | 1 | 199.8 | 0.99 | 0.89 | 1.05 | 0.94 | 0.92 | 0.90 | 0.96 | 1.22 | 1.02 |
| Q96ST2 | Protein IWS1 homolog | IWS1 | 440 | S | 1 | 199.8 | 0.99 | 0.89 | 1.05 | 0.94 | 0.92 | 0.90 | 0.96 | 1.22 | 1.02 |
| Q96ST2 | Protein IWS1 homolog | IWS1 | 300 | S | 0.999 | 101.2 | NaN | NaN | 0.45 | 0.42 | NaN | 0.67 | 0.43 | 1.11 | 0.60 |
| Q96ST2 | Protein IWS1 homolog | IWS1 | 313 | S | 0.996 | 101.2 | NaN | NaN | 0.45 | 0.42 | NaN | 0.50 | 0.43 | 0.37 | 0.60 |
| Q96ST2 | Protein IWS1 homolog | IWS1 | 415 | S | 1 | 166.7 | 0.36 | NaN | NaN | NaN | 0.34 | 0.40 | NaN | NaN | 0.41 |
| Q96ST2 | Protein IWS1 homolog | IWS1 | 420 | S | 1 | 196.7 | 0.36 | NaN | 0.36 | 0.28 | NaN | 0.40 | 0.34 | 0.38 | 0.41 |
| Q96ST2 | Protein IWS1 homolog | IWS1 | 422 | S | 1 | 196.7 | NaN | NaN | 0.36 | 0.28 | 0.34 | NaN | 0.34 | 0.38 | NaN |
| Q96ST3 | Paired amphipathic helix protein Sin3a | SIN3A | 832 | S | 1 | 196.5 | NaN | 0.49 | 0.47 | NaN | 0.87 | 0.73 | NaN | 0.72 | 0.75 |
| Q96T23 | Remodeling and spacing factor 1 | RSF1 | 604 | S | 1 | 92.83 | 0.77 | 0.77 | 0.75 | 1.00 | 0.72 | 0.81 | 0.93 | 1.07 | 0.97 |
| Q96T23 | Remodeling and spacing factor 1 | RSF1 | 1345 | S | 1 | 138.3 | 0.91 | NaN | NaN | NaN | 0.57 | 0.76 | NaN | NaN | 1.02 |
| Q96T23 | Remodeling and spacing factor 1 | RSF1 | 397 | S | 1 | 55.45 | 0.63 | NaN | 0.77 | NaN | 1.12 | 1.01 | 0.68 | 0.78 | 0.59 |

| | | | | | | | | | | | | | | | |
|---|---|---|---|---|---|---|---|---|---|---|---|---|---|---|---|
| Q96T23 | Remodeling and spacing factor 1 | RSF1 | 622 | S | 0.999 | 116.9 | 0.76 | 0.72 | 0.69 | 0.96 | 0.73 | 0.78 | 0.94 | 1.01 | 1.07 |
| Q96T23 | Remodeling and spacing factor 1 | RSF1 | 629 | S | 0.773 | 100.8 | 0.73 | 0.84 | NaN | 0.96 | 0.99 | 0.65 | 0.89 | NaN | 1.07 |
| Q96T23 | Remodeling and spacing factor 1 | RSF1 | 1359 | S | 0.984 | 65.89 | 0.64 | 0.69 | 0.54 | 0.59 | 0.69 | 0.58 | 0.70 | 0.51 | 0.76 |
| Q96T23 | Remodeling and spacing factor 1 | RSF1 | 1375 | S | 0.904 | 65.89 | 0.64 | 0.69 | 0.54 | 0.59 | 0.69 | 0.58 | 0.70 | 0.51 | 0.76 |
| Q96T58 | Msx2-interacting protein | SPEN | 727 | S | 1 | 64.06 | 0.95 | 0.76 | NaN | NaN | NaN | NaN | NaN | NaN | NaN |
| Q96TA1 | Niban-like protein 1 | FAM129B | 691 | S | 0.89 | 232.1 | NaN | 1.05 | 2.74 | 2.98 | 0.81 | 0.83 | 0.95 | 1.00 | 1.32 |
| Q96TA1 | Niban-like protein 1 | FAM129B | 692 | S | 0.995 | 261.7 | 1.10 | 1.15 | 1.07 | 0.80 | NaN | 0.65 | 1.07 | 0.83 | 1.01 |
| Q96TA1 | Niban-like protein 1 | FAM129B | 696 | S | 1 | 211.3 | 2.65 | 2.71 | 2.92 | 2.59 | 2.49 | 2.64 | 1.31 | 1.28 | 1.32 |
| Q96TA1 | Niban-like protein 1 | FAM129B | 665 | S | 1 | 116.4 | 2.00 | 1.93 | 1.68 | 2.25 | 3.02 | 2.09 | 1.51 | 1.25 | 0.98 |
| Q96TA1 | Niban-like protein 1 | FAM129B | 681 | S | 1 | 112.7 | 2.00 | 1.93 | 1.68 | 2.25 | 3.02 | 2.09 | 0.95 | NaN | 0.98 |
| Q96TA1 | Niban-like protein 1 | FAM129B | 638 | S | 1 | 107 | 2.91 | 3.29 | 3.57 | 3.45 | 4.21 | 4.98 | NaN | NaN | NaN |
| Q96TA1 | Niban-like protein 1 | FAM129B | 641 | S | 1 | 147.2 | 3.43 | 3.54 | 3.10 | 2.70 | 4.21 | 2.82 | NaN | NaN | NaN |
| Q96TA1 | Niban-like protein 1 | FAM129B | 646 | S | 0.998 | 147.2 | 3.43 | 3.54 | 3.10 | 2.70 | 4.21 | 2.82 | NaN | NaN | NaN |
| Q99459 | Cell division cycle 5-like | CDC5L | 303 | S | 1 | 157.3 | 1.32 | 2.11 | 2.07 | 1.62 | 1.84 | 1.75 | 2.65 | 2.42 | 2.85 |
| Q99523 | Sortilin | SORT1 | 825 | S | 1 | 156 | 0.94 | 0.93 | NaN | 0.72 | 0.80 | 0.68 | 0.82 | 0.73 | 0.76 |
| Q99549 | M-phase phosphoprotein 8 | MPHOSPH8 | 164 | S | 1 | 51.61 | NaN | 0.46 | NaN | 0.47 | NaN | 0.59 | 0.59 | 0.64 | 0.73 |
| Q99549 | M-phase phosphoprotein 8 | MPHOSPH8 | 51 | S | 1 | 250 | 0.52 | 0.47 | NaN | 0.71 | 0.58 | 0.46 | 0.63 | 0.58 | 0.79 |
| Q99549 | M-phase phosphoprotein 8 | MPHOSPH8 | 403 | S | 0.929 | 120.3 | 0.56 | NaN | NaN | 0.57 | NaN | 0.99 | 1.09 | 0.86 | NaN |
| Q99549 | M-phase phosphoprotein 8 | MPHOSPH8 | 85 | S | 0.964 | 166.2 | 0.44 | 0.37 | 0.38 | 0.54 | 0.45 | 0.50 | 0.61 | 0.58 | 0.65 |
| Q99567 | Nuclear pore complex protein Nup88 | NUP88 | 517 | S | 1 | 71.51 | 1.42 | 1.28 | NaN | NaN | NaN | NaN | NaN | NaN | NaN |
| Q99567 | Nuclear pore complex protein Nup88 | NUP88 | 35 | S | 0.995 | 107 | NaN | NaN | NaN | 2.01 | 1.65 | 1.42 | 1.58 | 1.64 | NaN |
| Q99638 | Cell cycle checkpoint control protein RAD9A | RAD9A | 375 | S | 0.675 | 40.76 | NaN | NaN | NaN | NaN | NaN | 1.65 | 1.98 | 1.60 | 1.61 |
| Q99638 | Cell cycle checkpoint control protein RAD9A | RAD9A | 380 | S | 0.674 | 40.76 | NaN | NaN | NaN | NaN | NaN | 1.65 | 1.98 | 1.60 | 1.61 |
| Q99638 | Cell cycle checkpoint control protein RAD9A | RAD9A | 387 | S | 1 | 112.7 | NaN | NaN | NaN | NaN | NaN | 1.65 | 1.98 | 1.60 | 1.61 |
| Q99959 | Plakophilin-2 | PKP2 | 151 | S | 1 | 97.42 | NaN | NaN | 1.10 | NaN | 0.83 | 1.11 | 1.13 | 0.80 | 0.92 |
| Q99959 | Plakophilin-2 | PKP2 | 154 | S | 0.823 | 97.42 | NaN | NaN | 1.10 | NaN | NaN | 1.11 | 1.13 | 0.80 | 0.92 |
| Q9BPX3 | Condensin complex subunit 3 | NCAPG | 975 | S | 1 | 167.3 | NaN | NaN | NaN | 0.81 | 0.88 | 0.66 | NaN | 0.72 | NaN |
| Q9BPX3 | Condensin complex subunit 3 | NCAPG | 1015 | S | 1 | 151.7 | 1.41 | 1.33 | 1.23 | 1.31 | 1.43 | 1.14 | 1.06 | 1.09 | 1.15 |
| Q9BPX3 | Condensin complex subunit 3 | NCAPG | 674 | S | 1 | 376.2 | 0.82 | 0.71 | 0.93 | 0.85 | 0.86 | 0.97 | 0.94 | 0.93 | 0.93 |
| Q9BQG0 | Myb-binding protein 1A | MYBBP1 | 11 | S | 0.996 | 79.46 | 0.88 | NaN | NaN | NaN | 1.18 | 1.33 | NaN | NaN | NaN |
| Q9BQG0 | Myb-binding protein 1A | MYBBP1 | 1163 | S | 0.961 | 112.6 | 0.87 | 0.86 | 0.88 | 1.41 | 1.50 | NaN | 0.66 | 0.66 | 0.63 |
| Q9BRD0 | BUD13 homolog | BUD13 | 357 | S | 0.731 | 147.2 | 0.71 | 0.79 | NaN | 0.81 | 0.80 | NaN | 0.94 | 0.98 | 0.84 |
| Q9BRD0 | BUD13 homolog | BUD13 | 197 | S | 1 | 70.09 | NaN | 0.82 | 0.90 | 0.88 | 0.87 | 0.95 | 0.99 | 0.98 | 1.04 |
| Q9BRD0 | BUD13 homolog | BUD13 | 201 | S | 1 | 70.09 | NaN | 0.82 | 0.90 | 0.88 | 0.87 | 0.95 | 0.99 | 0.98 | 1.04 |

| | | | | | | | | | | | | | | |
|---|---|---|---|---|---|---|---|---|---|---|---|---|---|---|
| Q9BRD0 | BUD13 homolog | BUD13 | 163 | S | 1 | 58.78 | 1.42 | 1.92 | NaN | NaN | NaN | NaN | 2.23 | 2.11 | NaN |
| Q9BRD0 | BUD13 homolog | BUD13 | 139 | S | 1 | 71.56 | 1.47 | NaN | 1.90 | NaN | NaN | NaN | 1.76 | NaN | NaN |
| Q9BRD0 | BUD13 homolog | BUD13 | 325 | S | 1 | 115.6 | 0.89 | 0.79 | NaN | 0.86 | 0.78 | 0.89 | 1.17 | 0.99 | NaN |
| Q9BRG2 | SH2 domain-containing protein 3A | SH2D3A | 180 | S | 0.992 | 92.61 | NaN | NaN | 0.39 | 0.60 | NaN | NaN | 0.99 | 0.98 | NaN |
| Q9BRL6 | Serine/arginine-rich splicing factor 8 | SRSF8 | 273 | S | 1 | 71.47 | NaN | 1.18 | 1.95 | 2.55 | 2.17 | NaN | 1.75 | 1.98 | 1.59 |
| Q9BRL6 | Serine/arginine-rich splicing factor 8 | SRSF8 | 282 | S | 0.756 | 71.47 | NaN | 1.18 | 1.95 | 2.55 | 2.17 | NaN | 1.75 | 1.98 | 1.59 |
| Q9BRQ0 | Pygopus homolog 2 | PYGO2 | 40 | S | 1 | 55.11 | 0.97 | 1.25 | 0.85 | NaN | 1.21 | 1.54 | 0.48 | 0.46 | NaN |
| Q9BRS8 | La-related protein 6 | LARP6 | 447 | S | 0.829 | 57.15 | NaN | NaN | NaN | NaN | NaN | NaN | 1.48 | 1.76 | 1.74 |
| Q9BSJ8 | Extended synaptotagmin-1 | ESYT1 | 1034 | S | 1 | 107.1 | NaN | NaN | NaN | 0.95 | 0.92 | NaN | 1.07 | 1.16 | 1.41 |
| Q9BTC0 | Death-inducer obliterator 1 | DIDO1 | 1260 | S | 0.984 | 60.66 | NaN | 0.85 | 1.25 | 1.11 | 1.40 | 1.24 | 0.75 | NaN | NaN |
| Q9BTC0 | Death-inducer obliterator 1 | DIDO1 | 1456 | S | 1 | 76.35 | NaN | 1.94 | NaN | 1.70 | 1.80 | 1.76 | NaN | 1.85 | NaN |
| Q9BTC0 | Death-inducer obliterator 1 | DIDO1 | 1713 | S | 0.519 | 61.94 | NaN | NaN | NaN | 0.72 | 1.12 | NaN | NaN | 1.04 | NaN |
| Q9BTC0 | Death-inducer obliterator 1 | DIDO1 | 1714 | S | 0.831 | 82.54 | 1.11 | 1.03 | 0.86 | NaN | NaN | 0.94 | 1.24 | NaN | 1.03 |
| Q9BTU6 | Phosphatidylinositol 4-kinase type 2-alpha | PI4K2A | 44 | S | 0.536 | 66.36 | NaN | 0.53 | NaN | NaN | 0.96 | 1.49 | NaN | NaN | NaN |
| Q9BTU6 | Phosphatidylinositol 4-kinase type 2-alpha | PI4K2A | 47 | S | 1 | 178.1 | 1.39 | 1.34 | 1.71 | 1.79 | 0.60 | 0.60 | 0.99 | 0.97 | 1.06 |
| Q9BTU6 | Phosphatidylinositol 4-kinase type 2-alpha | PI4K2A | 51 | S | 1 | 178.1 | 1.39 | 1.34 | 1.35 | 1.79 | 1.01 | 1.32 | 1.30 | 1.61 | 1.28 |
| Q9BU76 | Multiple myeloma tumor-associated protein 2 | MMTAG2 | 220 | S | 0.937 | 88.78 | NaN | NaN | NaN | 0.68 | 0.60 | NaN | 0.65 | 0.71 | NaN |
| Q9BUA3 | Uncharacterized protein C11orf84 | C11orf84 | 308 | S | 1 | 134.6 | 1.27 | 1.19 | NaN | 1.11 | 1.15 | NaN | 1.30 | 1.32 | 1.25 |
| Q9BUA3 | Uncharacterized protein C11orf84 | C11orf84 | 248 | S | 1 | 106.1 | 1.20 | 0.85 | 1.07 | 0.76 | NaN | 0.95 | NaN | NaN | 1.18 |
| Q9BUA3 | Uncharacterized protein C11orf84 | C11orf84 | 251 | S | 0.97 | 106.1 | 1.20 | 2.58 | 1.07 | 0.76 | NaN | 0.95 | NaN | NaN | 1.18 |
| Q9BUH6 | Protein PAXX | C9orf142 | 148 | S | 1 | 222.6 | 1.39 | 1.29 | 1.51 | 1.42 | 1.46 | 1.55 | 1.07 | 1.10 | 0.95 |
| Q9BV36 | Melanophilin | MLPH | 266 | S | 0.872 | 200.4 | 2.63 | 1.42 | NaN | 3.10 | 2.02 | NaN | NaN | NaN | NaN |
| Q9BVG4 | Protein PBDC1 | PBDC1 | 197 | S | 1 | 132 | 0.91 | 1.06 | 0.82 | 0.94 | 0.96 | 0.90 | 0.92 | 0.95 | 0.97 |
| Q9BVV8 | Uncharacterized membrane protein C19orf24 | C19orf24 | 117 | S | 0.998 | 128.9 | 1.19 | 1.13 | 0.57 | NaN | 1.65 | 1.62 | NaN | NaN | NaN |
| Q9BVV8 | Uncharacterized membrane protein C19orf24 | C19orf24 | 120 | S | 0.999 | 128.9 | 1.19 | 1.13 | NaN | NaN | 1.65 | 1.62 | NaN | NaN | NaN |
| Q9BW61 | DET1- and DDB1-associated protein 1 | DDA1 | 95 | S | 1 | 101.1 | 0.43 | 0.38 | 0.41 | 0.55 | 0.44 | 0.44 | 0.65 | 0.77 | 0.69 |
| Q9BW71 | HIRA-interacting protein 3 | HIRIP3 | 159 | S | 1 | 46.07 | NaN | NaN | NaN | NaN | 0.90 | NaN | NaN | 0.96 | 0.93 |
| Q9BW71 | HIRA-interacting protein 3 | HIRIP3 | 160 | S | 1 | 46.07 | NaN | NaN | NaN | NaN | 0.90 | NaN | NaN | 0.96 | 0.93 |
| Q9BW71 | HIRA-interacting protein 3 | HIRIP3 | 196 | S | 1 | 289.4 | 0.59 | 0.58 | NaN | NaN | NaN | 0.62 | NaN | NaN | 0.88 |
| Q9BW85 | Coiled-coil domain-containing protein 94 | CCDC94 | 211 | S | 1 | 144.9 | 0.86 | 0.81 | 0.68 | 0.83 | 0.80 | 0.74 | 0.88 | 0.86 | 0.85 |
| Q9BW85 | Coiled-coil domain-containing protein 94 | CCDC94 | 213 | S | 1 | 144.9 | 0.86 | 0.81 | 0.68 | 0.83 | 0.80 | 0.74 | 0.88 | 0.86 | 0.85 |

| | | | | | | | | | | | | | | | |
|---|---|---|---|---|---|---|---|---|---|---|---|---|---|---|---|
| Q9BX95 | Sphingosine-1-phosphate phosphatase 1 | SGPP1 | 112 | S | 0.994 | 77.05 | 1.04 | 0.72 | NaN | NaN | NaN | NaN | 1.05 | 0.89 | NaN |
| Q9BXP5 | Serrate RNA effector molecule homolog | SRRT | 67 | S | 1 | 107 | 1.04 | 1.16 | 1.01 | 0.97 | 0.97 | 0.90 | 1.22 | 1.20 | 1.11 |
| Q9BXP5 | Serrate RNA effector molecule homolog | SRRT | 74 | S | 1 | 118.3 | 1.19 | 1.14 | 1.22 | 1.15 | 1.20 | 1.10 | 1.25 | 1.34 | 1.22 |
| Q9BXW6 | Oxysterol-binding protein-related protein 1 | OSBPL1A | 499 | S | 1 | 42.32 | 1.18 | 0.80 | NaN | NaN | NaN | NaN | NaN | NaN | NaN |
| Q9BY89 | Uncharacterized protein KIAA1671 | KIAA1671 | 1701 | S | 1 | 99.71 | 0.66 | 0.65 | NaN | 0.74 | 0.68 | NaN | 0.88 | 0.95 | NaN |
| Q9BY89 | Uncharacterized protein KIAA1671 | KIAA1671 | 1019 | S | 1 | 84.9 | 0.98 | 0.91 | 0.99 | 1.10 | 1.16 | 1.14 | 0.98 | 0.96 | 0.80 |
| Q9BY89 | Uncharacterized protein KIAA1671 | KIAA1671 | 1363 | S | 0.557 | 58.37 | NaN | NaN | NaN | 1.20 | NaN | 0.88 | 1.15 | 0.94 | 0.76 |
| Q9BY89 | Uncharacterized protein KIAA1671 | KIAA1671 | 1366 | S | 0.832 | 54.2 | 0.74 | 0.84 | NaN | NaN | 1.04 | NaN | 0.96 | 0.85 | 1.01 |
| Q9BYW2 | Histone-lysine N-methyltransferase SETD2 | SETD2 | 624 | S | 0.951 | 57.79 | NaN | 0.86 | NaN | 1.60 | NaN | 1.30 | 1.40 | NaN | 1.38 |
| Q9BYX2 | TBC1 domain family member 2A | TBC1D2 | 920 | S | 1 | 145.6 | 0.75 | 0.87 | 0.88 | 0.75 | 0.75 | 0.78 | 1.18 | 1.06 | 1.09 |
| Q9BZF1 | Oxysterol-binding protein-related protein 8 | OSBPL8 | 807 | S | 0.998 | 160.8 | 0.84 | 0.75 | 0.76 | 0.72 | 0.73 | 0.65 | 0.92 | 1.10 | 0.92 |
| Q9BZF1 | Oxysterol-binding protein-related protein 8 | OSBPL8 | 808 | S | 0.999 | 160.8 | 0.84 | 0.75 | 0.76 | 0.72 | NaN | 0.65 | 0.92 | 1.10 | 0.92 |
| Q9BZF1 | Oxysterol-binding protein-related protein 8 | OSBPL8 | 810 | S | 0.997 | 81.97 | 1.49 | 1.17 | 1.06 | NaN | 0.73 | NaN | 1.91 | NaN | NaN |
| Q9BZH6 | WD repeat-containing protein 11 | WDR11 | 208 | S | 0.857 | 143.8 | NaN | NaN | NaN | NaN | NaN | NaN | 1.04 | 0.91 | NaN |
| Q9BZQ8 | Protein Niban | FAM129A | 622 | S | 1 | 178.3 | 0.59 | 0.92 | 0.90 | 0.68 | NaN | NaN | 0.91 | 0.84 | NaN |
| Q9BZQ8 | Protein Niban | FAM129A | 926 | S | 1 | 102.6 | 1.75 | 1.65 | NaN | NaN | NaN | NaN | NaN | NaN | NaN |
| Q9BZQ8 | Protein Niban | FAM129A | 646 | S | 1 | 98.15 | 1.48 | 1.72 | NaN | NaN | 2.24 | NaN | 1.12 | NaN | NaN |
| Q9C0B5 | Palmitoyltransferase ZDHHC5 | ZDHHC5 | 529 | S | 0.831 | 66.87 | 1.61 | NaN | NaN | 1.24 | 1.30 | NaN | NaN | NaN | 1.18 |
| Q9C0C2 | 182 kDa tankyrase-1-binding protein | TNKS1BP1 | 672 | S | 1 | 195.4 | 1.26 | 1.28 | 1.22 | 1.17 | 1.06 | 1.13 | 1.48 | 1.41 | 1.37 |
| Q9C0C2 | 182 kDa tankyrase-1-binding protein | TNKS1BP1 | 1620 | S | 1 | 171.8 | 1.02 | 1.11 | 1.10 | 0.99 | 1.03 | 1.01 | 1.25 | 1.24 | 1.20 |
| Q9C0C2 | 182 kDa tankyrase-1-binding protein | TNKS1BP1 | 1621 | S | 1 | 171.8 | 1.02 | 1.11 | 1.10 | 0.99 | 1.03 | 1.01 | 1.25 | 1.24 | 1.20 |
| Q9C0C2 | 182 kDa tankyrase-1-binding protein | TNKS1BP1 | 1024 | S | 0.999 | 77.4 | NaN | 1.16 | 1.49 | NaN | NaN | NaN | 1.35 | 1.66 | NaN |
| Q9C0C2 | 182 kDa tankyrase-1-binding protein | TNKS1BP1 | 494 | S | 1 | 224.6 | 0.69 | 0.77 | 0.85 | 0.77 | 1.39 | 2.00 | 1.85 | NaN | 1.06 |
| Q9C0C2 | 182 kDa tankyrase-1-binding protein | TNKS1BP1 | 498 | S | 0.999 | 224.6 | 1.81 | 1.56 | 1.97 | 1.57 | NaN | 2.00 | 1.85 | 0.93 | 2.40 |
| Q9C0C2 | 182 kDa tankyrase-1-binding protein | TNKS1BP1 | 1666 | S | 1 | 85.29 | 0.68 | 0.69 | NaN | 0.68 | 0.92 | 1.27 | NaN | 1.22 | 1.24 |

| | | | | | | | | | | | | | | | |
|---|---|---|---|---|---|---|---|---|---|---|---|---|---|---|---|
| Q9C0C2 | 182 kDa tankyrase-1-binding protein | TNKS1BP 1 | 987 | S | 0.962 | 202 | 2.98 | 2.94 | NaN | 3.39 | NaN | NaN | NaN | NaN | NaN |
| Q9C0C2 | 182 kDa tankyrase-1-binding protein | TNKS1BP 1 | 1554 | S | 0.719 | 145.9 | NaN | 1.16 | 2.47 | NaN | NaN | NaN | NaN | NaN | NaN |
| Q9C0C2 | 182 kDa tankyrase-1-binding protein | TNKS1BP 1 | 836 | S | 1 | 148.7 | 3.52 | 3.78 | 3.96 | 2.50 | 2.46 | 2.44 | 2.32 | 2.60 | 2.67 |
| Q9C0C2 | 182 kDa tankyrase-1-binding protein | TNKS1BP 1 | 1138 | S | 0.921 | 79.28 | 1.39 | 1.40 | NaN | NaN | 2.11 | NaN | 1.06 | 1.24 | NaN |
| Q9C0C2 | 182 kDa tankyrase-1-binding protein | TNKS1BP 1 | 601 | S | 1 | 185 | 1.60 | 1.85 | NaN | 1.40 | 1.62 | 1.56 | 1.27 | 1.37 | 1.50 |
| Q9C0C9 | E2/E3 hybrid ubiquitin-protein ligase UBE2O | UBE2O | 87 | S | 1 | 180.1 | 0.58 | 0.62 | 0.63 | 0.87 | 0.85 | 0.71 | 0.79 | 0.75 | 0.81 |
| Q9C0C9 | E2/E3 hybrid ubiquitin-protein ligase UBE2O | UBE2O | 89 | S | 1 | 180.1 | 0.58 | 0.62 | 0.63 | 0.87 | 0.85 | 0.71 | 0.79 | 0.75 | 0.81 |
| Q9C0C9 | E2/E3 hybrid ubiquitin-protein ligase UBE2O | UBE2O | 836 | S | 0.919 | 103.9 | 1.34 | 0.88 | 0.72 | 1.53 | 1.52 | 1.15 | NaN | NaN | NaN |
| Q9C0C9 | E2/E3 hybrid ubiquitin-protein ligase UBE2O | UBE2O | 839 | S | 0.786 | 224.5 | 0.87 | 0.74 | 1.06 | 1.17 | NaN | 1.37 | 0.54 | 0.81 | 0.75 |
| Q9GZP8 | Immortalization up-regulated protein | IMUP | 29 | S | 1 | 91.47 | 0.99 | 1.07 | 1.08 | 1.09 | 1.13 | 1.04 | 1.06 | 1.06 | 1.13 |
| Q9H0D6 | 5'-3' exoribonuclease 2 | XRN2 | 499 | S | 1 | 186.6 | 0.72 | 0.40 | 0.72 | 0.60 | 0.55 | 0.72 | 0.80 | 0.76 | 0.92 |
| Q9H0D6 | 5'-3' exoribonuclease 2 | XRN2 | 501 | S | 1 | 217.7 | 0.72 | 0.76 | 0.79 | 0.75 | 0.78 | 0.72 | 0.90 | 0.99 | 0.92 |
| Q9H0D6 | 5'-3' exoribonuclease 2 | XRN2 | 448 | S | 1 | 87.88 | 1.00 | 1.52 | NaN | 1.75 | 1.78 | 1.78 | 0.94 | 1.11 | 1.07 |
| Q9H0G5 | Nuclear speckle splicing regulatory protein 1 | NSRP1 | 33 | S | 1 | 167 | 1.09 | 1.00 | NaN | NaN | NaN | NaN | 1.20 | NaN | 1.80 |
| Q9H1B7 | Interferon regulatory factor 2-binding protein-like | IRF2BPL | 547 | S | 1 | 90.82 | 0.78 | 0.87 | 0.78 | 0.73 | 0.66 | 0.78 | 1.37 | 1.33 | 1.43 |
| Q9H1E3 | Nuclear ubiquitous casein and cyclin-dependent kinase substrate 1 | NUCKS1 | 181 | S | 1 | 198.5 | 2.75 | 2.68 | 2.59 | 2.88 | 3.22 | 2.72 | 1.16 | 1.14 | 1.16 |
| Q9H1E3 | Nuclear ubiquitous casein and cyclin-dependent kinase substrate 1 | NUCKS1 | 73 | S | 1 | 89.68 | 1.17 | 1.07 | NaN | NaN | NaN | 1.01 | NaN | NaN | NaN |
| Q9H1E3 | Nuclear ubiquitous casein and cyclin-dependent kinase substrate 1 | NUCKS1 | 75 | S | 1 | 163.1 | 0.29 | 0.34 | 1.03 | 1.01 | 1.02 | 1.11 | 0.59 | 0.53 | 1.06 |
| Q9H1E3 | Nuclear ubiquitous casein and cyclin-dependent kinase substrate 1 | NUCKS1 | 79 | S | 1 | 170.3 | 0.32 | 0.30 | 0.31 | 1.01 | 1.02 | 1.05 | 0.49 | 0.46 | 1.06 |
| Q9H1E3 | Nuclear ubiquitous casein and cyclin-dependent kinase substrate 1 | NUCKS1 | 214 | S | 1 | 271.5 | 0.74 | 0.75 | 0.69 | 0.75 | 0.76 | 0.72 | 0.90 | 0.91 | 0.89 |
| Q9H1E3 | Nuclear ubiquitous casein and cyclin-dependent kinase substrate 1 | NUCKS1 | 204 | S | 1 | 144.7 | 0.92 | 1.23 | 1.27 | 1.07 | 0.89 | 1.17 | 0.96 | 1.03 | 1.37 |

| | | | | | | | | | | | | | | | |
|---|---|---|---|---|---|---|---|---|---|---|---|---|---|---|---|
| Q9H1E3 | Nuclear ubiquitous casein and cyclin-dependent kinase substrate 1 | NUCKS1 | 113 | S | 1 | 162.2 | NaN | NaN | 0.29 | 0.36 | 0.31 | 0.27 | 0.22 | 0.26 | 0.25 |
| Q9H1E3 | Nuclear ubiquitous casein and cyclin-dependent kinase substrate 1 | NUCKS1 | 19 | S | 1 | 349 | 1.19 | 1.18 | 1.15 | 1.10 | 1.08 | 1.08 | 1.16 | 1.18 | 1.19 |
| Q9H1E3 | Nuclear ubiquitous casein and cyclin-dependent kinase substrate 1 | NUCKS1 | 54 | S | 0.998 | 159 | 0.92 | 0.65 | 0.99 | 0.69 | 0.81 | 1.02 | 0.57 | 0.98 | 0.85 |
| Q9H1E3 | Nuclear ubiquitous casein and cyclin-dependent kinase substrate 1 | NUCKS1 | 58 | S | 1 | 187 | 0.68 | 0.85 | 0.74 | 0.88 | 0.70 | 0.70 | 0.98 | 0.62 | 0.84 |
| Q9H1E3 | Nuclear ubiquitous casein and cyclin-dependent kinase substrate 1 | NUCKS1 | 61 | S | 1 | 228.6 | 0.63 | 0.67 | 0.62 | 0.70 | 0.71 | 0.87 | 0.83 | 0.94 | 0.99 |
| Q9H1E3 | Nuclear ubiquitous casein and cyclin-dependent kinase substrate 1 | NUCKS1 | 50 | S | 0.547 | 63.48 | 0.76 | 0.85 | NaN | NaN | 0.97 | NaN | NaN | NaN | NaN |
| Q9H1E3 | Nuclear ubiquitous casein and cyclin-dependent kinase substrate 1 | NUCKS1 | 223 | S | 1 | 150.2 | 0.32 | 0.35 | 0.36 | 0.39 | 0.38 | 0.39 | 0.47 | 0.47 | 0.49 |
| Q9H246 | Uncharacterized protein C1orf21 | C1orf21 | 115 | S | 0.999 | 65.09 | NaN | NaN | NaN | 1.19 | 1.01 | 0.93 | 1.13 | 1.05 | NaN |
| Q9H307 | Pinin | PNN | 375 | S | 0.954 | 104.3 | NaN | NaN | 0.87 | 1.32 | 1.10 | 1.18 | 0.54 | 0.55 | 0.48 |
| Q9H307 | Pinin | PNN | 381 | S | 1 | 224.6 | 0.88 | 0.89 | 0.75 | 1.19 | 1.18 | 0.99 | 0.57 | 0.56 | 0.55 |
| Q9H307 | Pinin | PNN | 100 | S | 1 | 209.1 | 0.87 | 0.89 | 0.96 | 0.93 | 0.95 | 0.92 | 0.95 | 0.91 | 0.96 |
| Q9H330 | Transmembrane protein 245 | TMEM245 | 332 | S | 0.638 | 121.1 | NaN | NaN | NaN | NaN | 0.99 | NaN | 1.25 | 1.26 | NaN |
| Q9H3N1 | Thioredoxin-related transmembrane protein 1 | TMX1 | 247 | S | 1 | 302 | 1.15 | 1.07 | 1.10 | 0.95 | 0.91 | 1.05 | 1.05 | 0.99 | 1.15 |
| Q9H3Q1 | Cdc42 effector protein 4 | CDC42EP 4 | 141 | S | 0.733 | 84.31 | 0.75 | NaN | NaN | 0.89 | NaN | 0.86 | NaN | NaN | NaN |
| Q9H3Q1 | Cdc42 effector protein 4 | CDC42EP 4 | 142 | S | 0.788 | 91.96 | NaN | 0.78 | 0.81 | NaN | 0.89 | NaN | 0.51 | 0.53 | 0.52 |
| Q9H3Z4 | DnaJ homolog subfamily C member 5 | DNAJC5 | 10 | S | 0.999 | 429.1 | 1.18 | 1.24 | 1.20 | 0.99 | 0.98 | 0.99 | 1.01 | 0.89 | 1.01 |
| Q9H4L5 | Oxysterol-binding protein-related protein 3 | OSBPL3 | 437 | S | 1 | 159.5 | 1.52 | 1.14 | NaN | NaN | NaN | 6.56 | NaN | 1.20 | NaN |
| Q9H4L5 | Oxysterol-binding protein-related protein 3 | OSBPL3 | 410 | S | 1 | 117 | 0.93 | 1.01 | 0.96 | 1.55 | 1.37 | 1.39 | 0.85 | 0.87 | 1.06 |
| Q9H4L5 | Oxysterol-binding protein-related protein 3 | OSBPL3 | 304 | S | 0.959 | 127.6 | 1.13 | 1.05 | NaN | NaN | 1.02 | NaN | NaN | NaN | NaN |
| Q9H4L5 | Oxysterol-binding protein-related protein 3 | OSBPL3 | 34 | S | 1 | 94.72 | 1.57 | 1.90 | 1.67 | 1.41 | 1.28 | NaN | 1.37 | 1.56 | 1.56 |
| Q9H4L5 | Oxysterol-binding protein-related protein 3 | OSBPL3 | 372 | S | 0.963 | 322.4 | 0.70 | 0.69 | 0.67 | 1.31 | 1.33 | 1.37 | 0.57 | 0.68 | 0.69 |
| Q9H4L5 | Oxysterol-binding protein-related protein 3 | OSBPL3 | 262 | S | 0.706 | 122.4 | NaN | 1.17 | 0.98 | 1.79 | NaN | 1.60 | 0.62 | NaN | 1.23 |

| | | | | | | | | | | | | | | | |
|---|---|---|---|---|---|---|---|---|---|---|---|---|---|---|---|
| Q9H4L5 | Oxysterol-binding protein-related protein 3 | OSBPL3 | 265 | S | 0.995 | 142.5 | 1.16 | 1.13 | NaN | 1.27 | 1.85 | NaN | NaN | 0.65 | 0.69 |
| Q9H501 | ESF1 homolog | ESF1 | 657 | S | 1 | 140.6 | 0.86 | 0.94 | 0.99 | 0.98 | 0.97 | 1.13 | 0.80 | 0.82 | 0.78 |
| Q9H501 | ESF1 homolog | ESF1 | 663 | S | 1 | 134.6 | 1.24 | 0.80 | NaN | 1.11 | 1.26 | 1.18 | 1.41 | 1.65 | 1.31 |
| Q9H501 | ESF1 homolog | ESF1 | 153 | S | 1 | 110.4 | 1.02 | 0.96 | 0.90 | 1.15 | 1.26 | 1.17 | 0.48 | 0.70 | 0.53 |
| Q9H501 | ESF1 homolog | ESF1 | 198 | S | 0.989 | 68.42 | 0.77 | 1.23 | NaN | 1.88 | NaN | NaN | NaN | NaN | NaN |
| Q9H6F5 | Coiled-coil domain-containing protein 86 | CCDC86 | 58 | S | 1 | 93.65 | 1.21 | 1.46 | 1.36 | 1.58 | 1.58 | 1.61 | 0.68 | 0.70 | 0.70 |
| Q9H6F5 | Coiled-coil domain-containing protein 86 | CCDC86 | 66 | S | 0.668 | 64.04 | 0.74 | 0.65 | 0.62 | 0.77 | 0.74 | NaN | NaN | NaN | 0.80 |
| Q9H6F5 | Coiled-coil domain-containing protein 86 | CCDC86 | 69 | S | 1 | 111.6 | 1.19 | 0.65 | 0.62 | 0.77 | 0.74 | 0.83 | 0.98 | NaN | 0.71 |
| Q9H6F5 | Coiled-coil domain-containing protein 86 | CCDC86 | 47 | S | 1 | 319.2 | 1.12 | 1.12 | 1.11 | 1.14 | 1.15 | 1.11 | 0.95 | 0.99 | 0.95 |
| Q9H6F5 | Coiled-coil domain-containing protein 86 | CCDC86 | 18 | S | 0.999 | 88.68 | 1.55 | 1.43 | 1.78 | NaN | 1.21 | 1.46 | 1.62 | 1.39 | 1.35 |
| Q9H6F5 | Coiled-coil domain-containing protein 86 | CCDC86 | 91 | S | 1 | 331.6 | 1.04 | 1.07 | 1.14 | 1.15 | 1.08 | 1.07 | 1.07 | 0.94 | 1.13 |
| Q9H6F5 | Coiled-coil domain-containing protein 86 | CCDC86 | 102 | S | 1 | 110.2 | 0.86 | NaN | 0.86 | NaN | 1.59 | NaN | 1.08 | 1.03 | NaN |
| Q9H6F5 | Coiled-coil domain-containing protein 86 | CCDC86 | 110 | S | 0.97 | 94.15 | 0.80 | NaN | 0.86 | NaN | 1.59 | 0.92 | 1.08 | 1.03 | NaN |
| Q9H6F5 | Coiled-coil domain-containing protein 86 | CCDC86 | 113 | S | 0.998 | 70.63 | NaN | 1.00 | 0.86 | NaN | 0.92 | NaN | 1.08 | 1.08 | 1.08 |
| Q9H6X2 | Anthrax toxin receptor 1 | ANTXR1 | 362 | S | 1 | 173.6 | 0.85 | NaN | 0.86 | 0.89 | 0.89 | 0.87 | 1.03 | 0.91 | 1.13 |
| Q9H7D0 | Dedicator of cytokinesis protein 5 | DOCK5 | 1789 | S | 0.985 | 47.43 | 1.53 | NaN | 1.12 | NaN | 1.02 | NaN | NaN | 1.08 | NaN |
| Q9H7D0 | Dedicator of cytokinesis protein 5 | DOCK5 | 1756 | S | 0.93 | 103.4 | NaN | NaN | 1.23 | 1.86 | NaN | 1.26 | NaN | NaN | NaN |
| Q9H7L9 | Sin3 histone deacetylase corepressor complex component SDS3 | SUDS3 | 234 | S | 0.985 | 125.4 | 0.83 | 0.90 | NaN | 0.65 | 0.79 | NaN | NaN | 0.89 | 1.04 |
| Q9H7N4 | Splicing factor, arginine/serine-rich 19 | SCAF1 | 734 | S | 1 | 116.8 | 1.41 | 1.63 | 1.20 | 1.13 | 1.03 | 1.44 | 1.15 | 1.14 | 1.71 |
| Q9H7N4 | Splicing factor, arginine/serine-rich 19 | SCAF1 | 738 | S | 1 | 116.8 | 1.41 | 1.63 | 1.20 | 1.13 | 1.03 | 1.44 | 1.15 | 1.14 | 1.71 |
| Q9H7N4 | Splicing factor, arginine/serine-rich 19 | SCAF1 | 239 | S | 1 | 156 | 0.82 | 0.84 | 0.85 | 0.82 | 0.87 | 0.93 | 1.02 | 0.86 | 0.78 |
| Q9H7N4 | Splicing factor, arginine/serine-rich 19 | SCAF1 | 963 | S | 0.982 | 175.2 | 1.09 | 0.88 | NaN | NaN | 1.13 | 1.13 | NaN | 0.87 | NaN |
| Q9H7N4 | Splicing factor, arginine/serine-rich 19 | SCAF1 | 724 | S | 0.937 | 97.75 | NaN | 0.57 | 0.61 | 0.53 | 0.85 | NaN | 0.70 | 0.61 | 0.64 |
| Q9H7N4 | Splicing factor, arginine/serine-rich 19 | SCAF1 | 725 | S | 0.894 | 97.75 | NaN | 0.51 | 0.61 | 0.30 | NaN | NaN | 0.70 | 0.52 | 0.64 |
| Q9H7N4 | Splicing factor, arginine/serine-rich 19 | SCAF1 | 498 | S | 1 | 136.3 | 1.09 | 1.20 | 1.21 | 1.16 | 1.20 | 1.07 | 1.36 | 1.30 | 1.18 |

| | | | | | | | | | | | | | | | |
|---|---|---|---|---|---|---|---|---|---|---|---|---|---|---|---|
| Q9H7N4 | Splicing factor, arginine/serine-rich 19 | SCAF1 | 500 | S | 1 | 136.3 | 1.09 | 1.20 | 1.21 | 1.16 | 1.20 | 1.07 | 1.36 | 1.30 | 1.18 |
| Q9H7N4 | Splicing factor, arginine/serine-rich 19 | SCAF1 | 612 | S | 1 | 51.76 | NaN | NaN | NaN | 0.91 | 0.90 | NaN | 0.92 | NaN | 0.88 |
| Q9H7N4 | Splicing factor, arginine/serine-rich 19 | SCAF1 | 614 | S | 1 | 51.76 | NaN | NaN | NaN | 0.91 | 0.90 | NaN | 0.92 | NaN | 0.88 |
| Q9H8G2 | Caspase activity and apoptosis inhibitor 1 | CAAP1 | 203 | S | 1 | 97.77 | 0.59 | 0.64 | 0.63 | 0.98 | 0.94 | 0.91 | 0.93 | 1.13 | 0.38 |
| Q9H8G2 | Caspase activity and apoptosis inhibitor 1 | CAAP1 | 312 | S | 0.909 | 88.18 | 1.05 | 1.26 | NaN | NaN | 1.61 | 1.69 | NaN | 1.39 | 1.12 |
| Q9H8M2 | Bromodomain-containing protein 9 | BRD9 | 588 | S | 1 | 143.6 | 0.88 | 0.66 | NaN | 0.99 | 0.78 | 0.66 | 1.04 | 0.83 | 0.79 |
| Q9H8Y8 | Golgi reassembly-stacking protein 2 | GORASP 2 | 451 | S | 1 | 122.7 | 0.72 | 0.88 | 0.86 | 1.24 | 1.18 | 1.39 | 1.00 | 0.99 | 0.99 |
| Q9HA65 | TBC1 domain family member | TBC1D17 | 602 | S | 0.997 | 161.7 | 1.31 | 0.85 | 2.02 | NaN | NaN | NaN | 1.03 | 1.57 | 1.32 |
| Q9HA65 | TBC1 domain family member | TBC1D17 | 604 | S | 0.928 | 161.7 | 1.31 | 0.85 | 2.02 | NaN | NaN | NaN | 1.03 | 1.57 | 1.32 |
| Q9HA65 | TBC1 domain family member | TBC1D17 | 613 | S | 0.88 | 127.3 | NaN | 0.85 | 2.02 | NaN | NaN | NaN | 0.65 | 1.42 | NaN |
| Q9HCD6 | Protein TANC2 | TANC2 | 169 | S | 0.986 | 228.5 | 0.87 | 1.19 | NaN | 1.34 | 1.41 | 1.25 | 1.29 | 1.13 | NaN |
| Q9HCD6 | Protein TANC2 | TANC2 | 1579 | S | 0.93 | 75.33 | 1.27 | 1.51 | NaN | 1.47 | 1.20 | NaN | NaN | 1.51 | 1.37 |
| Q9HCE5 | N6-adenosine-methyltransferase subunit METTL14 | METTL14 | 399 | S | 1 | 77.59 | 0.56 | NaN | 0.57 | 0.70 | NaN | 0.69 | 0.88 | NaN | 0.84 |
| Q9HCN4 | GPN-loop GTPase 1 | GPN1 | 314 | S | 0.996 | 90.56 | 1.31 | 1.56 | 1.13 | 1.40 | 1.29 | 1.37 | 0.89 | 0.90 | NaN |
| Q9NQC3 | Reticulon-4 | RTN4 | 181 | S | 0.807 | 114.5 | 0.48 | 1.07 | 0.94 | 1.01 | 0.89 | 0.98 | 1.19 | 0.80 | NaN |
| Q9NQC3 | Reticulon-4 | RTN4 | 182 | S | 0.808 | 115.1 | 1.00 | 1.07 | 0.94 | 1.01 | 0.89 | 0.98 | 1.14 | 0.80 | NaN |
| Q9NQC3 | Reticulon-4 | RTN4 | 184 | S | 0.837 | 115.1 | 1.16 | 1.28 | 0.94 | 1.01 | 0.89 | 0.98 | 1.00 | 0.99 | NaN |
| Q9NQG5 | Regulation of nuclear pre-mRNA domain-containing protein 1B | RPRD1B | 134 | S | 0.906 | 73.98 | 0.78 | 0.53 | 0.74 | 0.53 | NaN | NaN | NaN | NaN | 0.55 |
| Q9NQG5 | Regulation of nuclear pre-mRNA domain-containing protein 1B | RPRD1B | 166 | S | 1 | 225.5 | 1.00 | 1.02 | 1.00 | 1.01 | 1.18 | 0.94 | 1.46 | 1.13 | 0.94 |
| Q9NQP4 | Prefoldin subunit 4 | PFDN4 | 125 | S | 1 | 53.14 | 1.94 | 2.04 | NaN | NaN | NaN | NaN | NaN | NaN | NaN |
| Q9NQS7 | Inner centromere protein | INCENP | 197 | S | 0.973 | 150.5 | 1.18 | NaN | NaN | 2.16 | 2.15 | 1.83 | NaN | NaN | NaN |
| Q9NQS7 | Inner centromere protein | INCENP | 214 | S | 0.783 | 150.5 | 1.18 | NaN | NaN | 2.16 | 2.15 | 1.83 | NaN | NaN | NaN |
| Q9NQW6 | Actin-binding protein anillin | ANLN | 182 | S | 1 | 209.2 | 0.91 | 1.01 | 0.98 | 1.84 | 1.97 | 2.00 | 0.63 | 0.65 | 0.70 |
| Q9NQW6 | Actin-binding protein anillin | ANLN | 54 | S | 1 | 83.31 | 1.27 | 1.57 | 1.26 | 2.75 | 2.78 | NaN | 0.82 | 1.01 | NaN |
| Q9NQW6 | Actin-binding protein anillin | ANLN | 517 | S | 0.815 | 149.5 | 0.81 | NaN | 1.05 | 2.25 | 2.39 | NaN | NaN | 0.92 | 0.80 |
| Q9NQW6 | Actin-binding protein anillin | ANLN | 518 | S | 0.992 | 260.1 | 0.70 | 0.65 | 0.86 | 2.04 | 2.18 | 2.26 | 1.04 | 0.86 | NaN |
| Q9NQW6 | Actin-binding protein anillin | ANLN | 792 | S | 0.999 | 98.16 | 0.93 | 0.84 | NaN | 2.01 | 1.94 | 2.16 | 0.27 | 0.30 | NaN |
| Q9NQW6 | Actin-binding protein anillin | ANLN | 323 | S | 1 | 77.64 | NaN | NaN | 0.79 | NaN | NaN | 1.80 | NaN | 0.65 | 0.64 |
| Q9NQW6 | Actin-binding protein anillin | ANLN | 65 | S | 0.994 | 102.6 | 0.73 | 0.67 | 0.68 | 1.64 | 1.67 | 1.58 | 0.58 | 0.58 | 0.56 |
| Q9NQZ2 | Something about silencing protein 10 | UTP3 | 365 | S | 1 | 162 | 1.50 | 1.23 | 2.13 | NaN | 1.33 | NaN | 1.69 | 2.14 | NaN |
| Q9NQZ2 | Something about silencing protein 10 | UTP3 | 368 | S | 1 | 162 | 1.50 | 1.23 | 2.13 | NaN | 1.33 | NaN | 1.69 | 2.14 | NaN |

| | | | | | | | | | | | | | | |
|---|---|---|---|---|---|---|---|---|---|---|---|---|---|---|
| Q9NR19 | Acetyl-coenzyme A synthetase, cytoplasmic | ACSS2 | 267 | S | 0.995 | 167.9 | NaN | 0.49 | NaN | 0.77 | NaN | 0.81 | NaN | 0.51 | 0.60 |
| Q9NR19 | Acetyl-coenzyme A synthetase, cytoplasmic | ACSS2 | 30 | S | 1 | 135.2 | 0.55 | 0.57 | 0.59 | 0.66 | 0.68 | 0.68 | 0.91 | 0.92 | 0.93 |
| Q9NR30 | Nucleolar RNA helicase 2 | DDX21 | 71 | S | 1 | 64.41 | 1.27 | 1.24 | NaN | NaN | NaN | NaN | NaN | NaN | NaN |
| Q9NR30 | Nucleolar RNA helicase 2 | DDX21 | 89 | S | 1 | 103.2 | 0.61 | 0.67 | 0.77 | 0.73 | 1.05 | 1.02 | 0.67 | 0.61 | 0.65 |
| Q9NR30 | Nucleolar RNA helicase 2 | DDX21 | 121 | S | 1 | 151.4 | 1.06 | 0.99 | 0.78 | 1.12 | 0.91 | 1.12 | 0.74 | 0.67 | 0.75 |
| Q9NRF2 | SH2B adapter protein 1 | SH2B1 | 125 | S | 0.859 | 100.7 | 0.95 | 0.97 | 0.68 | NaN | NaN | NaN | NaN | NaN | NaN |
| Q9NRX5 | Serine incorporator 1 | SERINC1 | 364 | S | 1 | 83.77 | 1.64 | 2.17 | 1.40 | 1.35 | 1.34 | NaN | 1.15 | 0.85 | NaN |
| Q9NRY4 | Rho GTPase-activating protein 35 | ARHGAP 35 | 975 | S | 1 | 144.4 | 0.97 | NaN | 0.87 | 0.79 | 1.33 | 0.84 | NaN | NaN | NaN |
| Q9NRY4 | Rho GTPase-activating protein 35 | ARHGAP 35 | 1179 | S | 1 | 254.6 | 0.93 | 0.92 | 0.84 | 0.94 | 0.99 | 0.85 | 1.00 | 0.98 | 1.02 |
| Q9NSV4 | Protein diaphanous homolog 3 | DIAPH3 | 1093 | S | 0.984 | 54.97 | 0.87 | NaN | 0.75 | 1.18 | 1.26 | NaN | 0.91 | 0.90 | 0.96 |
| Q9NTI5 | Sister chromatid cohesion protein PDS5 homolog B | PDS5B | 1358 | S | 1 | 250.4 | NaN | NaN | NaN | 1.78 | NaN | NaN | 2.07 | NaN | 2.75 |
| Q9NTI5 | Sister chromatid cohesion protein PDS5 homolog B | PDS5B | 1283 | S | 1 | 124.8 | 0.94 | 0.86 | 0.81 | 1.29 | 1.21 | 1.15 | 0.74 | 0.84 | 0.76 |
| Q9NTI5 | Sister chromatid cohesion protein PDS5 homolog B | PDS5B | 1166 | S | 0.975 | 401.3 | 0.71 | 0.56 | 0.84 | 0.94 | 1.10 | 0.94 | 0.50 | 0.50 | NaN |
| Q9NTZ6 | RNA-binding protein 12 | RBM12 | 411 | S | 0.968 | 61.42 | NaN | NaN | NaN | NaN | NaN | NaN | 0.90 | NaN | 0.99 |
| Q9NTZ6 | RNA-binding protein 12 | RBM12 | 413 | S | 0.93 | 54.65 | 1.10 | NaN | NaN | 0.98 | NaN | 0.99 | NaN | NaN | NaN |
| Q9NVD7 | Alpha-parvin | PARVA | 8 | S | 0.711 | 68.88 | NaN | NaN | NaN | NaN | 1.08 | 1.44 | NaN | 0.98 | NaN |
| Q9NVD7 | Alpha-parvin | PARVA | 14 | S | 0.997 | 129.5 | 1.71 | 1.32 | 1.29 | 1.34 | 1.30 | 1.35 | 1.48 | 1.60 | 1.61 |
| Q9NVD7 | Alpha-parvin | PARVA | 19 | S | 0.983 | 113.7 | 0.60 | 0.61 | 0.61 | 0.48 | 0.45 | 1.28 | 0.90 | 0.97 | 0.90 |
| Q9NW82 | WD repeat-containing protein 70 | WDR70 | 638 | S | 1 | 161.3 | 0.65 | 0.52 | NaN | 1.38 | 1.17 | NaN | NaN | 2.72 | 1.90 |
| Q9NWB6 | Arginine and glutamate-rich protein 1 | ARGLU1 | 76 | S | 0.709 | 95.07 | NaN | NaN | NaN | NaN | 0.84 | NaN | 1.10 | 0.91 | NaN |
| Q9NWB6 | Arginine and glutamate-rich protein 1 | ARGLU1 | 77 | S | 0.999 | 155.3 | 0.93 | 0.88 | 0.85 | 0.78 | 0.77 | 0.76 | 0.86 | 0.90 | 0.91 |
| Q9NWH9 | SAFB-like transcription modulator | SLTM | 289 | S | 1 | 218.4 | 0.64 | 0.64 | 0.65 | 0.71 | 0.70 | 0.70 | 0.68 | 0.71 | 0.71 |
| Q9NWZ5 | Uridine-cytidine kinase-like 1 | UCKL1 | 56 | S | 0.982 | 91.59 | 1.31 | 1.28 | 1.30 | 1.81 | 1.97 | NaN | NaN | 0.93 | NaN |
| Q9NWZ5 | Uridine-cytidine kinase-like 1 | UCKL1 | 539 | S | 1 | 126.2 | 0.52 | 0.74 | NaN | NaN | NaN | NaN | 0.90 | NaN | 1.13 |
| Q9NXC5 | WD repeat-containing protein mio | MIOS | 766 | S | 0.888 | 125.3 | NaN | NaN | NaN | 0.69 | NaN | NaN | 0.63 | 0.69 | 0.80 |
| Q9NXE8 | Pre-mRNA-splicing factor CWC25 homolog | CWC25 | 218 | S | 0.62 | 68.46 | NaN | NaN | NaN | 0.99 | 0.96 | NaN | 0.68 | 0.57 | NaN |
| Q9NY61 | Protein AATF | AATF | 316 | S | 1 | 131.1 | 0.70 | 0.74 | 0.72 | 0.80 | 0.80 | 0.74 | 0.72 | 0.87 | 0.79 |
| Q9NY61 | Protein AATF | AATF | 320 | S | 1 | 141.7 | 0.58 | 0.63 | 0.86 | 0.85 | 0.59 | 0.55 | 1.12 | 1.08 | 0.79 |
| Q9NY61 | Protein AATF | AATF | 321 | S | 1 | 141.7 | 0.58 | 0.63 | 0.86 | 0.85 | 0.59 | 0.55 | 1.12 | 1.08 | 0.79 |
| Q9NYQ8 | Protocadherin Fat 2 | FAT2 | 1873 | S | 0.971 | 43.8 | NaN | NaN | NaN | 0.89 | 0.93 | 0.95 | 0.40 | 0.46 | NaN |
| Q9NZ63 | Uncharacterized protein C9orf78 | C9orf78 | 261 | S | 1 | 102.5 | 1.19 | 1.07 | 1.08 | NaN | NaN | 1.08 | NaN | 1.10 | 1.19 |
| Q9NZL4 | Hsp70-binding protein 1 | HSPBP1 | 351 | S | 0.803 | 154.5 | NaN | 1.53 | 1.40 | NaN | NaN | NaN | NaN | NaN | NaN |
| Q9NZM1 | Myoferlin | MYOF | 1915 | S | 0.998 | 48.39 | 0.65 | 0.65 | NaN | NaN | NaN | 0.85 | NaN | 1.15 | 0.87 |

| | | | | | | | | | | | | | | | |
|---|---|---|---|---|---|---|---|---|---|---|---|---|---|---|---|
| Q9NZN4 | EH domain-containing protein | EHD2 | 438 | S | 1 | 288.3 | 0.44 | 0.46 | 0.49 | 0.64 | 0.63 | 0.62 | 0.58 | 0.57 | 0.58 |
| Q9NZN4 | EH domain-containing protein | EHD2 | 468 | S | 0.85 | 52.47 | NaN | NaN | NaN | NaN | 0.87 | 0.74 | 0.62 | NaN | NaN |
| Q9NZN4 | EH domain-containing protein | EHD2 | 470 | S | 0.575 | 78.76 | 1.31 | NaN | NaN | 0.79 | NaN | 0.74 | 0.62 | 0.65 | 0.71 |
| Q9NZQ7 | Programmed cell death 1 ligand 1 | CD274 | 283 | S | 0.976 | 58.57 | NaN | NaN | 2.48 | NaN | NaN | NaN | 1.86 | NaN | 1.82 |
| Q9P0K7 | Ankycorbin | RAI14 | 327 | S | 0.971 | 112.4 | 1.36 | 1.34 | NaN | NaN | NaN | NaN | NaN | NaN | NaN |
| Q9P0K7 | Ankycorbin | RAI14 | 281 | S | 0.996 | 105.3 | 1.32 | NaN | 1.75 | 1.57 | 1.33 | 1.30 | 1.10 | 1.09 | NaN |
| Q9P0K7 | Ankycorbin | RAI14 | 412 | S | 0.742 | 60.36 | NaN | 1.43 | NaN | NaN | 1.34 | 1.38 | 1.47 | 1.44 | 1.32 |
| Q9P206 | Uncharacterized protein KIAA1522 | KIAA1522 | 669 | S | 1 | 88 | 1.22 | 0.98 | 0.68 | 0.65 | 0.70 | 0.62 | 1.02 | 1.02 | 1.01 |
| Q9P206 | Uncharacterized protein KIAA1522 | KIAA1522 | 673 | S | 0.997 | 88 | 1.22 | 0.98 | 0.68 | 0.65 | 0.70 | 0.62 | 1.02 | 1.02 | 1.01 |
| Q9P206 | Uncharacterized protein KIAA1522 | KIAA1522 | 545 | S | 1 | 104.2 | NaN | 2.23 | 2.23 | NaN | NaN | NaN | 1.37 | 2.11 | 2.33 |
| Q9P2B4 | CTTNBP2 N-terminal-like protein | CTTNBP2 NL | 488 | S | 1 | 113.4 | 0.76 | 0.73 | 0.64 | 0.63 | 0.71 | 0.57 | 0.61 | 0.69 | 0.69 |
| Q9P2B4 | CTTNBP2 N-terminal-like protein | CTTNBP2 NL | 481 | S | 0.889 | 85.85 | 1.01 | NaN | 0.78 | NaN | 0.49 | NaN | 1.07 | 0.88 | 0.89 |
| Q9P2B4 | CTTNBP2 N-terminal-like protein | CTTNBP2 NL | 568 | S | 0.939 | 129.3 | 0.93 | 0.94 | NaN | 0.85 | NaN | 0.71 | 1.51 | 1.63 | 1.46 |
| Q9P2C4 | Transmembrane protein 181 | TMEM181 | 580 | S | 0.992 | 98.99 | NaN | NaN | NaN | 0.96 | NaN | 1.42 | NaN | NaN | NaN |
| Q9P2E9 | Ribosome-binding protein 1 | RRBP1 | 615 | S | 1 | 168.7 | 0.59 | 0.66 | 0.74 | 1.08 | 1.13 | 1.19 | 0.76 | 0.66 | 0.64 |
| Q9P2E9 | Ribosome-binding protein 1 | RRBP1 | 1276 | S | 0.726 | 111.2 | 0.99 | 0.95 | 0.80 | 0.93 | 0.99 | NaN | 0.70 | 0.60 | 0.47 |
| Q9P2E9 | Ribosome-binding protein 1 | RRBP1 | 1277 | S | 0.779 | 111.2 | NaN | NaN | NaN | 0.93 | 0.95 | NaN | NaN | 0.68 | 0.47 |
| Q9P2G1 | Ankyrin repeat and IBR domain-containing protein 1 | ANKIB1 | 737 | S | 1 | 92.04 | 0.62 | 0.87 | NaN | 0.36 | NaN | NaN | NaN | 0.79 | NaN |
| Q9P2I0 | Cleavage and polyadenylation specificity factor subunit 2 | CPSF2 | 419 | S | 0.912 | 110.8 | 0.42 | 0.19 | 3.75 | 0.63 | NaN | NaN | 2.02 | NaN | NaN |
| Q9P2I0 | Cleavage and polyadenylation specificity factor subunit 2 | CPSF2 | 420 | S | 0.912 | 110.8 | 0.42 | 0.19 | 3.75 | 0.63 | NaN | NaN | 2.02 | NaN | NaN |
| Q9P2I0 | Cleavage and polyadenylation specificity factor subunit 2 | CPSF2 | 423 | S | 0.734 | 93.73 | 0.42 | NaN | 3.75 | NaN | NaN | NaN | 2.02 | NaN | NaN |
| Q9P2Q2 | FERM domain-containing protein 4A | FRMD4A | 694 | S | 0.798 | 79.07 | NaN | NaN | NaN | NaN | 0.74 | 1.52 | NaN | 0.86 | NaN |
| Q9UBB9 | Tuftelin-interacting protein 11 | TFIP11 | 98 | S | 1 | 165.5 | 0.79 | 0.92 | 0.95 | 1.02 | 0.87 | 0.84 | 1.14 | 1.29 | 1.17 |
| Q9UBB9 | Tuftelin-interacting protein 11 | TFIP11 | 210 | S | 1 | 144.7 | 0.80 | 0.82 | 1.02 | 1.01 | 0.92 | 1.39 | 0.69 | 0.83 | 0.71 |
| Q9UDT6 | CAP-Gly domain-containing linker protein 2 | CLIP2 | 53 | S | 0.538 | 83.32 | NaN | NaN | 1.25 | NaN | NaN | 2.10 | 0.61 | NaN | 0.78 |
| Q9UER7 | Death domain-associated protein 6 | DAXX | 495 | S | 1 | 168.4 | NaN | 0.51 | 0.71 | 0.73 | 0.72 | 0.98 | 0.63 | 0.83 | 0.62 |
| Q9UEY8 | Gamma-adducin | ADD3 | 677 | S | 0.957 | 132.8 | 0.58 | 0.58 | NaN | 0.57 | 0.66 | NaN | 0.46 | 0.87 | NaN |
| Q9UEY8 | Gamma-adducin | ADD3 | 679 | S | 0.926 | 99.93 | 0.58 | 0.58 | 0.48 | 0.57 | 0.66 | NaN | 0.46 | 0.91 | NaN |
| Q9UEY8 | Gamma-adducin | ADD3 | 681 | S | 0.689 | 87.67 | 0.50 | 0.52 | NaN | NaN | 0.66 | NaN | NaN | NaN | NaN |
| Q9UGU5 | HMG domain-containing protein 4 | HMGXB4 | 497 | S | 0.997 | 52.25 | NaN | NaN | 0.95 | 1.07 | 1.38 | NaN | 1.09 | NaN | NaN |
| Q9UHB6 | LIM domain and actin-binding protein 1 | LIMA1 | 373 | S | 0.912 | 172.1 | 0.67 | NaN | NaN | 0.99 | NaN | 1.08 | 0.81 | NaN | 0.89 |

| | | | | | | | | | | | | | | |
|---|---|---|---|---|---|---|---|---|---|---|---|---|---|---|
| Q9UHB6 | LIM domain and actin-binding protein 1 | LIMA1 | 374 | S | 0.983 | 168.8 | NaN | 0.67 | 0.73 | NaN | 0.99 | NaN | NaN | 0.80 | NaN |
| Q9UHB6 | LIM domain and actin-binding protein 1 | LIMA1 | 686 | S | 0.987 | 75.77 | NaN | NaN | NaN | 0.11 | 0.14 | 0.14 | 0.09 | 0.18 | 0.18 |
| Q9UHB6 | LIM domain and actin-binding protein 1 | LIMA1 | 490 | S | 1 | 199.4 | 1.03 | 1.04 | 0.98 | 1.10 | 1.13 | 1.06 | 0.93 | 0.89 | 0.91 |
| Q9UHB6 | LIM domain and actin-binding protein 1 | LIMA1 | 132 | S | 1 | 78.71 | 0.82 | 0.91 | 1.10 | 0.98 | 0.97 | 1.28 | NaN | 0.71 | 0.98 |
| Q9UHB6 | LIM domain and actin-binding protein 1 | LIMA1 | 698 | S | 1 | 168.5 | 0.76 | 0.71 | 0.90 | 0.89 | 0.88 | 0.86 | 0.83 | NaN | 0.99 |
| Q9UHB6 | LIM domain and actin-binding protein 1 | LIMA1 | 362 | S | 1 | 203.3 | 0.65 | 0.70 | 0.68 | 0.95 | 0.97 | 0.89 | 0.74 | 0.76 | 0.82 |
| Q9UHB6 | LIM domain and actin-binding protein 1 | LIMA1 | 609 | S | 1 | 117.8 | 0.70 | 0.65 | 0.62 | 0.75 | NaN | 0.77 | NaN | 1.08 | 0.92 |
| Q9UHB7 | AF4/FMR2 family member 4 | AFF4 | 1055 | S | 1 | 94.19 | NaN | NaN | NaN | 1.85 | NaN | NaN | 1.08 | 0.74 | NaN |
| Q9UHB7 | AF4/FMR2 family member 4 | AFF4 | 1058 | S | 1 | 99.53 | NaN | 1.13 | 1.01 | 1.39 | 1.29 | 1.16 | 0.55 | NaN | 0.73 |
| Q9UHB7 | AF4/FMR2 family member 4 | AFF4 | 703 | S | 1 | 113.2 | 1.16 | 1.14 | 1.25 | 1.63 | 1.71 | 1.13 | 1.13 | 0.97 | 0.99 |
| Q9UHB7 | AF4/FMR2 family member 4 | AFF4 | 706 | S | 1 | 69.7 | 2.54 | 2.10 | 2.19 | 2.55 | 2.62 | 2.25 | 1.38 | 1.22 | NaN |
| Q9UHB7 | AF4/FMR2 family member 4 | AFF4 | 180 | S | 0.957 | 88.28 | 1.70 | 0.93 | 1.60 | 1.60 | NaN | 1.51 | NaN | NaN | 0.92 |
| Q9UHD8 | Septin-9 | 9-Sep | 85 | S | 1 | 150.4 | 1.38 | 1.36 | 1.28 | 1.10 | 1.02 | 1.17 | 1.20 | 1.16 | 1.23 |
| Q9UHD8 | Septin-9 | 9-Sep | 30 | S | 1 | 262.4 | 1.77 | 1.72 | 1.86 | 1.62 | 1.65 | 1.47 | 1.86 | 1.38 | 1.75 |
| Q9UHK0 | Nuclear fragile X mental retardation-interacting protein | NUFIP1 | 338 | S | 1 | 70.42 | 1.08 | 1.30 | NaN | NaN | NaN | NaN | NaN | NaN | NaN |
| Q9UHK0 | Nuclear fragile X mental retardation-interacting protein | NUFIP1 | 340 | S | 0.997 | 70.42 | 1.08 | 1.30 | NaN | NaN | NaN | NaN | NaN | NaN | NaN |
| Q9UHK0 | Nuclear fragile X mental retardation-interacting protein | NUFIP1 | 342 | S | 0.994 | 70.42 | 1.08 | 1.30 | NaN | NaN | NaN | NaN | NaN | NaN | NaN |
| Q9UHR4 | Brain-specific angiogenesis inhibitor 1-associated protein 2-like protein 1 | BAIAP2L1 | 414 | S | 0.779 | 79.46 | 2.09 | 1.48 | 1.57 | 1.90 | NaN | NaN | 2.53 | NaN | 2.53 |
| Q9UHV7 | Mediator of RNA polymerase II transcription subunit 13 | MED13 | 890 | S | 0.97 | 53.36 | 1.08 | NaN | NaN | NaN | 1.02 | NaN | 0.93 | 1.18 | NaN |
| Q9UIG0 | Tyrosine-protein kinase | BAZ1B | 1468 | S | 1 | 173.9 | 0.88 | 1.14 | NaN | 0.80 | NaN | NaN | 1.56 | 1.11 | 1.03 |
| Q9UIQ6 | Leucyl-cystinyl aminopeptidase;Leucyl-cystinyl aminopeptidase, pregnancy serum form | LNPEP | 329 | S | 0.644 | 45.92 | 0.64 | NaN | 0.65 | 0.66 | 0.80 | 0.68 | NaN | 0.60 | 0.91 |
| Q9UJM3 | ERBB receptor feedback inhibitor 1 | ERRFI1 | 273 | S | 0.995 | 50.36 | NaN | NaN | 1.84 | NaN | NaN | NaN | 1.43 | 2.42 | NaN |
| Q9UJM3 | ERBB receptor feedback inhibitor 1 | ERRFI1 | 251 | S | 0.997 | 93.92 | 2.71 | 2.64 | 2.71 | 2.14 | 2.26 | NaN | 2.15 | 2.13 | 2.13 |
| Q9UJX2 | Cell division cycle protein 23 homolog | CDC23 | 588 | S | 0.996 | 79.88 | NaN | 0.59 | 1.33 | NaN | NaN | NaN | 1.18 | NaN | 0.86 |
| Q9UK58 | Cyclin-L1 | CCNL1 | 352 | S | 1 | 104.1 | 1.13 | 1.10 | 1.10 | 1.12 | 1.14 | 0.99 | 0.98 | 0.97 | 1.04 |
| Q9UK58 | Cyclin-L1 | CCNL1 | 335 | S | 0.982 | 149.2 | 2.35 | NaN | 1.95 | NaN | NaN | 1.75 | 2.24 | NaN | 1.78 |
| Q9UK58 | Cyclin-L1 | CCNL1 | 341 | S | 0.778 | 140.3 | NaN | 0.77 | 1.95 | NaN | NaN | 1.75 | NaN | 0.62 | 1.78 |
| Q9UK61 | Protein FAM208A | FAM208A | 927 | S | 0.763 | 47.73 | NaN | NaN | NaN | 0.60 | 0.54 | 0.56 | 1.01 | 0.96 | NaN |

| | | | | | | | | | | | | | | | |
|---|---|---|---|---|---|---|---|---|---|---|---|---|---|---|---|
| Q9UK76 | Hematological and neurological expressed 1 protein;Hematological and neurological expressed 1 protein, N-terminally | HN1 | 131 | S | 1 | 101.9 | 3.53 | 2.89 | NaN | 2.96 | NaN | 3.45 | NaN | 2.55 | 2.58 |
| Q9UK76 | Hematological and neurological expressed 1 protein;Hematological and neurological expressed 1 protein, N-terminally | HN1 | 87 | S | 0.997 | 319.4 | 1.03 | 1.04 | 0.99 | 0.83 | 0.83 | 0.82 | 1.75 | 1.74 | 1.82 |
| Q9UK76 | Hematological and neurological expressed 1 protein;Hematological and neurological expressed 1 protein, N-terminally | HN1 | 88 | S | 0.627 | 172.1 | NaN | 1.16 | NaN | 0.81 | 0.81 | 0.88 | 1.69 | 1.61 | NaN |
| Q9UKA4 | A-kinase anchor protein 11 | AKAP11 | 448 | S | 1 | 115.6 | 3.19 | 2.94 | 2.89 | NaN | NaN | NaN | NaN | NaN | NaN |
| Q9UKA4 | A-kinase anchor protein 11 | AKAP11 | 1240 | S | 0.501 | 66.33 | NaN | 0.78 | 0.77 | NaN | NaN | NaN | NaN | NaN | NaN |
| Q9UKA4 | A-kinase anchor protein 11 | AKAP11 | 1242 | S | 1 | 152.9 | 0.89 | 0.82 | 0.81 | 0.95 | 0.85 | 0.88 | 0.77 | 0.68 | 0.76 |
| Q9UKE5 | TRAF2 and NCK-interacting protein kinase | TNIK | 769 | S | 1 | 93.77 | 0.55 | 0.47 | NaN | NaN | NaN | NaN | NaN | NaN | NaN |
| Q9UKG1 | DCC-interacting protein 13-alpha | APPL1 | 401 | S | 0.829 | 91.59 | NaN | NaN | 4.72 | 5.07 | NaN | 3.88 | 4.80 | NaN | 3.93 |
| Q9UKJ3 | G patch domain-containing protein 8 | GPATCH 8 | 1081 | S | 0.982 | 71.4 | NaN | NaN | NaN | NaN | 1.05 | 1.12 | NaN | 0.82 | NaN |
| Q9UKJ3 | G patch domain-containing protein 8 | GPATCH 8 | 740 | S | 0.979 | 98 | 1.71 | 1.52 | 1.58 | 1.63 | NaN | 1.53 | 1.35 | NaN | 1.18 |
| Q9UKJ3 | G patch domain-containing protein 8 | GPATCH 8 | 653 | S | 0.822 | 57.62 | 1.58 | 1.33 | 1.55 | NaN | NaN | NaN | NaN | NaN | NaN |
| Q9UKL0 | REST corepressor 1 | RCOR1 | 260 | S | 1 | 170.2 | 0.74 | 0.65 | NaN | NaN | NaN | NaN | NaN | NaN | NaN |
| Q9UKM9 | RNA-binding protein Raly | RALY | 135 | S | 1 | 110.8 | 1.13 | 1.14 | 1.13 | 1.10 | 1.08 | 1.11 | 1.05 | 1.06 | 1.09 |
| Q9ULE0 | Protein WWC3 | WWC3 | 927 | S | 0.995 | 79.23 | NaN | NaN | NaN | NaN | 0.64 | NaN | 1.24 | 1.19 | 0.96 |
| Q9ULL5 | Proline-rich protein 12 | PRR12 | 651 | S | 1 | 98.63 | 0.51 | 0.58 | NaN | 0.49 | NaN | NaN | NaN | NaN | NaN |
| Q9ULW0 | Targeting protein for Xklp2 | TPX2 | 186 | S | 0.927 | 52.74 | 0.41 | 0.38 | NaN | 1.03 | NaN | NaN | 0.43 | NaN | NaN |
| Q9ULW0 | Targeting protein for Xklp2 | TPX2 | 738 | S | 1 | 144 | 2.34 | 3.77 | 2.45 | 4.98 | 6.39 | NaN | 1.34 | 1.30 | 1.49 |
| Q9ULX3 | RNA-binding protein NOB1 | NOB1 | 201 | S | 1 | 142.2 | NaN | NaN | NaN | NaN | 0.85 | 0.92 | NaN | NaN | NaN |
| Q9UNZ2 | NSFL1 cofactor p47 | NSFL1C | 114 | S | 1 | 136.4 | 1.00 | NaN | 1.02 | 1.11 | 1.01 | 0.88 | 0.83 | 0.86 | 0.80 |
| Q9UPT8 | Zinc finger CCCH domain-containing protein 4 | ZC3H4 | 1104 | S | 0.947 | 89.15 | 1.05 | NaN | 1.13 | 0.90 | 0.96 | 0.88 | 0.82 | 0.90 | 0.96 |
| Q9UPT8 | Zinc finger CCCH domain-containing protein 4 | ZC3H4 | 1110 | S | 0.754 | 88.57 | 1.08 | NaN | 0.93 | 0.98 | NaN | NaN | 1.05 | NaN | 0.87 |
| Q9UPT8 | Zinc finger CCCH domain-containing protein 4 | ZC3H4 | 1114 | S | 0.917 | 94.62 | 1.05 | 0.98 | 0.95 | 0.94 | 0.86 | 0.78 | NaN | 0.95 | 0.78 |
| Q9UPT8 | Zinc finger CCCH domain-containing protein 4 | ZC3H4 | 908 | S | 0.83 | 80.36 | NaN | NaN | 1.09 | 1.23 | 1.92 | 2.02 | 1.02 | NaN | NaN |
| Q9UPT8 | Zinc finger CCCH domain-containing protein 4 | ZC3H4 | 159 | S | 1 | 87.16 | 1.10 | 1.16 | 1.12 | 1.06 | 1.25 | 1.07 | 0.93 | 0.83 | NaN |

| | | | | | | | | | | | | | | | |
|---|---|---|---|---|---|---|---|---|---|---|---|---|---|---|---|
| Q9UPT8 | Zinc finger CCCH domain-containing protein 4 | ZC3H4 | 1269 | S | 0.695 | 45.13 | 1.54 | 1.80 | NaN | NaN | NaN | NaN | 1.96 | NaN | NaN |
| Q9UPT8 | Zinc finger CCCH domain-containing protein 4 | ZC3H4 | 1275 | S | 1 | 94.92 | 0.89 | 1.80 | 1.04 | 1.11 | 1.06 | 0.59 | 1.96 | 1.04 | 0.97 |
| Q9UPU5 | Ubiquitin carboxyl-terminal hydrolase 24 | USP24 | 1943 | S | 1 | 111.7 | NaN | NaN | NaN | 1.01 | NaN | NaN | 0.77 | NaN | 0.67 |
| Q9UPU5 | Ubiquitin carboxyl-terminal hydrolase 24 | USP24 | 2047 | S | 1 | 103.3 | 0.72 | 0.83 | 0.83 | 0.74 | 0.94 | 0.79 | 0.90 | 1.11 | 0.89 |
| Q9UPU7 | TBC1 domain family member 2B | TBC1D2B | 957 | S | 1 | 172.1 | 1.81 | 0.85 | 0.94 | 0.76 | 0.74 | 0.58 | 1.10 | 1.49 | 1.35 |
| Q9UQ35 | Serine/arginine repetitive matrix protein 2 | SRRM2 | 1404 | S | 0.997 | 270.5 | 0.73 | 0.82 | 0.65 | 0.86 | NaN | 0.71 | 0.62 | 0.70 | 0.59 |
| Q9UQ35 | Serine/arginine repetitive matrix protein 2 | SRRM2 | 1415 | S | 0.905 | 52.1 | 0.72 | 0.61 | NaN | NaN | NaN | NaN | NaN | NaN | NaN |
| Q9UQ35 | Serine/arginine repetitive matrix protein 2 | SRRM2 | 1012 | S | 0.625 | 79.37 | 1.58 | 1.33 | NaN | NaN | NaN | NaN | 1.40 | NaN | NaN |
| Q9UQ35 | Serine/arginine repetitive matrix protein 2 | SRRM2 | 1014 | S | 0.998 | 150.9 | 1.33 | 1.38 | NaN | 1.37 | 1.37 | 1.28 | NaN | 1.44 | 1.57 |
| Q9UQ35 | Serine/arginine repetitive matrix protein 2 | SRRM2 | 2132 | S | 1 | 85.86 | 1.34 | 1.28 | 1.62 | NaN | NaN | 1.39 | 1.41 | 1.55 | 1.42 |
| Q9UQ35 | Serine/arginine repetitive matrix protein 2 | SRRM2 | 1188 | S | 0.999 | 60.46 | 2.33 | 2.11 | NaN | NaN | NaN | NaN | NaN | NaN | NaN |
| Q9UQ35 | Serine/arginine repetitive matrix protein 2 | SRRM2 | 454 | S | 1 | 110.4 | 0.97 | NaN | NaN | NaN | NaN | NaN | 1.25 | 0.72 | NaN |
| Q9UQ35 | Serine/arginine repetitive matrix protein 2 | SRRM2 | 455 | S | 0.989 | 114.9 | 0.88 | 0.74 | NaN | 0.68 | NaN | NaN | 0.70 | NaN | 0.63 |
| Q9UQ35 | Serine/arginine repetitive matrix protein 2 | SRRM2 | 456 | S | 0.995 | 114.9 | 0.88 | NaN | NaN | 0.80 | 0.72 | 0.63 | 1.20 | NaN | 0.62 |
| Q9UQ35 | Serine/arginine repetitive matrix protein 2 | SRRM2 | 1318 | S | 1 | 122.8 | 0.48 | 0.55 | 0.71 | 0.82 | 0.79 | 0.66 | 1.81 | 1.21 | 1.41 |
| Q9UQ35 | Serine/arginine repetitive matrix protein 2 | SRRM2 | 1320 | S | 1 | 246.3 | 1.24 | 1.27 | 1.41 | 1.04 | 1.06 | 1.05 | 1.06 | 1.25 | 1.12 |
| Q9UQ35 | Serine/arginine repetitive matrix protein 2 | SRRM2 | 1329 | S | 1 | 160.8 | 1.11 | 1.08 | 1.12 | 1.08 | 1.01 | 1.04 | 1.39 | 1.39 | 1.38 |
| Q9UQ35 | Serine/arginine repetitive matrix protein 2 | SRRM2 | 2740 | S | 1 | 60.12 | 1.11 | 1.09 | NaN | NaN | NaN | NaN | NaN | NaN | NaN |
| Q9UQ35 | Serine/arginine repetitive matrix protein 2 | SRRM2 | 1124 | S | 0.999 | 134 | 1.04 | 0.95 | 1.08 | 1.28 | 1.18 | 1.32 | 0.98 | 0.96 | 0.89 |
| Q9UQ35 | Serine/arginine repetitive matrix protein 2 | SRRM2 | 322 | S | 0.999 | 263.5 | 0.84 | 0.82 | 1.18 | 0.91 | 1.15 | 1.05 | 1.40 | 1.25 | 0.94 |
| Q9UQ35 | Serine/arginine repetitive matrix protein 2 | SRRM2 | 323 | S | 0.999 | 245 | 0.90 | 0.96 | 0.93 | 0.88 | 0.88 | 0.83 | 0.95 | 0.99 | 1.12 |
| Q9UQ35 | Serine/arginine repetitive matrix protein 2 | SRRM2 | 1072 | S | 0.787 | 97.47 | NaN | 0.49 | NaN | NaN | NaN | NaN | 0.58 | 0.46 | NaN |
| Q9UQ35 | Serine/arginine repetitive matrix protein 2 | SRRM2 | 1073 | S | 0.905 | 97.16 | 0.54 | NaN | 0.53 | 0.51 | NaN | 0.53 | NaN | NaN | 0.51 |

| | | | | | | | | | | | | | | | |
|---|---|---|---|---|---|---|---|---|---|---|---|---|---|---|---|
| Q9UQ35 | Serine/arginine repetitive matrix protein 2 | SRRM2 | 1482 | S | 1 | 120.8 | 0.51 | 0.48 | 0.61 | 0.71 | 0.62 | 0.74 | 0.98 | 0.60 | 0.58 |
| Q9UQ35 | Serine/arginine repetitive matrix protein 2 | SRRM2 | 1483 | S | 1 | 172.2 | 0.64 | 0.58 | 0.49 | 0.78 | 0.62 | 0.63 | 0.73 | 0.60 | 0.70 |
| Q9UQ35 | Serine/arginine repetitive matrix protein 2 | SRRM2 | 1762 | S | 1 | 53 | 1.13 | NaN | NaN | NaN | 1.15 | NaN | 1.16 | 1.16 | NaN |
| Q9UQ35 | Serine/arginine repetitive matrix protein 2 | SRRM2 | 1764 | S | 1 | 53 | 1.13 | NaN | NaN | NaN | 1.15 | NaN | 1.16 | 1.16 | NaN |
| Q9UQ35 | Serine/arginine repetitive matrix protein 2 | SRRM2 | 435 | S | 1 | 121 | 0.95 | 0.95 | 1.02 | 0.97 | 0.83 | 0.99 | 0.99 | 0.97 | 0.98 |
| Q9UQ35 | Serine/arginine repetitive matrix protein 2 | SRRM2 | 436 | S | 1 | 121 | 0.95 | 0.95 | 1.02 | 0.97 | 0.83 | 0.99 | 0.99 | 0.97 | 0.98 |
| Q9UQ35 | Serine/arginine repetitive matrix protein 2 | SRRM2 | 437 | S | 1 | 121 | 0.95 | 0.95 | 1.02 | 0.97 | 0.84 | 0.99 | 0.99 | 0.91 | 0.89 |
| Q9UQ35 | Serine/arginine repetitive matrix protein 2 | SRRM2 | 440 | S | 1 | 121 | 0.95 | 0.95 | 1.02 | 0.97 | 0.84 | 0.99 | 0.99 | 0.91 | 0.89 |
| Q9UQ35 | Serine/arginine repetitive matrix protein 2 | SRRM2 | 377 | S | 0.999 | 170.5 | 1.00 | 1.91 | 0.97 | 1.33 | 0.88 | 1.53 | 1.08 | 1.10 | NaN |
| Q9UQ35 | Serine/arginine repetitive matrix protein 2 | SRRM2 | 387 | S | 0.986 | 92.7 | NaN | 2.09 | 1.20 | NaN | NaN | NaN | NaN | NaN | NaN |
| Q9UQ35 | Serine/arginine repetitive matrix protein 2 | SRRM2 | 398 | S | 0.873 | 170.5 | 0.97 | 1.07 | 0.97 | 1.33 | 0.92 | 1.53 | 0.82 | 1.10 | NaN |
| Q9UQ35 | Serine/arginine repetitive matrix protein 2 | SRRM2 | 892 | S | 0.792 | 92.94 | NaN | NaN | NaN | NaN | 0.98 | 0.86 | NaN | 1.03 | NaN |
| Q9UQ35 | Serine/arginine repetitive matrix protein 2 | SRRM2 | 895 | S | 0.724 | 79.29 | NaN | NaN | 1.00 | NaN | NaN | NaN | 1.12 | NaN | 1.01 |
| Q9UQ35 | Serine/arginine repetitive matrix protein 2 | SRRM2 | 1462 | S | 0.659 | 137.4 | NaN | NaN | NaN | NaN | 0.87 | 0.55 | 0.48 | NaN | NaN |
| Q9UQ35 | Serine/arginine repetitive matrix protein 2 | SRRM2 | 1463 | S | 0.922 | 129.2 | 0.59 | NaN | 0.62 | 0.83 | NaN | 0.81 | 0.48 | NaN | NaN |
| Q9UQ35 | Serine/arginine repetitive matrix protein 2 | SRRM2 | 2690 | S | 0.942 | 105.2 | 0.74 | NaN | 1.51 | NaN | NaN | NaN | NaN | NaN | 1.47 |
| Q9UQ35 | Serine/arginine repetitive matrix protein 2 | SRRM2 | 2692 | S | 0.999 | 94.19 | 1.40 | 1.59 | NaN | 1.30 | 1.18 | 1.27 | 1.39 | 1.41 | NaN |
| Q9UQ35 | Serine/arginine repetitive matrix protein 2 | SRRM2 | 2694 | S | 1 | 105.2 | 1.40 | 1.59 | 1.51 | 1.30 | 1.18 | 1.27 | 1.39 | 1.41 | 1.47 |
| Q9UQ35 | Serine/arginine repetitive matrix protein 2 | SRRM2 | 1179 | S | 1 | 160 | 0.86 | 0.83 | 0.87 | 0.99 | 1.04 | 0.97 | 0.56 | 0.78 | 0.80 |
| Q9UQ35 | Serine/arginine repetitive matrix protein 2 | SRRM2 | 2449 | S | 0.948 | 211.3 | 2.16 | 2.23 | 2.42 | NaN | 2.09 | 1.75 | NaN | 2.06 | 1.89 |
| Q9UQ35 | Serine/arginine repetitive matrix protein 2 | SRRM2 | 2121 | S | 0.927 | 55.84 | 0.98 | 1.41 | 1.44 | NaN | 2.10 | NaN | NaN | 0.84 | NaN |
| Q9UQ35 | Serine/arginine repetitive matrix protein 2 | SRRM2 | 2123 | S | 0.934 | 55.84 | 0.98 | 1.41 | 1.44 | NaN | 2.10 | NaN | NaN | 0.84 | NaN |
| Q9UQ35 | Serine/arginine repetitive matrix protein 2 | SRRM2 | 2100 | S | 1 | 98 | 1.00 | 1.00 | 1.21 | 1.01 | 0.93 | 1.07 | 1.12 | 1.18 | NaN |

| | | | | | | | | | | | | | | | |
|---|---|---|---|---|---|---|---|---|---|---|---|---|---|---|---|
| Q9UQ35 | Serine/arginine repetitive matrix protein 2 | SRRM2 | 2102 | S | 1 | 98 | 1.00 | 1.00 | 1.21 | 1.01 | 0.93 | 1.07 | 1.12 | 1.18 | NaN |
| Q9UQ35 | Serine/arginine repetitive matrix protein 2 | SRRM2 | 854 | S | 0.917 | 78.16 | 0.95 | 0.78 | 1.60 | 0.91 | NaN | 1.55 | 0.83 | 2.17 | 2.32 |
| Q9UQ35 | Serine/arginine repetitive matrix protein 2 | SRRM2 | 857 | S | 1 | 203.2 | 0.76 | 0.75 | 0.74 | 0.87 | 0.77 | 1.55 | 0.92 | 0.88 | 0.92 |
| Q9UQ35 | Serine/arginine repetitive matrix protein 2 | SRRM2 | 1083 | S | 1 | 173.6 | 0.98 | 0.99 | 0.99 | 0.95 | 1.07 | 0.97 | 1.07 | 1.07 | 1.11 |
| Q9UQ35 | Serine/arginine repetitive matrix protein 2 | SRRM2 | 994 | S | 0.98 | 86.55 | 1.96 | 1.70 | 1.96 | 1.71 | 1.45 | 1.68 | 1.83 | 2.13 | 1.86 |
| Q9UQ35 | Serine/arginine repetitive matrix protein 2 | SRRM2 | 839 | S | 0.746 | 70.65 | 1.23 | 1.26 | 1.32 | 1.23 | 1.14 | 1.31 | 1.24 | NaN | 1.38 |
| Q9UQ35 | Serine/arginine repetitive matrix protein 2 | SRRM2 | 2702 | S | 1 | 89.56 | 1.04 | 1.10 | 0.99 | 0.92 | 0.94 | 0.97 | 1.05 | 1.07 | 1.14 |
| Q9UQ35 | Serine/arginine repetitive matrix protein 2 | SRRM2 | 1729 | S | 1 | 59.15 | NaN | 1.31 | 0.43 | NaN | 0.51 | NaN | NaN | 0.77 | NaN |
| Q9UQ35 | Serine/arginine repetitive matrix protein 2 | SRRM2 | 1732 | S | 1 | 59.15 | NaN | 1.31 | 0.43 | NaN | 0.51 | NaN | NaN | 0.77 | NaN |
| Q9UQ35 | Serine/arginine repetitive matrix protein 2 | SRRM2 | 950 | S | 1 | 76.05 | 1.22 | 1.36 | 1.18 | 1.27 | 1.18 | 1.15 | NaN | 1.59 | 1.56 |
| Q9UQ35 | Serine/arginine repetitive matrix protein 2 | SRRM2 | 952 | S | 1 | 102.7 | 0.80 | 0.91 | 1.02 | 1.28 | 1.02 | 0.95 | 0.95 | 0.95 | 0.75 |
| Q9UQ35 | Serine/arginine repetitive matrix protein 2 | SRRM2 | 954 | S | 0.999 | 127.3 | 0.39 | 0.40 | 0.42 | 0.45 | 0.45 | 0.42 | 0.40 | 0.41 | 0.38 |
| Q9UQ35 | Serine/arginine repetitive matrix protein 2 | SRRM2 | 957 | S | 0.917 | 69.11 | NaN | NaN | NaN | NaN | 1.18 | 1.21 | NaN | 1.59 | 1.56 |
| Q9UQ35 | Serine/arginine repetitive matrix protein 2 | SRRM2 | 1691 | S | 0.896 | 47.61 | 1.12 | 1.37 | NaN | NaN | NaN | NaN | NaN | NaN | NaN |
| Q9UQ35 | Serine/arginine repetitive matrix protein 2 | SRRM2 | 1694 | S | 0.964 | 80.68 | 0.58 | 0.55 | 0.54 | 0.65 | 0.59 | 0.63 | NaN | NaN | NaN |
| Q9UQ35 | Serine/arginine repetitive matrix protein 2 | SRRM2 | 2581 | S | 0.984 | 127.8 | 0.97 | 1.01 | 0.97 | 0.97 | 0.98 | 0.94 | 1.06 | 1.07 | 1.09 |
| Q9UQ35 | Serine/arginine repetitive matrix protein 2 | SRRM2 | 346 | S | 0.975 | 51.7 | 1.06 | 1.09 | NaN | NaN | 1.01 | 0.92 | 1.15 | NaN | NaN |
| Q9UQ35 | Serine/arginine repetitive matrix protein 2 | SRRM2 | 351 | S | 0.996 | 102.1 | 1.00 | 1.04 | 1.05 | 0.95 | 0.94 | 0.92 | 1.09 | 1.08 | 1.10 |
| Q9UQ35 | Serine/arginine repetitive matrix protein 2 | SRRM2 | 353 | S | 1 | 102.1 | 1.00 | 1.04 | 1.05 | 0.95 | 0.94 | 0.92 | 1.09 | 1.08 | 1.09 |
| Q9UQ35 | Serine/arginine repetitive matrix protein 2 | SRRM2 | 875 | S | 1 | 156.7 | 1.04 | 1.10 | NaN | NaN | 1.06 | 1.37 | 1.27 | 1.23 | 1.92 |
| Q9UQ35 | Serine/arginine repetitive matrix protein 2 | SRRM2 | 876 | S | 1 | 236.9 | 1.02 | 1.08 | 0.98 | 1.02 | 1.06 | 1.09 | 1.25 | NaN | 1.25 |
| Q9UQ35 | Serine/arginine repetitive matrix protein 2 | SRRM2 | 1132 | S | 1 | 131 | NaN | 0.82 | 0.84 | 0.95 | 0.95 | 0.94 | 0.71 | 0.74 | 0.76 |
| Q9UQ35 | Serine/arginine repetitive matrix protein 2 | SRRM2 | 1444 | S | 1 | 148.5 | NaN | 0.65 | 0.69 | 0.74 | 0.71 | NaN | NaN | 0.60 | NaN |

| | | | | | | | | | | | | | | | |
|---|---|---|---|---|---|---|---|---|---|---|---|---|---|---|---|
| Q9UQ35 | Serine/arginine repetitive matrix protein 2 | SRRM2 | 1541 | S | 0.97 | 51 | NaN | NaN | 0.54 | NaN | 0.89 | 0.38 | 0.75 | 0.66 | NaN |
| Q9UQ35 | Serine/arginine repetitive matrix protein 2 | SRRM2 | 1550 | S | 0.604 | 50.51 | NaN | NaN | 0.54 | NaN | 0.89 | NaN | 0.59 | 0.66 | NaN |
| Q9UQ35 | Serine/arginine repetitive matrix protein 2 | SRRM2 | 846 | S | 0.538 | 75.18 | 0.95 | NaN | 1.02 | 1.58 | NaN | 1.09 | 2.11 | 0.81 | 2.32 |
| Q9UQ35 | Serine/arginine repetitive matrix protein 2 | SRRM2 | 778 | S | 1 | 85.86 | 2.64 | 2.39 | 2.47 | 1.99 | 4.32 | 1.49 | 2.07 | 3.97 | 2.58 |
| Q9UQ35 | Serine/arginine repetitive matrix protein 2 | SRRM2 | 780 | S | 1 | 85.86 | 2.64 | 2.56 | 2.47 | 1.99 | 1.85 | 1.49 | 1.68 | 3.16 | 2.25 |
| Q9UQ35 | Serine/arginine repetitive matrix protein 2 | SRRM2 | 782 | S | 0.671 | 96.64 | 1.04 | 2.39 | 0.92 | NaN | 1.85 | 1.07 | 2.56 | NaN | NaN |
| Q9UQ35 | Serine/arginine repetitive matrix protein 2 | SRRM2 | 783 | S | 0.988 | 89.9 | 2.64 | 2.56 | 2.47 | 0.97 | 4.32 | 1.84 | 2.47 | 2.51 | 0.96 |
| Q9UQ35 | Serine/arginine repetitive matrix protein 2 | SRRM2 | 2067 | S | 1 | 73.93 | 2.36 | 1.20 | 0.24 | 1.22 | 1.23 | 1.28 | 1.19 | 1.21 | 1.28 |
| Q9UQ35 | Serine/arginine repetitive matrix protein 2 | SRRM2 | 2071 | S | 1 | 73.93 | 2.36 | 1.20 | 1.32 | 1.22 | 1.23 | 1.28 | 1.19 | 1.21 | 1.28 |
| Q9UQ35 | Serine/arginine repetitive matrix protein 2 | SRRM2 | 1916 | S | 1 | 112.6 | 0.92 | 0.94 | 0.96 | 0.95 | 0.97 | 0.98 | 1.11 | 1.01 | NaN |
| Q9UQ35 | Serine/arginine repetitive matrix protein 2 | SRRM2 | 2046 | S | 1 | 80.98 | 0.24 | 0.19 | 1.58 | 1.29 | 1.35 | 1.35 | 1.58 | 1.59 | 1.67 |
| Q9UQ35 | Serine/arginine repetitive matrix protein 2 | SRRM2 | 1497 | S | 1 | 122.6 | 0.80 | 0.52 | 1.09 | 1.03 | 1.08 | 1.00 | 1.25 | 1.19 | 1.19 |
| Q9UQ35 | Serine/arginine repetitive matrix protein 2 | SRRM2 | 1499 | S | 1 | 122.6 | 0.80 | 1.10 | 1.09 | 1.03 | 1.08 | 1.00 | 1.25 | 1.19 | 1.19 |
| Q9UQ35 | Serine/arginine repetitive matrix protein 2 | SRRM2 | 1501 | S | 1 | 103.3 | 1.17 | 0.52 | 1.09 | 1.07 | 1.10 | 1.02 | 1.28 | 1.27 | 1.22 |
| Q9UQ35 | Serine/arginine repetitive matrix protein 2 | SRRM2 | 1502 | S | 1 | 122.6 | 1.11 | 1.10 | 1.09 | 1.03 | 1.08 | 1.00 | 1.25 | 1.19 | 1.19 |
| Q9UQ35 | Serine/arginine repetitive matrix protein 2 | SRRM2 | 1099 | S | 0.99 | 52.93 | NaN | NaN | NaN | 1.11 | 1.16 | 1.07 | 1.37 | 1.27 | 1.54 |
| Q9UQ35 | Serine/arginine repetitive matrix protein 2 | SRRM2 | 1101 | S | 0.986 | 52.93 | 1.31 | 1.19 | NaN | 1.11 | 1.16 | 1.07 | 1.37 | 1.27 | 1.54 |
| Q9UQ35 | Serine/arginine repetitive matrix protein 2 | SRRM2 | 1102 | S | 0.887 | 88.77 | 1.31 | 1.19 | NaN | NaN | NaN | NaN | NaN | NaN | NaN |
| Q9UQ35 | Serine/arginine repetitive matrix protein 2 | SRRM2 | 1103 | S | 0.999 | 166.5 | 0.89 | 0.86 | 0.81 | 0.63 | 0.56 | 0.57 | 0.70 | 0.75 | 0.85 |
| Q9UQ35 | Serine/arginine repetitive matrix protein 2 | SRRM2 | 1112 | S | 0.955 | 93.88 | 0.68 | 0.63 | 0.69 | 0.72 | 0.59 | 0.65 | 0.56 | 0.80 | 0.77 |
| Q9UQ35 | Serine/arginine repetitive matrix protein 2 | SRRM2 | 2020 | S | 1 | 76.85 | 1.36 | 1.42 | 1.34 | 1.17 | 1.22 | 1.20 | 1.42 | 1.43 | 1.48 |
| Q9UQ35 | Serine/arginine repetitive matrix protein 2 | SRRM2 | 2407 | S | 0.992 | 57.53 | 1.67 | 1.67 | 1.60 | 1.38 | NaN | 1.38 | 1.48 | 1.58 | 1.49 |
| Q9UQ35 | Serine/arginine repetitive matrix protein 2 | SRRM2 | 1925 | S | 1 | 72.58 | 1.37 | 1.38 | 1.27 | 1.19 | NaN | 1.20 | 1.45 | 1.42 | 1.52 |

| | | | | | | | | | | | | | | |
|---|---|---|---|---|---|---|---|---|---|---|---|---|---|---|
| Q9UQ35 | Serine/arginine repetitive matrix protein 2 | SRRM2 | 1975 | S | 0.948 | 86.11 | 1.14 | 0.75 | 0.76 | 0.81 | 0.83 | 0.84 | 0.90 | 0.85 | NaN |
| Q9UQ35 | Serine/arginine repetitive matrix protein 2 | SRRM2 | 1893 | S | 0.91 | 77.87 | 0.70 | 0.73 | NaN | 0.84 | 0.80 | 0.79 | 0.94 | 0.94 | 0.88 |
| Q9UQ35 | Serine/arginine repetitive matrix protein 2 | SRRM2 | 1987 | S | 0.997 | 128.9 | 0.75 | 0.72 | 0.70 | 0.76 | 0.78 | 0.79 | 0.91 | 0.92 | 0.93 |
| Q9UQ35 | Serine/arginine repetitive matrix protein 2 | SRRM2 | 1382 | S | 0.905 | 170 | 1.60 | 1.33 | NaN | NaN | NaN | NaN | 0.92 | NaN | NaN |
| Q9UQ35 | Serine/arginine repetitive matrix protein 2 | SRRM2 | 1387 | S | 1 | 232.7 | 0.92 | 0.89 | 0.88 | 0.83 | 0.76 | 0.80 | 0.86 | 0.89 | 0.83 |
| Q9UQ35 | Serine/arginine repetitive matrix protein 2 | SRRM2 | 901 | S | 0.766 | 66.39 | NaN | NaN | 0.91 | NaN | 0.89 | 0.83 | NaN | NaN | 0.97 |
| Q9UQ35 | Serine/arginine repetitive matrix protein 2 | SRRM2 | 902 | S | 0.717 | 76.16 | NaN | NaN | NaN | NaN | NaN | NaN | NaN | 1.08 | 1.09 |
| Q9UQ35 | Serine/arginine repetitive matrix protein 2 | SRRM2 | 908 | S | 0.996 | 93.39 | 0.97 | 0.91 | 0.91 | 0.84 | 0.87 | 0.84 | 1.06 | 1.08 | 0.97 |
| Q9UQ35 | Serine/arginine repetitive matrix protein 2 | SRRM2 | 910 | S | 0.652 | 52.25 | NaN | 1.80 | 1.06 | NaN | NaN | NaN | NaN | 1.11 | 1.14 |
| Q9UQ35 | Serine/arginine repetitive matrix protein 2 | SRRM2 | 912 | S | 0.968 | 93.39 | NaN | 1.02 | 1.07 | 1.00 | NaN | NaN | 1.07 | 1.11 | 1.14 |
| Q9UQ35 | Serine/arginine repetitive matrix protein 2 | SRRM2 | 913 | S | 0.584 | 48.33 | NaN | 1.80 | 1.06 | NaN | NaN | NaN | 1.07 | NaN | 1.18 |
| Q9UQ35 | Serine/arginine repetitive matrix protein 2 | SRRM2 | 914 | S | 0.818 | 93.39 | NaN | 1.02 | 1.07 | 1.00 | NaN | NaN | 1.07 | 1.11 | 1.10 |
| Q9UQ35 | Serine/arginine repetitive matrix protein 2 | SRRM2 | 295 | S | 1 | 199 | 1.15 | 1.14 | 1.08 | 0.93 | 1.01 | 0.92 | 1.11 | 1.09 | 1.22 |
| Q9UQ35 | Serine/arginine repetitive matrix protein 2 | SRRM2 | 297 | S | 0.999 | 199 | 1.15 | 1.14 | 1.08 | 0.93 | 0.99 | 0.92 | 1.11 | 1.12 | 1.22 |
| Q9UQ35 | Serine/arginine repetitive matrix protein 2 | SRRM2 | 2398 | S | 0.993 | 158.9 | 1.02 | 1.06 | 1.10 | 1.01 | 0.99 | 0.95 | 1.04 | 1.21 | 1.20 |
| Q9UQ35 | Serine/arginine repetitive matrix protein 2 | SRRM2 | 972 | S | 0.812 | 91.28 | NaN | NaN | NaN | 0.66 | NaN | 0.80 | 0.76 | NaN | NaN |
| Q9UQ35 | Serine/arginine repetitive matrix protein 2 | SRRM2 | 973 | S | 0.773 | 91.28 | NaN | NaN | NaN | 0.66 | NaN | 0.80 | 0.76 | NaN | NaN |
| Q9UQ35 | Serine/arginine repetitive matrix protein 2 | SRRM2 | 974 | S | 0.8 | 86.94 | NaN | NaN | NaN | 0.66 | 0.75 | 0.80 | NaN | NaN | NaN |
| Q9UQR0 | Sex comb on midleg-like protein 2 | SCML2 | 267 | S | 0.548 | 81.73 | NaN | NaN | NaN | NaN | 0.96 | 0.91 | NaN | 0.49 | NaN |
| Q9Y232 | Chromodomain Y-like protein | CDYL | 201 | S | 1 | 80.98 | 0.98 | 0.94 | 1.17 | 1.10 | 1.26 | 1.32 | 0.71 | 0.81 | 0.85 |
| Q9Y2D5 | A-kinase anchor protein 2 | AKAP2 | 152 | S | 0.955 | 218.9 | 2.21 | 2.48 | 2.48 | 1.67 | 1.61 | 1.72 | 1.57 | 1.61 | 1.62 |
| Q9Y2D5 | A-kinase anchor protein 2 | AKAP2 | 393 | S | 1 | 195.5 | 1.04 | 0.87 | 1.25 | 1.01 | 1.07 | 0.99 | 0.79 | 1.29 | 1.04 |
| Q9Y2D5 | A-kinase anchor protein 2 | AKAP2 | 778 | S | 0.985 | 229.3 | 1.24 | 1.36 | 1.51 | 1.05 | 0.96 | 1.17 | 1.25 | 1.34 | 1.30 |
| Q9Y2U5 | Mitogen-activated protein kinase kinase kinase 2 | MAP3K2 | 163 | S | 0.63 | 98.53 | 1.14 | 0.96 | NaN | NaN | NaN | NaN | 1.11 | NaN | NaN |
| Q9Y2U8 | Inner nuclear membrane protein Man1 | LEMD3 | 140 | S | 0.998 | 85.56 | 1.37 | 1.19 | 1.72 | 1.49 | 1.79 | 1.28 | 0.91 | 1.11 | 1.20 |

| | | | | | | | | | | | | | | | |
|---|---|---|---|---|---|---|---|---|---|---|---|---|---|---|---|
| Q9Y2U8 | Inner nuclear membrane protein Man1 | LEMD3 | 141 | S | 0.994 | 85.56 | 1.37 | 1.19 | 1.72 | 1.49 | 1.79 | 1.28 | 0.91 | 1.11 | 1.20 |
| Q9Y2W1 | Thyroid hormone receptor-associated protein 3 | THRAP3 | 243 | S | 1 | 228.6 | 1.19 | 1.16 | 1.15 | 1.09 | 1.17 | 1.15 | 1.28 | 1.25 | 1.32 |
| Q9Y2W1 | Thyroid hormone receptor-associated protein 3 | THRAP3 | 248 | S | 1 | 110.9 | 1.27 | 1.31 | 1.24 | 1.13 | 1.14 | 1.13 | 1.46 | 1.44 | 1.48 |
| Q9Y2W1 | Thyroid hormone receptor-associated protein 3 | THRAP3 | 253 | S | 1 | 110.9 | 3.18 | 3.17 | 3.22 | 4.02 | 4.42 | 1.36 | 1.00 | 0.77 | NaN |
| Q9Y2W1 | Thyroid hormone receptor-associated protein 3 | THRAP3 | 379 | S | 1 | 205.9 | 2.03 | 2.78 | 2.77 | 1.99 | 2.36 | NaN | 2.66 | 2.44 | 2.60 |
| Q9Y2W1 | Thyroid hormone receptor-associated protein 3 | THRAP3 | 682 | S | 0.999 | 155.5 | 1.05 | 1.10 | 1.10 | 1.02 | 1.08 | 1.08 | 1.28 | 1.13 | 1.34 |
| Q9Y2W1 | Thyroid hormone receptor-associated protein 3 | THRAP3 | 575 | S | 1 | 65.81 | 0.98 | 1.09 | 1.32 | 1.94 | 1.81 | 1.55 | 1.67 | 1.56 | NaN |
| Q9Y2W1 | Thyroid hormone receptor-associated protein 3 | THRAP3 | 672 | S | 1 | 66.71 | 0.90 | 0.97 | 0.93 | 0.87 | NaN | 0.90 | 0.97 | 0.97 | 1.01 |
| Q9Y2W1 | Thyroid hormone receptor-associated protein 3 | THRAP3 | 535 | S | 0.995 | 91.32 | 0.99 | NaN | NaN | 0.94 | NaN | NaN | 1.21 | NaN | 1.09 |
| Q9Y2W1 | Thyroid hormone receptor-associated protein 3 | THRAP3 | 55 | S | 0.994 | 76.28 | 0.42 | NaN | NaN | NaN | 0.50 | NaN | 0.42 | 0.45 | 0.48 |
| Q9Y2X3 | Nucleolar protein 58 | NOP58 | 502 | S | 1 | 168.5 | 0.89 | 0.88 | 0.89 | 0.74 | 0.66 | 0.67 | 1.15 | 0.90 | 1.03 |
| Q9Y2X3 | Nucleolar protein 58 | NOP58 | 514 | S | 1 | 168.5 | 1.13 | 1.09 | 1.01 | 1.58 | 2.58 | 1.32 | 0.73 | 0.78 | 0.97 |
| Q9Y3E7 | Charged multivesicular body protein 3 | CHMP3 | 200 | S | 1 | 153 | 1.02 | 1.02 | NaN | NaN | NaN | NaN | NaN | NaN | NaN |
| Q9Y3F4 | Serine-threonine kinase receptor-associated protein | STRAP | 335 | S | 0.998 | 110 | NaN | 3.32 | NaN | 2.27 | NaN | 2.22 | 1.34 | 1.65 | NaN |
| Q9Y3F4 | Serine-threonine kinase receptor-associated protein | STRAP | 338 | S | 0.998 | 110 | NaN | 3.32 | NaN | 2.27 | NaN | 2.22 | 1.34 | 1.65 | NaN |
| Q9Y3T9 | Nucleolar complex protein 2 homolog | NOC2L | 672 | S | 1 | 218.4 | 1.20 | NaN | NaN | 1.13 | 1.13 | NaN | NaN | 1.05 | NaN |
| Q9Y3T9 | Nucleolar complex protein 2 homolog | NOC2L | 673 | S | 1 | 218.4 | 1.20 | NaN | NaN | 1.13 | 1.13 | NaN | NaN | 1.05 | NaN |
| Q9Y3T9 | Nucleolar complex protein 2 homolog | NOC2L | 49 | S | 1 | 78.65 | 0.67 | 0.73 | 0.71 | 0.97 | 1.04 | 0.57 | 0.90 | 0.83 | 0.96 |
| Q9Y3T9 | Nucleolar complex protein 2 homolog | NOC2L | 56 | S | 0.885 | 78.65 | 1.49 | NaN | NaN | NaN | 1.30 | 1.38 | 0.59 | NaN | NaN |
| Q9Y426 | C2 domain-containing protein | C2CD2 | 438 | S | 0.608 | 52.73 | 0.88 | 1.11 | NaN | NaN | NaN | NaN | 0.84 | NaN | NaN |
| Q9Y490 | Talin-1 | TLN1 | 425 | S | 0.996 | 161 | 0.74 | NaN | NaN | 1.63 | 1.74 | NaN | NaN | NaN | NaN |
| Q9Y4B5 | Microtubule cross-linking factor 1 | MTCL1 | 756 | S | 0.947 | 81.2 | 0.32 | NaN | 0.37 | 0.56 | 0.51 | NaN | NaN | 0.46 | 0.42 |
| Q9Y4B5 | Microtubule cross-linking factor 1 | MTCL1 | 1421 | S | 1 | 80.53 | 0.84 | 0.90 | 0.77 | 0.99 | 0.97 | 0.94 | 0.86 | 0.89 | 0.79 |
| Q9Y4B5 | Microtubule cross-linking factor 1 | MTCL1 | 263 | S | 1 | 90.53 | 1.04 | 1.01 | 1.17 | 1.40 | 1.34 | 1.31 | 1.22 | 1.26 | 1.47 |
| Q9Y4B5 | Microtubule cross-linking factor 1 | MTCL1 | 306 | S | 1 | 65.09 | NaN | 0.71 | 0.59 | NaN | NaN | 0.72 | 0.78 | 0.82 | 0.78 |

| | | | | | | | | | | | | | | | |
|---|---|---|---|---|---|---|---|---|---|---|---|---|---|---|---|
| Q9Y4B5 | Microtubule cross-linking factor 1 | MTCL1 | 1812 | S | 1 | 80.54 | 0.76 | NaN | 0.80 | 1.11 | 0.95 | NaN | 0.83 | 0.92 | 0.80 |
| Q9Y4B5 | Microtubule cross-linking factor 1 | MTCL1 | 1814 | S | 1 | 80.54 | 0.76 | NaN | 0.80 | 1.11 | 0.95 | NaN | 0.83 | 0.92 | 0.80 |
| Q9Y4B5 | Microtubule cross-linking factor 1 | MTCL1 | 776 | S | 1 | 215.1 | 0.76 | 0.81 | NaN | NaN | NaN | 0.85 | NaN | NaN | 0.67 |
| Q9Y4B5 | Microtubule cross-linking factor 1 | MTCL1 | 549 | S | 0.994 | 67.5 | NaN | NaN | 0.94 | 1.44 | NaN | 1.46 | NaN | NaN | 0.82 |
| Q9Y4G2 | Pleckstrin homology domain-containing family M member 1 | PLEKHM1 | 435 | S | 0.778 | 80.78 | NaN | NaN | NaN | 0.78 | NaN | 1.24 | NaN | NaN | NaN |
| Q9Y4H2 | Insulin receptor substrate 2 | IRS2 | 730 | S | 0.592 | 111.8 | 1.41 | 1.88 | NaN | NaN | NaN | NaN | NaN | NaN | NaN |
| Q9Y4H2 | Insulin receptor substrate 2 | IRS2 | 731 | S | 0.948 | 183.8 | NaN | 1.24 | NaN | 1.30 | 0.94 | 0.86 | NaN | NaN | NaN |
| Q9Y4H2 | Insulin receptor substrate 2 | IRS2 | 735 | S | 0.864 | 183.8 | 1.20 | 1.88 | 1.08 | 0.91 | 2.53 | NaN | 0.94 | 0.97 | 0.86 |
| Q9Y4H2 | Insulin receptor substrate 2 | IRS2 | 1203 | S | 0.866 | 217.7 | 2.25 | 1.93 | 2.40 | NaN | 1.62 | NaN | NaN | NaN | NaN |
| Q9Y4H2 | Insulin receptor substrate 2 | IRS2 | 973 | S | 0.852 | 102.6 | 0.79 | 0.87 | NaN | NaN | NaN | NaN | NaN | 0.81 | NaN |
| Q9Y4H2 | Insulin receptor substrate 2 | IRS2 | 976 | S | 0.867 | 69.98 | NaN | NaN | NaN | 0.74 | 0.72 | NaN | 0.92 | NaN | NaN |
| Q9Y4P1 | Cysteine protease ATG4B | ATG4B | 383 | S | 1 | 158.4 | 1.27 | 1.33 | NaN | 1.52 | 1.21 | 1.27 | 1.36 | 1.36 | NaN |
| Q9Y4P8 | WD repeat domain phosphoinositide-interacting protein 2 | WIPI2 | 412 | S | 0.7 | 139 | 0.83 | NaN | 0.73 | 0.83 | NaN | 0.82 | 1.07 | NaN | 1.05 |
| Q9Y5B6 | PAX3- and PAX7-binding protein 1 | PAXBP1 | 262 | S | 1 | 84.67 | 1.11 | 1.16 | NaN | 1.02 | 1.18 | 1.09 | NaN | NaN | 0.96 |
| Q9Y5J1 | U3 small nucleolar RNA-associated protein 18 homolog | UTP18 | 206 | S | 0.999 | 245.6 | 0.68 | NaN | NaN | 0.89 | 0.82 | NaN | NaN | 0.87 | NaN |
| Q9Y5J1 | U3 small nucleolar RNA-associated protein 18 homolog | UTP18 | 210 | S | 1 | 245.6 | 0.71 | 0.79 | 0.94 | 0.84 | 0.82 | 0.83 | 0.79 | 0.95 | 0.90 |
| Q9Y5J1 | U3 small nucleolar RNA-associated protein 18 homolog | UTP18 | 121 | S | 1 | 271.7 | 0.69 | 0.71 | 0.77 | 0.79 | 0.77 | 0.72 | 0.66 | 0.63 | 0.67 |
| Q9Y5J1 | U3 small nucleolar RNA-associated protein 18 homolog | UTP18 | 124 | S | 1 | 183.6 | 0.83 | 0.57 | 0.49 | 0.49 | 0.52 | 0.54 | 0.68 | 0.76 | 0.78 |
| Q9Y5T5 | Ubiquitin carboxyl-terminal hydrolase 16 | USP16 | 415 | S | 1 | 197.3 | 0.59 | 0.64 | 0.56 | 0.69 | 0.58 | 0.58 | 0.77 | 0.80 | 0.66 |
| Q9Y5T5 | Ubiquitin carboxyl-terminal hydrolase 16 | USP16 | 552 | S | 0.748 | 190 | 0.55 | 0.64 | NaN | NaN | NaN | NaN | NaN | NaN | NaN |
| Q9Y608 | Leucine-rich repeat flightless-interacting protein 2 | LRRFIP2 | 260 | S | 0.999 | 70.06 | NaN | NaN | 2.03 | 1.61 | 1.28 | 1.54 | 3.46 | 3.85 | 4.08 |
| Q9Y608 | Leucine-rich repeat flightless-interacting protein 2 | LRRFIP2 | 328 | S | 1 | 254.2 | 1.07 | 1.10 | 1.03 | 0.90 | 0.93 | 0.88 | 1.09 | 1.07 | NaN |
| Q9Y608 | Leucine-rich repeat flightless-interacting protein 2 | LRRFIP2 | 320 | S | 0.817 | 109 | NaN | NaN | NaN | NaN | NaN | NaN | 2.43 | 1.76 | NaN |
| Q9Y613 | FH1/FH2 domain-containing protein 1 | FHOD1 | 523 | S | 1 | 51.98 | 3.30 | 2.88 | NaN | NaN | NaN | NaN | NaN | NaN | NaN |
| Q9Y6D5 | Brefeldin A-inhibited guanine nucleotide-exchange protein 2 | ARFGEF2 | 218 | S | 0.999 | 133.7 | 1.76 | 1.76 | 1.65 | 1.50 | 1.47 | 1.60 | 1.28 | 1.38 | 1.34 |
| Q9Y6D5 | Brefeldin A-inhibited guanine nucleotide-exchange protein 2 | ARFGEF2 | 227 | S | 1 | 133.7 | 1.76 | 1.76 | 1.65 | 1.50 | 1.47 | 1.60 | 1.28 | 1.38 | 1.34 |

| | | | | | | | | | | | | | | | |
|---|---|---|---|---|---|---|---|---|---|---|---|---|---|---|---|
| Q9Y6D5 | Brefeldin A-inhibited guanine nucleotide-exchange protein 2 | ARFGEF2 | 1528 | S | 1 | 133.9 | NaN | NaN | NaN | NaN | 0.81 | 1.21 | 1.34 | NaN | 1.46 |
| Q9Y6D5 | Brefeldin A-inhibited guanine nucleotide-exchange protein 2 | ARFGEF2 | 349 | S | 0.704 | 77.36 | NaN | 0.82 | NaN | NaN | NaN | NaN | 1.13 | 1.22 | NaN |
| Q9Y6I3 | Epsin-1 | EPN1 | 435 | S | 1 | 103.9 | NaN | NaN | NaN | 3.19 | 2.66 | NaN | NaN | NaN | NaN |
| Q9Y6Q9 | Nuclear receptor coactivator 3 | NCOA3 | 728 | S | 1 | 66.57 | NaN | 2.08 | 1.70 | 2.47 | 2.96 | 1.64 | 0.80 | 1.19 | 0.86 |
| Q9Y6X9 | MORC family CW-type zinc finger protein 2 | MORC2 | 615 | S | 1 | 43.12 | 0.71 | 0.54 | NaN | NaN | NaN | NaN | NaN | NaN | NaN |
| S5FZ81 | | PHF10 | 376 | S | 0.999 | 45.14 | 0.86 | NaN | NaN | NaN | 1.23 | 0.87 | 0.93 | 1.57 | NaN |
| S5FZ81 | PHD finger protein 10 | PHF10 | 297 | S | 1 | 119.9 | 0.81 | 0.58 | 0.81 | NaN | 0.89 | 0.53 | 0.71 | 0.93 | 0.77 |
| S5FZ81 | PHD finger protein 10 | PHF10 | 301 | S | 1 | 119.9 | 0.81 | 0.58 | 0.81 | NaN | 0.89 | 0.53 | 0.71 | 0.93 | 0.77 |
| E7ER32 | MKL/myocardin-like protein 1 | MKL1 | 450 | T | 0.98 | 172.9 | 1.26 | 1.45 | NaN | NaN | NaN | NaN | NaN | NaN | NaN |
| A0A087WUZ3 | Spectrin beta chain, non-erythrocytic 1 | SPTBN1 | 2161 | T | 0.806 | 107.4 | NaN | 0.65 | NaN | 0.74 | 0.87 | 0.76 | 1.12 | 1.06 | 0.96 |
| A0A087WVA8 | Testis-expressed sequence 2 protein | TEX2 | 269 | T | 0.836 | 116.8 | NaN | NaN | NaN | NaN | 1.25 | 1.35 | NaN | 0.90 | NaN |
| Q15136 | cAMP-dependent protein kinase catalytic subunit beta;cAMP-dependent protein kinase catalytic subunit gamma;cAMP-dependent protein kinase catalytic subunit | KIN27 | 181 | T | 1 | 216.2 | 1.24 | 1.00 | 1.10 | 1.29 | NaN | NaN | 0.80 | 0.87 | 1.01 |
| A0A087WW66 | 26S proteasome non-ATPase regulatory subunit 1 | PSMD1 | 311 | T | 0.998 | 84.95 | NaN | 0.95 | 0.95 | 1.04 | 1.08 | 1.22 | 1.01 | 0.98 | 1.26 |
| A0A087WW66 | 26S proteasome non-ATPase regulatory subunit 1 | PSMD1 | 273 | T | 1 | 218 | 1.72 | 1.62 | NaN | 2.09 | 2.25 | NaN | NaN | NaN | NaN |
| A0A087WWA3 | Kinesin-like protein;Kinesin-like protein KIF1B | KIF1B | 1049 | T | 0.972 | 46.76 | 0.70 | 0.64 | NaN | NaN | NaN | NaN | NaN | NaN | NaN |
| A0A087WWA3 | Kinesin-like protein;Kinesin-like protein KIF1B | KIF1B | 1426 | T | 0.804 | 69.82 | NaN | 0.48 | 0.62 | NaN | NaN | NaN | NaN | NaN | NaN |
| A0A087WWJ1 | DNA mismatch repair protein Msh6 | MSH6 | 923 | T | 0.987 | 64.04 | NaN | NaN | NaN | 1.29 | 1.28 | NaN | 0.53 | NaN | NaN |
| A0A499FJY3 | Putative RNA-binding protein 15 | RBM15 | 524 | T | 1 | 76.68 | 0.88 | 0.87 | 0.76 | 0.82 | 0.88 | 0.83 | 0.89 | 0.89 | 0.92 |
| A0A087WWY2 | | FOLR2 | 157 | T | 0.964 | 48.11 | 0.80 | 0.67 | NaN | NaN | 0.73 | NaN | 0.81 | 0.98 | NaN |
| A0A087WWY2 | | FOLR2 | 160 | T | 0.963 | 48.11 | 0.80 | 0.67 | NaN | NaN | 0.73 | NaN | 0.81 | 0.98 | NaN |
| A0A087WZ13 | Ribonucleoprotein PTB-binding 1 | RAVER1 | 463 | T | 1 | 81.79 | 1.77 | 1.88 | NaN | 1.79 | 1.84 | NaN | NaN | 1.39 | NaN |
| B7Z385 | Adenylyl cyclase-associated protein;Adenylyl cyclase-associated protein 2 | CAP2 | 199 | T | 0.711 | 121.6 | NaN | 0.48 | NaN | 0.86 | 0.66 | NaN | 0.56 | 0.54 | NaN |
| A0A087X1A5 | Double-stranded RNA-binding protein Staufen homolog 1 | STAU1 | 196 | T | 0.615 | 56.37 | 1.44 | 1.23 | 1.63 | NaN | NaN | 2.32 | NaN | NaN | NaN |
| E9PL71 | Elongation factor 1-delta | EEF1D | 123 | T | 1 | 359 | 1.18 | 1.20 | 1.12 | 1.45 | 1.45 | 1.50 | 1.91 | 1.31 | 1.35 |
| F8W7U3 | WASH complex subunit FAM21C;WASH complex subunit FAM21A | FAM21C | 331;243 | T | 1 | 98.76 | 1.10 | 1.12 | 1.31 | 1.19 | 1.00 | 1.14 | 1.01 | 1.21 | 1.10 |

| | | | | | | | | | | | | | | | | |
|---|---|---|---|---|---|---|---|---|---|---|---|---|---|---|---|---|
| Q5JSD2 | Voltage-dependent anion-selective channel protein 2 | VDAC2 | 113 | T | 0.691 | 73.31 | NaN | NaN | NaN | 0.70 | 0.70 | NaN | NaN | NaN | NaN |
| A0A0A0MRW1 | Protein FAM219A | FAM219A | 84 | T | 1 | 172.4 | 1.06 | 1.19 | NaN | 1.84 | NaN | 1.26 | NaN | 0.82 | 1.11 |
| A0A0A0MSK5 | Torsin-1A-interacting protein 1 | TOR1AIP 1 | 99 | T | 1 | 185 | 2.19 | 2.16 | NaN | NaN | NaN | NaN | 2.16 | 1.49 | NaN |
| K7EP41 | Glucose-6-phosphate isomerase | GPI | 109 | T | 0.98 | 49.24 | 1.17 | NaN | 1.12 | NaN | NaN | 0.93 | NaN | 1.13 | 1.37 |
| F8WF49 | Disks large-associated protein 4 | DLGAP4 | 221 | T | 1 | 105.8 | 1.00 | 1.36 | NaN | 1.28 | 1.67 | NaN | 1.46 | 1.25 | NaN |
| A0A2R8YCY2 | Protein furry homolog | FRY | 1920 | T | 0.522 | 47.65 | NaN | NaN | NaN | 0.79 | 0.82 | NaN | NaN | 0.86 | NaN |
| A0A0C4DH22 | Band 4.1-like protein 1 | EPB41L1 | 475 | T | 0.995 | 119.9 | NaN | NaN | 1.58 | 1.33 | 1.38 | 1.16 | 0.95 | 0.96 | 0.95 |
| Q5QPR4 | Cyclin-dependent kinase 11A;Cyclin-dependent kinase 11B | CDK11A | 702 | T | 0.542 | 98.7 | 1.25 | 1.29 | 1.60 | 1.12 | NaN | NaN | 1.13 | NaN | NaN |
| A0A0D9SEV0 | Bifunctional polynucleotide phosphatase/kinase;Polynucleotide 3'-phosphatase;Polynucleotide 5'-hydroxyl-kinase | PNKP | 118 | T | 1 | 174.5 | 0.57 | 0.63 | 0.52 | 0.64 | 0.50 | 0.62 | 0.78 | 0.61 | 0.75 |
| H7C3Z6 | Mitogen-activated protein kinase kinase kinase kinase 4 | MAP4K4 | 240 | T | 0.705 | 54.2 | NaN | NaN | NaN | NaN | NaN | NaN | NaN | 1.03 | 1.16 |
| A0A0G2JMS7 | Protein scribble homolog | SCRIB | 1468 | T | 0.937 | 101.5 | NaN | NaN | NaN | NaN | NaN | NaN | 1.44 | NaN | 1.33 |
| A0A0J9YWL0 | Absent in melanoma 1 protein | AIM1 | 933 | T | 0.813 | 72.91 | 0.58 | NaN | 0.69 | NaN | NaN | NaN | 0.75 | NaN | 1.01 |
| A0A1B0GTL5 | | | 899 | T | 0.887 | 95.07 | 1.04 | 1.05 | 1.21 | 1.13 | NaN | 1.10 | 1.28 | 1.26 | 1.23 |
| A0A1B0GUM1 | Pleckstrin homology domain-containing family A member 7 | PLEKHA7 | 91 | T | 0.541 | 92.73 | 0.51 | 1.04 | 0.69 | NaN | NaN | NaN | NaN | NaN | NaN |
| H0YN01 | Talin-2 | TLN2 | 236 | T | 1 | 94.36 | 1.42 | 0.78 | 0.73 | 0.75 | 0.74 | 0.71 | 1.13 | 1.09 | 1.05 |
| B4DP61 | Survival motor neuron protein | SMN2 | 25 | T | 0.809 | 132.5 | NaN | 2.60 | NaN | 2.50 | 1.05 | 1.04 | 0.96 | 0.99 | 0.99 |
| A0A2R8YF61 | Cyclin-dependent kinase 13 | CDK13 | 649 | T | 1 | 82.55 | 0.95 | 1.10 | 1.28 | 1.24 | 1.24 | 1.18 | 1.30 | 1.40 | 1.10 |
| A0A2R8Y4Z8 | Coiled-coil domain-containing protein 9 | CCDC9 | 389 | T | 0.714 | 98.77 | 0.82 | 0.80 | NaN | 0.80 | NaN | 0.98 | NaN | NaN | 0.78 |
| H0YH87 | Ataxin-2 | ATXN2 | 581 | T | 0.997 | 64.73 | 2.07 | NaN | 0.61 | 1.90 | 1.81 | 2.31 | 1.91 | 1.61 | 1.67 |
| H0Y470 | Girdin | CCDC88A | 690 | T | 0.592 | 230.1 | NaN | 0.66 | NaN | 0.82 | NaN | 0.77 | NaN | NaN | 0.77 |
| F8WF45 | TATA element modulatory factor | TMF1 | 401 | T | 0.789 | 139.8 | NaN | NaN | NaN | NaN | 1.08 | NaN | NaN | 1.29 | 1.08 |
| C9JBX5 | Translocon-associated protein subunit alpha | SSR1 | 192 | T | 0.644 | 62.41 | 1.90 | NaN | NaN | NaN | NaN | 2.08 | 0.95 | NaN | 0.91 |
| A0A3B3ISN0 | | | 192 | T | 0.985 | 81.77 | 2.13 | 2.28 | 2.43 | NaN | 1.10 | 1.03 | 3.04 | 3.11 | 3.13 |
| A0A3F2YNX0 | Protein transport protein Sec16A | SEC16A | 415 | T | 0.917 | 130.9 | 0.73 | NaN | NaN | 0.63 | 1.04 | NaN | 1.10 | NaN | NaN |
| A0A494BZT8 | Transforming acidic coiled-coil-containing protein 3 | TACC3 | 249 | T | 0.573 | 73.46 | NaN | 2.32 | NaN | NaN | 3.19 | 3.22 | NaN | NaN | NaN |
| D6RC77 | Phosphoacetylglucosamine mutase | PGM3 | 90 | T | 0.87 | 124.1 | NaN | NaN | 0.46 | NaN | 0.69 | 0.84 | 0.38 | NaN | NaN |
| A0A494C1F2 | Nuclear pore complex protein Nup214 | NUP214 | 437 | T | 0.947 | 91.66 | 1.39 | NaN | 1.62 | 1.59 | NaN | 1.50 | 1.70 | NaN | 1.33 |

| | | | | | | | | | | | | | | |
|---|---|---|---|---|---|---|---|---|---|---|---|---|---|---|
| Q7Z6P5 | DNA replication licensing factor MCM3 | MCM3 | 217 | T | 1 | 153.3 | 0.37 | 0.40 | 0.42 | 0.62 | 0.65 | 0.67 | 0.36 | 0.41 | NaN |
| P07355 | Annexin A2;Putative annexin A2-like protein;Annexin | ANXA2 | 19 | T | 0.992 | 220.9 | 7.38 | 6.29 | 6.83 | NaN | 11.44 | 11.86 | 0.65 | 0.65 | 0.65 |
| A9Z1X7 | Serine/arginine repetitive matrix protein 1 | SRRM1 | 802 | T | 1 | 61.1 | 0.67 | 0.70 | NaN | NaN | 0.82 | 0.85 | NaN | 1.06 | NaN |
| A9Z1X7 | Serine/arginine repetitive matrix protein 1 | SRRM1 | 220 | T | 1 | 146.3 | 0.99 | 1.01 | 0.97 | 0.95 | 0.96 | 0.96 | 1.01 | 1.00 | 1.05 |
| A9Z1X7 | Serine/arginine repetitive matrix protein 1 | SRRM1 | 401 | T | 1 | 109.5 | 1.68 | 1.63 | 1.46 | 1.30 | 1.17 | 1.36 | 1.34 | 1.37 | 1.48 |
| A9Z1X7 | Serine/arginine repetitive matrix protein 1 | SRRM1 | 881 | T | 1 | 180 | 1.88 | 1.64 | 2.18 | 1.86 | 1.43 | 1.43 | 2.55 | 1.30 | 1.94 |
| A9Z1X7 | Serine/arginine repetitive matrix protein 1 | SRRM1 | 590 | T | 1 | 65.88 | 1.15 | 1.15 | 1.31 | 1.14 | 1.19 | 1.24 | 1.24 | 1.27 | 1.32 |
| A9Z1X7 | Serine/arginine repetitive matrix protein 1 | SRRM1 | 581 | T | 1 | 68 | 1.15 | 1.25 | 1.22 | 1.10 | 1.11 | 1.10 | 1.15 | 1.17 | 1.20 |
| A9Z1X7 | Serine/arginine repetitive matrix protein 1 | SRRM1 | 583 | T | 1 | 68 | 1.15 | 1.25 | 1.22 | 1.10 | 1.11 | 1.10 | 1.15 | 1.17 | 1.20 |
| A9Z1X7 | Serine/arginine repetitive matrix protein 1 | SRRM1 | 623 | T | 1 | 106.3 | 1.24 | 1.32 | 1.34 | 0.93 | 1.18 | 1.14 | 0.90 | 1.26 | 1.27 |
| H0YCM0 | Non-specific serine/threonine protein kinase;Serine/threonine- | PAK1 | 121 | T | 0.769 | 79.36 | NaN | NaN | NaN | NaN | NaN | NaN | 0.92 | 0.87 | NaN |
| E7EX54 | Mitogen-activated protein kinase 14 | MAPK14 | 103 | T | 0.905 | 109.2 | NaN | NaN | NaN | 0.91 | 1.15 | NaN | NaN | NaN | NaN |
| B5MBZ0 | Echinoderm microtubule-associated protein-like 4 | EML4 | 910 | T | 0.995 | 130.4 | NaN | NaN | 0.85 | 0.92 | NaN | NaN | 1.21 | NaN | 1.20 |
| F8VRJ2 | Nucleosome assembly protein 1-like 1 | NAP1L1 | 21 | T | 1 | 185 | 3.75 | 3.40 | NaN | NaN | 3.56 | 2.34 | 1.11 | NaN | NaN |
| C9J1H7 | Nucleolin | NCL | 53 | T | 0.92 | 87.9 | NaN | NaN | NaN | 1.29 | 1.29 | NaN | 0.53 | 0.53 | NaN |
| C9J6P4 | Zinc finger CCCH-type antiviral protein 1 | ZC3HAV1 | 273 | T | 0.998 | 94.02 | NaN | 1.15 | NaN | 1.16 | NaN | 0.80 | NaN | NaN | NaN |
| C9JC20 | NCK-interacting protein with SH3 domain | NCKIPSD | 65 | T | 0.918 | 50.48 | NaN | NaN | NaN | NaN | NaN | NaN | 1.17 | 1.15 | NaN |
| E7EV54 | Proline-, glutamic acid- and leucine-rich protein 1 | PELP1 | 598 | T | 0.941 | 98.8 | NaN | 1.14 | 0.88 | NaN | 0.97 | NaN | 1.36 | NaN | 1.18 |
| C9JN71 | Zinc finger protein 878 | ZNF878 | 216 | T | 0.851 | 62.24 | NaN | NaN | 0.55 | 1.02 | 0.67 | 0.53 | 0.62 | 0.90 | 0.67 |
| C9JN71 | Zinc finger protein 878 | ZNF878 | 222 | T | 0.997 | 62.24 | NaN | NaN | 0.55 | 1.02 | 0.67 | 0.53 | 0.62 | 0.90 | 0.67 |
| C9K0J5 | Ras-associated and pleckstrin homology domains-containing protein 1 | RAPH1 | 1205 | T | 0.623 | 77.52 | NaN | 1.77 | NaN | 1.84 | 1.49 | NaN | 0.86 | 0.98 | 0.84 |
| H0Y3P2 | Eukaryotic translation initiation factor 4 gamma 2 | EIF4G2 | 468 | T | 0.713 | 89.91 | NaN | NaN | NaN | 1.01 | NaN | 1.26 | 1.32 | 1.37 | NaN |
| H0Y3P2 | Eukaryotic translation initiation factor 4 gamma 2 | EIF4G2 | 470 | T | 1 | 117.4 | NaN | NaN | 1.17 | 1.13 | 1.12 | 1.25 | 1.38 | 1.23 | 1.27 |

| | | | | | | | | | | | | | | |
|---|---|---|---|---|---|---|---|---|---|---|---|---|---|---|
| H0Y9J2 | Calcium/calmodulin-dependent protein kinase type II subunit delta | CAMK2D | 41 | T | 0.988 | 337.4 | 0.66 | 0.57 | NaN | NaN | NaN | 0.88 | 0.71 | NaN | 0.62 |
| D6REC6 | Cyclin-dependent kinase 7 | CDK7 | 77 | T | 0.999 | 65.84 | 0.66 | NaN | 0.53 | 0.89 | 0.89 | 0.83 | 0.58 | 0.70 | 0.80 |
| D6RCF2 | Histone H2A;Core histone macro-H2A.1 | H2AFY | 129 | T | 1 | 58.67 | NaN | 1.71 | NaN | 3.88 | 3.61 | NaN | NaN | NaN | NaN |
| E3W994 | CLIP-associating protein 2 | CLASP2 | 573 | T | 0.677 | 96.81 | 1.76 | 2.06 | 1.62 | 1.92 | NaN | NaN | 1.26 | 1.83 | 1.63 |
| E5RHT6 | ADP-ribosylation factor GTPase-activating protein 1 | ARFGAP1 | 61 | T | 1 | 67.99 | NaN | 1.71 | 1.78 | 1.36 | 1.86 | NaN | NaN | 2.80 | NaN |
| E7ERH2 | S-phase kinase-associated protein 1 | SKP1 | 131 | T | 1 | 58.34 | 0.71 | NaN | 0.96 | NaN | NaN | NaN | 1.24 | 1.13 | 1.19 |
| E7EV99 | Alpha-adducin | ADD1 | 11 | T | 0.586 | 67.9 | 1.33 | 2.66 | NaN | 3.03 | 2.74 | NaN | NaN | 0.76 | NaN |
| E7EPK0 | LIM and calponin homology domains-containing protein 1 | LIMCH1 | 513 | T | 0.587 | 70.41 | 0.52 | 0.62 | NaN | NaN | NaN | 0.82 | 0.91 | 1.16 | 0.64 |
| G3V1Q4 | Septin-7 | 7-Sep | 192 | T | 1 | 76.49 | 1.01 | 1.03 | 1.10 | 1.13 | 1.33 | 0.94 | 0.94 | 0.75 | 1.15 |
| E7EPN9 | Protein PRRC2C | PRRC2C | 2675 | T | 1 | 126.3 | 3.68 | 3.80 | 4.05 | 2.84 | NaN | 2.67 | NaN | 2.41 | 2.35 |
| E7EPN9 | Protein PRRC2C | PRRC2C | 2684 | T | 0.996 | 67.06 | NaN | 3.56 | 3.34 | NaN | NaN | 2.20 | 2.38 | 2.06 | 2.34 |
| S4R3H4 | Apoptotic chromatin condensation inducer in the nucleus | ACIN1 | 356 | T | 0.995 | 94.38 | NaN | 0.83 | 1.32 | 1.25 | 1.14 | 1.27 | 1.06 | 0.91 | 1.08 |
| E7ESS4 | Intercellular adhesion molecule 1 | ICAM1 | 308 | T | 0.999 | 73.93 | 0.67 | 0.59 | 0.70 | 1.58 | NaN | 1.06 | NaN | 0.53 | NaN |
| E7EX17 | Eukaryotic translation initiation factor 4B | EIF4B | 97 | T | 0.739 | 69.15 | NaN | 1.41 | 1.41 | NaN | NaN | 1.34 | NaN | 1.26 | NaN |
| E7EX44 | Caldesmon | CALD1 | 552 | T | 0.794 | 56.75 | 0.99 | 1.11 | 1.06 | NaN | 1.12 | 1.26 | 1.05 | 1.03 | NaN |
| F8VZJ2 | Nascent polypeptide-associated complex subunit alpha;Nascent polypeptide-associated complex subunit alpha, muscle-specific form | NACA | 82 | T | 1 | 217.1 | 1.20 | 1.09 | 1.16 | 1.03 | 1.02 | 1.05 | 1.08 | NaN | 1.11 |
| E9PFD7 | Receptor protein-tyrosine kinase;Epidermal growth factor receptor | EGFR | 648 | T | 0.996 | 121.4 | NaN | NaN | NaN | NaN | NaN | NaN | 2.75 | NaN | 2.86 |
| E9PHI6 | Cytoplasmic dynein 1 light intermediate chain 1 | DYNC1LI1 | 396 | T | 0.822 | 154.3 | NaN | NaN | 1.27 | 0.64 | NaN | 1.18 | 0.95 | NaN | 0.93 |
| E9PHI6 | Cytoplasmic dynein 1 light intermediate chain 1 | DYNC1LI1 | 397 | T | 0.985 | 147.5 | 0.94 | 0.91 | 1.57 | 1.70 | NaN | 2.29 | NaN | 0.76 | 0.82 |
| E9PHI6 | Cytoplasmic dynein 1 light intermediate chain 1 | DYNC1LI1 | 399 | T | 0.968 | 176.1 | NaN | 0.91 | NaN | 1.70 | 0.80 | NaN | NaN | NaN | NaN |
| E9PK52 | Band 4.1-like protein 2 | EPB41L2 | 600 | T | 0.531 | 58.07 | NaN | NaN | NaN | 2.04 | 2.11 | NaN | 0.86 | 0.96 | NaN |
| E9PI52 | Arginine/serine-rich coiled-coil protein 2 | RSRC2 | 16 | T | 0.772 | 79.01 | 0.83 | 0.95 | 1.04 | 0.97 | 1.10 | 0.81 | 1.29 | 0.88 | 1.41 |
| E9PSF4 | 40S ribosomal protein S3 | RPS3 | 95 | T | 0.996 | 167.9 | 1.74 | 1.96 | 2.35 | 2.12 | 1.77 | 2.17 | 2.05 | 1.77 | 2.01 |
| E9PMG1 | RalBP1-associated Eps domain-containing protein 1 | REPS1 | 173 | T | 0.722 | 132.6 | NaN | NaN | 0.75 | NaN | 1.70 | 0.77 | NaN | NaN | 0.84 |
| E9PMS6 | LIM domain only protein 7 | LMO7 | 555 | T | 0.839 | 187.1 | 2.08 | 2.09 | 2.13 | 2.13 | 2.18 | NaN | 1.43 | 1.18 | 1.55 |
| E9PMS6 | LIM domain only protein 7 | LMO7 | 613 | T | 1 | 68.84 | NaN | 1.07 | 0.85 | NaN | 1.14 | 1.47 | 0.64 | 0.73 | 0.71 |

| | | | | | | | | | | | | | | | |
|---|---|---|---|---|---|---|---|---|---|---|---|---|---|---|---|
| E9PS23 | Cofilin-1 | CFL1 | 25 | T | 0.997 | 128.9 | 2.66 | 2.75 | 2.32 | 4.77 | NaN | 4.42 | 0.93 | NaN | 0.92 |
| F5H8D7 | DNA repair protein XRCC1 | XRCC1 | 422 | T | 0.999 | 115.3 | 0.51 | 0.54 | 0.61 | 0.75 | 0.69 | 0.70 | 0.90 | 0.87 | 0.93 |
| F8W6N3 | Ubiquitin carboxyl-terminal hydrolase;Ubiquitin carboxyl-terminal hydrolase BAP1 | BAP1 | 499 | T | 0.876 | 63.23 | 1.01 | 0.69 | NaN | NaN | NaN | 0.79 | 0.76 | 0.78 | NaN |
| P42166 | Lamina-associated polypeptide 2, isoforms beta/gamma;Thymopoietin;Thymopentin;Lamina-associated polypeptide 2, isoform alpha;Thymopoietin;Thymopentin | TMPO | 74;74 | T | 1 | 159.8 | 1.56 | 2.60 | 2.75 | 2.45 | 2.58 | 1.02 | 2.88 | 0.70 | 2.86 |
| H0Y4V9 | La-related protein 4B | LARP4B | 84 | T | 0.953 | 107.2 | 1.63 | 1.35 | 1.22 | 1.06 | NaN | 1.18 | 1.50 | 0.98 | 1.68 |
| H0Y4Z8 | Rab-like protein 6 | RABL6 | 600 | T | 1 | 150.5 | 1.19 | 1.15 | 1.46 | 1.34 | 1.06 | 1.09 | 1.17 | 1.36 | 1.28 |
| H0Y4Z8 | Rab-like protein 6 | RABL6 | 469 | T | 0.967 | 60.62 | 1.51 | 0.54 | NaN | 0.65 | 1.35 | 1.58 | 1.10 | 0.49 | 1.23 |
| H0Y5S9 | Casein kinase I isoform | CSNK1E | 65 | T | 0.5 | 88.16 | 0.79 | NaN | NaN | 0.97 | 0.79 | NaN | 1.06 | 1.00 | 1.01 |
| H0Y6J7 | | PLXNB2 | 185 | T | 0.993 | 56.92 | 0.97 | 0.91 | 0.91 | 0.87 | 0.91 | NaN | 1.04 | 1.06 | 1.08 |
| H7BZT5 | Zinc finger protein 185 | ZNF185 | 206;450 | T | 0.935 | 187.5 | 0.92 | 0.88 | 0.87 | 0.92 | 0.91 | 0.80 | 1.38 | 1.39 | 1.24 |
| H7BZT5 | Zinc finger protein 185 | ZNF185 | 272;516 | T | 0.963 | 199.6 | NaN | 1.08 | 0.89 | NaN | NaN | 0.87 | NaN | NaN | 1.45 |
| H0YE35 | Synembryn-A | RIC8A | 49 | T | 0.989 | 243.2 | 0.93 | 1.03 | 0.95 | 1.15 | 0.74 | 1.11 | 1.02 | 1.06 | 1.00 |
| J3KPH4 | Sorting nexin-1 | SNX1 | 41 | T | 1 | 198.4 | 0.69 | 0.64 | 1.02 | 0.75 | 1.09 | 0.79 | 0.82 | 1.12 | 0.83 |
| H3BN50 | | SLC9A3R2 | 8 | T | 1 | 152.7 | 2.12 | 1.93 | 2.11 | 1.43 | 1.46 | 1.41 | 2.07 | NaN | 2.14 |
| H3BRW3 | Sulfhydryl oxidase;FAD-linked sulfhydryl oxidase ALR | GFER | 79 | T | 1 | 56.12 | NaN | 0.75 | 0.77 | 0.90 | NaN | NaN | 1.23 | 0.86 | 1.15 |
| J3QLT7 | Nucleolar protein 3 | NOL3 | 72 | T | 0.995 | 186.2 | 1.24 | 0.70 | 0.85 | NaN | 0.98 | 1.03 | 1.35 | 1.15 | 1.11 |
| H7BZT5 | Zinc finger protein 185 | ZNF185 | 158 | T | 0.998 | 184.4 | 1.69 | 1.40 | 1.89 | 1.41 | 1.44 | 1.27 | 2.41 | 2.96 | 2.38 |
| H7C128 | | BRD8 | 255 | T | 1 | 85.53 | 0.73 | 0.61 | NaN | 1.06 | 0.86 | NaN | 1.06 | 0.82 | 0.88 |
| I3L467 | Unconventional prefoldin RPB5 interactor 1 | URI1 | 5 | T | 0.838 | 46.98 | NaN | NaN | NaN | 0.89 | NaN | NaN | 0.60 | 0.82 | NaN |
| J3QLL0 | Importin subunit alpha-1 | KPNA2 | 61 | T | 0.517 | 135.4 | NaN | NaN | NaN | NaN | NaN | 4.44 | 0.76 | 0.67 | 0.84 |
| J3QK89 | Calcium homeostasis endoplasmic reticulum protein | CHERP | 830 | T | 0.972 | 107 | 0.85 | 1.03 | 1.06 | NaN | 0.87 | NaN | 1.13 | 1.07 | 1.13 |
| J3QSD7 | Cyclin-dependent kinase 12 | CDK12 | 691 | T | 1 | 58.68 | 0.68 | NaN | 0.78 | 0.62 | 0.96 | 0.76 | 0.84 | 0.73 | 0.82 |
| K4DI93 | Cullin-4B | CUL4B | 36 | T | 0.73 | 76.05 | NaN | 0.70 | NaN | 1.22 | 1.11 | 1.29 | 0.71 | 0.61 | NaN |
| K7ELG9 | Protein LSM12 homolog | LSM12 | 75 | T | 1 | 72.89 | 1.19 | 1.30 | NaN | 1.30 | 0.97 | NaN | NaN | 2.35 | NaN |
| K7EMR2 | Choline transporter-like protein 2 | SLC44A2 | 14 | T | 1 | 74.79 | 0.63 | 0.70 | 0.58 | 0.81 | NaN | 0.69 | 0.70 | 0.91 | NaN |
| M0QXD6 | General transcription factor IIF subunit 1 | GTF2F1 | 305 | T | 0.929 | 90.96 | NaN | 2.18 | 6.35 | NaN | NaN | NaN | NaN | NaN | NaN |
| M0QXD6 | General transcription factor IIF subunit 1 | GTF2F1 | 247 | T | 1 | 68.13 | NaN | 0.82 | 0.73 | 0.98 | 1.00 | 1.03 | 0.94 | 0.91 | 0.97 |
| M0QZ09 | NAD-dependent protein deacetylase sirtuin-6 | SIRT6 | 220 | T | 1 | 66.6 | 1.30 | 1.10 | 1.15 | 1.17 | 0.96 | 1.10 | 1.03 | 0.86 | 1.09 |
| O00267 | Transcription elongation factor SPT5 | SUPT5H | 1034 | T | 0.981 | 69.76 | NaN | NaN | 1.81 | 3.84 | NaN | NaN | 0.64 | 0.95 | NaN |

| | | | | | | | | | | | | | | |
|---|---|---|---|---|---|---|---|---|---|---|---|---|---|---|
| O15173 | Membrane-associated progesterone receptor component 2 | PGRMC2 | 211 | T | 1 | 311.2 | 0.93 | 0.84 | 0.89 | 0.98 | 0.93 | 1.00 | 1.20 | 1.20 | 1.19 |
| O43379 | WD repeat-containing protein 62 | WDR62 | 1148 | T | 0.827 | 60.01 | NaN | NaN | 0.37 | NaN | 0.64 | NaN | NaN | 0.67 | 0.67 |
| O43379 | WD repeat-containing protein 62 | WDR62 | 1053 | T | 0.693 | 52.96 | NaN | NaN | 0.59 | NaN | NaN | NaN | NaN | 0.65 | 0.80 |
| O43493 | Trans-Golgi network integral membrane protein 2 | TGOLN2 | 296 | T | 0.788 | 152.6 | NaN | 1.40 | NaN | NaN | 2.11 | 1.33 | NaN | 1.28 | NaN |
| O43765 | Small glutamine-rich tetratricopeptide repeat-containing protein alpha | SGTA | 81 | T | 0.989 | 121.9 | 0.64 | 0.87 | 0.77 | 1.25 | 1.16 | 1.08 | 0.91 | 1.22 | 0.95 |
| O43765 | Small glutamine-rich tetratricopeptide repeat-containing protein alpha | SGTA | 303 | T | 0.525 | 239.4 | 1.45 | 1.29 | NaN | NaN | NaN | NaN | NaN | NaN | NaN |
| O60271 | C-Jun-amino-terminal kinase-interacting protein 4 | SPAG9 | 217 | T | 1 | 130.1 | 3.06 | 3.42 | NaN | 2.45 | 3.05 | NaN | 3.31 | 3.75 | NaN |
| O60292 | Signal-induced proliferation-associated 1-like protein 3 | SIPA1L3 | 1531 | T | 0.5 | 48.33 | NaN | 0.97 | 0.81 | 1.09 | NaN | NaN | NaN | NaN | NaN |
| O60832 | H/ACA ribonucleoprotein complex subunit 4 | DKC1 | 458 | T | 1 | 45.94 | NaN | 0.92 | 1.00 | NaN | 0.97 | 0.97 | NaN | 0.99 | NaN |
| O75475 | PC4 and SFRS1-interacting protein | PSIP1 | 272 | T | 0.845 | 271.3 | NaN | NaN | 0.50 | NaN | NaN | NaN | NaN | 0.73 | 0.63 |
| O95674 | Phosphatidate cytidylyltransferase 2 | CDS2 | 31 | T | 0.58 | 138.9 | NaN | 1.11 | NaN | 1.01 | 0.91 | 1.02 | 0.84 | 0.75 | NaN |
| O95684 | FGFR1 oncogene partner | FGFR1OP | 170 | T | 0.895 | 64.52 | NaN | NaN | NaN | 3.03 | 3.10 | NaN | NaN | NaN | NaN |
| O95817 | BAG family molecular chaperone regulator 3 | BAG3 | 406 | T | 1 | 87.28 | 1.35 | 1.01 | 1.36 | 2.10 | 2.30 | 2.27 | 0.75 | 0.82 | 0.83 |
| P07948 | Tyrosine-protein kinase Lyn;Non-specific protein-tyrosine kinase;Tyrosine-protein kinase HCK | LYN | 398 | T | 0.605 | 76.22 | 0.55 | NaN | 0.47 | 0.30 | 0.36 | 0.33 | 0.95 | 0.64 | NaN |
| P08670 | Vimentin | VIM | 336 | T | 0.897 | 94.03 | NaN | NaN | 1.16 | 1.03 | 1.09 | NaN | 1.27 | 1.18 | 1.02 |
| P12931 | Proto-oncogene tyrosine-protein kinase Src;Non-specific protein-tyrosine kinase;Tyrosine-protein kinase Lck;Tyrosine-protein kinase Fyn;Tyrosine-protein kinase Yes | SRC | 420 | T | 0.759 | 119.8 | NaN | 0.81 | 0.77 | 0.76 | 0.80 | 0.71 | 1.21 | 1.05 | NaN |
| P13639 | Elongation factor 2 | EEF2 | 435 | T | 1 | 82.58 | 0.93 | 0.89 | 0.87 | 1.50 | 1.70 | 1.65 | 0.67 | 0.64 | 0.63 |
| P16144 | Integrin beta-4 | ITGB4 | 1530 | T | 0.993 | 124.5 | 1.00 | 0.99 | 1.11 | 1.47 | 1.40 | 1.27 | 1.31 | 1.40 | 1.39 |
| P17252 | Protein kinase C alpha type;Protein kinase C beta type;Protein kinase C gamma type | PRKCA | 501 | T | 0.587 | 88.79 | 0.62 | 0.60 | NaN | NaN | NaN | 0.58 | 1.09 | 1.11 | NaN |

| | | | | | | | | | | | | | | | | |
|---|---|---|---|---|---|---|---|---|---|---|---|---|---|---|---|---|
| P17275 | Transcription factor jun-B | JUNB | 255 | T | 1 | 140.3 | 4.31 | NaN | NaN | 0.99 | NaN | 1.22 | NaN | 5.63 | 5.63 |
| P18858 | DNA ligase 1;DNA ligase | LIG1 | 195 | T | 0.967 | 128.7 | 0.49 | 0.54 | NaN | NaN | 0.81 | NaN | NaN | NaN | NaN |
| P26641 | Elongation factor 1-gamma | EEF1G | 43 | T | 0.837 | 61.37 | 0.93 | 0.98 | NaN | NaN | NaN | NaN | NaN | NaN | NaN |
| P27816 | Microtubule-associated protein 4;Microtubule-associated protein | MAP4 | 512 | T | 0.59 | 117.2 | 0.55 | 0.76 | NaN | NaN | 1.65 | 1.81 | NaN | NaN | 0.68 |
| P27816 | Microtubule-associated protein 4;Microtubule-associated protein | MAP4 | 521 | T | 1 | 279.1 | 0.65 | 0.65 | 0.66 | 0.85 | 0.85 | 0.90 | 0.78 | 1.10 | 0.80 |
| P27816 | Microtubule-associated protein 4 | MAP4 | 687 | T | 0.956 | 72.37 | NaN | NaN | NaN | NaN | NaN | 0.53 | 0.83 | 0.81 | 1.08 |
| P27824 | Calnexin | CANX | 562 | T | 1 | 187.3 | 1.11 | 1.11 | 0.81 | 0.81 | 1.44 | 1.08 | 1.06 | 0.96 | 0.82 |
| P29966 | Myristoylated alanine-rich C-kinase substrate | MARCKS | 143 | T | 1 | 213 | 1.04 | 1.42 | 1.15 | 2.23 | 2.69 | 1.67 | 1.05 | 1.07 | 0.75 |
| P29966 | Myristoylated alanine-rich C-kinase substrate | MARCKS | 150 | T | 1 | 301.2 | 1.97 | 2.17 | 2.35 | 1.82 | 1.93 | 1.93 | 2.38 | 3.47 | 2.51 |
| P29966 | Myristoylated alanine-rich C-kinase substrate | MARCKS | 120 | T | 0.731 | 170.9 | 2.78 | 2.75 | NaN | NaN | 0.76 | NaN | NaN | NaN | NaN |
| P29966 | Myristoylated alanine-rich C-kinase substrate | MARCKS | 133 | T | 0.671 | 91.1 | NaN | 1.14 | 1.39 | 0.68 | NaN | NaN | NaN | NaN | NaN |
| P36507 | Dual specificity mitogen-activated protein kinase kinase 2 | MAP2K2 | 394 | T | 1 | 144.6 | 1.24 | 1.31 | 1.24 | NaN | NaN | 1.17 | 1.50 | 1.43 | 1.54 |
| P41743 | Protein kinase C iota type | PRKCI | 564 | T | 1 | 186.1 | 0.56 | 0.94 | 0.88 | 0.75 | 0.77 | 0.76 | 0.82 | 0.89 | 0.77 |
| P46087 | Probable 28S rRNA (cytosine(4447)-C(5))-methyltransferase | NOP2 | 784 | T | 0.637 | 68.52 | 1.44 | NaN | 1.33 | NaN | NaN | 1.97 | NaN | NaN | NaN |
| P46821 | Microtubule-associated protein 1B;MAP1B heavy chain;MAP1 light chain LC1 | MAP1B | 1067 | T | 0.97 | 102.2 | 1.10 | 1.31 | 1.42 | 2.20 | 2.41 | 2.38 | 1.37 | NaN | 1.25 |
| P46821 | Microtubule-associated protein 1B;MAP1B heavy chain;MAP1 light chain LC1 | MAP1B | 2305 | T | 0.73 | 43.81 | NaN | NaN | NaN | NaN | NaN | 4.40 | 0.69 | NaN | 1.00 |
| P46821 | Microtubule-associated protein 1B;MAP1B heavy chain;MAP1 light chain LC1 | MAP1B | 704 | T | 1 | 51.01 | NaN | 0.48 | NaN | NaN | 0.67 | NaN | 0.80 | 0.79 | 0.80 |
| P46821 | Microtubule-associated protein 1B;MAP1B heavy chain;MAP1 light chain LC1 | MAP1B | 1503 | T | 0.567 | 104.8 | NaN | 0.71 | NaN | 0.85 | 0.91 | NaN | NaN | 0.99 | NaN |
| P46821 | Microtubule-associated protein 1B;MAP1B heavy chain;MAP1 light chain LC1 | MAP1B | 1788 | T | 1 | 134.8 | 1.41 | 1.38 | 1.51 | 1.42 | 1.32 | 1.32 | 1.98 | 1.74 | 2.13 |
| P46821 | Microtubule-associated protein 1B;MAP1B heavy chain;MAP1 light chain LC1 | MAP1B | 1282 | T | 1 | 122.1 | 2.11 | 1.79 | 2.11 | 3.51 | 3.23 | 3.00 | 0.81 | 1.06 | 0.76 |

| | | | | | | | | | | | | | | | | |
|---|---|---|---|---|---|---|---|---|---|---|---|---|---|---|---|---|
| P46821 | Microtubule-associated protein 1B;MAP1B heavy chain;MAP1 light chain LC1 | MAP1B | 1913 | T | 0.748 | 169.1 | 0.73 | 0.74 | 0.77 | 0.91 | NaN | 0.85 | 0.89 | NaN | 0.91 |
| P46821 | Microtubule-associated protein 1B;MAP1B heavy chain;MAP1 light chain LC1 | MAP1B | 1930 | T | 0.541 | 139.8 | 1.08 | 0.94 | NaN | NaN | NaN | 1.03 | 0.74 | NaN | NaN |
| P46821 | Microtubule-associated protein 1B;MAP1B heavy chain;MAP1 light chain LC1 | MAP1B | 1932 | T | 0.985 | 125.4 | 1.19 | NaN | NaN | NaN | NaN | NaN | 0.82 | NaN | 0.83 |
| P46821 | Microtubule-associated protein 1B;MAP1B heavy chain;MAP1 light chain LC1 | MAP1B | 1947 | T | 0.67 | 143.5 | 0.78 | NaN | NaN | NaN | NaN | NaN | 1.19 | NaN | 1.05 |
| P46937 | Transcriptional coactivator YAP1 | YAP1 | 63 | T | 0.552 | 222.8 | NaN | NaN | 0.83 | NaN | 1.06 | 0.91 | NaN | NaN | 0.66 |
| P48634 | Protein PRRC2A | PRRC2A | 610 | T | 0.828 | 71.65 | 1.65 | NaN | 1.60 | 1.78 | 1.89 | 1.69 | 1.29 | 1.44 | 1.25 |
| P49006 | MARCKS-related protein | MARCKSL1 | 148 | T | 0.999 | 101.4 | 0.98 | 0.83 | 0.83 | 0.82 | 1.01 | 0.73 | 0.80 | 0.69 | 0.63 |
| P49585 | Choline-phosphate cytidylyltransferase A | PCYT1A | 342 | T | 0.847 | 57.2 | NaN | NaN | NaN | NaN | NaN | NaN | 0.83 | NaN | 0.58 |
| P51116 | Fragile X mental retardation syndrome-related protein 2 | FXR2 | 598 | T | 0.992 | 109.7 | 1.57 | 1.50 | NaN | 0.95 | NaN | NaN | 1.55 | NaN | NaN |
| P52732 | Kinesin-like protein KIF11 | KIF11 | 926 | T | 0.767 | 78.9 | 1.38 | 1.44 | 1.31 | NaN | NaN | 2.54 | 0.53 | NaN | 0.62 |
| P55081 | Microfibrillar-associated protein 1 | MFAP1 | 267 | T | 1 | 281.1 | 0.95 | 1.20 | 1.18 | 0.94 | 1.14 | 0.99 | 1.20 | 1.13 | 1.16 |
| P80723 | Brain acid soluble protein 1 | BASP1 | 36 | T | 1 | 241.5 | NaN | 1.17 | NaN | 0.50 | 0.55 | NaN | 0.54 | 0.43 | NaN |
| P80723 | Brain acid soluble protein 1 | BASP1 | 196 | T | 0.929 | 192.4 | 2.53 | NaN | NaN | 3.54 | 3.07 | NaN | 2.48 | NaN | NaN |
| P85037 | Forkhead box protein K1 | FOXK1 | 436 | T | 0.999 | 69.46 | 1.75 | 1.84 | NaN | 1.56 | NaN | NaN | NaN | NaN | NaN |
| P85037 | Forkhead box protein K1 | FOXK1 | 245 | T | 0.687 | 83.25 | NaN | NaN | NaN | 1.33 | 0.84 | NaN | NaN | NaN | NaN |
| Q01130 | Serine/arginine-rich splicing factor 2 | SRSF2 | 22 | T | 0.774 | 89.98 | 0.94 | NaN | NaN | NaN | NaN | 1.15 | 0.64 | NaN | 0.88 |
| Q03111 | Protein ENL | MLLT1 | 314 | T | 0.836 | 85.62 | NaN | NaN | NaN | 0.93 | NaN | NaN | 0.97 | NaN | 0.87 |
| Q05209 | Tyrosine-protein phosphatase non-receptor type 12 | PTPN12 | 509 | T | 0.915 | 92.55 | NaN | 0.79 | NaN | NaN | NaN | NaN | 1.05 | NaN | 1.09 |
| Q09666 | Neuroblast differentiation-associated protein AHNAK | AHNAK | 5729 | T | 0.67 | 84.38 | NaN | NaN | NaN | 1.00 | 1.11 | NaN | 0.82 | 0.84 | NaN |
| Q09666 | Neuroblast differentiation-associated protein AHNAK | AHNAK | 5839 | T | 0.738 | 108.7 | NaN | 0.89 | 0.83 | 0.72 | 0.87 | NaN | 0.81 | 1.13 | 1.01 |
| Q09666 | Neuroblast differentiation-associated protein AHNAK | AHNAK | 490 | T | 1 | 120.9 | 0.61 | 0.75 | 0.68 | 1.15 | 1.27 | 1.17 | 0.64 | 0.60 | 0.68 |
| Q12906 | Interleukin enhancer-binding factor 3 | ILF3 | 486 | T | 0.545 | 73.39 | 0.52 | NaN | 0.81 | NaN | NaN | 0.76 | 1.05 | 0.96 | 0.97 |
| Q12906 | Interleukin enhancer-binding factor 3 | ILF3 | 592 | T | 1 | 133.2 | 2.88 | 2.63 | 2.52 | 4.67 | 5.09 | 4.21 | 0.76 | 0.75 | 0.76 |
| Q13177 | Serine/threonine-protein kinase PAK 2;PAK-2p27;PAK- | PAK2 | 169 | T | 1 | 166.9 | 2.25 | 1.71 | 2.19 | 1.91 | 1.86 | 1.46 | 1.77 | 1.70 | NaN |

| | | | | | | | | | | | | | | |
|---|---|---|---|---|---|---|---|---|---|---|---|---|---|---|
| Q13443 | Disintegrin and metalloproteinase domain-containing protein 9 | ADAM9 | 761 | T | 1 | 73.41 | 2.24 | NaN | NaN | NaN | 2.76 | 2.66 | NaN | NaN | NaN |
| Q13469 | Nuclear factor of activated T-cells, cytoplasmic 2 | NFATC2 | 325 | T | 0.678 | 113.2 | 1.44 | 0.98 | NaN | NaN | NaN | NaN | 1.67 | NaN | 1.55 |
| Q13501 | Sequestosome-1 | SQSTM1 | 269 | T | 1 | 189.4 | 1.17 | 1.42 | 1.39 | 1.75 | 1.49 | 1.40 | 0.67 | 0.58 | 0.80 |
| Q13541 | Eukaryotic translation initiation factor 4E-binding protein 1 | EIF4EBP1 | 68 | T | 0.882 | 152.9 | 0.84 | 0.71 | NaN | 2.03 | 2.61 | 2.44 | 1.84 | 1.44 | 1.88 |
| Q13541 | Eukaryotic translation initiation factor 4E-binding protein 1 | EIF4EBP1 | 70 | T | 0.757 | 98.69 | 2.91 | 2.68 | 2.72 | 2.12 | 2.38 | 2.33 | 0.72 | 0.83 | 2.30 |
| Q13541 | Eukaryotic translation initiation factor 4E-binding protein 1 | EIF4EBP1 | 37 | T | 0.883 | 106.9 | 2.50 | 2.34 | 2.68 | 2.04 | NaN | 2.08 | 2.60 | NaN | 2.26 |
| Q13541 | Eukaryotic translation initiation factor 4E-binding protein 1 | EIF4EBP1 | 46 | T | 0.951 | 106.9 | 2.40 | 1.93 | 2.68 | 2.01 | 1.91 | 2.10 | NaN | NaN | 2.26 |
| Q13542 | Eukaryotic translation initiation factor 4E-binding protein 2 | EIF4EBP2 | 36 | T | 0.736 | 79.87 | NaN | 3.40 | NaN | NaN | NaN | 2.17 | 3.25 | 2.70 | NaN |
| Q13595 | Transformer-2 protein homolog alpha | TRA2A | 88 | T | 0.997 | 68.37 | 1.08 | 0.50 | 0.39 | 0.53 | 0.83 | 0.94 | 1.32 | 1.24 | 1.25 |
| Q14247 | Src substrate cortactin | CTTN | 399 | T | 0.633 | 101.8 | 1.15 | 1.11 | 1.22 | 2.74 | 2.95 | 1.02 | 1.36 | 1.08 | 1.43 |
| Q14247 | Src substrate cortactin | CTTN | 401 | T | 1 | 252.6 | 1.11 | 0.97 | 0.95 | 1.14 | 1.22 | 0.99 | 1.31 | 1.41 | 1.32 |
| Q15648 | Mediator of RNA polymerase II transcription subunit 1 | MED1 | 1051 | T | 0.993 | 64.41 | 0.81 | 0.69 | 0.79 | 0.84 | 1.01 | 0.62 | 1.27 | 1.46 | 0.95 |
| Q15678 | Tyrosine-protein phosphatase non-receptor type 14 | PTPN14 | 309 | T | 0.509 | 48.22 | NaN | NaN | NaN | NaN | 0.86 | NaN | NaN | 1.47 | 1.30 |
| Q15910 | Histone-lysine N-methyltransferase EZH2 | EZH2 | 487 | T | 1 | 175.8 | 0.54 | 0.54 | 0.67 | 1.02 | 0.70 | 0.69 | 0.96 | 0.82 | 0.87 |
| Q16513 | Serine/threonine-protein kinase N2 | PKN2 | 958 | T | 1 | 106.5 | NaN | NaN | 0.88 | 0.89 | 1.24 | 1.26 | 0.91 | 1.00 | NaN |
| Q1RLN5 | Rho GTPase-activating protein 12 | ARHGAP 12 | 230 | T | 0.603 | 117.9 | NaN | NaN | NaN | 0.88 | 0.93 | 1.14 | NaN | 0.83 | 0.87 |
| Q1RLN5 | Rho GTPase-activating protein 12 | ARHGAP 12 | 231 | T | 0.965 | 79.29 | 0.84 | 0.85 | 0.80 | NaN | 0.93 | 1.46 | 0.86 | 0.83 | NaN |
| Q27J81 | Inverted formin-2 | INF2 | 1148 | T | 0.663 | 88.74 | 1.27 | 1.13 | NaN | 0.66 | NaN | NaN | NaN | 1.19 | NaN |
| Q2M2I8 | AP2-associated protein kinase 1 | AAK1 | 620 | T | 1 | 131.1 | 0.51 | 0.42 | 1.41 | 0.60 | 0.56 | 1.34 | 0.56 | 0.57 | 0.55 |
| Q2M2I8 | AP2-associated protein kinase 1 | AAK1 | 606 | T | 0.966 | 98.37 | 0.61 | 0.60 | NaN | 0.94 | NaN | 0.94 | NaN | NaN | 0.88 |
| Q32MZ4 | Leucine-rich repeat flightless-interacting protein 1 | LRRFIP1 | 123 | T | 0.869 | 114.5 | NaN | 1.14 | 1.26 | NaN | NaN | NaN | NaN | NaN | NaN |
| Q3KQU3 | MAP7 domain-containing protein 1 | MAP7D1 | 97 | T | 0.6 | 46.96 | 1.03 | 0.98 | 0.87 | 0.95 | 1.03 | 1.23 | 0.90 | NaN | NaN |
| Q4G0J3 | La-related protein 7 | LARP7 | 338 | T | 1 | 115.3 | 1.07 | 1.03 | 0.93 | NaN | 1.20 | 0.91 | 1.31 | 1.14 | 0.97 |
| Q4KMP7 | TBC1 domain family member 10B | TBC1D10 B | 148 | T | 0.995 | 99.87 | 1.50 | 1.87 | NaN | NaN | NaN | NaN | 4.15 | NaN | NaN |
| Q4VCS5 | Angiomotin | AMOT | 12 | T | 0.691 | 61.17 | 0.83 | NaN | 0.87 | 0.70 | 0.78 | 0.74 | 0.90 | 0.94 | NaN |
| Q5VZS7 | Programmed cell death protein 4 | PDCD4 | 79 | T | 0.873 | 155.2 | 0.76 | 0.75 | NaN | 0.84 | NaN | NaN | 1.04 | NaN | NaN |

| | | | | | | | | | | | | | | | |
|---|---|---|---|---|---|---|---|---|---|---|---|---|---|---|---|
| Q53TN4 | Cytochrome b reductase 1 | CYBRD1 | 285 | T | 0.737 | 172.6 | NaN | 0.40 | NaN | 0.70 | NaN | 0.76 | NaN | NaN | NaN |
| Q58WW2 | DDB1- and CUL4-associated factor 6 | DCAF6 | 654 | T | 0.999 | 76.2 | 0.61 | 0.62 | 0.62 | NaN | NaN | NaN | NaN | 0.98 | NaN |
| Q5JTD0 | Tight junction-associated protein 1 | TJAP1 | 422 | T | 1 | 82.59 | 2.30 | 2.05 | NaN | 2.30 | 2.66 | 2.68 | 1.12 | 1.25 | NaN |
| Q5STZ8 | ATP-binding cassette sub-family F member 1 | ABCF1 | 109 | T | 1 | 123.2 | 1.17 | 0.45 | 2.28 | 1.61 | 1.56 | 1.51 | 1.76 | 0.55 | 0.54 |
| Q5T200 | Zinc finger CCCH domain-containing protein 13 | ZC3H13 | 263 | T | 1 | 78.99 | 0.82 | 0.84 | 0.91 | 0.82 | 0.88 | 1.04 | 0.81 | 0.98 | 0.75 |
| Q5T2Z0 | Proline/serine-rich coiled-coil protein 1 | PSRC1 | 177 | T | 0.812 | 56.71 | NaN | NaN | 0.71 | 0.67 | NaN | 0.74 | 0.90 | 1.05 | 0.96 |
| Q5VT52 | Regulation of nuclear pre-mRNA domain-containing protein 2 | RPRD2 | 723 | T | 0.997 | 57.21 | 1.19 | 1.27 | NaN | NaN | NaN | NaN | NaN | NaN | 2.13 |
| Q5VT52 | Regulation of nuclear pre-mRNA domain-containing protein 2 | RPRD2 | 732 | T | 0.993 | 117.3 | 0.43 | 0.51 | 0.62 | 0.80 | NaN | 0.82 | NaN | 0.33 | NaN |
| Q63ZY6 | Putative methyltransferase NSUN5C | NSUN5P2 | 258 | T | 0.986 | 42.63 | NaN | NaN | 0.59 | NaN | 1.12 | 0.72 | NaN | NaN | 0.60 |
| Q66K74 | Microtubule-associated protein 1S;MAP1S heavy chain;MAP1S light chain | MAP1S | 638 | T | 1 | 176 | 0.94 | 0.99 | 0.91 | 0.99 | 0.93 | 0.87 | 1.01 | 1.04 | 1.08 |
| Q6IQ49 | Protein SDE2 homolog | SDE2 | 274 | T | 0.545 | 78.98 | NaN | 1.08 | NaN | 1.15 | NaN | NaN | 0.97 | NaN | 1.41 |
| Q6NZI2 | Polymerase I and transcript release factor | PTRF | 376 | T | 0.85 | 131.5 | 0.57 | 0.48 | NaN | NaN | NaN | NaN | NaN | NaN | NaN |
| Q6PD62 | RNA polymerase-associated protein CTR9 homolog | CTR9 | 925 | T | 1 | 135.8 | 0.80 | 0.90 | 0.79 | 0.86 | 0.69 | 0.89 | 0.96 | 0.88 | 0.98 |
| Q6PKG0 | La-related protein 1 | LARP1 | 526 | T | 1 | 59.43 | 2.69 | 2.45 | 1.97 | NaN | 1.97 | 1.54 | 2.06 | 2.20 | NaN |
| Q6QNY0 | Biogenesis of lysosome-related organelles complex 1 | BLOC1S3 | 63 | T | 1 | 128.9 | 1.15 | 1.16 | 1.25 | 1.09 | 1.11 | 0.39 | 1.09 | 0.45 | 1.16 |
| Q6UN15 | Pre-mRNA 3'-end-processing factor FIP1 | FIP1L1 | 494 | T | 0.565 | 79.34 | NaN | NaN | NaN | NaN | NaN | NaN | 1.20 | 1.02 | NaN |
| Q6ZTN6 | Ankyrin repeat domain-containing protein 13D | ANKRD13D | 469 | T | 1 | 66.08 | 0.81 | 0.71 | NaN | 1.00 | 1.13 | 0.81 | 0.94 | 1.20 | 0.91 |
| Q7L4S7 | Protein ARMCX6 | ARMCX6 | 166 | T | 0.587 | 53.33 | 1.32 | 1.22 | NaN | NaN | NaN | NaN | NaN | NaN | NaN |
| Q7Z417 | Nuclear fragile X mental retardation-interacting protein | NUFIP2 | 571 | T | 0.889 | 93.84 | 1.86 | 1.55 | NaN | 1.80 | 1.64 | NaN | 1.43 | 0.86 | NaN |
| Q86TB9 | Protein PAT1 homolog 1 | PATL1 | 194 | T | 1 | 99.34 | 1.15 | 1.43 | 1.64 | 1.41 | 1.59 | 1.65 | 1.60 | 1.36 | 1.58 |
| Q86UP2 | Kinectin | KTN1 | 153 | T | 1 | 62.04 | 0.91 | NaN | NaN | 1.20 | NaN | 1.07 | 1.07 | 1.08 | NaN |
| Q86UU1 | Pleckstrin homology-like domain family B member 1 | PHLDB1 | 417 | T | 0.732 | 59.15 | 5.48 | 5.63 | 6.05 | NaN | 1.96 | 2.08 | 6.17 | NaN | 4.83 |
| Q8N3D4 | EH domain-binding protein 1-like protein 1 | EHBP1L1 | 284 | T | 0.616 | 180 | 1.00 | NaN | 0.98 | 1.15 | 1.13 | 1.03 | 0.96 | 0.83 | 0.82 |
| Q8NC44 | Protein FAM134A | FAM134A | 279 | T | 0.997 | 118.3 | NaN | 0.99 | 0.95 | NaN | 2.01 | 1.01 | 1.32 | 1.34 | 1.65 |
| Q8NC56 | LEM domain-containing protein 2 | LEMD2 | 147 | T | 1 | 131.1 | 0.77 | 0.78 | 0.94 | 0.88 | 1.04 | 0.98 | NaN | NaN | NaN |
| Q8ND56 | Protein LSM14 homolog A | LSM14A | 194 | T | 0.805 | 116.8 | NaN | 1.35 | NaN | NaN | NaN | NaN | 0.89 | NaN | 1.25 |

| | | | | | | | | | | | | | | | |
|---|---|---|---|---|---|---|---|---|---|---|---|---|---|---|---|
| Q8NI08 | Nuclear receptor coactivator 7 | NCOA7 | 210 | T | 0.673 | 116.6 | 0.88 | NaN | NaN | 1.06 | NaN | 0.87 | 0.94 | NaN | NaN |
| Q8WVC0 | RNA polymerase-associated protein LEO1 | LEO1 | 629 | T | 0.681 | 77.07 | 0.83 | 0.86 | 0.90 | NaN | 0.62 | 0.58 | NaN | NaN | 0.66 |
| Q8WVC0 | RNA polymerase-associated protein LEO1 | LEO1 | 188 | T | 1 | 56.47 | 0.68 | 0.46 | NaN | NaN | NaN | NaN | 0.66 | NaN | NaN |
| Q8WXX0 | Dynein heavy chain 7, axonemal | DNAH7 | 2197 | T | 0.989 | 72.88 | NaN | NaN | NaN | 0.94 | 0.76 | NaN | NaN | NaN | NaN |
| Q8WXX0 | Dynein heavy chain 7, axonemal | DNAH7 | 2198 | T | 0.989 | 72.88 | NaN | NaN | NaN | 0.94 | 0.76 | NaN | NaN | NaN | NaN |
| Q92688 | Acidic leucine-rich nuclear phosphoprotein 32 family member B | ANP32B | 244 | T | 1 | 251.9 | 0.93 | 0.96 | 0.96 | 0.96 | 0.97 | 0.80 | 0.94 | 0.95 | 0.96 |
| Q92692 | Nectin-2 | PVRL2 | 410 | T | 0.997 | 137.5 | 0.93 | 1.14 | 1.31 | 1.32 | 1.28 | 0.89 | 1.15 | 1.43 | 1.83 |
| Q92797 | Symplekin | SYMPK | 1257 | T | 1 | 120.7 | 0.67 | 0.85 | 0.62 | 1.11 | NaN | 1.07 | 0.78 | 0.67 | 0.84 |
| Q92995 | Ubiquitin carboxyl-terminal hydrolase 13 | USP13 | 122 | T | 1 | 105.5 | 0.66 | 0.68 | NaN | NaN | NaN | NaN | 0.42 | NaN | NaN |
| Q96A49 | Synapse-associated protein 1 | SYAP1 | 248 | T | 1 | 190.8 | NaN | NaN | 1.03 | 0.91 | 0.88 | 0.94 | 1.32 | 1.32 | 1.16 |
| Q96N67 | Dedicator of cytokinesis protein 7 | DOCK7 | 909 | T | 0.964 | 325.4 | NaN | 0.84 | 1.09 | 0.91 | NaN | 0.78 | 0.86 | NaN | NaN |
| Q96PK6 | RNA-binding protein 14 | RBM14 | 206 | T | 1 | 117.5 | 1.31 | 1.23 | 1.28 | 1.09 | 1.13 | 1.09 | 1.32 | 1.24 | 1.27 |
| Q96PX8 | SLIT and NTRK-like protein 1 | SLITRK1 | 590 | T | 0.918 | 79.04 | NaN | NaN | 1.05 | 0.91 | 0.70 | NaN | 1.17 | 1.14 | NaN |
| Q96T23 | Remodeling and spacing factor 1 | RSF1 | 628 | T | 0.599 | 116.9 | NaN | 0.62 | 0.69 | NaN | 0.95 | 0.93 | 0.98 | 1.11 | NaN |
| Q99442 | Translocation protein SEC62 | SEC62 | 158 | T | 0.972 | 74.53 | 1.39 | 1.37 | NaN | NaN | NaN | 0.86 | 1.25 | NaN | NaN |
| Q9BQA1 | Methylosome protein 50 | WDR77 | 5 | T | 1 | 48.11 | 1.17 | 1.28 | 1.19 | 1.30 | 1.18 | 1.37 | 1.10 | 1.00 | 1.13 |
| Q9BRD0 | BUD13 homolog | BUD13 | 159 | T | 1 | 58.78 | 1.42 | 1.92 | NaN | NaN | NaN | NaN | 2.23 | 2.11 | NaN |
| Q9BRD0 | BUD13 homolog | BUD13 | 135 | T | 1 | 71.56 | 1.47 | NaN | 1.90 | NaN | NaN | NaN | 1.76 | NaN | NaN |
| Q9BRQ0 | Pygopus homolog 2 | PYGO2 | 302 | T | 1 | 167.3 | 1.06 | 0.91 | 1.00 | NaN | NaN | 0.94 | 1.01 | 1.05 | 0.97 |
| Q9BRS8 | La-related protein 6 | LARP6 | 450 | T | 0.533 | 57.15 | NaN | NaN | NaN | NaN | NaN | NaN | 1.48 | 1.76 | 1.74 |
| Q9BTC0 | Death-inducer obliterator 1 | DIDO1 | 1255 | T | 0.841 | 41.59 | NaN | NaN | NaN | 1.10 | 1.24 | NaN | 0.95 | NaN | NaN |
| Q9BTC0 | Death-inducer obliterator 1 | DIDO1 | 1256 | T | 0.841 | 60.66 | NaN | NaN | NaN | 1.10 | 1.24 | NaN | 0.95 | NaN | NaN |
| Q9BU76 | Multiple myeloma tumor-associated protein 2 | MMTAG2 | 215 | T | 0.956 | 90.87 | 0.59 | 0.73 | NaN | NaN | 1.35 | NaN | 1.61 | NaN | NaN |
| Q9BU76 | Multiple myeloma tumor-associated protein 2 | MMTAG2 | 219 | T | 0.95 | 90.87 | 0.59 | 0.73 | NaN | 0.74 | 0.60 | NaN | NaN | 0.76 | NaN |
| Q9BXP5 | Serrate RNA effector molecule homolog | SRRT | 544 | T | 1 | 144.4 | 0.91 | 0.97 | 1.02 | 1.12 | 1.12 | 1.08 | 1.15 | 1.24 | 1.06 |
| Q9C0B5 | Palmitoyltransferase ZDHHC5 | ZDHHC5 | 524 | T | 0.568 | 60.76 | NaN | 1.56 | 1.68 | NaN | NaN | 1.25 | NaN | NaN | NaN |
| Q9H1E3 | Nuclear ubiquitous casein and cyclin-dependent kinase substrate 1 | NUCKS1 | 179 | T | 1 | 155.5 | 1.79 | 1.89 | 2.00 | 3.16 | 2.83 | 3.27 | 1.05 | 1.04 | 1.08 |
| Q9H1E3 | Nuclear ubiquitous casein and cyclin-dependent kinase substrate 1 | NUCKS1 | 202 | T | 1 | 144.7 | 0.78 | 1.23 | 1.27 | 1.07 | 0.89 | 1.17 | 0.96 | 1.03 | 1.37 |
| Q9H6F5 | Coiled-coil domain-containing protein 86 | CCDC86 | 65 | T | 0.874 | 64.04 | 0.74 | 0.65 | 0.62 | 0.77 | 0.74 | 0.83 | NaN | NaN | 0.55 |

| | | | | | | | | | | | | | | | |
|---|---|---|---|---|---|---|---|---|---|---|---|---|---|---|---|
| Q9H6F5 | Coiled-coil domain-containing protein 86 | CCDC86 | 42 | T | 0.998 | 122 | 1.12 | NaN | NaN | 1.21 | NaN | NaN | 1.00 | 1.01 | NaN |
| Q9H7N4 | Splicing factor, arginine/serine-rich 19 | SCAF1 | 989 | T | 1 | 177 | 0.77 | 0.86 | 0.78 | 1.30 | 1.03 | 0.83 | 0.64 | 0.66 | 1.26 |
| Q9H7N4 | Splicing factor, arginine/serine-rich 19 | SCAF1 | 1001 | T | 0.865 | 116.5 | NaN | NaN | NaN | 1.03 | NaN | 0.99 | NaN | NaN | NaN |
| Q9H7N4 | Splicing factor, arginine/serine-rich 19 | SCAF1 | 976 | T | 0.981 | 72.37 | 1.02 | 0.94 | 0.89 | NaN | 0.97 | 1.00 | 1.01 | 0.98 | NaN |
| Q9HA65 | TBC1 domain family member | TBC1D17 | 606 | T | 0.802 | 97.04 | NaN | NaN | 2.02 | NaN | NaN | NaN | 0.65 | 1.74 | NaN |
| Q9NQS7 | Inner centromere protein | INCENP | 219 | T | 0.787 | 150.5 | 1.18 | NaN | NaN | 2.16 | NaN | 1.83 | NaN | NaN | NaN |
| Q9NVD7 | Alpha-parvin | PARVA | 16 | T | 0.941 | 129.5 | NaN | 1.32 | 0.61 | NaN | 1.13 | 0.46 | 0.90 | 0.89 | 1.08 |
| Q9NWZ5 | Uridine-cytidine kinase-like 1 | UCKL1 | 53 | T | 0.86 | 125.4 | 1.26 | 1.23 | NaN | NaN | NaN | 1.88 | 0.91 | NaN | 0.83 |
| Q9P2Q2 | FERM domain-containing protein 4A | FRMD4A | 696 | T | 0.5 | 79.07 | NaN | NaN | NaN | NaN | 0.74 | 1.52 | NaN | 0.86 | NaN |
| Q9UIQ6 | Leucyl-cystinyl aminopeptidase;Leucyl-cystinyl aminopeptidase, pregnancy serum form | LNPEP | 326 | T | 0.644 | 45.92 | 0.64 | NaN | 0.65 | 0.66 | 0.80 | 0.68 | NaN | 0.60 | 0.91 |
| Q9UK61 | Protein FAM208A | FAM208A | 921 | T | 0.598 | 53.78 | 0.50 | 0.50 | NaN | NaN | NaN | NaN | NaN | NaN | 0.95 |
| Q9UK76 | Hematological and neurological expressed 1 protein;Hematological and neurological expressed 1 protein, N-terminally | HN1 | 54 | T | 1 | 183.8 | 5.27 | 5.14 | 4.83 | 9.89 | 8.91 | 10.25 | NaN | 2.08 | NaN |
| Q9UKJ3 | G patch domain-containing protein 8 | GPATCH 8 | 655 | T | 0.626 | 69.74 | NaN | NaN | NaN | 1.50 | NaN | 1.27 | NaN | NaN | 0.94 |
| Q9UKK3 | Poly [ADP-ribose] polymerase | PARP4 | 1493 | T | 0.5 | 129 | 0.83 | 0.65 | 0.75 | 0.74 | 0.70 | 0.86 | 1.01 | 1.03 | 0.99 |
| Q9UKK3 | Poly [ADP-ribose] polymerase | PARP4 | 1494 | T | 0.5 | 129 | 0.83 | 0.65 | 0.75 | 0.74 | 0.70 | 0.86 | 1.01 | 1.03 | 0.99 |
| Q9UQ35 | Serine/arginine repetitive matrix protein 2 | SRRM2 | 1492 | T | 1 | 73.63 | NaN | 1.25 | NaN | NaN | NaN | NaN | 1.81 | NaN | 2.06 |
| Q9UQ35 | Serine/arginine repetitive matrix protein 2 | SRRM2 | 1003 | T | 1 | 181 | 0.55 | 0.60 | 0.50 | 1.37 | 0.71 | 0.74 | 0.83 | 0.67 | 0.85 |
| Q9UQ35 | Serine/arginine repetitive matrix protein 2 | SRRM2 | 1434 | T | 1 | 72.53 | 1.75 | 1.78 | 1.78 | 1.46 | 1.59 | 1.57 | 1.77 | 1.65 | 1.61 |
| Q9UQ35 | Serine/arginine repetitive matrix protein 2 | SRRM2 | 1208 | T | 1 | 110.4 | 1.05 | 1.14 | 1.14 | 1.06 | 1.18 | 1.10 | 1.13 | 1.10 | 1.14 |
| Q9UQ35 | Serine/arginine repetitive matrix protein 2 | SRRM2 | 1231 | T | 0.708 | 77.85 | NaN | 1.05 | 0.97 | NaN | NaN | NaN | NaN | NaN | NaN |
| Q9UQ35 | Serine/arginine repetitive matrix protein 2 | SRRM2 | 2738 | T | 1 | 51.03 | 1.11 | 1.09 | NaN | NaN | NaN | NaN | NaN | NaN | NaN |
| Q9UQ35 | Serine/arginine repetitive matrix protein 2 | SRRM2 | 383 | T | 0.778 | 96.79 | NaN | 1.75 | 1.53 | 1.33 | NaN | NaN | NaN | NaN | NaN |
| Q9UQ35 | Serine/arginine repetitive matrix protein 2 | SRRM2 | 1177 | T | 0.726 | 79.02 | 0.59 | 0.62 | 0.72 | 0.80 | NaN | NaN | NaN | NaN | 0.83 |
| Q9UQ35 | Serine/arginine repetitive matrix protein 2 | SRRM2 | 2104 | T | 1 | 98 | 1.00 | 1.00 | 1.21 | 1.01 | 0.93 | 1.07 | 1.12 | 1.18 | NaN |

| | | | | | | | | | | | | | | |
|---|---|---|---|---|---|---|---|---|---|---|---|---|---|---|
| Q9UQ35 | Serine/arginine repetitive matrix protein 2 | SRRM2 | 856 | T | 0.812 | 115.8 | 1.35 | 1.81 | 0.68 | NaN | NaN | NaN | 2.11 | 0.74 | 2.32 |
| Q9UQ35 | Serine/arginine repetitive matrix protein 2 | SRRM2 | 866 | T | 0.989 | 119.2 | 2.66 | 1.81 | 1.85 | 2.15 | 1.58 | 1.60 | 2.45 | 2.85 | 2.34 |
| Q9UQ35 | Serine/arginine repetitive matrix protein 2 | SRRM2 | 2583 | T | 0.978 | 66.5 | 0.94 | NaN | 1.06 | 0.98 | NaN | 0.97 | 1.06 | NaN | 1.11 |
| Q9UQ35 | Serine/arginine repetitive matrix protein 2 | SRRM2 | 2092 | T | 0.985 | 56.01 | NaN | NaN | 0.44 | 0.58 | NaN | 0.63 | NaN | NaN | 0.44 |
| Q9UQ35 | Serine/arginine repetitive matrix protein 2 | SRRM2 | 848 | T | 0.917 | 115.8 | 0.89 | 0.83 | 0.83 | 0.84 | 0.82 | 1.60 | 0.73 | 0.74 | 0.72 |
| Q9UQ35 | Serine/arginine repetitive matrix protein 2 | SRRM2 | 2069 | T | 1 | 68.46 | 2.36 | 2.46 | 2.63 | 2.00 | 1.96 | NaN | 0.28 | 2.61 | NaN |
| Q9UQ35 | Serine/arginine repetitive matrix protein 2 | SRRM2 | 2022 | T | 1 | 76.85 | 1.36 | 1.42 | 1.34 | 1.17 | 1.22 | 1.20 | 1.42 | 1.43 | 1.48 |
| Q9UQ35 | Serine/arginine repetitive matrix protein 2 | SRRM2 | 2409 | T | 1 | 106.3 | 0.91 | 1.07 | 0.88 | 0.91 | 0.98 | 0.81 | 1.08 | 0.97 | 0.89 |
| Q9UQ35 | Serine/arginine repetitive matrix protein 2 | SRRM2 | 1927 | T | 1 | 72.58 | 1.37 | 1.38 | 1.27 | 1.19 | NaN | 1.20 | 1.45 | 1.42 | 1.52 |
| Q9UQ35 | Serine/arginine repetitive matrix protein 2 | SRRM2 | 1043 | T | 0.987 | 157.4 | 0.88 | 0.80 | 0.76 | 0.97 | 0.81 | 0.75 | 0.99 | 0.93 | 0.93 |
| Q9UQ35 | Serine/arginine repetitive matrix protein 2 | SRRM2 | 903 | T | 0.903 | 93.39 | 0.97 | 0.91 | 1.07 | NaN | 0.85 | 0.84 | 1.06 | NaN | NaN |
| Q9UQ35 | Serine/arginine repetitive matrix protein 2 | SRRM2 | 1527 | T | 0.862 | 62.34 | 2.57 | NaN | 1.78 | 1.47 | 1.57 | NaN | 2.41 | NaN | NaN |
| Q9UQ35 | Serine/arginine repetitive matrix protein 2 | SRRM2 | 983 | T | 1 | 173.4 | 0.82 | 0.80 | 0.86 | 0.81 | 0.85 | 0.88 | 0.87 | 1.05 | 0.81 |
| Q9Y3P9 | Rab GTPase-activating protein 1 | RABGAP 1 | 996 | T | 1 | 163.9 | 1.03 | 0.84 | 0.66 | 0.77 | 0.81 | 0.54 | 0.66 | 0.89 | 0.67 |
| Q9Y4B5 | Microtubule cross-linking factor 1 | MTCL1 | 1417 | T | 1 | 80.53 | 0.84 | 0.90 | NaN | 0.99 | 0.97 | NaN | NaN | 0.89 | NaN |
| Q9Y4D8 | Probable E3 ubiquitin-protein ligase HECTD4 | HECTD4 | 2080 | T | 0.999 | 69.03 | 0.69 | 0.76 | 0.70 | 0.86 | NaN | 0.88 | 1.02 | 0.80 | 0.93 |
| Q9Y4H2 | Insulin receptor substrate 2 | IRS2 | 1202 | T | 0.832 | 183.2 | 2.45 | NaN | 2.40 | 1.37 | NaN | 1.41 | 2.38 | 1.73 | 1.79 |
| Q9Y4H2 | Insulin receptor substrate 2 | IRS2 | 779 | T | 0.946 | 56.9 | NaN | NaN | NaN | 1.45 | NaN | 1.29 | NaN | NaN | NaN |
| G3V317 | Cyclin-dependent kinase 1;Cyclin-dependent kinase 2;Cyclin-dependent kinase 3 | CDK2 | 15 | Y | 1 | 100.8 | NaN | 0.91 | NaN | NaN | 1.47 | 1.50 | NaN | 0.69 | 0.71 |
| A0A087WY22 | Protein-tyrosine-phosphatase;Receptor-type tyrosine-protein phosphatase | PTPRT | 1045 | Y | 1 | 63.73 | NaN | 0.80 | NaN | 0.78 | 0.74 | NaN | NaN | NaN | 0.91 |
| A0A087WTN1 | Uncharacterized protein C9orf173 | C9orf173 | 76 | Y | 0.864 | 46.92 | NaN | NaN | NaN | NaN | 0.78 | 0.77 | NaN | NaN | 0.82 |
| A0A087WWY2 | | FOLR2 | 144 | Y | 0.991 | 48.11 | 0.80 | 0.67 | NaN | NaN | 0.73 | NaN | 0.81 | 0.98 | NaN |
| A0A087X0K9 | Tight junction protein ZO-1 | TJP1 | 120;132 | Y | 0.703 | 77.04 | NaN | NaN | NaN | 1.80 | 1.42 | NaN | 1.52 | NaN | NaN |
| A0A087X0K9 | Tight junction protein ZO-1 | TJP1 | 1440;153[illegible] | Y | 0.527 | 42.81 | 0.32 | 0.57 | NaN | NaN | NaN | NaN | NaN | NaN | NaN |
| A0A096LP07 | | GPS1 | 207 | Y | 0.722 | 47.13 | NaN | NaN | 1.60 | 1.42 | 1.41 | 1.36 | 1.54 | 1.52 | NaN |
| A0A096LP07 | | GPS1 | 217 | Y | 0.743 | 47.13 | NaN | NaN | 1.60 | 1.42 | 1.41 | 1.36 | 1.54 | 1.52 | NaN |

| | | | | | | | | | | | | | | | |
|---|---|---|---|---|---|---|---|---|---|---|---|---|---|---|---|
| A0A0A0MRA3 | Titin | TTN | 25698 | Y | 1 | 59.26 | NaN | NaN | 0.78 | 0.69 | 0.61 | 0.71 | 1.11 | 1.02 | 0.95 |
| A0A0A0MRZ4 | TNFAIP3-interacting protein 1 | TNIP1 | 7 | Y | 0.506 | 50.23 | 2.02 | 1.69 | NaN | 2.72 | 3.58 | NaN | NaN | 0.76 | NaN |
| A0A0A0MRZ4 | TNFAIP3-interacting protein 1 | TNIP1 | 10 | Y | 0.506 | 50.23 | 2.02 | 1.69 | NaN | 2.72 | 3.58 | NaN | NaN | 0.76 | NaN |
| A0A0D9SEV1 | | KCNQ2 | 566 | Y | 1 | 43.99 | NaN | NaN | 1.49 | 1.34 | 1.29 | 1.02 | 1.07 | 1.20 | 1.14 |
| F5GZ78 | Paxillin | PXN | 116 | Y | 0.923 | 81.8 | 2.09 | 2.28 | NaN | 1.61 | 1.67 | 1.38 | 0.91 | NaN | NaN |
| A0A2R8Y6M1 | ATP-binding cassette sub-family G member 8 | ABCG8 | 325 | Y | 0.998 | 45.72 | NaN | NaN | 1.05 | NaN | 0.83 | 0.83 | NaN | 1.24 | 1.04 |
| A0A2R8YEM9 | TPR and ankyrin repeat-containing protein 1 | TRANK1 | 1677 | Y | 0.999 | 40.29 | NaN | NaN | NaN | 1.12 | 0.95 | NaN | NaN | NaN | NaN |
| A8MT37 | Glycogen synthase kinase-3 beta;Glycogen synthase kinase-3 alpha | GSK3A | 197 | Y | 0.987 | 163.5 | NaN | NaN | 0.76 | NaN | 0.71 | 0.78 | 1.16 | 1.16 | 1.26 |
| A0A3B3IUF8 | Rho guanine nucleotide exchange factor 15 | ARHGEF 15 | 253 | Y | 0.657 | 47.22 | 1.43 | 1.63 | NaN | NaN | 1.41 | NaN | 0.89 | 1.57 | NaN |
| A6NCQ9 | RING finger protein 222 | RNF222 | 66 | Y | 0.784 | 47.54 | NaN | NaN | 1.44 | 1.27 | 0.73 | NaN | 0.72 | 0.91 | NaN |
| P07355 | Annexin A2;Putative annexin A2-like protein;Annexin | ANXA2 | 24 | Y | 0.896 | 242.1 | 0.60 | 0.68 | 0.77 | 0.82 | NaN | 0.94 | 0.75 | 0.76 | NaN |
| E7EX54 | Mitogen-activated protein kinase 14 | MAPK14 | 105 | Y | 0.71 | 101.6 | NaN | NaN | NaN | NaN | NaN | NaN | 1.00 | NaN | 0.88 |
| D6R9P1 | Ectonucleotide pyrophosphatase/phosphodiesterase family member 6 | ENPP6 | 131 | Y | 0.976 | 46.88 | NaN | NaN | NaN | 0.75 | 0.89 | NaN | NaN | NaN | NaN |
| H0YDD4 | Acetyltransferase component of pyruvate dehydrogenase complex;Dihydrolipoyllysine-residue acetyltransferase component of pyruvate dehydrogenase complex, mitochondrial | DLAT | 278 | Y | 0.599 | 40.09 | NaN | NaN | NaN | 0.95 | 1.17 | NaN | NaN | NaN | NaN |
| H0YE41 | Tyrosine-protein kinase;Non-receptor tyrosine-protein kinase TYK2 | TYK2 | 71 | Y | 1 | 93.26 | NaN | NaN | NaN | NaN | NaN | NaN | 1.41 | 1.27 | 1.59 |
| F5GZJ9 | | PPP1R32 | 26 | Y | 1 | 41.62 | 0.46 | 0.56 | NaN | NaN | NaN | NaN | 0.86 | 0.74 | NaN |
| F5H7D6 | Transducin-like enhancer protein 3 | TLE3 | 182 | Y | 0.949 | 95.34 | NaN | NaN | NaN | 0.44 | 0.39 | NaN | NaN | NaN | 1.14 |
| F5H7P5 | Palmitoyltransferase;Probable palmitoyltransferase ZDHHC11B | ZDHHC11 B | 282 | Y | 0.911 | 54.74 | NaN | NaN | NaN | 1.37 | NaN | NaN | 1.50 | 1.48 | 1.45 |
| F6VUX8 | Centromere protein J | CENPJ | 980 | Y | 1 | 44.83 | 0.70 | 0.95 | 1.14 | 1.00 | 0.55 | NaN | 1.28 | 0.81 | NaN |
| F8VXW2 | | ARPC1B | 124 | Y | 0.998 | 41.05 | 1.25 | 1.39 | NaN | 1.11 | NaN | 0.84 | 1.29 | 1.68 | NaN |
| F8W6Z3 | | ODF3 | 51 | Y | 0.991 | 41.08 | NaN | NaN | NaN | NaN | NaN | NaN | 2.31 | 1.26 | NaN |
| H0YGI1 | Uncharacterized protein C12orf56 | C12orf56 | 39 | Y | 0.999 | 40.29 | NaN | NaN | NaN | NaN | NaN | NaN | 0.50 | 0.81 | NaN |
| H7C1A6 | | ADCY3 | 10 | Y | 0.951 | 40.55 | 1.07 | NaN | NaN | 1.02 | 1.10 | NaN | 1.29 | NaN | NaN |
| H7C1A6 | | ADCY3 | 11 | Y | 0.999 | 40.55 | 1.07 | NaN | NaN | 1.02 | 1.10 | NaN | 1.29 | NaN | NaN |
| J3KSR8 | Serine/arginine-rich splicing factor 1 | SRSF1 | 97 | Y | 0.924 | 54.95 | NaN | NaN | 1.27 | 1.48 | 1.34 | 1.27 | 1.45 | 1.29 | NaN |

| | | | | | | | | | | | | | | | | |
|---|---|---|---|---|---|---|---|---|---|---|---|---|---|---|---|---|
| P49448 | Glutamate dehydrogenase 2, mitochondrial;Glutamate dehydrogenase 1, | GLUD2 | 170 | Y | 0.937 | 43.79 | NaN | NaN | NaN | NaN | 1.23 | 1.10 | 1.17 | 1.08 | 0.97 |
| P28482 | Mitogen-activated protein kinase 1 | MAPK1 | 187 | Y | 1 | 250.5 | 1.99 | 2.07 | 1.93 | 1.93 | 1.84 | 1.75 | 1.11 | 1.16 | 1.08 |
| Q13523 | Serine/threonine-protein kinase PRP4 homolog | PRPF4B | 849 | Y | 0.986 | 261.3 | 1.10 | 1.07 | 1.09 | 1.17 | 1.12 | 1.17 | 1.15 | 1.14 | 1.10 |
| Q13541 | Eukaryotic translation initiation factor 4E-binding protein 1 | EIF4EBP1 | 34 | Y | 0.72 | 81.21 | NaN | 2.29 | 2.19 | 2.01 | NaN | 2.06 | 2.05 | NaN | 2.05 |
| Q13595 | Transformer-2 protein homolog alpha | TRA2A | 87 | Y | 0.671 | 61.44 | 1.08 | NaN | 0.97 | 0.90 | 0.83 | 0.94 | 1.32 | 1.24 | 1.25 |
| Q8N2E6 | Prosalusin;Salusin-alpha;Salusin-beta;Torsin-2A | TOR2A | 185 | Y | 0.605 | 42.68 | 0.96 | 0.78 | 1.08 | 0.90 | 0.70 | 0.97 | NaN | 0.73 | NaN |
| Q8N2E6 | Prosalusin;Salusin-alpha;Salusin-beta;Torsin-2A | TOR2A | 189 | Y | 0.992 | 42.68 | 0.96 | 0.78 | 1.08 | 0.90 | 0.70 | 0.97 | NaN | 0.73 | NaN |
| Q68DU9 | Kelch-like protein 8 | DKFZp78 1C1855 | 353 | Y | 0.999 | 43.18 | NaN | NaN | NaN | 0.96 | 1.13 | 1.21 | NaN | NaN | 1.04 |
| Q6PI26 | Protein SHQ1 homolog | SHQ1 | 344 | Y | 1 | 43.59 | NaN | NaN | NaN | NaN | NaN | NaN | 1.05 | 0.85 | NaN |
| Q86YV5 | Tyrosine-protein kinase SgK223 | SGK223 | 413 | Y | 0.926 | 57.29 | 1.23 | 0.86 | NaN | NaN | 0.66 | NaN | NaN | NaN | NaN |
| Q96M20 | Cyclic nucleotide-binding domain-containing protein 2 | CNBD2 | 302 | Y | 0.971 | 45.88 | NaN | NaN | NaN | 1.56 | 2.72 | NaN | NaN | NaN | NaN |
| Q9NWQ8 | Phosphoprotein associated with glycosphingolipid-enriched microdomains 1 | PAG1 | 417 | Y | 1 | 124.5 | NaN | NaN | NaN | 0.75 | 0.52 | NaN | NaN | 1.13 | NaN |
| Q9NYQ8 | Protocadherin Fat 2 | FAT2 | 1877 | Y | 0.994 | 43.8 | NaN | NaN | NaN | 0.89 | 0.93 | 0.95 | 0.40 | 0.46 | NaN |
| Q9UIQ6 | Leucyl-cystinyl aminopeptidase;Leucyl-cystinyl aminopeptidase, pregnancy serum form | LNPEP | 325 | Y | 0.73 | 45.92 | 0.64 | NaN | 0.65 | 0.66 | 0.80 | 0.68 | NaN | 0.60 | 0.91 |

Supplementary Table S3 The Bonding Energy of SFN docking to RAFs

| Software | | | Autodock4 [1] | Autodock Vina [2] | DockThor [3] |
|---|---|---|---|---|---|
| Protein | PDB id | ligand | Bonding energy (Kcal/mol) | Bonding energy (Kcal/mol) | Affinity (Kcal/mol) |
| A-RAF | 1wxm | R,S-SFN | -2.8 | -3.78 | -6.909 |
| | | R-SFN | -3.0 | -3.69 | -6.988 |
| | | S-SFN | -2.8 | -4.37 | -6.909 |
| B-RAF | 6p3d | R,S-SFN | -3.7 | -5.19 | -7.139 |
| | | R-SFN | -4.0 | -5.12 | -7.139 |
| | | S-SFN | -4.0 | -5.47 | -7.144 |
| C-RAF | 3omv | R,S-SFN | -3.8 | -5.0 | -7.522 |
| | | R-SFN | -3.0 | -5.34 | -7.421 |
| | | S-SFN | -3.4 | -5.27 | -6.849 |

References


[1] Morris, G.M. et al. AutoDock4 and AutoDockTools4: Automated docking with selective receptor flexibility. *J Comput Chem* **30,** 2785-2791 (2009).

[2] Trott, O., & Olson, A.J. AutoDock Vina: improving the speed and accuracy of docking with a new scoring function, efficient optimization, and multithreading. *J Comput Chem* **31,** 455-461 (2010).

[3] Santos, K.B., Guedes, I.A., Karl, A.L.M., & Dardenne, L.E. Highly Flexible Ligand Docking: Benchmarking of the DockThor Program on the LEADS-PEP Protein-Peptide Data Set. *J Chem Inf Model* **60,** 667-683 (2020).

# Supplementary Figure S1

Uncropped western blots presented in Figure 7A are shown.

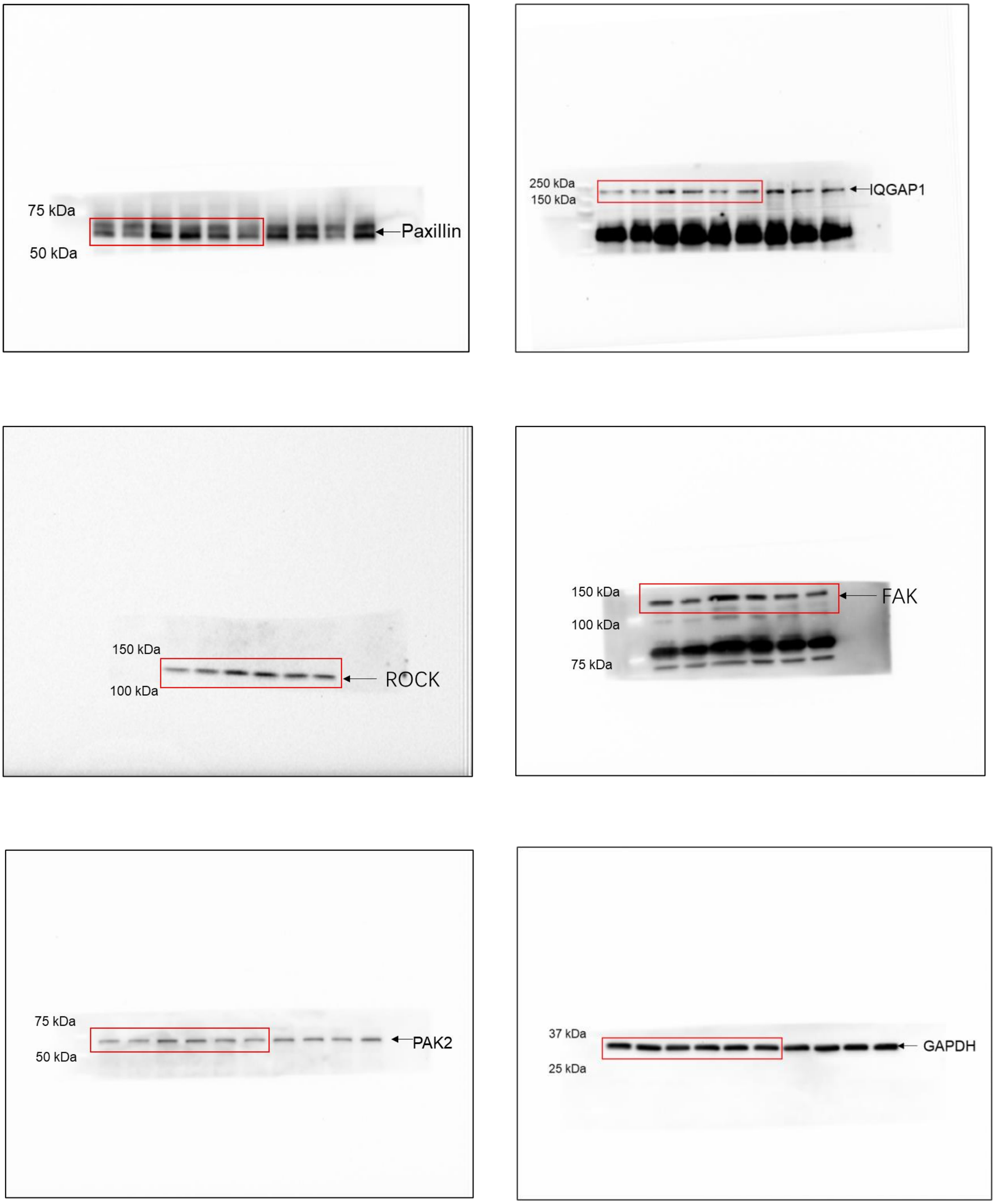

# Supplementary Figure S1

Uncropped western blots presented in Figure 7B are shown.

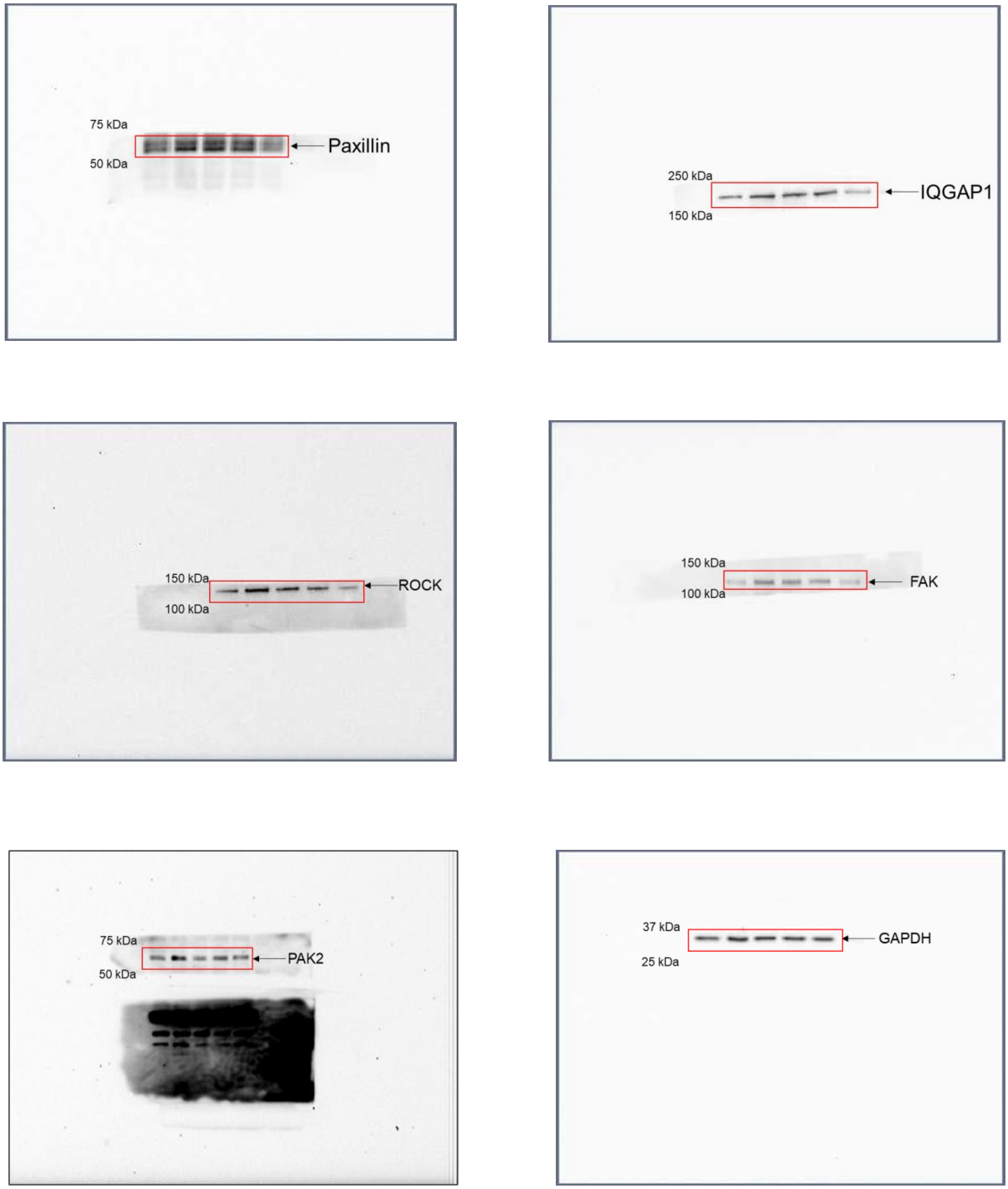

# Supplementary Figure S1

Uncropped western blots presented in Figure 8A are shown.

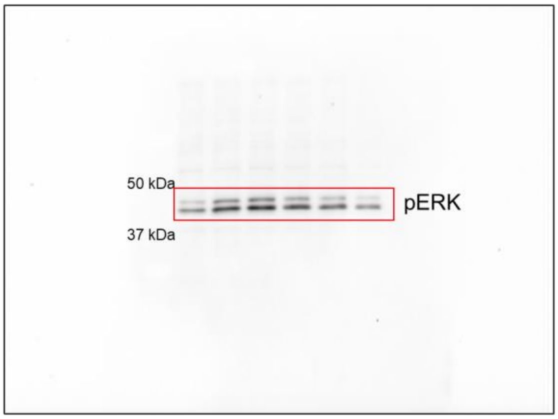


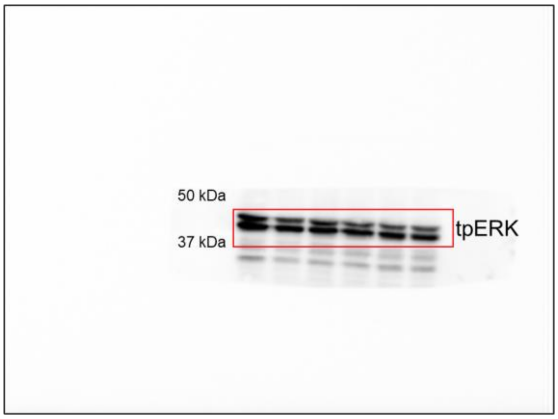


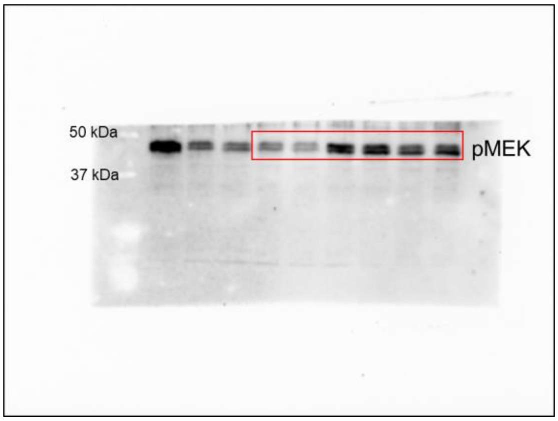


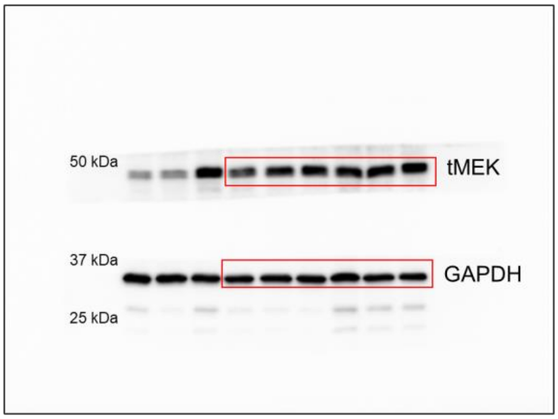

Supplement: Supplementary file 1 — Supplementary material [file 41523_2022_402_MOESM1_ESM.pdf]
