# Supplementary material for: Sulforaphane suppresses metastasis of triple-negative breast cancer cells by targeting the RAF/MEK/ERK pathway
Source: NPJ Breast Cancer. 2022 Mar 24;8:40. doi: 10.1038/s41523-022-00402-4 (PMC8948359; doi:10.1038/s41523-022-00402-4)
Supplement: Supplementary file 2 — Related Manuscript File [file 41523_2022_402_MOESM2_ESM.pdf]

nature portfolio

Corresponding author(s): DBPR NPJBCANCER-01392R

Last updated by author(s): Nov 15, 2021

Reporting Summary

Nature Portfolio wishes to improve the reproducibility of the work that we publish. This form provides structure for consistency and transparency in reporting. For further information on Nature Portfolio policies, see our Editorial Policies and the Editorial Policy Checklist.

Statistics

For all statistical analyses, confirm that the following items are present in the figure legend, table legend, main text, or Methods section.

n/a	Confirmed	
☐	☒	The exact sample size (n) for each experimental group/condition, given as a discrete number and unit of measurement
☐	☒	A statement on whether measurements were taken from distinct samples or whether the same sample was measured repeatedly
☐	☒	The statistical test(s) used AND whether they are one- or two-sided<br>*Only common tests should be described solely by name; describe more complex techniques in the Methods section.*
☒	☐	A description of all covariates tested
☒	☐	A description of any assumptions or corrections, such as tests of normality and adjustment for multiple comparisons
☒	☐	A full description of the statistical parameters including central tendency (e.g. means) or other basic estimates (e.g. regression coefficient) AND variation (e.g. standard deviation) or associated estimates of uncertainty (e.g. confidence intervals)
☒	☐	For null hypothesis testing, the test statistic (e.g. F, t, r) with confidence intervals, effect sizes, degrees of freedom and P value noted<br>*Give P values as exact values whenever suitable.*
☒	☐	For Bayesian analysis, information on the choice of priors and Markov chain Monte Carlo settings
☒	☐	For hierarchical and complex designs, identification of the appropriate level for tests and full reporting of outcomes
☒	☐	Estimates of effect sizes (e.g. Cohen's d, Pearson's r), indicating how they were calculated

Our web collection on statistics for biologists contains articles on many of the points above.

Software and code

Policy information about availability of computer code

Data collection	DockThor database,PubChem , Protein Data Bank
Data analysis	GraphPad Prism 8.0.2

For manuscripts utilizing custom algorithms or software that are central to the research but not yet described in published literature, software must be made available to editors and reviewers. We strongly encourage code deposition in a community repository (e.g. GitHub). See the Nature Portfolio guidelines for submitting code & software for further information.

Data

Policy information about availability of data

All manuscripts must include a data availability statement. This statement should provide the following information, where applicable:

- Accession codes, unique identifiers, or web links for publicly available datasets
- A description of any restrictions on data availability
- For clinical datasets or third party data, please ensure that the statement adheres to our policy

All data generated or analysed during this study are included in this published article (and its supplementary information files).

Field-specific reporting

Please select the one below that is the best fit for your research. If you are not sure, read the appropriate sections before making your selection.

☒ Life sciences ☐ Behavioural & social sciences ☐ Ecological, evolutionary & environmental sciences

For a reference copy of the document with all sections, see nature.com/documents/nr-reporting-summary-flat.pdf

Life sciences study design

All studies must disclose on these points even when the disclosure is negative.

Sample size	All experiments were performed on at least three independent occasions.
Data exclusions	No data were excluded from the analyses.
Replication	All attempts at replication were successful.
Randomization	This is not relevant to my study.
Blinding	This is not relevant to my study.

Reporting for specific materials, systems and methods

We require information from authors about some types of materials, experimental systems and methods used in many studies. Here, indicate whether each material, system or method listed is relevant to your study. If you are not sure if a list item applies to your research, read the appropriate section before selecting a response.

Materials & experimental systems

n/a	Involved in the study	
☐	☒	Antibodies
☐	☒	Eukaryotic cell lines
☒	☐	Palaeontology and archaeology
☒	☐	Animals and other organisms
☒	☐	Human research participants
☒	☐	Clinical data
☒	☐	Dual use research of concern

Methods

n/a	Involved in the study	
☒	☐	ChIP-seq
☒	☐	Flow cytometry
☒	☐	MRI-based neuroimaging

Antibodies

Antibodies used	The following antibodies were used: (1) anti-paxillin antibody (BD Biosciences, CA, USA,1:5,000), (2) anti-GAPDH monoclonal antibody (Wako, Osaka, Japan, 1:10,000), (3) anti-Rho-kinase II antibody (BD Biosciences, CA, USA, 1:500), (4) anti-IQGAP1 antibody (H-109, Santa Cruz, Dallas, USA, 1:500), (5) anti-FAK antibody (Sigma-Aldrich, St. Louis, MO, 1:500), (6) anti-PAK2 antibody (Cosmo Bio Co., LTD, Tokyo, Japan,1:200), (7) anti-phospho-MEK1/2 (Ser217/221) (41G9) rabbit antibody (Cell Signaling, MA, USA, 1:1,000), (8) anti-MEK antibody (Cell Signaling, MA, USA, 1:1,000), (9) anti-phospho-p44/42 Erk1/2 (Thr202/Tyr204) antibody (Cell Signaling, MA, USA, 1:1,000), (10) anti-ERK antibody (Cell Signaling, MA, USA, 1:1,000), (11) Secondary HRP-labeled antibodies (anti-mouse and anti-rabbit, Promega, 1:5,000), (12) Alexa Fluor-conjugated secondary antibodies for immunofluorescence imaging (Molecular Probes, Eugene, OR, 1:500)
Validation	Antibodies were used according to the manufacturer' datasheet.

Eukaryotic cell lines

Policy information about cell lines

Cell line source(s)	MDA-MB-231 cell line from Ds Pharma Biomedical; MDA-MB-157 cell line from ATCC
Authentication	None of the cell lines used were authenticated.
Mycoplasma contamination	All cell lines tested negative for mycoplasma contamination.
Commonly misidentified lines (See ICLAC register)	N/A
